# AI is a viable alternative to high throughput screening: a 318-target study

**DOI:** 10.1038/s41598-024-54655-z

**Published:** 2024-04-02

**Authors:** The Atomwise AIMS Program, Izhar Wallach, Denzil Bernard, Kong Nguyen, Gregory Ho, Adrian Morrison, Adrian Stecula, Andreana Rosnik, Ann Marie O’Sullivan, Aram Davtyan, Ben Samudio, Bill Thomas, Brad Worley, Brittany Butler, Christian Laggner, Desiree Thayer, Ehsan Moharreri, Greg Friedland, Ha Truong, Henry van den Bedem, Ho Leung Ng, Kate Stafford, Krishna Sarangapani, Kyle Giesler, Lien Ngo, Michael Mysinger, Mostafa Ahmed, Nicholas J. Anthis, Niel Henriksen, Pawel Gniewek, Sam Eckert, Saulo de Oliveira, Shabbir Suterwala, Srimukh Veccham Krishna PrasadPrasad, Stefani Shek, Stephanie Contreras, Stephanie Hare, Teresa Palazzo, Terrence E. O’Brien, Tessa Van Grack, Tiffany Williams, Ting-Rong Chern, Victor Kenyon, Andreia H. Lee, Andrew B. Cann, Bastiaan Bergman, Brandon M. Anderson, Bryan D. Cox, Jeffrey M. Warrington, Jon M. Sorenson, Joshua M. Goldenberg, Matthew A. Young, Nicholas DeHaan, Ryan P. Pemberton, Stefan Schroedl, Tigran M. Abramyan, Tushita Gupta, Venkatesh Mysore, Adam G. Presser, Adolfo A. Ferrando, Adriano D. Andricopulo, Agnidipta Ghosh, Aicha Gharbi Ayachi, Aisha Mushtaq, Ala M. Shaqra, Alan Kie Leong Toh, Alan V. Smrcka, Alberto Ciccia, Aldo Sena de Oliveira, Aleksandr Sverzhinsky, Alessandra Mara de Sousa, Alexander I. Agoulnik, Alexander Kushnir, Alexander N. Freiberg, Alexander V. Statsyuk, Alexandre R. Gingras, Alexei Degterev, Alexey Tomilov, Alice Vrielink, Alisa A. Garaeva, Amanda Bryant-Friedrich, Amedeo Caflisch, Amit K. Patel, Amith Vikram Rangarajan, An Matheeussen, Andrea Battistoni, Andrea Caporali, Andrea Chini, Andrea Ilari, Andrea Mattevi, Andrea Talbot Foote, Andrea Trabocchi, Andreas Stahl, Andrew B. Herr, Andrew Berti, Andrew Freywald, Andrew G. Reidenbach, Andrew Lam, Andrew R. Cuddihy, Andrew White, Angelo Taglialatela, Anil K. Ojha, Ann M. Cathcart, Anna A. L. Motyl, Anna Borowska, Anna D’Antuono, Anna K. H. Hirsch, Anna Maria Porcelli, Anna Minakova, Anna Montanaro, Anna Müller, Annarita Fiorillo, Anniina Virtanen, Anthony J. O’Donoghue, Antonio Del Rio Flores, Antonio E. Garmendia, Antonio Pineda-Lucena, Antonito T. Panganiban, Ariela Samantha, Arnab K. Chatterjee, Arthur L. Haas, Ashleigh S. Paparella, Ashley L. St. John, Ashutosh Prince, Assmaa ElSheikh, Athena Marie Apfel, Audrey Colomba, Austin O’Dea, Bakary N’tji Diallo, Beatriz Murta Rezende Moraes Ribeiro, Ben A. Bailey-Elkin, Benjamin L. Edelman, Benjamin Liou, Benjamin Perry, Benjamin Soon Kai Chua, Benjámin Kováts, Bernhard Englinger, Bijina Balakrishnan, Bin Gong, Bogos Agianian, Brandon Pressly, Brenda P. Medellin Salas, Brendan M. Duggan, Brian V. Geisbrecht, Brian W. Dymock, Brianna C. Morten, Bruce D. Hammock, Bruno Eduardo Fernandes Mota, Bryan C. Dickinson, Cameron Fraser, Camille Lempicki, Carl D. Novina, Carles Torner, Carlo Ballatore, Carlotta Bon, Carly J. Chapman, Carrie L. Partch, Catherine T. Chaton, Chang Huang, Chao-Yie Yang, Charlene M. Kahler, Charles Karan, Charles Keller, Chelsea L. Dieck, Chen Huimei, Chen Liu, Cheryl Peltier, Chinmay Kumar Mantri, Chinyere Maat Kemet, Christa E. Müller, Christian Weber, Christina M. Zeina, Christine S. Muli, Christophe Morisseau, Cigdem Alkan, Clara Reglero, Cody A. Loy, Cornelia M. Wilson, Courtney Myhr, Cristina Arrigoni, Cristina Paulino, César Santiago, Dahai Luo, Damon J. Tumes, Daniel A. Keedy, Daniel A. Lawrence, Daniel Chen, Danny Manor, Darci J. Trader, David A. Hildeman, David H. Drewry, David J. Dowling, David J. Hosfield, David M. Smith, David Moreira, David P. Siderovski, David Shum, David T. Krist, David W. H. Riches, Davide Maria Ferraris, Deborah H. Anderson, Deirdre R. Coombe, Derek S. Welsbie, Di Hu, Diana Ortiz, Dina Alramadhani, Dingqiang Zhang, Dipayan Chaudhuri, Dirk J. Slotboom, Donald R. Ronning, Donghan Lee, Dorian Dirksen, Douglas A. Shoue, Douglas William Zochodne, Durga Krishnamurthy, Dustin Duncan, Dylan M. Glubb, Edoardo Luigi Maria Gelardi, Edward C. Hsiao, Edward G. Lynn, Elany Barbosa Silva, Elena Aguilera, Elena Lenci, Elena Theres Abraham, Eleonora Lama, Eleonora Mameli, Elisa Leung, Ellie Giles, Emily M. Christensen, Emily R. Mason, Enrico Petretto, Ephraim F. Trakhtenberg, Eric J. Rubin, Erick Strauss, Erik W. Thompson, Erika Cione, Erika Mathes Lisabeth, Erkang Fan, Erna Geessien Kroon, Eunji Jo, Eva M. García-Cuesta, Evgenia Glukhov, Evripidis Gavathiotis, Fang Yu, Fei Xiang, Fenfei Leng, Feng Wang, Filippo Ingoglia, Focco van den Akker, Francesco Borriello, Franco J. Vizeacoumar, Frank Luh, Frederick S. Buckner, Frederick S. Vizeacoumar, Fredj Ben Bdira, Fredrik Svensson, G. Marcela Rodriguez, Gabriella Bognár, Gaia Lembo, Gang Zhang, Garrett Dempsey, Gary Eitzen, Gaétan Mayer, Geoffrey L. Greene, George A. Garcia, Gergely L. Lukacs, Gergely Prikler, Gian Carlo G. Parico, Gianni Colotti, Gilles De Keulenaer, Gino Cortopassi, Giovanni Roti, Giulia Girolimetti, Giuseppe Fiermonte, Giuseppe Gasparre, Giuseppe Leuzzi, Gopal Dahal, Gracjan Michlewski, Graeme L. Conn, Grant David Stuchbury, Gregory R. Bowman, Grzegorz Maria Popowicz, Guido Veit, Guilherme Eduardo de Souza, Gustav Akk, Guy Caljon, Guzmán Alvarez, Gwennan Rucinski, Gyeongeun Lee, Gökhan Cildir, Hai Li, Hairol E. Breton, Hamed Jafar-Nejad, Han Zhou, Hannah P. Moore, Hannah Tilford, Haynes Yuan, Heesung Shim, Heike Wulff, Heinrich Hoppe, Helena Chaytow, Heng-Keat Tam, Holly Van Remmen, Hongyang Xu, Hosana Maria Debonsi, Howard B. Lieberman, Hoyoung Jung, Hua-Ying Fan, Hui Feng, Hui Zhou, Hyeong Jun Kim, Iain R. Greig, Ileana Caliandro, Ileana Corvo, Imanol Arozarena, Imran N. Mungrue, Ingrid M. Verhamme, Insaf Ahmed Qureshi, Irina Lotsaris, Isin Cakir, J. Jefferson P. Perry, Jacek Kwiatkowski, Jacob Boorman, Jacob Ferreira, Jacob Fries, Jadel Müller Kratz, Jaden Miner, Jair L. Siqueira-Neto, James G. Granneman, James Ng, James Shorter, Jan Hendrik Voss, Jan M. Gebauer, Janelle Chuah, Jarrod J. Mousa, Jason T. Maynes, Jay D. Evans, Jeffrey Dickhout, Jeffrey P. MacKeigan, Jennifer N. Jossart, Jia Zhou, Jiabei Lin, Jiake Xu, Jianghai Wang, Jiaqi Zhu, Jiayu Liao, Jingyi Xu, Jinshi Zhao, Jiusheng Lin, Jiyoun Lee, Joana Reis, Joerg Stetefeld, John B. Bruning, John Burt Bruning, John G. Coles, John J. Tanner, John M. Pascal, Jonathan So, Jordan L. Pederick, Jose A. Costoya, Joseph B. Rayman, Joseph J. Maciag, Joshua Alexander Nasburg, Joshua J. Gruber, Joshua M. Finkelstein, Joshua Watkins, José Miguel Rodríguez-Frade, Juan Antonio Sanchez Arias, Juan José Lasarte, Julen Oyarzabal, Julian Milosavljevic, Julie Cools, Julien Lescar, Julijus Bogomolovas, Jun Wang, Jung-Min Kee, Jung-Min Kee, Junzhuo Liao, Jyothi C. Sistla, Jônatas Santos Abrahão, Kamakshi Sishtla, Karol R. Francisco, Kasper B. Hansen, Kathleen A. Molyneaux, Kathryn A. Cunningham, Katie R. Martin, Kavita Gadar, Kayode K. Ojo, Keith S. Wong, Kelly L. Wentworth, Kent Lai, Kevin A. Lobb, Kevin M. Hopkins, Keykavous Parang, Khaled Machaca, Kien Pham, Kim Ghilarducci, Kim S. Sugamori, Kirk James McManus, Kirsikka Musta, Kiterie M. E. Faller, Kiyo Nagamori, Konrad J. Mostert, Konstantin V. Korotkov, Koting Liu, Kristiana S. Smith, Kristopher Sarosiek, Kyle H. Rohde, Kyu Kwang Kim, Kyung Hyeon Lee, Lajos Pusztai, Lari Lehtiö, Larisa M. Haupt, Leah E. Cowen, Lee J. Byrne, Leila Su, Leon Wert-Lamas, Leonor Puchades-Carrasco, Lifeng Chen, Linda H. Malkas, Ling Zhuo, Lizbeth Hedstrom, Lizbeth Hedstrom, Loren D. Walensky, Lorenzo Antonelli, Luisa Iommarini, Luke Whitesell, Lía M. Randall, M. Dahmani Fathallah, Maira Harume Nagai, Mairi Louise Kilkenny, Manu Ben-Johny, Marc P. Lussier, Marc P. Windisch, Marco Lolicato, Marco Lucio Lolli, Margot Vleminckx, Maria Cristina Caroleo, Maria J. Macias, Marilia Valli, Marim M. Barghash, Mario Mellado, Mark A. Tye, Mark A. Wilson, Mark Hannink, Mark R. Ashton, Mark Vincent C.dela Cerna, Marta Giorgis, Martin K. Safo, Martin St. Maurice, Mary Ann McDowell, Marzia Pasquali, Masfique Mehedi, Mateus Sá Magalhães Serafim, Matthew B. Soellner, Matthew G. Alteen, Matthew M. Champion, Maxim Skorodinsky, Megan L. O’Mara, Mel Bedi, Menico Rizzi, Michael Levin, Michael Mowat, Michael R. Jackson, Mikell Paige, Minnatallah Al-Yozbaki, Miriam A. Giardini, Mirko M. Maksimainen, Monica De Luise, Muhammad Saddam Hussain, Myron Christodoulides, Natalia Stec, Natalia Zelinskaya, Natascha Van Pelt, Nathan M. Merrill, Nathanael Singh, Neeltje A. Kootstra, Neeraj Singh, Neha S. Gandhi, Nei-Li Chan, Nguyen Mai Trinh, Nicholas O. Schneider, Nick Matovic, Nicola Horstmann, Nicola Longo, Nikhil Bharambe, Nirvan Rouzbeh, Niusha Mahmoodi, Njabulo Joyfull Gumede, Noelle C. Anastasio, Noureddine Ben Khalaf, Obdulia Rabal, Olga Kandror, Olivier Escaffre, Olli Silvennoinen, Ozlem Tastan Bishop, Pablo Iglesias, Pablo Sobrado, Patrick Chuong, Patrick O’Connell, Pau Martin-Malpartida, Paul Mellor, Paul V. Fish, Paulo Otávio Lourenço Moreira, Pei Zhou, Pengda Liu, Pengda Liu, Pengpeng Wu, Percy Agogo-Mawuli, Peter L. Jones, Peter Ngoi, Peter Toogood, Philbert Ip, Philipp von Hundelshausen, Pil H. Lee, Rachael B. Rowswell-Turner, Rafael Balaña-Fouce, Rafael Eduardo Oliveira Rocha, Rafael V. C. Guido, Rafaela Salgado Ferreira, Rajendra K. Agrawal, Rajesh K. Harijan, Rajesh Ramachandran, Rajkumar Verma, Rakesh K. Singh, Rakesh Kumar Tiwari, Ralph Mazitschek, Rama K. Koppisetti, Remus T. Dame, Renée N. Douville, Richard C. Austin, Richard E. Taylor, Richard G. Moore, Richard H. Ebright, Richard M. Angell, Riqiang Yan, Rishabh Kejriwal, Robert A. Batey, Robert Blelloch, Robert J. Vandenberg, Robert J. Hickey, Robert J. Kelm, Robert J. Lake, Robert K. Bradley, Robert M. Blumenthal, Roberto Solano, Robin Matthias Gierse, Ronald E. Viola, Ronan R. McCarthy, Rosa Maria Reguera, Ruben Vazquez Uribe, Rubens Lima do Monte-Neto, Ruggiero Gorgoglione, Ryan T. Cullinane, Sachin Katyal, Sakib Hossain, Sameer Phadke, Samuel A. Shelburne, Sandra E. Geden, Sandra Johannsen, Sarah Wazir, Scott Legare, Scott M. Landfear, Senthil K. Radhakrishnan, Serena Ammendola, Sergei Dzhumaev, Seung-Yong Seo, Shan Li, Shan Zhou, Shaoyou Chu, Shefali Chauhan, Shinsaku Maruta, Shireen R. Ashkar, Show-Ling Shyng, Silvestro G. Conticello, Silvia Buroni, Silvia Garavaglia, Simon J. White, Siran Zhu, Sofiya Tsimbalyuk, Somaia Haque Chadni, Soo Young Byun, Soonju Park, Sophia Q. Xu, Sourav Banerjee, Stefan Zahler, Stefano Espinoza, Stefano Gustincich, Stefano Sainas, Stephanie L. Celano, Stephen J. Capuzzi, Stephen N. Waggoner, Steve Poirier, Steven H. Olson, Steven O. Marx, Steven R. Van Doren, Suryakala Sarilla, Susann M. Brady-Kalnay, Sydney Dallman, Syeda Maryam Azeem, Tadahisa Teramoto, Tamar Mehlman, Tarryn Swart, Tatjana Abaffy, Tatos Akopian, Teemu Haikarainen, Teresa Lozano Moreda, Tetsuro Ikegami, Thaiz Rodrigues Teixeira, Thilina D. Jayasinghe, Thomas H. Gillingwater, Thomas Kampourakis, Timothy I. Richardson, Timothy J. Herdendorf, Timothy J. Kotzé, Timothy R. O’Meara, Timothy W. Corson, Tobias Hermle, Tomisin Happy Ogunwa, Tong Lan, Tong Su, Toshihiro Banjo, Tracy A. O’Mara, Tristan Chou, Tsui-Fen Chou, Ulrich Baumann, Umesh R. Desai, Vaibhav P. Pai, Van Chi Thai, Vasudha Tandon, Versha Banerji, Victoria L. Robinson, Vignesh Gunasekharan, Vigneshwaran Namasivayam, Vincent F. M. Segers, Vincent Maranda, Vincenza Dolce, Vinícius Gonçalves Maltarollo, Viola Camilla Scoffone, Virgil A. Woods, Virginia Paola Ronchi, Vuong Van Hung Le, W. Brent Clayton, W. Todd Lowther, Walid A. Houry, Wei Li, Weiping Tang, Wenjun Zhang, Wesley C. Van Voorhis, William A. Donaldson, William C. Hahn, William G. Kerr, William H. Gerwick, William J. Bradshaw, Wuen Ee Foong, Xavier Blanchet, Xiaoyang Wu, Xin Lu, Xin Qi, Xin Xu, Xinfang Yu, Xingping Qin, Xingyou Wang, Xinrui Yuan, Xu Zhang, Yan Jessie Zhang, Yanmei Hu, Yasser Ali Aldhamen, Yicheng Chen, Yihe Li, Ying Sun, Yini Zhu, Yogesh K. Gupta, Yolanda Pérez-Pertejo, Yong Li, Young Tang, Yuan He, Yuk-Ching Tse-Dinh, Yulia A. Sidorova, Yun Yen, Yunlong Li, Zachary J. Frangos, Zara Chung, Zhengchen Su, Zhenghe Wang, Zhiguo Zhang, Zhongle Liu, Zintis Inde, Zoraima Artía, Abraham Heifets

**Affiliations:** 1 San Fransico, CA USA; 2 Atomwise Inc., San Fransico, USA; 3 Amgen, Thousand Oaks, USA; 4 OpenAI, San Francisco, USA; 5 Model Medicines, La Jolla, USA; 6 Atomic.AI, San Francisco, USA; 7 Edifice Health, Inc., San Mateo, USA; 8 METiS Therapeutics, Cambridge, USA; 9 Genentech, San Mateo, USA; 10 US Navy Medical Service Corps Officer (2300/1810D), San Mateo, USA; 11 Totus Medicines, Inc., Emeryville, USA; 12 Cytokinetics, Inc., South San Francisco, USA; 13 Nurix Therapeutics, San Francisco, USA; 14 Amazon Alexa, Suite, USA; 15 The University of North Carolina at Chapel Hill Eshelman School of Pharmacy, Chapel Hill, USA; 16 Refibered Inc., Cupertino, USA; 17 NVIDIA, Santa Clara, USA; 18 Harvard TH Chan School of Public Health, Boston, USA; 19 Columbia University, New York, USA; 20 University of São Paulo, São Paulo, Brazil; 21 Albert Einstein College of Medicine, Bronx, USA; 22 Nanyang Technological University, Singapore, Singapore; 23 University of Washington, Seattle, USA; 24 Chan Medical School, University of Massachusetts, Worcester, USA; 25 Queensland University of Technology, Brisbane, Australia; 26 University of Michigan Medical School, Ann Arbor, USA; 27 Columbia University Irving Medical Center, New York, USA; 28 Universidade Federal de Santa Catarina, Florianópolis, Brazil; 29 Université de Montréal, Montreal, Canada; 30 Instituto René Rachou-Fundação Oswaldo Cruz/Fiocruz Minas, Belo Horizonte, Brazil; 31 Herbert Wertheim College of Medicine, Biomolecular Science Institute, Florida International University, Miami, USA; 32 NYU Langone Health, New York, USA; 33 The University of Texas Medical Branch at Galveston, Galveston, USA; 34 University of Houston, Galveston, USA; 35 University of California, San Diego, USA; 36 School of Medicine, Tufts University, Medford, USA; 37 University of California, Davis, Davis, USA; 38 University of Western Australia, Crawley, Australia; 39 University of Groningen, Groningen, The Netherlands; 40 Wayne State University, Detroit, USA; 41 University of Zurich, Zürich, Switzerland; 42 Stanford University, Stanford, USA; 43 University of Antwerp, Antwerp, Belgium; 44 University of Rome Tor Vergata, Rome, Italy; 45 University of Edinburgh, Edinburgh, UK; 46 Department of Plant Molecular Genetics, Centro Nacional de Biotecnología, Consejo Superior de Investigaciones Científicas (CNB-CSIC), Madrid, Spain; 47 CNR (Italian National Research Council), Rome, Italy; 48 University of Pavia, Pavia, Italy; 49 University of Vermont, Burlington, USA; 50 University of Florence, Florence, Italy; 51 University of California, Berkeley, Berkeley, USA; 52 Cincinnati Children’s Hospital Medical Center, Cincinnati, USA; 53 University of Saskatchewan, Saskatoon, Canada; 54 Broad Institute of MIT and Harvard, Cambridge, USA; 55 Boston University, Boston, USA; 56 CancerCare Manitoba Research Institute, Winnipeg, Canada; 57 University of Michigan, Ann Arbor, USA; 58 Wadsworth Center, New York State Department of Health and University at Albany, Albany, USA; 59 Dana-Farber Cancer Institute, Boston, USA; 60 University of Parma, Parma, Italy; 61 Helmholtz Institute for Pharmaceutical Research Saarland, Saarbrücken, Germany; 62 University of Bologna, Bologna, Italy; 63 Sapienza University of Rome, Rome, Italy; 64 Tampere University, Tampere, Finland; 65 University of Connecticut, Storrs, USA; 66 Centro de Investigación Médica Aplicada, Universidad de Navarra, Pamplona, Spain; 67 Tulane National Primate Research Center, Tulane University, Covington, USA; 68 Scripps Research, San Diego, USA; 69 Louisiana State University School of Medicine, New Orleans, USA; 70 Duke-NUS Medical School, Singapore, Singapore; 71 Case Western Reserve University, Cleveland, USA; 72 Oregon Health and Science University and Tanta University in Tanta, Tanta, Egypt; 73 University College London, London, UK; 74 Saint Louis University, St. Louis, USA; 75 Rhodes University, Makhanda, South Africa; 76 Universidade Federal de Minas Gerais (UFMG), Belo Horizonte, Brazil; 77 University of Manitoba, Winnipeg, Canada; 78 National Jewish Health, Denver, USA; 79 Drugs for Neglected Diseases Initiative (DNDi), Geneva, Switzerland; 80 The University of Adelaide, Adelaide, Australia; 81 Mcule, Budapest, Hungary; 82 University of Utah, Salt Lake City, USA; 83 The University of Texas at Austin, Austin, USA; 84 Kansas State University, Manhattan, USA; 85 UniQuest Pty Ltd, St Lucia, Australia; 86 University of Chicago, Chicago, USA; 87 Harvard University, Cambridge, USA; 88 University of Freiburg, Freiburg Im Breisgau, Germany; 89 Dana-Farber Cancer Institute and Harvard Medical School, Boston, USA; 90 IRB Barcelona, Barcelona, Spain; 91 Istituto Italiano Di Tecnologia, Genoa, Italy; 92 QIMR Berghofer Medical Research Institute, Herston, Australia; 93 University of California, Santa Cruz, Santa Cruz, USA; 94 University of Kentucky, Lexington, USA; 95 University of Tennessee Health Science Center, Memphis, USA; 96 Children’s Cancer Therapy Development Institute, Beaverton, USA; 97 Columbia University Medical Center, New York, USA; 98 Yale School of Medicine, New Haven, USA; 99 University of Bonn, Bonn, Germany; 100 Ludwig-Maximilians-Universität München, Munich, Germany; 101 Purdue University, West Lafayette, USA; 102 Canterbury Christ Church University, Canterbury, UK; 103 National Centre for Biotechnology (CNB-CSIC), Madrid, Spain; 104 University of South Australia and SA Pathology, Adelaide, Australia; 105 CUNY Advanced Science Research Center, New York, USA; 106 The University of Toledo, Toledo, USA; 107 University of North Carolina at Chapel Hill, Chapel Hill, USA; 108 Boston Children’s Hospital and Harvard Medical School, Boston, USA; 109 West Virginia University, Morgantown, USA; 110 Universidade de Santiago de Compostela, Santiago, Spain; 111 University of North Texas Health Science Center at Fort Worth, Fort Worth, USA; 112 Institut Pasteur Korea, Seongnam, South Korea; 113 Carle Illinois College of Medicine, Urbana, USA; 114 Università del Piemonte Orientale, Vercelli, Italy; 115 Saskatchewan Cancer Agency, Saskatoon, Canada; 116 Curtin University, Bentley, Australia; 117 Oregon Health and Science University, Portland, USA; 118 Virginia Commonwealth University, Richmond, USA; 119 Tufts University, Medford, USA; 120 University of Nebraska Medical Center, Omaha, USA; 121 University of Louisville, Louisville, USA; 122 Dana Farber Cancer Institute, Boston, USA; 123 University of Notre Dame, Notre Dame, USA; 124 University of Alberta, Edmonton, Canada; 125 University of Toronto, Toronto, Canada; 126 University of Piemonte Orientale, Vercelli, Italy; 127 University of California, San Francisco, San Francisco, USA; 128 St. Joseph’s Healthcare Hamilton, and Hamilton Center for Kidney Research, McMaster University, Hamilton, Canada; 129 Skaggs School of Pharmacy and Pharmaceutical Sciences, University of California San Diego, San Diego, USA; 130 Universidad de La República, Montevideo, Uruguay; 131 University of Cologne, Cologne, Germany; 132 Johnson University, Knoxville, USA; 133 Indiana University, Bloomington, USA; 134 School of Medicine, University of Connecticut, Farmington, USA; 135 Stellenbosch University, Stellenbosch, South Africa; 136 University of Calabria, Arcavacata, Italy; 137 Michigan State University, East Lansing, USA; 138 University of Washington, Washington, USA; 139 Weill Cornell Medicine-Qatar, Ar-Rayyan, Qatar; 140 Gachon University, Seongnam, South Korea; 141 Florida International University, Miami, USA; 142 California Institute of Technology, Pasadena, USA; 143 Boston Children’s Hospital, Boston, USA; 144 Saskatchewan Cancer Agency and University of Saskatchewan, Saskatchewan, Canada; 145 Sino-American Cancer Foundation, Covina, USA; 146 Leiden University, Leiden, The Netherlands; 147 Rutgers University, Newark, USA; 148 Core Research Laboratory, ISPRO, Florence, Italy; 149 Caltech, Pasadena, USA; 150 University of Alberta, Edmonton, USA; 151 Montreal Heart Institute and Université de Montréal, Montreal, Canada; 152 McGill University, Montreal, Canada; 153 Antwerp University, Antwerp, Belgium; 154 University of Bari Aldo Moro, Bari, Italy; 155 Alma Mater Studiorum-University of Bologna, Bologna, Italy; 156 University of Toledo, Toledo, USA; 157 International Institute of Molecular and Cell Biology in Warsaw, Warsaw, Poland; 158 Infection Medicine, University of Edinburgh The Chancellor’s Building, Edinburgh, UK; 159 Emory University, Atlanta, USA; 160 University of Pennsylvania, Philadelphia, USA; 161 Helmholtz Zentrum München, Munich, Germany; 162 Washington University School of Medicine, St. Louis, USA; 163 CENUR Litoral Norte, Universidad de La República, Montevideo, Uruguay; 164 University of Southampton, Southampton, UK; 165 Centre for Cancer Biology, University of South Australia, Adelaide, Australia; 166 University of Missouri, Columbia, USA; 167 Baylor College of Medicine, Houston, USA; 168 Yale University, New Haven, USA; 169 Reno School of Medicine, University of Nevada, Reno, USA; 170 University of Manitoba and CancerCare Manitoba, Winnipeg, Canada; 171 Goethe University Frankfurt, Frankfurt, Germany; 172 Oklahoma Medical Research Foundation/Oklahoma City VA Medical Center, Oklahoma City, USA; 173 Oklahoma Medical Research Foundation, Oklahoma City, USA; 174 Department of Biomolecular Sciences, School of Pharmaceutical Sciences of Ribeirão Preto, University of São Paulo, Ribeirão Preto, SP Brazil; 175 Ulsan National Institute of Science and Technology, Ulsan, South Korea; 176 University of New Mexico Comprehensive Cancer Center, Albuquerque, USA; 177 Ulsan National Institute of Science and Technology (UNIST), Ulsan, South Korea; 178 University of Aberdeen, Aberdeen, UK; 179 University of Turin, Turin, Italy; 180 Universidad de La República, CenUR LN, Montevideo, Uruguay; 181 Navarrabiomed-IdiSNA, Pamplona, Spain; 182 Independent, Los Angeles, USA; 183 Vanderbilt University Medical Center, Nashville, USA; 184 University of Hyderabad, Hyderabad, India; 185 University of Sydney, Sydney, Australia; 186 City of Hope Medical Center, Duarte, USA; 187 Weill Cornell Medicine, New York, NY 10065 USA; 188 University of Toledo College of Medicine and Life Sciences, Toledo, USA; 189 School of Medicine, Wayne State University, Detroit, USA; 190 University of Georgia, Athens, USA; 191 The Hospital for Sick Children, Toronto, Canada; 192 United States Department of Agriculture, Agricultural Research Service (USDA-ARS), Washington, DC USA; 193 McMaster University, Hamilton, Canada; 194 University of California, Riverside, Riverside, USA; 195 The University of Western Australia, Perth, Australia; 196 The University of Connecticut, Storrs, USA; 197 Duke University School of Medicine, Durham, USA; 198 University of Nebraska-Lincoln, Lincoln, USA; 199 Sungshin University, Seoul, South Korea; 200 University of Adelaide, Adelaide, Australia; 201 University Toronto, Toronto, Canada; 202 University of Texas Southwestern Medical Center, Dallas, USA; 203 Centro Nacional de Biotecnologia/CSIC, Madrid, Spain; 204 Centro de Investigación Médica Aplicada, Pamplona, Spain; 205 Centro de Investigación Médica Aplicada, Universidad de Navarra, Pamplona, Spain; 206 University of Wisconsin-Madison, Madison, USA; 207 Indiana University School of Medicine, Indianapolis, USA; 208 University of Montana, Missoula, USA; 209 Brunel University London, London, UK; 210 Chapman University, Orange, USA; 211 Weill Cornell Medicine Qatar, Ar-Rayyan, Qatar; 212 Université du Québec À Montréal, Montreal, Canada; 213 National Taiwan University, Taipei, Taiwan; 214 Rhodes College, Memphis, USA; 215 Harvard School of Public Health, Boston, USA; 216 University of Central Florida, Orlando, USA; 217 University of Rochester, Rochester, USA; 218 George Mason University, Fairfax, USA; 219 University of Oulu, Oulu, Finland; 220 Instituto Investigación Sanitaria La Fe, Valencia, Spain; 221 Ludwig-Maximilians-University, Munich, Germany; 222 Brandeis University, Waltham, USA; 223 Universidad de La República, CENUR Litoral Norte, Montevideo, Uruguay; 224 Arabian Gulf University, Manama, Bahrain; 225 University of Cambridge, Cambridge, UK; 226 University of Magna Graecia, Catanzaro, Italy; 227 Massachusetts General Hospital, Boston, USA; 228 University of Missouri-Columbia, Columbia, USA; 229 Marquette University, Milwaukee, USA; 230 University of North Dakota, Grand Forks, USA; 231 Simon Fraser University, Burnaby, Canada; 232 CancerCare Manitoba Research Institute (CCMR), Winnipeg, Canada; 233 The University of Queensland, Brisbane, Australia; 234 University of Manitoba and CancerCare Manitoba Research Institute, Winnipeg, Canada; 235 Sanford Burnham Prebys, La Jolla, USA; 236 University of Amsterdam, Amsterdam, The Netherlands; 237 UConn Health, Farmington, USA; 238 The University of Texas MD Anderson Cancer Center, Houston, USA; 239 Walter Sisulu University, Mthatha, South Africa; 240 Virginia Tech, Blacksburg, USA; 241 University of Houston, Houston, USA; 242 Rutgers University, New Brunswick, USA; 243 University of Nevada, Reno, USA; 244 Universidad de León, León, Spain; 245 School of Medicine, Case Western Reserve University, Cleveland, USA; 246 School of Medicine, UConn Health, Farmington, USA; 247 University of Rochester Medical Center, Rochester, USA; 248 Chapman University School of Pharmacy, Irvine, USA; 249 University of Winnipeg/St. Boniface Research Centre, Winnipeg, Canada; 250 Fred Hutchinson Cancer Center, Seattle, USA; 251 Helmholtz Institute for Pharmaceutical Research Saarland (HIPS), Saarbrücken, Germany; 252 Technical University of Denmark, Kongens Lyngby, Denmark; 253 The City College of New York, New York, USA; 254 Children’s Cancer, Therapy Development Institute (Cc-TDI), Beaverton, USA; 255 Soka University, Hachioji, Japan; 256 Institute of Clinical Physiology, National Research Council, Pisa, Italy; 257 Charles Sturt University, Bathurst, Australia; 258 Washington University, St Louis, USA; 259 University of Dundee, Dundee, UK; 260 Montreal Heart Institute, Montreal, Canada; 261 Columbia University Vagelos College of Physicians and Surgeons, Columbia, USA; 262 Georgetown University, Washington, USA; 263 Duke University, Durham, USA; 264 Center for Applied Medical Research, University of Navarra, Pamplona, Spain; 265 King’s College London, London, UK; 266 Precision Vaccines Program, Division of Infectious Diseases, Boston Children’s Hospital, Boston, USA; 267 Fred Hutchinson Cancer Research Center, Seattle, USA; 268 Louisiana State University, Baton Rouge, USA; 269 Massey University, Palmerston North, New Zealand; 270 Wake Forest University School of Medicine, Winston-Salem, USA; 271 Central South University, Changsha, China; 272 SUNY Upstate Medical University, Syracuse, USA; 273 University of Oxford, Oxford, UK; 274 Goethe-University, Frankfurt, Frankfurt, Germany; 275 Institute for Cardiovascular Prevention (IPEK), Ludwig-Maximilians-Universität München, Munich, Germany; 276 Harvard T.H. Chan School of Public Health, Boston, USA; 277 School of Medicine, Boston University, Boston, USA; 278 University of Texas Health Science Center at San Antonio, San Antonio, USA; 279 University of Helsinki, Helsinki, Finland; 280 Wadsworth Center, NYSDOH, Albany, USA; 281 The University of Sydney, Sydney, Australia

**Keywords:** Drug discovery, High-throughput screening, Virtual screening, Machine learning

## Abstract

High throughput screening (HTS) is routinely used to identify bioactive small molecules. This requires physical compounds, which limits coverage of accessible chemical space. Computational approaches combined with vast on-demand chemical libraries can access far greater chemical space, provided that the predictive accuracy is sufficient to identify useful molecules. Through the largest and most diverse virtual HTS campaign reported to date, comprising 318 individual projects, we demonstrate that our AtomNet® convolutional neural network successfully finds novel hits across every major therapeutic area and protein class. We address historical limitations of computational screening by demonstrating success for target proteins without known binders, high-quality X-ray crystal structures, or manual cherry-picking of compounds. We show that the molecules selected by the AtomNet® model are novel drug-like scaffolds rather than minor modifications to known bioactive compounds. Our empirical results suggest that computational methods can substantially replace HTS as the first step of small-molecule drug discovery.

## Introduction

Despite present interest in AI/ML and thirty years of case studies^1–4^, computational screening techniques have achieved limited adoption within the pharmaceutical industry. A recent investigation into the origins of 156 clinical candidates^5^ found that only 1% came from virtual screening; in contrast, over 90% of clinical candidates were derived from patent busting or high throughput screening (HTS). Unfortunately, these sources are increasingly challenged, given the pharmaceutical industry’s shift to novel target classes, such as proximity-induced protein degradation^6^, protein–protein interactions^7^, and RNA targeting^8^.

Currently, HTS is the critical tool in drug discovery, providing most novel scaffolds of recent clinical candidates^5,9,10^. These initial starting points crucially shape the course of downstream medicinal chemistry efforts, as most drugs preserve at least 80% of the scaffold of the initially identified lead^11^. Despite these foundational contributions, HTS suffers from practical limitations. Principally, HTS, like all physical experiments, requires that the compounds exist. However, with the advent of synthesis-on-demand libraries, most commercially-available molecules have yet to be synthesized. Still, they can be made and delivered for testing in a matter of weeks^12–14^. These libraries comprise trillions of molecules^14,15^ that exemplify millions of otherwise-unavailable scaffolds^12^, providing an opportunity to substantially expand the scope and diversity of available chemical space explored in the standard drug discovery process.

Computational approaches unlock this opportunity by reversing the requirement to make molecules before testing them. When computational experiments replace HTS as the primary screen, molecules are tested *before* they are made, and the results from these experiments can inform which molecules are worth synthesizing. Computational experiments further promise to improve upon HTS in terms of cost, speed, need to produce significant quantities of protein^16^, effort of miniaturizing assay formats while maintaining experimental integrity^17–19^, and reducing false-positive and false-negative rates^16,20–23^ including artifacts from aggregation, covalent modification of the target, autofluorescence, or interactions with the reporter rather than the target^20,24,25^. Historical computational techniques such as ligand-based QSAR^26–28^, structure-based docking^29,30^, and machine learning^31,32^ purport to address these limitations of physical screening methods. Unfortunately, these techniques have not replaced HTS; in fact, despite increasing interest in ML, the proportion of drugs discovered with computational techniques has remained steady over the past decades^5,10^.

**Figure 1:**
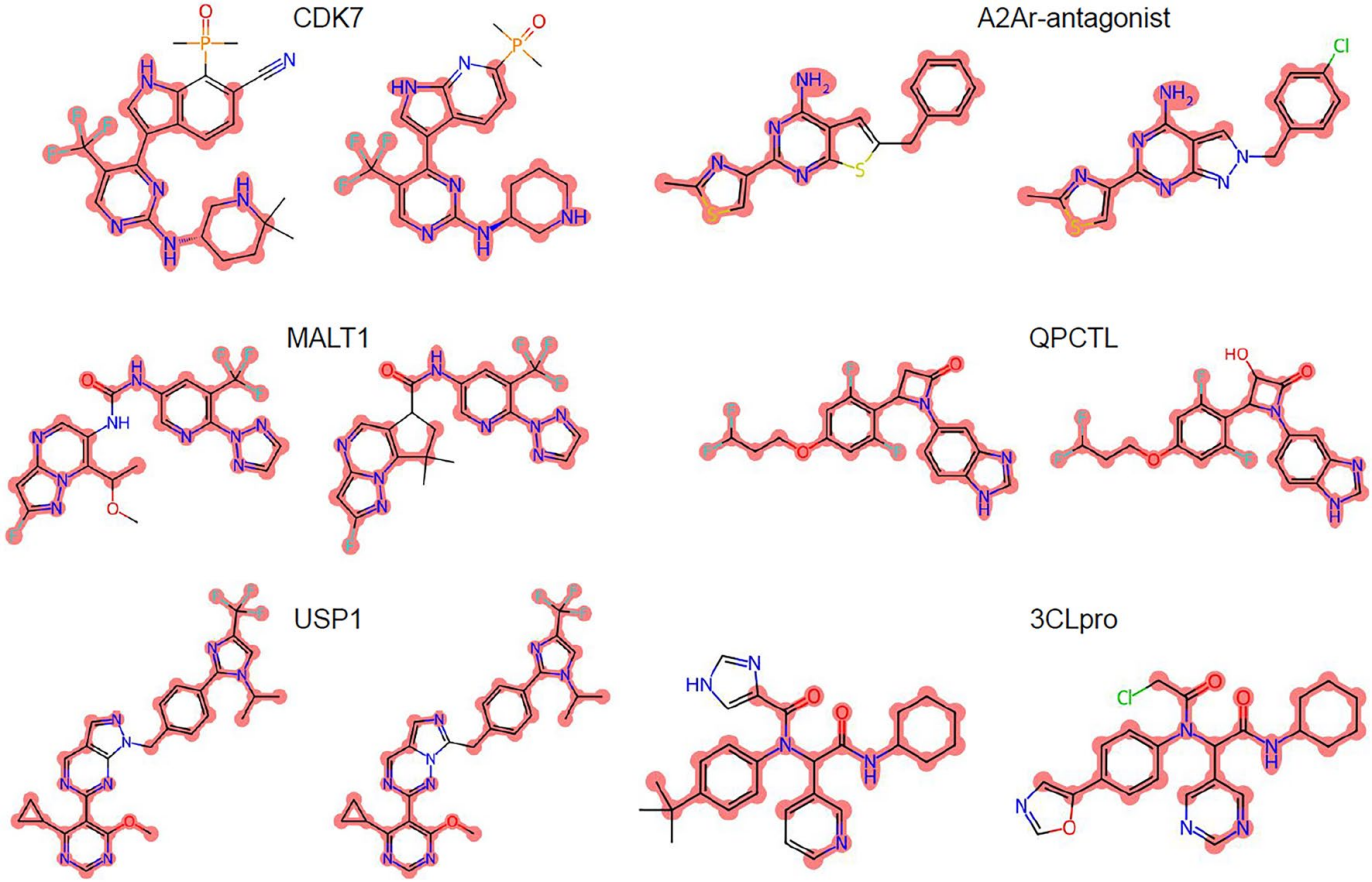
Pairs of representative compounds extracted from AI patents (right) and corresponding prior patents (left) for clinical-stage programs (CDK7^92,93^, A2Ar-antagonist^94,95^, MALT1^96,97^, QPCTL^98,99^, USP1^100,101^, and 3CLpro^102,103^). The identical atoms between the chemical structures are highlighted in red.

Because there will always be individual targets for which one screening technique can identify more hits than another, the key question governing if computation is ready to be the default hit discovery technique is whether computational screens can identify hits successfully across a broad range of diverse targets. Unfortunately, despite excellent benchmark accuracies^33–35^, prospective discovery accuracy remains modest^33,36,37^. For example, Cerón-Carrasco^38^ reported over 700 virtual screens against the SARS-CoV-2 main protease. However, when the author sought to validate the computational predictions via physical experiments, the identified compounds were barely active (800uM). Computational approaches have also been limited by a need for extensive target-specific training data^31,39–41^, a requirement for high-quality X-ray crystal structures^42,43^, dependence on human adjudication (so-called ‘cherry-picking’)^12^, or a limited domain of applicability^44–48^. Even recent systems have demonstrated utility only in identifying minor variants of known molecules for well-studied proteins with tens of thousands of known binders in their training data^49,50^. Figure 1 exemplifies the striking similarities between recently ML-developed compounds and their preceding published chemical matter. This is particularly concerning, as a myopic focus on well-studied proteins has been identified as a cause of low productivity in pharmaceutical discovery^51^.

Nevertheless, we have observed that deep learning approaches are not as limited as these historical examples would imply. Using our AtomNet^52–54^ screening system, we have previously reported success in finding novel scaffolds for targets without known ligands^55–57^, X-ray crystal structures^56–60^, or both^56,57^, as well as challenging modulation via protein–protein interaction^59,61^ or allosteric binding^60^ (see Supplementary Table S1 for examples). However, individual examples do not demonstrate the overall success of such deep learning systems. We therefore report our internal discovery efforts against 22 targets of pharmaceutical interest. We then attempted to further assess the generalizability and robustness of deep learning predictive systems by identifying bioactive molecules for a diverse set of targets. We partnered with 482 academic labs and screening centers, from 257 different academic institutions across 30 countries, through our academic collaboration program, the Artificial Intelligence Molecular Screen (AIMS). This collaboration afforded an opportunity to prospectively evaluate the utility of the AtomNet model as a primary screen across a broad range of diverse, challenging, and realistic targets. In aggregate, we report successes and failures from 318 prospective experiments and evaluate our AtomNet machine-learning technology’s ability to serve as a viable alternative to physical HTS campaigns.

## Results

We investigated the ability of deep learning-based methods to identify novel bioactive chemotypes by applying the AtomNet model to identify hits for 22 internal targets of pharmaceutical interest. We also explored the breadth of applicability of this approach by attempting to identify drug-like hits in single-dose screens for 296 academic targets, of which 49 were followed up with dose–response experiments, and 21 were further validated by exploring analogs of the initial hits. The average hit rate for our internal projects (6.7%) was comparable to the hit rate for our academic collaborations (7.6%).

### Internal portfolio validation

As part of Atomwise’s internal drug discovery efforts, we used the AtomNet model instead of high-throughput or DNA-encoded library (DEL) screening. We screened a 16-billion synthesis-on-demand chemical space^62^, which is several thousand times larger than HTS libraries and even exceeds the size of most DELs without suffering limitations of DNA-compatible chemistry^16,23^. Each screen requires over 40,000 CPUs, 3,500 GPUs, 150 TB of main memory, and 55 TB of data transfers. We describe the protocol in detail in the Methods section; briefly, we computationally scored each catalog compound after removing molecules that were prone to interfere with the assays or were too similar to known binders of the target or its homologs. The neural network analyzes and scores the 3D coordinates of each generated protein–ligand co-complex, producing a list of ligands ranked by their predicted binding probability. Our workflow then clusters the top-ranked molecules to ensure diversity and algorithmically selects the highest-scoring exemplars from each cluster. At no point are compounds manually cherry-picked. The molecules were synthesized at Enamine (https://enamine.net) and quality controlled by LC–MS to purity > 90%, in agreement with HTS standards^63^. Hits were further validated using NMR. We then physically tested, on average, 440 compounds per target at reputable contract research organizations (CROs), while attempting to mitigate assay interferences such as aggregation and oxidation with standard additives (*e.g.*, Tween-20, Triton-X 100, and dithiothreitol (DTT)). We describe the assay protocols in detail in the Supplementary Data S1.

We describe the results of the 22 experiments in Table 1. In 91% of the experiments, we identified single-dose (SD) hits that were reconfirmed in dose–response (DR) experiments. The average target DR hit rate was 6.7% compared to 8.8% from the SD screens. Only 16 of the 22 projects were structurally enabled with X-ray crystallography; one used a cryo-EM structure, while five used homology models with an average sequence identity of 42% to their template protein. The DR hit rate for the cryo-EM project was 10.56%, while the average hit rate for the homology models was a similar 10.8%.

**Table 1:** Results from 22 Atomwise internal programs.

Gene name	# of compounds tested	SD hit rate (%)	DR hit rate (%)	Potency range (IC50/Ki, uM)	# of analog tested	SD analog hit rate (%)	DR analog hit rate (%)	Analog potency range (IC50/Ki, uM)
ASAH1	376	10.64	7.71	0.3–102	–	–	–	–
AXL	597	12.06	8.21	0.181–71	3200	35.59	33.56	0.079–86
BCL2	422	3.08	0.00	–	–	–	–	–
CBLB	422	1.66	0.00	–	–	–	–	–
CDK5	786	10.69	10.43	0.049–79	587	47.53	43.61	0.43–76
CDK7	786	10.69	10.56	0.099–60	735	28.44	27.35	0.191–10
GFPT1	384	6.51	2.34	31–86	734	24.93	24.11	1–194
KCNT1	416	9.62	7.69	1.1–30	–	–	–	–
KDM6A	356	3.93	1.12	24–58	–	–	–	–
LATS1	418	18.18	17.94	0.077–82	841	51.72	45.78	0.034–98
MC2R	208	11.54	9.62	16–68	419	39.38	38.42	2.4–97
MDM4	422	2.37	0.47	5.9–29.8	192	18.23	18.23	4.4–90
NT5E	335	1.49	0.30	176	221	9.95	1.81	8.3–65
PARG	334	7.78	7.78	15–250	–	–	–	–
PARP14	576	5.38	2.95	3–96	616	26.46	26.30	0.2–95
POLQ	330	11.82	11.52	1.2–49	559	11.27	8.77	1.5–42
PPARA	422	4.03	0.24	131	211	14.22	3.79	59–95
PPM1D	530	11.89	6.98	4.5–98	–	–	–	–
PRMT5	422	4.03	0.95	7.2–79	415	7.95	5.54	19–114
PRODH2	542	2.77	1.11	15–84	–	–	–	–
TYK2	189	38.10	34.39	0.016–9	457	71.33	60.39	0.006–10
VCP	416	4.81	4.81	2.4–64	738	–	–	–

SD and DR denote single-dose and dose–response, respectively.

We then advanced 14 projects with at least one dose-responsive scaffold to a round of analog expansion. We found new bioactive analogs in the SD screen for all projects, with an average hit rate of 29.8%. Further validation with DR resulted in an average hit rate of 26% per project, which compares favorably with typical HTS hit rates ranging from 0.151 to 0.001%^64,65^. We note that the size and chemical diversity within and between physical^66^ and virtual^14^ HTS libraries prevent an explicit evaluation of the methods over the same chemical space. The most potent analogs ranged from single-digit nanomolar, against a kinase, to double-digit micromolar, against a transcription factor (Supplementary Table S2). Additionally, we present two internal studies in detail. For Large Tumor Suppressor Kinase 1 (LATS1), we identified potent compounds despite the lack of a crystal structure or known active compounds. For ATP-driven chaperone Valosin Containing Protein (VCP) we identified novel allosteric and orthosteric modulators.

### Academic validation

In addition to our internal discovery efforts, we performed virtual screens for 296 targets, comprising more than 20 billion individual neural network scores of generated protein–ligand co-complexes. We purchased, on average, 85 off-the-shelf commercially available compounds, quality controlled by NMR and LC–MS to > 90% purity^63^, and plated in a single 96-well plate. The compounds were then physically screened for activity against the target of interest in single-dose assays (see Supplemental Data S1 for assay protocols). As with HTS primary screens, additional characterization studies are required to validate the initially identified hits so, in 49 projects, we performed dose–response studies and analog expansion. We present a summary of our results in Supplementary Table S3.

**Figure 2:**
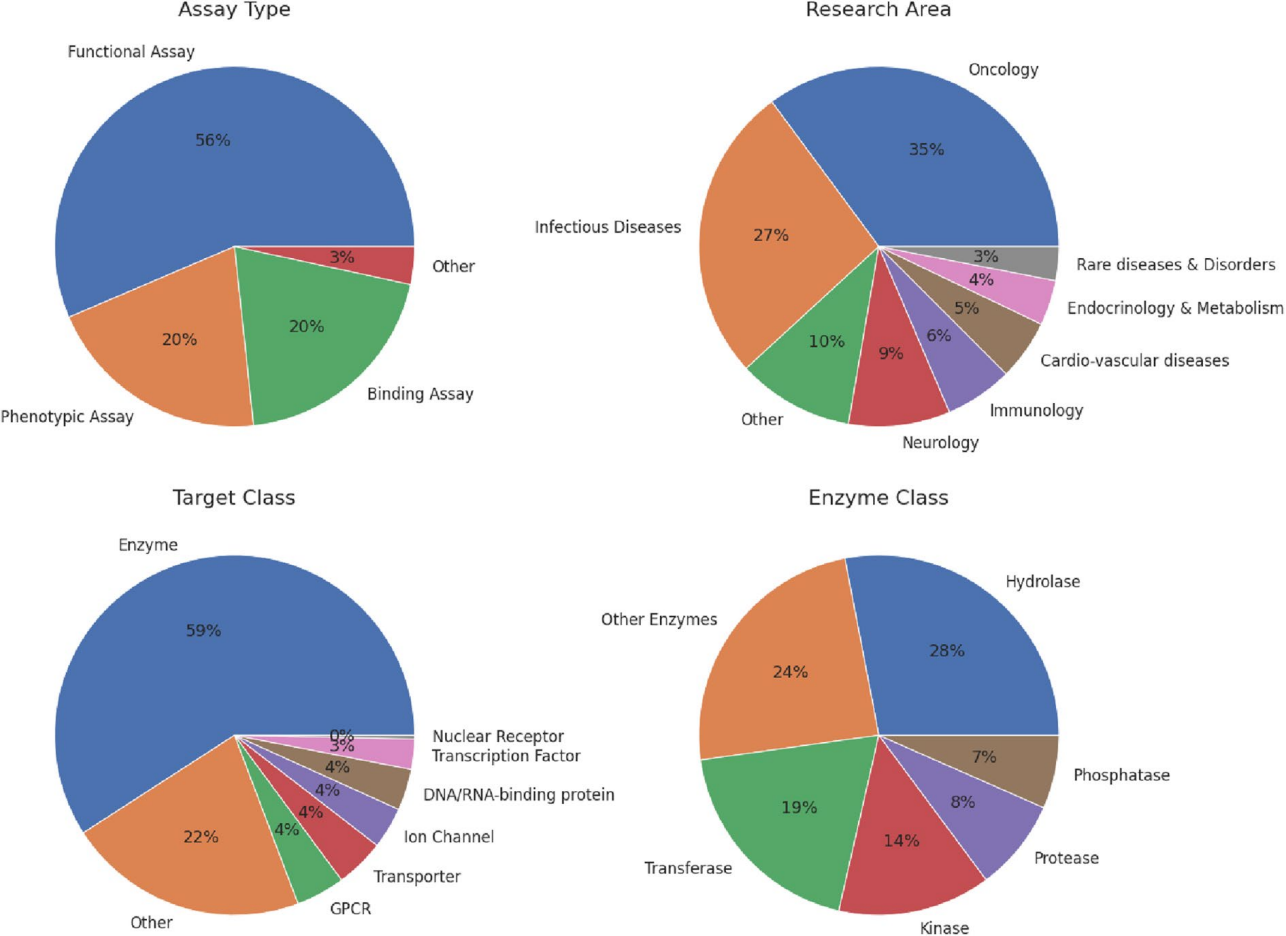
The distributions of 296 AIMS projects across assay types used in the primary screen, research areas, target classes, and further breakdown to enzyme classes when applicable.

Figure 2 illustrates the distributions of projects across therapeutic areas, protein families, and assay types. Every major therapeutic area is represented, with the most frequent area being oncology, comprising 35% of projects, followed by infectious diseases and neurology, comprising 27% and 9% of projects, respectively. Breaking down the projects by protein families reveals that all major enzyme classes are represented, with enzymes comprising 59% of the targets and membrane proteins such as GPCR, transporters, and ion channels, representing 12% of the targets. Working on a large and diverse set of therapeutic targets requires a heterogeneous collection of biological assays; 20% of the assays measured direct binding, whereas 56% and 20% were functional and phenotypic.

In 215 projects, we identified at least one bioactive compound for the target in a biochemical or cell-based assay. This 73% success rate substantially improves over the ∼50% success rate for HTS^21,67^. On average, we screened 85 compounds per project and discovered 4.6 active hits, with an average hit rate of 5.5%. For the subset of targets where we found any hits, the average was 6.4 hits per project. Thus, we achieved an average hit rate of 7.6%, which again compares favorably with typical HTS hit rates. See Supplementary Material S1 for all assay definitions and conditions. Supplementary Table S4 shows a representative bioactive compound from each of the 215 successful projects, and Supplementary Fig. S2 shows that the physicochemical properties of the identified hits are largely druglike and Lipinski-compliant.

**Figure 3:**
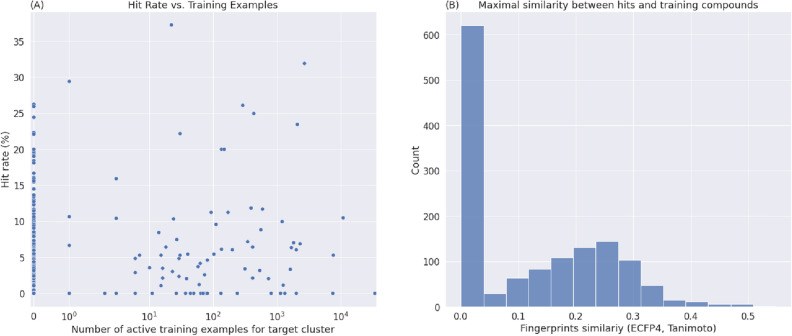
(**A**) An illustration of the hit rate versus the number of training examples available to our model. Each point represents a project, with the x-axis denoting the number of active molecules in our training for the target protein or homologs and the y-axis denoting the hit rate of the project (the percentage of molecules tested in the project that were active). The model shows no dependence on the availability of on-target training examples. For 70% of the targets, the AtomNet model training data lacked any active molecules for that target or any similar targets with greater than 70% sequence identity, yet the model achieved a hit rate of 5.3% compared to 6.1% when on-target data was available. (**B**) The distribution of similarities between hits and their most-similar bioactive compounds in our training data. Our screening protocol ensures that the compounds subjected to physical testing are not similar to known active compounds or close homologs (< 0.5 Tanimoto similarity using ECFP4, 1024 bits). Because 70% of the AIMS targets had no annotated bioactivities in our training dataset, hits identified in these projects have a similarity value of zero.

The AtomNet technology robustly identified active molecules, even for targets that lacked prior on-target bioactivity data. This ability to identify hits for previously undrugged targets is critical if machine learning-based approaches are to replace HTS as the default primary screening approach. For 207 out of the 296 targets (70%), the training data available for AtomNet models lacked a single active molecule for that target or any closely related protein (i.e., proteins with sequence identity greater than 70%). We interpret this as evidence of the ability of properly-architected machine learning systems to extrapolate to novel biological space. Figure 3A illustrates the hit rate versus the number of training examples available to our model. Although previous computational approaches typically require thousands of on-target training examples^31,39,42^, the lack of correlation between training examples and hit rate (R^2^ = 0.0021, p-value = 0.43) shows that our ML algorithm is agnostic to the availability of such data. We achieved an average success rate of 75% and hit rates of 5.3% when no training data was available, comparable to the 67% and 6.1% success and hit rates achieved when binding data was available in the training set. Interestingly, we also do not see a significant increase in hit rate attributable to the proportion of binding data available for a target (R^2^ = 0.008, p-value = 0.39). This reflects the robustness of the screening protocol and the chemical dissimilarity of scaffolds identified by AtomNet models to previously known bioactive compounds.

Next, we assessed the ability of the AtomNet models to identify novel scaffolds. This is a critical capability for primary screens, as follow-up assays tend to work within the chemical space uncovered in the initial screen. The task of novel scaffold identification appears in two distinct scenarios: (1) when no scaffold is known for the target and we wish to identify the first scaffold, and (2) when some scaffolds are known but we wish to identify dissimilar scaffolds because novel chemical matter can yield improved selectivity, toxicity, pharmacokinetics, or patentability. Performance of AtomNet models for the first scenario, when no scaffolds for the target existed in the AtomNet model training data, was evaluated on 70% of the targets, where the training data contained no active molecules for the target or its homologs (vide supra). We achieved an average hit rate of 5.3% for targets with no training data. For the second scenario, we analyzed the similarity of the identified hits to known bioactive compounds in our training data (Fig. 3B). Our screening protocol ensures that the compounds subjected to physical testing are not similar to known active compounds or close homologs (< 0.5 Tanimoto similarity using ECFP4^68^, 1024 bits). We interpret this as evidence of the ability of properly-architected machine learning systems to extrapolate to novel chemical space as well. For cases where training data was available (i.e., the Tanimoto similarity is above zero), the similarity distribution is close to the one expected by random compound pairs^69^. The novelty of the small-molecule structures is striking because target-specific machine-learning algorithms tend to uncover highly similar analogs for known bioactive molecules^50,70,71^. The superior performance of the AtomNet model is expected, considering the bias-variance tradeoff^72^ in machine learning algorithms. Because the AtomNet convolutional neural network is a global model, concurrently trained on millions of bioactivities, hundreds of thousands of small molecules, and thousands of protein binding sites, it can reduce both bias and variance of the model compared to target-specific ones^33^. Specifically, our global model can benefit from multiple levels of information captured in the structures of the small molecules, the sequences of the target proteins, and the three-dimensional interactions between the two.

**Figure 4:**
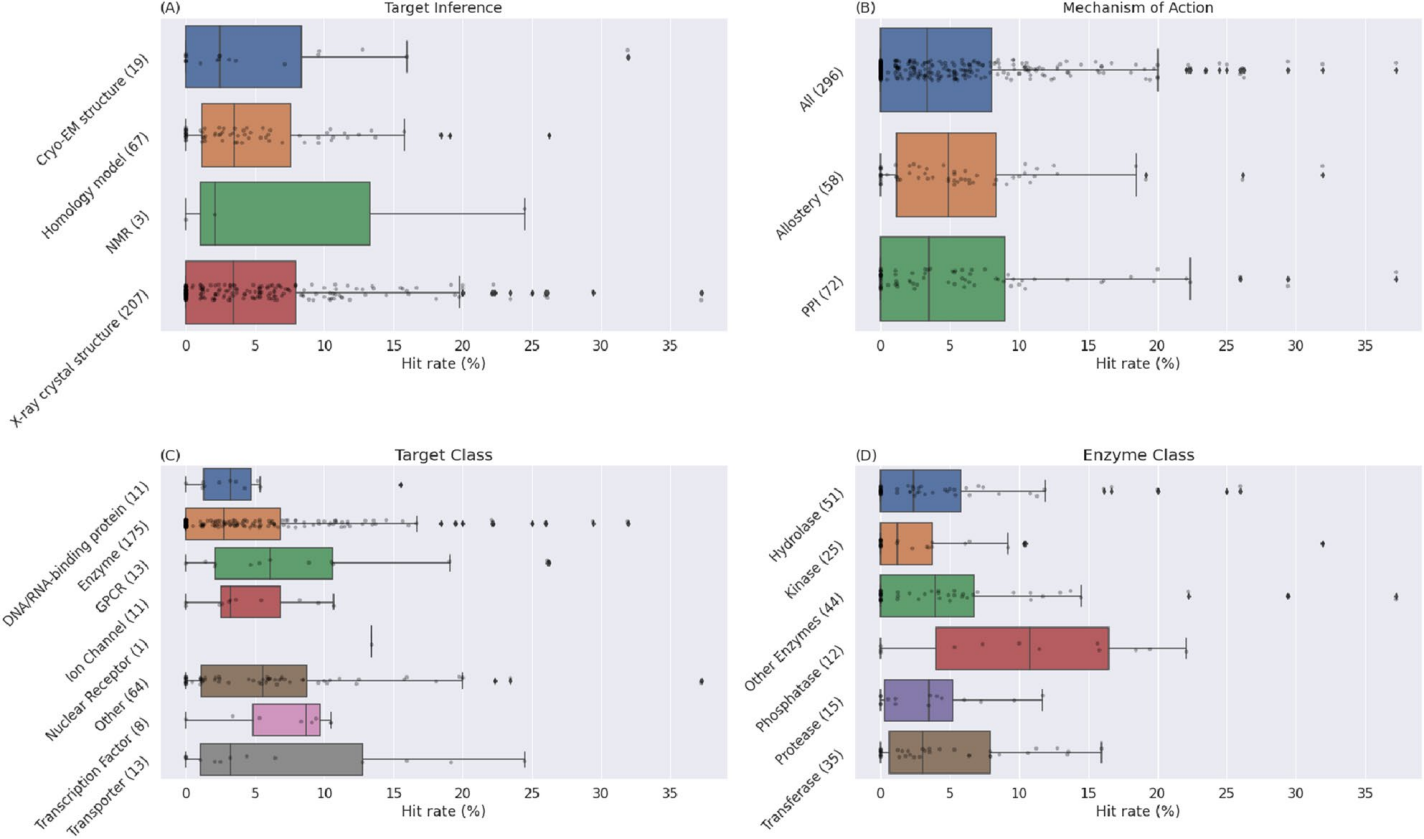
Hit rates obtained for the 296 AIMS projects. (**A**) A comparison of hit rates using X-ray crystallography, NMR, Cryo-EM, and homology for modeling the structure of the proteins. Each point represents a project with the x-axis denoting the hit rate of the project (the percentage of molecules tested in the project that were active). The number of projects of each type is given in parentheses. We observed no substantial difference in success rate between the physical and the computationally inferred models. We achieved average hit rates of 5.6%, 5.5%, and 5.1% for crystal structures, cryo-EM, and homology modeling, respectively. The number of projects using NMR structures is too small to make statistically-robust claims. (**B**) A comparison of hit rates observed for traditionally challenging target classes such as protein–protein interactions (PPI) and allosteric binding. Of the 296 projects, 72 targeted PPIs and 58 allosteric binding sites. The average hit rates were 6.4% and 5.8% for PPIs and allosteric binding, respectively. (**C**) Comparison of hit rates observed for different target classes and (**D**) enzyme classes. No protein or enzyme class falls outside the domain of applicability of the algorithm.

AtomNet also successfully identified active molecules when there was no X-ray crystal structure of the receptor. Figure 4A compares the hit rates obtained with 3-dimensional crystal structures, cryo-EM, and homology modeling. We did not attempt to select targets based on the similarity to the template but rather used the best template available. We observe no substantial difference in success rate between the three, in contrast to the common challenges in using homology models or low-precision structures for structure-based discovery^42,43,73^. We achieved average hit rates of 5.6%, 5.5%, and 5.1% for crystal structures, cryo-EM, and homology modeling. We also successfully identified active compounds in projects with NMR structures, but the number of such targets is too small to make statistically-robust claims.

An interesting demonstration of the robustness of the AtomNet model to low data and poorly characterized protein structure is its ability to identify novel hits for traditionally challenging target classes such as protein–protein interaction (PPI) sites and allosteric binding sites (Fig. 3B). Of the 296 projects, 72 targeted PPIs and 58 allosteric binding sites. We identified hits for 53 (74%) PPI sites and 46 (79%) allosteric sites, with 13 projects representing allosteric sites at PPI interfaces. The average hit rate was 6.4% and 5.8% for PPIs and allosteric binding sites, respectively. The algorithm's success in these target classes, which often suffer from poorly characterized binding sites and a lack of bioactivity training data, is not surprising because Fig. 2A shows that our model is largely not dependent on the availability of on-target training data.

Finally, we investigated whether the algorithm exhibits domain of applicability limitations regarding different protein classes. Figures 4C and 3D illustrate the hit rate observed for each protein and enzyme class. No protein or enzyme class falls outside the domain of applicability of the algorithm, demonstrating that machine learning-based approaches are well-suited as a default technology for new scaffold identification. The hit rate for nuclear receptors is an outlier, with seemingly better accuracy than other classes, but a single data point is not statistically meaningful.

### Dose–response validation studies

We performed additional validation studies for 49 AIMS projects with at least one reported hit. The objective of the validation studies was to establish dose–response (DR) relationships for the single-dose (SD) hits. We describe the protocol of the DR experiments in the Methods section. Briefly, we performed dose–response measurements for the reported hits from the single-dose primary screens. DR was determined using the same assay and screening protocol as the single-dose screens, at the same lab, and with the same personnel. Full dose response curves were obtained in most cases, however in some instances a full curve was not obtained, or concentration dependent activity was qualitatively determined by testing at concentrations other than that for the primary screen. The distribution of assay types and target classes for the projects selected for DR validation also was similar to that of the AIMS projects (Supplementary Fig. S3).

We describe the results of the DR experiments in Supplementary Table S5. In 84% of the experiments, we validated at least one SD hit and got a DR readout. The median activity for the total of 144 DR measurements was 15.4 µM (which compares favorably with HTS^25,74^), of which 13% showed sub-µM potency. Overall, we achieved an average of 2.8 hits per validation study, resulting in a hit rate of 51%. The false positive rate of 49% observed in these experiments is favorably compared to HTS’ which can be as high as 95%^20,75^. This difference in false positive rates may stem from the comparative ease and robustness of the low-throughput assay format we employed versus high-throughput assay. Representative dose–response curves for each of the 49 projects are shown in Supplementary Table S6.

### Analog validation studies

For a subset of 21 projects, we further validated hits with DR activity by testing analogs of the active compounds. In those cases, we used the AtomNet platform to search a purchasable space for additional bioactive compounds chemically analogous to the SD hits. We selected up to 35 additional compounds for testing, including the active compounds from the SD screens.

We describe the results of the analoging experiments in Supplementary Table S7. We identified additional analogs with DR readouts for 16 projects (76%). The median DR activity of the 154 validated analogs was 7.4 µM compared to the median of 15.4 µM of the parent compound (Supplementary Fig. S4).

## Methods

### Screening protocols

#### AIMS screening protocol

We began by evaluating screening libraries of millions of catalog compounds from commercial vendors MCule (10 M)^76^ and Enamine in-stock (2.5 M)^77^. We then selected a drug-like subset via algorithmic filtering by applying Eli Lilly medicinal chemistry filters^78^ and removing likely false positives, such as aggregators, autofluorescers, and PAINS^79^ (see Fig. 2 for the distributions of drug-like properties of the SD hits). The resulting library was virtually screened against the target of interest, removing any molecules with greater than 0.5 Tanimoto similarity in ECFP4 space to any known binders of the target and its homologs within 70% sequence identity. For kinase targets, we extend the exclusion to the whole kinome. The binding site was defined using co-complexes, mutagenesis studies, co-complexes of homologs, or by identifying potential sites using ICM Pocket Finder^80^ or Fpocket^81^. Some were orthosteric, while others were allosteric, or as yet unestablished biological functions. In 64 cases, we built homology models using the closest sequence, with an average sequence similarity of 54%. We clustered the top 30,000 molecules using the Butina^82^ algorithm with a Tanimoto similarity cutoff of 0.35 in ECFP4 space, selecting the highest-scoring exemplars. Additional computed physico-chemical property filters were applied as needed. At no point were compounds cherry-picked. We purchased, on average, 85 compounds, quality controlled by LC–MS to > 90% purity, generally dispensed as 10 mM DMSO stocks plated in a single 96-well plate. In addition, two vials of DMSO-only negative controls were included before scrambling the compound locations on the plate, by the supplier, for blinded experimental testing. To further control for potential artifacts, we removed compounds that showed measurable activity toward more than one target from the analysis.

#### Dose–response and analoging validation screening protocol

We considered advancing AIMS projects to additional validation studies based on the ability to reorder at least some of the initial SD hits, the availability of chemical analogs in the screening library to the initial hits, the capability to perform dose–response experiments, and the ability of the collaborators to perform additional screens and return results promptly.

We performed two sets of experiments: DR validation of the SD hits from AIMS and analoging with DR readouts. We performed DR measurements using the same assays and protocols as SD.

We performed an analoging round by identifying, for each AIMS hit, its 1000 nearest neighbors from the Mcule library^76^, using molecular fingerprints similarity^68^. We augmented the set with additional analogs using substructure^83^ or FTrees^84^ searches, if needed. We used an AtomNet regression model, trained to predict quantitative bioactivities (e.g., IC50 or Ki), to score and rank the analogs. A set of 20—35 compounds from the analogs space of an initial hit were then obtained based on similarity and top scores from the AtomNet model for testing.

#### Internal portfolio screening protocol

We followed a protocol similar to the AIMS screen with a few deviations. First, we used the Enamine REAL library of over 16 billion compounds^62^. Second, we used an ensemble of six AtomNet models for the screens. Last, on average, we selected a set of 440 compounds for testing.

The analoging protocol is similar to the AIMS validation studies, with the following deviations. First, we used the Enamine REAL library for analog search. Second, we selected an average of 676 analogs per project. Third, the analog search protocol was more complex, pulling nearest neighbors based on maximum common substructure and graph edit distance in addition to the ECFP4-based one.

### AtomNet® model architecture

We previously published in detail^52,53,55,58,59,61,85,86^ during the course of the AIMS program, and we described the most recent version of the AtomNet model architecture in detail elsewhere^53^. We provide a brief description below.

The AtomNet model is a Graph Convolution Network architecture with atoms represented as vertices and pair-wise, distance-dependent, edges representing atom proximities. The input is a graph network of features characterizing the atom types and topologies of an ensemble of protein–ligand complexes. Receptor atoms more than 7 Å away from any ligand atom are excluded from the complexes, and each node in the graph is associated with a feature vector representing the atom type using Sybyl typing^87^.

The network has five graph convolutional blocks. In the first two graph convolution blocks, all ligand and receptor atoms 5 Å apart from each other are considered, and 64 filters per block are used. In the third block, the cutoff radius and filters are increased to 7 Å and 128, respectively. Only ligand features in the last two blocks are considered without changing the threshold cutoff or the number of filters. Finally, the sum-pool of the ligand-only layer creates a 3-task layer on top of the network. That multi-task layer predicts three endpoints: bioactivity, pose quality, and a physics-based docking score^88^.

We trained an ensemble of 6 models, splitting the training data into sixfold cross-validation sets based on a protein sequence similarity cutoff of 70%. Then, each model in the ensemble was trained on a different fold for 10 epochs, using the ADAM optimizer^89^ with a learning rate of 0.001, and targets were sampled with replacement, proportional to the number of active compounds associated with that target.

### Data

All data generated or analyzed during this study are included in this published article (and its supplementary information S1 files). Boxplots illustrations show the quartiles (Q1 and Q3) of the dataset while the whiskers extend to show the rest of the distribution, except for points that are determined to be “outliers” (1.5 × of the inter-quartile range, as implemented in the Seaborn and Matplotlib toolboxes^90,91^).

## Conclusion

HTS is the most widely-used tool for hit discovery for new targets. Unfortunately, all physical screening methods share the critical limitation that a molecule must exist to be screened. Computational methods enable a fundamental shift to a test-then-make paradigm. In this work, we report on 318 projects (22 internal projects and 296 collaborations) where we used the AtomNet platform as the primary screening tool coupled with low-throughput physical screens as validation. The AtomNet technology can identify bioactive scaffolds across a wide range of proteins, even without known binders, X-ray structures, or manual cherry-picking of compounds. Our empirical results suggest that machine learning approaches have reached a computational accuracy that can replace HTS as the first step of small-molecule drug discovery.

## Supplementary Information

Supplementary Information 1.

Supplementary Information 2.

## Data Availability

All data generated or analyzed during this study are included in this published article and its supplementary information files.
