# Author Correction: AI is a viable alternative to high throughput screening: a 318-target study

**DOI:** 10.1038/s41598-024-70321-w

**Published:** 2024-09-16

**Authors:** The Atomwise AIMS Program, Izhar Wallach, Denzil Bernard, Kong Nguyen, Gregory Ho, Adrian Morrison, Adrian Stecula, Andreana Rosnik, Ann Marie O’Sullivan, Aram Davtyan, Ben Samudio, Bill Thomas, Brad Worley, Brittany Butler, Christian Laggner, Desiree Thayer, Ehsan Moharreri, Greg Friedland, Ha Truong, Henry van den Bedem, Ho Leung Ng, Kate Stafford, Krishna Sarangapani, Kyle Giesler, Lien Ngo, Michael Mysinger, Mostafa Ahmed, Nicholas J. Anthis, Niel Henriksen, Pawel Gniewek, Sam Eckert, Saulo de Oliveira, Shabbir Suterwala, Srimukh Veccham Krishna PrasadPrasad, Stefani Shek, Stephanie Contreras, Stephanie Hare, Teresa Palazzo, Terrence E. O’Brien, Tessa Van Grack, Tiffany Williams, Ting-Rong Chern, Victor Kenyon, Andreia H. Lee, Andrew B. Cann, Bastiaan Bergman, Brandon M. Anderson, Bryan D. Cox, Jeffrey M. Warrington, Jon M. Sorenson, Joshua M. Goldenberg, Matthew A. Young, Nicholas DeHaan, Ryan P. Pemberton, Stefan Schroedl, Tigran M. Abramyan, Tushita Gupta, Venkatesh Mysore, Adam G. Presser, Adolfo A. Ferrando, Adriano D. Andricopulo, Agnidipta Ghosh, Aicha Gharbi Ayachi, Aisha Mushtaq, Ala M. Shaqra, Alan Kie Leong Toh, Alan V. Smrcka, Alberto Ciccia, Aldo Sena de Oliveira, Aleksandr Sverzhinsky, Alessandra Mara de Sousa, Alexander I. Agoulnik, Alexander Kushnir, Alexander N. Freiberg, Alexander V. Statsyuk, Alexandre R. Gingras, Alexei Degterev, Alexey Tomilov, Alice Vrielink, Alisa A. Garaeva, Amanda Bryant-Friedrich, Amedeo Caflisch, Amit K. Patel, Amith Vikram Rangarajan, An Matheeussen, Andrea Battistoni, Andrea Caporali, Andrea Chini, Andrea Ilari, Andrea Mattevi, Andrea Talbot Foote, Andrea Trabocchi, Andreas Stahl, Andrew B. Herr, Andrew Berti, Andrew Freywald, Andrew G. Reidenbach, Andrew Lam, Andrew R. Cuddihy, Andrew White, Angelo Taglialatela, Anil K. Ojha, Ann M. Cathcart, Anna A. L. Motyl, Anna Borowska, Anna D’Antuono, Anna K. H. Hirsch, Anna Maria Porcelli, Anna Minakova, Anna Montanaro, Anna Müller, Annarita Fiorillo, Anniina Virtanen, Anthony J. O’Donoghue, Antonio Del Rio Flores, Antonio E. Garmendia, Antonio Pineda-Lucena, Antonito T. Panganiban, Ariela Samantha, Arnab K. Chatterjee, Arthur L. Haas, Ashleigh S. Paparella, Ashley L. St. John, Ashutosh Prince, Assmaa ElSheikh, Athena Marie Apfel, Audrey Colomba, Austin O’Dea, Bakary N’tji Diallo, Beatriz Murta Rezende Moraes Ribeiro, Ben A. Bailey-Elkin, Benjamin L. Edelman, Benjamin Liou, Benjamin Perry, Benjamin Soon Kai Chua, Benjámin Kováts, Bernhard Englinger, Bijina Balakrishnan, Bin Gong, Bogos Agianian, Brandon Pressly, Brenda P. Medellin Salas, Brendan M. Duggan, Brian V. Geisbrecht, Brian W. Dymock, Brianna C. Morten, Bruce D. Hammock, Bruno Eduardo Fernandes Mota, Bryan C. Dickinson, Cameron Fraser, Camille Lempicki, Carl D. Novina, Carles Torner, Carlo Ballatore, Carlotta Bon, Carly J. Chapman, Carrie L. Partch, Catherine T. Chaton, Chang Huang, Chao-Yie Yang, Charlene M. Kahler, Charles Karan, Charles Keller, Chelsea L. Dieck, Chen Huimei, Chen Liu, Cheryl Peltier, Chinmay Kumar Mantri, Chinyere Maat Kemet, Christa E. Müller, Christian Weber, Christina M. Zeina, Christine S. Muli, Christophe Morisseau, Cigdem Alkan, Clara Reglero, Cody A. Loy, Cornelia M. Wilson, Courtney Myhr, Cristina Arrigoni, Cristina Paulino, César Santiago, Dahai Luo, Damon J. Tumes, Daniel A. Keedy, Daniel A. Lawrence, Daniel Chen, Danny Manor, Darci J. Trader, David A. Hildeman, David H. Drewry, David J. Dowling, David J. Hosfield, David M. Smith, David Moreira, David P. Siderovski, David Shum, David T. Krist, David W. H. Riches, Davide Maria Ferraris, Deborah H. Anderson, Deirdre R. Coombe, Derek S. Welsbie, Di Hu, Diana Ortiz, Dina Alramadhani, Dingqiang Zhang, Dipayan Chaudhuri, Dirk J. Slotboom, Donald R. Ronning, Donghan Lee, Dorian Dirksen, Douglas A. Shoue, Douglas William Zochodne, Durga Krishnamurthy, Dustin Duncan, Dylan M. Glubb, Edoardo Luigi Maria Gelardi, Edward C. Hsiao, Edward G. Lynn, Elany Barbosa Silva, Elena Aguilera, Elena Lenci, Elena Theres Abraham, Eleonora Lama, Eleonora Mameli, Elisa Leung, Emily M. Christensen, Emily R. Mason, Enrico Petretto, Ephraim F. Trakhtenberg, Eric J. Rubin, Erick Strauss, Erik W. Thompson, Erika Cione, Erika Mathes Lisabeth, Erkang Fan, Erna Geessien Kroon, Eunji Jo, Eva M. García-Cuesta, Evgenia Glukhov, Evripidis Gavathiotis, Fang Yu, Fei Xiang, Fenfei Leng, Feng Wang, Filippo Ingoglia, Focco van den Akker, Francesco Borriello, Franco J. Vizeacoumar, Frank Luh, Frederick S. Buckner, Frederick S. Vizeacoumar, Fredj Ben Bdira, Fredrik Svensson, G. Marcela Rodriguez, Gabriella Bognár, Gaia Lembo, Gang Zhang, Garrett Dempsey, Gary Eitzen, Gaétan Mayer, Geoffrey L. Greene, George A. Garcia, Gergely L. Lukacs, Gergely Prikler, Gian Carlo G. Parico, Gianni Colotti, Gilles De Keulenaer, Gino Cortopassi, Giovanni Roti, Giulia Girolimetti, Giuseppe Fiermonte, Giuseppe Gasparre, Giuseppe Leuzzi, Gopal Dahal, Gracjan Michlewski, Graeme L. Conn, Grant David Stuchbury, Gregory R. Bowman, Grzegorz Maria Popowicz, Guido Veit, Guilherme Eduardo de Souza, Gustav Akk, Guy Caljon, Guzmán Alvarez, Gwennan Rucinski, Gyeongeun Lee, Gökhan Cildir, Hai Li, Hairol E. Breton, Hamed Jafar-Nejad, Han Zhou, Hannah P. Moore, Hannah Tilford, Haynes Yuan, Heesung Shim, Heike Wulff, Heinrich Hoppe, Helena Chaytow, Heng-Keat Tam, Holly Van Remmen, Hongyang Xu, Hosana Maria Debonsi, Howard B. Lieberman, Hoyoung Jung, Hua-Ying Fan, Hui Feng, Hui Zhou, Hyeong Jun Kim, Iain R. Greig, Ileana Caliandro, Ileana Corvo, Imanol Arozarena, Imran N. Mungrue, Ingrid M. Verhamme, Insaf Ahmed Qureshi, Irina Lotsaris, Isin Cakir, J. Jefferson P. Perry, Jacek Kwiatkowski, Jacob Boorman, Jacob Ferreira, Jacob Fries, Jadel Müller Kratz, Jaden Miner, Jair L. Siqueira-Neto, James G. Granneman, James Ng, James Shorter, Jan Hendrik Voss, Jan M. Gebauer, Janelle Chuah, Jarrod J. Mousa, Jason T. Maynes, Jay D. Evans, Jeffrey Dickhout, Jeffrey P. MacKeigan, Jennifer N. Jossart, Jia Zhou, Jiabei Lin, Jiake Xu, Jianghai Wang, Jiaqi Zhu, Jiayu Liao, Jingyi Xu, Jinshi Zhao, Jiusheng Lin, Jiyoun Lee, Joana Reis, Joerg Stetefeld, John B. Bruning, John Burt Bruning, John G. Coles, John J. Tanner, John M. Pascal, Jonathan So, Jordan L. Pederick, Jose A. Costoya, Joseph B. Rayman, Joseph J. Maciag, Joshua Alexander Nasburg, Joshua J. Gruber, Joshua M. Finkelstein, Joshua Watkins, José Miguel Rodríguez-Frade, Juan Antonio Sanchez Arias, Juan José Lasarte, Julen Oyarzabal, Julian Milosavljevic, Julie Cools, Julien Lescar, Julijus Bogomolovas, Jun Wang, Jung-Min Kee, Jung-Min Kee, Junzhuo Liao, Jyothi C. Sistla, Jônatas Santos Abrahão, Kamakshi Sishtla, Karol R. Francisco, Kasper B. Hansen, Kathleen A. Molyneaux, Kathryn A. Cunningham, Katie R. Martin, Kavita Gadar, Kayode K. Ojo, Keith S. Wong, Kelly L. Wentworth, Kent Lai, Kevin A. Lobb, Kevin M. Hopkins, Keykavous Parang, Khaled Machaca, Kien Pham, Kim Ghilarducci, Kim S. Sugamori, Kirk James McManus, Kirsikka Musta, Kiterie M. E. Faller, Kiyo Nagamori, Konrad J. Mostert, Konstantin V. Korotkov, Koting Liu, Kristiana S. Smith, Kristopher Sarosiek, Kyle H. Rohde, Kyu Kwang Kim, Kyung Hyeon Lee, Lajos Pusztai, Lari Lehtiö, Larisa M. Haupt, Leah E. Cowen, Lee J. Byrne, Leila Su, Leon Wert-Lamas, Leonor Puchades-Carrasco, Lifeng Chen, Linda H. Malkas, Ling Zhuo, Lizbeth Hedstrom, Lizbeth Hedstrom, Loren D. Walensky, Lorenzo Antonelli, Luisa Iommarini, Luke Whitesell, Lía M. Randall, M. Dahmani Fathallah, Maira Harume Nagai, Mairi Louise Kilkenny, Manu Ben-Johny, Marc P. Lussier, Marc P. Windisch, Marco Lolicato, Marco Lucio Lolli, Margot Vleminckx, Maria Cristina Caroleo, Maria J. Macias, Marilia Valli, Marim M. Barghash, Mario Mellado, Mark A. Tye, Mark A. Wilson, Mark Hannink, Mark R. Ashton, Mark Vincent C.dela Cerna, Marta Giorgis, Martin K. Safo, Martin St. Maurice, Mary Ann McDowell, Marzia Pasquali, Masfique Mehedi, Mateus Sá Magalhães Serafim, Matthew B. Soellner, Matthew G. Alteen, Matthew M. Champion, Maxim Skorodinsky, Megan L. O’Mara, Mel Bedi, Menico Rizzi, Michael Levin, Michael Mowat, Michael R. Jackson, Mikell Paige, Minnatallah Al-Yozbaki, Miriam A. Giardini, Mirko M. Maksimainen, Monica De Luise, Muhammad Saddam Hussain, Myron Christodoulides, Natalia Stec, Natalia Zelinskaya, Natascha Van Pelt, Nathan M. Merrill, Nathanael Singh, Neeltje A. Kootstra, Neeraj Singh, Neha S. Gandhi, Nei-Li Chan, Nguyen Mai Trinh, Nicholas O. Schneider, Nick Matovic, Nicola Horstmann, Nicola Longo, Nikhil Bharambe, Nirvan Rouzbeh, Niusha Mahmoodi, Njabulo Joyfull Gumede, Noelle C. Anastasio, Noureddine Ben Khalaf, Obdulia Rabal, Olga Kandror, Olivier Escaffre, Olli Silvennoinen, Ozlem Tastan Bishop, Pablo Iglesias, Pablo Sobrado, Patrick Chuong, Patrick O’Connell, Pau Martin-Malpartida, Paul Mellor, Paul V. Fish, Paulo Otávio Lourenço Moreira, Pei Zhou, Pengda Liu, Pengda Liu, Pengpeng Wu, Percy Agogo-Mawuli, Peter L. Jones, Peter Ngoi, Peter Toogood, Philbert Ip, Philipp von Hundelshausen, Pil H. Lee, Rachael B. Rowswell-Turner, Rafael Balaña-Fouce, Rafael Eduardo Oliveira Rocha, Rafael V. C. Guido, Rafaela Salgado Ferreira, Rajendra K. Agrawal, Rajesh K. Harijan, Rajesh Ramachandran, Rajkumar Verma, Rakesh K. Singh, Rakesh Kumar Tiwari, Ralph Mazitschek, Rama K. Koppisetti, Remus T. Dame, Renée N. Douville, Richard C. Austin, Richard E. Taylor, Richard G. Moore, Richard H. Ebright, Richard M. Angell, Riqiang Yan, Rishabh Kejriwal, Robert A. Batey, Robert Blelloch, Robert J. Vandenberg, Robert J. Hickey, Robert J. Kelm, Robert J. Lake, Robert K. Bradley, Robert M. Blumenthal, Roberto Solano, Robin Matthias Gierse, Ronald E. Viola, Ronan R. McCarthy, Rosa Maria Reguera, Ruben Vazquez Uribe, Rubens Lima do Monte-Neto, Ruggiero Gorgoglione, Ryan T. Cullinane, Sachin Katyal, Sakib Hossain, Sameer Phadke, Samuel A. Shelburne, Sandra E. Geden, Sandra Johannsen, Sarah Wazir, Scott Legare, Scott M. Landfear, Senthil K. Radhakrishnan, Serena Ammendola, Sergei Dzhumaev, Seung-Yong Seo, Shan Li, Shan Zhou, Shaoyou Chu, Shefali Chauhan, Shinsaku Maruta, Shireen R. Ashkar, Show-Ling Shyng, Silvestro G. Conticello, Silvia Buroni, Silvia Garavaglia, Simon J. White, Siran Zhu, Sofiya Tsimbalyuk, Somaia Haque Chadni, Soo Young Byun, Soonju Park, Sophia Q. Xu, Sourav Banerjee, Stefan Zahler, Stefano Espinoza, Stefano Gustincich, Stefano Sainas, Stephanie L. Celano, Stephen J. Capuzzi, Stephen N. Waggoner, Steve Poirier, Steven H. Olson, Steven O. Marx, Steven R. Van Doren, Suryakala Sarilla, Susann M. Brady-Kalnay, Sydney Dallman, Syeda Maryam Azeem, Tadahisa Teramoto, Tamar Mehlman, Tarryn Swart, Tatjana Abaffy, Tatos Akopian, Teemu Haikarainen, Teresa Lozano Moreda, Tetsuro Ikegami, Thaiz Rodrigues Teixeira, Thilina D. Jayasinghe, Thomas H. Gillingwater, Thomas Kampourakis, Timothy I. Richardson, Timothy J. Herdendorf, Timothy J. Kotzé, Timothy R. O’Meara, Timothy W. Corson, Tobias Hermle, Tomisin Happy Ogunwa, Tong Lan, Tong Su, Toshihiro Banjo, Tracy A. O’Mara, Tristan Chou, Tsui-Fen Chou, Ulrich Baumann, Umesh R. Desai, Vaibhav P. Pai, Van Chi Thai, Vasudha Tandon, Versha Banerji, Victoria L. Robinson, Vignesh Gunasekharan, Vigneshwaran Namasivayam, Vincent F. M. Segers, Vincent Maranda, Vincenza Dolce, Vinícius Gonçalves Maltarollo, Viola Camilla Scoffone, Virgil A. Woods, Virginia Paola Ronchi, Vuong Van Hung Le, W. Brent Clayton, W. Todd Lowther, Walid A. Houry, Wei Li, Weiping Tang, Wenjun Zhang, Wesley C. Van Voorhis, William A. Donaldson, William C. Hahn, William G. Kerr, William H. Gerwick, William J. Bradshaw, Wuen Ee Foong, Xavier Blanchet, Xiaoyang Wu, Xin Lu, Xin Qi, Xin Xu, Xinfang Yu, Xingping Qin, Xingyou Wang, Xinrui Yuan, Xu Zhang, Yan Jessie Zhang, Yanmei Hu, Yasser Ali Aldhamen, Yicheng Chen, Yihe Li, Ying Sun, Yini Zhu, Yogesh K. Gupta, Yolanda Pérez-Pertejo, Yong Li, Young Tang, Yuan He, Yuk-Ching Tse-Dinh, Yulia A. Sidorova, Yun Yen, Yunlong Li, Zachary J. Frangos, Zara Chung, Zhengchen Su, Zhenghe Wang, Zhiguo Zhang, Zhongle Liu, Zintis Inde, Zoraima Artía, Abraham Heifets, Ellie Giles

**Affiliations:** 1 San Fransico, CA USA; 2 Atomwise Inc., San Fransico, USA; 3 Amgen, Thousand Oaks, USA; 4 OpenAI, San Francisco, USA; 5 Model Medicines, La Jolla, USA; 6 Atomic.AI, San Francisco, USA; 7 Edifice Health, Inc., San Mateo, USA; 8 METiS Therapeutics, Cambridge, USA; 9 Genentech, San Mateo, USA; 10 US Navy Medical Service Corps Officer (2300/1810D), San Mateo, USA; 11 Totus Medicines, Inc., Emeryville, USA; 12 Cytokinetics, Inc., South San Francisco, USA; 13 Nurix Therapeutics, San Francisco, USA; 14 Amazon Alexa, Suite, USA; 15 The University of North Carolina at Chapel Hill Eshelman School of Pharmacy, Chapel Hill, USA; 16 Refibered Inc., Cupertino, USA; 17 NVIDIA, Santa Clara, USA; 18 Harvard TH Chan School of Public Health, Boston, USA; 19 Columbia University, New York, USA; 20 University of São Paulo, São Paulo, Brazil; 21 Albert Einstein College of Medicine, Bronx, USA; 22 Nanyang Technological University, Singapore, Singapore; 23 University of Washington, Seattle, USA; 24 Chan Medical School, University of Massachusetts, Worcester, USA; 25 Queensland University of Technology, Brisbane, Australia; 26 University of Michigan Medical School, Ann Arbor, USA; 27 Columbia University Irving Medical Center, New York, USA; 28 Universidade Federal de Santa Catarina, Florianópolis, Brazil; 29 Université de Montréal, Montreal, Canada; 30 Instituto René Rachou-Fundação Oswaldo Cruz/Fiocruz Minas, Belo Horizonte, Brazil; 31 Herbert Wertheim College of Medicine, Biomolecular Science Institute, Florida International University, Miami, USA; 32 NYU Langone Health, New York, USA; 33 The University of Texas Medical Branch at Galveston, Galveston, USA; 34 University of Houston, Galveston, USA; 35 University of California, San Diego, USA; 36 School of Medicine, Tufts University, Medford, USA; 37 University of California, Davis, Davis, USA; 38 University of Western Australia, Crawley, Australia; 39 University of Groningen, Groningen, The Netherlands; 40 Wayne State University, Detroit, USA; 41 University of Zurich, Zürich, Switzerland; 42 Stanford University, Stanford, USA; 43 University of Antwerp, Antwerp, Belgium; 44 University of Rome Tor Vergata, Rome, Italy; 45 University of Edinburgh, Edinburgh, UK; 46 Department of Plant Molecular Genetics, Centro Nacional de Biotecnología, Consejo Superior de Investigaciones Científicas (CNB-CSIC), Madrid, Spain; 47 CNR (Italian National Research Council), Rome, Italy; 48 University of Pavia, Pavia, Italy; 49 University of Vermont, Burlington, USA; 50 University of Florence, Florence, Italy; 51 University of California, Berkeley, Berkeley, USA; 52 Cincinnati Children’s Hospital Medical Center, Cincinnati, USA; 53 University of Saskatchewan, Saskatoon, Canada; 54 Broad Institute of MIT and Harvard, Cambridge, USA; 55 Boston University, Boston, USA; 56 CancerCare Manitoba Research Institute, Winnipeg, Canada; 57 University of Michigan, Ann Arbor, USA; 58 Wadsworth Center, New York State Department of Health and University at Albany, Albany, USA; 59 Dana-Farber Cancer Institute, Boston, USA; 60 University of Parma, Parma, Italy; 61 Helmholtz Institute for Pharmaceutical Research Saarland, Saarbrücken, Germany; 62 University of Bologna, Bologna, Italy; 63 Sapienza University of Rome, Rome, Italy; 64 Tampere University, Tampere, Finland; 65 University of Connecticut, Storrs, USA; 66 Centro de Investigación Médica Aplicada, Universidad de Navarra, Pamplona, Spain; 67 Tulane National Primate Research Center, Tulane University, Covington, USA; 68 Scripps Research, San Diego, USA; 69 Louisiana State University School of Medicine, New Orleans, USA; 70 Duke-NUS Medical School, Singapore, Singapore; 71 Case Western Reserve University, Cleveland, USA; 72 Oregon Health and Science University and Tanta University in Tanta, Tanta, Egypt; 73 University College London, London, UK; 74 Saint Louis University, St. Louis, USA; 75 Rhodes University, Makhanda, South Africa; 76 Universidade Federal de Minas Gerais (UFMG), Belo Horizonte, Brazil; 77 University of Manitoba, Winnipeg, Canada; 78 National Jewish Health, Denver, USA; 79 Drugs for Neglected Diseases Initiative (DNDi), Geneva, Switzerland; 80 The University of Adelaide, Adelaide, Australia; 81 Mcule, Budapest, Hungary; 82 University of Utah, Salt Lake City, USA; 83 The University of Texas at Austin, Austin, USA; 84 Kansas State University, Manhattan, USA; 85 UniQuest Pty Ltd, St Lucia, Australia; 86 University of Chicago, Chicago, USA; 87 Harvard University, Cambridge, USA; 88 University of Freiburg, Freiburg Im Breisgau, Germany; 89 Dana-Farber Cancer Institute and Harvard Medical School, Boston, USA; 90 IRB Barcelona, Barcelona, Spain; 91 Istituto Italiano Di Tecnologia, Genoa, Italy; 92 QIMR Berghofer Medical Research Institute, Herston, Australia; 93 University of California, Santa Cruz, Santa Cruz, USA; 94 University of Kentucky, Lexington, USA; 95 University of Tennessee Health Science Center, Memphis, USA; 96 Children’s Cancer Therapy Development Institute, Beaverton, USA; 97 Columbia University Medical Center, New York, USA; 98 Yale School of Medicine, New Haven, USA; 99 University of Bonn, Bonn, Germany; 100 Ludwig-Maximilians-Universität München, Munich, Germany; 101 Purdue University, West Lafayette, USA; 102 Canterbury Christ Church University, Canterbury, UK; 103 National Centre for Biotechnology (CNB-CSIC), Madrid, Spain; 104 University of South Australia and SA Pathology, Adelaide, Australia; 105 CUNY Advanced Science Research Center, New York, USA; 106 The University of Toledo, Toledo, USA; 107 University of North Carolina at Chapel Hill, Chapel Hill, USA; 108 Boston Children’s Hospital and Harvard Medical School, Boston, USA; 109 West Virginia University, Morgantown, USA; 110 Universidade de Santiago de Compostela, Santiago, Spain; 111 University of North Texas Health Science Center at Fort Worth, Fort Worth, USA; 112 Institut Pasteur Korea, Seongnam, South Korea; 113 Carle Illinois College of Medicine, Urbana, USA; 114 Università del Piemonte Orientale, Vercelli, Italy; 115 Saskatchewan Cancer Agency, Saskatoon, Canada; 116 Curtin University, Bentley, Australia; 117 Oregon Health and Science University, Portland, USA; 118 Virginia Commonwealth University, Richmond, USA; 119 Tufts University, Medford, USA; 120 University of Nebraska Medical Center, Omaha, USA; 121 University of Louisville, Louisville, USA; 122 Dana Farber Cancer Institute, Boston, USA; 123 University of Notre Dame, Notre Dame, USA; 124 University of Alberta, Edmonton, Canada; 125 University of Toronto, Toronto, Canada; 126 University of Piemonte Orientale, Vercelli, Italy; 127 University of California, San Francisco, San Francisco, USA; 128 St. Joseph’s Healthcare Hamilton, and Hamilton Center for Kidney Research, McMaster University, Hamilton, Canada; 129 Skaggs School of Pharmacy and Pharmaceutical Sciences, University of California San Diego, San Diego, USA; 130 Universidad de La República, Montevideo, Uruguay; 131 University of Cologne, Cologne, Germany; 132 Johnson University, Knoxville, USA; 133 Indiana University, Bloomington, USA; 134 School of Medicine, University of Connecticut, Farmington, USA; 135 Stellenbosch University, Stellenbosch, South Africa; 136 University of Calabria, Arcavacata, Italy; 137 Michigan State University, East Lansing, USA; 138 University of Washington, Washington, USA; 139 Weill Cornell Medicine-Qatar, Ar-Rayyan, Qatar; 140 Gachon University, Seongnam, South Korea; 141 Florida International University, Miami, USA; 142 California Institute of Technology, Pasadena, USA; 143 Boston Children’s Hospital, Boston, USA; 144 Saskatchewan Cancer Agency and University of Saskatchewan, Saskatchewan, Canada; 145 Sino-American Cancer Foundation, Covina, USA; 146 Leiden University, Leiden, The Netherlands; 147 Rutgers University, Newark, USA; 148 Core Research Laboratory, ISPRO, Florence, Italy; 149 Caltech, Pasadena, USA; 150 University of Alberta, Edmonton, USA; 151 Montreal Heart Institute and Université de Montréal, Montreal, Canada; 152 McGill University, Montreal, Canada; 153 Antwerp University, Antwerp, Belgium; 154 University of Bari Aldo Moro, Bari, Italy; 155 Alma Mater Studiorum-University of Bologna, Bologna, Italy; 156 University of Toledo, Toledo, USA; 157 International Institute of Molecular and Cell Biology in Warsaw, Warsaw, Poland; 158 Infection Medicine, University of Edinburgh The Chancellor’s Building, Edinburgh, UK; 159 Emory University, Atlanta, USA; 160 University of Pennsylvania, Philadelphia, USA; 161 Helmholtz Zentrum München, Munich, Germany; 162 Washington University School of Medicine, St. Louis, USA; 163 CENUR Litoral Norte, Universidad de La República, Montevideo, Uruguay; 164 University of Southampton, Southampton, UK; 165 Centre for Cancer Biology, University of South Australia, Adelaide, Australia; 166 University of Missouri, Columbia, USA; 167 Baylor College of Medicine, Houston, USA; 168 Yale University, New Haven, USA; 169 Reno School of Medicine, University of Nevada, Reno, USA; 170 University of Manitoba and CancerCare Manitoba, Winnipeg, Canada; 171 Goethe University Frankfurt, Frankfurt, Germany; 172 Oklahoma Medical Research Foundation/Oklahoma City VA Medical Center, Oklahoma City, USA; 173 Oklahoma Medical Research Foundation, Oklahoma City, USA; 174 Department of Biomolecular Sciences, School of Pharmaceutical Sciences of Ribeirão Preto, University of São Paulo, Ribeirão Preto, SP Brazil; 175 Ulsan National Institute of Science and Technology, Ulsan, South Korea; 176 University of New Mexico Comprehensive Cancer Center, Albuquerque, USA; 177 Ulsan National Institute of Science and Technology (UNIST), Ulsan, South Korea; 178 University of Aberdeen, Aberdeen, UK; 179 University of Turin, Turin, Italy; 180 Universidad de La República, CenUR LN, Montevideo, Uruguay; 181 Navarrabiomed-IdiSNA, Pamplona, Spain; 182 Independent, Los Angeles, USA; 183 Vanderbilt University Medical Center, Nashville, USA; 184 University of Hyderabad, Hyderabad, India; 185 University of Sydney, Sydney, Australia; 186 City of Hope Medical Center, Duarte, USA; 187 Weill Cornell Medicine, New York, NY 10065 USA; 188 University of Toledo College of Medicine and Life Sciences, Toledo, USA; 189 School of Medicine, Wayne State University, Detroit, USA; 190 University of Georgia, Athens, USA; 191 The Hospital for Sick Children, Toronto, Canada; 192 United States Department of Agriculture, Agricultural Research Service (USDA-ARS), Washington, DC USA; 193 McMaster University, Hamilton, Canada; 194 University of California, Riverside, Riverside, USA; 195 The University of Western Australia, Perth, Australia; 196 The University of Connecticut, Storrs, USA; 197 Duke University School of Medicine, Durham, USA; 198 University of Nebraska-Lincoln, Lincoln, USA; 199 Sungshin University, Seoul, South Korea; 200 University of Adelaide, Adelaide, Australia; 201 University Toronto, Toronto, Canada; 202 University of Texas Southwestern Medical Center, Dallas, USA; 203 Centro Nacional de Biotecnologia/CSIC, Madrid, Spain; 204 Centro de Investigación Médica Aplicada, Pamplona, Spain; 205 Centro de Investigación Médica Aplicada, Universidad de Navarra, Pamplona, Spain; 206 University of Wisconsin-Madison, Madison, USA; 207 Indiana University School of Medicine, Indianapolis, USA; 208 University of Montana, Missoula, USA; 209 Brunel University London, London, UK; 210 Chapman University, Orange, USA; 211 Weill Cornell Medicine Qatar, Ar-Rayyan, Qatar; 212 Université du Québec À Montréal, Montreal, Canada; 213 National Taiwan University, Taipei, Taiwan; 214 Rhodes College, Memphis, USA; 215 Harvard School of Public Health, Boston, USA; 216 University of Central Florida, Orlando, USA; 217 University of Rochester, Rochester, USA; 218 George Mason University, Fairfax, USA; 219 University of Oulu, Oulu, Finland; 220 Instituto Investigación Sanitaria La Fe, Valencia, Spain; 221 Ludwig-Maximilians-University, Munich, Germany; 222 Brandeis University, Waltham, USA; 223 Universidad de La República, CENUR Litoral Norte, Montevideo, Uruguay; 224 Arabian Gulf University, Manama, Bahrain; 225 University of Cambridge, Cambridge, UK; 226 University of Magna Graecia, Catanzaro, Italy; 227 Massachusetts General Hospital, Boston, USA; 228 University of Missouri-Columbia, Columbia, USA; 229 Marquette University, Milwaukee, USA; 230 University of North Dakota, Grand Forks, USA; 231 Simon Fraser University, Burnaby, Canada; 232 CancerCare Manitoba Research Institute (CCMR), Winnipeg, Canada; 233 The University of Queensland, Brisbane, Australia; 234 University of Manitoba and CancerCare Manitoba Research Institute, Winnipeg, Canada; 235 Sanford Burnham Prebys, La Jolla, USA; 236 University of Amsterdam, Amsterdam, The Netherlands; 237 UConn Health, Farmington, USA; 238 The University of Texas MD Anderson Cancer Center, Houston, USA; 239 Walter Sisulu University, Mthatha, South Africa; 240 Virginia Tech, Blacksburg, USA; 241 University of Houston, Houston, USA; 242 Rutgers University, New Brunswick, USA; 243 University of Nevada, Reno, USA; 244 Universidad de León, León, Spain; 245 School of Medicine, Case Western Reserve University, Cleveland, USA; 246 School of Medicine, UConn Health, Farmington, USA; 247 University of Rochester Medical Center, Rochester, USA; 248 Chapman University School of Pharmacy, Irvine, USA; 249 University of Winnipeg/St. Boniface Research Centre, Winnipeg, Canada; 250 Fred Hutchinson Cancer Center, Seattle, USA; 251 Helmholtz Institute for Pharmaceutical Research Saarland (HIPS), Saarbrücken, Germany; 252 Technical University of Denmark, Kongens Lyngby, Denmark; 253 The City College of New York, New York, USA; 254 Children’s Cancer, Therapy Development Institute (Cc-TDI), Beaverton, USA; 255 Soka University, Hachioji, Japan; 256 Institute of Clinical Physiology, National Research Council, Pisa, Italy; 257 Charles Sturt University, Bathurst, Australia; 258 Washington University, St Louis, USA; 259 University of Dundee, Dundee, UK; 260 Montreal Heart Institute, Montreal, Canada; 261 Columbia University Vagelos College of Physicians and Surgeons, Columbia, USA; 262 Georgetown University, Washington, USA; 263 Duke University, Durham, USA; 264 Center for Applied Medical Research, University of Navarra, Pamplona, Spain; 265 King’s College London, London, UK; 266 Precision Vaccines Program, Division of Infectious Diseases, Boston Children’s Hospital, Boston, USA; 267 Fred Hutchinson Cancer Research Center, Seattle, USA; 268 Louisiana State University, Baton Rouge, USA; 269 Massey University, Palmerston North, New Zealand; 270 Wake Forest University School of Medicine, Winston-Salem, USA; 271 Central South University, Changsha, China; 272 SUNY Upstate Medical University, Syracuse, USA; 273 University of Oxford, Oxford, UK; 274 Goethe-University, Frankfurt, Frankfurt, Germany; 275 Institute for Cardiovascular Prevention (IPEK), Ludwig-Maximilians-Universität München, Munich, Germany; 276 Harvard T.H. Chan School of Public Health, Boston, USA; 277 School of Medicine, Boston University, Boston, USA; 278 University of Texas Health Science Center at San Antonio, San Antonio, USA; 279 University of Helsinki, Helsinki, Finland; 280 Wadsworth Center, NYSDOH, Albany, USA; 281 The University of Sydney, Sydney, Australia

Correction to: *Scientific Reports* 10.1038/s41598-024-54655-z, published online 02 April 2024

The original version of this Article contained errors.

In the original version of this article, Ellie Giles was omitted from the Author list.

Additionally, the following Affiliation information has been updated:

1. Affiliation 25 was incorrect.

Affiliation 25

‘Queensland University of Technology, Brisbane, USA.’

now reads,

‘Queensland University of Technology, Brisbane, Australia.’

2. Marta Giorgis was incorrectly affiliated with the ‘University of Aberdeen, Aberdeen, UK.’

The correct Affiliation is listed below:

‘University of Turin, Turin, Italy.’

3. Affiliations 52, 125 and 261 were duplicated.

As a result, the correct Affiliation for Andrew B. Herr, Benjamin Liou, David A. Hildeman, Joseph J. Maciag, Ying Sun, Durga Krishnamurthy, and Stephen N. Waggoner is:

‘Cincinnati Children’s Hospital Medical Center, Cincinnati, USA.’

Furthermore, an outdated version of Figure 1 was typeset. The original Figure 1 and accompanying legend appear below.

**Figure 1:**
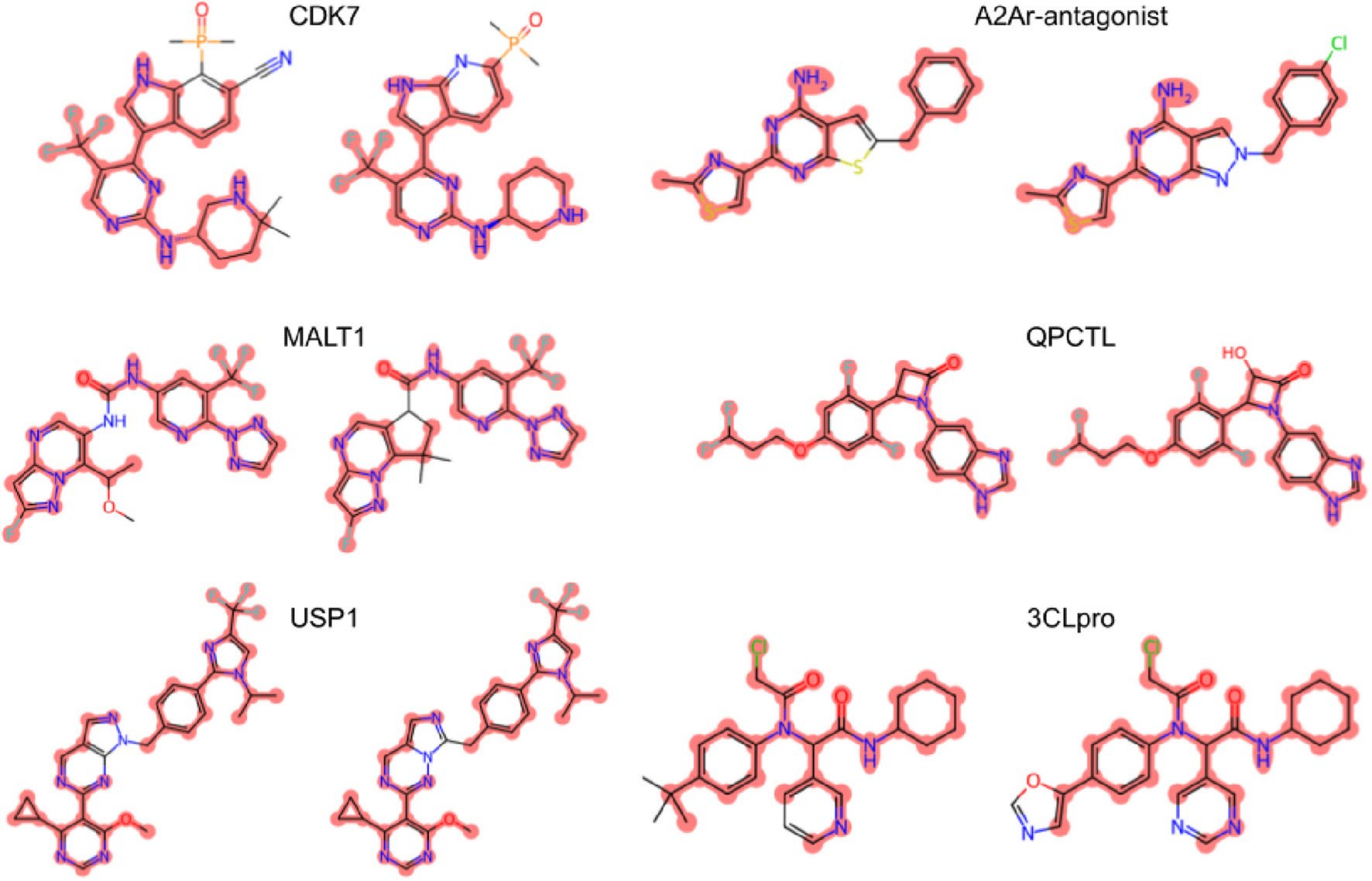
Pairs of representative compounds extracted from AI patents (right) and corresponding prior patents (left) for clinical-stage programs (CDK7^92,93^, A2Ar-antagonist^94,95^, MALT1^96,97^, QPCTL^98,99^, USP1^100,101^, and 3CLpro^102,103^). The identical atoms between the chemical structures are highlighted in red.

Lastly, The Acknowledgements section contained an error.

“See Supplementary section S1.”

now reads,

“See Supplementary section S2.”

The original Article has been corrected.

