# Supplementary material for: AI is a viable alternative to high throughput screening: a 318-target study
Source: Sci Rep. 2024 Apr 2;14:7526. doi: 10.1038/s41598-024-54655-z (PMC10987645; doi:10.1038/s41598-024-54655-z)

MaxPeak: 100.00%
Ret_Time: 0.922 min

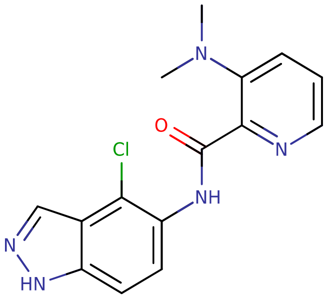


**Mol Wt** 315.76

**Exact Mass** 315.1

| # | Time | Area% |
|---|---|---|
| 1 | 0.922 | 100.00 |

# U267892$2

DAD1 A, Sig=215,16 Ref=off (D:\D\12_30\L321530D\SAMPL000002.D)

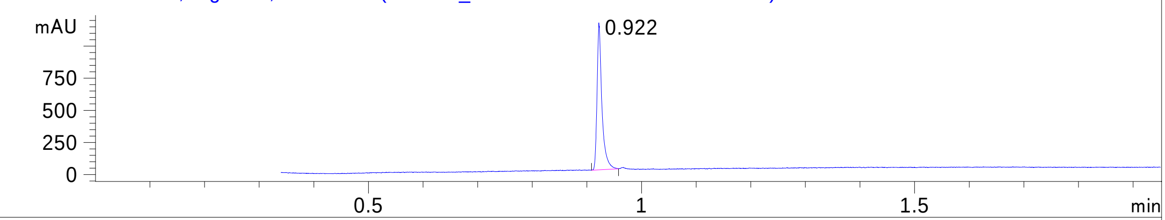


DAD1 B, Sig=254,16 Ref=off (D:\D\12_30\L321530D\SAMPL000002.D)

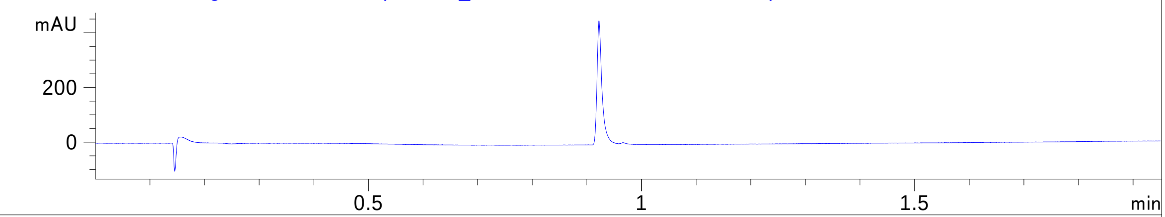


MSD1 TIC, MS File (D:\D\12_30\L321530D\SAMPL000002.D) ES-API, Scan, Frag: 100, "POS"

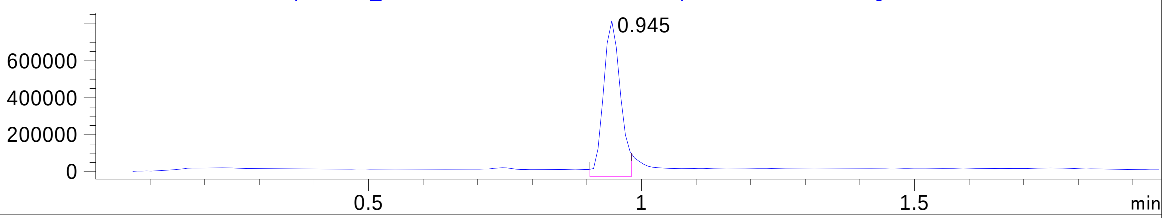


MSD2 TIC, MS File (D:\D\12_30\L321530D\SAMPL000002.D) ES-API, Scan, Frag: 100, "NEG"

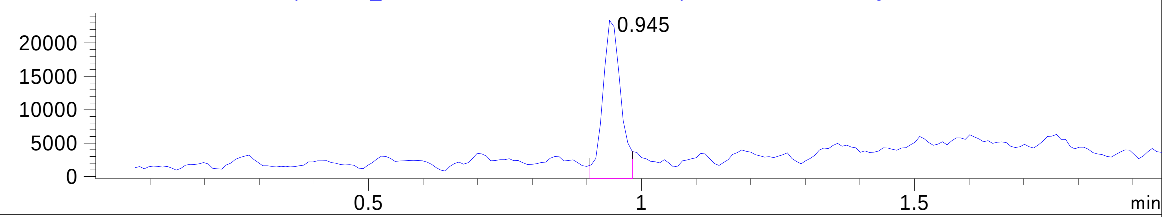


ADC1 A, ADC1 (D:\D\12_30\L321530D\SAMPL000002.D)

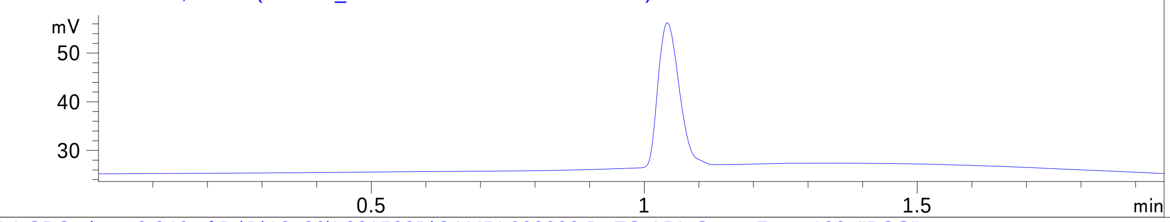


*MSD1 SPC, time=0.946 of D:\D\12_30\L321530D\SAMPL000002.D ES-API, Scan, Frag: 100, "POS"

RT 0.945

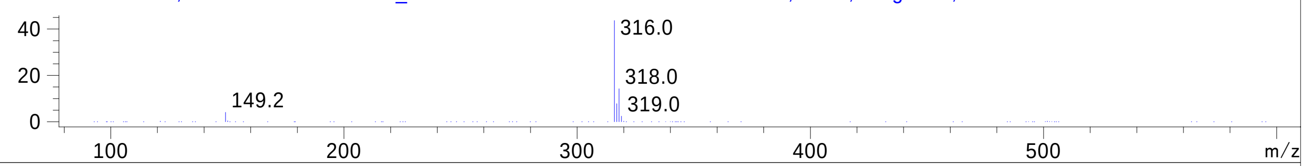


*MSD2 SPC, time=0.941 of D:\D\12_30\L321530D\SAMPL000002.D ES-API, Scan, Frag: 100, "NEG"

RT 0.945

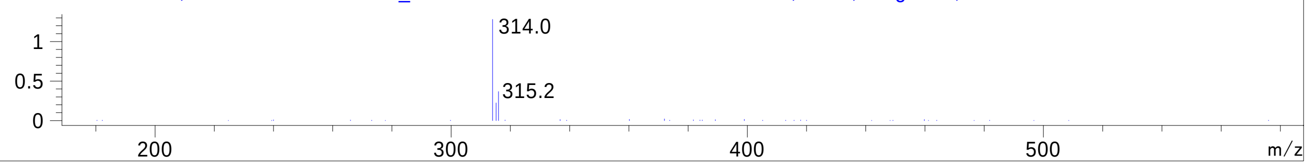

Supplement: Supplementary file 1 — Supplementary Information 1. [file 41598_2024_54655_MOESM1_ESM.zip › Nature SREP/QC_AIDD_cs_selected/LATS1_HID_2_LCMS.pdf]
