# Supplementary material for: AI is a viable alternative to high throughput screening: a 318-target study
Source: Sci Rep. 2024 Apr 2;14:7526. doi: 10.1038/s41598-024-54655-z (PMC10987645; doi:10.1038/s41598-024-54655-z)
Supplement: Supplementary file 1 — Supplementary Information 1. [file 41598_2024_54655_MOESM1_ESM.zip › Nature SREP/QC_AIDD_cs_selected/LATS1_HID_5_LCMS.pdf]

MaxPeak: 97.31%
Ret_Time: 0.746 min

U753165$4

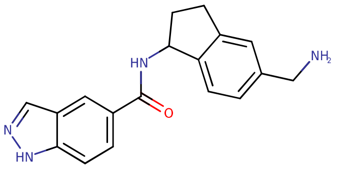

Mol Wt 306.36

Exact Mass 306.17

#	Time	Area%
1	0.746	97.31
2	0.844	1.33
3	0.984	1.36

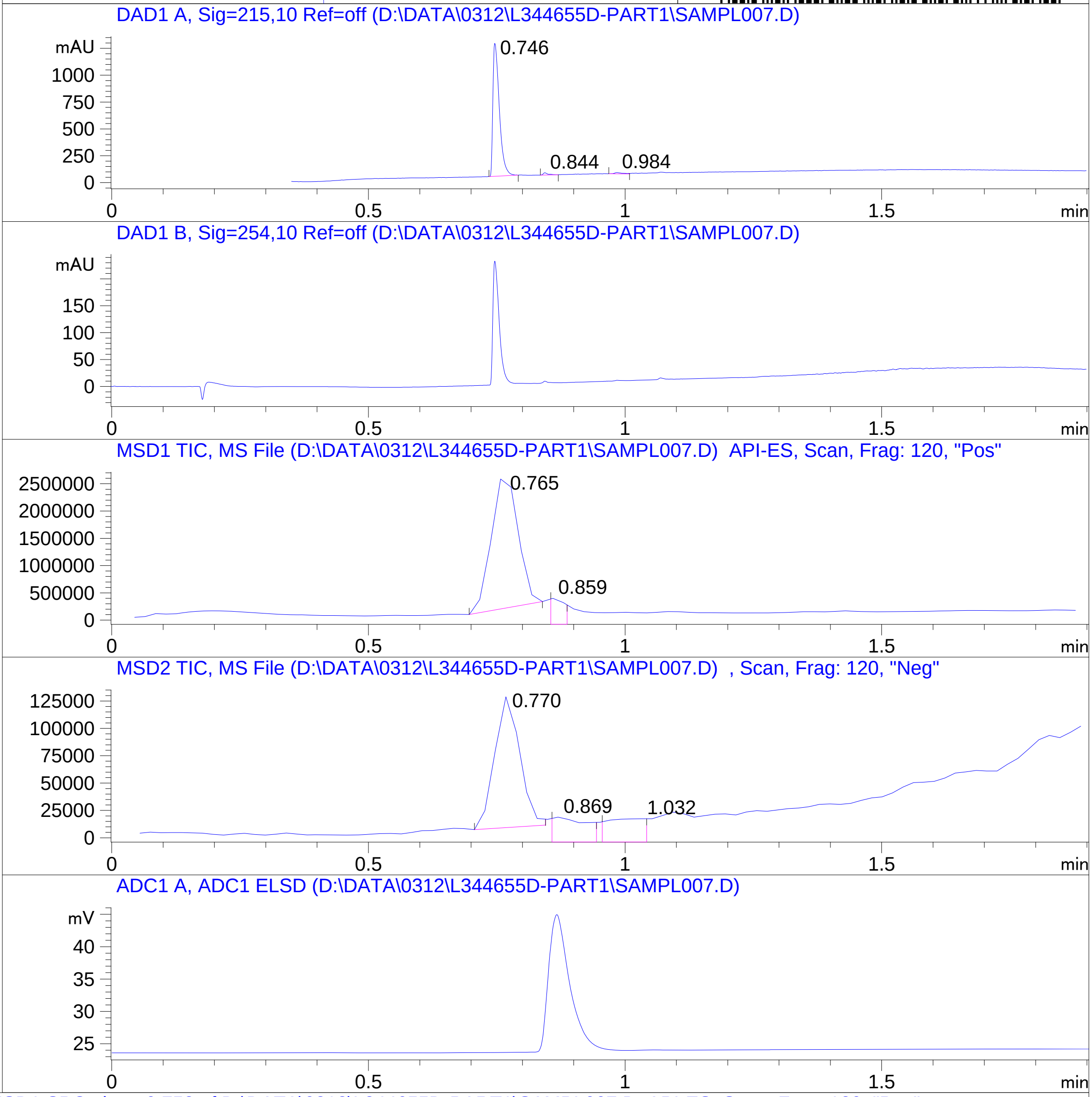

RT 0.765

RT 0.859

RT 0.770

RT 0.869

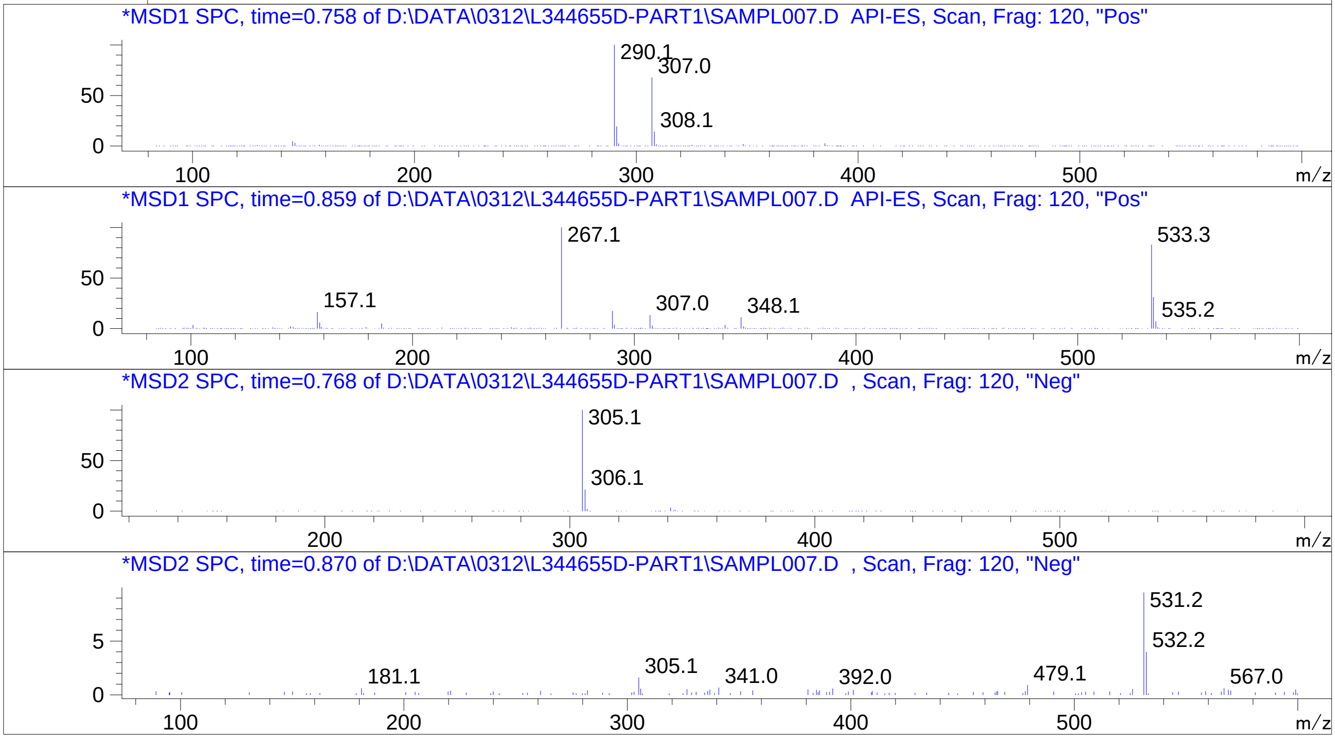

Inj.Date 3/12/2021 OA -VL- Acq. Method C:\HPCHEM\-> ->

RT 1.032

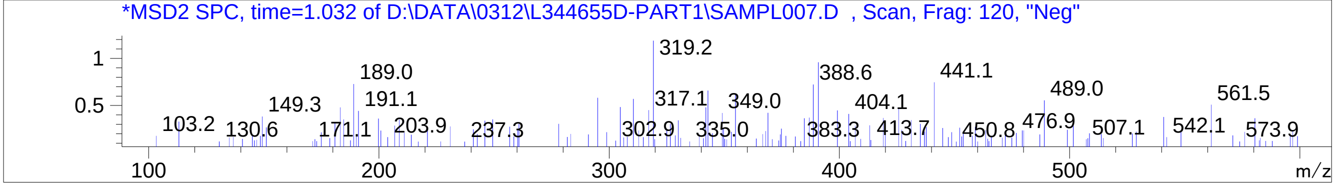

Inj.Date 3/12/2021 OA -VL- Acq. Method C:\HPCHEM\-> ->
