# Supplementary material for: AI is a viable alternative to high throughput screening: a 318-target study
Source: Sci Rep. 2024 Apr 2;14:7526. doi: 10.1038/s41598-024-54655-z (PMC10987645; doi:10.1038/s41598-024-54655-z)

MaxPeak: 99.10%
Ret_Time: 0.884 min

U267611$1

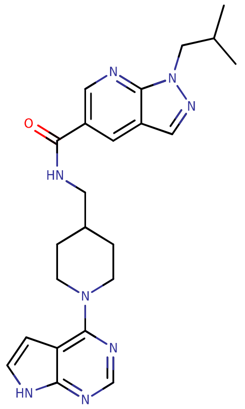

Mol Wt 432.52

Exact Mass 432.27

#	Time	Area%
1	0.884	99.10
2	1.332	0.90

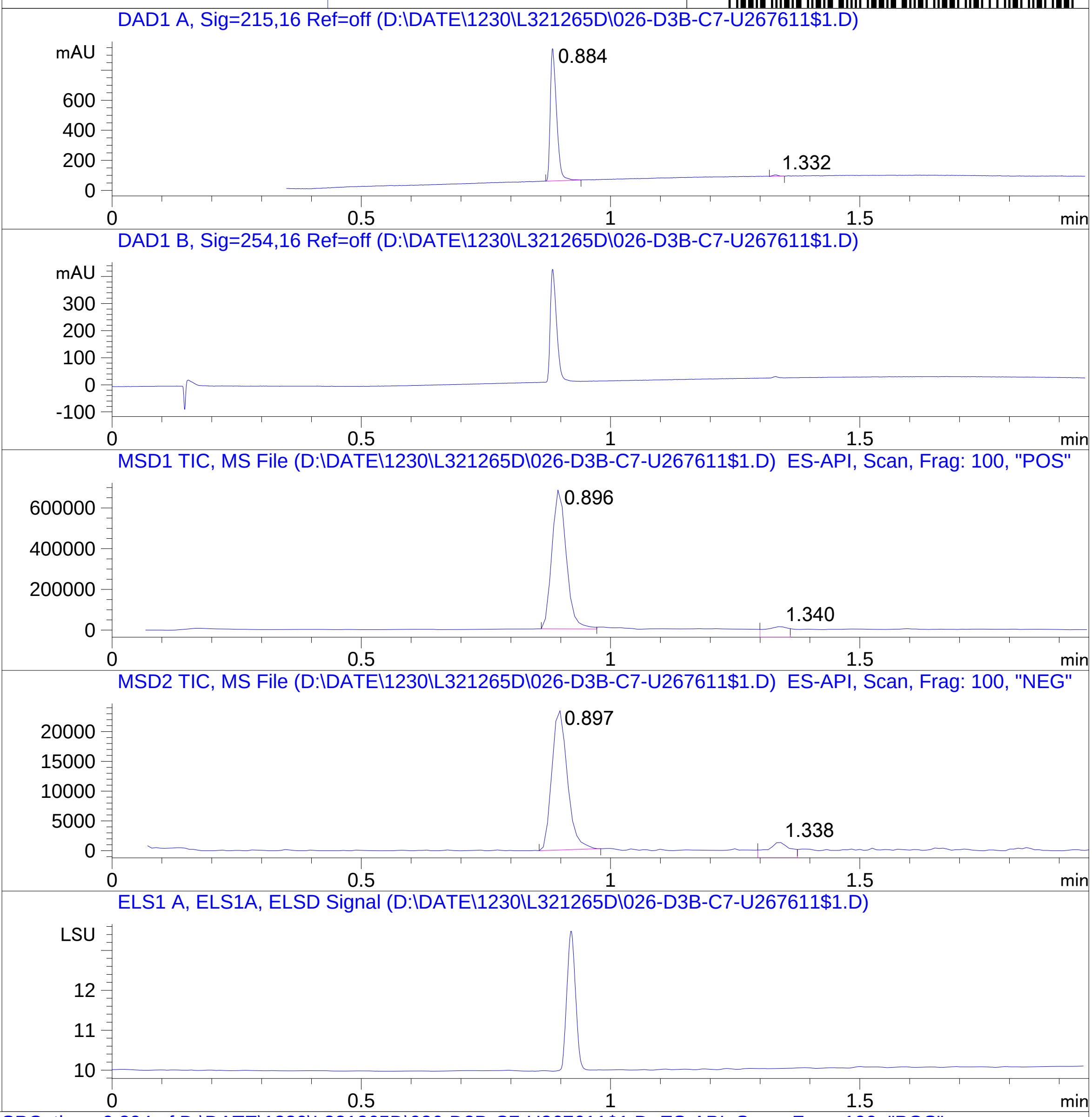

RT 0.896

RT 1.340

RT 0.897

RT 1.338

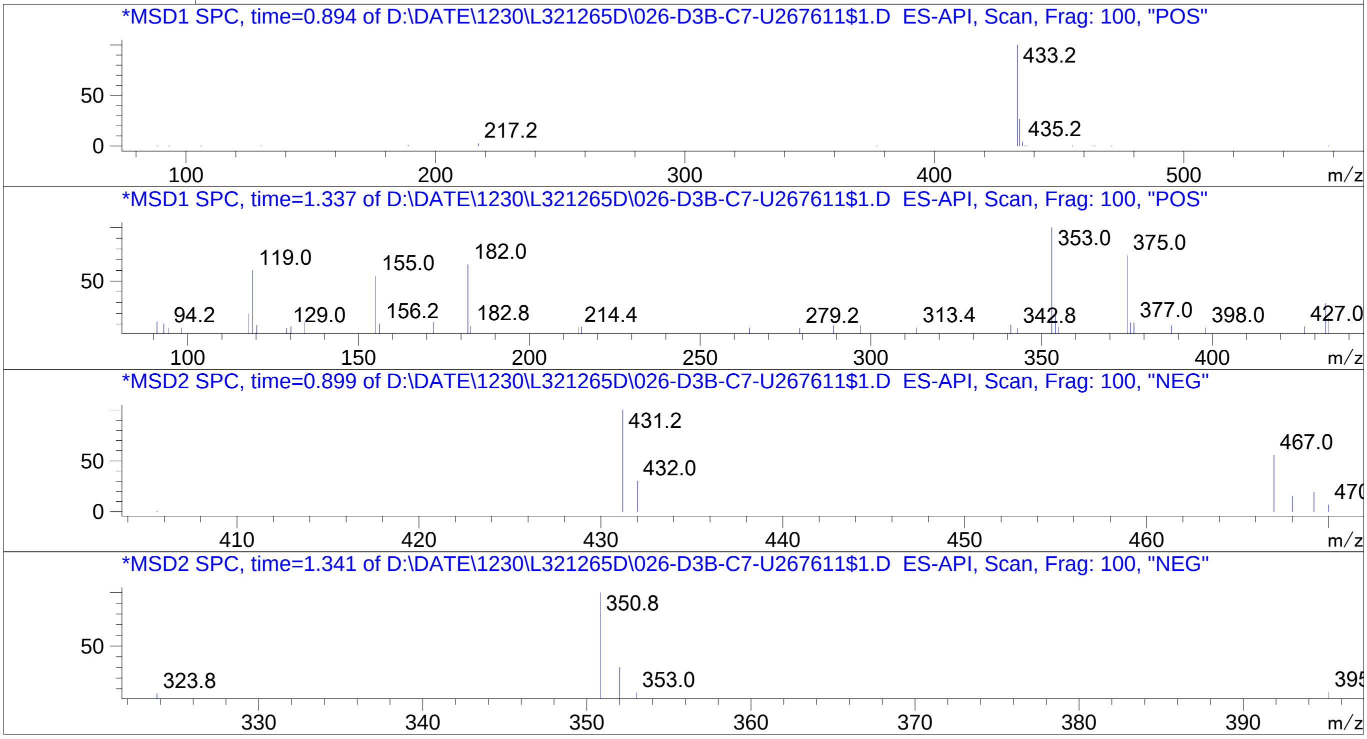

Supplement: Supplementary file 1 — Supplementary Information 1. [file 41598_2024_54655_MOESM1_ESM.zip › Nature SREP/QC_AIDD_cs_selected/LATS1_HID_6_LCMS.pdf]
