# Supplementary material for: AI is a viable alternative to high throughput screening: a 318-target study
Source: Sci Rep. 2024 Apr 2;14:7526. doi: 10.1038/s41598-024-54655-z (PMC10987645; doi:10.1038/s41598-024-54655-z)

MaxPeak: 100.00%
Ret_Time: 1.242 min

U753327$2

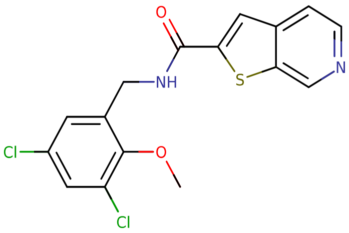

Mol Wt 367.25

Exact Mass 366.01

#	Time	Area%
1	1.242	100.00

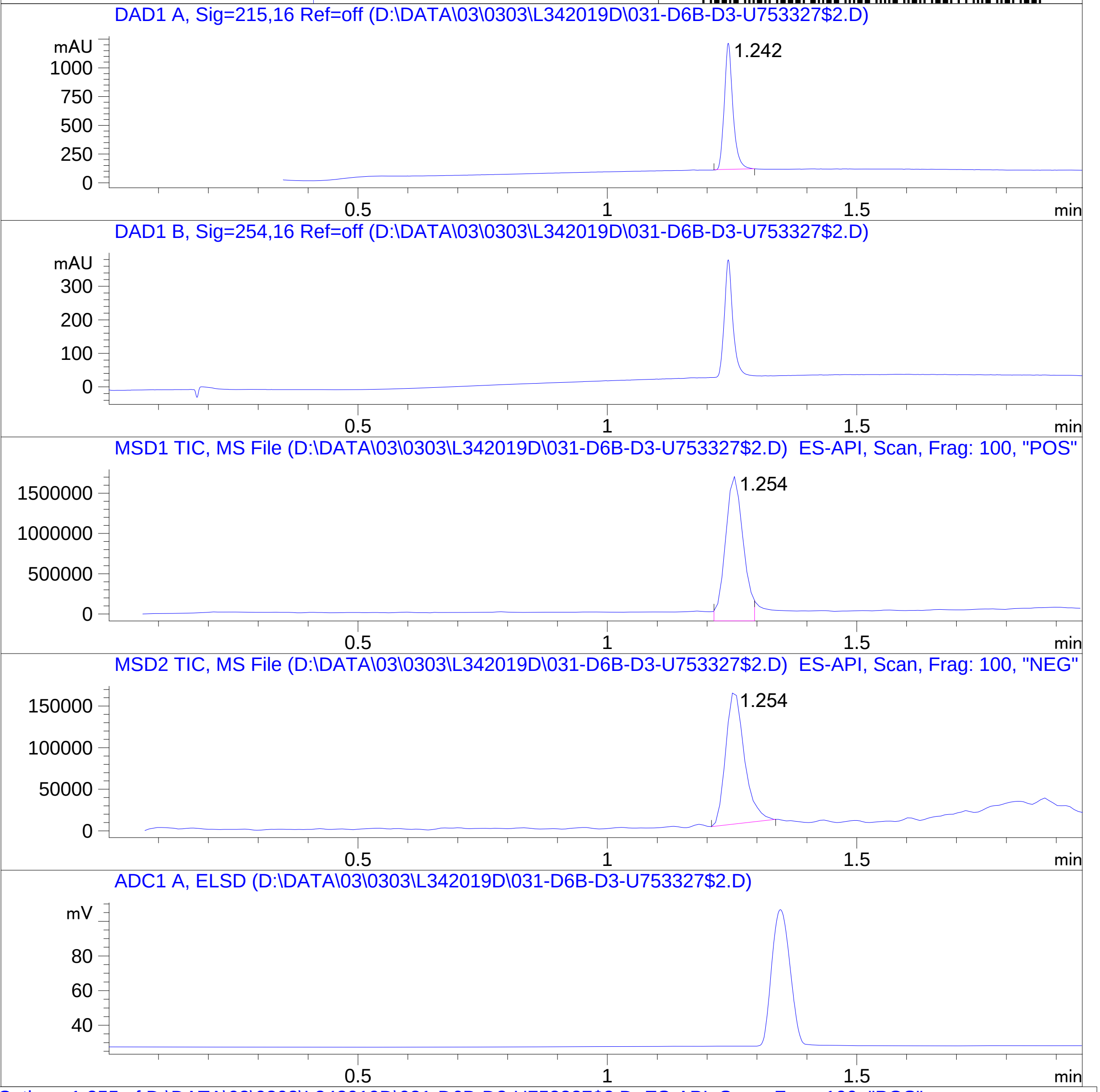

RT 1.254

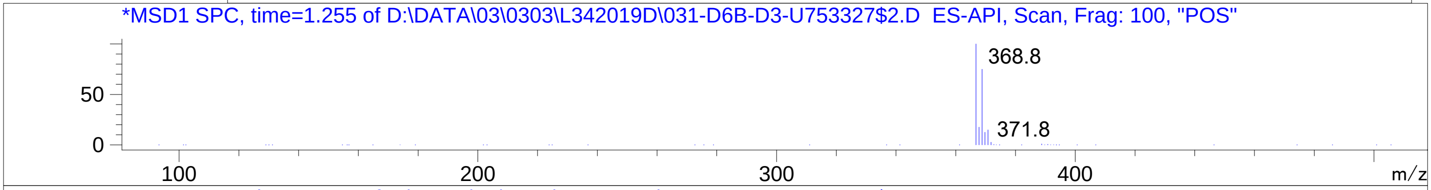

RT 1.254

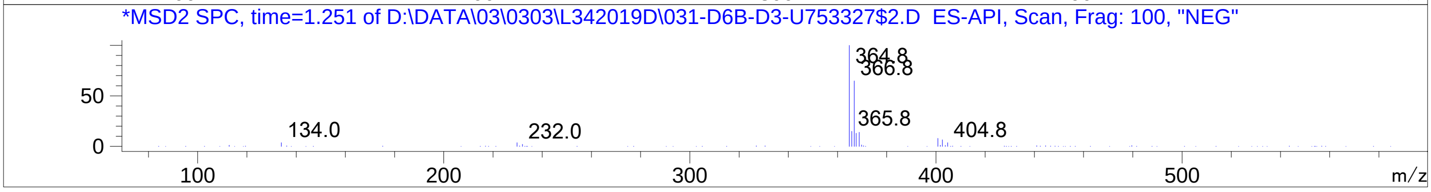

Supplement: Supplementary file 1 — Supplementary Information 1. [file 41598_2024_54655_MOESM1_ESM.zip › Nature SREP/QC_AIDD_cs_selected/LATS1_HID_8_LCMS.pdf]
