# Supplementary material for: AI is a viable alternative to high throughput screening: a 318-target study
Source: Sci Rep. 2024 Apr 2;14:7526. doi: 10.1038/s41598-024-54655-z (PMC10987645; doi:10.1038/s41598-024-54655-z)
Supplement: Supplementary file 1 — Supplementary Information 1. [file 41598_2024_54655_MOESM1_ESM.zip › Nature SREP/QC_AIDD_cs_selected/LATS1_HID_9_LCMS.pdf]

MaxPeak: 100.00%
Ret_Time: 1.359 min

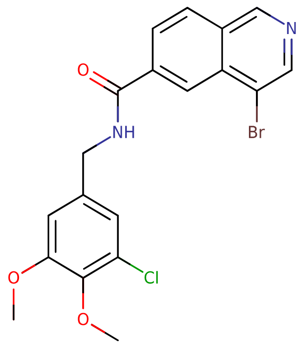

Mol Wt 435.7

Exact Mass 436.02

#	Time	Area%
1	1.359	100.00

U267646$3

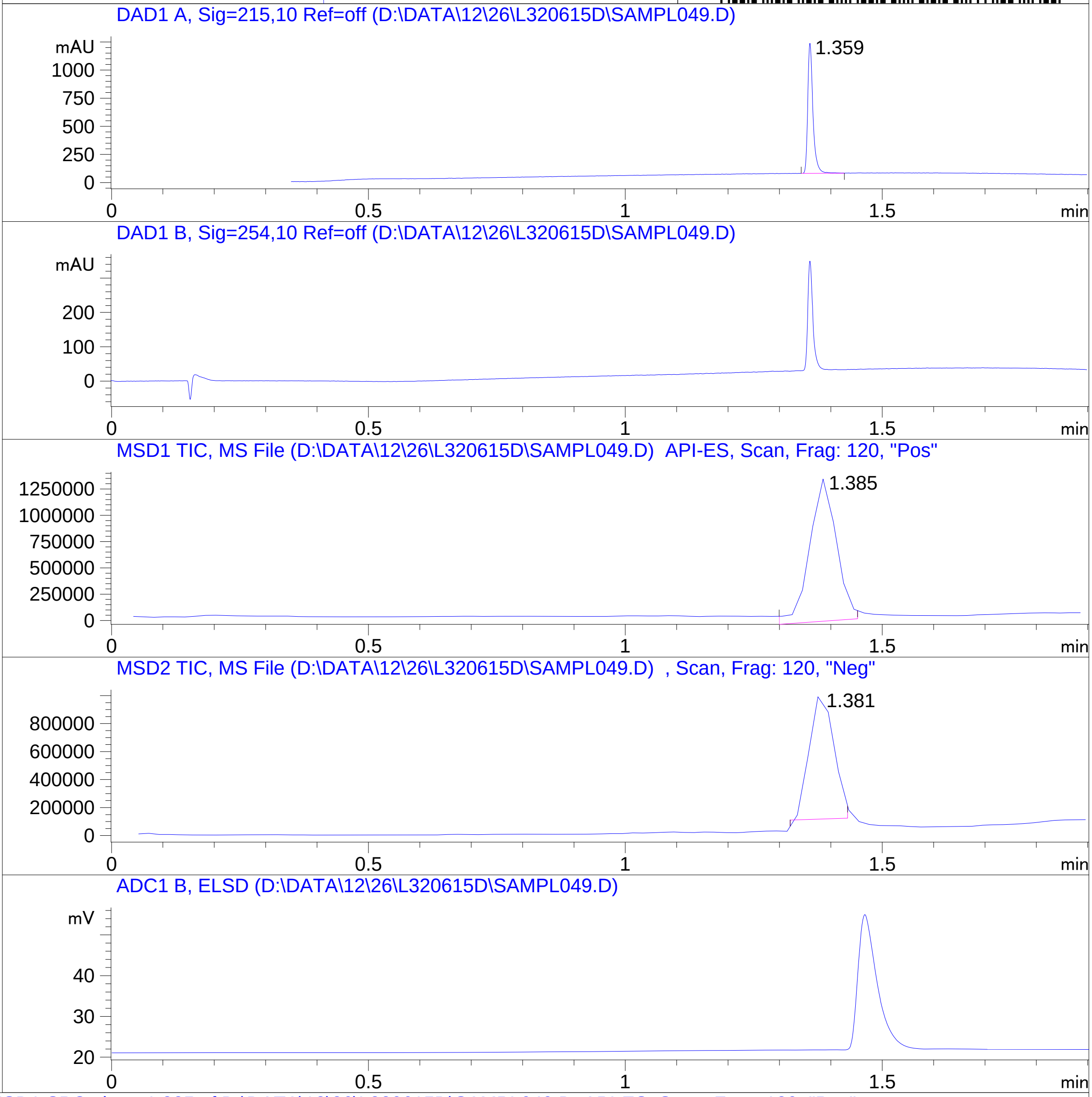

RT 1.385

RT 1.381

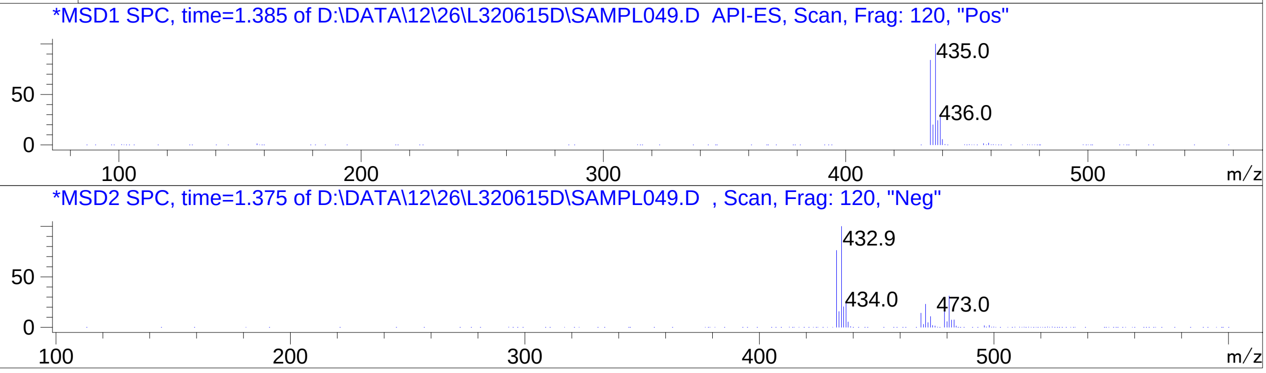

Inj.Date 12/25/2020 LB -SL- Acq. Method C:\HPCHEM\-> ->
