# Supplementary material for: AI is a viable alternative to high throughput screening: a 318-target study
Source: Sci Rep. 2024 Apr 2;14:7526. doi: 10.1038/s41598-024-54655-z (PMC10987645; doi:10.1038/s41598-024-54655-z)
Supplement: Supplementary file 1 — Supplementary Information 1. [file 41598_2024_54655_MOESM1_ESM.zip › Nature SREP/QC_AIDD_cs_selected/LATS1_HVE_2_LCMS.pdf]

W537860$1

MaxPeak: 100.00%
Ret_Time: 0.885 min

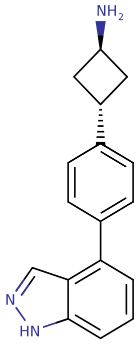

Mol Wt 263.34

Exact Mass 263.17

#	Time	Area%
1	0.885	100.00

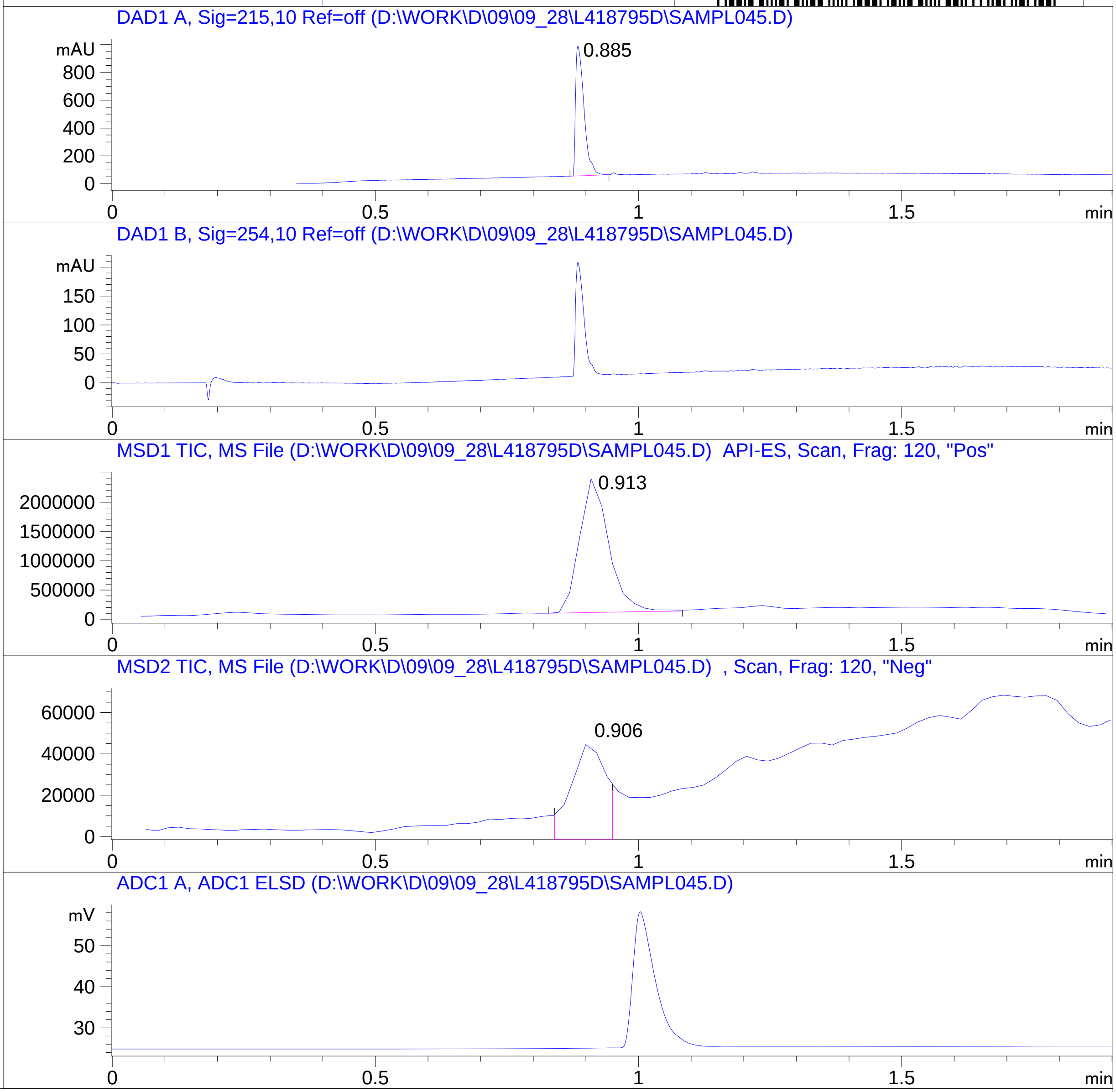

RT 0.913

RT 0.906

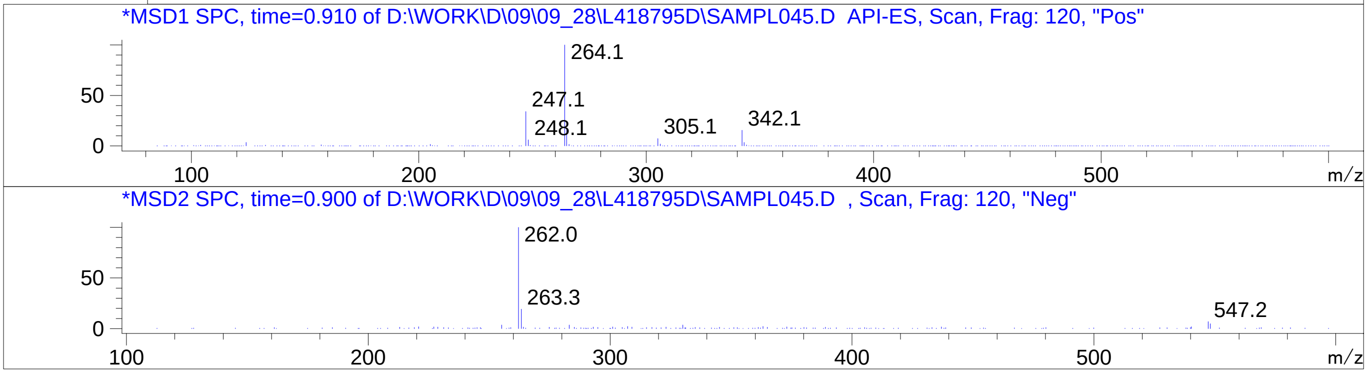

Inj.Date 9/28/2021 K P2-E-09 -VL- Acq. Method C:\HPCHEM\-> ->
