# Supplementary material for: AI is a viable alternative to high throughput screening: a 318-target study
Source: Sci Rep. 2024 Apr 2;14:7526. doi: 10.1038/s41598-024-54655-z (PMC10987645; doi:10.1038/s41598-024-54655-z)
Supplement: Supplementary file 1 — Supplementary Information 1. [file 41598_2024_54655_MOESM1_ESM.zip › Nature SREP/QC_AIDD_cs_selected/LATS1_HVE_3_LCMS.pdf]

# W538579$4

MaxPeak: 96.03%
Ret_Time: 1.163 min

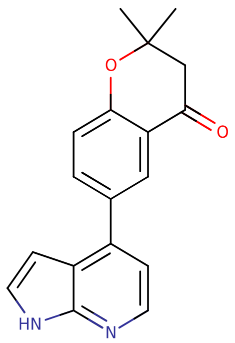


**Mol Wt** 292.33

**Exact Mass** 292.14

| # | Time | Area% |
|---|---|---|
| 1 | 1.163 | 96.03 |
| 2 | 1.205 | 2.12 |
| 3 | 1.321 | 1.85 |

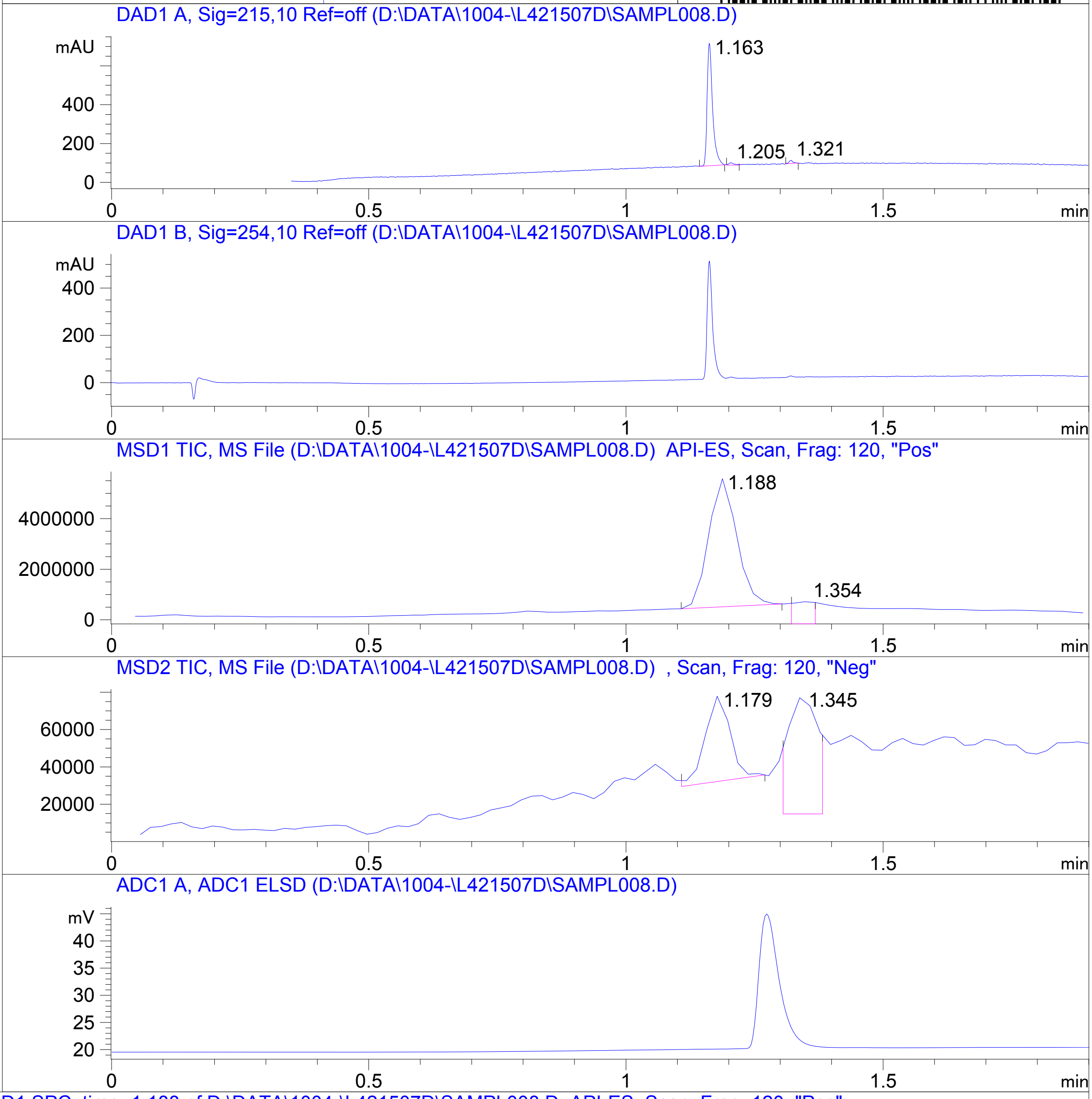


RT 1.188

RT 1.354

RT 1.179

RT 1.345

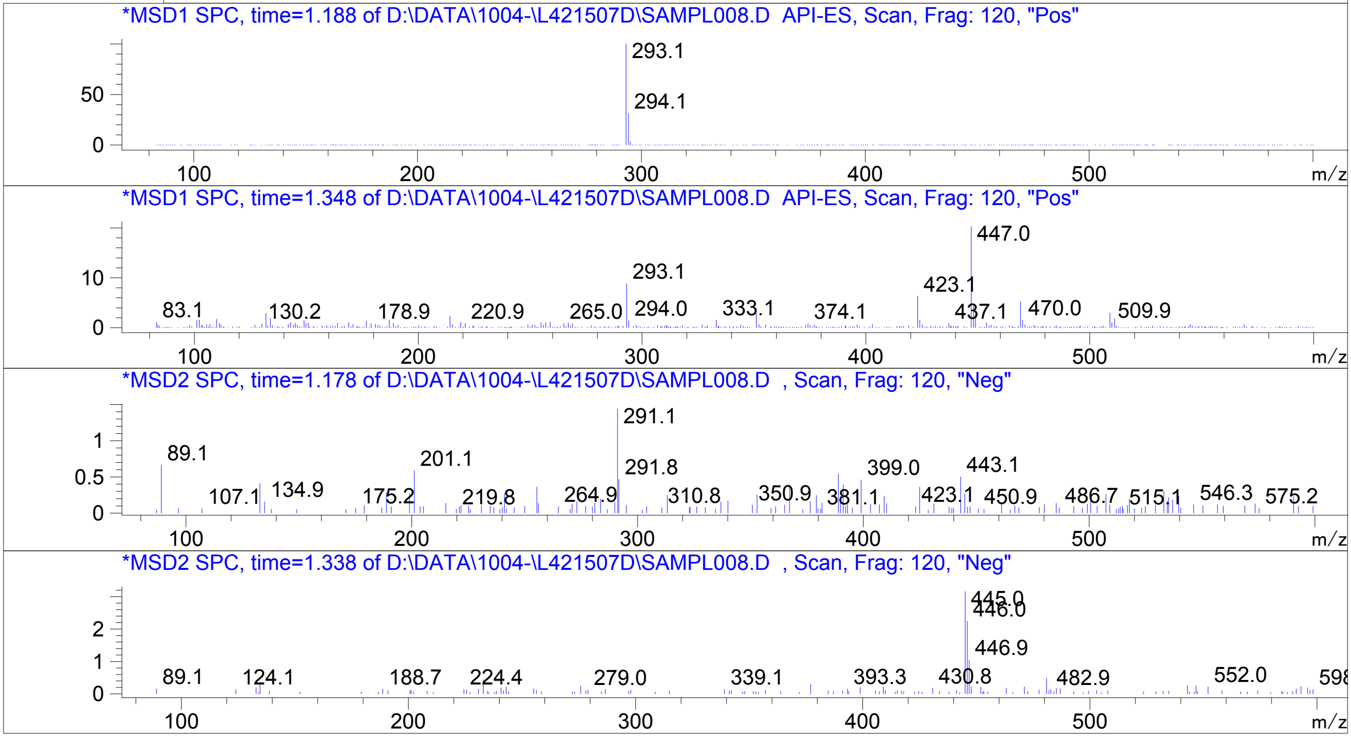


Inj.Date 10/4/2021 OA -SL- Acq. Method C:\HPCHEM\-> ->
