# Supplementary material for: AI is a viable alternative to high throughput screening: a 318-target study
Source: Sci Rep. 2024 Apr 2;14:7526. doi: 10.1038/s41598-024-54655-z (PMC10987645; doi:10.1038/s41598-024-54655-z)
Supplement: Supplementary file 1 — Supplementary Information 1. [file 41598_2024_54655_MOESM1_ESM.zip › Nature SREP/QC_AIDD_cs_selected/LATS1_HVE_4_LCMS.pdf]

W537261$1

MaxPeak: 94.82%
Ret_Time: 1.212 min

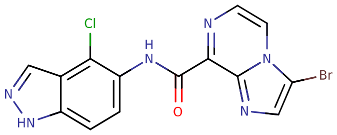

Mol Wt 391.61

Exact Mass 391.96

#	Time	Area%
1	0.791	1.89
2	1.212	94.82
3	1.416	3.29

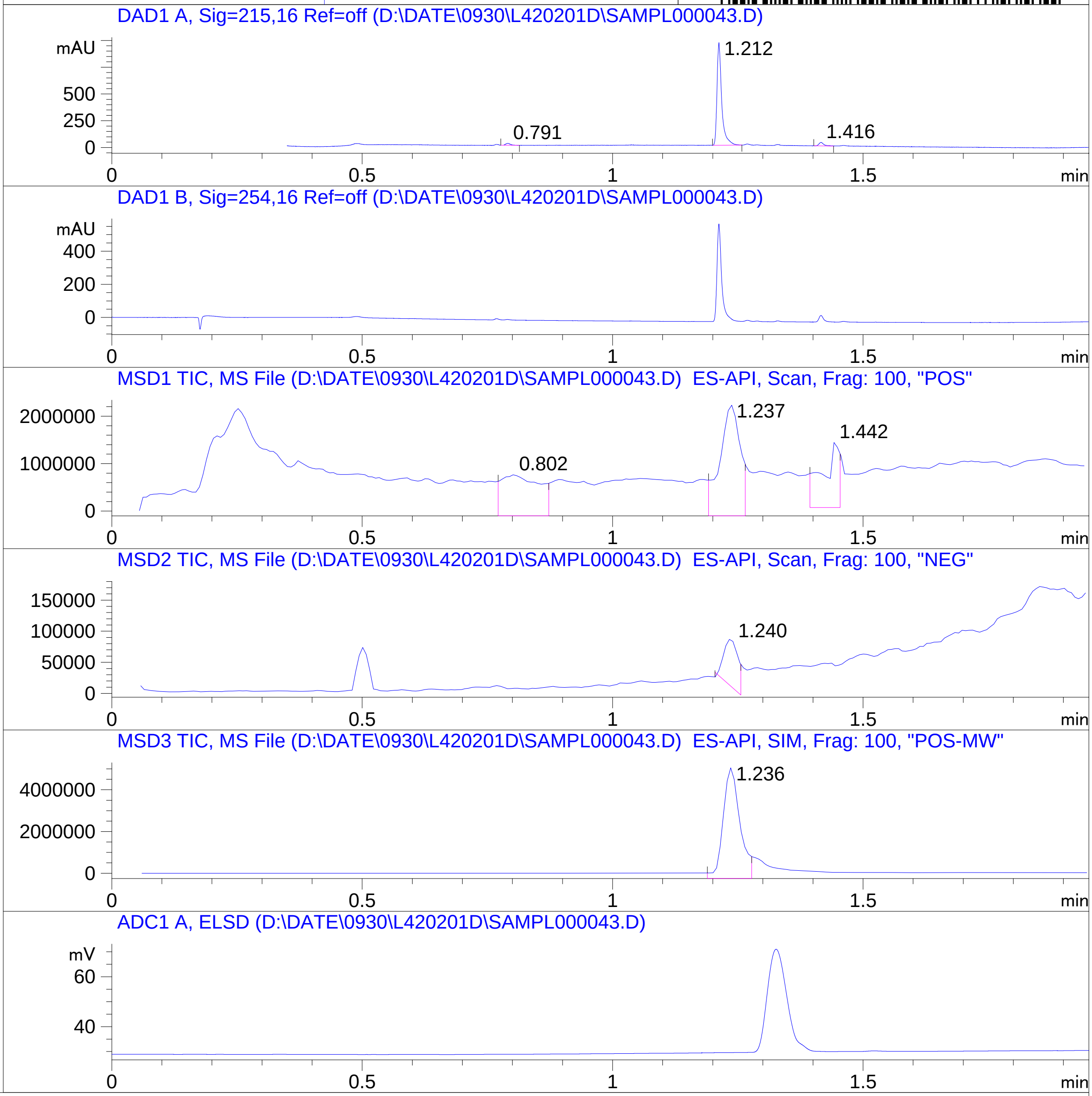

RT 0.802

RT 1.237

RT 1.442

RT 1.240

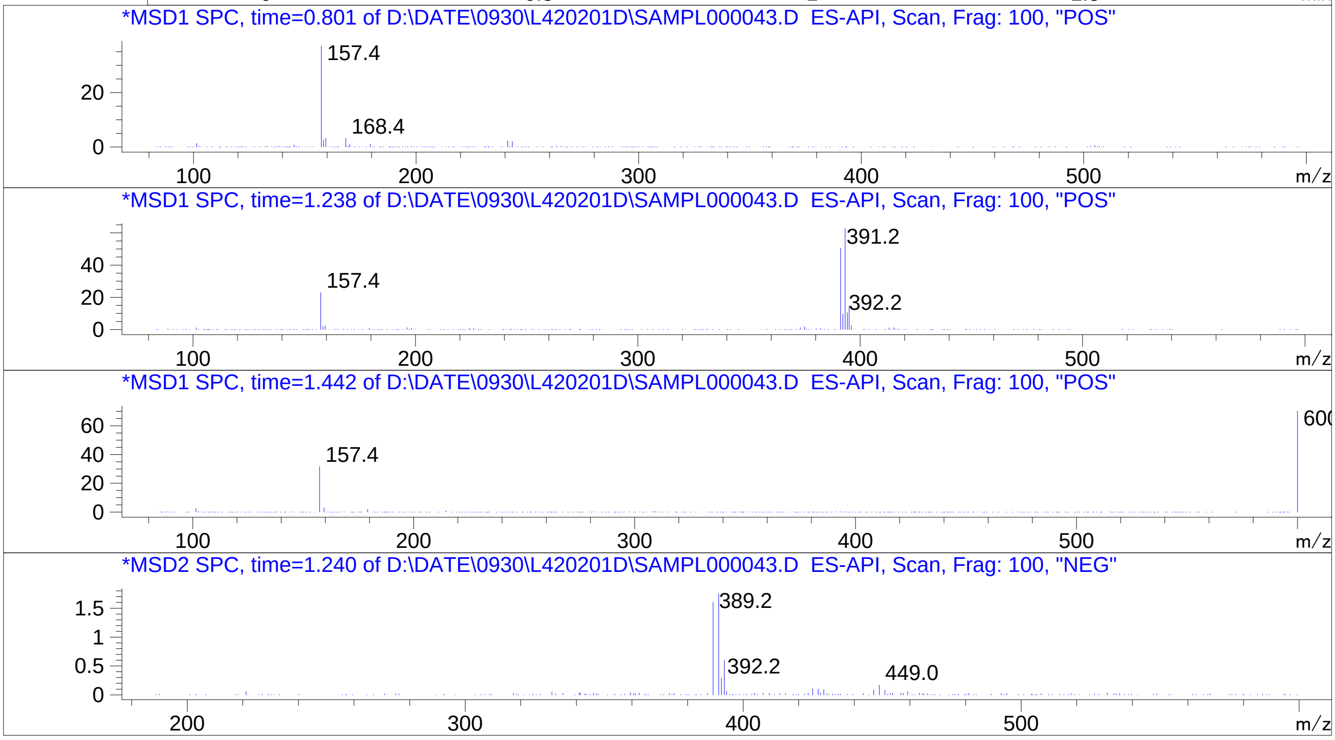

RT 1.236

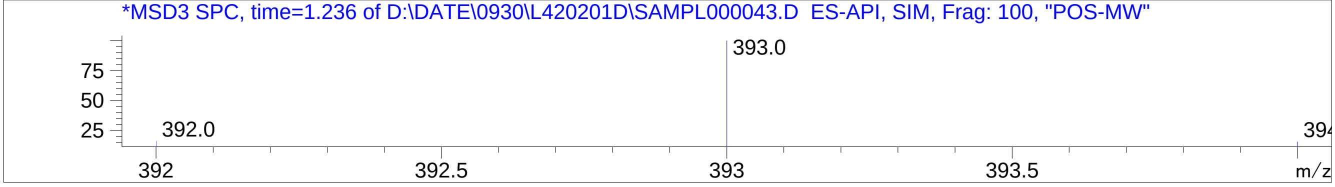
*MSD3 SPC, time=1.236 of D:\DATE\0930\L420201D\SAMPL000043.D ES-API, SIM, Frag: 100, "POS-MW"
393.0
392.0
75
50
25
392
392.5
393
393.5
m/z
