# Supplementary material for: AI is a viable alternative to high throughput screening: a 318-target study
Source: Sci Rep. 2024 Apr 2;14:7526. doi: 10.1038/s41598-024-54655-z (PMC10987645; doi:10.1038/s41598-024-54655-z)

MaxPeak: 96.78%
Ret_Time: 0.974 min

W537700$1

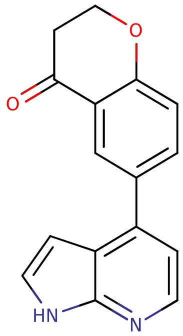

Mol Wt 264.28

Exact Mass 264.1

#	Time	Area%
1	0.974	96.78
2	1.128	3.22

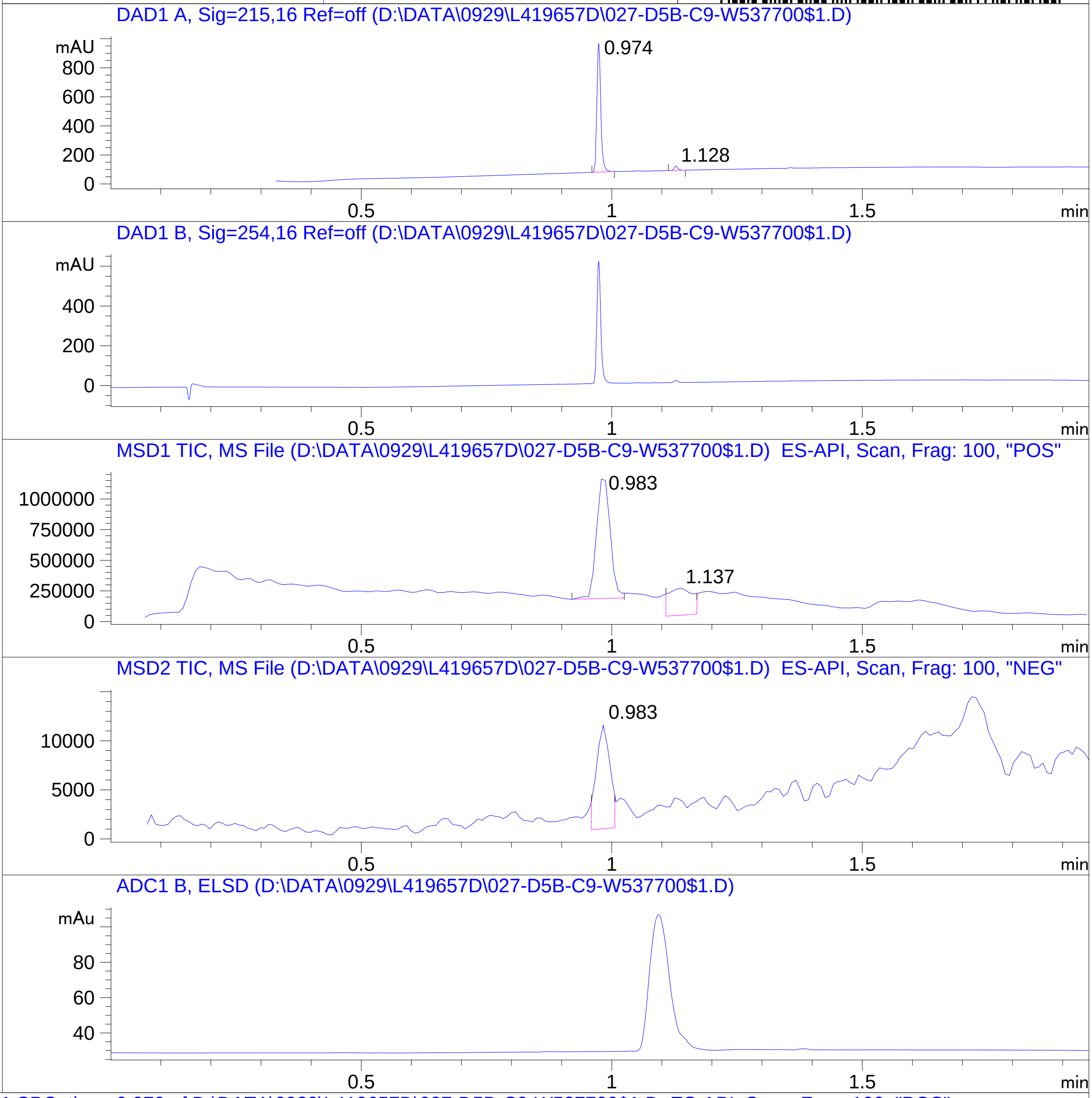

RT 0.983

RT 1.137

RT 0.983

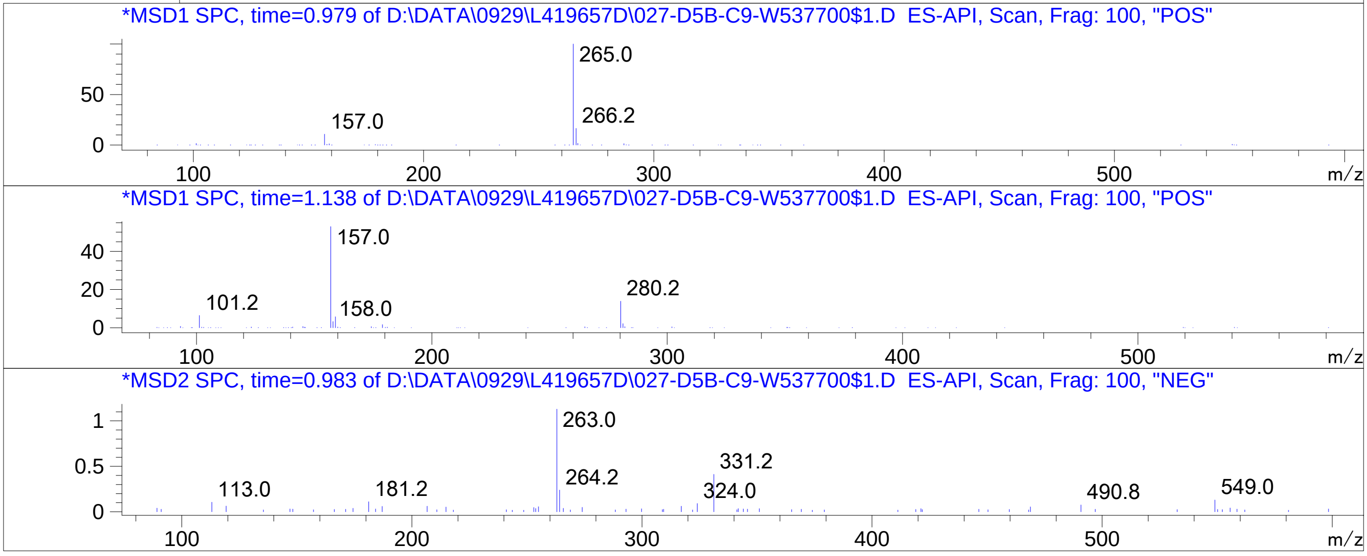

Supplement: Supplementary file 1 — Supplementary Information 1. [file 41598_2024_54655_MOESM1_ESM.zip › Nature SREP/QC_AIDD_cs_selected/LATS1_HVE_5_LCMS.pdf]
