# Supplementary material for: AI is a viable alternative to high throughput screening: a 318-target study
Source: Sci Rep. 2024 Apr 2;14:7526. doi: 10.1038/s41598-024-54655-z (PMC10987645; doi:10.1038/s41598-024-54655-z)
Supplement: Supplementary file 1 — Supplementary Information 1. [file 41598_2024_54655_MOESM1_ESM.zip › Nature SREP/QC_AIDD_cs_selected/LATS1_HVE_6_LCMS.pdf]

MaxPeak: 100.00%
Ret_Time: 0.879 min

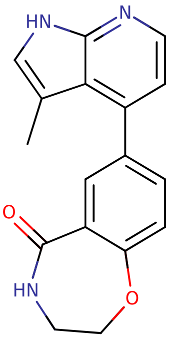

Mol Wt 293.32

Exact Mass 293.13

#	Time	Area%
1	0.879	100.00

U268083$2

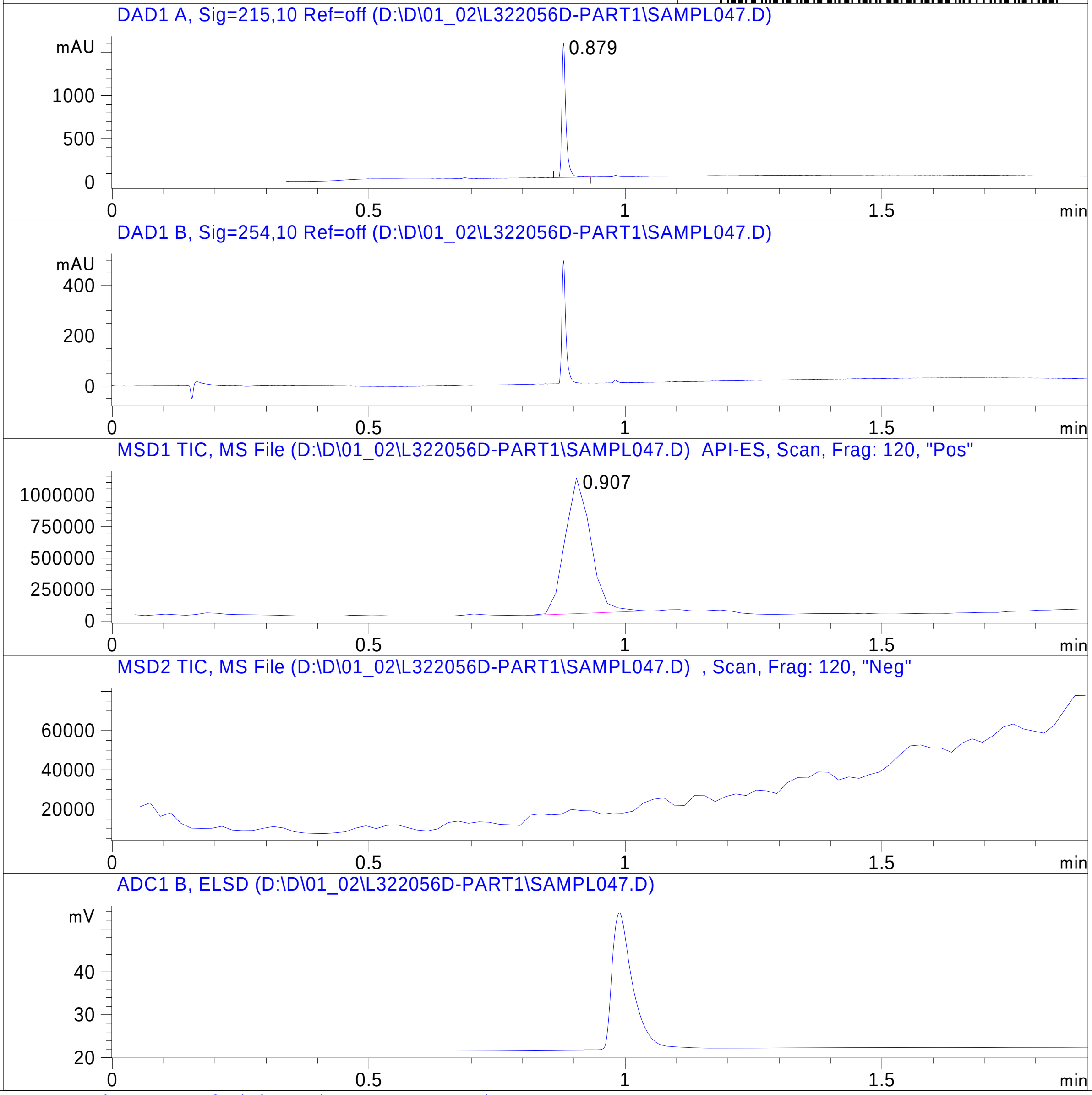

RT 0.907

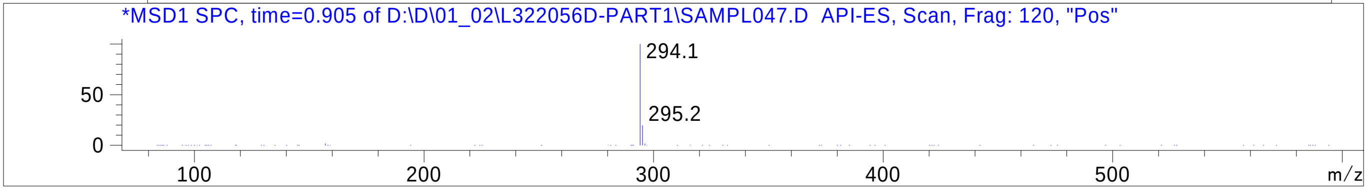

Inj.Date 12/31/2020 N -SL- Acq. Method C:\HPCHEM\-> ->
