# Supplementary material for: AI is a viable alternative to high throughput screening: a 318-target study
Source: Sci Rep. 2024 Apr 2;14:7526. doi: 10.1038/s41598-024-54655-z (PMC10987645; doi:10.1038/s41598-024-54655-z)
Supplement: Supplementary file 1 — Supplementary Information 1. [file 41598_2024_54655_MOESM1_ESM.zip › Nature SREP/QC_AIDD_cs_selected/LATS1_HVE_BEST_4_LCMS.pdf]

W538336$2

MaxPeak: 100.00%
Ret_Time: 0.723 min

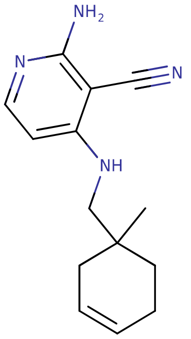

Mol Wt 242.32

Exact Mass 242.18

#	Time	Area%
1	0.723	100.00

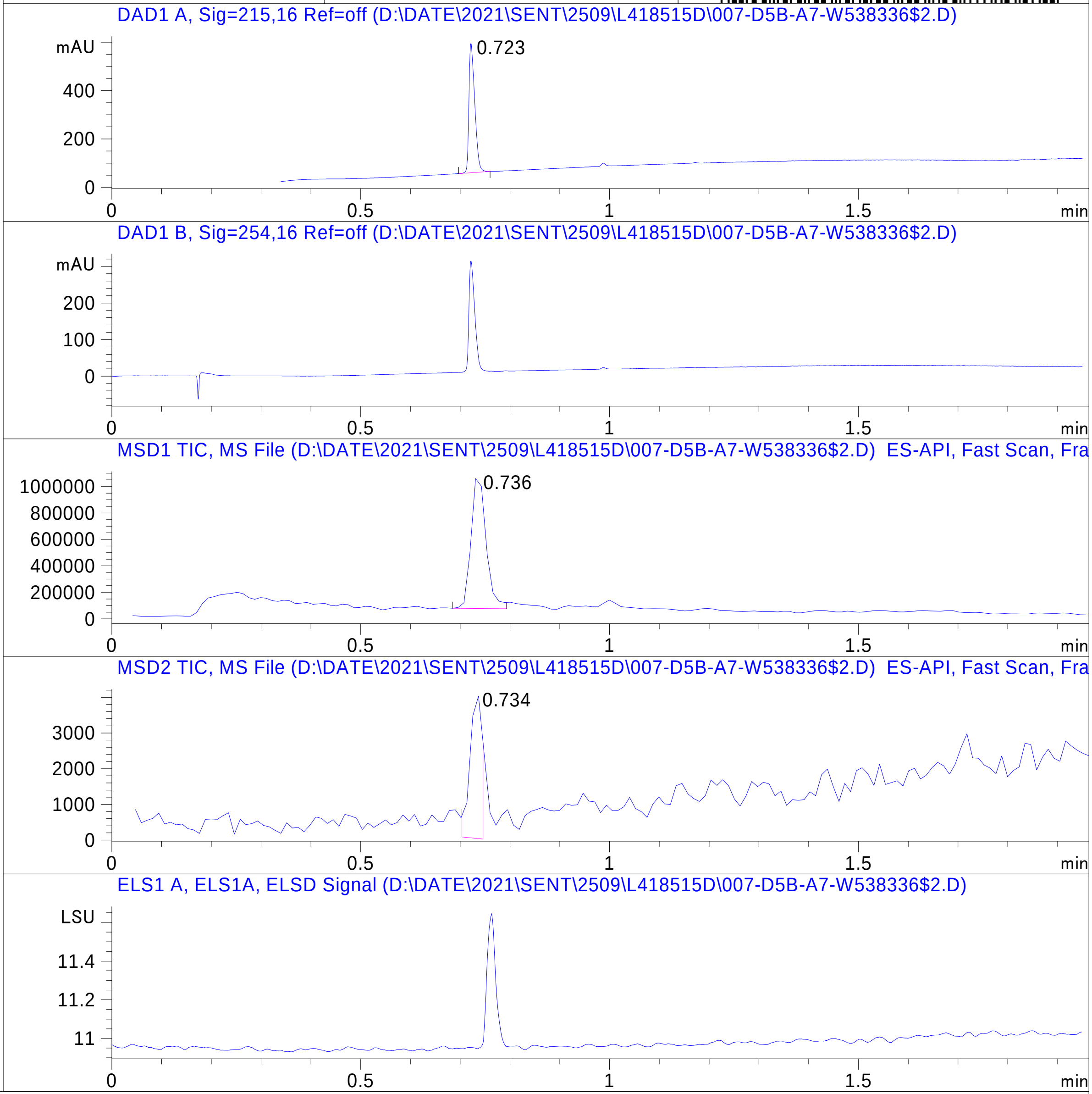

RT 0.736

RT 0.734

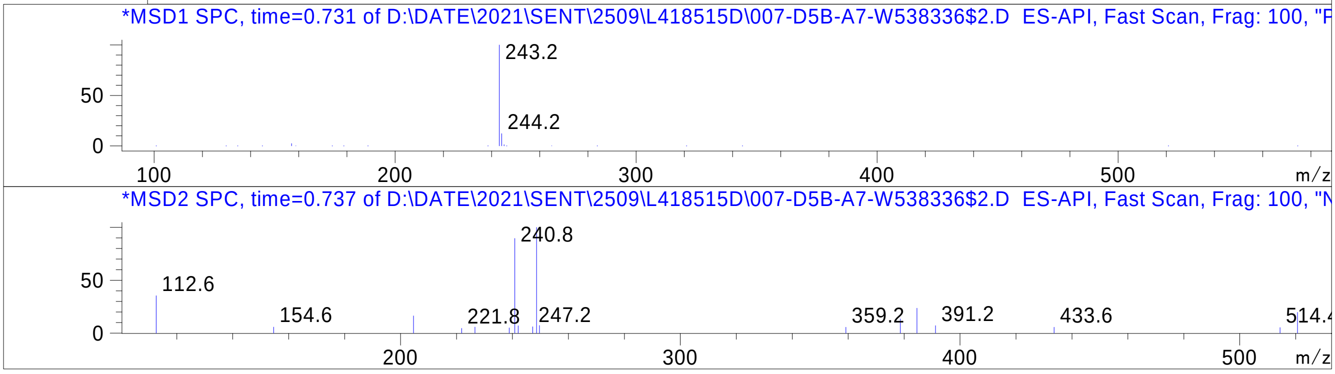

Inj.Date 25-Sep-21 A <invalid> Acq. Method C:\Users\ -> ->
