# Supplementary material for: AI is a viable alternative to high throughput screening: a 318-target study
Source: Sci Rep. 2024 Apr 2;14:7526. doi: 10.1038/s41598-024-54655-z (PMC10987645; doi:10.1038/s41598-024-54655-z)

W537628$1

MaxPeak: 95.69%
Ret_Time: 0.897 min

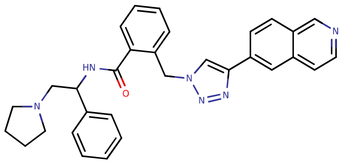

Mol Wt 502.61

Exact Mass 502.29

#	Time	Area%
1	0.897	95.69
2	0.998	1.45
3	1.044	2.86

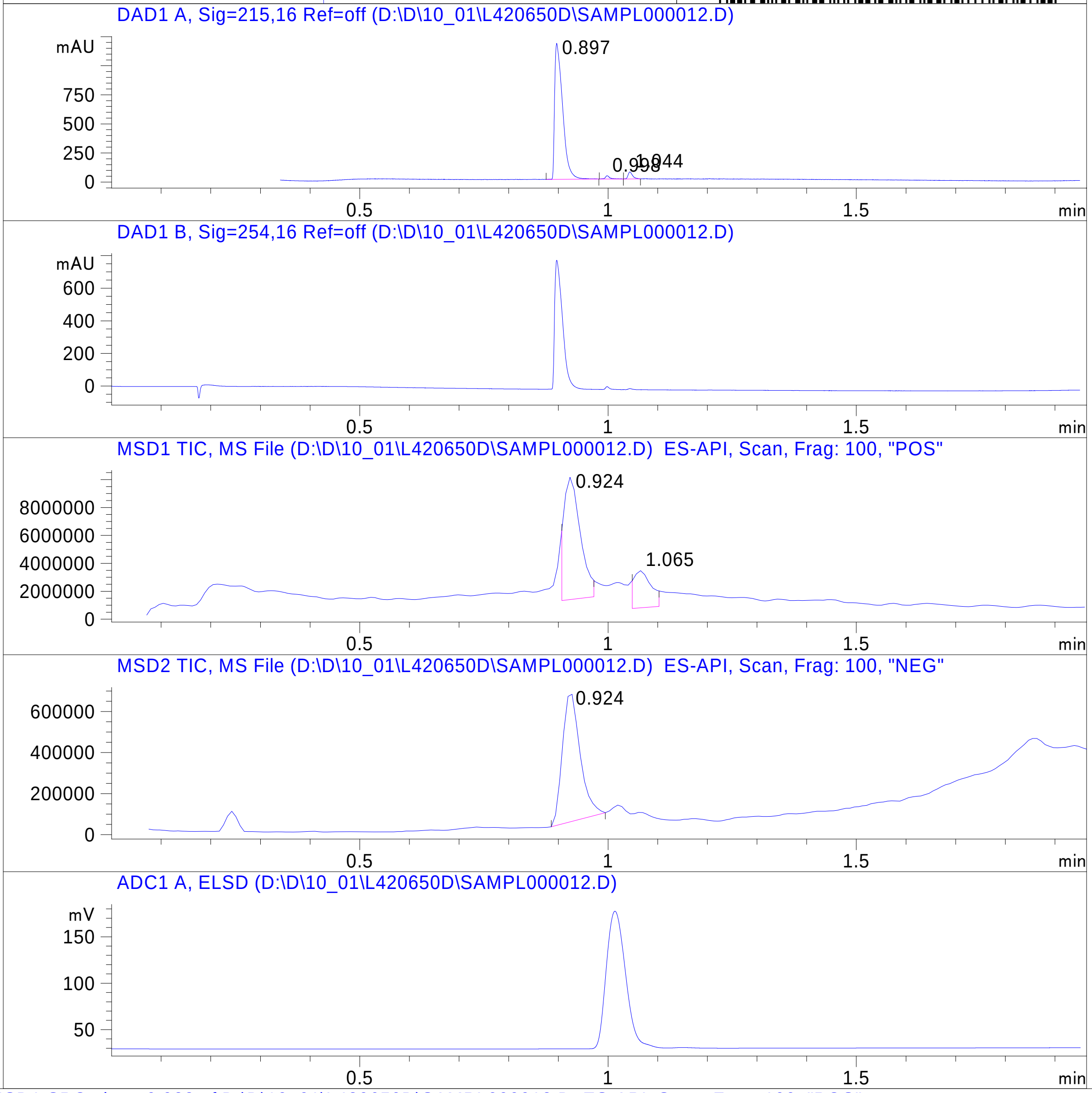

RT 0.924

RT 1.065

RT 0.924

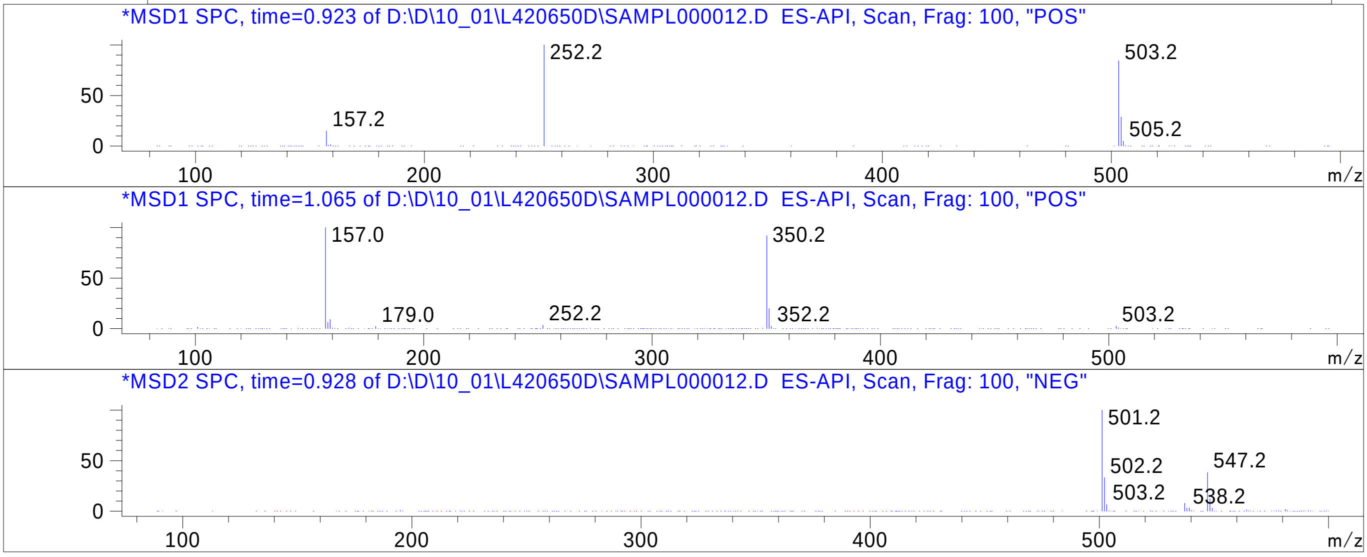

Supplement: Supplementary file 1 — Supplementary Information 1. [file 41598_2024_54655_MOESM1_ESM.zip › Nature SREP/QC_AIDD_cs_selected/LATS1_HVE_BEST_6_LCMS.pdf]
