# Supplementary material for: AI is a viable alternative to high throughput screening: a 318-target study
Source: Sci Rep. 2024 Apr 2;14:7526. doi: 10.1038/s41598-024-54655-z (PMC10987645; doi:10.1038/s41598-024-54655-z)
Supplement: Supplementary file 1 — Supplementary Information 1. [file 41598_2024_54655_MOESM1_ESM.zip › Nature SREP/QC_AIDD_cs_selected/LATS1_HVE_BEST_7_LCMS.pdf]

W537250$1

MaxPeak: 93.21%
Ret_Time: 0.522 min

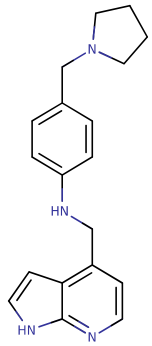

Mol Wt 306.4

Exact Mass 306.22

#	Time	Area%
1	0.522	93.21
2	0.549	3.25
3	0.611	1.23
4	0.897	2.31

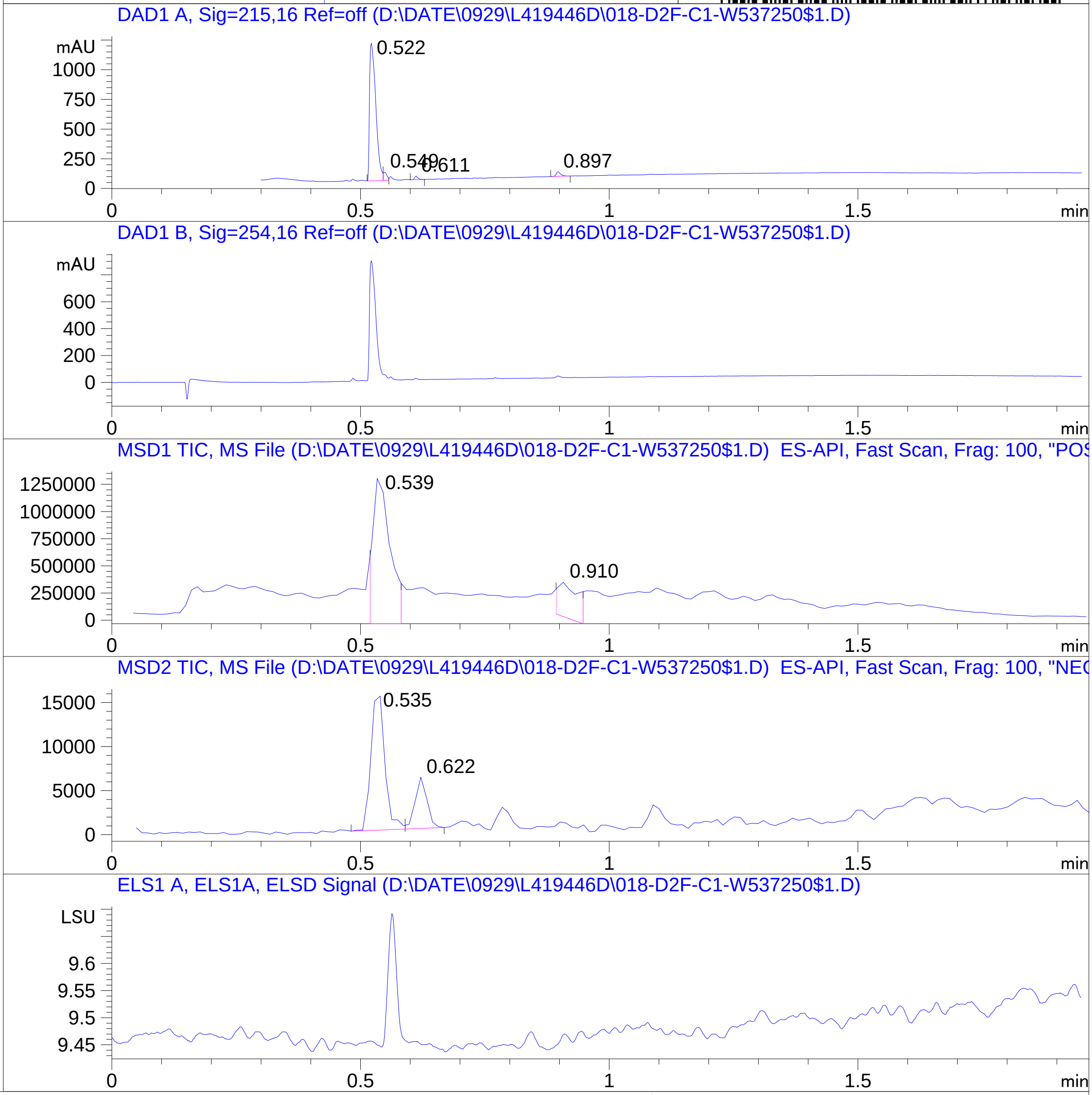

RT 0.539

RT 0.910

RT 0.535

RT 0.622

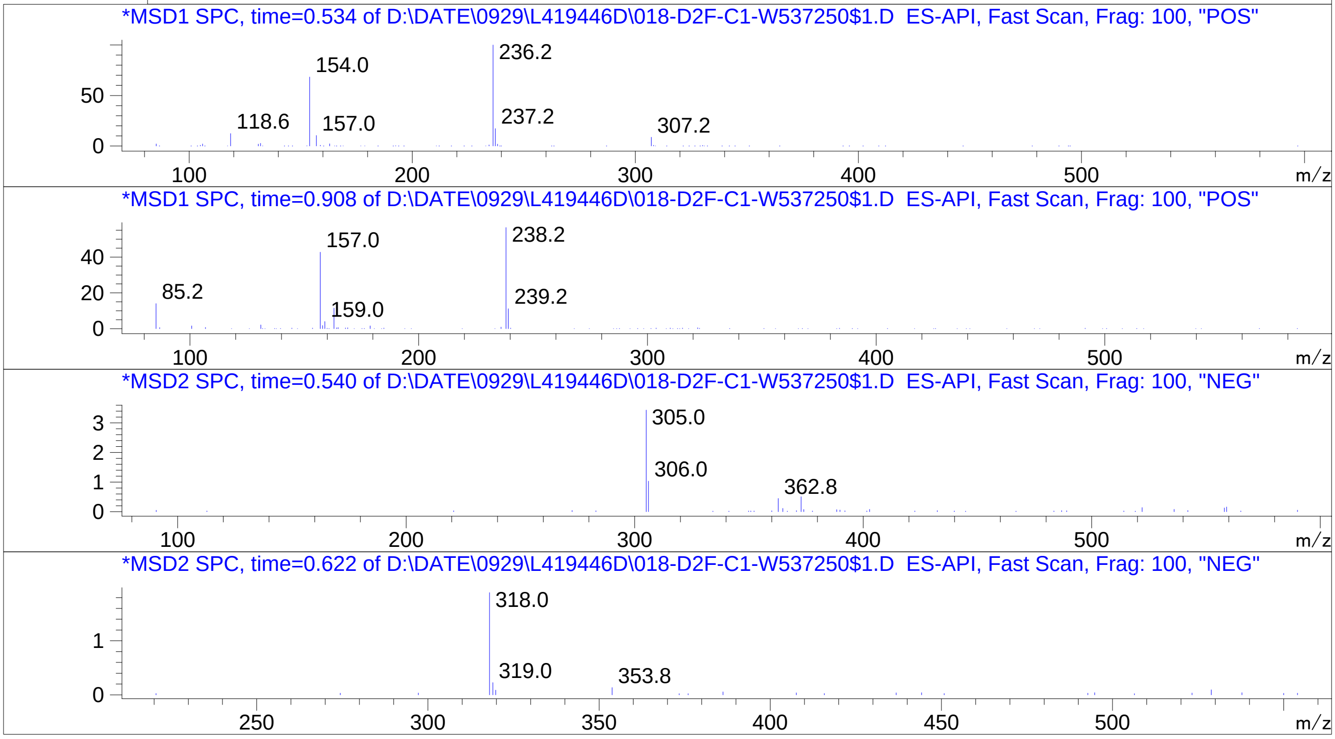

Inj.Date 9/29/2021 E Acq. Method C:\Users\ -> ->
