# Supplementary material for: AI is a viable alternative to high throughput screening: a 318-target study
Source: Sci Rep. 2024 Apr 2;14:7526. doi: 10.1038/s41598-024-54655-z (PMC10987645; doi:10.1038/s41598-024-54655-z)
Supplement: Supplementary file 1 — Supplementary Information 1. [file 41598_2024_54655_MOESM1_ESM.zip › Nature SREP/QC_AIDD_cs_selected/LATS1_HVE_PARENT_5_LCMS.pdf]

U753314$3

MaxPeak: 97.48%
Ret_Time: 0.890 min

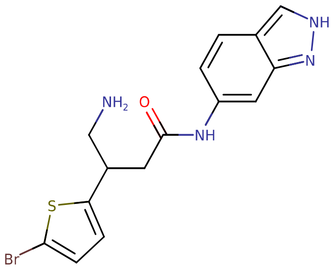

Mol Wt 379.28

Exact Mass 380.03

#	Time	Area%
1	0.890	97.48
2	1.135	2.52

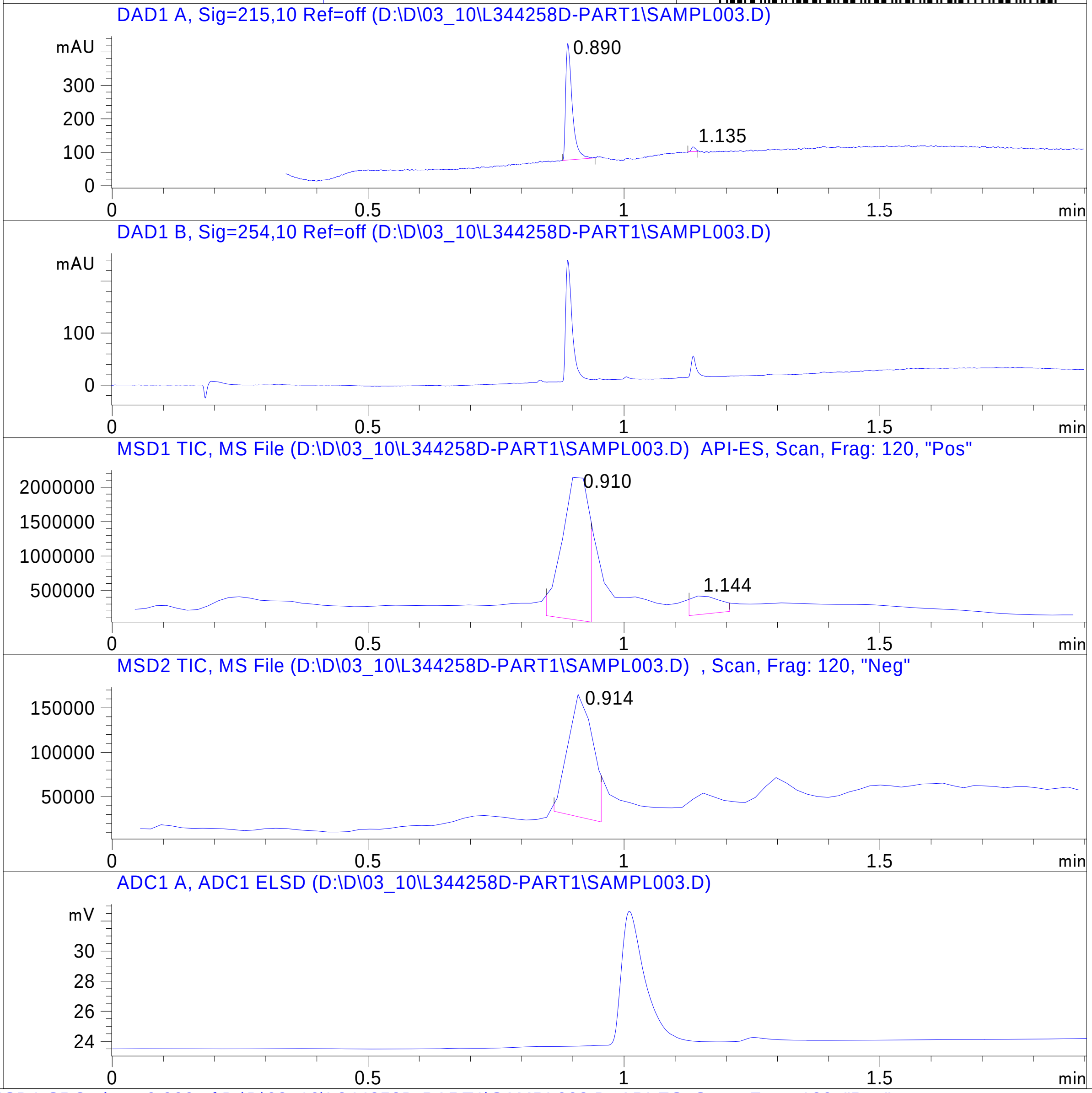

RT 0.910

RT 1.144

RT 0.914

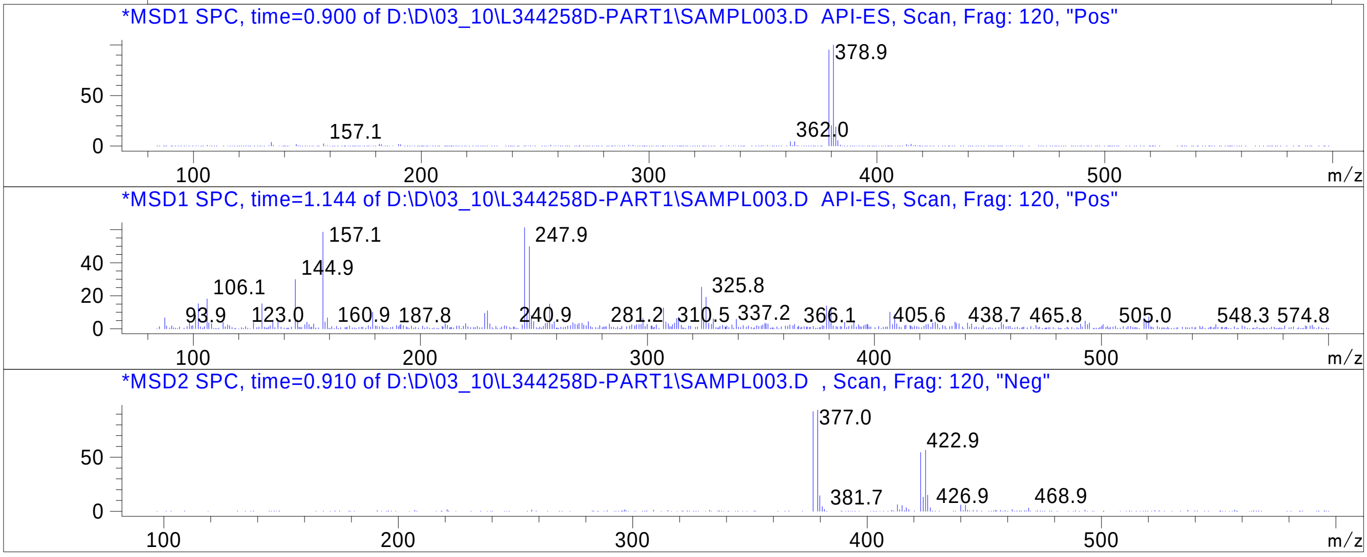

Inj.Date 3/10/2021 N -VL- Acq. Method C:\HPCHEM\-> ->
