# Supplementary material for: AI is a viable alternative to high throughput screening: a 318-target study
Source: Sci Rep. 2024 Apr 2;14:7526. doi: 10.1038/s41598-024-54655-z (PMC10987645; doi:10.1038/s41598-024-54655-z)
Supplement: Supplementary file 1 — Supplementary Information 1. [file 41598_2024_54655_MOESM1_ESM.zip › Nature SREP/QC_AIDD_cs_selected/LATS1_HVE_PARENT_6_LCMS.pdf]

MaxPeak: 100.00%
Ret_Time: 0.708 min

W946903$916

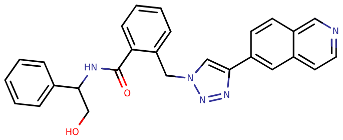

Mol Wt 449.5

Exact Mass 449.21

#	Time	Area%
1	0.708	100.00

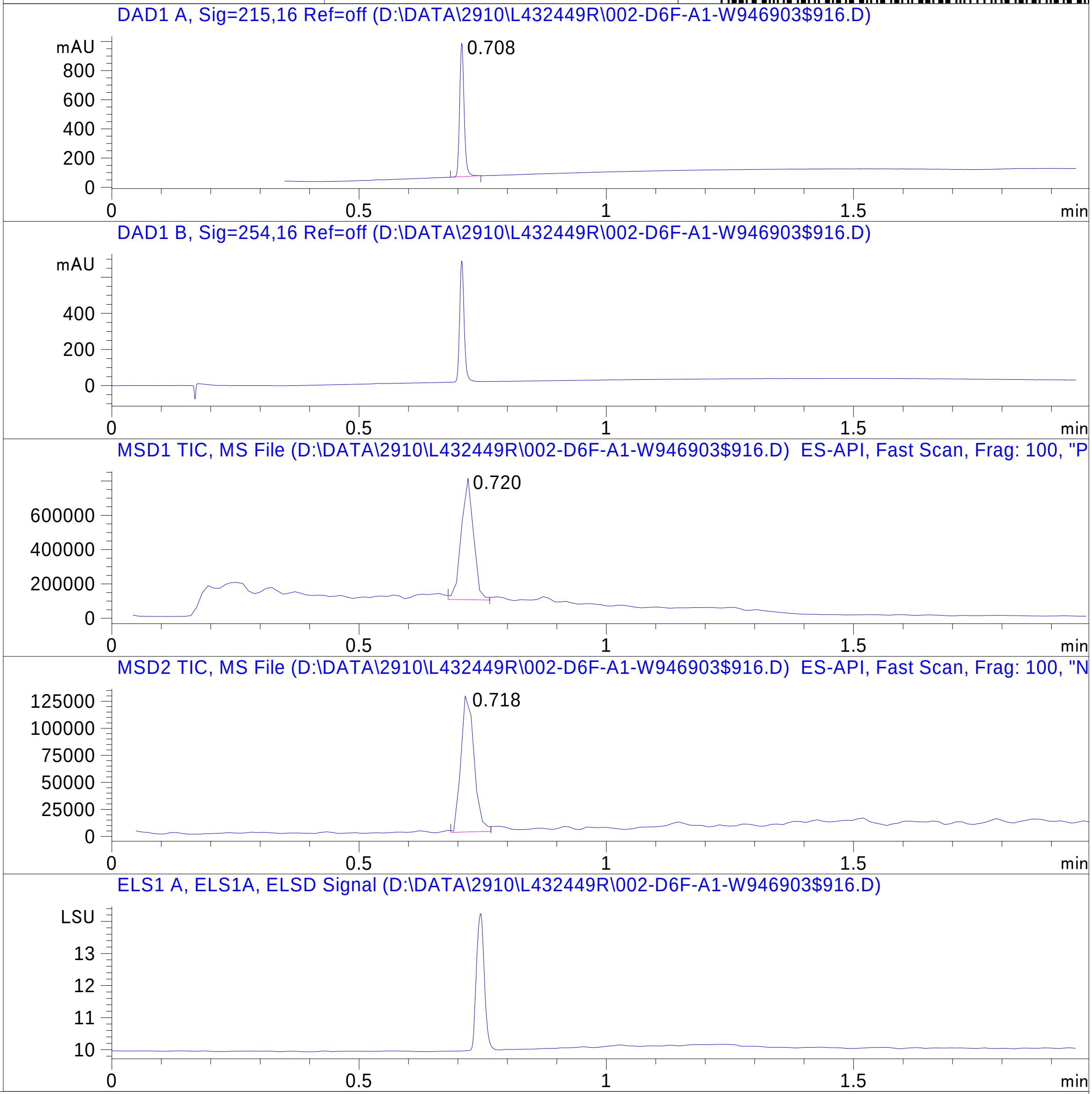

RT 0.720

RT 0.718

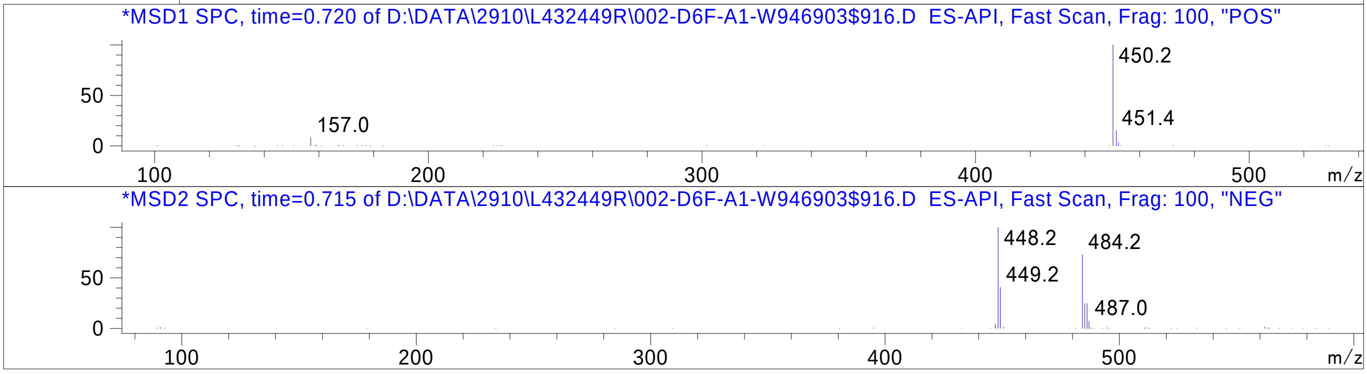

Inj.Date 10/28/2021 H 22 Acq. Method C:\Users\ -> ->
