# Supplementary material for: AI is a viable alternative to high throughput screening: a 318-target study
Source: Sci Rep. 2024 Apr 2;14:7526. doi: 10.1038/s41598-024-54655-z (PMC10987645; doi:10.1038/s41598-024-54655-z)
Supplement: Supplementary file 1 — Supplementary Information 1. [file 41598_2024_54655_MOESM1_ESM.zip › Nature SREP/QC_AIDD_cs_selected/LATS1_HVE_PARENT_7_LCMS.pdf]

MaxPeak: 95.05%
Ret_Time: 0.634 min

U267852$5

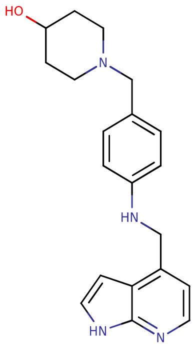

Mol Wt 336.43

Exact Mass 336.23

#	Time	Area%
1	0.634	95.05
2	0.821	3.04
3	1.224	1.90

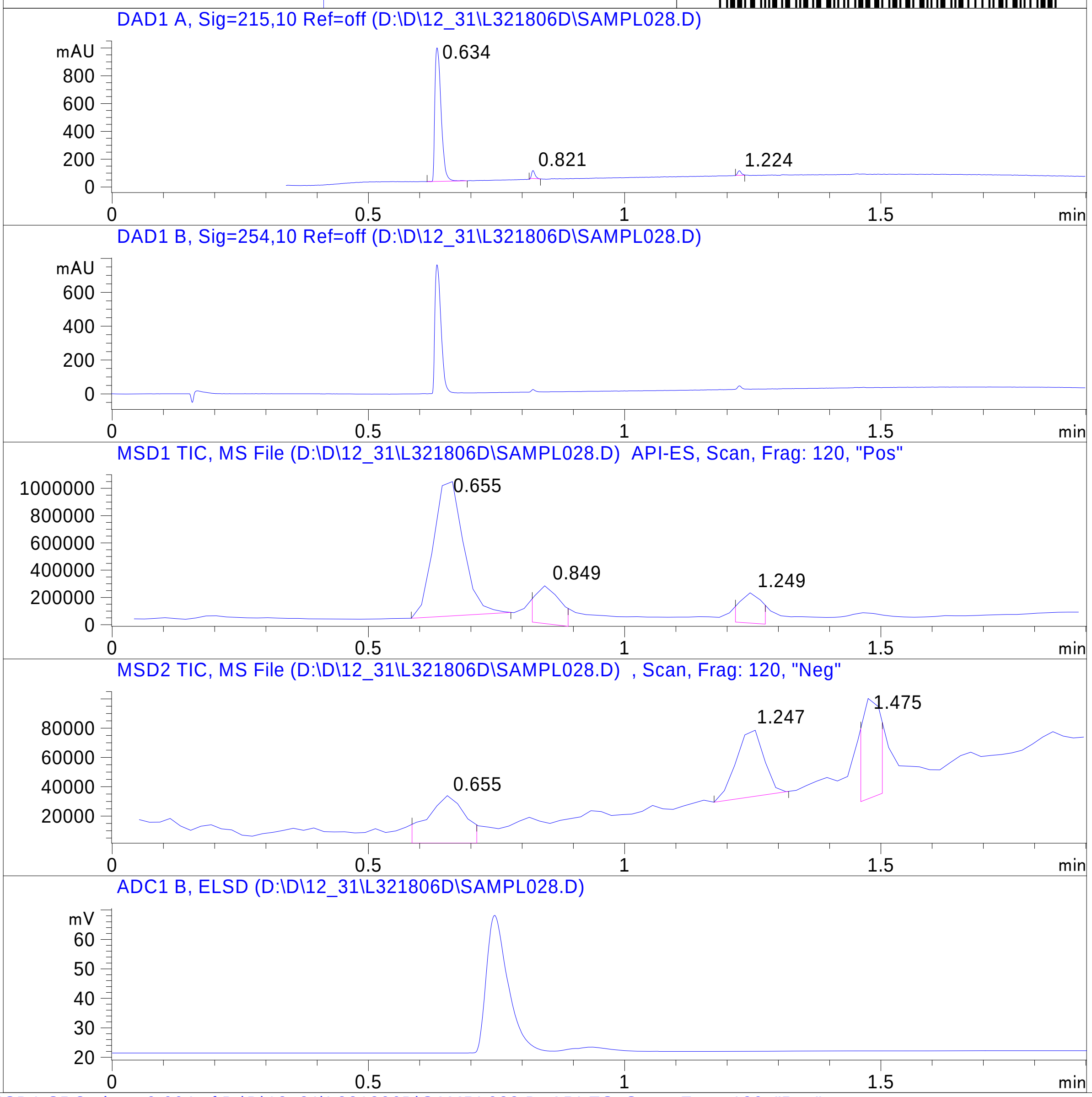

RT 0.655

RT 0.849

RT 1.249

RT 0.655

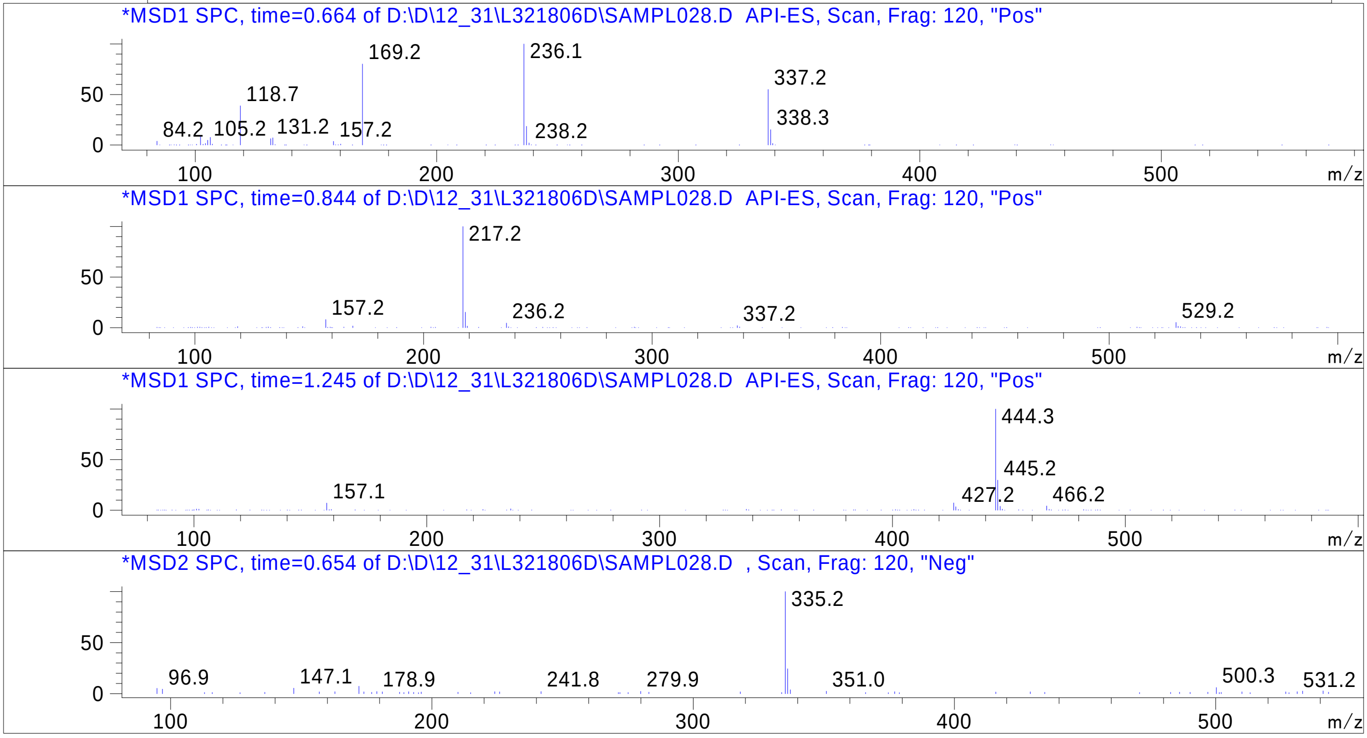

Inj.Date 12/31/2020 N -SL- Acq. Method C:\HPCHEM\-> ->

RT 1.247

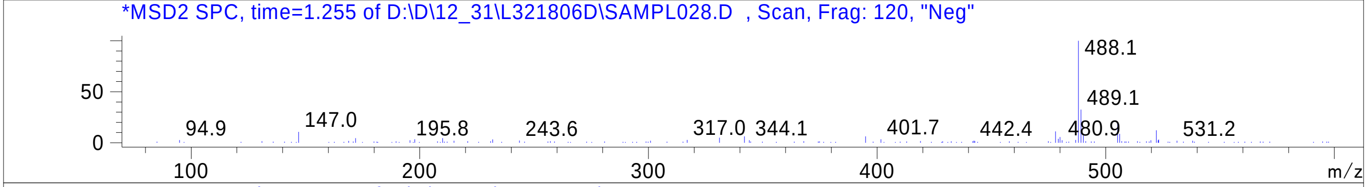

RT 1.475

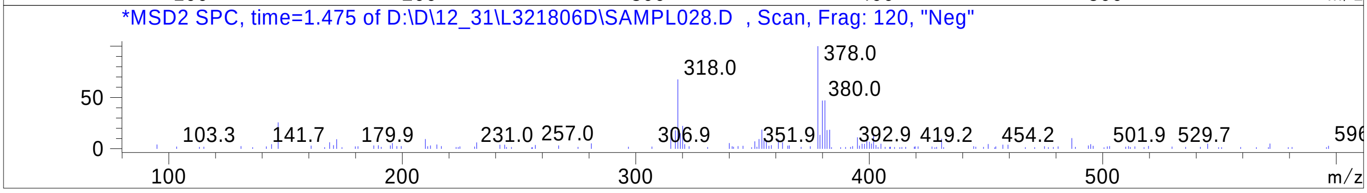

Inj.Date 12/31/2020 N -SL- Acq. Method C:\HPCHEM\-> ->
