# Supplementary material for: AI is a viable alternative to high throughput screening: a 318-target study
Source: Sci Rep. 2024 Apr 2;14:7526. doi: 10.1038/s41598-024-54655-z (PMC10987645; doi:10.1038/s41598-024-54655-z)
Supplement: Supplementary file 1 — Supplementary Information 1. [file 41598_2024_54655_MOESM1_ESM.zip › Nature SREP/QC_AIDD_cs_selected/LATS1_HVE_PARENT_8_LCMS.pdf]

U267659$23

MaxPeak: 100.00%
Ret_Time: 1.064 min

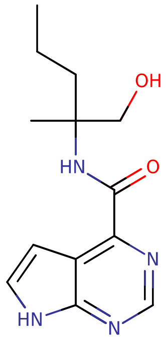

Mol Wt 262.31

Exact Mass 262.16

#	Time	Area%
1	1.064	100.00

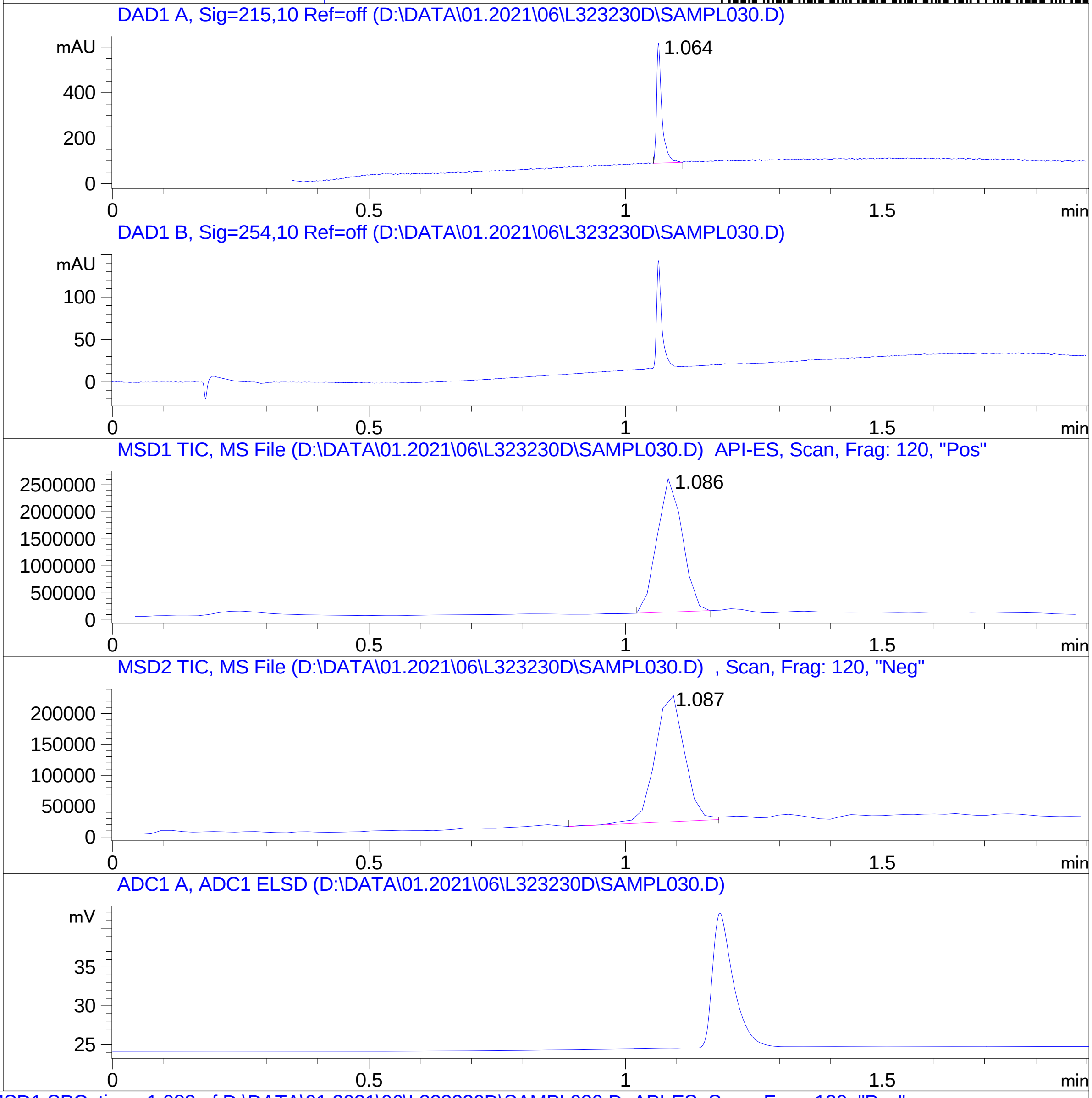

RT 1.086

RT 1.087

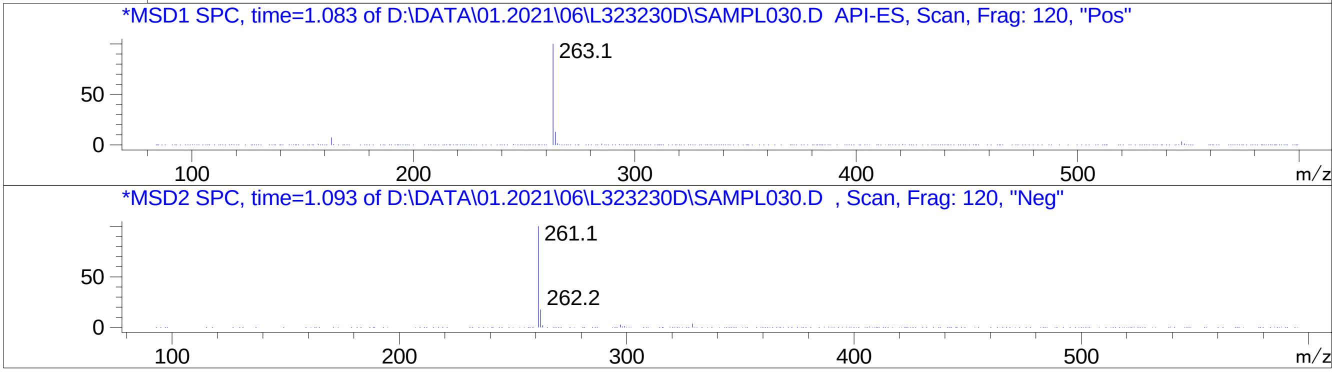

Inj.Date 1/6/2021 CH P2-D-07 -VL- Acq. Method C:\HPCHEM\-> ->
