# Supplementary material for: AI is a viable alternative to high throughput screening: a 318-target study
Source: Sci Rep. 2024 Apr 2;14:7526. doi: 10.1038/s41598-024-54655-z (PMC10987645; doi:10.1038/s41598-024-54655-z)

V026274$4

MaxPeak: 100.00%
Ret_Time: 0.933 min

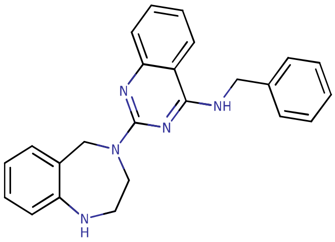

Mol Wt 381.47

Exact Mass 381.23

#	Time	Area%
1	0.933	100.00

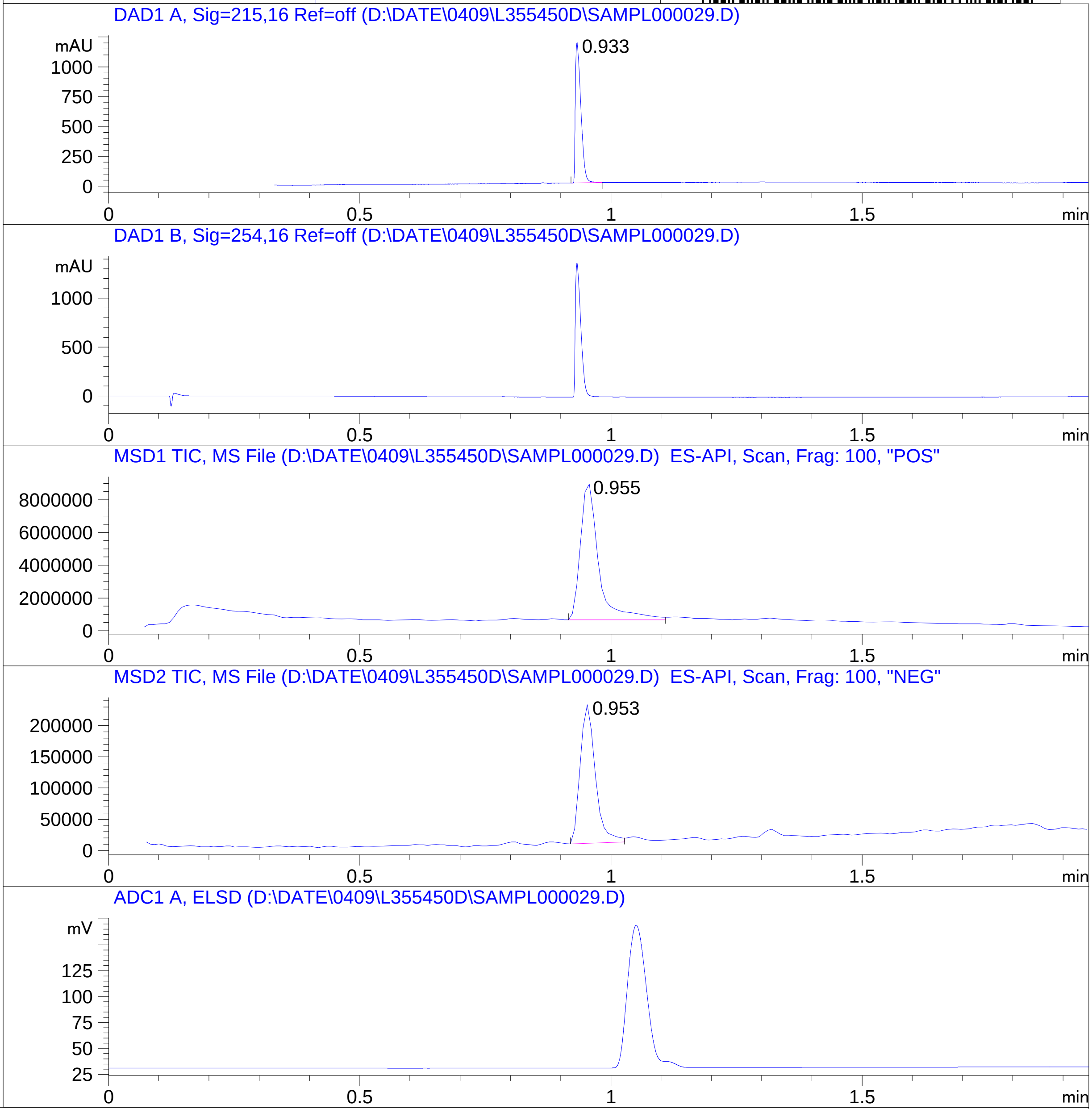

RT 0.955

RT 0.953

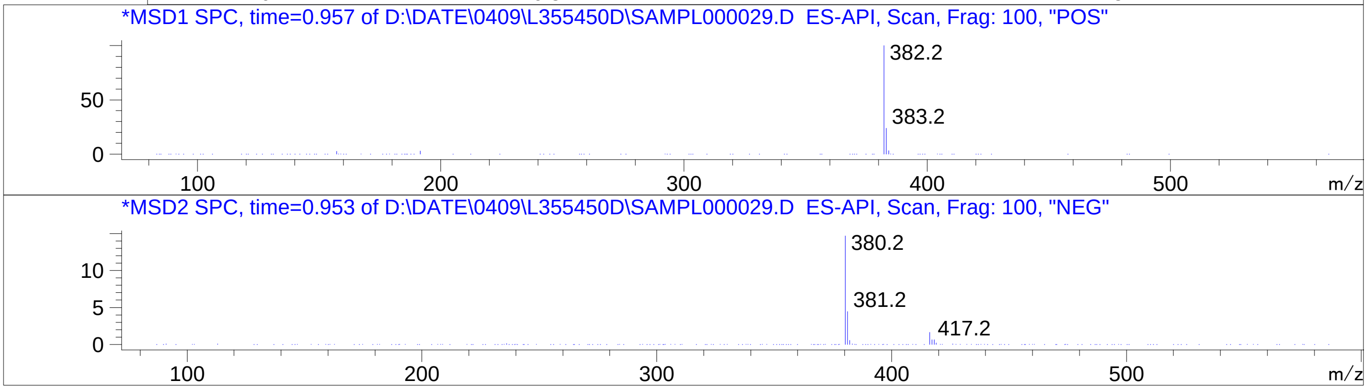

Supplement: Supplementary file 1 — Supplementary Information 1. [file 41598_2024_54655_MOESM1_ESM.zip › Nature SREP/QC_AIDD_cs_selected/VCP_HID_1_LCMS.pdf]
