# Supplementary material for: AI is a viable alternative to high throughput screening: a 318-target study
Source: Sci Rep. 2024 Apr 2;14:7526. doi: 10.1038/s41598-024-54655-z (PMC10987645; doi:10.1038/s41598-024-54655-z)
Supplement: Supplementary file 1 — Supplementary Information 1. [file 41598_2024_54655_MOESM1_ESM.zip › Nature SREP/QC_AIDD_cs_selected/VCP_HID_2_LCMS.pdf]

MaxPeak: 100.00%
Ret_Time: 1.499 min

V026594$3

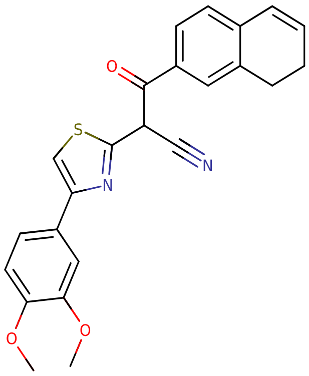

Mol Wt 416.49

Exact Mass 416.14

#	Time	Area%
1	1.499	100.00

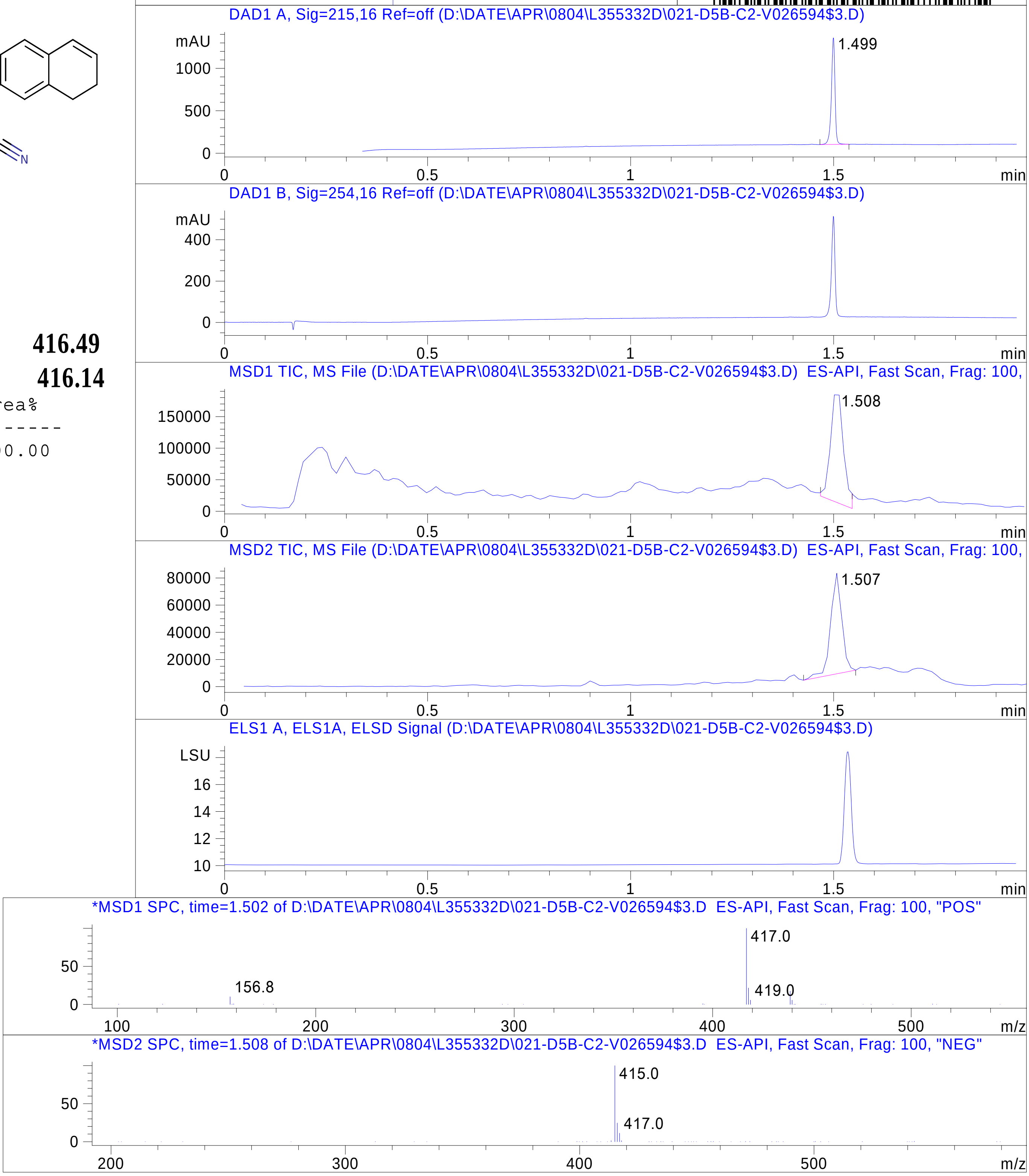

RT 1.508

RT 1.507

Inj.Date 4/8/2021 Y Acq. Method C:\Users\ -> ->
