# Supplementary material for: AI is a viable alternative to high throughput screening: a 318-target study
Source: Sci Rep. 2024 Apr 2;14:7526. doi: 10.1038/s41598-024-54655-z (PMC10987645; doi:10.1038/s41598-024-54655-z)
Supplement: Supplementary file 1 — Supplementary Information 1. [file 41598_2024_54655_MOESM1_ESM.zip › Nature SREP/QC_AIDD_cs_selected/VCP_HID_3_LCMS.pdf]

MaxPeak: 100.00%
Ret_Time: 1.483 min

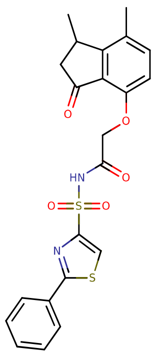

Mol Wt 456.54

Exact Mass 456.09

#	Time	Area%
1	1.483	100.00

V026625$4

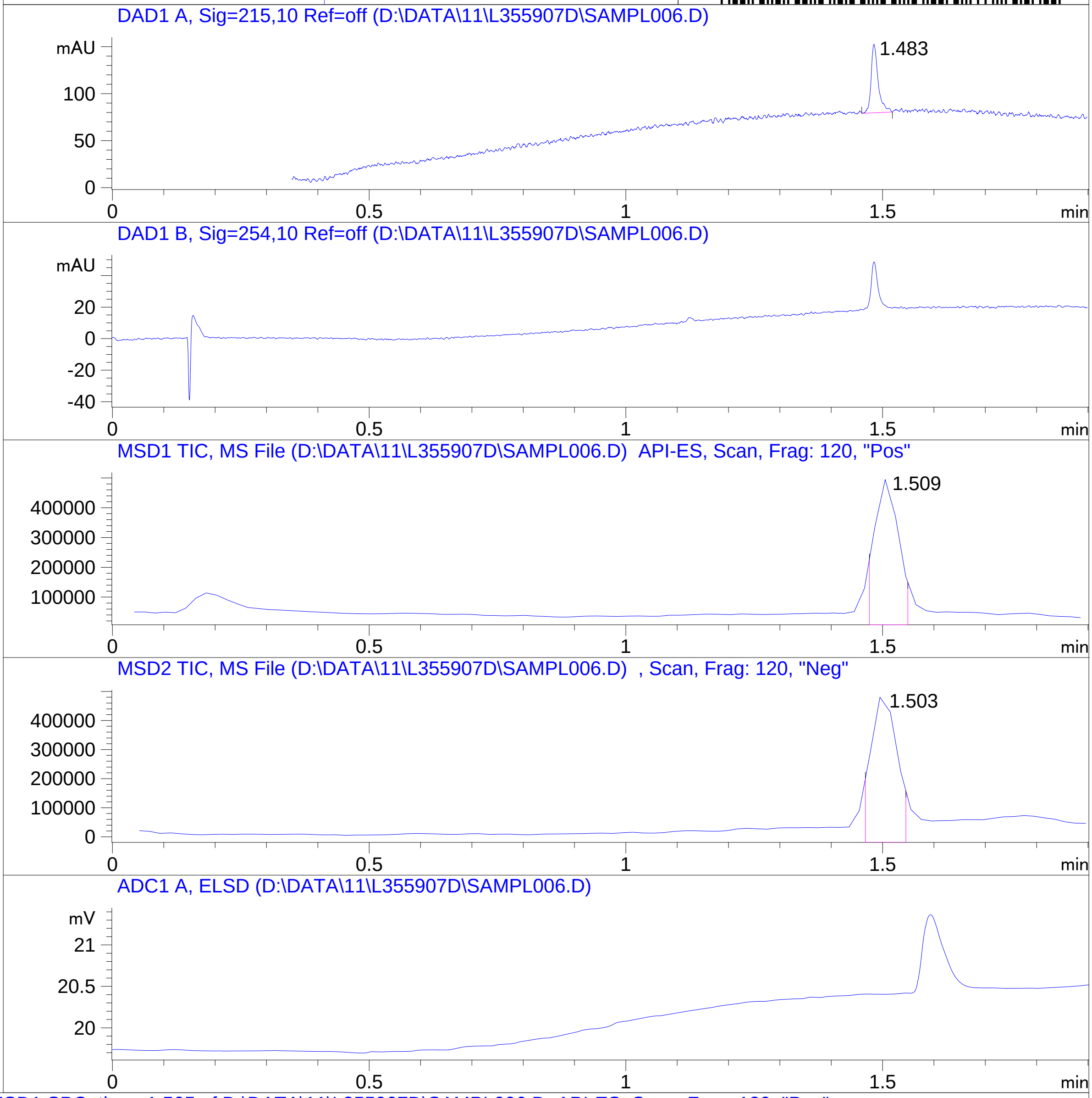

RT 1.509

RT 1.503

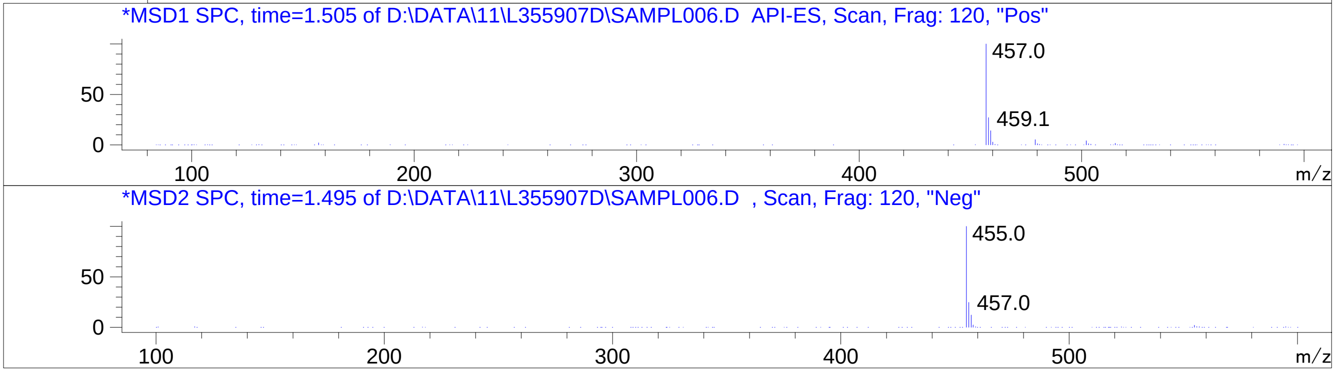

Inj.Date 4/10/2021 LB -SL- Acq. Method C:\HPCHEM\-> ->
