# Supplementary material for: AI is a viable alternative to high throughput screening: a 318-target study
Source: Sci Rep. 2024 Apr 2;14:7526. doi: 10.1038/s41598-024-54655-z (PMC10987645; doi:10.1038/s41598-024-54655-z)
Supplement: Supplementary file 1 — Supplementary Information 1. [file 41598_2024_54655_MOESM1_ESM.zip › Nature SREP/QC_AIDD_cs_selected/VCP_HID_4_LCMS.pdf]

MaxPeak: 91.11%
Ret_Time: 1.333 min

V026218$1

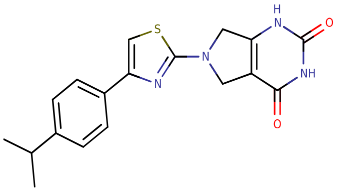

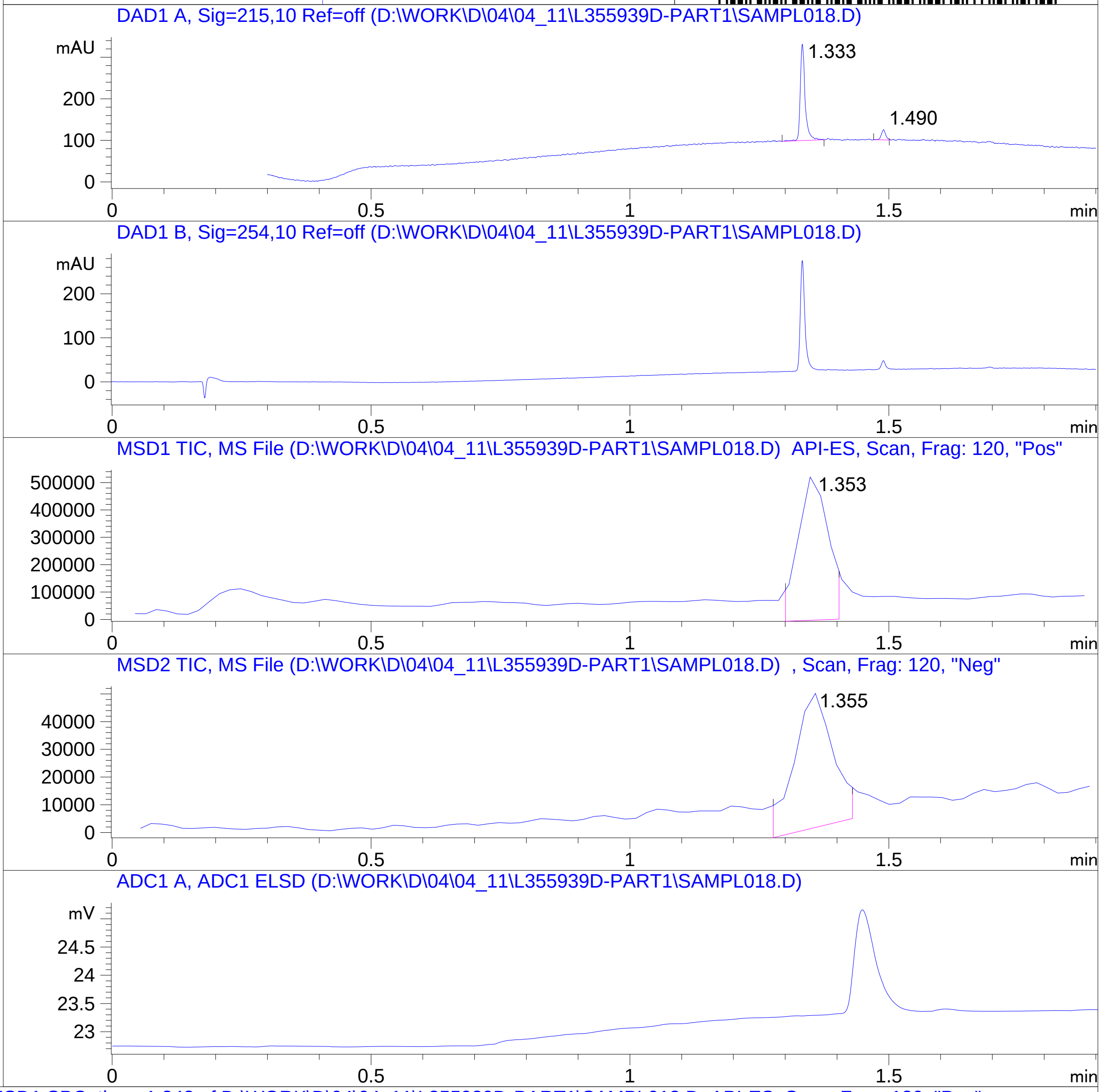

Mol Wt 354.43

Exact Mass 354.13

#	Time	Area%
1	1.333	91.11
2	1.490	8.89

RT 1.353

RT 1.355

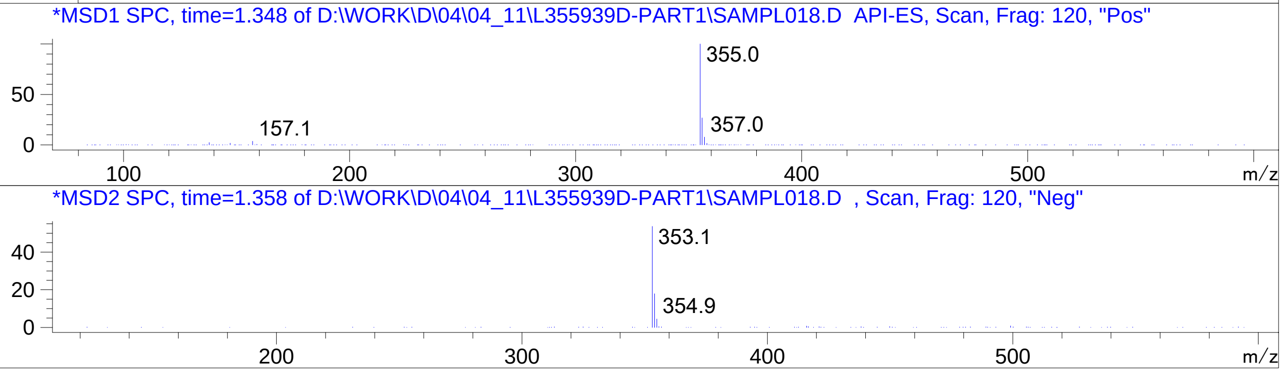

Inj.Date 4/11/2021 K -VL- Acq. Method C:\HPCHEM\-> ->
