# Supplementary material for: AI is a viable alternative to high throughput screening: a 318-target study
Source: Sci Rep. 2024 Apr 2;14:7526. doi: 10.1038/s41598-024-54655-z (PMC10987645; doi:10.1038/s41598-024-54655-z)

MaxPeak: 91.50%
Ret_Time: 1.154 min

V026221

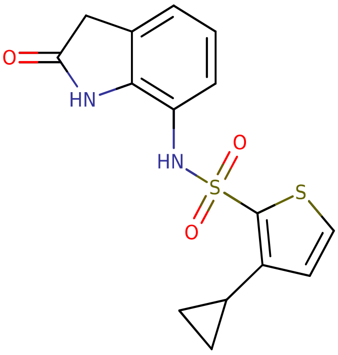

Mol Wt 334.41

Exact Mass 334.05

#	Time	Area%
1	0.647	3.24
2	1.154	91.50
3	1.187	5.26

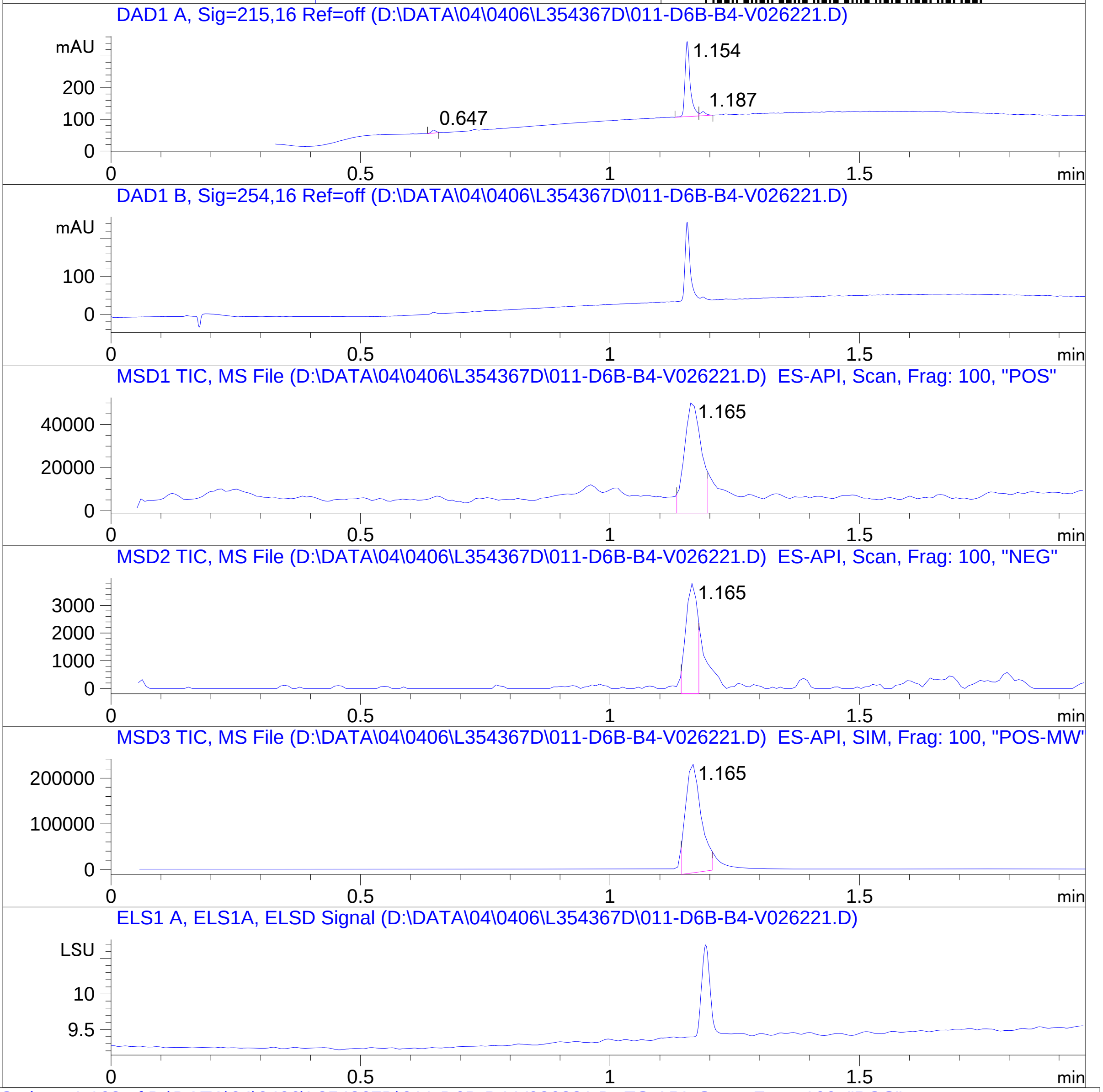

RT 1.165

RT 1.165

RT 1.165

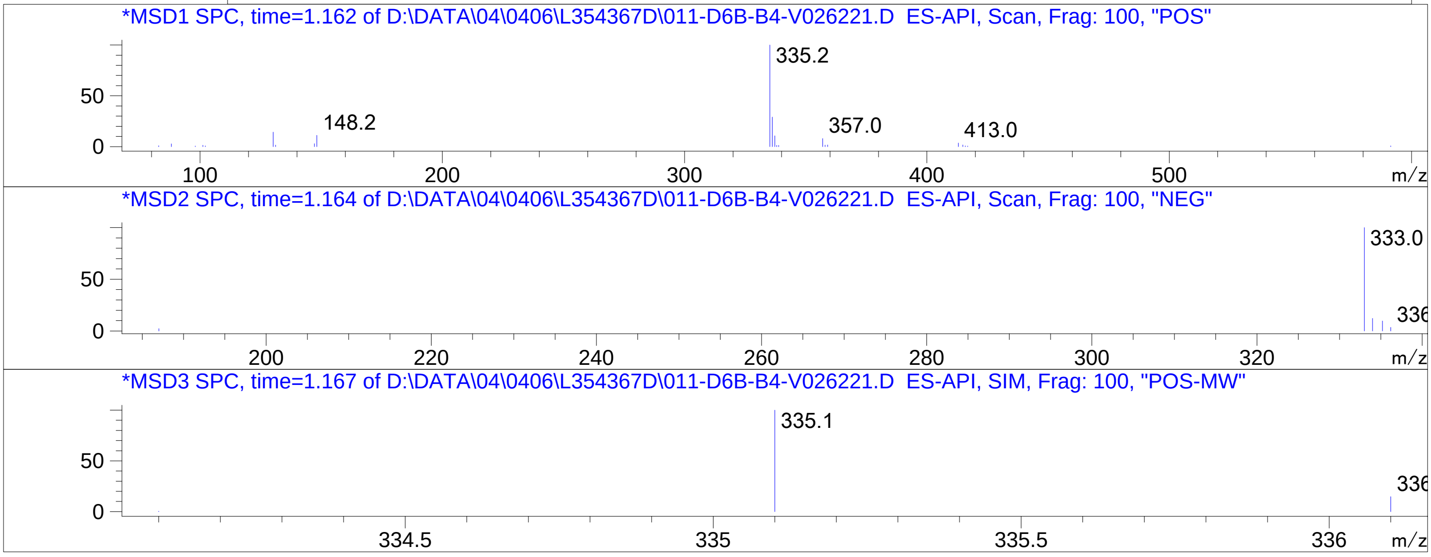

Supplement: Supplementary file 1 — Supplementary Information 1. [file 41598_2024_54655_MOESM1_ESM.zip › Nature SREP/QC_AIDD_cs_selected/VCP_HID_5_LCMS.pdf]
