# Supplementary material for: AI is a viable alternative to high throughput screening: a 318-target study
Source: Sci Rep. 2024 Apr 2;14:7526. doi: 10.1038/s41598-024-54655-z (PMC10987645; doi:10.1038/s41598-024-54655-z)
Supplement: Supplementary file 1 — Supplementary Information 1. [file 41598_2024_54655_MOESM1_ESM.zip › Nature SREP/QC_AIDD_cs_selected/VCP_HID_7_LCMS.pdf]

MaxPeak: 100.00%
Ret_Time: 1.151 min

V026774$2

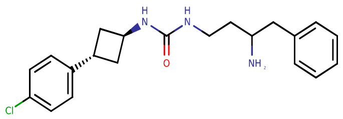

Mol Wt 371.9

Exact Mass 371.22

```
#  Time   Area%
-----------------
1  1.151  100.00
```

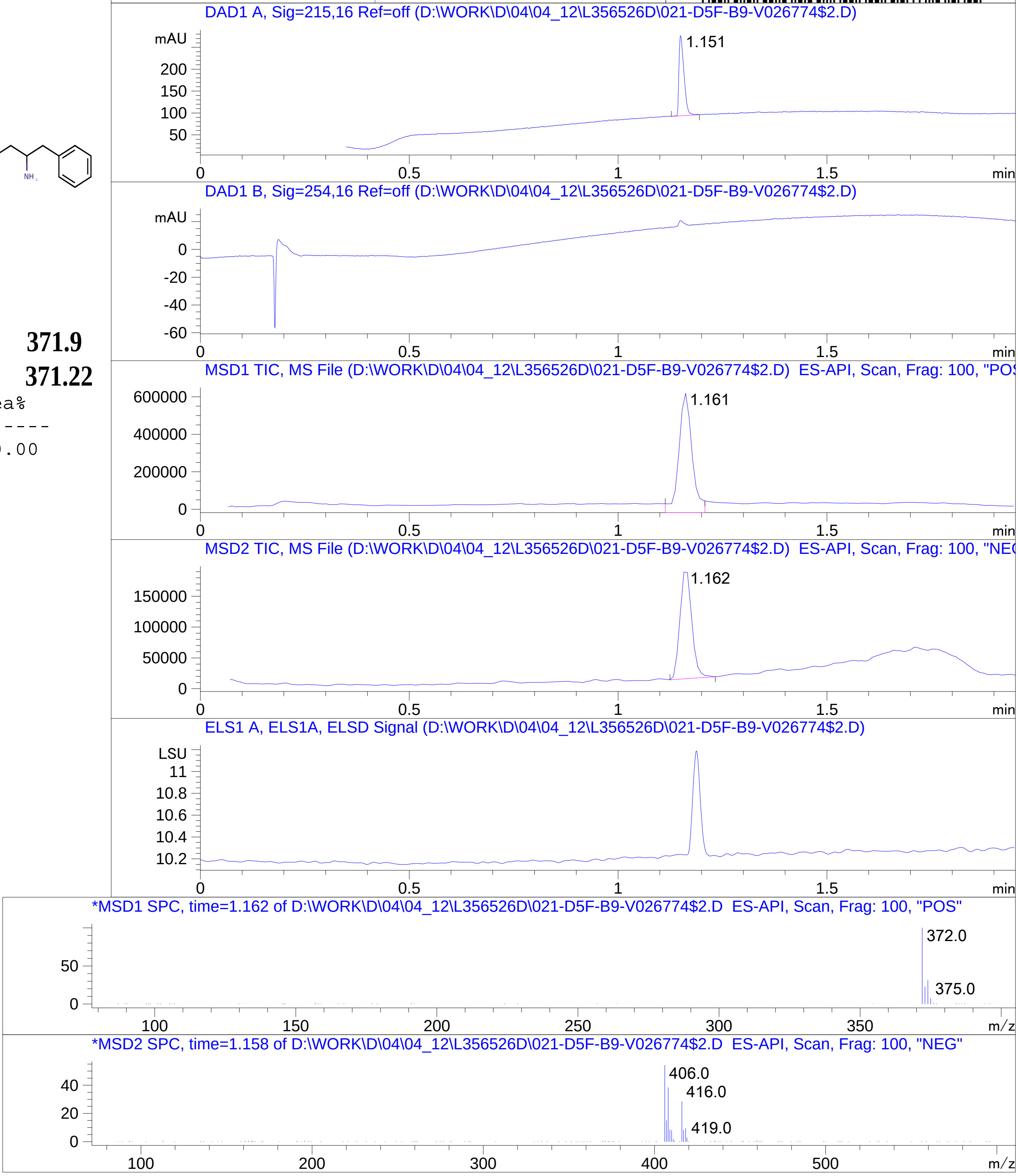

RT 1.161

RT 1.162
