# Supplementary material for: AI is a viable alternative to high throughput screening: a 318-target study
Source: Sci Rep. 2024 Apr 2;14:7526. doi: 10.1038/s41598-024-54655-z (PMC10987645; doi:10.1038/s41598-024-54655-z)
Supplement: Supplementary file 1 — Supplementary Information 1. [file 41598_2024_54655_MOESM1_ESM.zip › Nature SREP/QC_AIDD_selected/ASAH1_DR_exemplar_LCMS.pdf]

MaxPeak: 96.37%
Ret_Time: 1.082 min

U523249$1

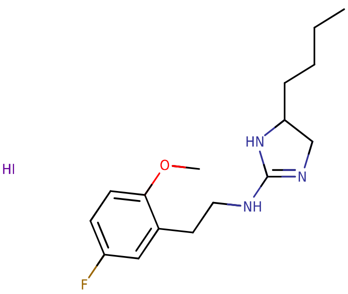

Mol Wt 421.29

Exact Mass 293.23

#	Time	Area%
1	1.082	96.37
2	1.261	3.63

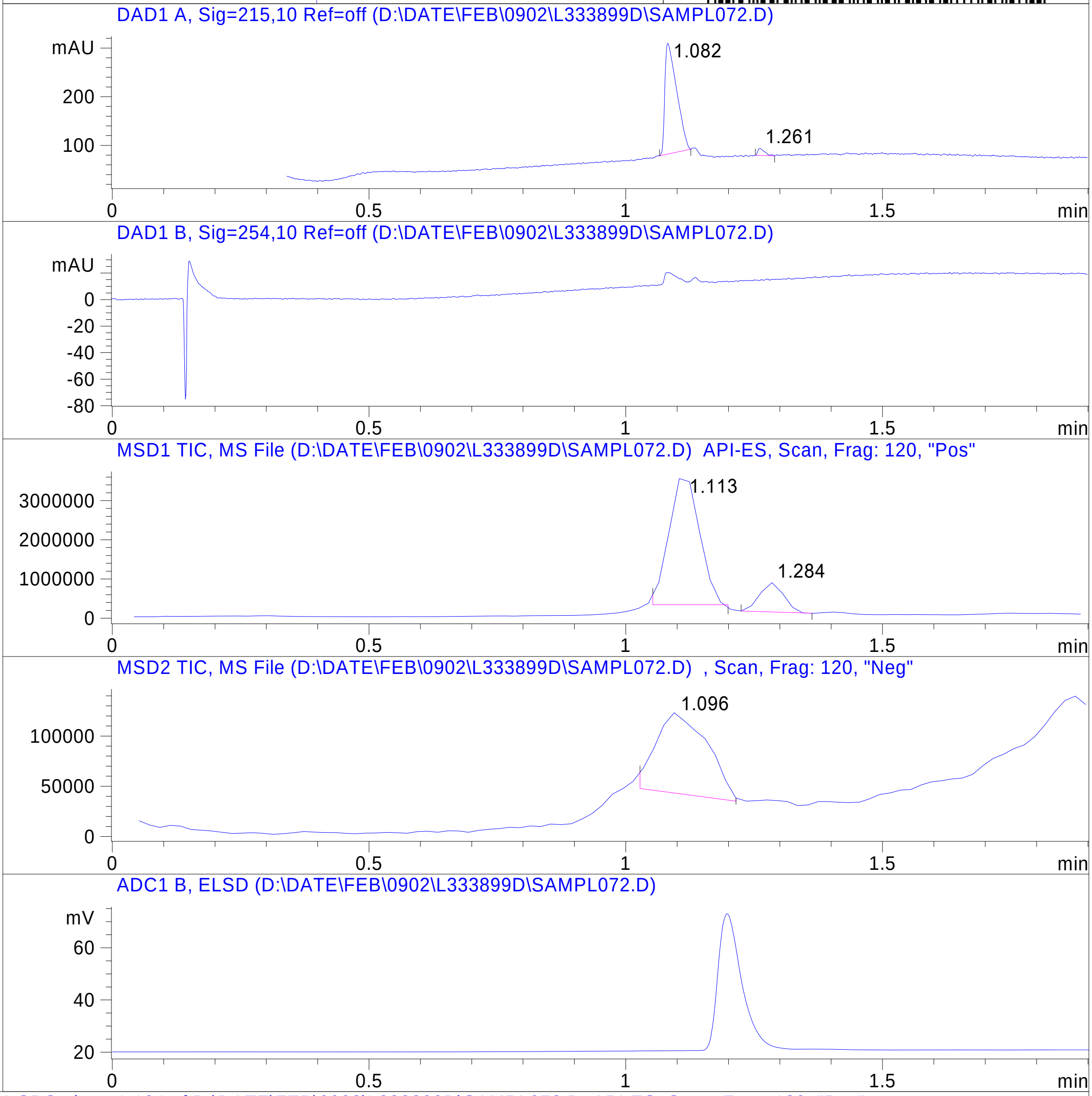

RT 1.113

RT 1.284

RT 1.096

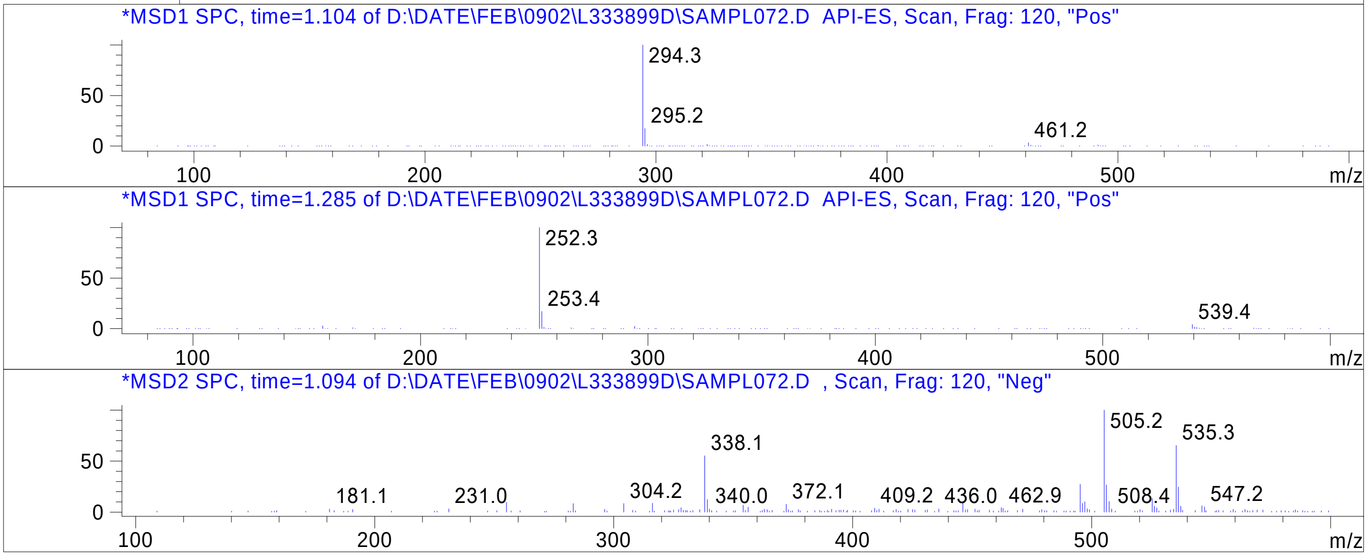

Inj.Date 2/9/2021 Y -SL- Acq. Method C:\HPCHEM\-> ->
