# Supplementary material for: AI is a viable alternative to high throughput screening: a 318-target study
Source: Sci Rep. 2024 Apr 2;14:7526. doi: 10.1038/s41598-024-54655-z (PMC10987645; doi:10.1038/s41598-024-54655-z)
Supplement: Supplementary file 1 — Supplementary Information 1. [file 41598_2024_54655_MOESM1_ESM.zip › Nature SREP/QC_AIDD_selected/AXL_DR_exemplar_LCMS.pdf]

V891692$2

MaxPeak: 97.81%
Ret_Time: 0.927 min

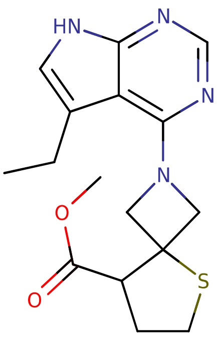

Mol Wt 332.42

Exact Mass 332.15

#	Time	Area%
1	0.927	97.81
2	1.062	2.19

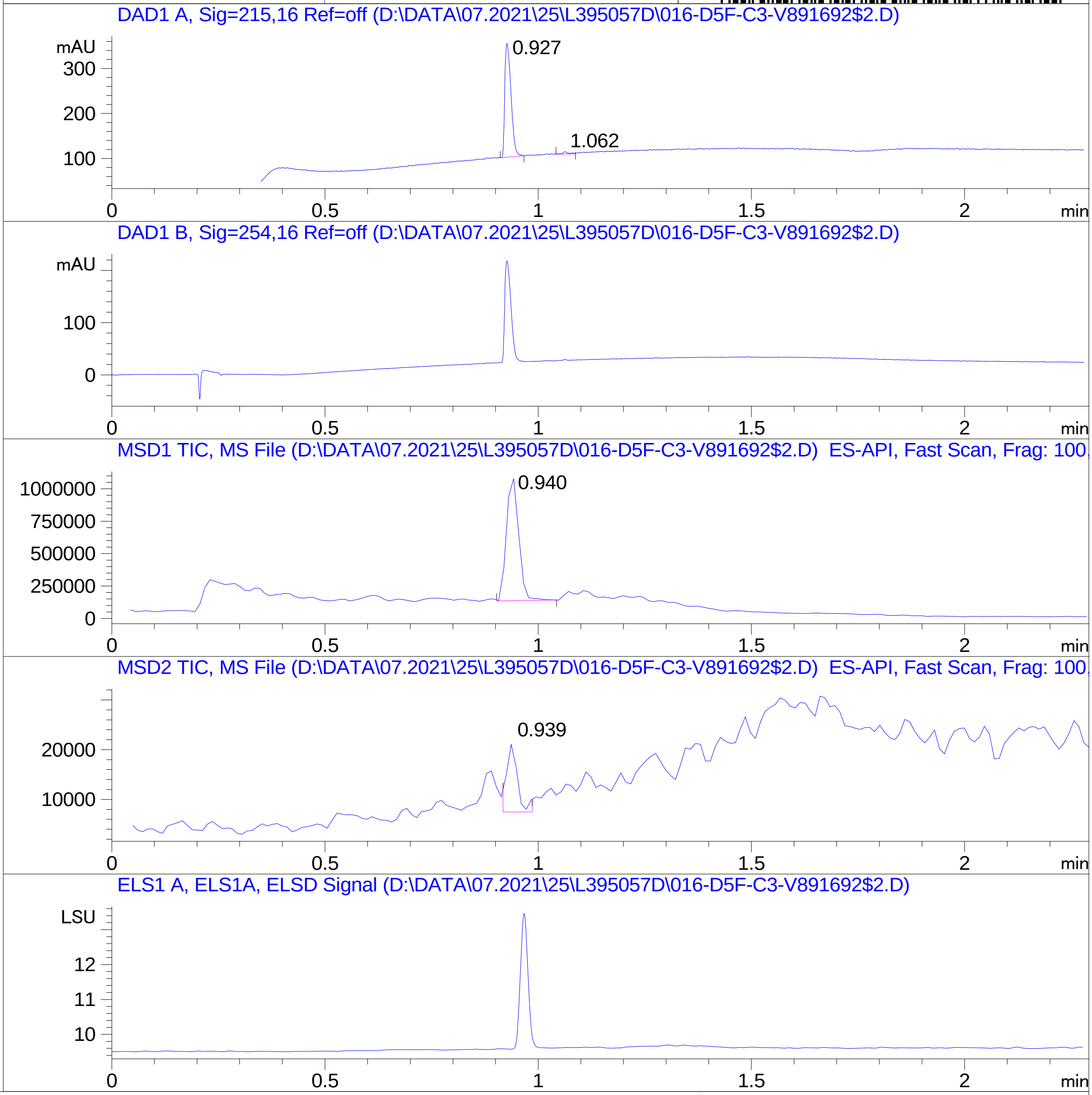

RT 0.940

RT 0.939

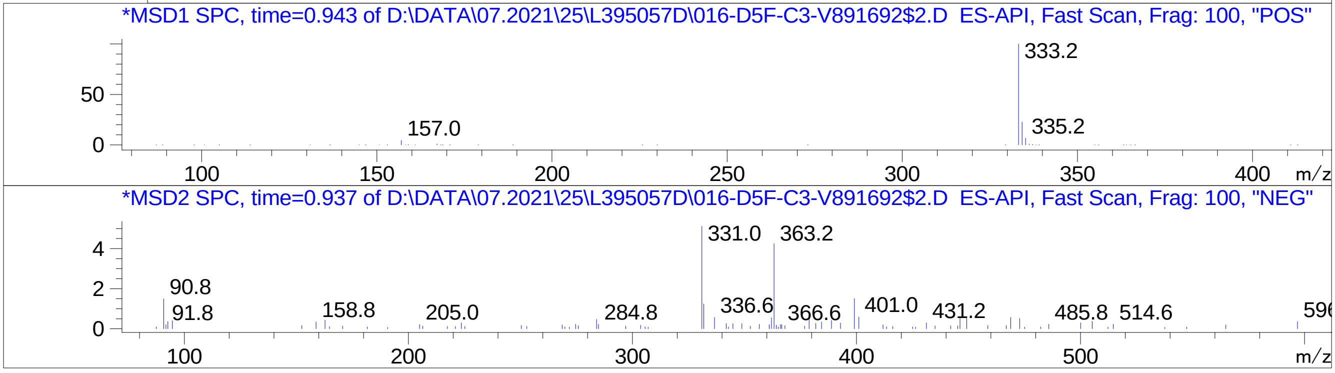

Inj.Date 7/23/2021 CH <invalid> 22 Acq. Method C:\Users\ -> ->
