# Supplementary material for: AI is a viable alternative to high throughput screening: a 318-target study
Source: Sci Rep. 2024 Apr 2;14:7526. doi: 10.1038/s41598-024-54655-z (PMC10987645; doi:10.1038/s41598-024-54655-z)
Supplement: Supplementary file 1 — Supplementary Information 1. [file 41598_2024_54655_MOESM1_ESM.zip › Nature SREP/QC_AIDD_selected/CDK5_DR_exemplar_LCMS.pdf]

U753243$2

```
MaxPeak:  100.00%
Ret_Time: 0.792 min
```

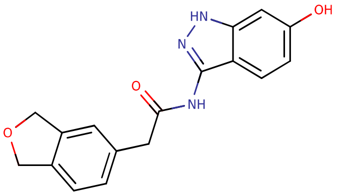

Mol Wt 309.32

Exact Mass 309.12

#	Time	Area%
1	0.792	100.00

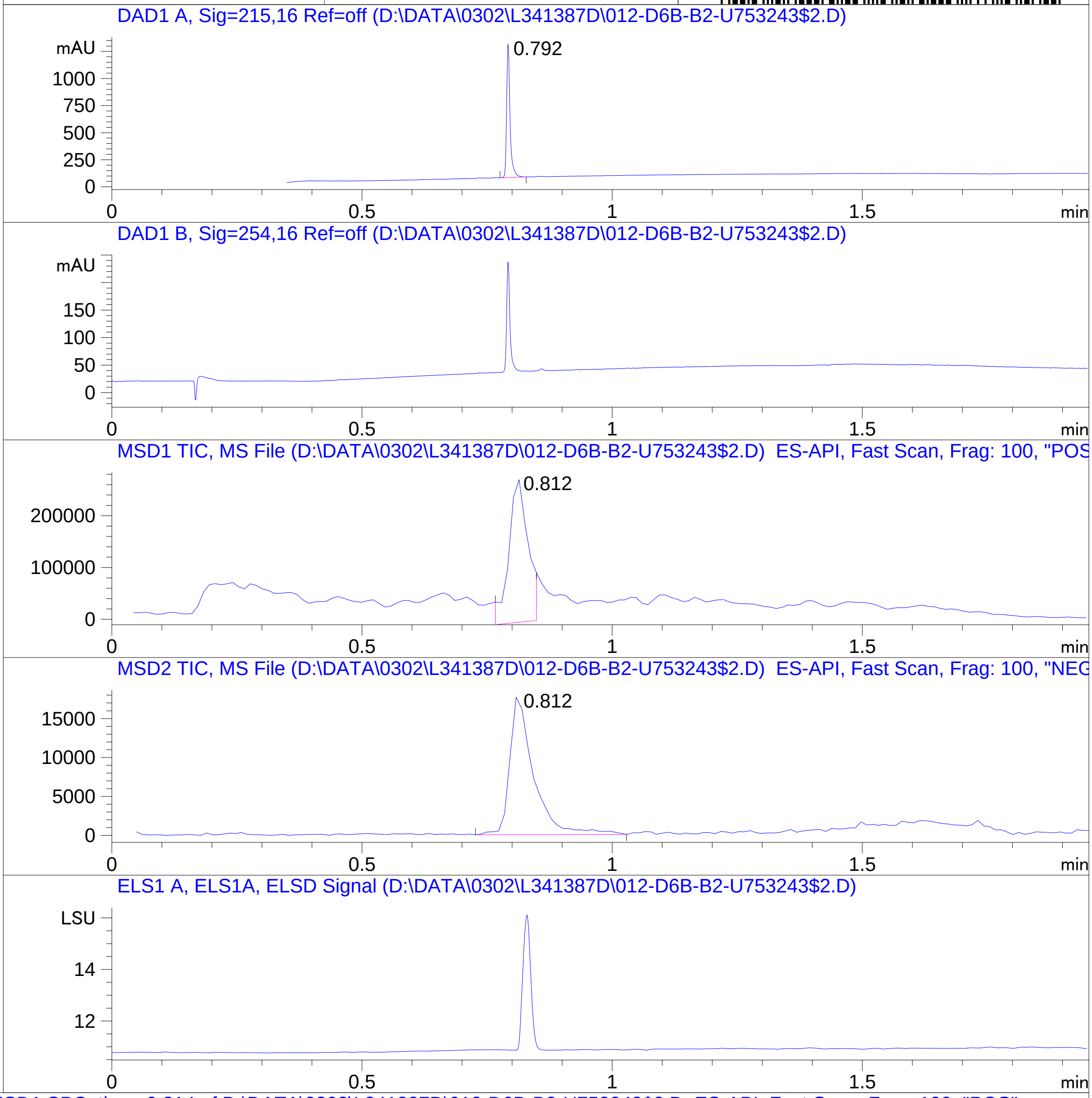

RT 0.812

RT 0.812

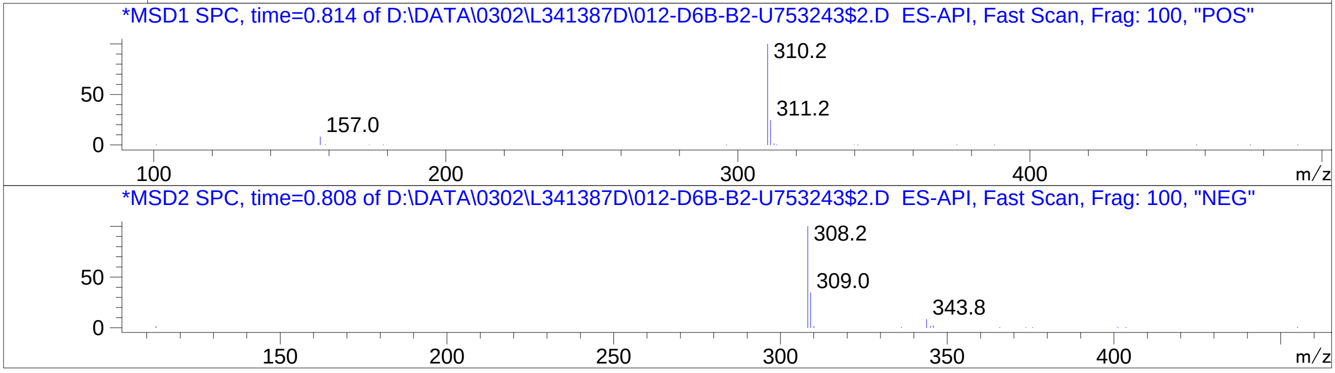

Inj.Date 3/1/2021 OA Acq. Method C:\Users\ -> ->
