# Supplementary material for: AI is a viable alternative to high throughput screening: a 318-target study
Source: Sci Rep. 2024 Apr 2;14:7526. doi: 10.1038/s41598-024-54655-z (PMC10987645; doi:10.1038/s41598-024-54655-z)

V263291$2

MaxPeak: 100.00%
Ret_Time: 1.028 min

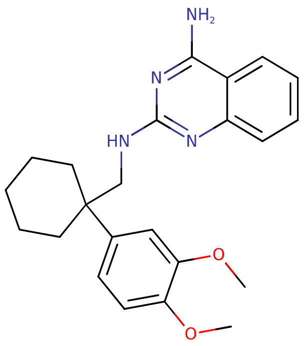

Mol Wt 392.49

Exact Mass 392.26

#	Time	Area%
1	1.028	100.00

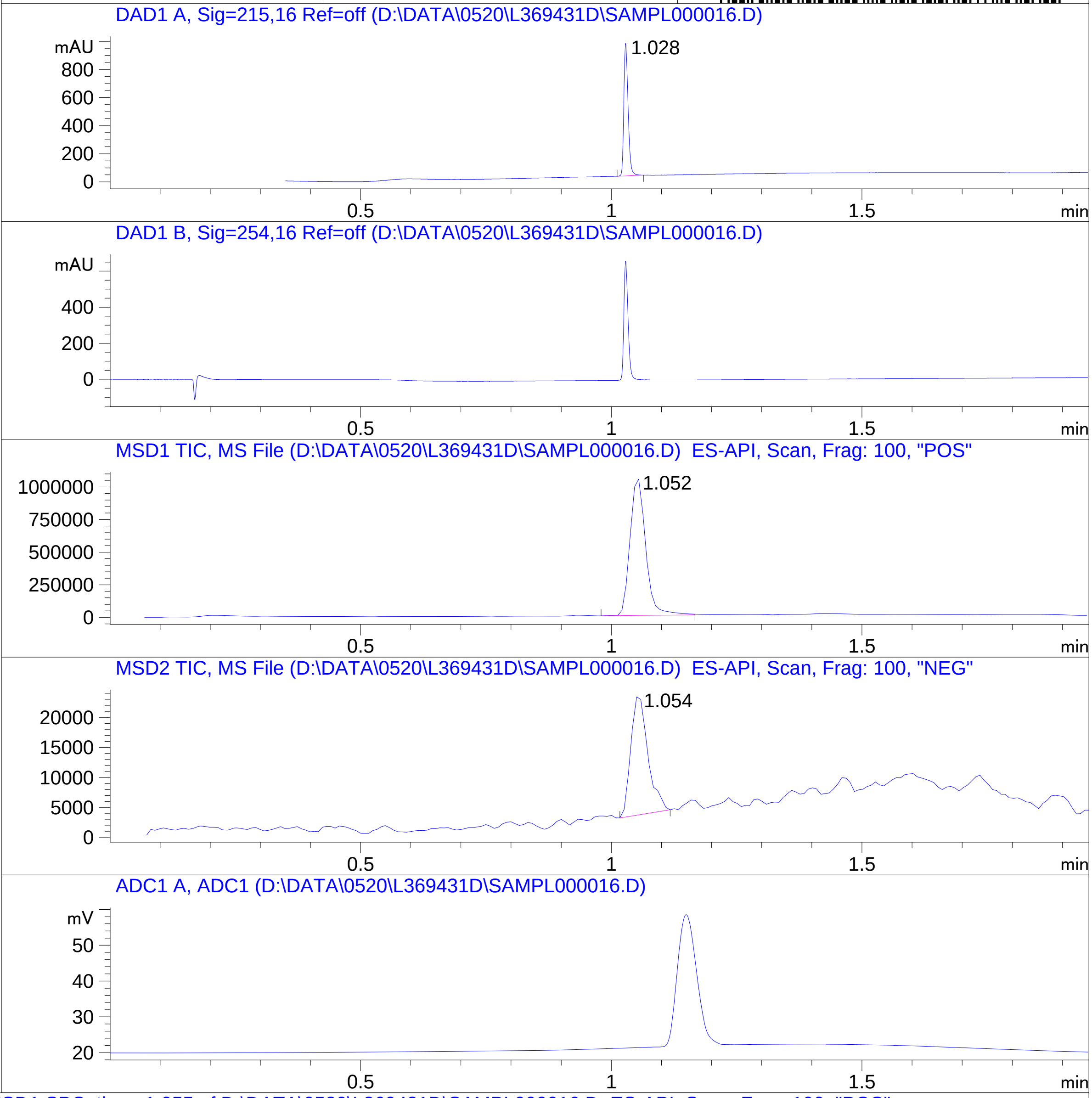

RT 1.052

RT 1.054

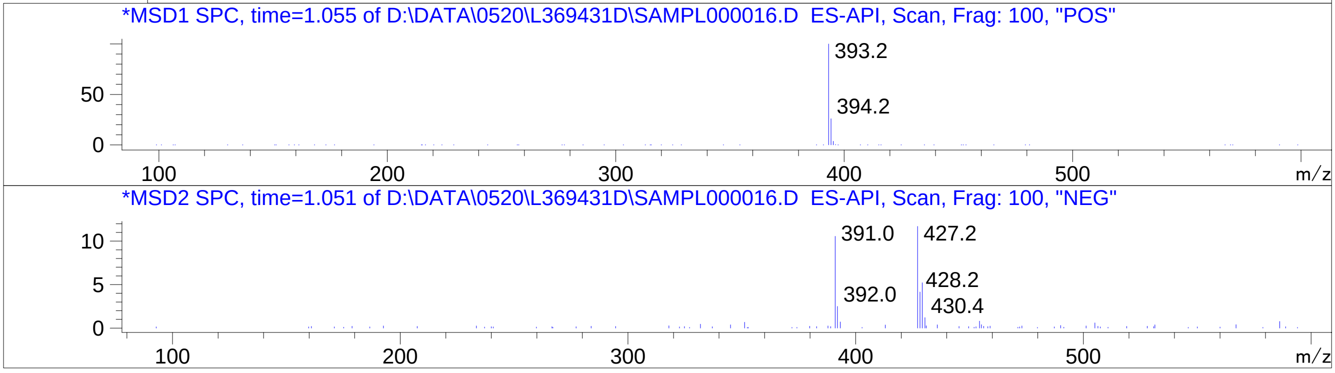

Supplement: Supplementary file 1 — Supplementary Information 1. [file 41598_2024_54655_MOESM1_ESM.zip › Nature SREP/QC_AIDD_selected/KCNT1_DR_exemplar_LCMS.pdf]
