# Supplementary material for: AI is a viable alternative to high throughput screening: a 318-target study
Source: Sci Rep. 2024 Apr 2;14:7526. doi: 10.1038/s41598-024-54655-z (PMC10987645; doi:10.1038/s41598-024-54655-z)
Supplement: Supplementary file 1 — Supplementary Information 1. [file 41598_2024_54655_MOESM1_ESM.zip › Nature SREP/QC_AIDD_selected/KDM6A_DR_exemplar_LCMS.pdf]

MaxPeak: 93.98%
Ret_Time: 1.359 min

V183812$3

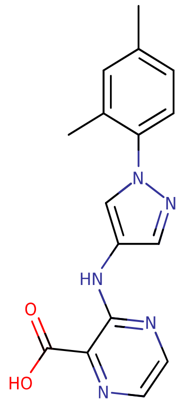

Mol Wt 309.32

Exact Mass 309.13

#	Time	Area%
1	1.132	1.59
2	1.248	4.43
3	1.359	93.98

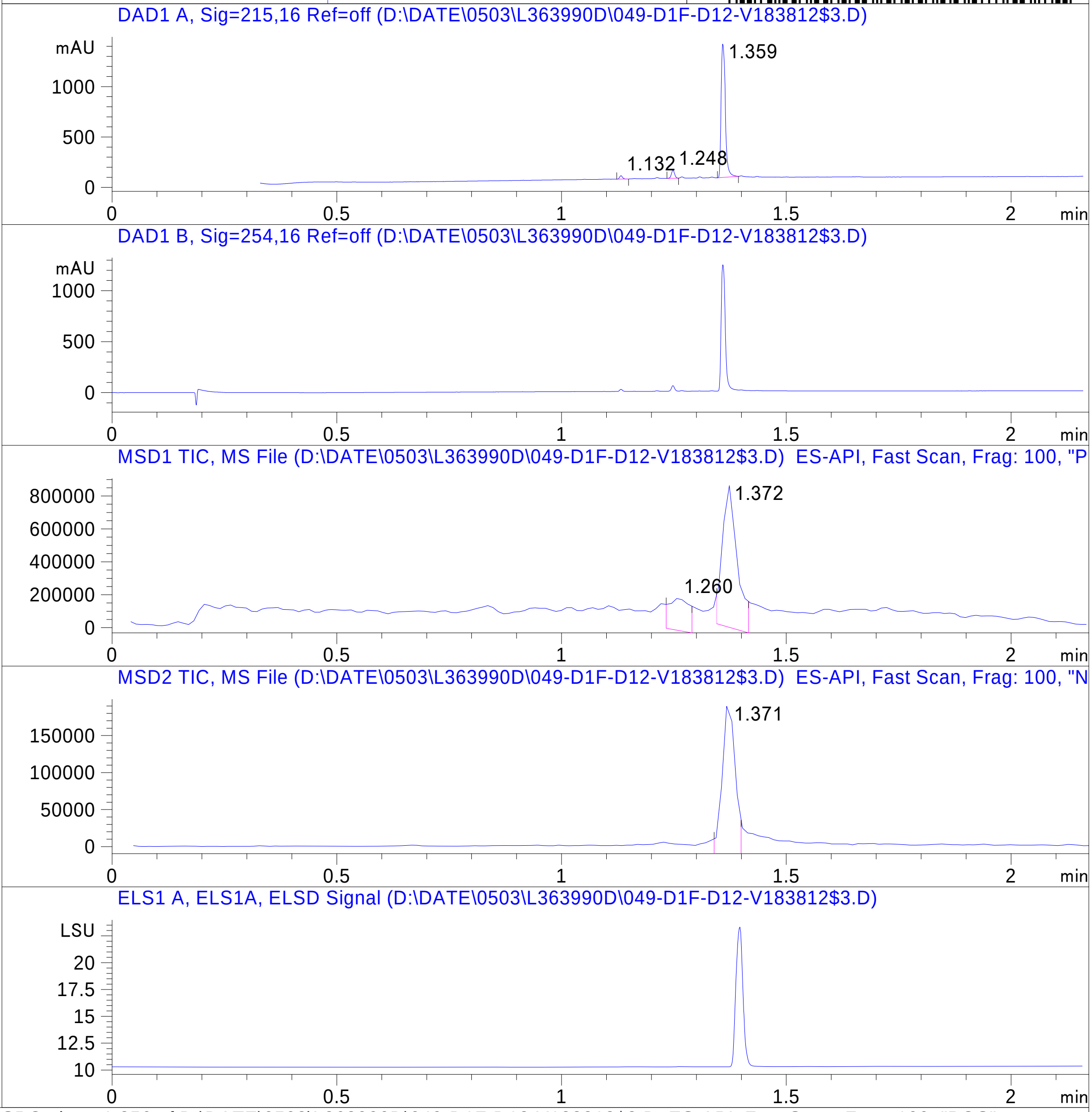

RT 1.260

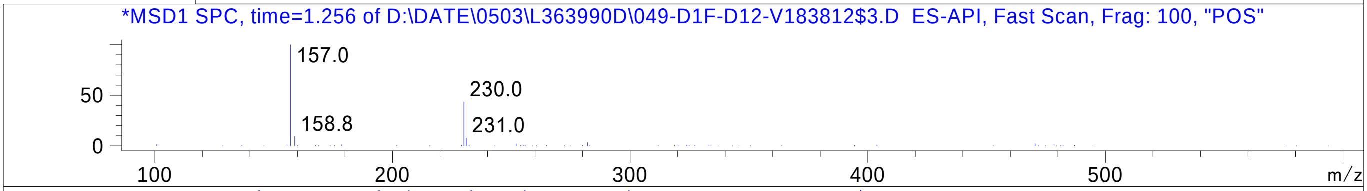

RT 1.372

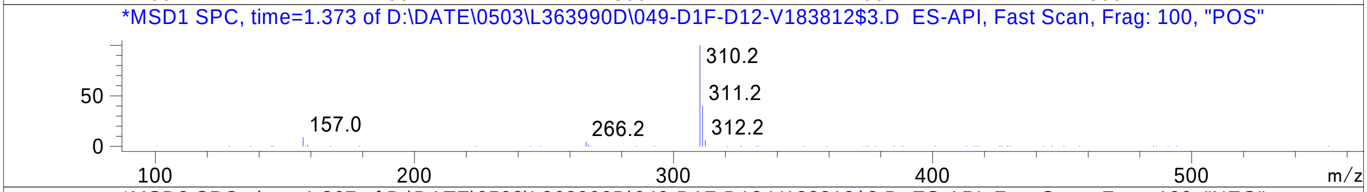

RT 1.371

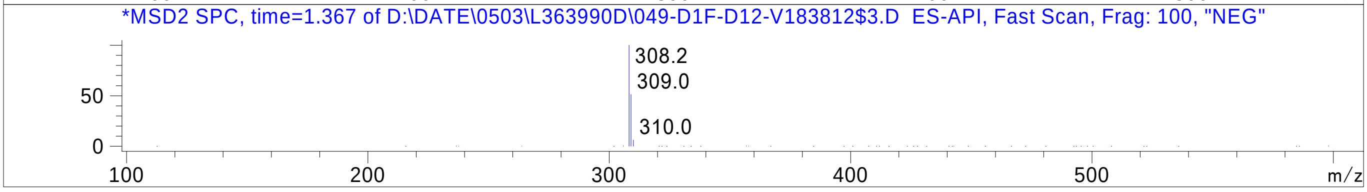

Inj.Date 5/2/2021 E Acq. Method C:\Users\ -> ->
