# Supplementary material for: AI is a viable alternative to high throughput screening: a 318-target study
Source: Sci Rep. 2024 Apr 2;14:7526. doi: 10.1038/s41598-024-54655-z (PMC10987645; doi:10.1038/s41598-024-54655-z)
Supplement: Supplementary file 1 — Supplementary Information 1. [file 41598_2024_54655_MOESM1_ESM.zip › Nature SREP/QC_AIDD_selected/POLQ_DR_exemplar_LCMS.pdf]

MaxPeak: 100.00%
Ret_Time: 1.099 min

U403796$9

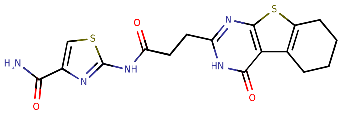


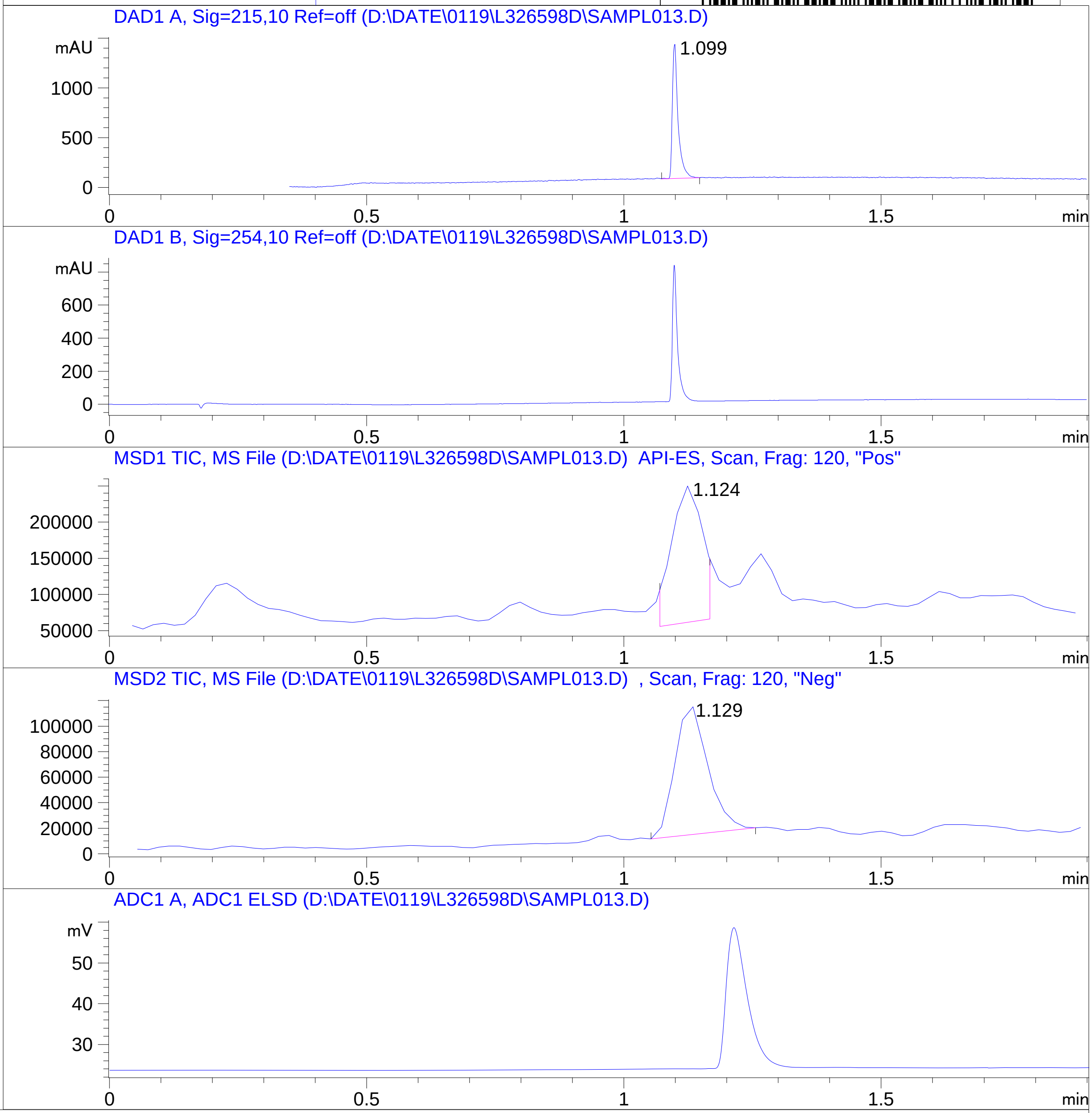


**Mol Wt** 403.48

**Exact Mass** 403.08

```
#  Time   Area%
-----------------
1  1.099  100.00
```

RT 1.124

RT 1.129

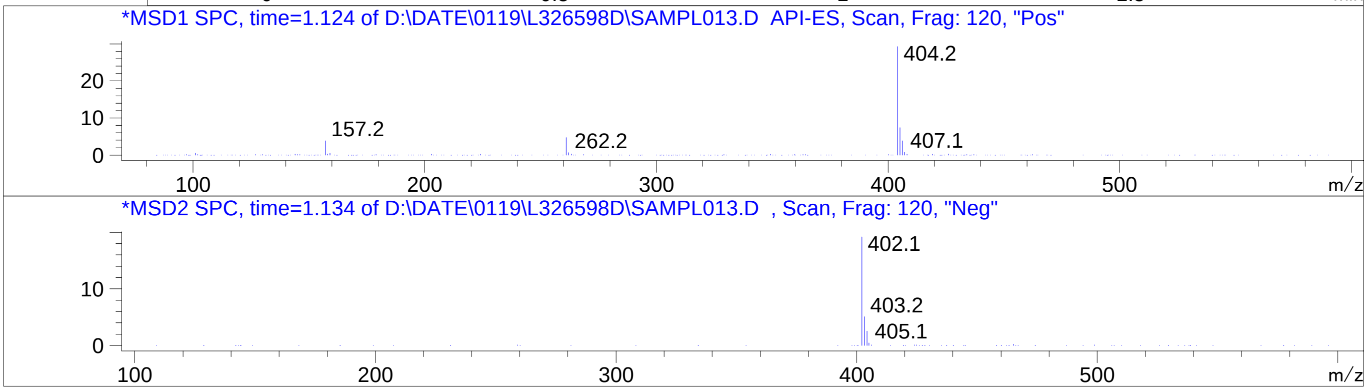


Inj.Date 1/19/2021    O    -VL-    Acq. Method C:\HPCHEM\->    ->
