# Supplementary material for: AI is a viable alternative to high throughput screening: a 318-target study
Source: Sci Rep. 2024 Apr 2;14:7526. doi: 10.1038/s41598-024-54655-z (PMC10987645; doi:10.1038/s41598-024-54655-z)
Supplement: Supplementary file 1 — Supplementary Information 1. [file 41598_2024_54655_MOESM1_ESM.zip › Nature SREP/QC_AIDD_selected/PPARA_DR_exemplar_LCMS.pdf]

MaxPeak: 91.51%
Ret_Time: 0.940 min

V488746$2

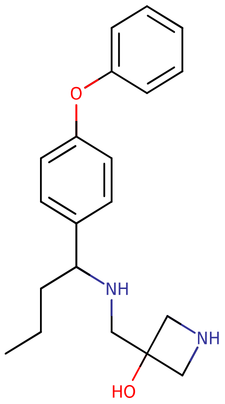

Mol Wt 326.43

Exact Mass 326.24

#	Time	Area%
1	0.940	91.51
2	1.066	1.29
3	1.113	4.77
4	1.181	2.42

DAD1 A, Sig=215,10 Ref=off (D:\DATE\0614\L379385D-PART1\SAMPL011.D)

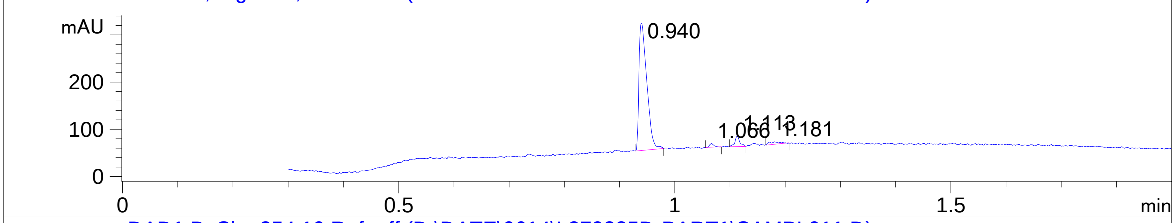

DAD1 B, Sig=254,10 Ref=off (D:\DATE\0614\L379385D-PART1\SAMPL011.D)

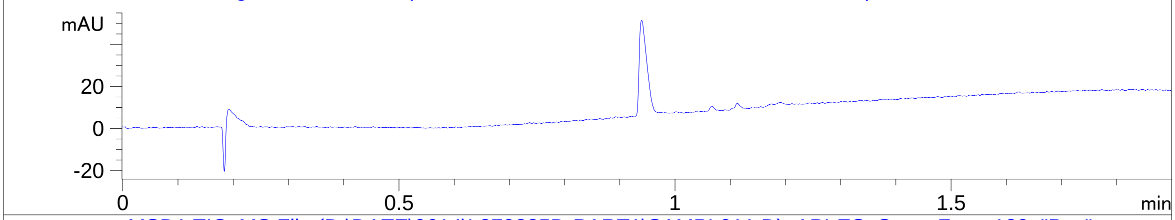

MSD1 TIC, MS File (D:\DATE\0614\L379385D-PART1\SAMPL011.D) API-ES, Scan, Frag: 120, "Pos"

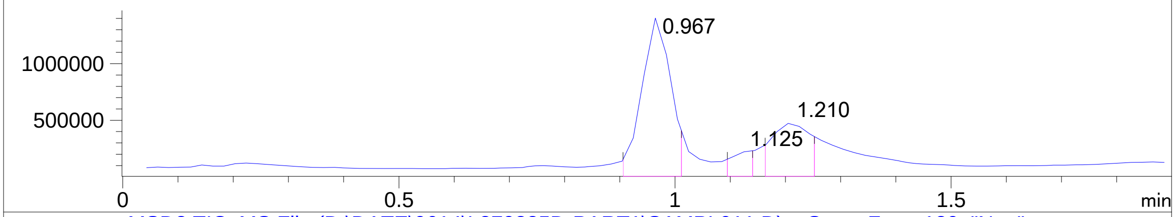

MSD2 TIC, MS File (D:\DATE\0614\L379385D-PART1\SAMPL011.D) , Scan, Frag: 120, "Neg"

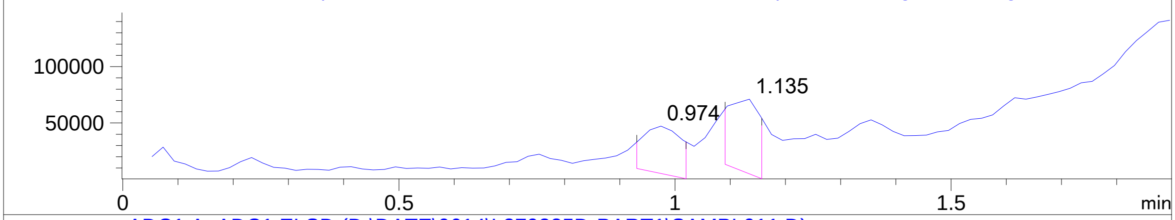

ADC1 A, ADC1 ELSD (D:\DATE\0614\L379385D-PART1\SAMPL011.D)

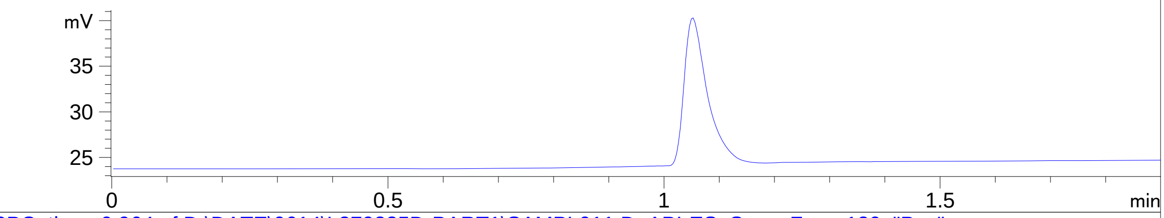

RT 0.967

*MSD1 SPC, time=0.964 of D:\DATE\0614\L379385D-PART1\SAMPL011.D API-ES, Scan, Frag: 120, "Pos"

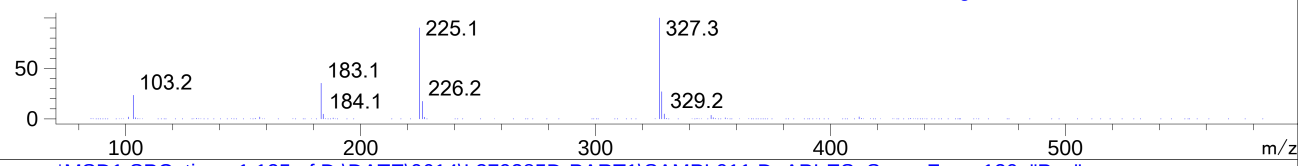

RT 1.125

*MSD1 SPC, time=1.125 of D:\DATE\0614\L379385D-PART1\SAMPL011.D API-ES, Scan, Frag: 120, "Pos"

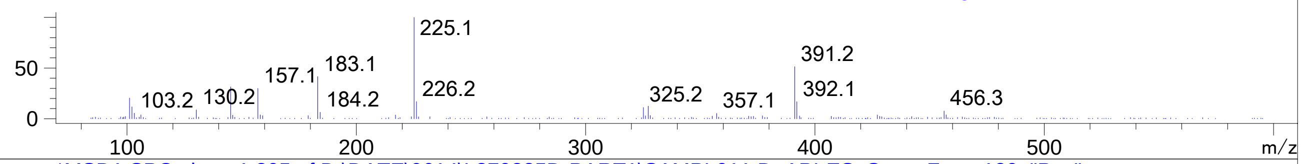

RT 1.210

*MSD1 SPC, time=1.205 of D:\DATE\0614\L379385D-PART1\SAMPL011.D API-ES, Scan, Frag: 120, "Pos"

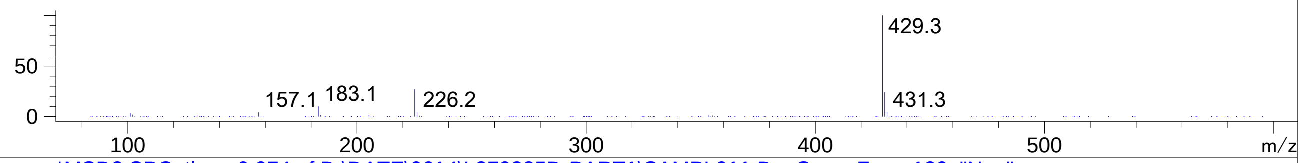

RT 0.974

*MSD2 SPC, time=0.974 of D:\DATE\0614\L379385D-PART1\SAMPL011.D , Scan, Frag: 120, "Neg"

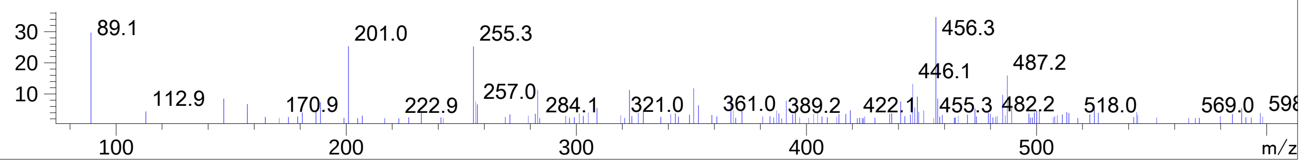

Inj.Date 6/13/2021 E -SL- Acq. Method C:\HPCHEM\-> ->

RT 1.135

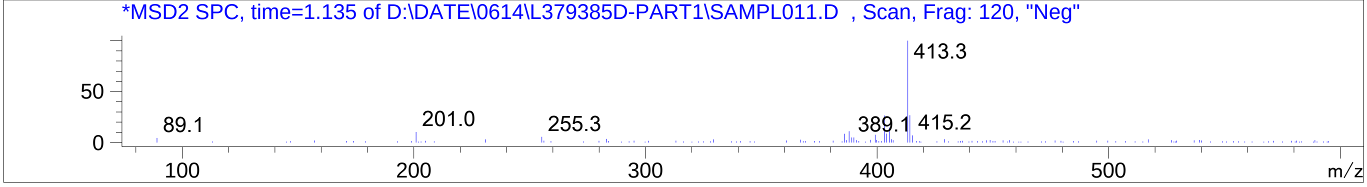

*MSD2 SPC, time=1.135 of D:\DATE\0614\L379385D-PART1\SAMPL011.D , Scan, Frag: 120, "Neg"
413.3
50
0
89.1
201.0
255.3
389.1
415.2
100
200
300
400
500
m/z
