# Supplementary material for: AI is a viable alternative to high throughput screening: a 318-target study
Source: Sci Rep. 2024 Apr 2;14:7526. doi: 10.1038/s41598-024-54655-z (PMC10987645; doi:10.1038/s41598-024-54655-z)
Supplement: Supplementary file 1 — Supplementary Information 1. [file 41598_2024_54655_MOESM1_ESM.zip › Nature SREP/QC_AIDD_selected/PPM1D_DR_exemplar_LCMS.pdf]

MaxPeak: 96.89%
Ret_Time: 0.784 min

W229174

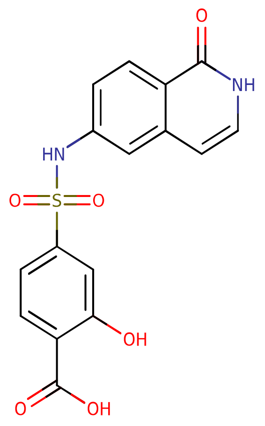

Mol Wt 360.34

Exact Mass 360.03

#	Time	Area%
1	0.644	1.67
2	0.784	96.89
3	0.925	1.44

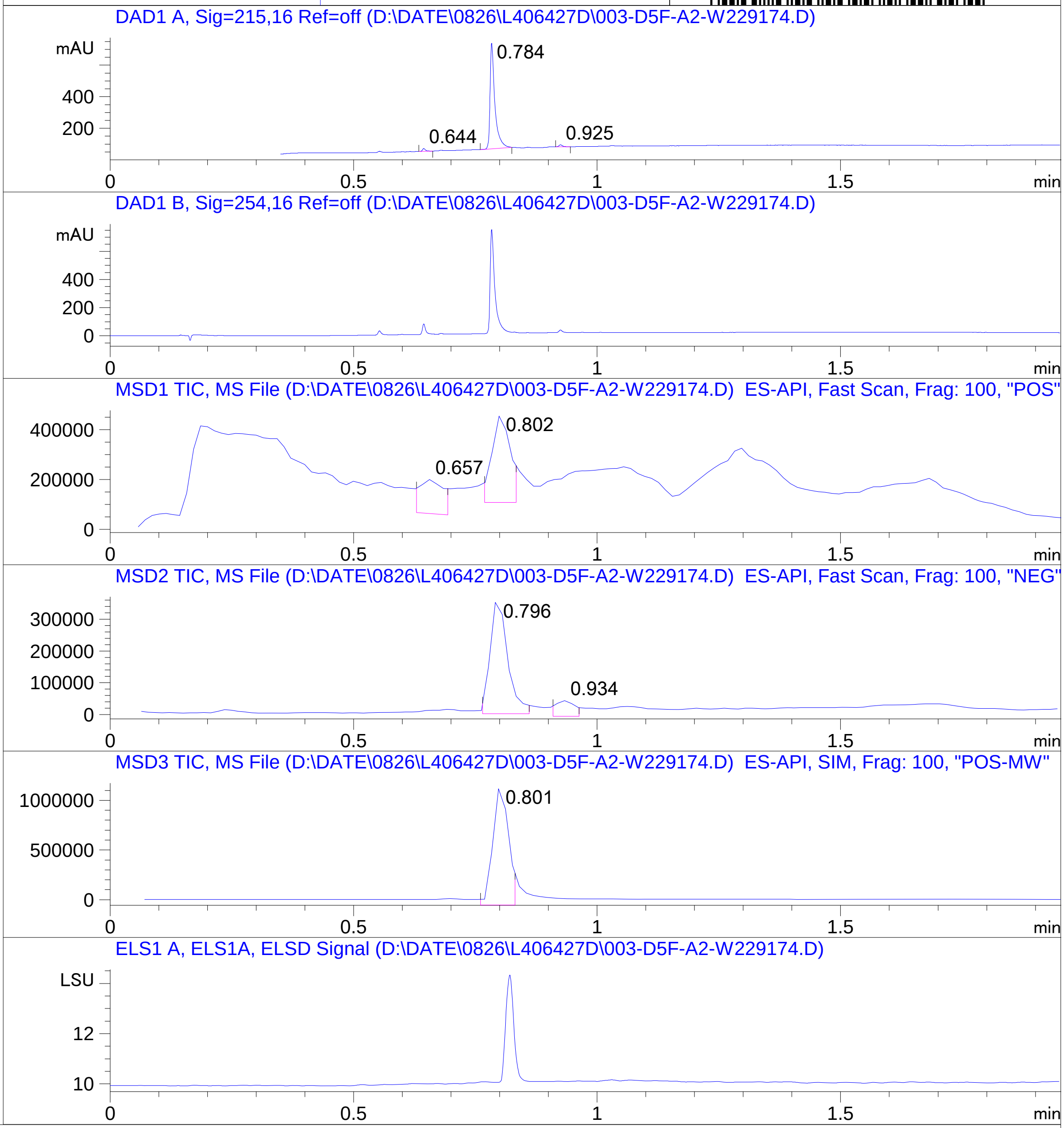

RT 0.657

RT 0.802

RT 0.796

RT 0.934

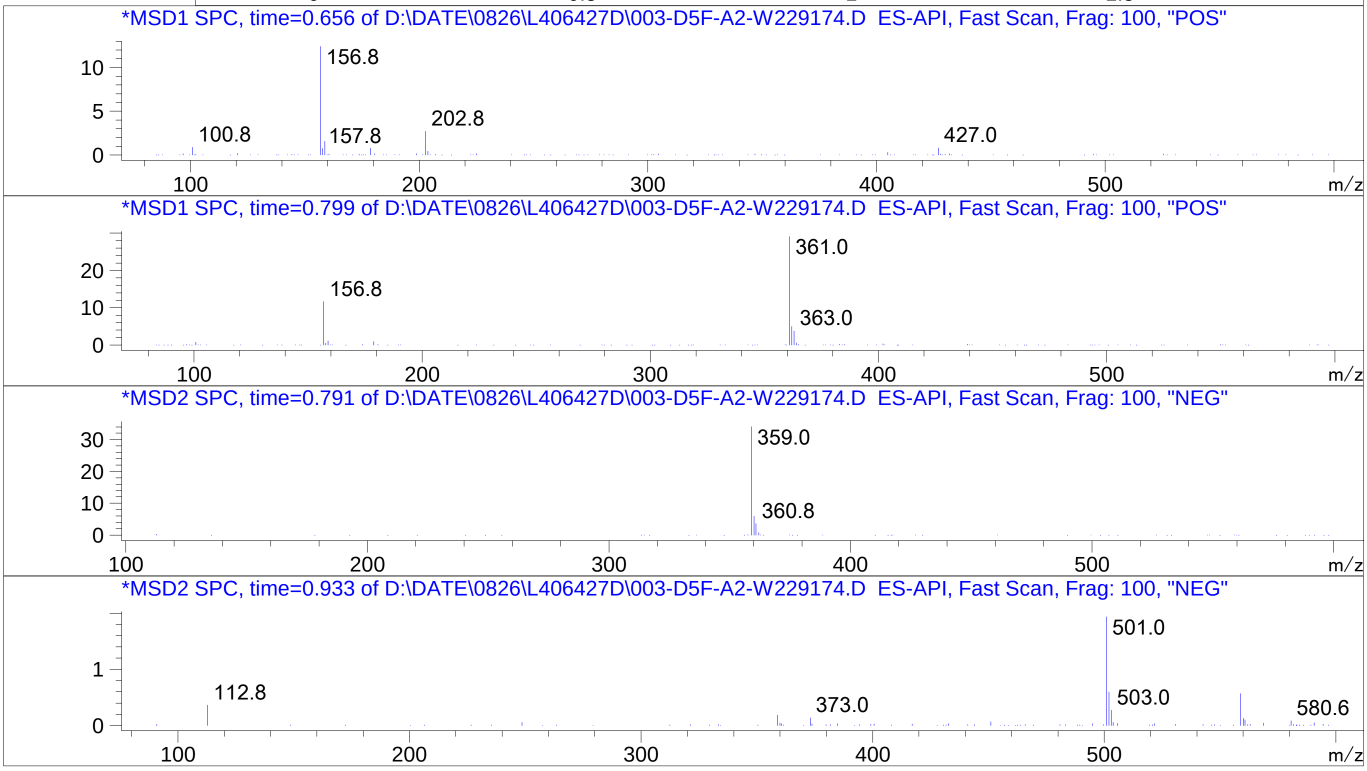

Inj.Date 8/26/2021 E Acq. Method C:\Users\ -> ->

RT 0.801

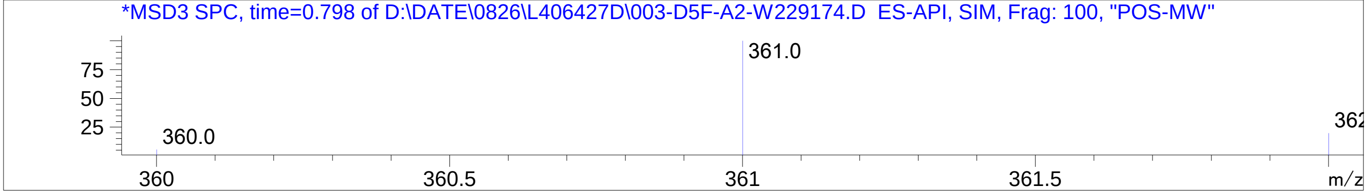

Inj.Date 8/26/2021 E Acq. Method C:\Users\ -> ->
