# Supplementary material for: AI is a viable alternative to high throughput screening: a 318-target study
Source: Sci Rep. 2024 Apr 2;14:7526. doi: 10.1038/s41598-024-54655-z (PMC10987645; doi:10.1038/s41598-024-54655-z)
Supplement: Supplementary file 1 — Supplementary Information 1. [file 41598_2024_54655_MOESM1_ESM.zip › Nature SREP/QC_AIDD_selected/PRODH2_DR_exemplar_LCMS.pdf]

V790470

```
MaxPeak:   97.83%
Ret_Time: 1.374 min
```

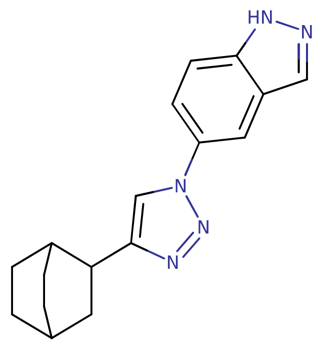

Mol Wt 293.37

Exact Mass 293.19

#	Time	Area%
1	1.309	2.17
2	1.374	97.83

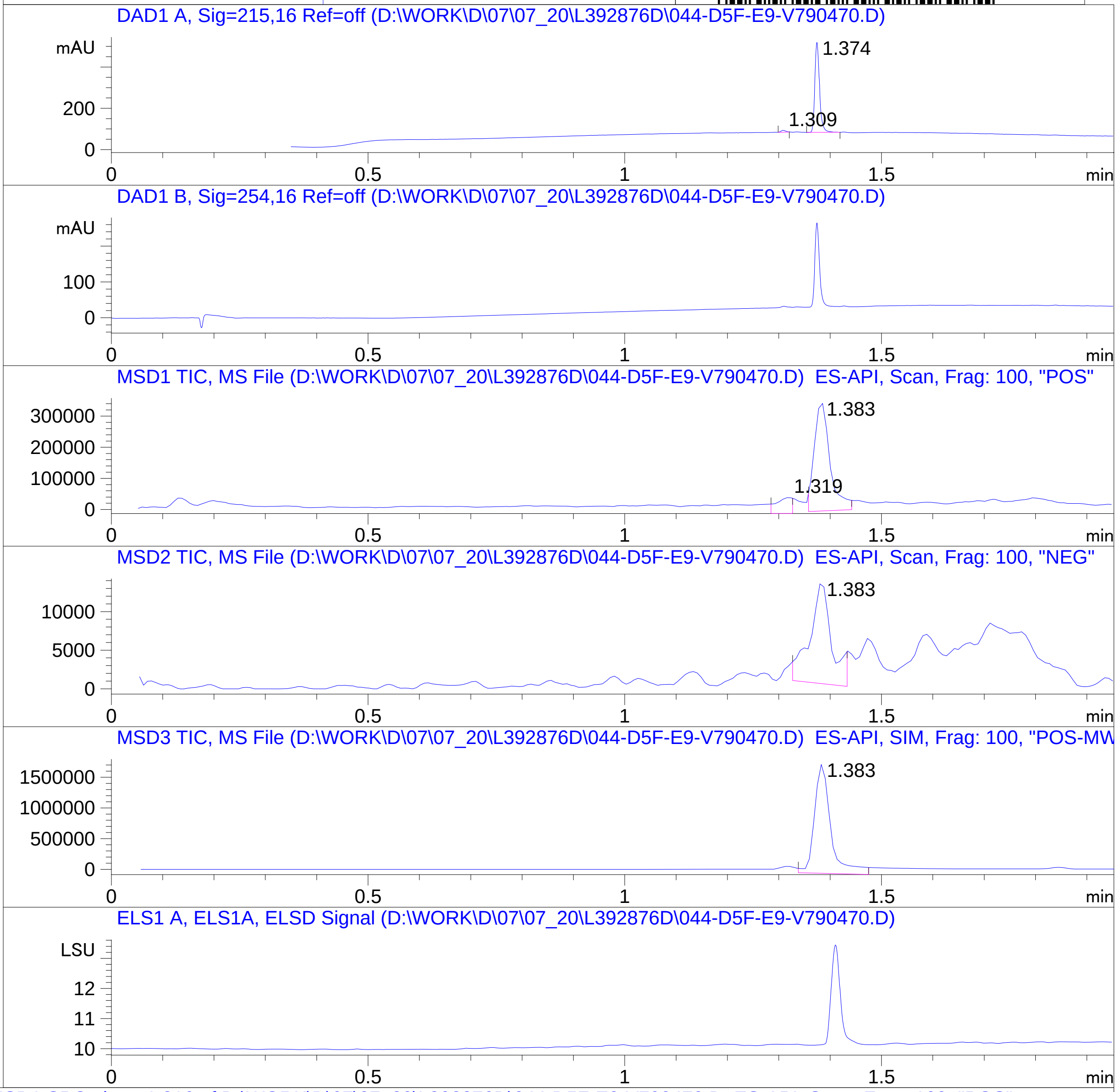

RT 1.319

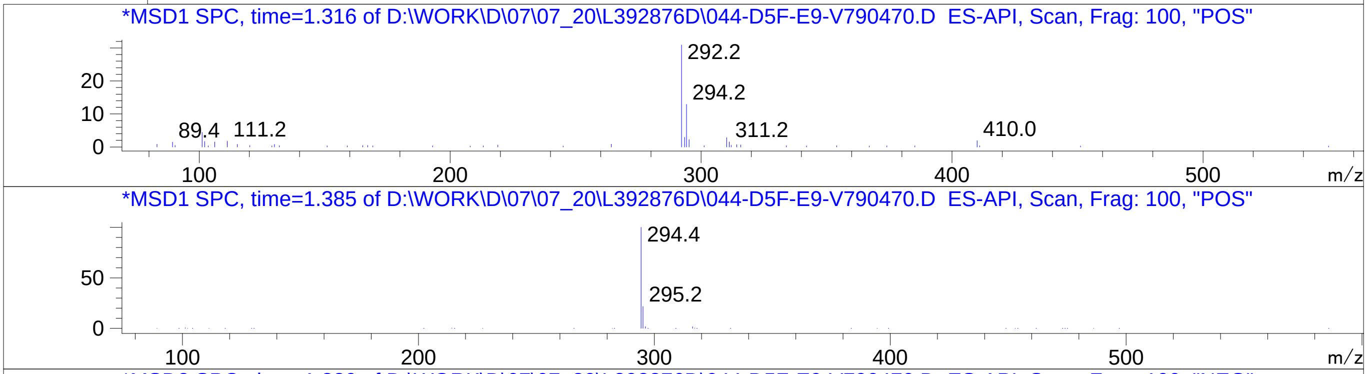

RT 1.383

RT 1.383

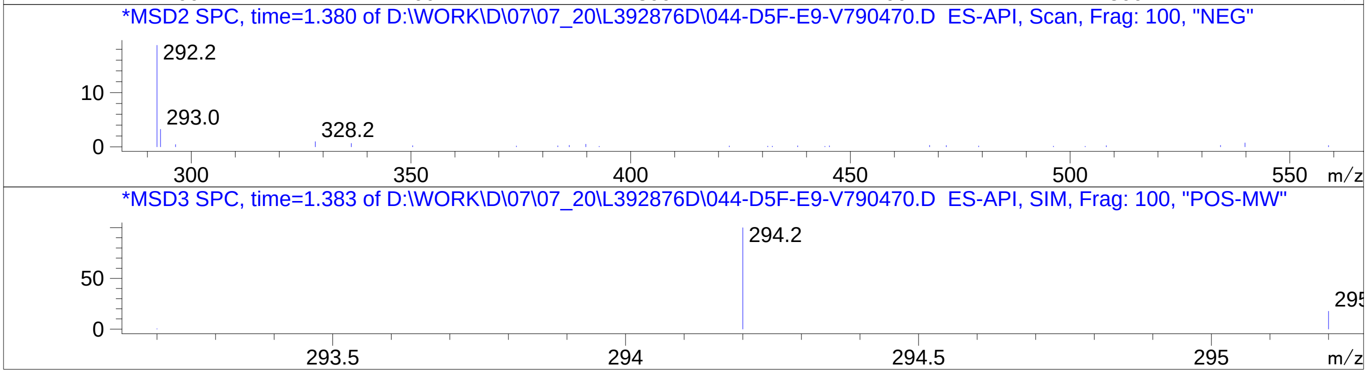

RT 1.383


```
Inj.Date 7/19/2021          K      &lt;invalid&gt; -16-     Acq. Method C:\Chem32\->   ->
```
