# Supplementary material for: AI is a viable alternative to high throughput screening: a 318-target study
Source: Sci Rep. 2024 Apr 2;14:7526. doi: 10.1038/s41598-024-54655-z (PMC10987645; doi:10.1038/s41598-024-54655-z)
Supplement: Supplementary file 1 — Supplementary Information 1. [file 41598_2024_54655_MOESM1_ESM.zip › Nature SREP/QC_AIMS_files/Proj008.pdf]

-.o.-Syntez Purity Report -.o.-

Agilent 1100 LC/MSD SL
Diodearrey G1315B (DAD1A-215nm; DAD1B-254nm)
Mass Quad G1956B (MSD1-Pos, MSD2-Neg)
ELSD Altech 3300 (ADC1 A, ELSD)

Mobile fase:A-H2O+0.1%HCOOH;B-MeOH+0.1%HCOOH
Separation column:
Rapid Resolutionn HT Cartige 4.6x30mm,
1.8-Micron, Zorbx SB-C18

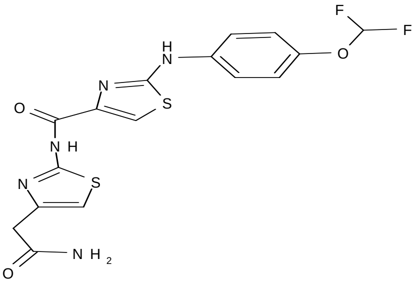

Mol.Weight: 425,44

F5448-0336

M4362 ->

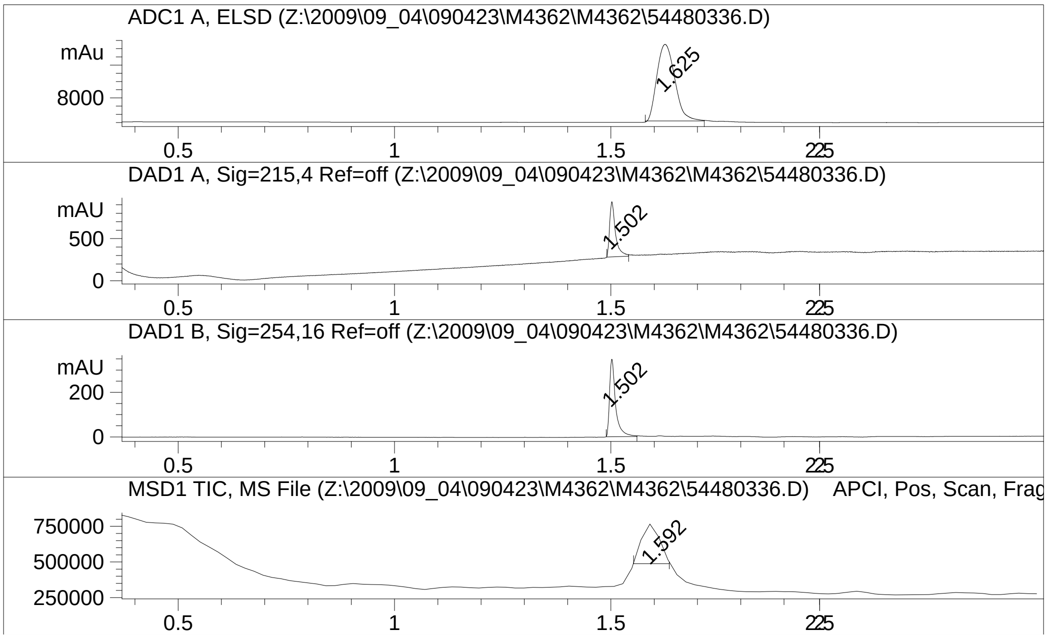

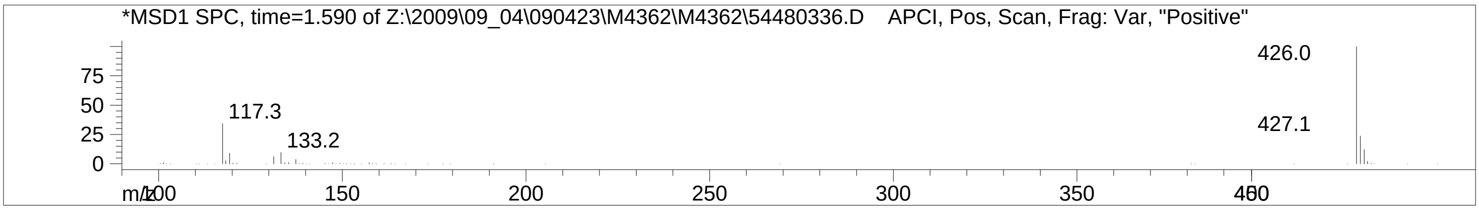

#	Signal	R.Time	Area %
1	ADC1 A, ELSD	1.625	100.000

#	Signal	R.Time	Area %
1	DAD1 A, Sig=215,4 Ref=off	1.502	100.000

#	Signal	R.Time	Area %
1	DAD1 B, Sig=254,16 Ref=off	1.502	100.000

#	Signal	R.Time	Area %
1	MSD1 TIC, MS File	1.592	100.000
