# Supplementary material for: AI is a viable alternative to high throughput screening: a 318-target study
Source: Sci Rep. 2024 Apr 2;14:7526. doi: 10.1038/s41598-024-54655-z (PMC10987645; doi:10.1038/s41598-024-54655-z)
Supplement: Supplementary file 1 — Supplementary Information 1. [file 41598_2024_54655_MOESM1_ESM.zip › Nature SREP/QC_AIMS_files/Proj016.PDF]

Sample: 128
File: 9575_28
Vial: D/4

Date: 26-Aug-2004
Time: 13:34:48
Description: 1006167

AMRI code: ALB-H05990348
Vial label: M5988802AMP0098

DAD: 220

max. intensity: 2E6

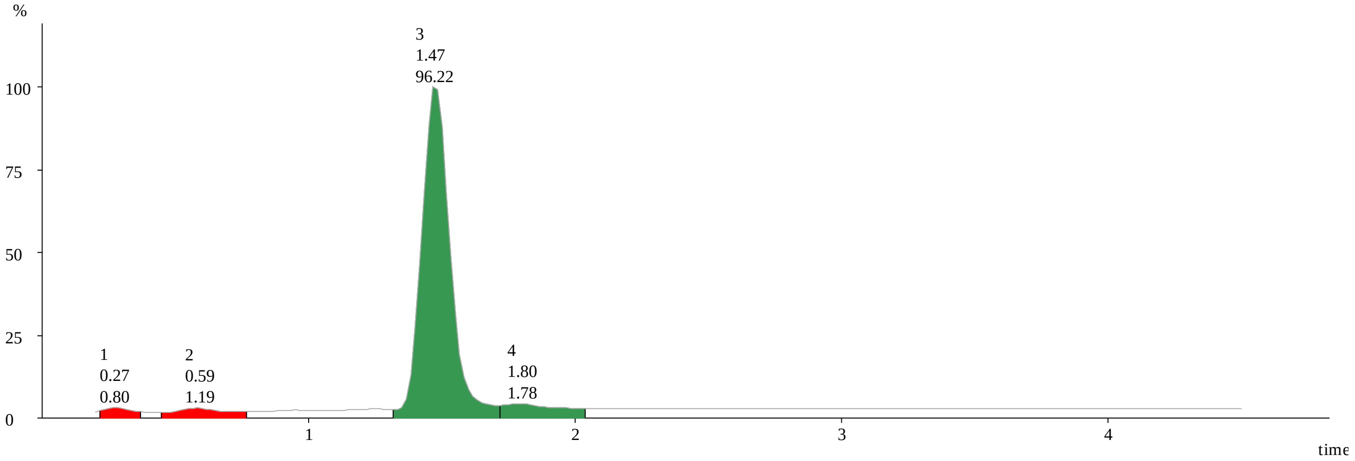

Peak_ID	Peak	Area	Area%	Height	Time	Mass Found
1	0.22 0.37	2.E3	0.8	2.E4	0.27	
2	0.45 0.77	3.E3	1.19	2.E4	0.59	
3	1.32 1.72	2.E5	96.22	2.E6	1.47	462.06
4	1.72 2.04	4.E3	1.78	3.E4	1.80	462.06

MS ES+ :463.06

max. intensity: 3.8E4

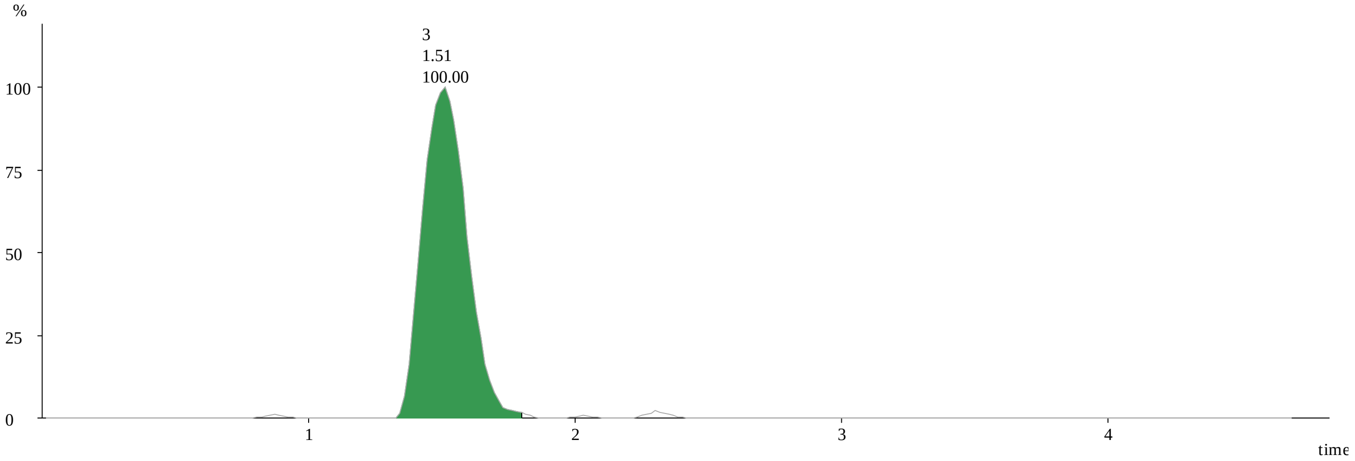

Peak_ID	Peak	Area	Area%	Height	Time	Mass Found
3	1.33 1.80	7.E3	100	4.E4	1.51	462.06

MS ES+ :TIC

max. intensity: 1.2E5

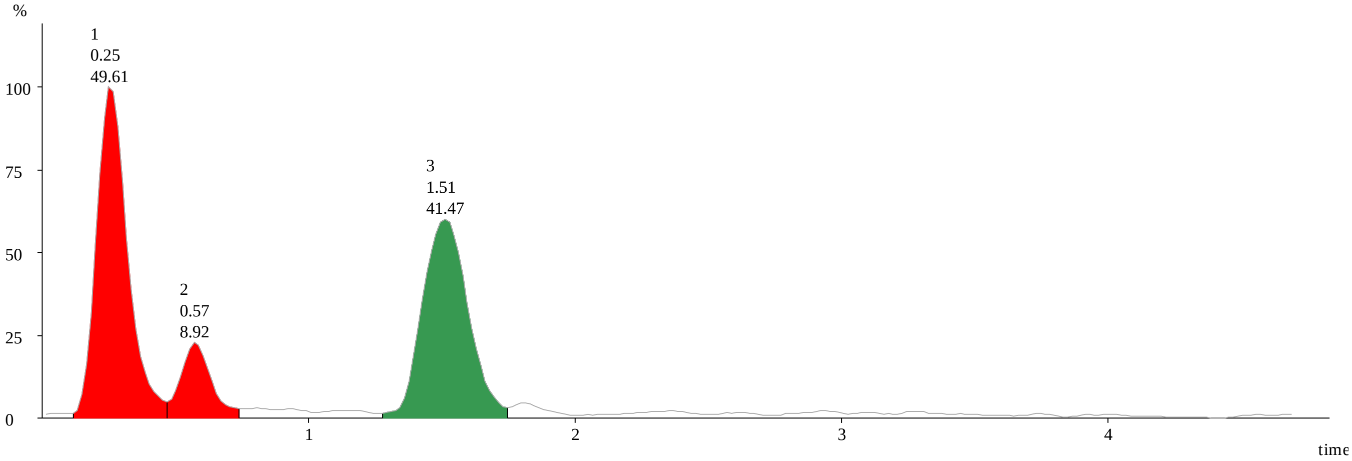

Peak_ID	Peak	Area	Area%	Height	Time	Mass Found
1	0.12 0.47	2.E4	49.61	1.E5	0.25	
2	0.47 0.74	3.E3	8.92	2.E4	0.57	
3	1.28 1.75	1.E4	41.47	7.E4	1.51	462.06

MS: ES+

Combine (33:35-(24:26+46:48))

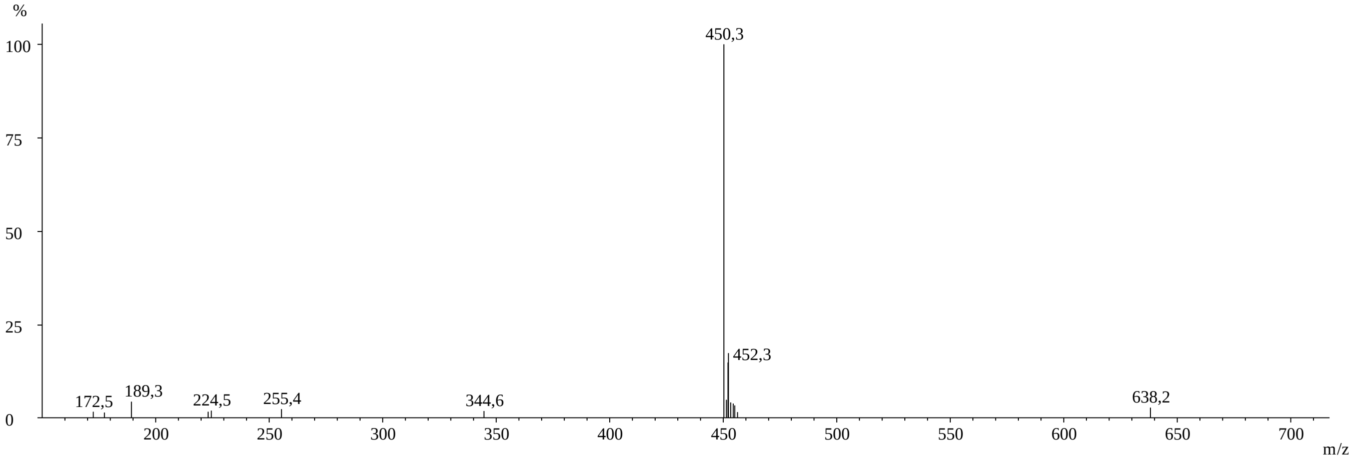

Peak_ID	Compound	Time	Mass found
2		0.57	

Sample: 128
File: 9575_28
Vial: D/4

Date: 26-Aug-2004
Time: 13:34:48
Description: 1006167

AMRI code: ALB-H05990348
Vial label: M5988802AMP0098

MS: ES+

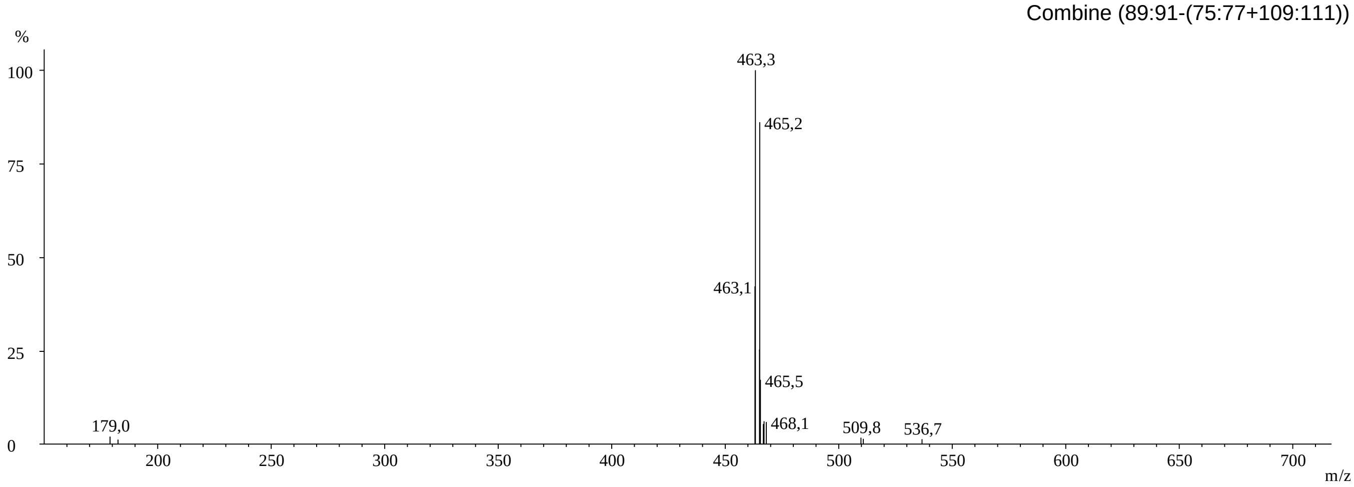

Peak_ID	Compound	Time	Mass found
3	Found	1.51	462.0600

MS: ES+

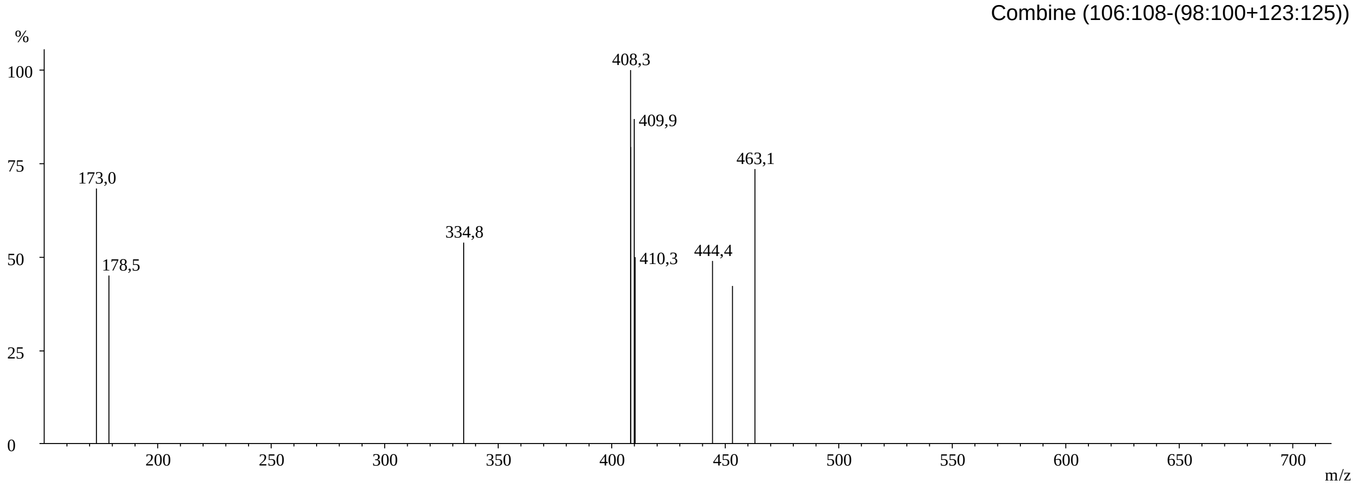

Peak_ID	Compound	Time	Mass found
4	Found	1.80	462.0600
