# Supplementary material for: AI is a viable alternative to high throughput screening: a 318-target study
Source: Sci Rep. 2024 Apr 2;14:7526. doi: 10.1038/s41598-024-54655-z (PMC10987645; doi:10.1038/s41598-024-54655-z)
Supplement: Supplementary file 1 — Supplementary Information 1. [file 41598_2024_54655_MOESM1_ESM.zip › Nature SREP/QC_AIMS_files/Proj020.pdf]

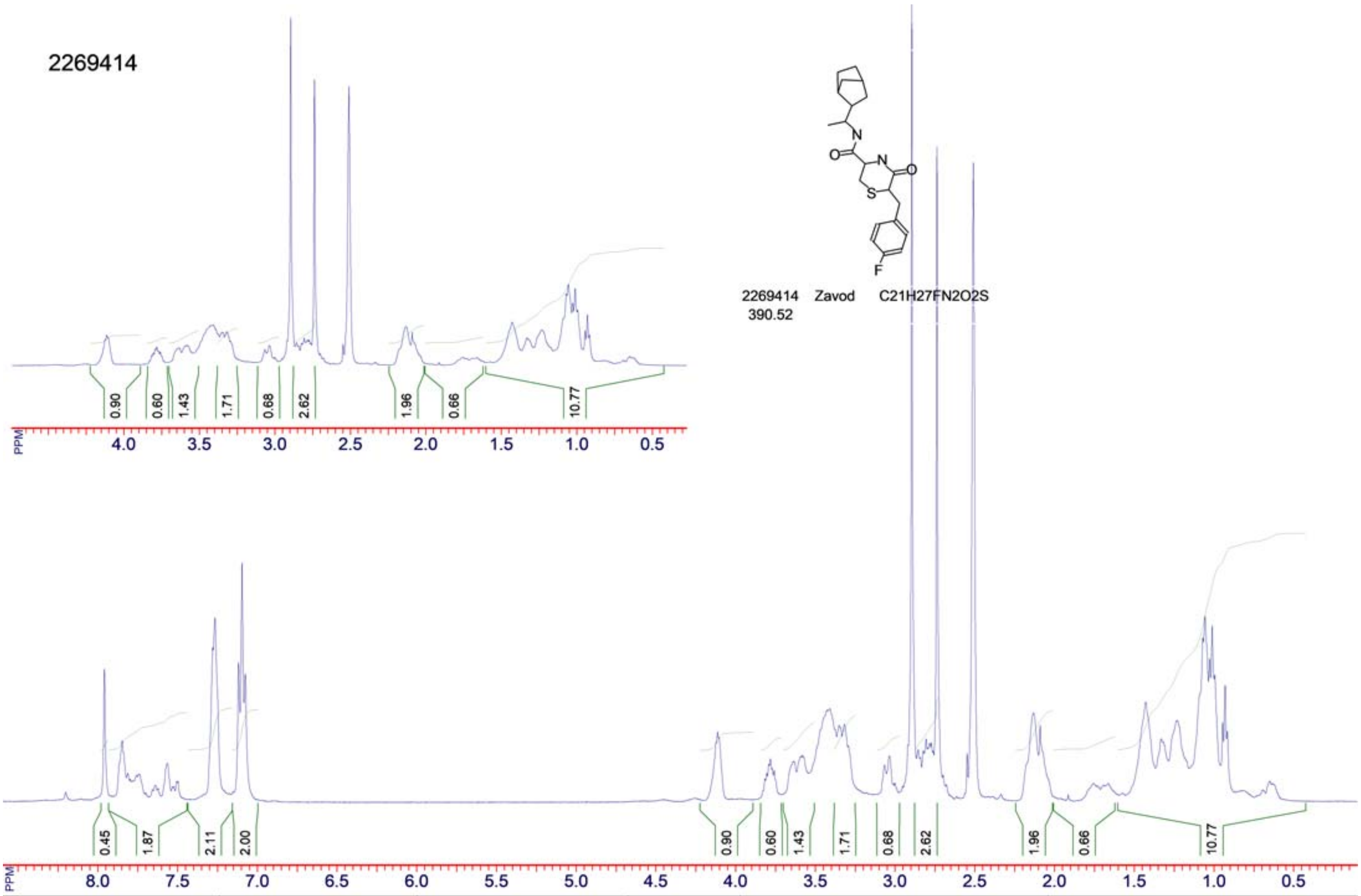

**File name: 2269414**	Vaskivska	SF: 399.9703 MHz	NSC: 0	PW: 15.00 usec, RG: 32	SI: 65536
Date: 22-Jun-2009	Solvent: dmso-d6	SW: 6803 Hz	TE: 293 K	AQ: 1.33 sec, RD: 0.00 sec	
