# Supplementary material for: AI is a viable alternative to high throughput screening: a 318-target study
Source: Sci Rep. 2024 Apr 2;14:7526. doi: 10.1038/s41598-024-54655-z (PMC10987645; doi:10.1038/s41598-024-54655-z)
Supplement: Supplementary file 1 — Supplementary Information 1. [file 41598_2024_54655_MOESM1_ESM.zip › Nature SREP/QC_AIMS_files/Proj025.pdf]

-.o.-Syntez Purity Report -.o.-

Agilent 1100 LC/MSD SL
Diodearrey G1315B (DAD1A-215nm; DAD1B-254nm)
Mass Quad G1956B (MSD1-Pos, MSD2-Neg)
ELSD Altech 3300 (ADC1 A, ELSD)

Mobile fase:A-H2O+0.1%HCOOH;B-MeOH+0.1%HCOOH
Separation column:
Rapid Resolutionn HT Cartige 4.6x30mm,
1.8-Micron, Zorbx SB-C18

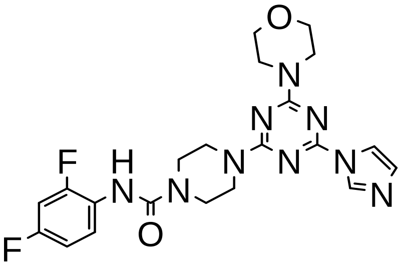

Mol.Weight: 471,47

F5601-0899

M4303 ->

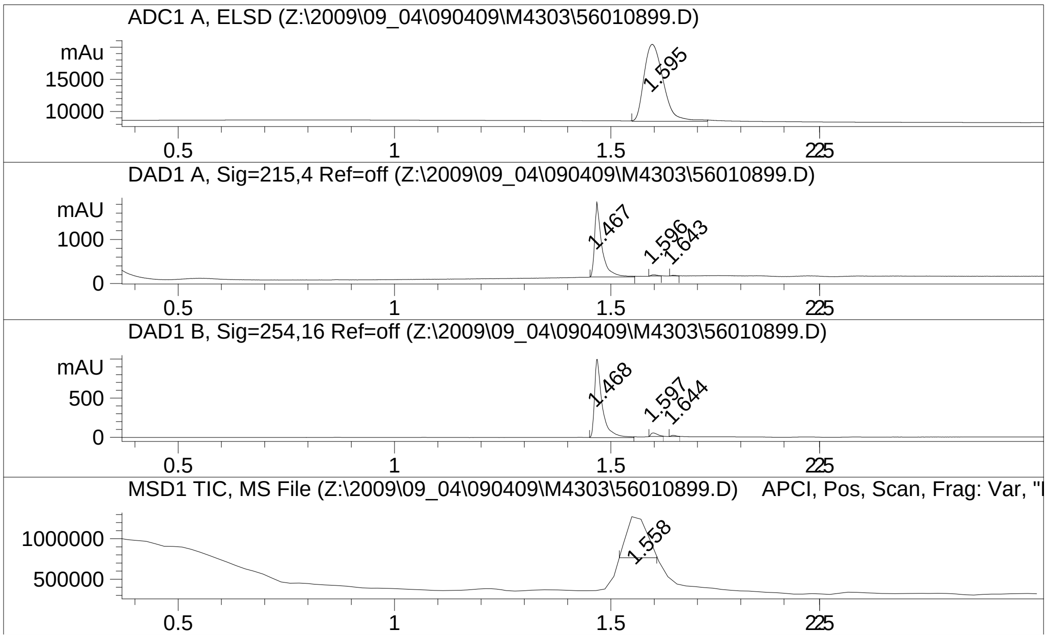

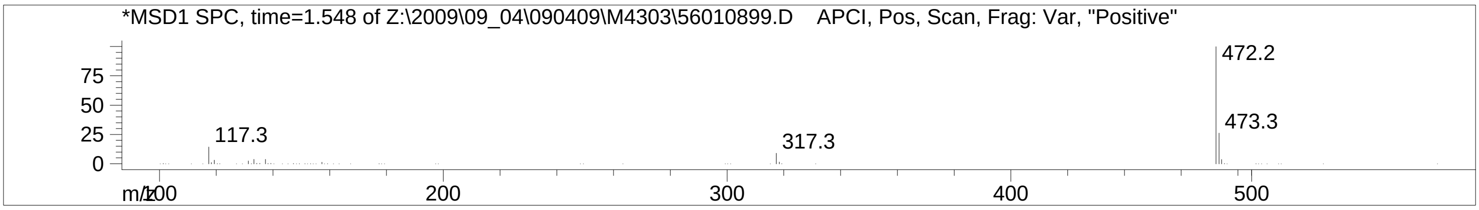

#	Signal	R.Time	Area %
1	ADC1 A, ELSD	1.595	100.000

#	Signal	R.Time	Area %
1	DAD1 A, Sig=215,4 Ref=off	1.467	97.893
2		1.596	1.485
3		1.643	0.622

#	Signal	R.Time	Area %
1	DAD1 B, Sig=254,16 Ref=off	1.468	94.987
2		1.597	3.949
3		1.644	1.064

#	Signal	R.Time	Area %
1	MSD1 TIC, MS File	1.558	100.000
