# Supplementary material for: AI is a viable alternative to high throughput screening: a 318-target study
Source: Sci Rep. 2024 Apr 2;14:7526. doi: 10.1038/s41598-024-54655-z (PMC10987645; doi:10.1038/s41598-024-54655-z)
Supplement: Supplementary file 1 — Supplementary Information 1. [file 41598_2024_54655_MOESM1_ESM.zip › Nature SREP/QC_AIMS_files/Proj032.PDF]

GC101163047

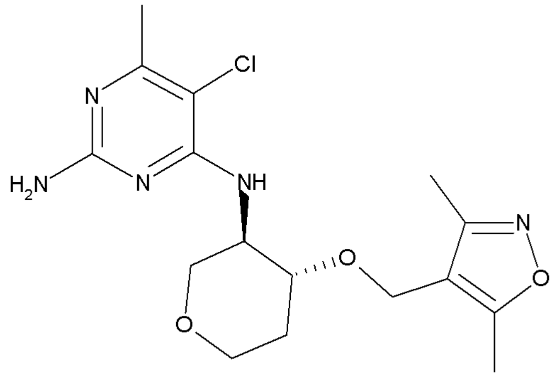

ID	35040238	367.8384	$C_{16}H_{22}ClN_5O_3$

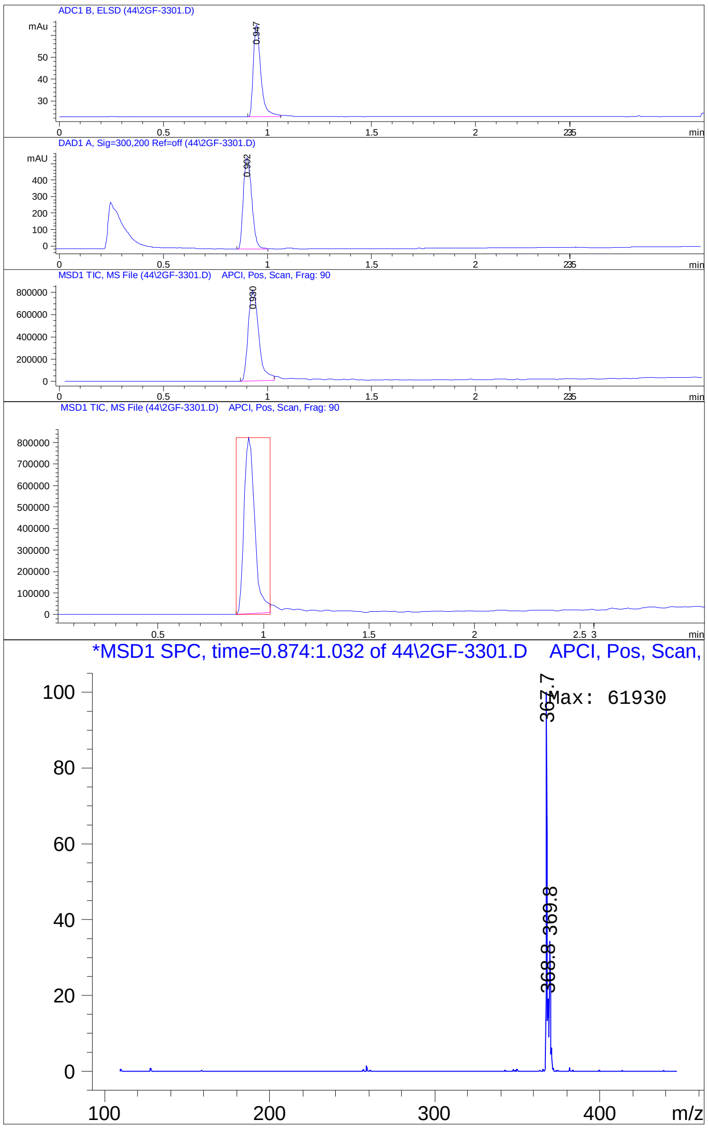

Data File D:\DATA\44\2GF-3301.D
Sample Name: GC1011630P2-G-06
Instrument 1 21/07/2014 18:30:31 №6
Column: Onyx C18 50x4.6mm | 3.75ml/min | Columns Reg Valve
Gradient: "c"->@2.4min->"D"(Hold 0.5min)->@0.2min->"C"->PostRun
PMP1, Solvent A : 0.1%TFA in Acn/H2O (2.5:97.5)
PMP1, Solvent B : 0.1%TFA in AcN
PMP1, Solvent C : 0.1%FA in Acn/H2O (2.5:97.5)
PMP1, Solvent D : 0.1%FA in AcN
Ionization mode : APCI Positive

Signal 1: ADC1 B, ELSD

Peak #	RetTime [min]	Type	Width [min]	Area [mAu*s]	Height [mAu]	Area %
1	0.947	PB	0.0383	105.00047	41.85051	100.0000
Totals :				105.00047	41.85051	

Signal 2: DAD1 A, Sig=300,200 Ref=off

Peak #	RetTime [min]	Type	Width [min]	Area [mAU*s]	Height [mAU]	Area %
1	0.902	PB	0.0449	1564.08911	554.75366	100.0000
Totals :				1564.08911	554.75366	

Signal 3: MSD1 TIC, MS File

Peak #	RetTime [min]	Type	Width [min]	Area	Height	Area %
1	0.930	PB	0.0548	2.86194e6	8.18230e5	100.0000
Totals :				2.86194e6	8.18230e5	
