# Supplementary material for: AI is a viable alternative to high throughput screening: a 318-target study
Source: Sci Rep. 2024 Apr 2;14:7526. doi: 10.1038/s41598-024-54655-z (PMC10987645; doi:10.1038/s41598-024-54655-z)
Supplement: Supplementary file 1 — Supplementary Information 1. [file 41598_2024_54655_MOESM1_ESM.zip › Nature SREP/QC_AIMS_files/Proj036.pdf]

U7566

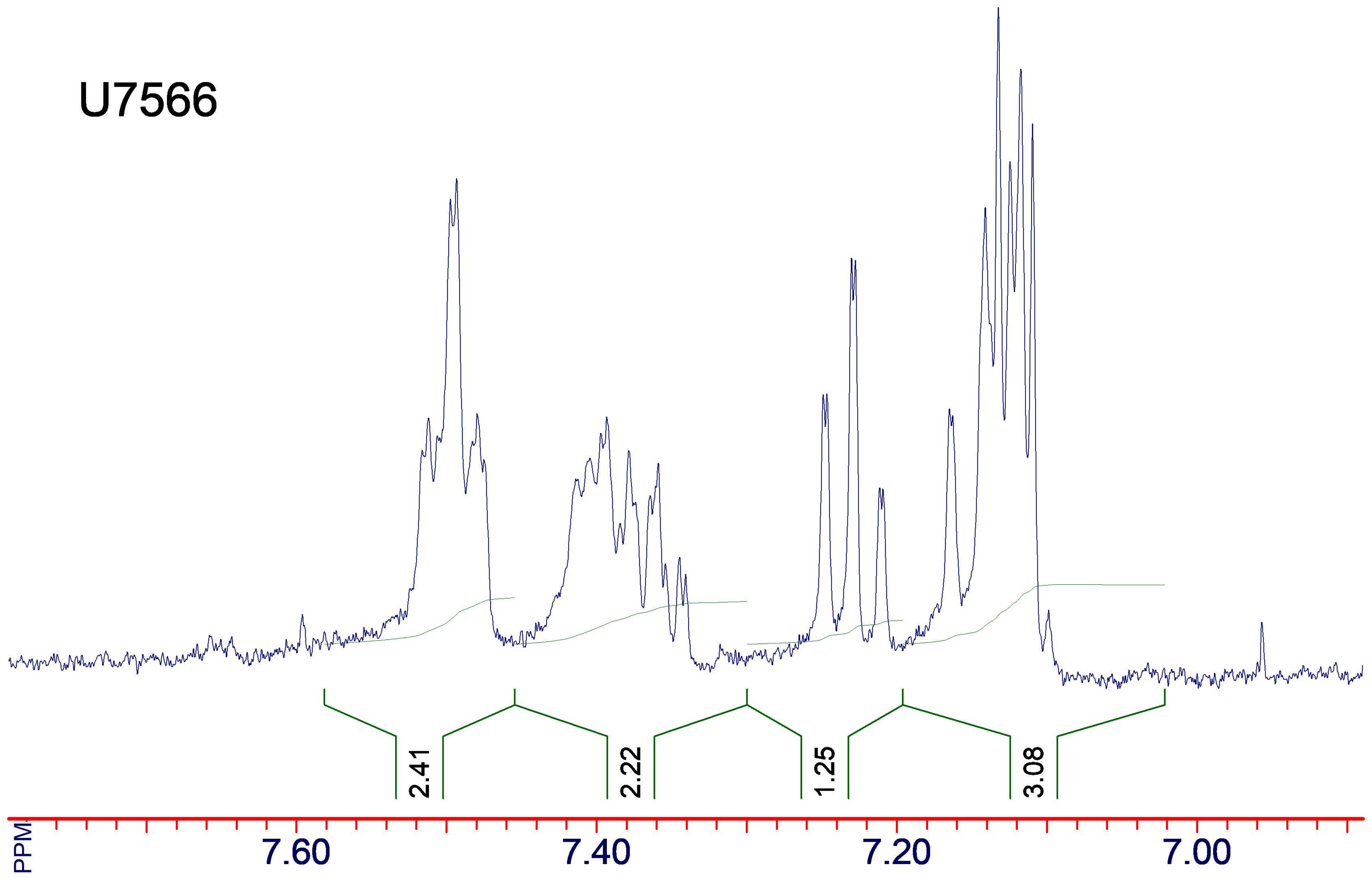

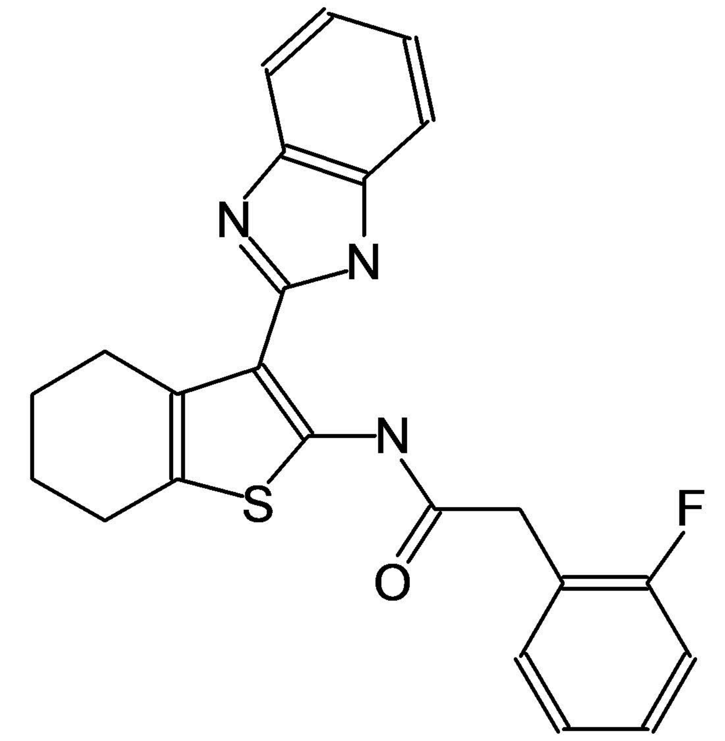

U7566 $C_{23}H_{20}FN_3OS$ 405.49

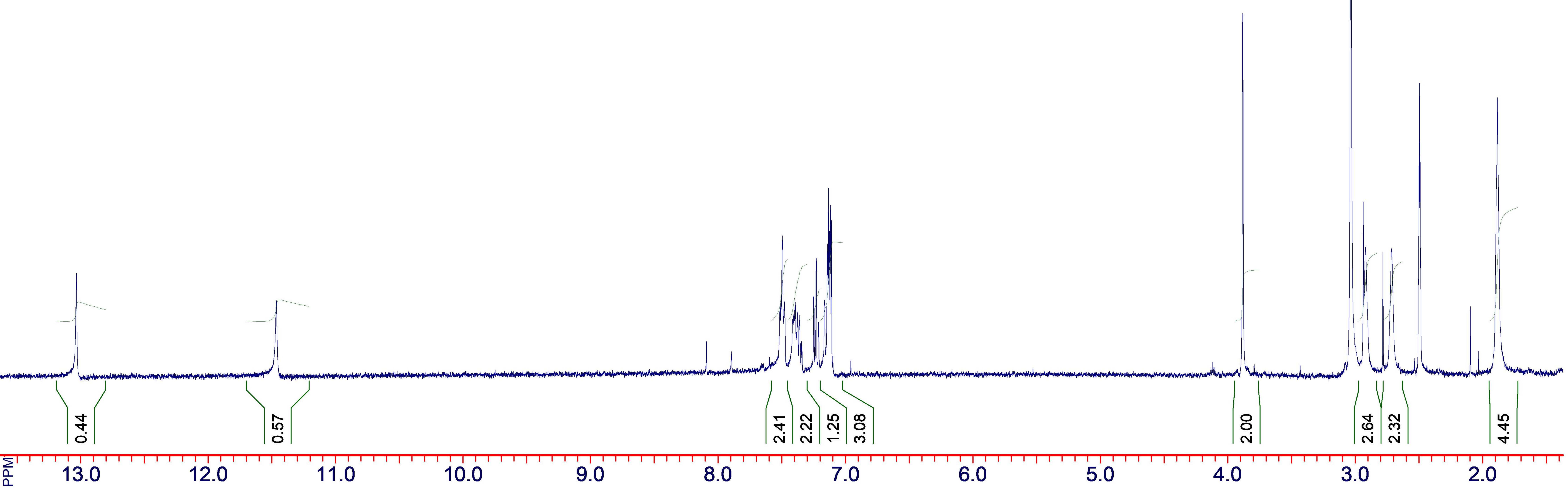

**File name:U7566**	Vladimirova	SF: 400.3960 MHz	NSC: 0	PW: 9.00 usec, RG: 24	SI: 65536
Date: 20-Jan-2004	Solvent: DMSO-d6 + CCl4	SW: 8000 Hz	TE: 293 K	AQ: 2.00 sec, RD: 0.00 sec	
