# Supplementary material for: AI is a viable alternative to high throughput screening: a 318-target study
Source: Sci Rep. 2024 Apr 2;14:7526. doi: 10.1038/s41598-024-54655-z (PMC10987645; doi:10.1038/s41598-024-54655-z)
Supplement: Supplementary file 1 — Supplementary Information 1. [file 41598_2024_54655_MOESM1_ESM.zip › Nature SREP/QC_AIMS_files/Proj040.pdf]

MaxPeak: 94.95%
Ret_Time: 1.051 min

# 6965831

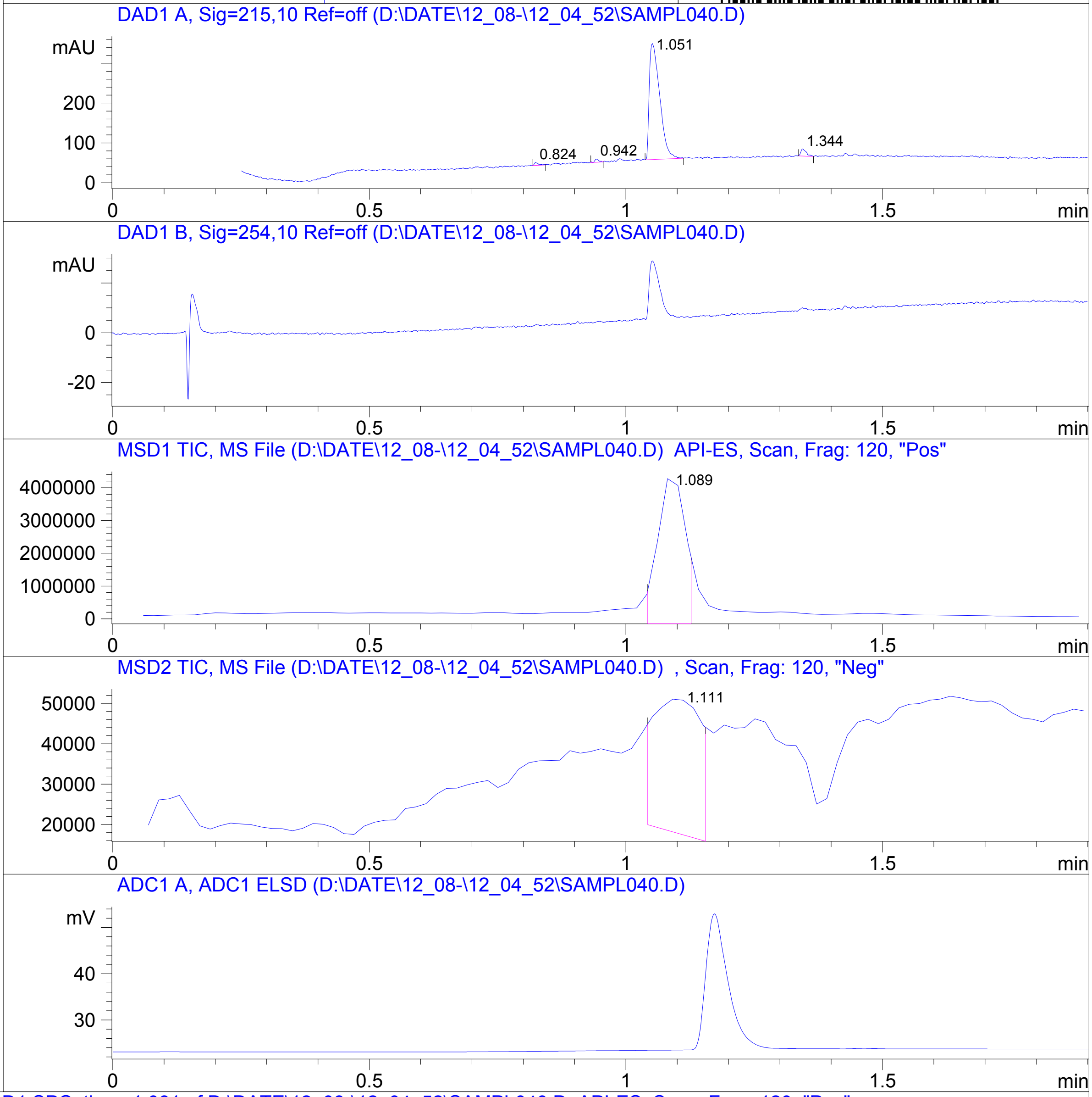


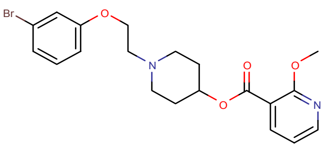


**Mol Wt** 435.31
**Exact Mass** 434.11

| # | Time | Area% |
|---|---|---|
| 1 | 0.824 | 1.00 |
| 2 | 0.942 | 1.00 |
| 3 | 1.051 | 94.95 |
| 4 | 1.344 | 3.04 |

RT 1.089

RT 1.111

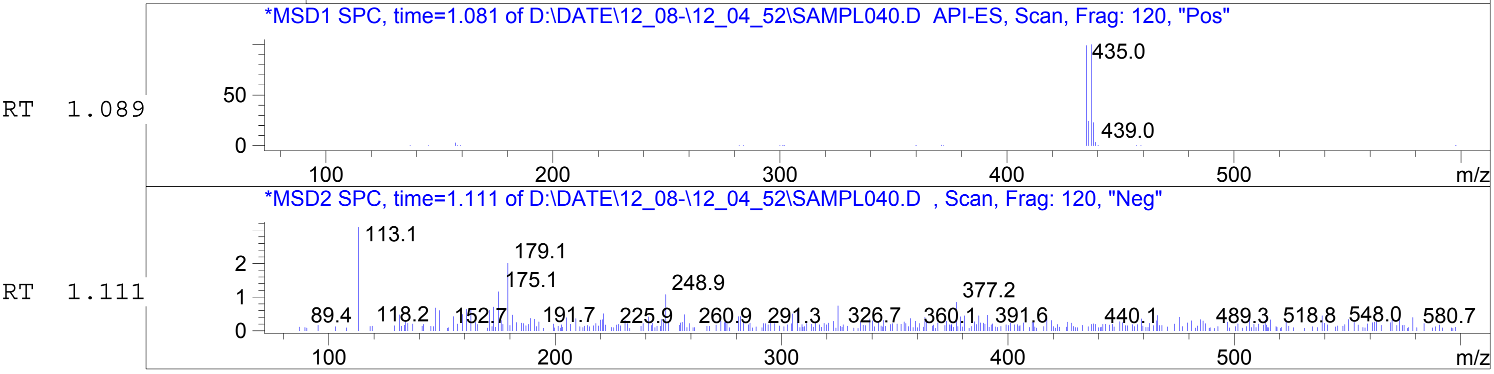


Inj.Date 12/8/2014 L P2-E-07 VL Acq. Method C:\HPCHEM\-> ->
