# Supplementary material for: AI is a viable alternative to high throughput screening: a 318-target study
Source: Sci Rep. 2024 Apr 2;14:7526. doi: 10.1038/s41598-024-54655-z (PMC10987645; doi:10.1038/s41598-024-54655-z)
Supplement: Supplementary file 1 — Supplementary Information 1. [file 41598_2024_54655_MOESM1_ESM.zip › Nature SREP/QC_AIMS_files/Proj041.pdf]

MaxPeak: 90.40%
Ret_Time: 1.369 min

6399894

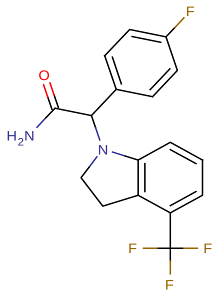

Mol Wt 338.3
Exact Mass 338.13

#	Time	Area%
1	1.160	3.25
2	1.301	2.79
3	1.369	90.40
4	1.601	3.56

DAD1 A, Sig=215,16 Ref=off (E:\WORK\D\09\09_19\09_19_28\SAMPL008.D)

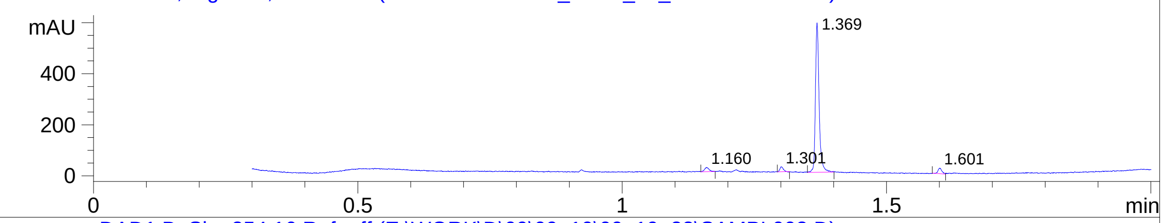

DAD1 B, Sig=254,16 Ref=off (E:\WORK\D\09\09_19\09_19_28\SAMPL008.D)

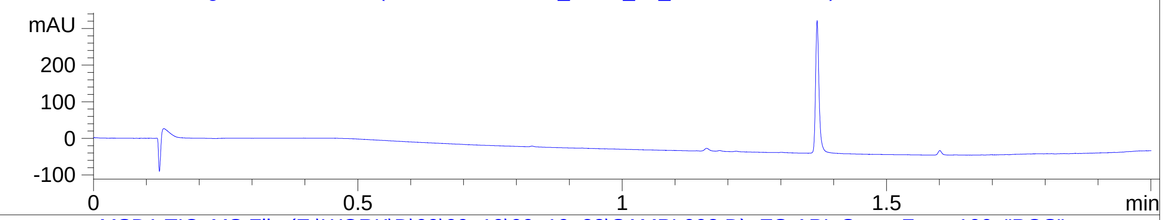

MSD1 TIC, MS File (E:\WORK\D\09\09_19\09_19_28\SAMPL008.D) ES-API, Scan, Frag: 100, "POS"

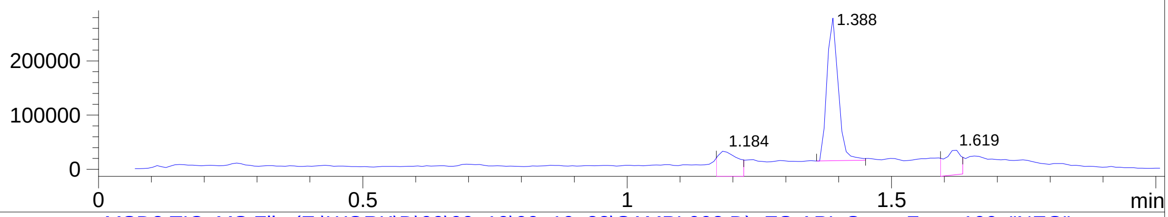

MSD2 TIC, MS File (E:\WORK\D\09\09_19\09_19_28\SAMPL008.D) ES-API, Scan, Frag: 100, "NEG"

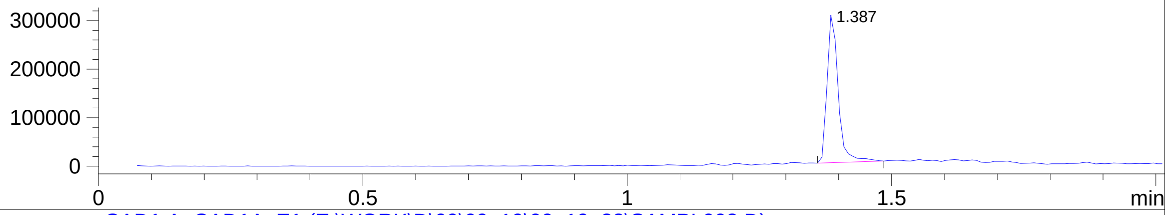

CAD1 A, CAD1A, E1 (E:\WORK\D\09\09_19\09_19_28\SAMPL008.D)

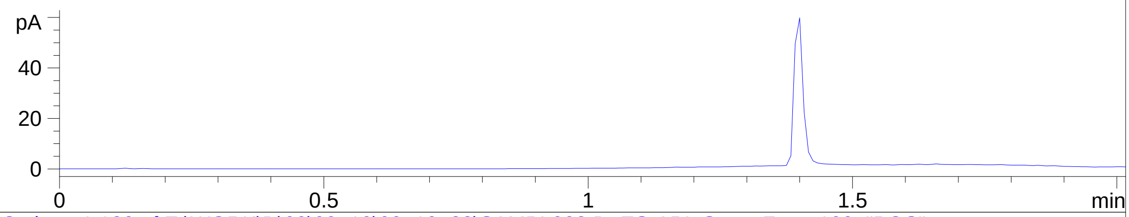

*MSD1 SPC, time=1.180 of E:\WORK\D\09\09_19\09_19_28\SAMPL008.D ES-API, Scan, Frag: 100, "POS"

RT 1.184

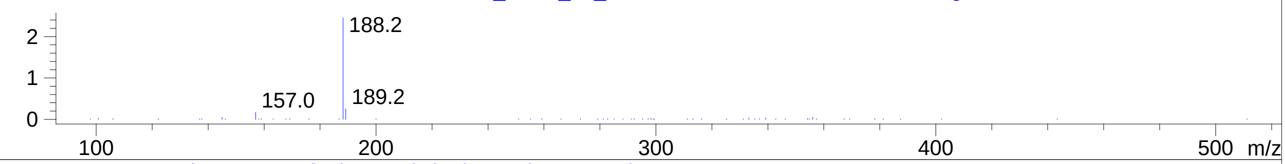

*MSD1 SPC, time=1.389 of E:\WORK\D\09\09_19\09_19_28\SAMPL008.D ES-API, Scan, Frag: 100, "POS"

RT 1.388

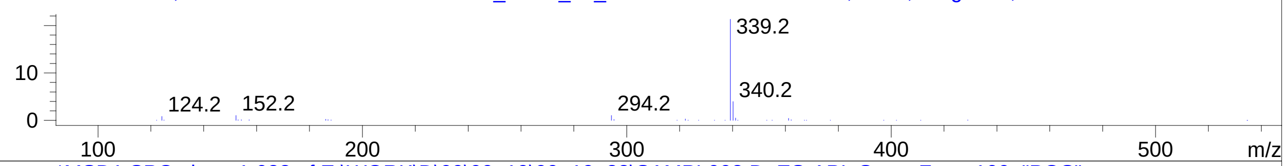

*MSD1 SPC, time=1.623 of E:\WORK\D\09\09_19\09_19_28\SAMPL008.D ES-API, Scan, Frag: 100, "POS"

RT 1.619

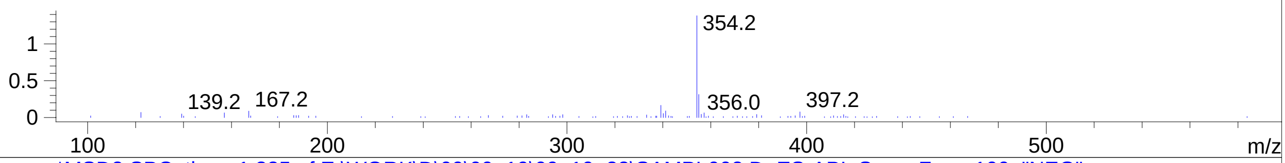

*MSD2 SPC, time=1.385 of E:\WORK\D\09\09_19\09_19_28\SAMPL008.D ES-API, Scan, Frag: 100, "NEG"

RT 1.387

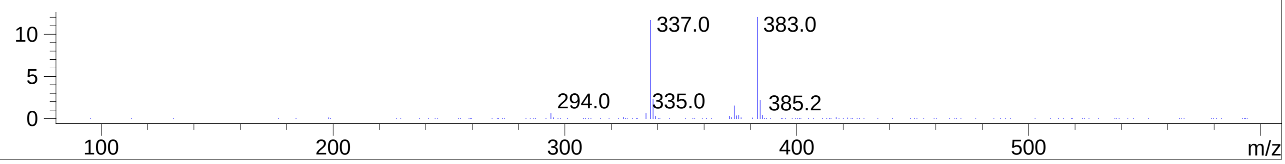

Inj.Date 9/19/2013 K P1-B-03 '4' Acq. Method C:\CHEM32\-> ->
