# Supplementary material for: AI is a viable alternative to high throughput screening: a 318-target study
Source: Sci Rep. 2024 Apr 2;14:7526. doi: 10.1038/s41598-024-54655-z (PMC10987645; doi:10.1038/s41598-024-54655-z)
Supplement: Supplementary file 1 — Supplementary Information 1. [file 41598_2024_54655_MOESM1_ESM.zip › Nature SREP/QC_AIMS_files/Proj044.pdf]

MaxPeak: 100.00%
Ret_Time: 0.829 min

9585275$1

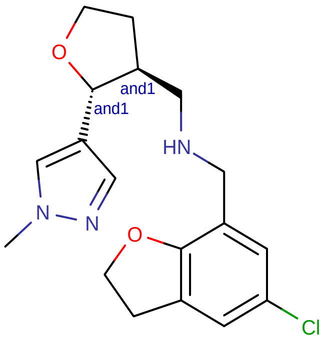

Mol Wt 347.84

Exact Mass 347.17

#	Time	Area%
1	0.829	100.00

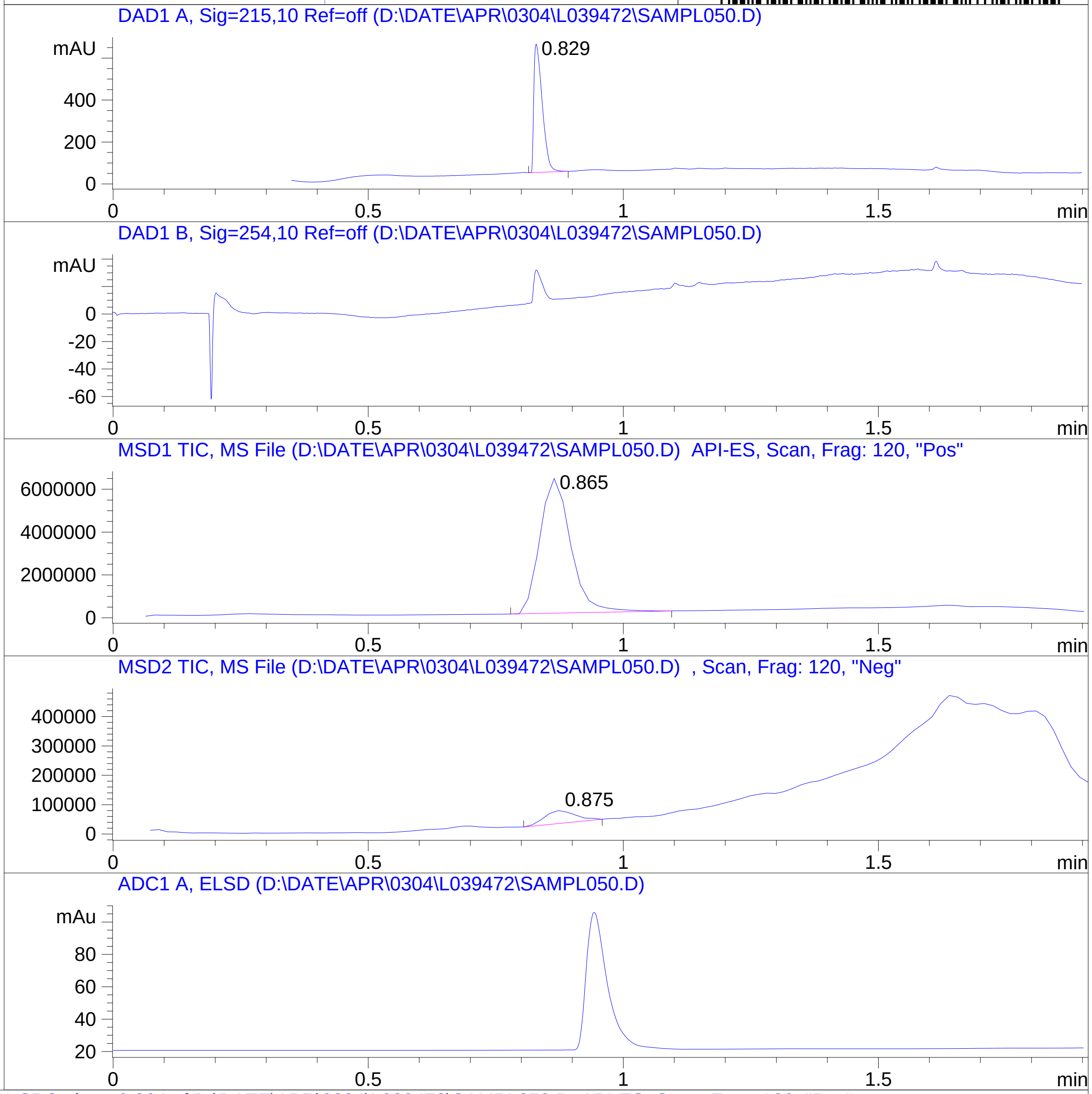

RT 0.865

RT 0.875

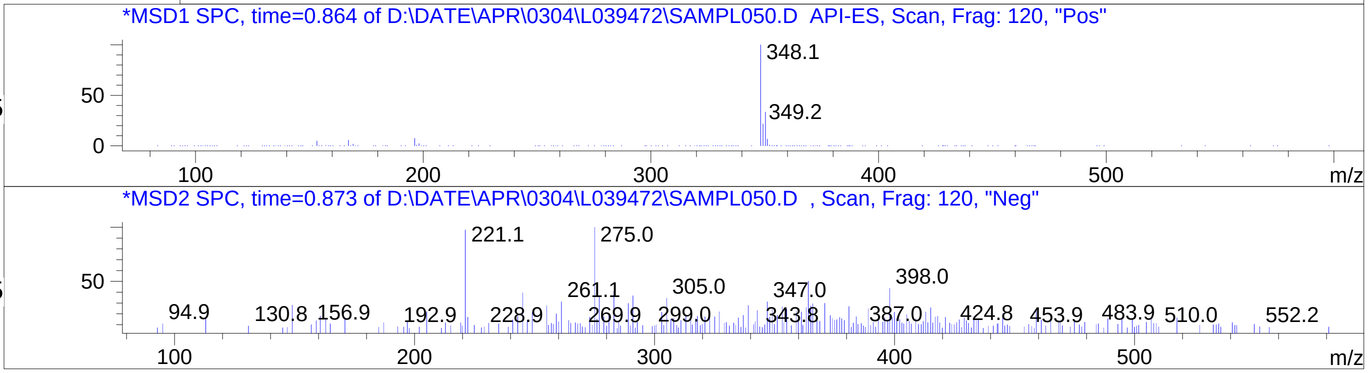

Inj.Date 4/3/2017 A P2-F-06 -SL- Acq. Method C:\HPCHEM\-> ->
