# Supplementary material for: AI is a viable alternative to high throughput screening: a 318-target study
Source: Sci Rep. 2024 Apr 2;14:7526. doi: 10.1038/s41598-024-54655-z (PMC10987645; doi:10.1038/s41598-024-54655-z)
Supplement: Supplementary file 1 — Supplementary Information 1. [file 41598_2024_54655_MOESM1_ESM.zip › Nature SREP/QC_AIMS_files/Proj045.pdf]

SHIMADZU LabSolutions

Analysis Report

<Sample Information>

Acquired by : Molnar Ildiko
Date Acquired : 2019. 09. 17. 13:55:31
Sample Name : PR-36081
Sample ID : H11
File Name :MC-QC-271_Gabi_posneg_190916_PR-36081_H11_096.lcd
Method Fiel : MCule_5min_posneg.lcm

<Chromatogram>

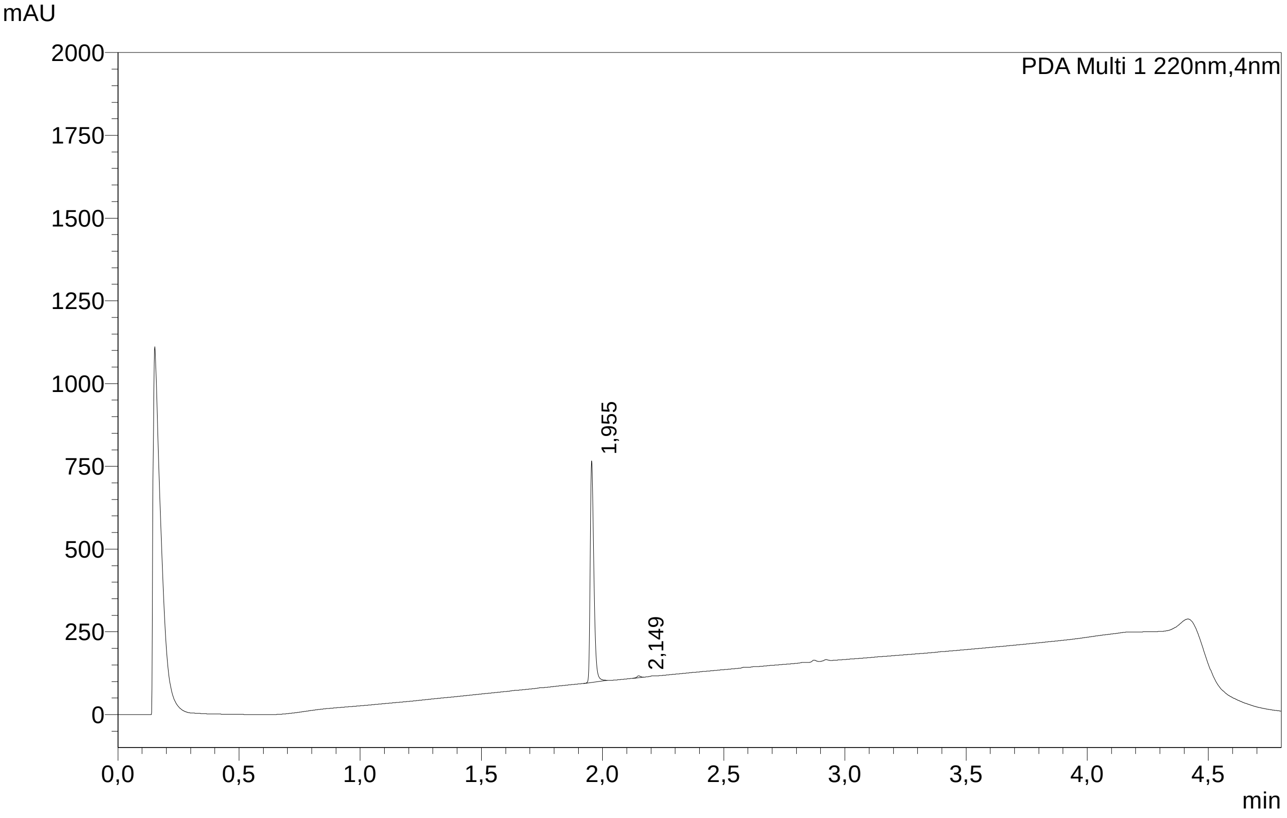

Peak Table

PDA Ch1 220nm

Peak#	Ret. Time	Area	Area%
1	1,955	702971	99
2	2,149	5849	1
Total		708820	100

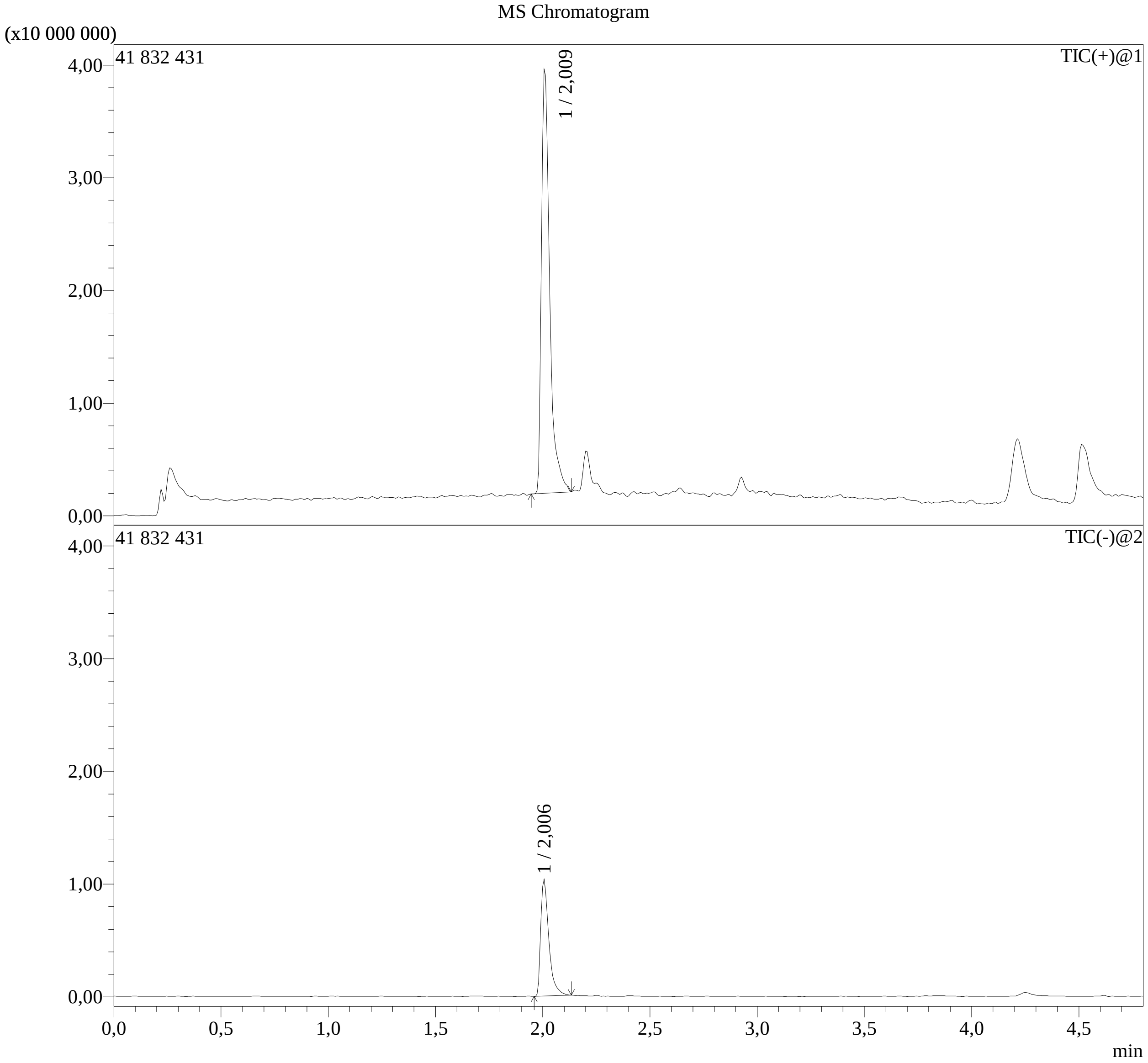

MASS Peak Table TIC

Peak#	Ret. Time	m/z	Area	Base Peak m/z
1	2,009	TIC	100058603	321,0
2	2,006	TIC	27091399	318,9
Total			127150002	

MS Spectrum

Peak#:1 R.Time:2,006(Scan#:602)
MassPeaks:32
Spectrum Mode:Averaged 1,997-2,010(600-604)
BG Mode:Calc Segment 1 - Event 2

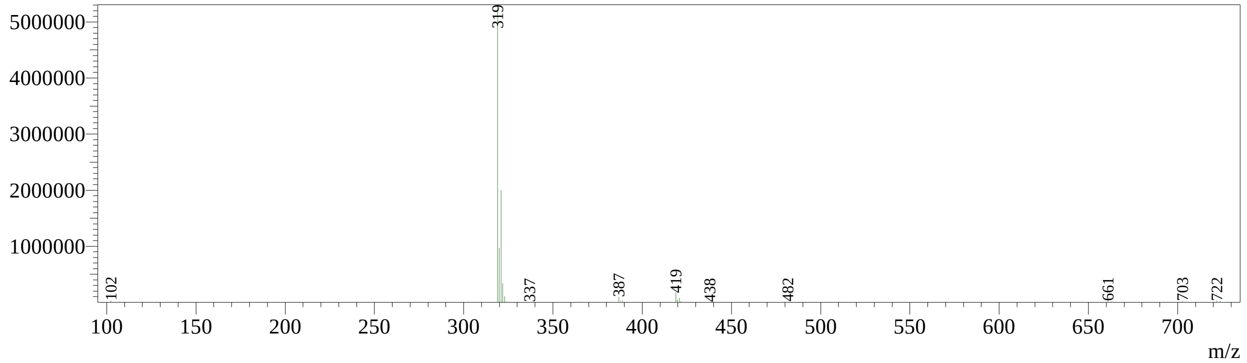

Peak#:1 R.Time:2,009(Scan#:603)
MassPeaks:95
Spectrum Mode:Averaged 2,000-2,013(601-605)
BG Mode:Calc Segment 1 - Event 1

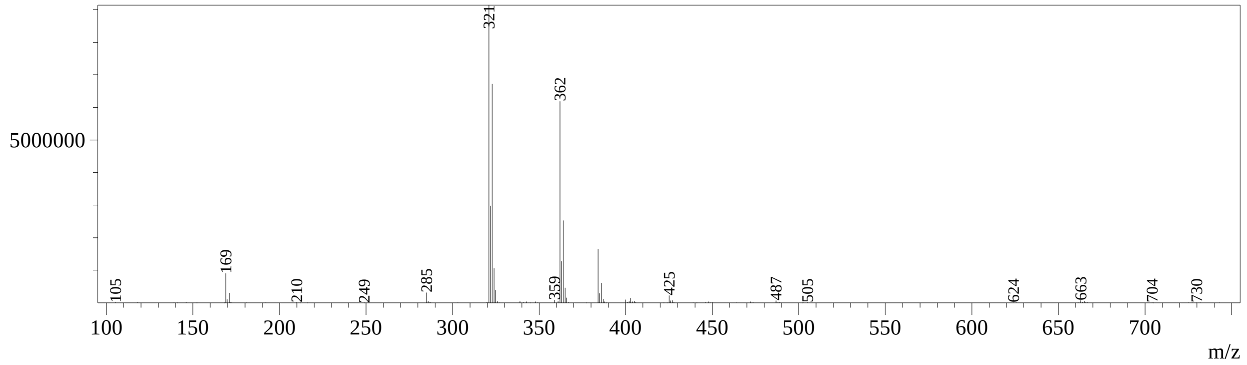

MS Spectrum
