# Supplementary material for: AI is a viable alternative to high throughput screening: a 318-target study
Source: Sci Rep. 2024 Apr 2;14:7526. doi: 10.1038/s41598-024-54655-z (PMC10987645; doi:10.1038/s41598-024-54655-z)
Supplement: Supplementary file 1 — Supplementary Information 1. [file 41598_2024_54655_MOESM1_ESM.zip › Nature SREP/QC_AIMS_files/Proj046.pdf]

SHIMADZU LabSolutions

Analysis Report

<Sample Information>

Acquired by : Steindl Kristof
Date Acquired : 2018. 11. 29. 0:43:40
Sample Name : PR-15114
Sample ID : C12
File Name :MC-QC-69_Gabi_posneg_181128_PR-15114_C12_038.lcd
Method Fiel : MCule_5min_posneg.lcm

<Chromatogram>

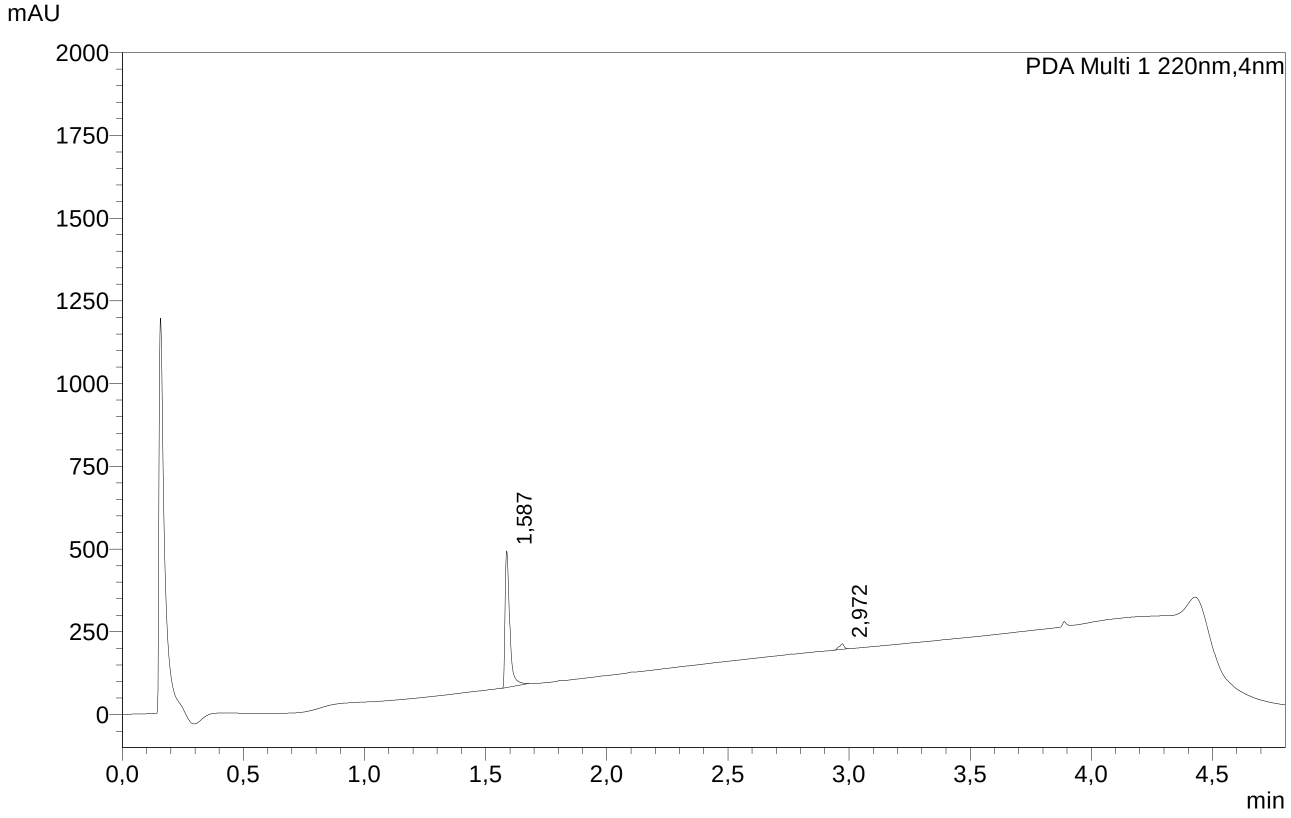

Peak Table

PDA Ch1 220nm

Peak#	Ret. Time	Area	Area%
1	1,587	542834	96
2	2,972	24335	4
Total		567168	100

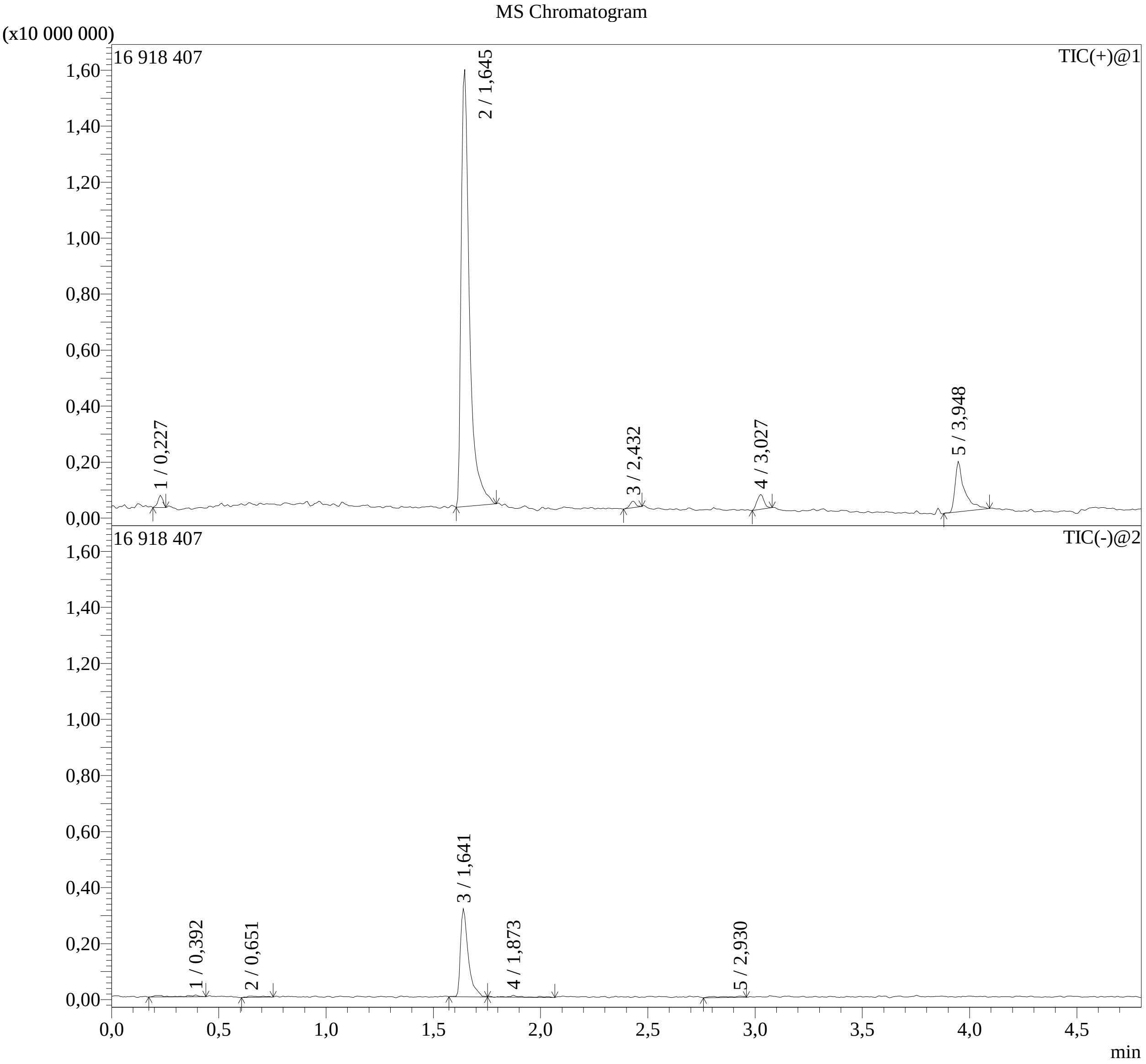

MASS Peak Table TIC

Peak#	Ret. Time	m/z	Area	Base Peak m/z
1	0,227	TIC	703908	214,0
2	1,645	TIC	41919296	310,5
3	2,432	TIC	462673	326,9
4	3,027	TIC	1213218	279,1
5	3,948	TIC	5503624	391,2
6	0,392	TIC	359074	244,0
7	0,651	TIC	239317	108,8
8	1,641	TIC	7818210	618,1
9	1,873	TIC	288264	101,2
10	2,930	TIC	329802	101,7
Total			58837385	

MS Spectrum

Peak#:1 R.Time:0,227(Scan#:69)
MassPeaks:21
Spectrum Mode:Averaged 0,220-0,233(67-71)
BG Mode:Calc Segment 1 - Event 1

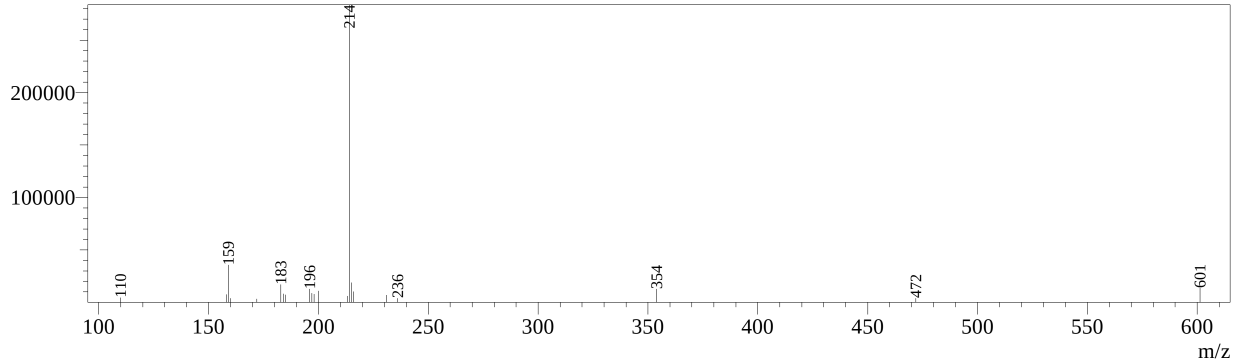

Peak#:1 R.Time:0,392(Scan#:118)
MassPeaks:8
Spectrum Mode:Averaged 0,383-0,397(116-120)
BG Mode:Calc Segment 1 - Event 2

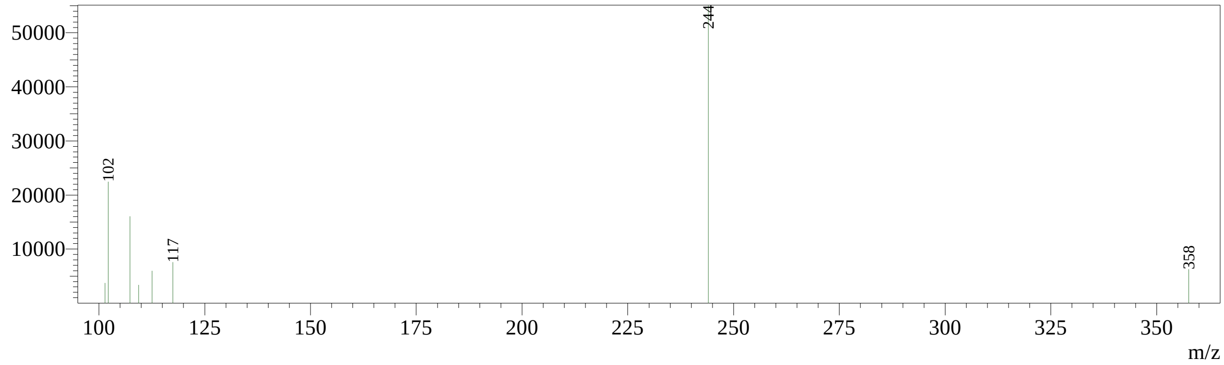

Peak#:2 R.Time:0,651(Scan#:196)
MassPeaks:9
Spectrum Mode:Averaged 0,643-0,657(194-198)
BG Mode:Calc Segment 1 - Event 2

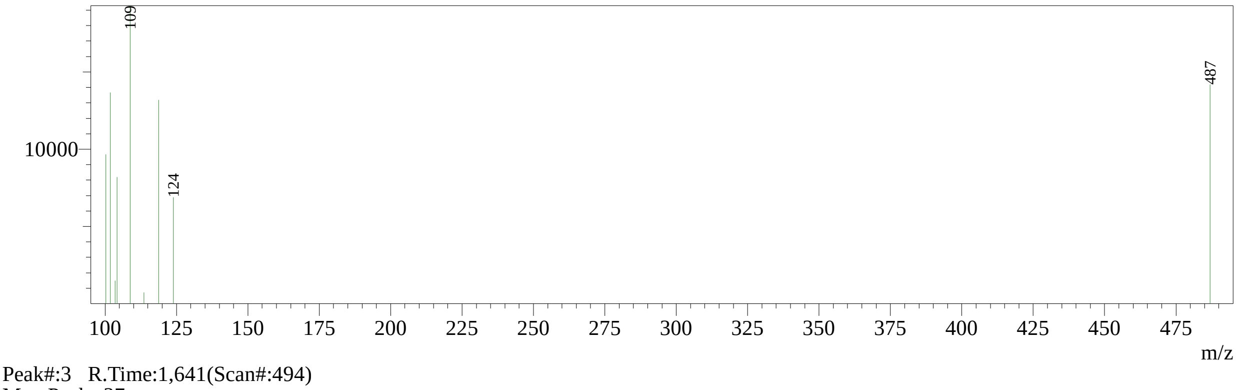

Peak#:3 R.Time:1,641(Scan#:494)
MassPeaks:37
Spectrum Mode:Averaged 1,637-1,650(492-496)
BG Mode:Calc Segment 1 - Event 2

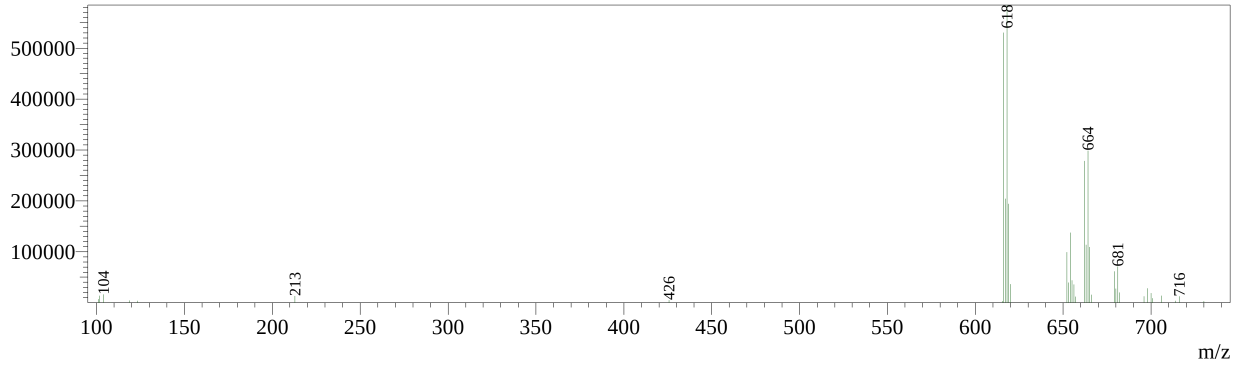

Peak#:2 R.Time:1,645(Scan#:493)
MassPeaks:34
Spectrum Mode:Averaged 1,633-1,647(491-495)
BG Mode:Calc Segment 1 - Event 1

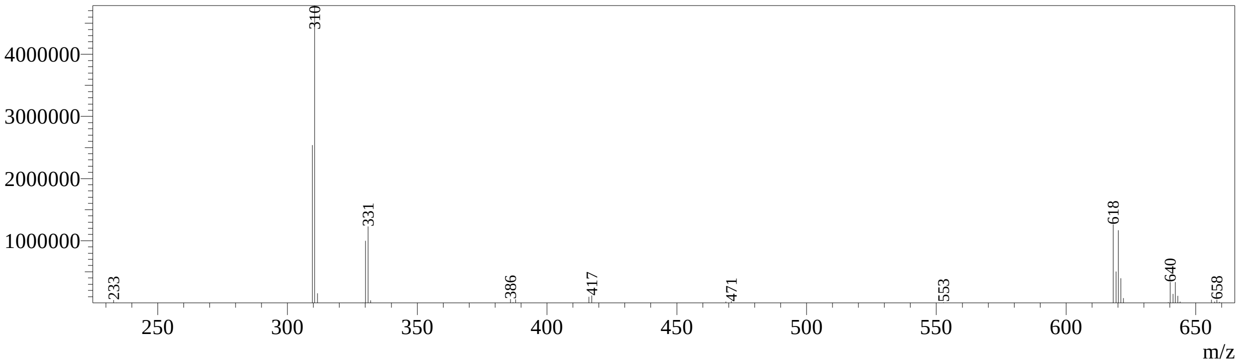

Peak#:4 R.Time:1,873(Scan#:564)
MassPeaks:7
Spectrum Mode:Averaged 1,870-1,883(562-566)
BG Mode:Calc Segment 1 - Event 2

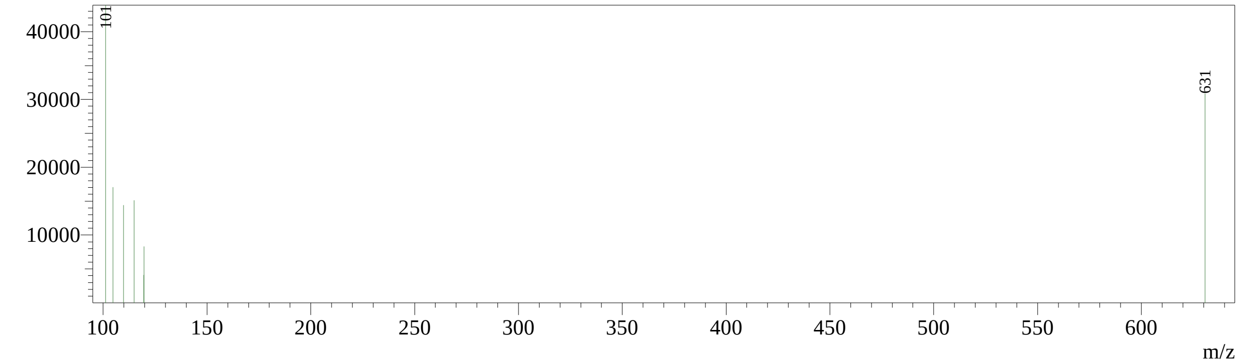

Peak#:3 R.Time:2,432(Scan#:729)
MassPeaks:8
Spectrum Mode:Averaged 2,420-2,433(727-731)
BG Mode:Calc Segment 1 - Event 1

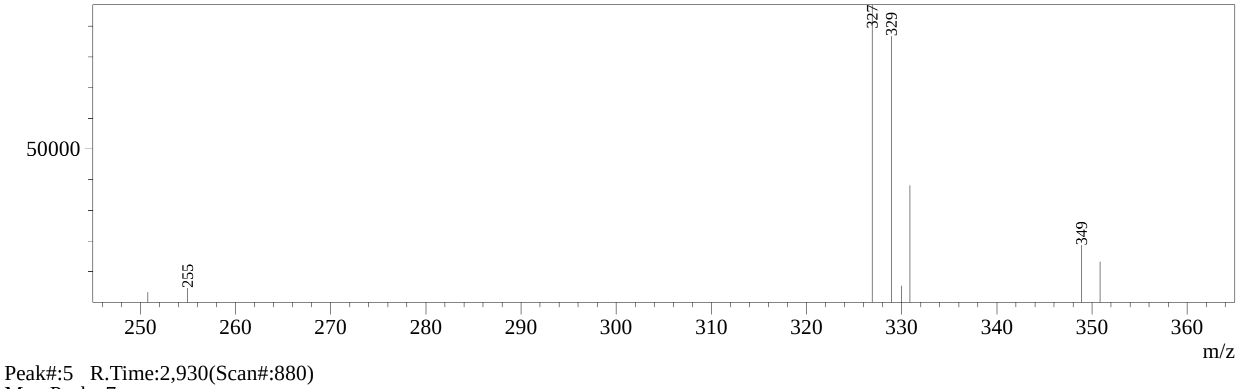

Peak#:5 R.Time:2,930(Scan#:880)
MassPeaks:7
Spectrum Mode:Averaged 2,923-2,937(878-882)
BG Mode:Calc Segment 1 - Event 2

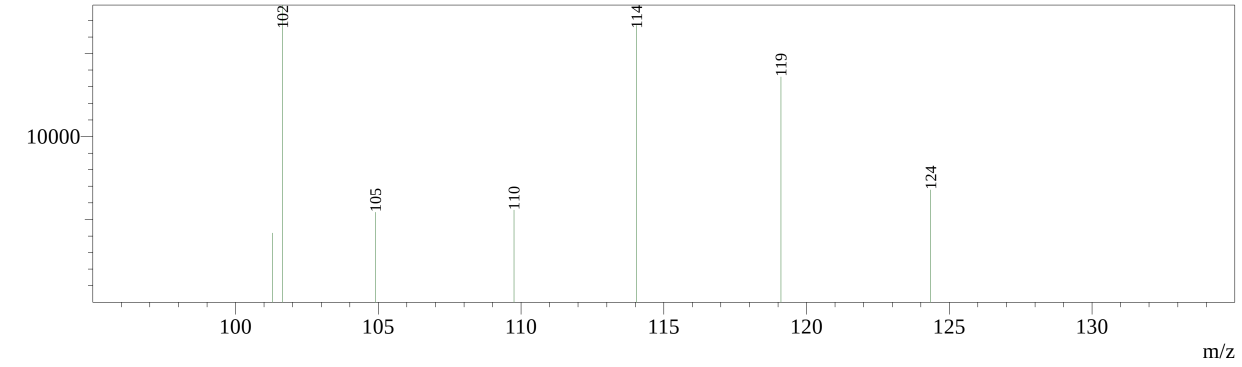

Peak#:4 R.Time:3,027(Scan#:907)
MassPeaks:11
Spectrum Mode:Averaged 3,013-3,027(905-909)
BG Mode:Calc Segment 1 - Event 1

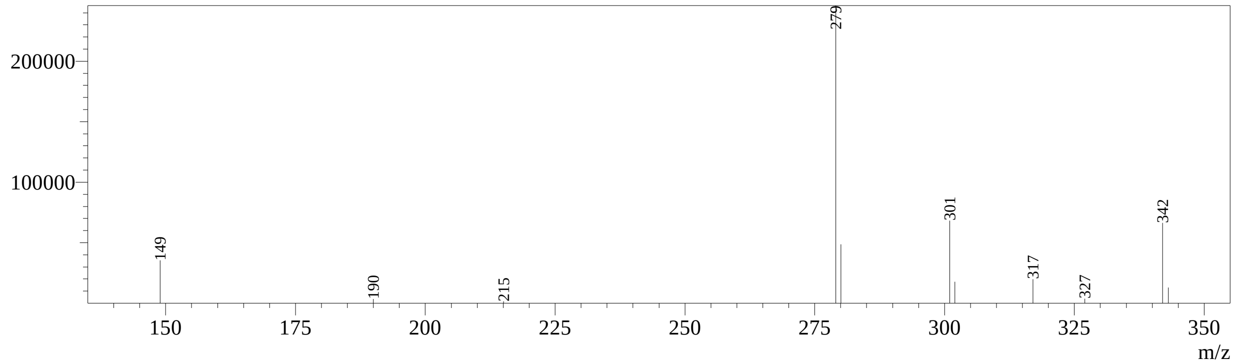

Peak#:5 R.Time:3,948(Scan#:1185)
MassPeaks:21
Spectrum Mode:Averaged 3,940-3,953(1183-1187)
BG Mode:Calc Segment 1 - Event 1

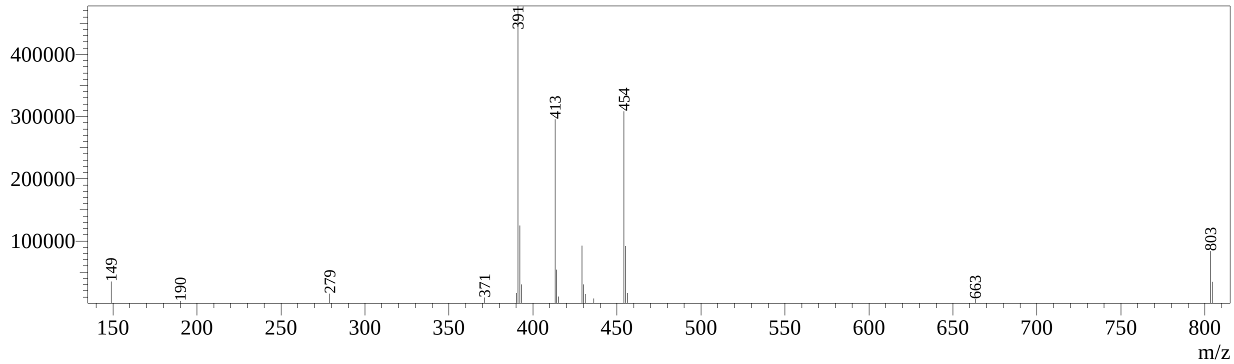

MS Spectrum
