# Supplementary material for: AI is a viable alternative to high throughput screening: a 318-target study
Source: Sci Rep. 2024 Apr 2;14:7526. doi: 10.1038/s41598-024-54655-z (PMC10987645; doi:10.1038/s41598-024-54655-z)
Supplement: Supplementary file 1 — Supplementary Information 1. [file 41598_2024_54655_MOESM1_ESM.zip › Nature SREP/QC_AIMS_files/Proj049.pdf]

SHIMADZU LabSolutions

Analysis Report

<Sample Information>

Acquired by : Molnar Ildiko
Date Acquired : 2019. 08. 26. 11:04:32
Sample Name : PR-34934
Sample ID : A05
File Name :MC-QC-250_Gabi_posneg_190826_PR-34934_A05_006.lcd
Method Fiel : MCule_5min_posneg.lcm

<Chromatogram>

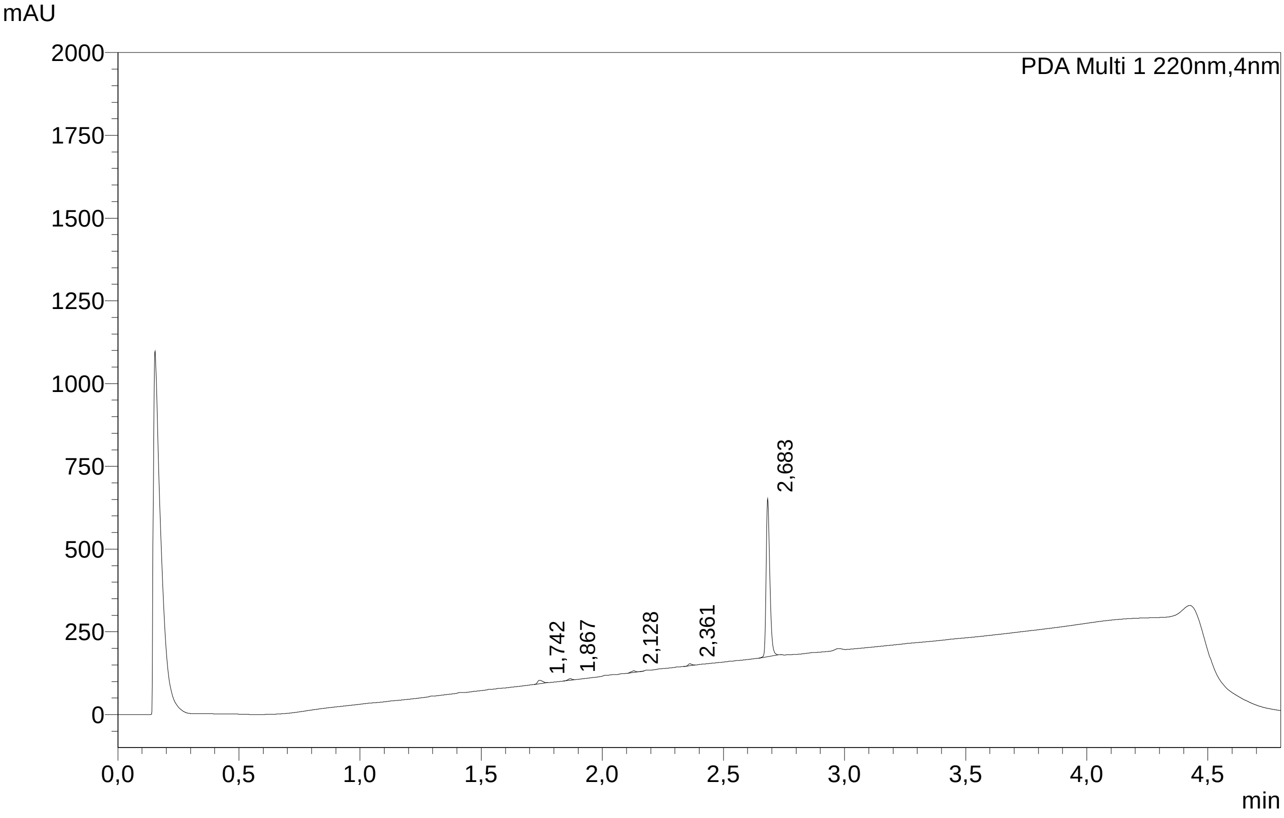

Peak Table

PDA Ch1 220nm

Peak#	Ret. Time	Area	Area%
1	1,742	14878	3
2	1,867	5536	1
3	2,128	6399	1
4	2,361	5842	1
5	2,683	507775	94
Total		540429	100

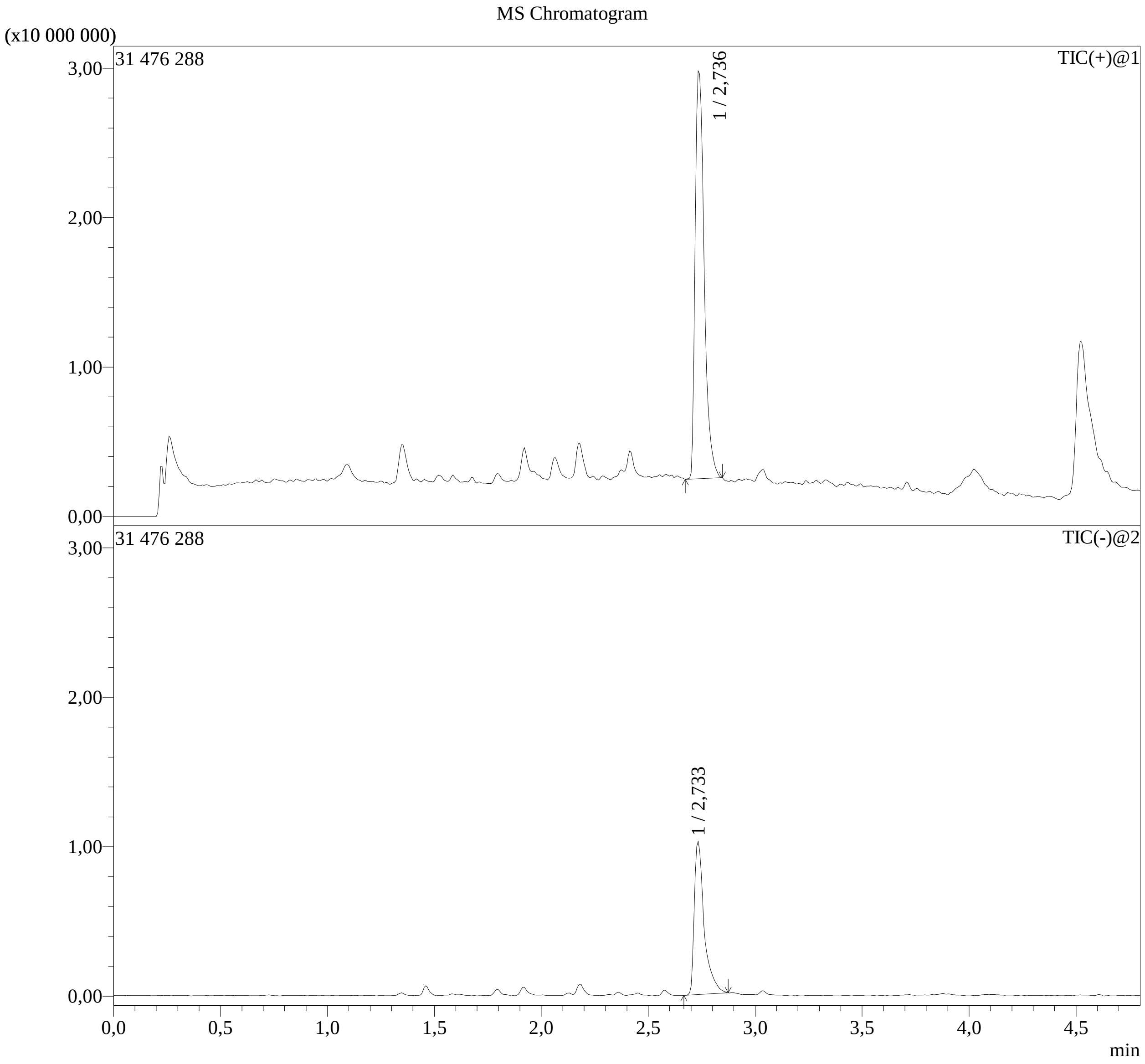

MASS Peak Table TIC

Peak#	Ret. Time	m/z	Area	Base Peak m/z
1	2,736	TIC	80396195	631,1
2	2,733	TIC	32907729	629,0
Total			113303924	

MS Spectrum

Peak#:1 R.Time:2,733(Scan#:820)
MassPeaks:22
Spectrum Mode:Averaged 2,723-2,737(818-822)
BG Mode:Calc Segment 1 - Event 2

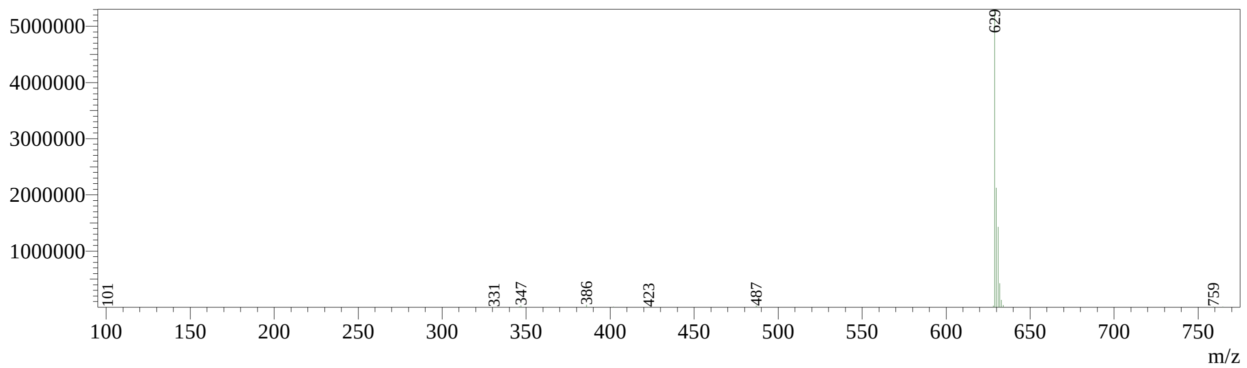

Peak#:1 R.Time:2,736(Scan#:821)
MassPeaks:98
Spectrum Mode:Averaged 2,727-2,740(819-823)
BG Mode:Calc Segment 1 - Event 1

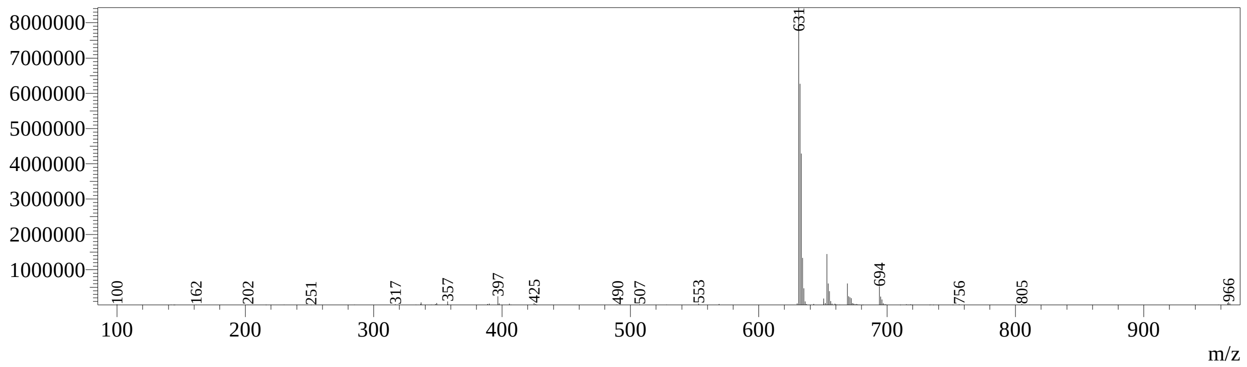

MS Spectrum
