# Supplementary material for: AI is a viable alternative to high throughput screening: a 318-target study
Source: Sci Rep. 2024 Apr 2;14:7526. doi: 10.1038/s41598-024-54655-z (PMC10987645; doi:10.1038/s41598-024-54655-z)
Supplement: Supplementary file 1 — Supplementary Information 1. [file 41598_2024_54655_MOESM1_ESM.zip › Nature SREP/QC_AIMS_files/Proj051.pdf]

MaxPeak: 95.15%
Ret_Time: 0.747 min

5202413

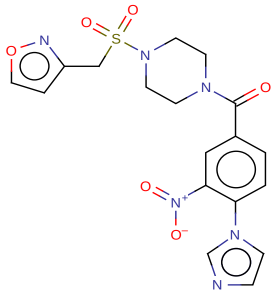

Mol Wt 446.44
Exact Mass 446.09

#	Time	Area%
1	0.592	4.85
2	0.747	95.15

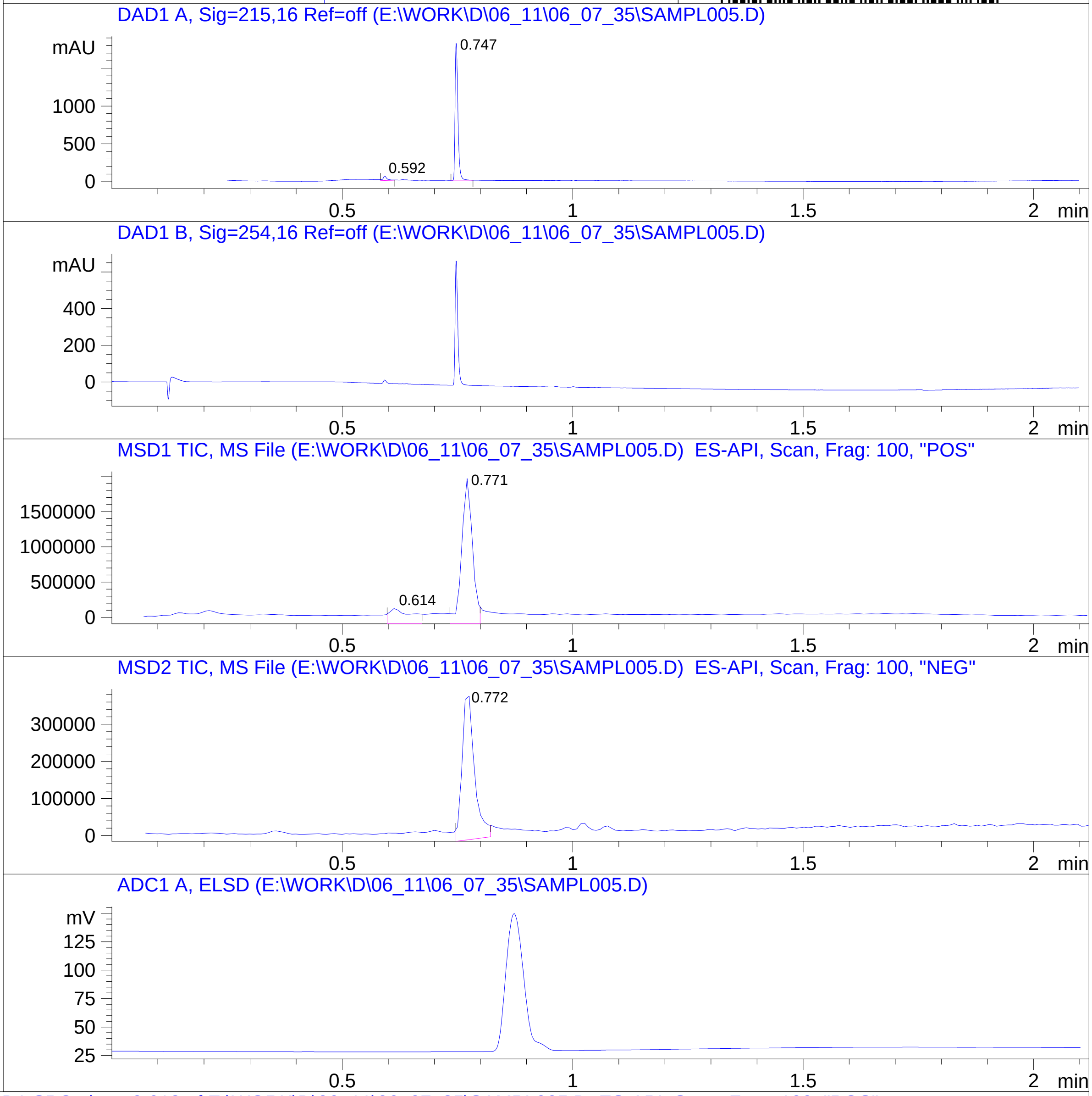

RT 0.614

RT 0.771

RT 0.772

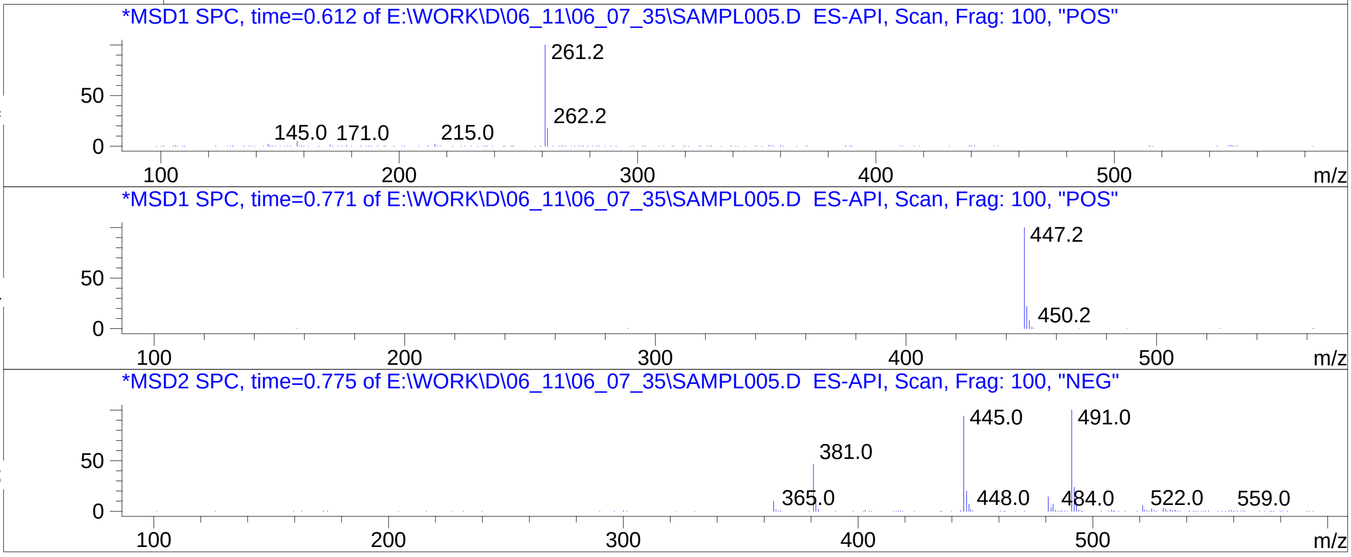

Inj.Date 6/11/2012 K P2-A-05 '4' Acq. Method C:\CHEM32\-> ->
