# Supplementary material for: AI is a viable alternative to high throughput screening: a 318-target study
Source: Sci Rep. 2024 Apr 2;14:7526. doi: 10.1038/s41598-024-54655-z (PMC10987645; doi:10.1038/s41598-024-54655-z)
Supplement: Supplementary file 1 — Supplementary Information 1. [file 41598_2024_54655_MOESM1_ESM.zip › Nature SREP/QC_AIMS_files/Proj063.pdf]

MaxPeak: 97.81%
Ret_Time: 1.181 min

2262259

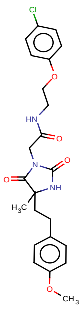

Mol Wt 459.923
Exact Mass 459.18

#	Time	Area%
1	0.828	2.19
2	1.181	97.81

RT 1.202

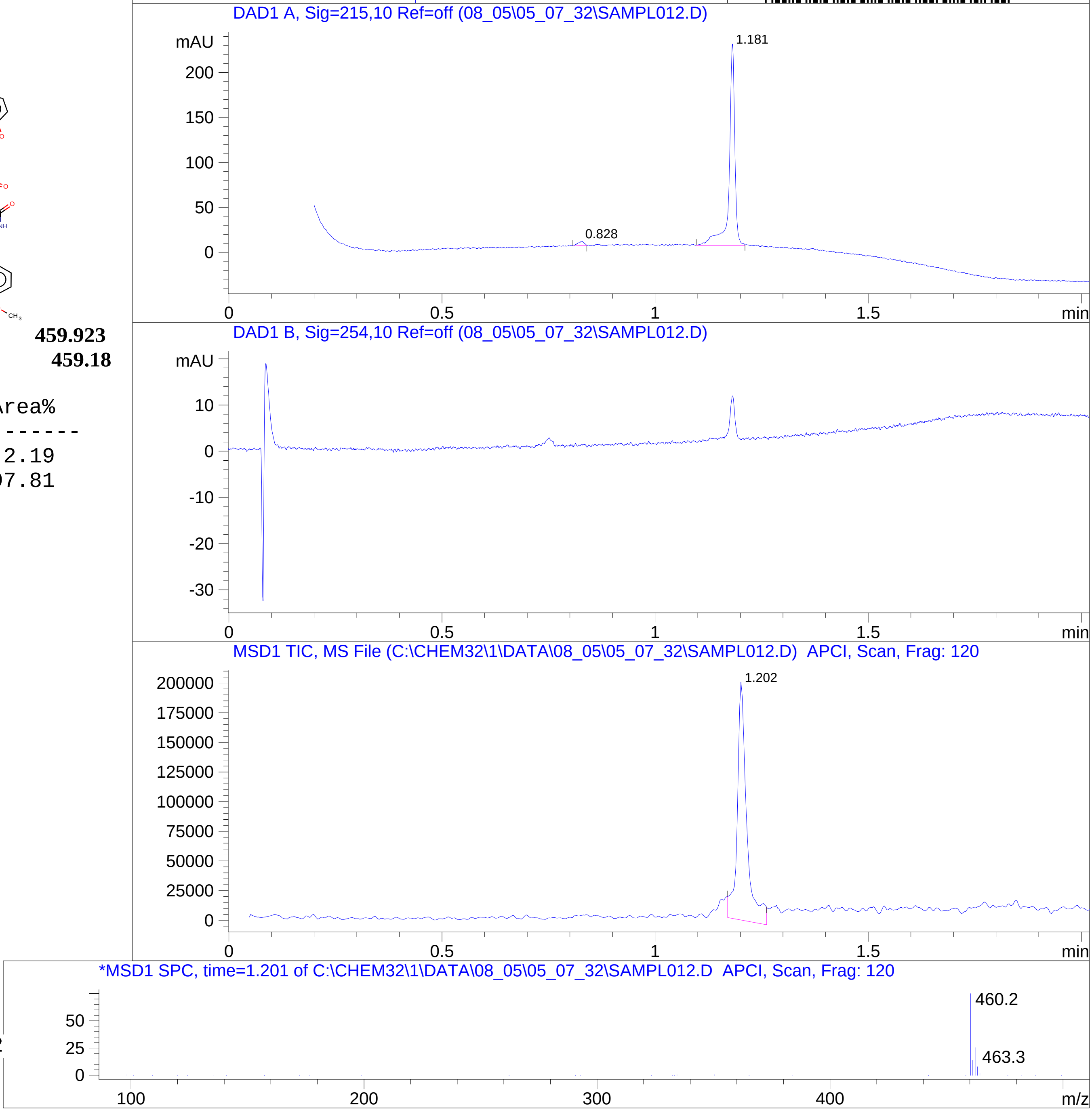

Inj.Date 5/8/2009 Link P2-B-03 VL Acq. Method C:\HPCHEM\-> ->
