# Supplementary material for: AI is a viable alternative to high throughput screening: a 318-target study
Source: Sci Rep. 2024 Apr 2;14:7526. doi: 10.1038/s41598-024-54655-z (PMC10987645; doi:10.1038/s41598-024-54655-z)
Supplement: Supplementary file 1 — Supplementary Information 1. [file 41598_2024_54655_MOESM1_ESM.zip › Nature SREP/QC_AIMS_files/Proj077.pdf]

MaxPeak: 100.00%
Ret_Time: 0.936 min

T8155027

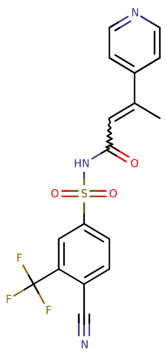

Mol Wt 395.36

Exact Mass 395.06

#	Time	Area%
1	0.936	100.00

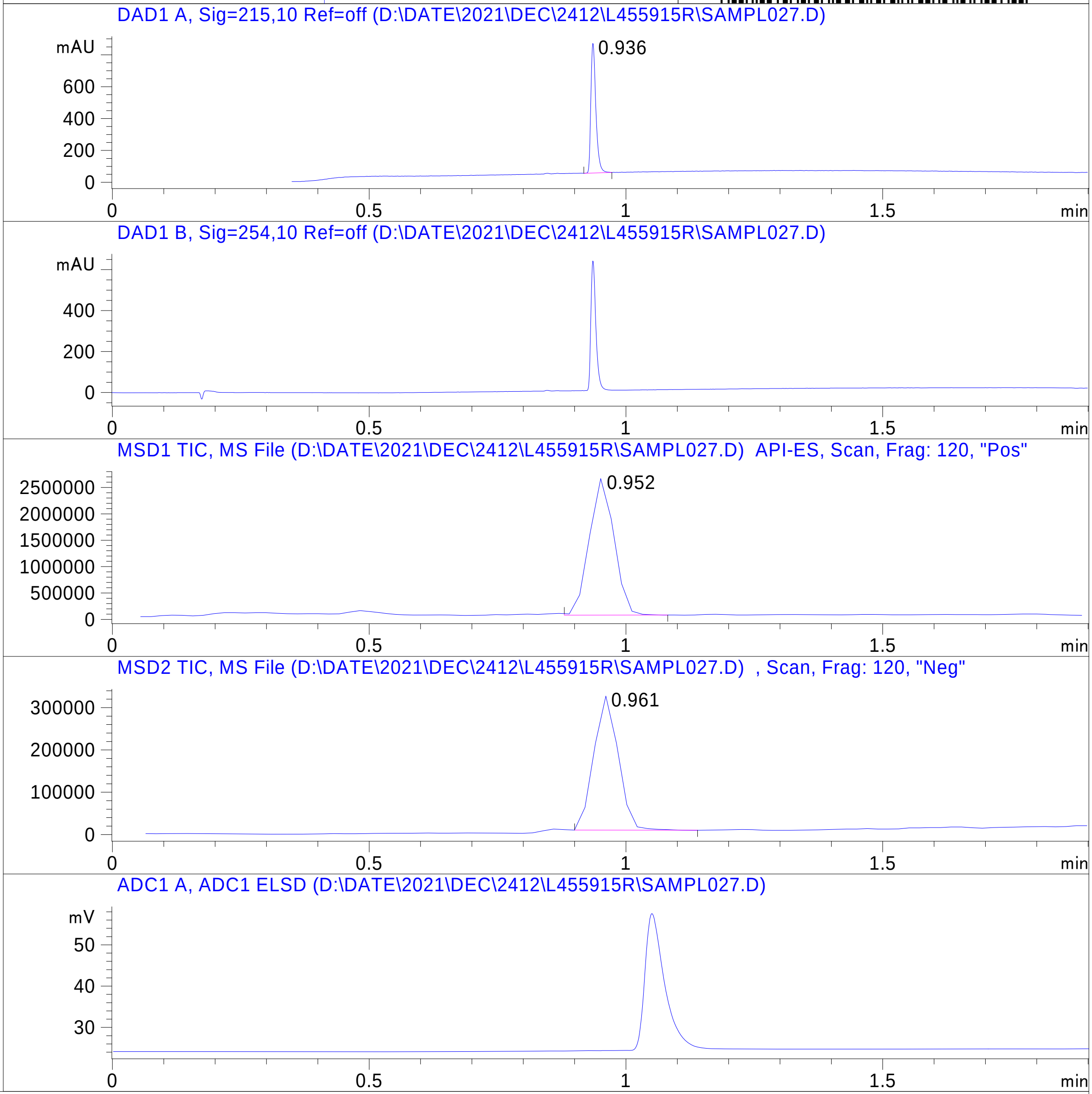

RT 0.952

RT 0.961

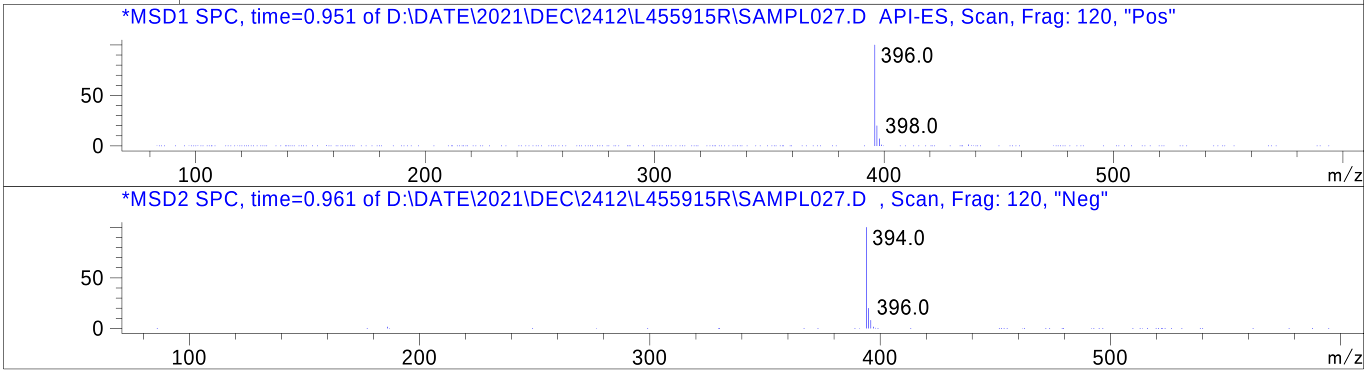

Inj.Date 24-Dec-21 Z P2-D-01 -VL- Acq. Method C:\HPCHEM\-> ->
