# Supplementary material for: AI is a viable alternative to high throughput screening: a 318-target study
Source: Sci Rep. 2024 Apr 2;14:7526. doi: 10.1038/s41598-024-54655-z (PMC10987645; doi:10.1038/s41598-024-54655-z)
Supplement: Supplementary file 1 — Supplementary Information 1. [file 41598_2024_54655_MOESM1_ESM.zip › Nature SREP/QC_AIMS_files/Proj085.pdf]

Data Sheet

**Product Name:**	YKL-05-099
**Cat. No.:**	CS-6458
**CAS No.:**	1936529-65-5
**Molecular Formula:**	$C_{32}H_{34}ClN_7O_3$
**Molecular Weight:**	600.11
**Target:**	Salt-inducible Kinase (SIK)
**Pathway:**	Immunology/Inflammation
**Solubility:**	DMSO : ≥ 75 mg/mL (124.98 mM)

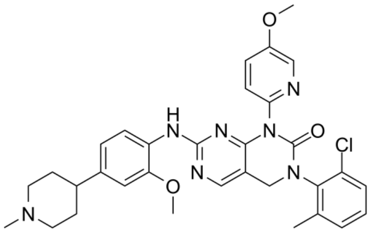

BIOLOGICAL ACTIVITY:

YKL-05-099 is a salt-inducible kinase (**SIK**) inhibitor. YKL-05-099 binds to **SIK1** and **SIK3** with **IC_{50}**s of ~10 and ~30 nM, respectively. YKL-05-099 has slightly less potent SIK2-inhibitory (IC_{50}=40 nM)[1]. IC50 & Target: IC50: 10 nM (SIK1), 30 nM (SIK3), 40 nM (SIK2) [1] **In Vitro:** YKL-05-099 has slightly less potent SIK2-inhibitory (IC_{50}=40 nM) and IL-10-enhancing activities (EC_{50}=460 nM). YKL-05-099 binds to SIK1 and SIK3 with IC_{50}s of 10 and 30 nM, respectively, in a competitive binding assay. Preincubating bone marrow-derived macrophages with YKL-05-099 reduces LPS stimulated phosphorylation of HDAC5 at the SIK-specific phosphorylation site Ser259. YKL-05-099 suppresses production of the inflammatory cytokines TNFα, IL-6 and IL-12p40, and only modestly enhances IL-1β release in BMDCs stimulated with the yeast cell wall extract Zymosan A[1]. **In Vivo:** YKL-05-099 is non-toxic at concentrations less than 10 μM and stable in mouse liver microsomes for more than 2 hours. YKL-05-099 is highly soluble (PBS solubility=428 μM) and present in an unbound state at appreciable levels in mouse plasma. YKL-05-099 dose dependently decreases phosphorylation of HDAC5 at the SIK-regulated site Ser259; reduced phosphorylation is observed at the lowest dose (5 mg/Kg) and is below the limit of detection by immunoblotting beginning at the 20 mg/Kg dose. YKL-05-099 dose-dependently reduces abundance of TNFα in serum beginning at 5 mg/Kg, and increases IL-10 levels at the 20 mg/Kg dose by more than 2-fold[1].

PROTOCOL (Extracted from published papers and Only for reference)

Animal Administration: [1]Mice: YKL-05-099 is diluted in 5% N-methyl-2-pyrrolidinone, 5% Solutol HS15 and 90% normal saline and administered IP to male 8–10 week-old C57BL/6 mice. Serum and tissue samples are collected after euthanizing mice by CO_2 inhalation overdose followed by cervical dislocation[1].

References:

[1]. Sundberg TB, et al. Development of Chemical Probes for Investigation of Salt-Inducible Kinase Function in Vivo. ACS Chem Biol. 2016 Aug 19;11(8):2105-11.

CAIndexNames:

Pyrimido[4,5-d]pyrimidin-2(1H)-one, 3-(2-chloro-6-methylphenyl)-3,4-dihydro-7-[[2-methoxy-4-(1-methyl-4-piperidinyl)phenyl]amino]-1-(5-methoxy-2-pyridinyl)-

SMILES:

O=C1N(C2=NC=C(OC)C=C2)C3=NC(NC4=CC=C(C5CCN(C)CC5)C=C4OC)=NC=C3CN1C6=C(C)C=CC=C6Cl

Caution: Product has not been fully validated for medical applications. For research use only.

Tel: 610-426-3128 Fax: 888-484-5008 E-mail: sales@ChemScene.com

Address: 1 Deer Park Dr, Suite Q, Monmouth Junction, NJ 08852, USA

Certificate of Analysis

**Product Name:**	YKL-05-099
**Cat. No.:**	CS-6458
**CAS No.:**	1936529-65-5
**Batch No.:**	33875
**Chemical Name:**	Pyrimido[4,5-d]pyrimidin-2(1H)-one, 3-(2-chloro-6-methylphenyl)-3,4-dihydro-7-[[2-methoxy-4-(1-methyl-4-piperidinyl)phenyl]amino]-1-(5-methoxy-2-pyridinyl)-

PHYSICAL AND CHEMICAL PROPERTIES

**Molecular Formula:**	$C_{32}H_{34}ClN_7O_3$
**Molecular Weight:**	600.11
**Storage:**	Storage temp. 2-8°C
**Chemical Structure:**	

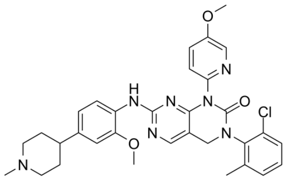

ANALYTICAL DATA

**Appearance:**	White to light yellow (Solid)
**^{1}H NMR Spectrum:**	Consistent with structure
**LCMS:**	Consistent with structure
**Purity (LCMS):**	99.76%
**Conclusion:**	The product has been tested and complies with the given specifications.

Caution: Product has not been fully validated for medical applications. For research use only.

Tel: 610-426-3128 Fax: 888-484-5008 E-mail: sales@ChemScene.com
Address: 1 Deer Park Dr, Suite Q, Monmouth Junction, NJ 08852, USA

Safety Data Sheet

Revision Date: May.-20-2021
Print Date: Jul.-30-2022

1. PRODUCT AND COMPANY IDENTIFICATION

1.1 Product identifier

Product name :	YKL-05-099
Catalog No. :	CS-6458
CAS No. :	1936529-65-5

1.2 Relevant identified uses of the substance or mixture and uses advised against

Identified uses :	Laboratory chemicals, manufacture of substances.

1.3 Details of the supplier of the safety data sheet

Company:	ChemScene LLC
Tel:	610-426-3128
Fax:	888-484-5008
E-mail:	sales@chemscene.com

1.4 Emergency telephone number

Emergency Phone #:	610-426-3128

2. HAZARDS IDENTIFICATION

2.1 Classification of the substance or mixture

GHS Classification in accordance with 29 CFR 1910 (OSHA HCS)

Acute toxicity, oral (Category 4),H302

Skin corrosion/irritation (Category 2),H315

Serious eye damage/eye irritation (Category 2A),H319

Specific target organ toxicity, single exposure; Respiratory tract irritation (Category 3),H335

2.2 GHS Label elements, including precautionary statements

Pictogram 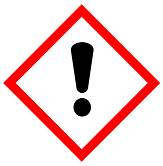

Signal word Warning

Hazard statement(s)

H302 Harmful if swallowed

H315 Causes skin irritation

H319 Causes serious eye irritation

H335 May cause respiratory irritation

Precautionary statement(s)

P261 Avoid breathing dust/fume/gas/mist/vapours/spray.

P280 Wear protective gloves/protective clothing/eye protection/face protection.

P301+P312 IF SWALLOWED: Call a POISON CENTER or doctor/physician if you feel unwell.

P302+P352 IF ON SKIN: Wash with plenty of soap and water.

P305+P351+P338 IF IN EYES: Rinse cautiously with water for several minutes. Remove contact lenses, if present and easy to do. Continue rinsing.

2.3 Other hazards

None.

3. COMPOSITION/INFORMATION ON INGREDIENTS

3.1 Substances

Formula:	$C_{32}H_{34}ClN_7O_3$
Molecular Weight:	600.11
CAS No. :	1936529-65-5

4. FIRST AID MEASURES

4.1 Description of first aid measures

Eye contact

Remove any contact lenses, locate eye-wash station, and flush eyes immediately with large amounts of water. Separate eyelids with fingers to ensure adequate flushing. Promptly call a physician.

Skin contact

Rinse skin thoroughly with large amounts of water. Remove contaminated clothing and shoes and call a physician.

Inhalation

Immediately relocate self or casualty to fresh air. If breathing is difficult, give cardiopulmonary resuscitation (CPR). Avoid mouth-to-mouth resuscitation.

Ingestion

Wash out mouth with water; Do NOT induce vomiting; call a physician.

4.2 Most important symptoms and effects, both acute and delayed

The most important known symptoms and effects are described in the labelling (see section 2.2).

4.3 Indication of any immediate medical attention and special treatment needed

Treat symptomatically.

5. FIRE FIGHTING MEASURES

5.1 Extinguishing media

Suitable extinguishing media

Use water spray, dry chemical, foam, and carbon dioxide fire extinguisher.

5.2 Special hazards arising from the substance or mixture

During combustion, may emit irritant fumes.

5.3 Advice for firefighters

Wear self-contained breathing apparatus and protective clothing.

6. ACCIDENTAL RELEASE MEASURES

6.1 Personal precautions, protective equipment and emergency procedures

Use full personal protective equipment. Avoid breathing vapors, mist, dust or gas. Ensure adequate ventilation. Evacuate personnel to safe areas.

Refer to protective measures listed in sections 8.

6.2 Environmental precautions

Try to prevent further leakage or spillage. Keep the product away from drains or water courses.

6.3 Methods and materials for containment and cleaning up

Absorb solutions with finely-powdered liquid-binding material (diatomite, universal binders); Decontaminate surfaces and equipment by scrubbing with alcohol; Dispose of contaminated material according to Section 13.

7. HANDLING AND STORAGE

7.1 Precautions for safe handling

Avoid inhalation, contact with eyes and skin. Avoid dust and aerosol formation. Use only in areas with appropriate exhaust ventilation.

7.2 Conditions for safe storage, including any incompatibilities

Keep container tightly sealed in cool, well-ventilated area. Keep away from direct sunlight and sources of ignition.

Recommended storage temperature: Storage temp. 2-8°C

Shipping at room temperature if less than 2 weeks.

7.3 Specific end use(s)

No data available.

8. EXPOSURE CONTROLS/PERSONAL PROTECTION

8.1 Control parameters

Components with workplace control parameters

This product contains no substances with occupational exposure limit values.

8.2 Exposure controls

Engineering controls

Ensure adequate ventilation. Provide accessible safety shower and eye wash station.

Personal protective equipment

**Eye protection**	Safety goggles with side-shields.
**Hand protection**	Protective gloves.
**Skin and body protection**	Impervious clothing.
**Respiratory protection**	Suitable respirator.
**Environmental exposure controls**	Keep the product away from drains, water courses or the soil. Clean spillages in a safe way as soon as possible.

9. PHYSICAL AND CHEMICAL PROPERTIES

9.1 Information on basic physical and chemical properties

**Appearance**	Solid
**Odor**	No data available
**Odor threshold**	No data available
**pH**	No data available
**Melting/freezing point**	No data available
**Boiling point/range**	No data available
**Flash point**	No data available
**Evaporation rate**	No data available
**Flammability (solid, gas)**	No data available
**Upper/lower flammability or explosive limits**	No data available
**Vapor pressure**	No data available
**Vapor density**	No data available
**Relative density**	No data available

**Water Solubility**	No data available
**Partition coefficient**	No data available
**Auto-ignition temperature**	No data available
**Decomposition temperature**	No data available
**Viscosity**	No data available
**Explosive properties**	No data available
**Oxidizing properties**	No data available

9.2 Other safety information

No data available.

10. STABILITY AND REACTIVITY

10.1 Reactivity

No data available.

10.2 Chemical stability

Stable under recommended storage conditions.

10.3 Possibility of hazardous reactions

No data available.

10.4 Conditions to avoid

No data available.

10.5 Incompatible materials

Strong acids/alkalis, strong oxidising/reducing agents.

10.6 Hazardous decomposition products

Under fire conditions, may decompose and emit toxic fumes.

Other decomposition products - no data available.

11.TOXICOLOGICAL INFORMATION

11.1 Information on toxicological effects

Acute toxicity

Classified based on available data. For more details, see section 2

Skin corrosion/irritation

Classified based on available data. For more details, see section 2

Serious eye damage/irritation

Classified based on available data. For more details, see section 2

Respiratory or skin sensitization

Classified based on available data. For more details, see section 2

Germ cell mutagenicity

Classified based on available data. For more details, see section 2

Carcinogenicity

IARC: No component of this product present at a level equal to or greater than 0.1% is identified as probable, possible or confirmed human carcinogen by IARC.

ACGIH: No component of this product present at a level equal to or greater than 0.1% is identified as a potential or confirmed carcinogen by ACGIH.

NTP: No component of this product present at a level equal to or greater than 0.1% is identified as a anticipated or confirmed carcinogen by NTP.

OSHA: No component of this product present at a level equal to or greater than 0.1% is identified as a potential or confirmed carcinogen by OSHA.

Reproductive toxicity

Classified based on available data. For more details, see section 2

Specific target organ toxicity - single exposure

Classified based on available data. For more details, see section 2

Specific target organ toxicity - repeated exposure

Classified based on available data. For more details, see section 2

Aspiration hazard

Classified based on available data. For more details, see section 2

Additional information

This information is based on our current knowledge. However the chemical, physical, and toxicological properties have not been completely investigated.

12. ECOLOGICAL INFORMATION

12.1 Toxicity

No data available.

12.2 Persistence and degradability

No data available.

12.3 Bioaccumlative potential

No data available.

12.4 Mobility in soil

No data available.

12.5 Results of PBT and vPvB assessment

PBT/vPvB assessment unavailable as chemical safety assessment not required or not conducted.

12.6 Other adverse effects

No data available.

13. DISPOSAL CONSIDERATIONS

13.1 Waste treatment methods

Product

Dispose substance in accordance with prevailing country, federal, state and local regulations.

Contaminated packaging

Conduct recycling or disposal in accordance with prevailing country, federal, state and local regulations.

14. TRANSPORT INFORMATION

DOT (US)

Proper shipping name: Not dangerous goods

UN number: -

Class: -

Packing group: -

IMDG

Proper shipping name: Not dangerous goods

UN number: -

Class: -

Packing group: -

IATA

Proper shipping name: Not dangerous goods

UN number: -

Class: -

Packing group: -

15. REGULATORY INFORMATION

SARA 302 Components:

No chemicals in this material are subject to the reporting requirements of SARA Title III, Section 302.

SARA 313 Components:

This material does not contain any chemical components with known CAS numbers that exceed the threshold (De Minimis) reporting levels established by SARA Title III, Section 313.

SARA 311/312 Hazards:

No SARA Hazards.

Massachusetts Right To Know Components:

No components are subject to the Massachusetts Right to Know Act.

Pennsylvania Right To Know Components:

No components are subject to the Pennsylvania Right to Know Act.

New Jersey Right To Know Components:

No components are subject to the New Jersey Right to Know Act.

California Prop. 65 Components:

This product does not contain any chemicals known to State of California to cause cancer, birth defects, or anyother reproductive harm.

16. OTHER INFORMATION

Copyright 2022 ChemScene. The above information is correct to the best of our present knowledge but does not purport to be all inclusive and should be used only as a guide. The product is for research use only and for experienced personnel. It must only be handled by suitably qualified experienced scientists in appropriately equipped and authorized facilities. The burden of safe use of this material rests entirely with the user. ChemScene disclaims all liability for any damage resulting from handling or from contact with this product.

File ..1\DATA\2018\20181101\475\BIZ2018-N01-CXL1.D Tgt Mass (CHM):
Injection Date : 1 Nov 18 5:02 pm +0800 Seq. Line : 9
Sample Name : BIZ2018-N01-CXL1 Location : P1-B-07
Acq. Operator : LQ_1052 Inj : 1
Spec. Reported : MS Integration Inj Volume : 1 ul
Acq. Method : D:\Chem32\1\data\2018\20181101\475\1-POS-3MIN-1.M
Analysis Method : D:\CHEM32\1\DATA\2018\20181101\475\1-POS-3MIN-1.M
Catalog No : CS-6458 Batch#33875 A-RP-314
Method Info : Mobile Phase: A: water(0.01%TFA) B:ACN(0.01%TFA)
Gradient: 5% to 95%B within 1.3 min
Flow Rate :1.8ml/min
Column :SunFire C18, 4.6*50mm,3.5um A-RP-314
Oven Temperature : 45

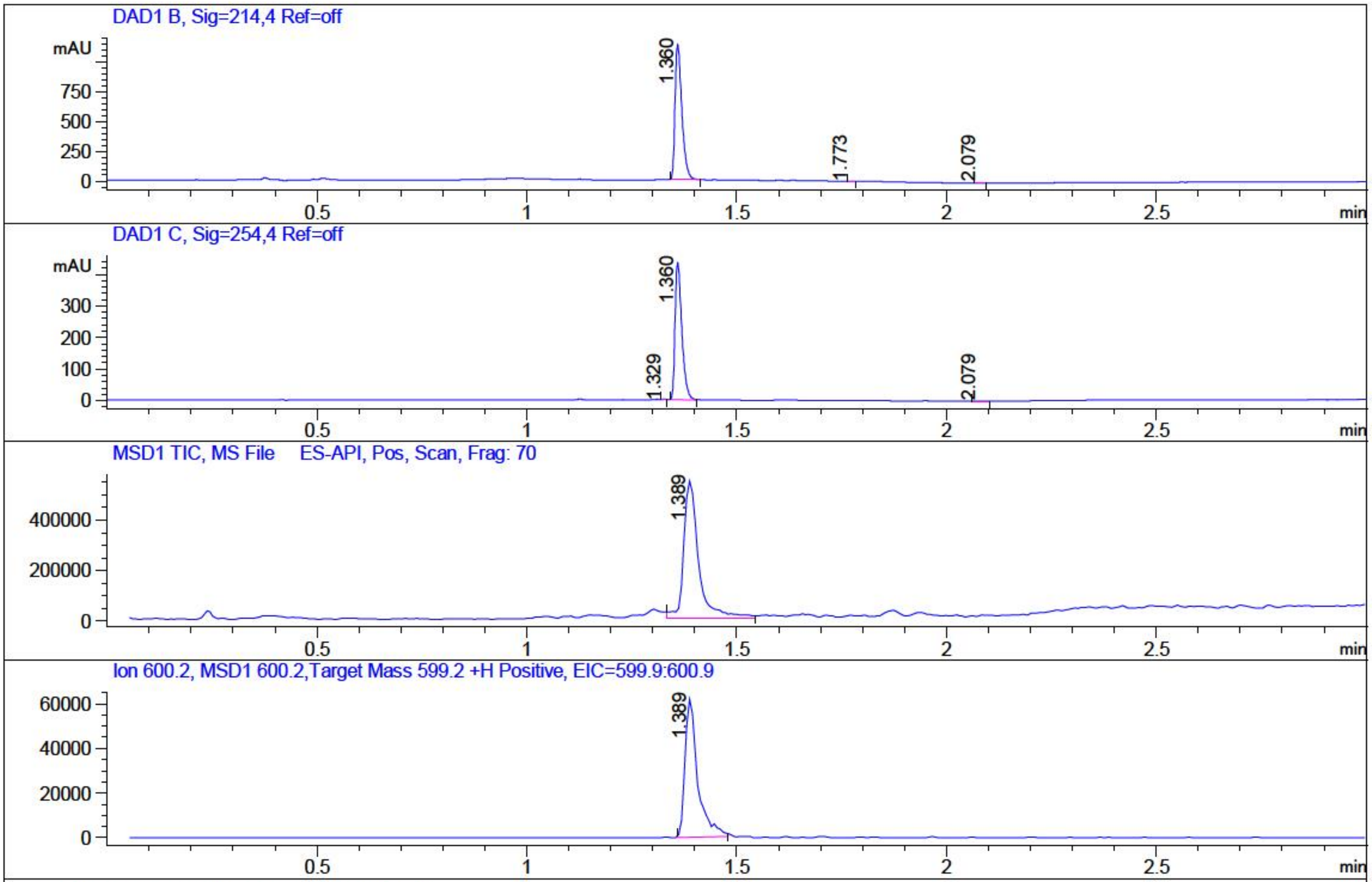

Integration Results for DAD1 B, Sig=214,4 Ref=off

RetTim	Width	Area	Height	Area%	MS(+)
1.36	0.02	1291.05	1134.26	99.76	301
1.77	0.01	0.86	1.16	0.07	141
2.08	0.01	2.25	2.57	0.17	141

Integration Results for DAD1 C, Sig=254,4 Ref=off

RetTim	Width	Area	Height	Area%	MS(+)
1.33	0.01	0.23	0.36	0.05	292
1.36	0.02	489.08	434.32	99.87	301
2.08	0.03	0.42	0.28	0.09	141

Integration Results for MSD1 TIC, MS File

RetTim	Width	Area	Height	Area%	MS(+)
1.39	0.04	1390705.00	548098.31	100.00	301

Ret. Time: 1.39 <<<< POSITIVE SPECTRA >>>>

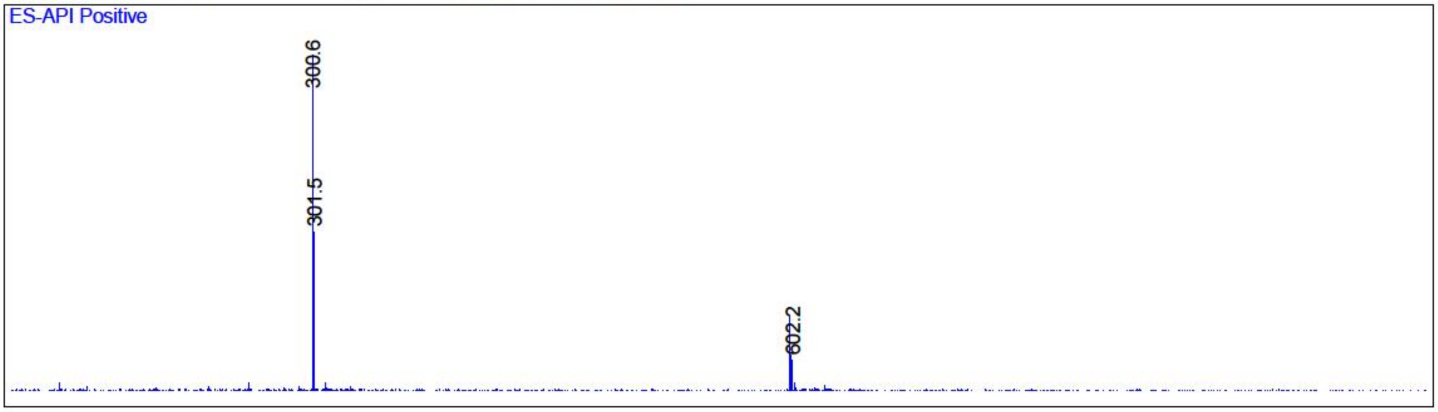

Sample ID: BIZ2018-N01-CXL1, Catalog No: CS-6458 Batch#33875 , DMSO

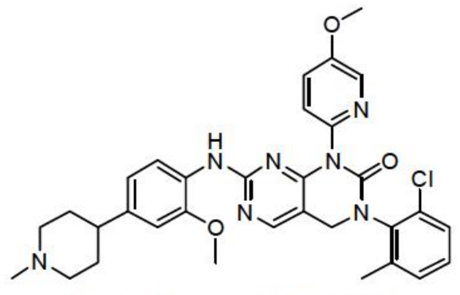

Chemical Formula: $C_{32}H_{34}ClN_7O_3$
Exact Mass: 599.24

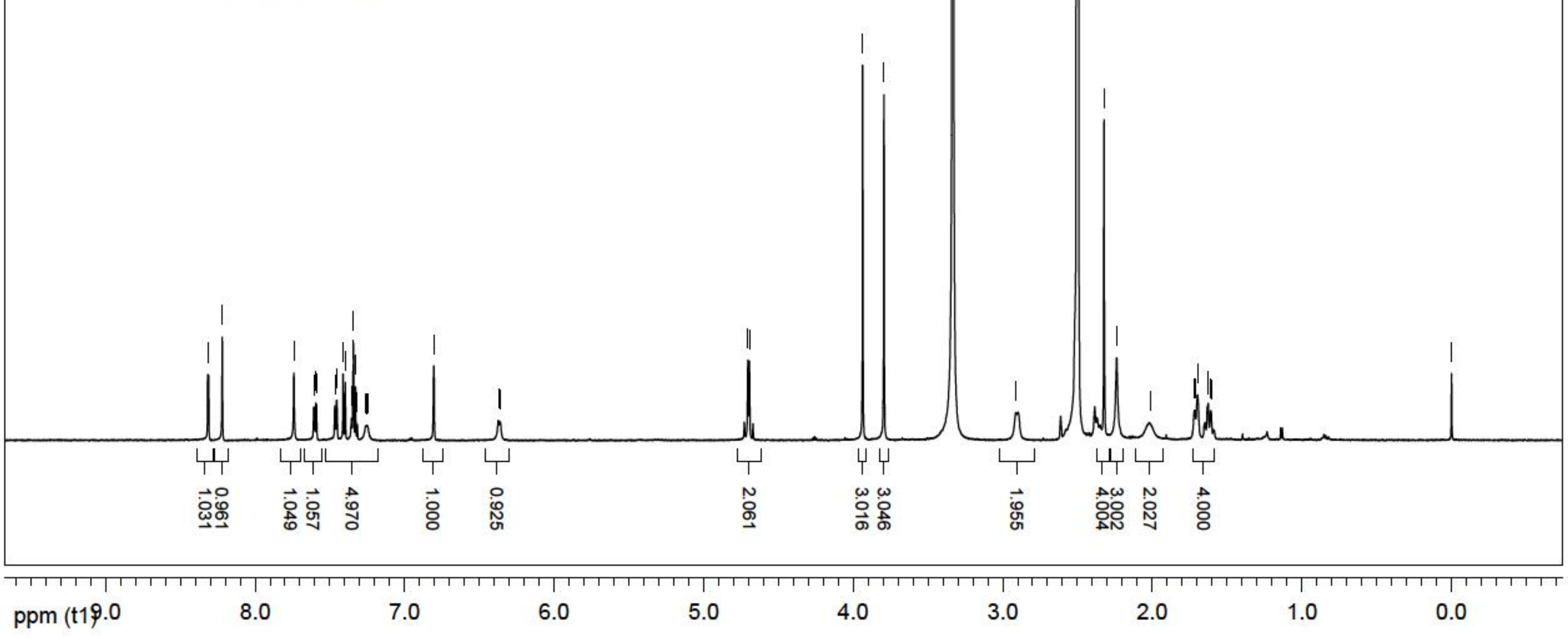

ChemScene

Date:
2 Nov 2018
Document's Title:
Catalog No: CS-6458 Batch#33875

Spectrum Title:
BIZ2018-N01-CXL1-DMSO-181101

Frequency (MHz):
(f1) 600.130
Original Points Count:
(f1) 65536
Actual Points Count:
(f1) 65536
Acquisition Time (sec):
(f1) 4.3691
Spectral Width (ppm):
(f1) 24.995
Pulse Program:
Unknown
