# Supplementary material for: AI is a viable alternative to high throughput screening: a 318-target study
Source: Sci Rep. 2024 Apr 2;14:7526. doi: 10.1038/s41598-024-54655-z (PMC10987645; doi:10.1038/s41598-024-54655-z)
Supplement: Supplementary file 1 — Supplementary Information 1. [file 41598_2024_54655_MOESM1_ESM.zip › Nature SREP/QC_AIMS_files/Proj090.pdf]

Data Sheet

**Product Name:**	RAF265
**Cat. No.:**	CS-0232
**CAS No.:**	927880-90-8
**Molecular Formula:**	$C_{24}H_{16}F_6N_6O$
**Molecular Weight:**	518.41
**Target:**	Apoptosis; Autophagy; Raf; VEGFR
**Pathway:**	Apoptosis; Autophagy; MAPK/ERK Pathway; Protein Tyrosine Kinase/RTK
**Solubility:**	DMSO : ≥ 26 mg/mL (50.15 mM); Ethanol : 10 mg/mL (19.29 mM; Need ultrasonic)

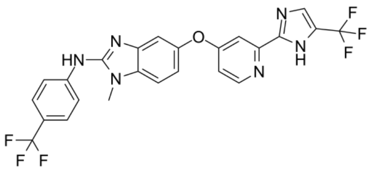

BIOLOGICAL ACTIVITY:

RAF265 is a potent **RAF/VEGFR2** inhibitor. IC50 & Target: RAF[1]
VEGFR2[1] **In Vitro:** The MTT assay reveals that in HT29 and MDAMB231 cells, RAF265 alone shows significant activity with IC_{20} values of 1 to 3 μM and IC_{50} values of 5 to 10 μM. In A549 and HCT116 cells, IC_{20} values are 1 μM for both, but RAF265 concentrations up to 10 μM do not reach IC_{50} values. However, in the presence of 1 nM RAD001, the IC_{50} for RAF265 is 5 μM in A549 cells and 10 μM in HCT116 cells[1]. **In Vivo:** In single-compound efficacy studies, optimal dosing of RAD001 and RAF265 is 5 to 12 mg/kg daily and 30 mg/kg every two days, respectively. However, combination tolerability studies in nontumor-bearing mice defin dose-limiting toxicity as a 10% weight loss with the combination of RAD001 at a dose of 12 mg/kg daily and RAF265 at a dose of 20 mg/kg every two days. Therefore, the combination of RAF265 at a dose of 12 mg/kg qd and RAD001 at a dose of 12 mg/kg qd seems to be the maximal tolerated dose. RAD001 and RAF265 are both given at a dose of 12 mg/kg qd, alone or concurrently, over 6 days. After a 2-day stop, the compounds are given for another 6 days, and the treatment is then stopped. To confirm the potential of the combination of RAF265 and RAD001, the antitumor effect of the combination is tested in HCT116 xenografts (KRAS mut, PIK3CA mut). In HCT116 xenografts, RAD001 or RAF265 given alone shows 60% to 65% and 71% to 72% TVI%, respectively[1].

PROTOCOL (Extracted from published papers and Only for reference)

Cell Assay: [1]The MTT assay and Bliss additivism model are used to assess the effect of the combination on cell viability. Human A549 and H460 lung, HT29 and HCT 116 colon, and MDAMB231 breast cancer cell lines are used. In each well of a 96-well plate, 1×10^4 cells are grown in 200 μL of medium. After 24 h, RAD001, **RAF265**, or the combination is added to achieve a final concentration of 0.1 to 10 nM and **0.1 to 10 μM**, respectively. After 48 h of treatment, 20 μL of 5 mg/mL MTT solution in PBS is added to each well. After 4 h, supernatant is removed and formazan crystals are discarded in 200 μL of DMSO. Absorbance is then measured at 595 nm using an absorbance plate reader. Data are expressed as the percentage of viable cells in treated relative to nontreated conditions[1].
Animal Administration: [1]Mice[1]
The efficacy of the combination is also tested in vivo. A total of 3×10^6 A549, H460, HCT116, or MDAMB231 cells are injected s.c. into the flank region of **6-wk-old female athymic mice**. When tumors reach 50 mm3, the mice are randomized into four groups (n=7/group) for the following treatment: vehicle, **RAF265 (12 mg/kg daily)**, RAD001 (12 mg/kg daily), or both. All drug are administered over 14 d (6 d on, 2 d off, 6 d on), and the drug combination is administered concurrently. Control mice receive the respective vehicles of both drugs. Animal weight and tumor volumes are taken twice weekly and expressed relative to initial tumor volume. Tumors are measured until achieving a relative volume of 10 times the initial volume, and the time to this end point is noted. Drug efficacy is assessed based on the tumor growth curve, growth delay, and tumor volume inhibition percentage. The tumor growth

curve is designed to depict the evolution of the relative tumor size over time. The tumor volume inhibition percentage (TVI%) is calculated[1].

References:

[1]. Mordant P, et al. Dependence on phosphoinositide 3-kinase and RAS-RAF pathways drive the activity of RAF265, a novel RAF/VEGFR2 inhibitor, and RAD001 (Everolimus) in combination. Mol Cancer Ther. 2010 Feb;9(2):358-68.

CAIndexNames:

1H-Benzimidazol-2-amine, 1-methyl-5-[[2-[5-(trifluoromethyl)-1H-imidazol-2-yl]-4-pyridinyl]oxy]-N-[4-(trifluoromethyl)phenyl]-

SMILES:

FC(F)(C1=CN=C(N1)C2=NC=CC(OC3=CC=C4N(C(NC5=CC=C(C(F)(F)F)C=C5)=NC4=C3)C)=C2)F

Caution: Product has not been fully validated for medical applications. For research use only.

Tel: 610-426-3128 Fax: 888-484-5008 E-mail: sales@ChemScene.com
Address: 1 Deer Park Dr, Suite Q, Monmouth Junction, NJ 08852, USA

Certificate of Analysis

**Product Name:**	RAF265
**Cat. No.:**	CS-0232
**CAS No.:**	927880-90-8
**Batch No.:**	22713
**Chemical Name:**	1H-Benzimidazol-2-amine, 1-methyl-5-[[2-[5-(trifluoromethyl)-1H-imidazol-2-yl]-4-pyridinyl]oxy]-N-[4-(trifluoromethyl)phenyl]-

PHYSICAL AND CHEMICAL PROPERTIES

**Molecular Formula:**	$C_{24}H_{16}F_6N_6O$
**Molecular Weight:**	518.41
**Storage:**	Storage temp. 2-8°C
**Chemical Structure:**	

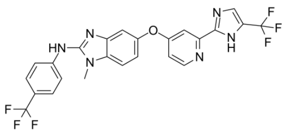

ANALYTICAL DATA

**Appearance:**	White to khaki (Solid)
**LCMS:**	Consistent with structure
**Purity (LCMS):**	99.90%
**Conclusion:**	The product has been tested and complies with the given specifications.

Caution: Product has not been fully validated for medical applications. For research use only.

Tel: 610-426-3128 Fax: 888-484-5008 E-mail: sales@ChemScene.com
Address: 1 Deer Park Dr, Suite Q, Monmouth Junction, NJ 08852, USA

Safety Data Sheet

Revision Date: Apr.-18-2022
Print Date: Jun.-17-2022

1. PRODUCT AND COMPANY IDENTIFICATION

1.1 Product identifier

Product name :	RAF265
Catalog No. :	CS-0232
CAS No. :	927880-90-8

1.2 Relevant identified uses of the substance or mixture and uses advised against

Identified uses :	Laboratory chemicals, manufacture of substances.

1.3 Details of the supplier of the safety data sheet

Company:	ChemScene LLC
Tel:	610-426-3128
Fax:	888-484-5008
E-mail:	sales@chemscene.com

1.4 Emergency telephone number

Emergency Phone #:	610-426-3128

2. HAZARDS IDENTIFICATION

2.1 Classification of the substance or mixture

Not a hazardous substance or mixture.

2.2 GHS Label elements, including precautionary statements

Not a hazardous substance or mixture.

2.3 Other hazards

None.

3. COMPOSITION/INFORMATION ON INGREDIENTS

3.1 Substances

Synonyms:	CHIR-265
Formula:	$C_{24}H_{16}F_6N_6O$
Molecular Weight:	518.41
CAS No. :	927880-90-8

4. FIRST AID MEASURES

4.1 Description of first aid measures

Eye contact

Remove any contact lenses, locate eye-wash station, and flush eyes immediately with large amounts of water. Separate eyelids with fingers to ensure adequate flushing. Promptly call a physician.

Skin contact

Rinse skin thoroughly with large amounts of water. Remove contaminated clothing and shoes and call a physician.

Inhalation

Immediately relocate self or casualty to fresh air. If breathing is difficult, give cardiopulmonary resuscitation (CPR). Avoid mouth-to-mouth resuscitation.

Ingestion

Wash out mouth with water; Do NOT induce vomiting; call a physician.

4.2 Most important symptoms and effects, both acute and delayed

The most important known symptoms and effects are described in the labelling (see section 2.2).

4.3 Indication of any immediate medical attention and special treatment needed

Treat symptomatically.

5. FIRE FIGHTING MEASURES

5.1 Extinguishing media

Suitable extinguishing media

Use water spray, dry chemical, foam, and carbon dioxide fire extinguisher.

5.2 Special hazards arising from the substance or mixture

During combustion, may emit irritant fumes.

5.3 Advice for firefighters

Wear self-contained breathing apparatus and protective clothing.

6. ACCIDENTAL RELEASE MEASURES

6.1 Personal precautions, protective equipment and emergency procedures

Use full personal protective equipment. Avoid breathing vapors, mist, dust or gas. Ensure adequate ventilation. Evacuate personnel to safe areas.

Refer to protective measures listed in sections 8.

6.2 Environmental precautions

Try to prevent further leakage or spillage. Keep the product away from drains or water courses.

6.3 Methods and materials for containment and cleaning up

Absorb solutions with finely-powdered liquid-binding material (diatomite, universal binders); Decontaminate surfaces and equipment by scrubbing with alcohol; Dispose of contaminated material according to Section 13.

7. HANDLING AND STORAGE

7.1 Precautions for safe handling

Avoid inhalation, contact with eyes and skin. Avoid dust and aerosol formation. Use only in areas with appropriate exhaust ventilation.

7.2 Conditions for safe storage, including any incompatibilities

Keep container tightly sealed in cool, well-ventilated area. Keep away from direct sunlight and sources of ignition.

Recommended storage temperature: Storage temp. 2-8°C

Shipping at room temperature if less than 2 weeks.

7.3 Specific end use(s)

No data available.

8. EXPOSURE CONTROLS/PERSONAL PROTECTION

8.1 Control parameters

Components with workplace control parameters

This product contains no substances with occupational exposure limit values.

8.2 Exposure controls

Engineering controls

Ensure adequate ventilation. Provide accessible safety shower and eye wash station.

Personal protective equipment

**Eye protection**	Safety goggles with side-shields.
**Hand protection**	Protective gloves.
**Skin and body protection**	Impervious clothing.
**Respiratory protection**	Suitable respirator.
**Environmental exposure controls**	Keep the product away from drains, water courses or the soil. Clean spillages in a safe way as soon as possible.

9. PHYSICAL AND CHEMICAL PROPERTIES

9.1 Information on basic physical and chemical properties

**Appearance**	Solid
**Odor**	No data available
**Odor threshold**	No data available
**pH**	No data available
**Melting/freezing point**	No data available
**Boiling point/range**	667.6±65.0°C at 760 mmHg
**Flash point**	No data available
**Evaporation rate**	No data available
**Flammability (solid, gas)**	No data available
**Upper/lower flammability or explosive limits**	No data available
**Vapor pressure**	No data available
**Vapor density**	No data available
**Relative density**	No data available
**Water Solubility**	No data available
**Partition coefficient**	No data available
**Auto-ignition temperature**	No data available
**Decomposition temperature**	No data available
**Viscosity**	No data available
**Explosive properties**	No data available
**Oxidizing properties**	No data available

9.2 Other safety information

No data available.

10. STABILITY AND REACTIVITY

10.1 Reactivity

No data available.

10.2 Chemical stability

Stable under recommended storage conditions.

10.3 Possibility of hazardous reactions

No data available.

10.4 Conditions to avoid

No data available.

10.5 Incompatible materials

Strong acids/alkalis, strong oxidising/reducing agents.

10.6 Hazardous decomposition products

Under fire conditions, may decompose and emit toxic fumes.

Other decomposition products - no data available.

11.TOXICOLOGICAL INFORMATION

11.1 Information on toxicological effects

Acute toxicity

Classified based on available data. For more details, see section 2

Skin corrosion/irritation

Classified based on available data. For more details, see section 2

Serious eye damage/irritation

Classified based on available data. For more details, see section 2

Respiratory or skin sensitization

Classified based on available data. For more details, see section 2

Germ cell mutagenicity

Classified based on available data. For more details, see section 2

Carcinogenicity

IARC: No component of this product present at a level equal to or greater than 0.1% is identified as probable, possible or confirmed human carcinogen by IARC.

ACGIH: No component of this product present at a level equal to or greater than 0.1% is identified as a potential or confirmed carcinogen by ACGIH.

NTP: No component of this product present at a level equal to or greater than 0.1% is identified as a anticipated or confirmed carcinogen by NTP.

OSHA: No component of this product present at a level equal to or greater than 0.1% is identified as a potential or confirmed carcinogen by OSHA.

Reproductive toxicity

Classified based on available data. For more details, see section 2

Specific target organ toxicity - single exposure

Classified based on available data. For more details, see section 2

Specific target organ toxicity - repeated exposure

Classified based on available data. For more details, see section 2

Aspiration hazard

Classified based on available data. For more details, see section 2

Additional information

This information is based on our current knowledge. However the chemical, physical, and toxicological properties have not been completely investigated.

12. ECOLOGICAL INFORMATION

12.1 Toxicity

No data available.

12.2 Persistence and degradability

No data available.

12.3 Bioaccumlative potential

No data available.

12.4 Mobility in soil

No data available.

12.5 Results of PBT and vPvB assessment

PBT/vPvB assessment unavailable as chemical safety assessment not required or not conducted.

12.6 Other adverse effects

No data available.

13. DISPOSAL CONSIDERATIONS

13.1 Waste treatment methods

Product

Dispose substance in accordance with prevailing country, federal, state and local regulations.

Contaminated packaging

Conduct recycling or disposal in accordance with prevailing country, federal, state and local regulations.

14. TRANSPORT INFORMATION

DOT (US)

Proper shipping name: Not dangerous goods

UN number: -

Class: -

Packing group: -

IMDG

Proper shipping name: Not dangerous goods

UN number: -

Class: -

Packing group: -

IATA

Proper shipping name: Not dangerous goods

UN number: -

Class: -

Packing group: -

15. REGULATORY INFORMATION

SARA 302 Components:

No chemicals in this material are subject to the reporting requirements of SARA Title III, Section 302.

SARA 313 Components:

This material does not contain any chemical components with known CAS numbers that exceed the threshold (De Minimis) reporting levels established by SARA Title III, Section 313.

SARA 311/312 Hazards:

No SARA Hazards.

Massachusetts Right To Know Components:

No components are subject to the Massachusetts Right to Know Act.

Pennsylvania Right To Know Components:

No components are subject to the Pennsylvania Right to Know Act.

New Jersey Right To Know Components:

No components are subject to the New Jersey Right to Know Act.

California Prop. 65 Components:

This product does not contain any chemicals known to State of California to cause cancer, birth defects, or anyother reproductive harm.

16. OTHER INFORMATION

Copyright 2022 ChemScene. The above information is correct to the best of our present knowledge but does not purport to be all inclusive and should be used only as a guide. The product is for research use only and for experienced personnel. It must only be handled by suitably qualified experienced scientists in appropriately equipped and authorized facilities. The burden of safe use of this material rests entirely with the user. ChemScene disclaims all liability for any damage resulting from handling or from contact with this product.

SAMPLE INFORMATION

CAS NO.:	927880-90-8	Acquired By:	LY
Sample infromation:		Date Acquired:	3/31/2022 4:54:29 PM CST
Vial:	1:B,4	Acq. Method Set:	1_POS_2MIN
Injection #:	1	Date Processed:	3/31/2022 5:51:19 PM CST,
Injection Volume:	1.00 ul	Processing Method:	PDA_01, MS_scan
Run Time:	2.8 Minutes	Channel Name:	PDA Ch1 214nm@4.8nm, MS TIC,
Sample Set Name:	20220331	Column Name:	

Auto-Scaled Chromatogram

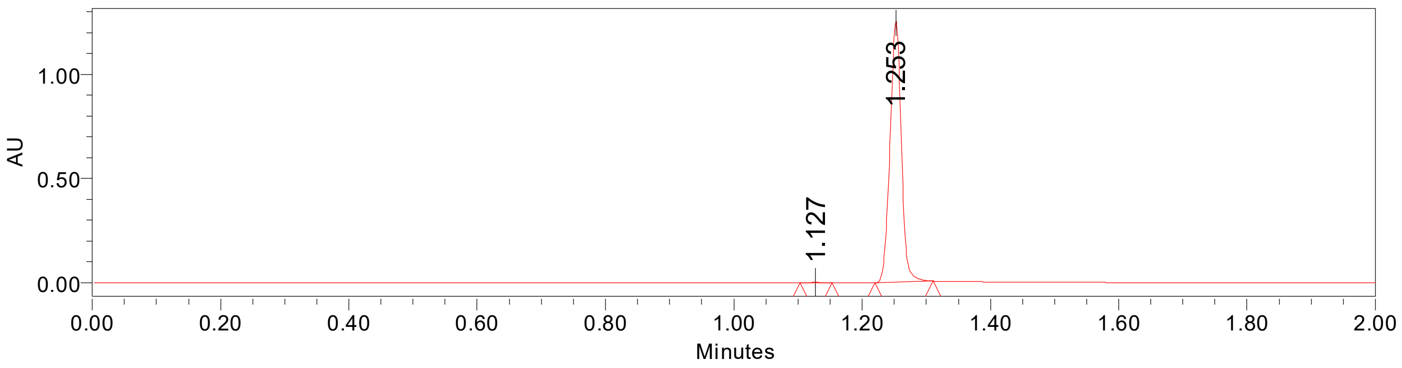

Processed Channel Descr. PDA Ch2 254nm@4.8nm

Auto-Scaled Chromatogram

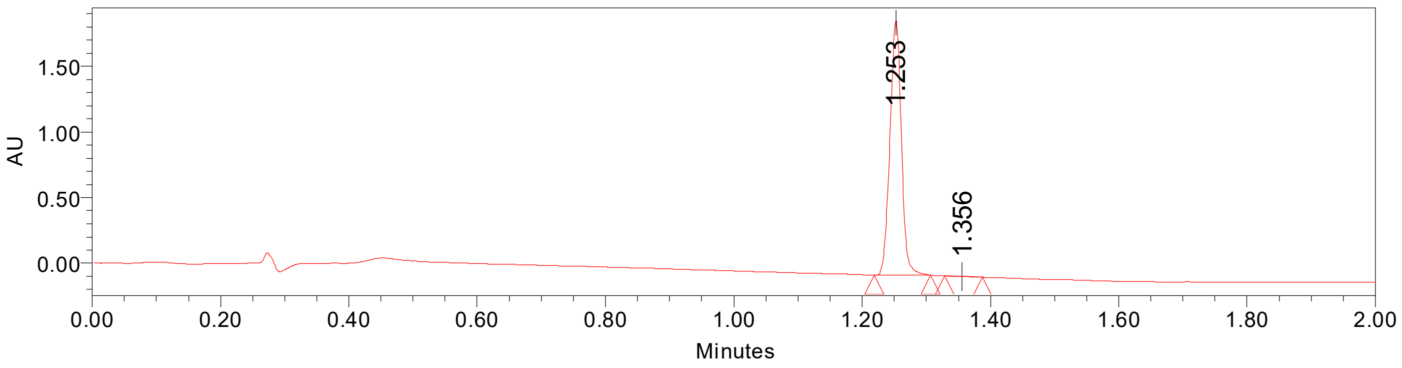

Processed Channel Descr. PDA Ch1 214nm@4.8nm

Auto-Scaled Chromatogram

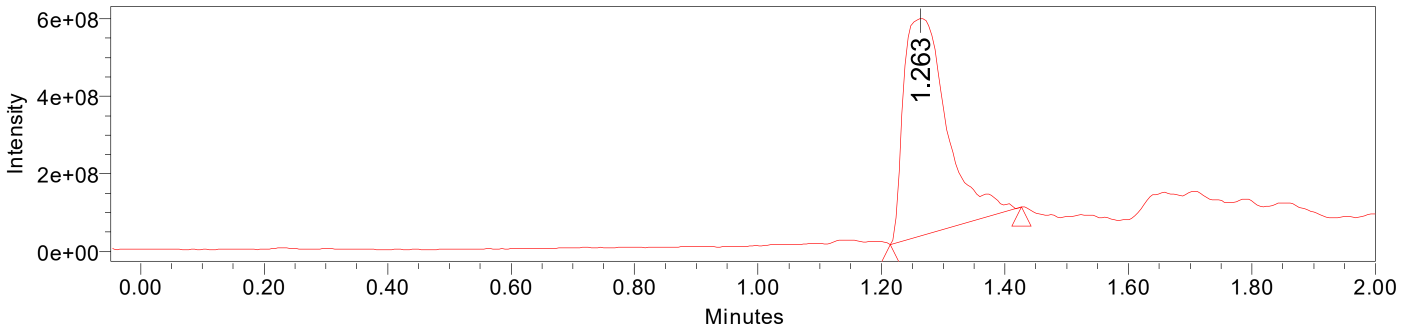

Processed Channel Descr. ACQ-SQD2 1: MS Scan MS TIC, Smoothed by 9 point Savitzky-Golay Filter., Time offset by -0.053 mins. (1: 150.00-1000.00 ES+, Centroid, CV=30)

Peak Results
Channel Name: PDA Ch2 254nm@4.8nm

	RT	Width (sec)	Area	Height	% Area	Base Peak (Combined) (m/z)	Channel Name
1	1.127	3.000	1591	1474	0.10	150.88	PDA Ch2 254nm@4.8nm
2	1.253	5.450	1535182	1250736	99.90	151.91	PDA Ch2 254nm@4.8nm

Peak Results
Channel Name: PDA Ch1 214nm@4.8nm

	RT	Width (sec)	Area	Height	% Area	Base Peak (Combined) (m/z)	Channel Name
1	1.253	5.250	2400695	1938690	99.90	151.91	PDA Ch1 214nm@4.8nm
2	1.356	3.550	2380	1714	0.10	280.52	PDA Ch1 214nm@4.8nm

Peak Results
Channel Name: MS TIC

	RT	Width (sec)	Area	Height	% Area	Base Peak (Combined) (m/z)	Channel Name
1	1.263	12.767	2739511452	560271305	100.00	260.07	MS TIC

Match Plot

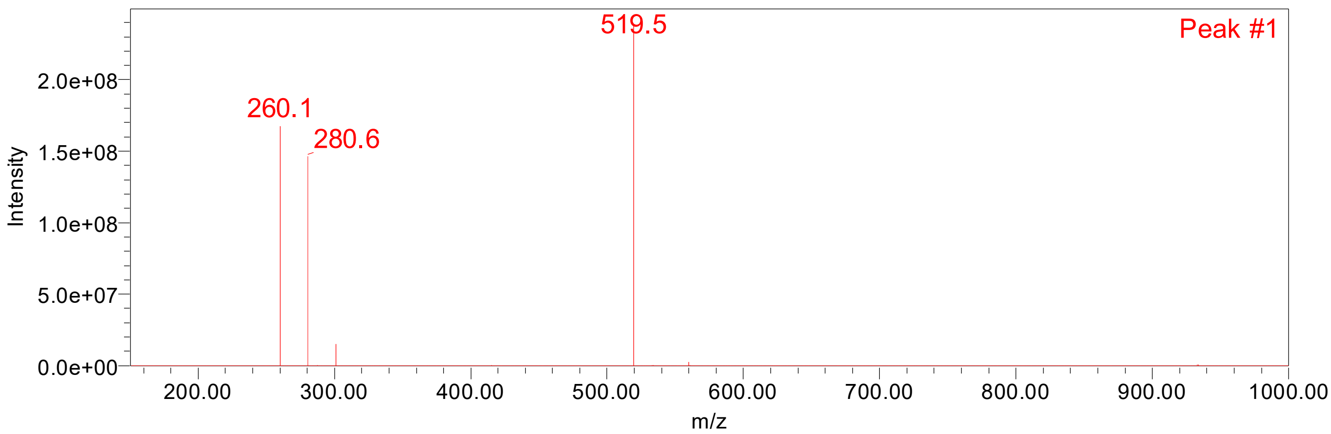

Retention Time 1.263 Channel Name MS TIC
