# Supplementary material for: AI is a viable alternative to high throughput screening: a 318-target study
Source: Sci Rep. 2024 Apr 2;14:7526. doi: 10.1038/s41598-024-54655-z (PMC10987645; doi:10.1038/s41598-024-54655-z)
Supplement: Supplementary file 1 — Supplementary Information 1. [file 41598_2024_54655_MOESM1_ESM.zip › Nature SREP/QC_AIMS_files/Proj094.pdf]

T7313932

MaxPeak: 97.68%
Ret_Time: 0.847 min

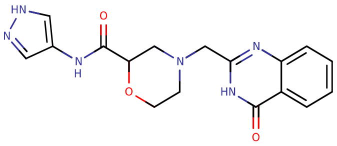

Mol Wt 354.36

Exact Mass 354.15

#	Time	Area%
1	0.847	97.68
2	0.947	2.32

DAD1 A, Sig=215,10 Ref=off (D:\D\06_29\L384981R\SAMPL054.D)

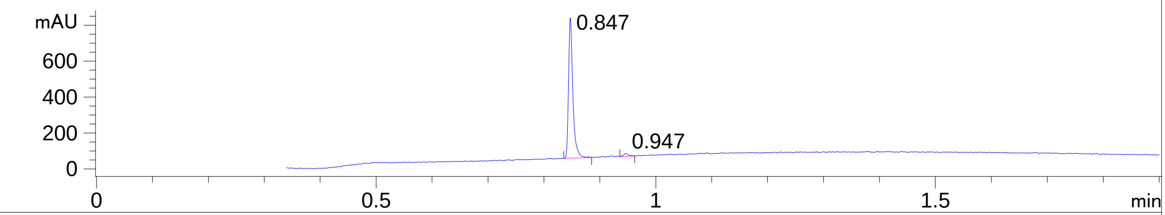

DAD1 B, Sig=254,10 Ref=off (D:\D\06_29\L384981R\SAMPL054.D)

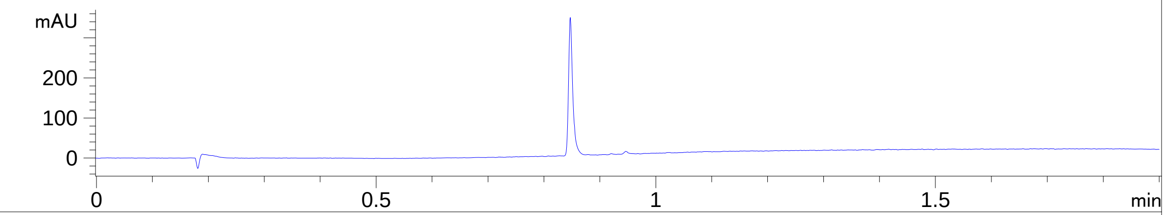

MSD1 TIC, MS File (D:\D\06_29\L384981R\SAMPL054.D) API-ES, Scan, Frag: 120, "Pos"

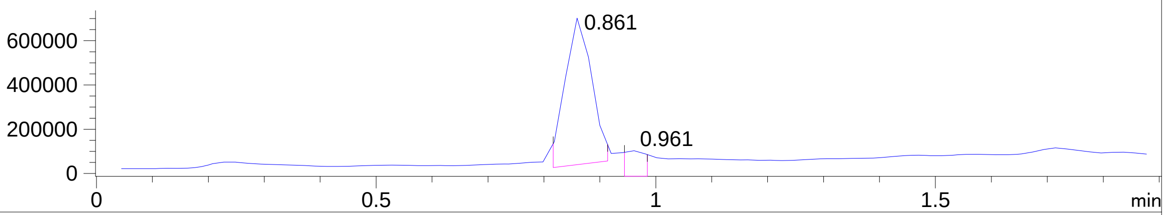

MSD2 TIC, MS File (D:\D\06_29\L384981R\SAMPL054.D) , Scan, Frag: 120, "Neg"

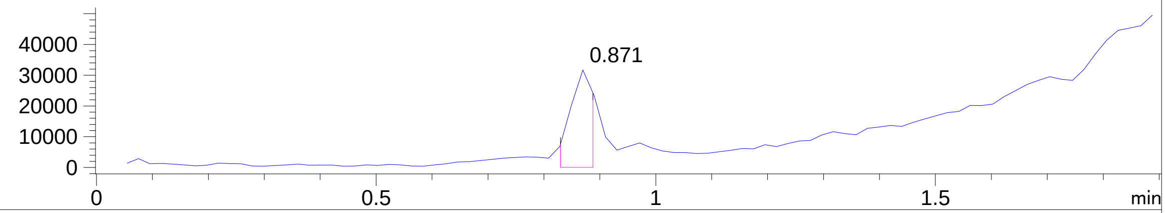

ADC1 A, ADC1 ELSD (D:\D\06_29\L384981R\SAMPL054.D)

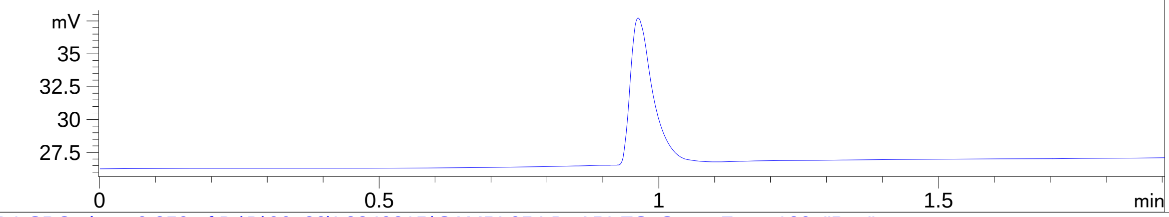

*MSD1 SPC, time=0.859 of D:\D\06_29\L384981R\SAMPL054.D API-ES, Scan, Frag: 120, "Pos"

RT 0.861

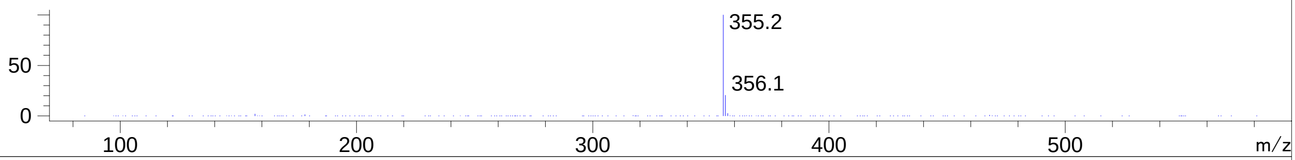

*MSD1 SPC, time=0.961 of D:\D\06_29\L384981R\SAMPL054.D API-ES, Scan, Frag: 120, "Pos"

RT 0.961

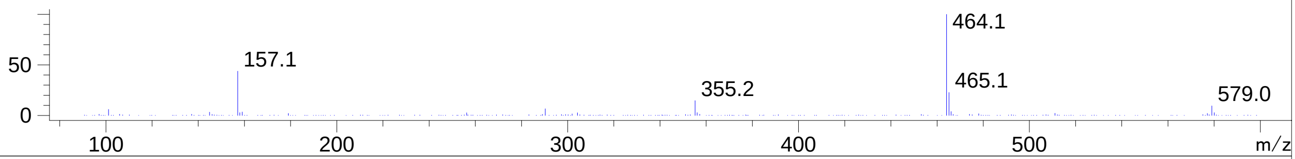

*MSD2 SPC, time=0.869 of D:\D\06_29\L384981R\SAMPL054.D , Scan, Frag: 120, "Neg"

RT 0.871

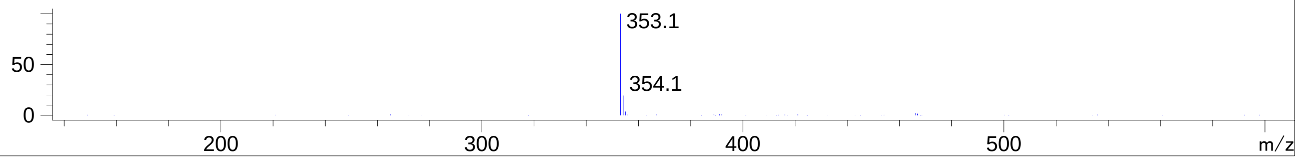

Inj.Date 6/30/2021 N -VL- Acq. Method C:\HPCHEM\-> ->
