# Supplementary material for: AI is a viable alternative to high throughput screening: a 318-target study
Source: Sci Rep. 2024 Apr 2;14:7526. doi: 10.1038/s41598-024-54655-z (PMC10987645; doi:10.1038/s41598-024-54655-z)
Supplement: Supplementary file 1 — Supplementary Information 1. [file 41598_2024_54655_MOESM1_ESM.zip › Nature SREP/QC_AIMS_files/Proj095.pdf]

MaxPeak: 98.89%
Ret_Time: 1.295 min

2322110

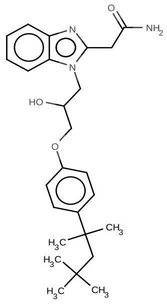

Mol Wt 437.575
Exact Mass 437.32

#	Time	Area%
1	1.295	98.89
2	1.493	1.11

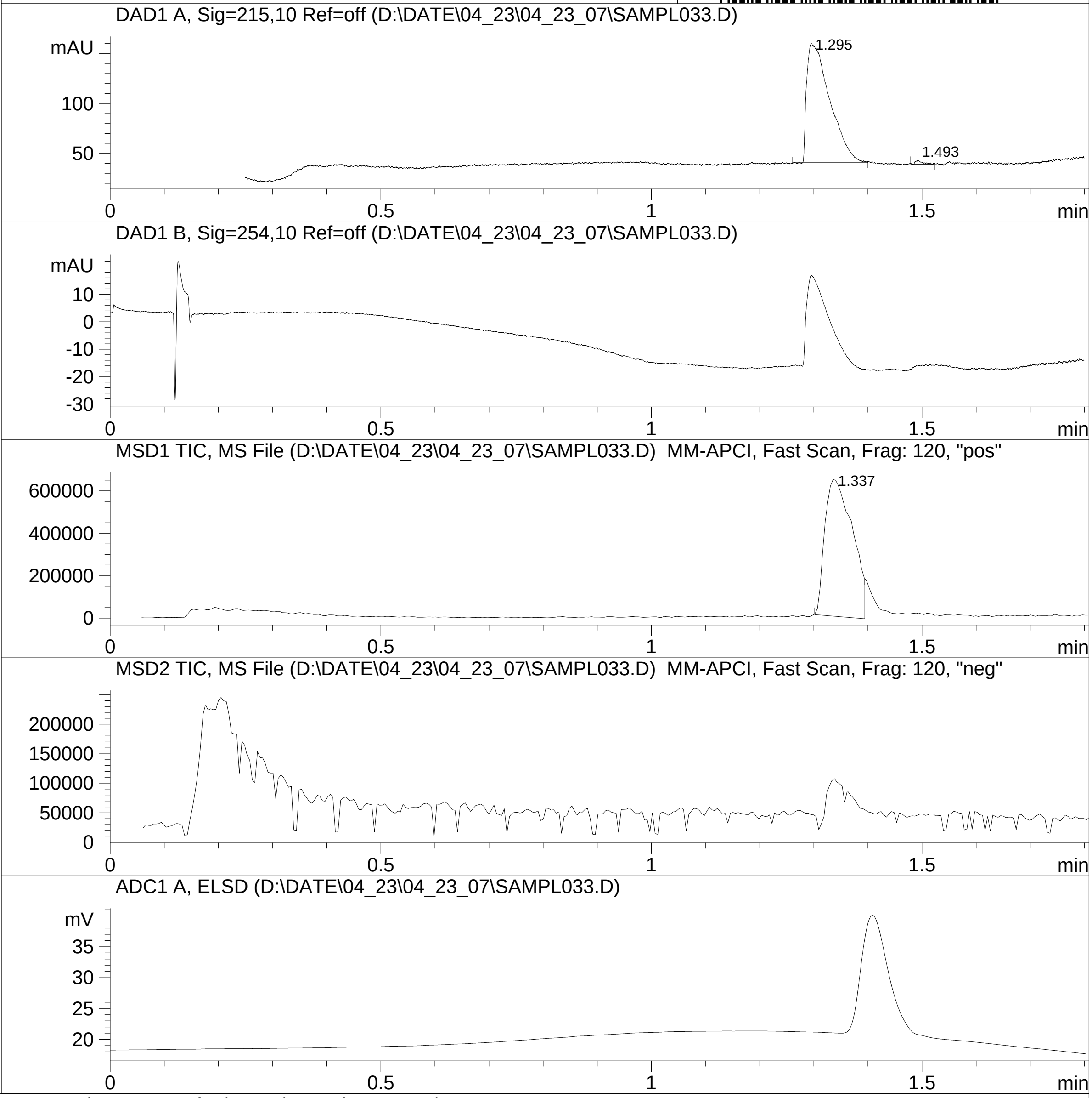

RT 1.337

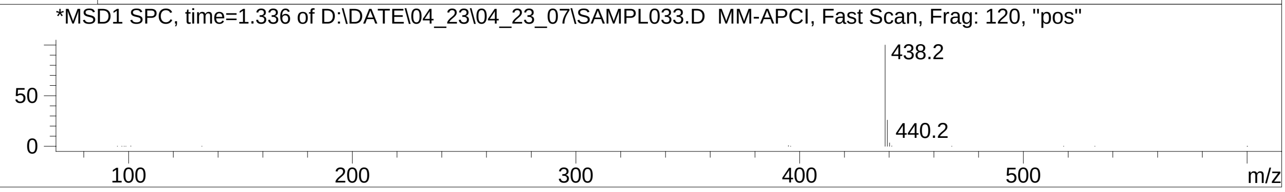

Inj.Date 4/23/2009 O P2-D-06 RR Acq. Method C:\CHEM32\-> ->
