# Supplementary material for: AI is a viable alternative to high throughput screening: a 318-target study
Source: Sci Rep. 2024 Apr 2;14:7526. doi: 10.1038/s41598-024-54655-z (PMC10987645; doi:10.1038/s41598-024-54655-z)
Supplement: Supplementary file 1 — Supplementary Information 1. [file 41598_2024_54655_MOESM1_ESM.zip › Nature SREP/QC_AIMS_files/Proj097.pdf]

T5163634

MaxPeak: 92.90%
Ret_Time: 1.349 min

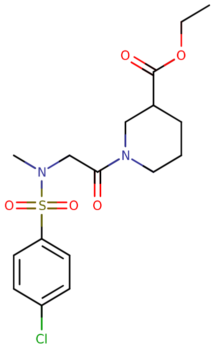

Mol Wt 402.89

Exact Mass 402.12

#	Time	Area%
1	1.079	4.30
2	1.131	1.45
3	1.349	92.90
4	1.471	1.35

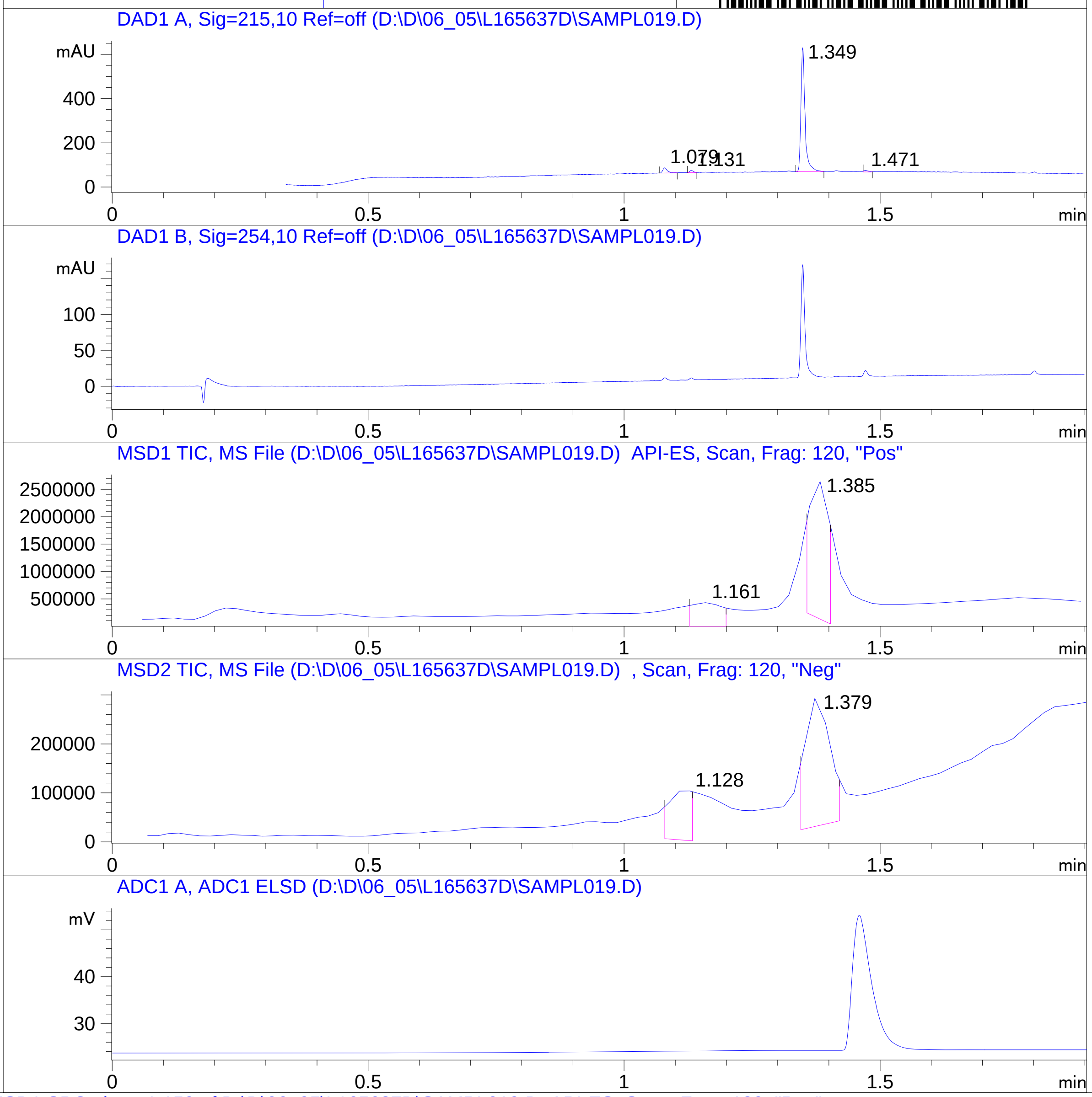

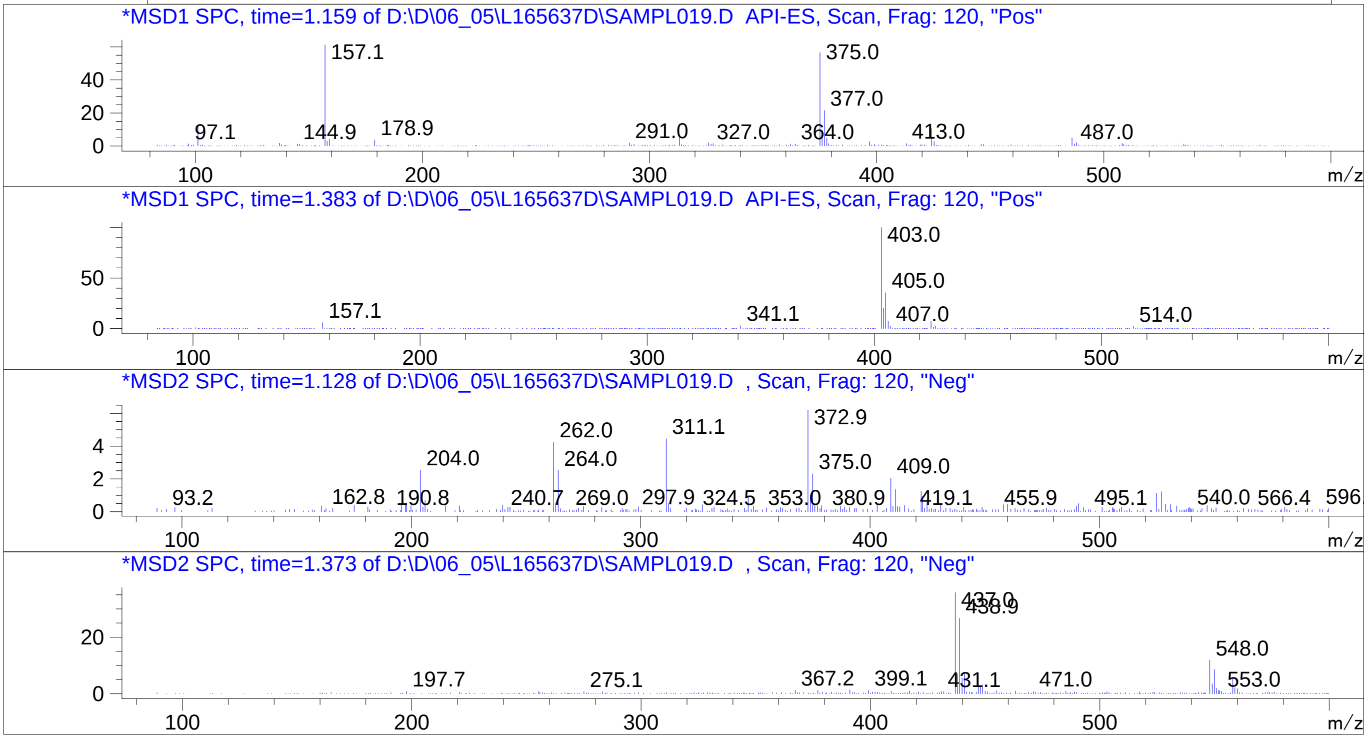

Inj.Date 6/5/2019 N P2-C-01 VL Acq. Method C:\HPCHEM\-> ->
