# Supplementary material for: AI is a viable alternative to high throughput screening: a 318-target study
Source: Sci Rep. 2024 Apr 2;14:7526. doi: 10.1038/s41598-024-54655-z (PMC10987645; doi:10.1038/s41598-024-54655-z)
Supplement: Supplementary file 1 — Supplementary Information 1. [file 41598_2024_54655_MOESM1_ESM.zip › Nature SREP/QC_AIMS_files/Proj100.pdf]

MaxPeak: 94.41%
Ret_Time: 1.178 min

T7433771

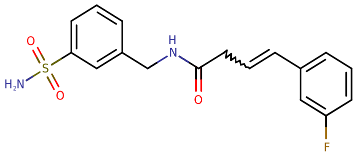

Mol Wt 348.39

Exact Mass 348.11

#	Time	Area%
1	0.925	1.70
2	1.178	94.41
3	1.231	2.36
4	1.352	1.53

DAD1 A, Sig=215,10 Ref=off (D:\DATA\05\0519\L369054R\SAMPL023.D)

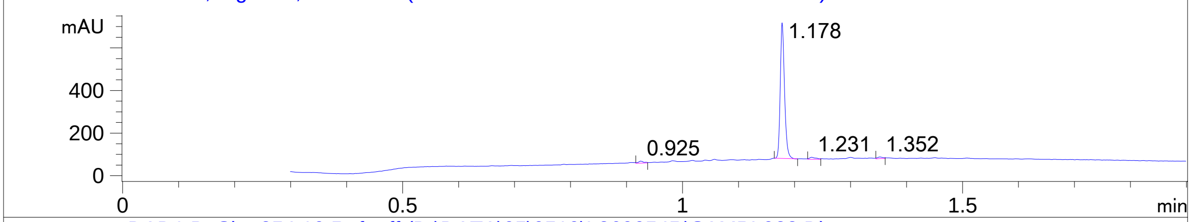

DAD1 B, Sig=254,10 Ref=off (D:\DATA\05\0519\L369054R\SAMPL023.D)

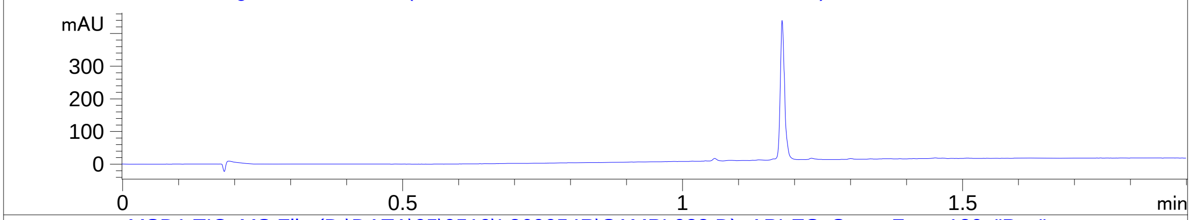

MSD1 TIC, MS File (D:\DATA\05\0519\L369054R\SAMPL023.D) API-ES, Scan, Frag: 120, "Pos"

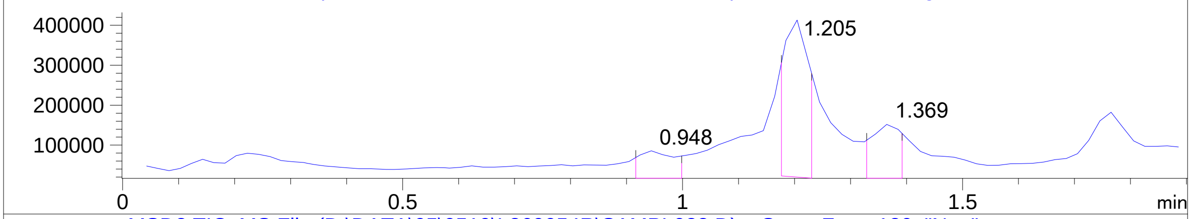

MSD2 TIC, MS File (D:\DATA\05\0519\L369054R\SAMPL023.D) , Scan, Frag: 120, "Neg"

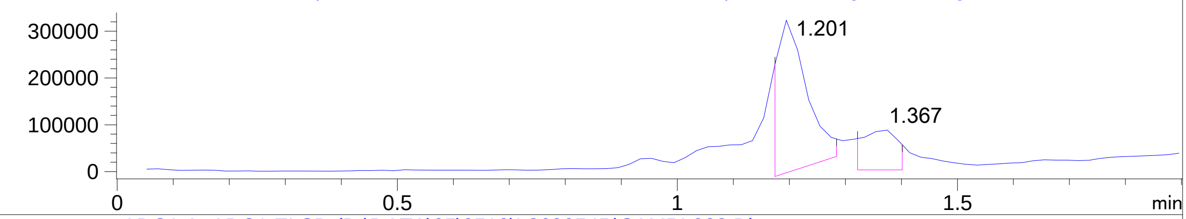

ADC1 A, ADC1 ELSD (D:\DATA\05\0519\L369054R\SAMPL023.D)

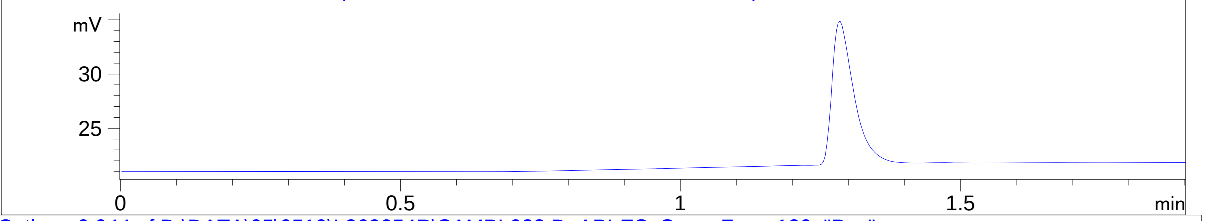

RT 0.948

*MSD1 SPC, time=0.944 of D:\DATA\05\0519\L369054R\SAMPL023.D API-ES, Scan, Frag: 120, "Pos"

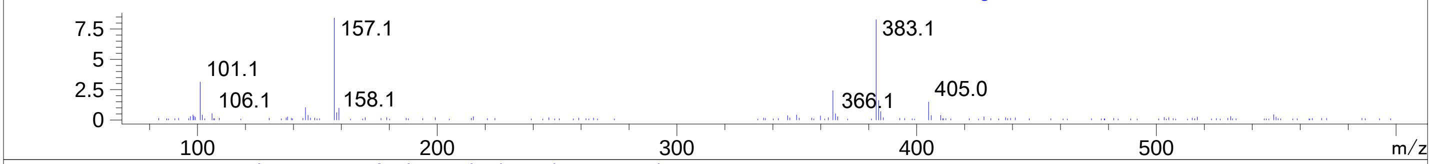

RT 1.205

*MSD1 SPC, time=1.205 of D:\DATA\05\0519\L369054R\SAMPL023.D API-ES, Scan, Frag: 120, "Pos"

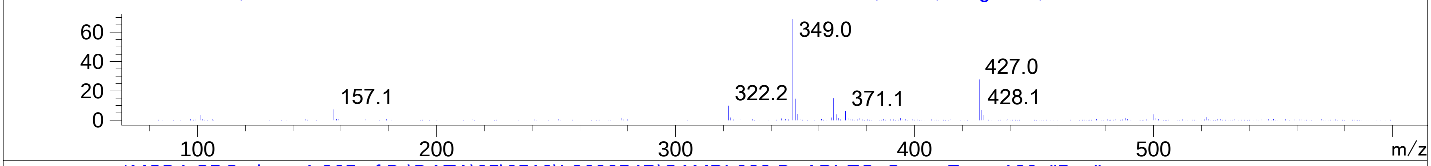

RT 1.369

*MSD1 SPC, time=1.365 of D:\DATA\05\0519\L369054R\SAMPL023.D API-ES, Scan, Frag: 120, "Pos"

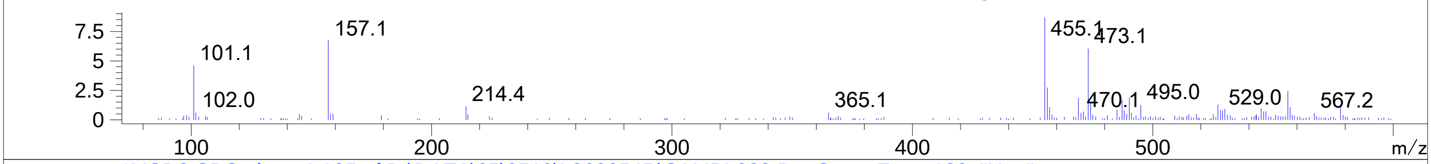

RT 1.201

*MSD2 SPC, time=1.195 of D:\DATA\05\0519\L369054R\SAMPL023.D , Scan, Frag: 120, "Neg"

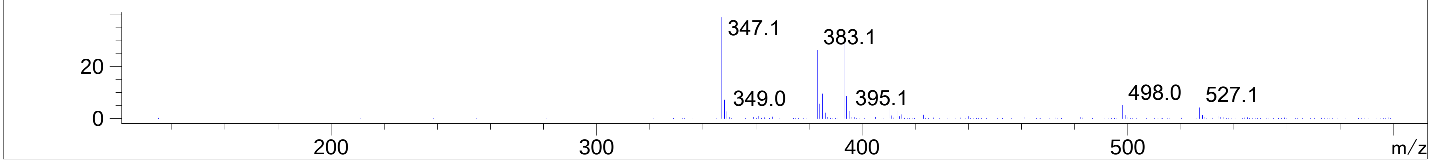

Inj.Date 5/19/2021 LT -SL- Acq. Method C:\HPCHEM\-> ->

RT 1.367

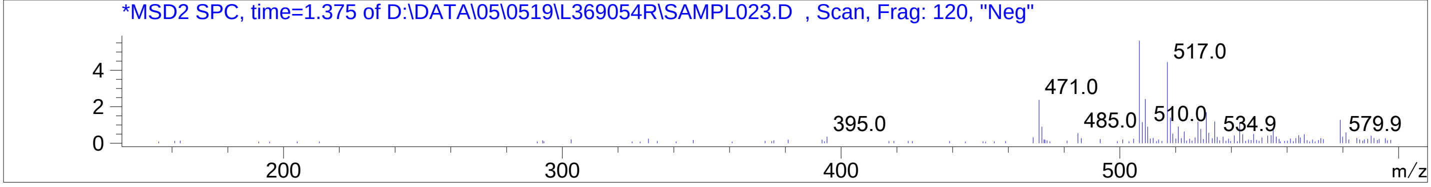

Inj.Date 5/19/2021 LT -SL- Acq. Method C:\HPCHEM\-> ->
