# Supplementary material for: AI is a viable alternative to high throughput screening: a 318-target study
Source: Sci Rep. 2024 Apr 2;14:7526. doi: 10.1038/s41598-024-54655-z (PMC10987645; doi:10.1038/s41598-024-54655-z)
Supplement: Supplementary file 1 — Supplementary Information 1. [file 41598_2024_54655_MOESM1_ESM.zip › Nature SREP/QC_AIMS_files/Proj108.pdf]

T8302868

MaxPeak: 97.22%
Ret_Time: 1.106 min

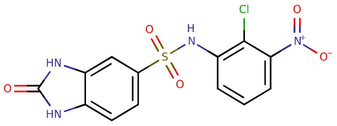

Mol Wt 368.75

Exact Mass 367.98

#	Time	Area%
1	1.106	97.22
2	1.150	2.78

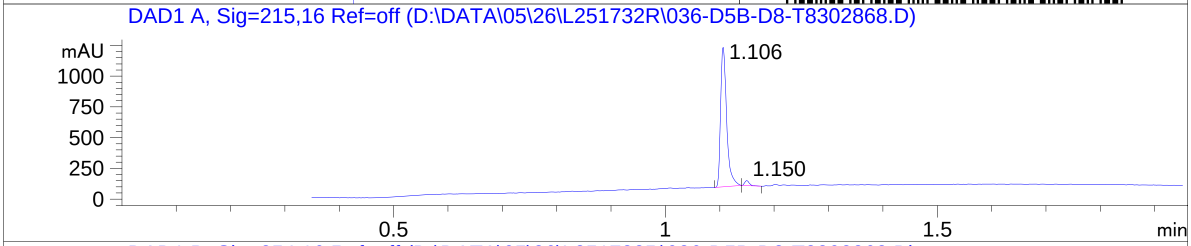

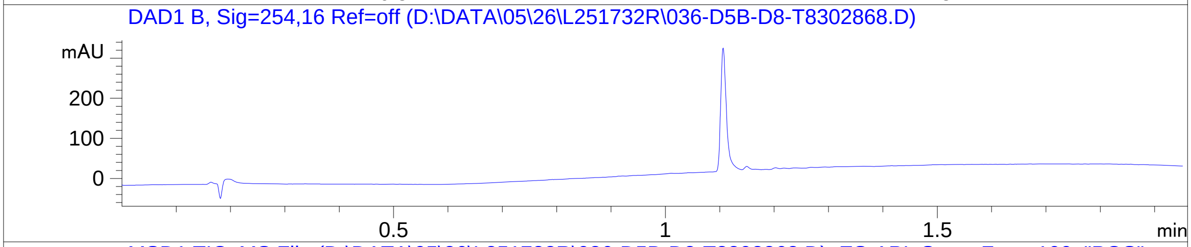

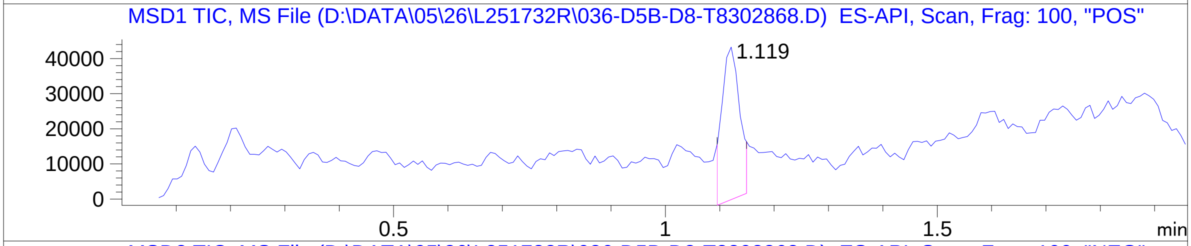

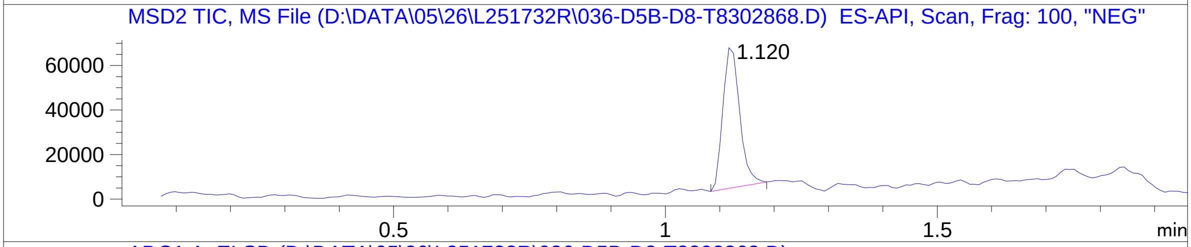

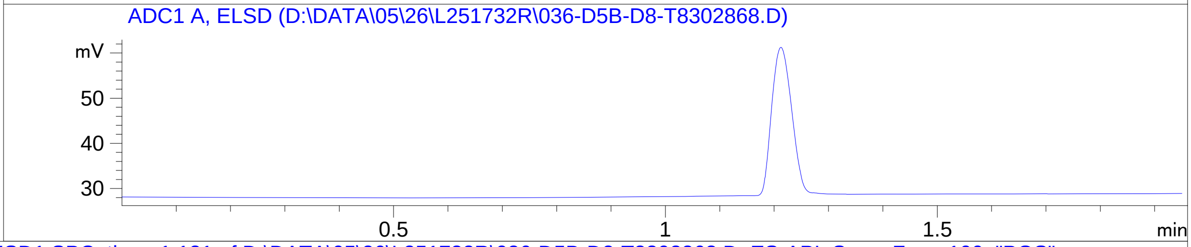

RT 1.119

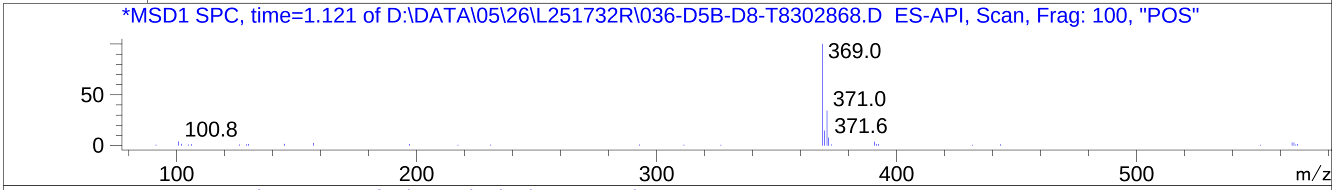

RT 1.120

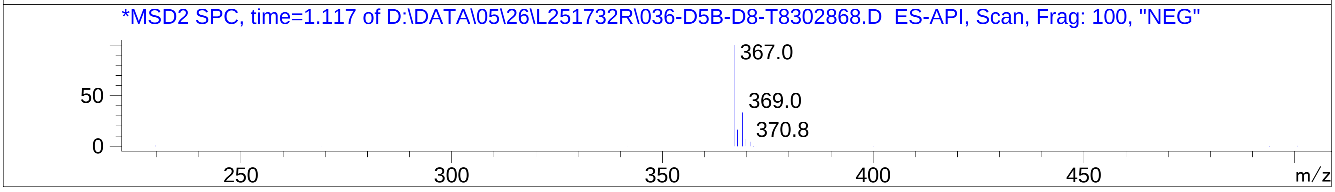

Inj.Date 5/25/2020   LB   Acq. Method C:\Chem32\-> ->
