# Supplementary material for: AI is a viable alternative to high throughput screening: a 318-target study
Source: Sci Rep. 2024 Apr 2;14:7526. doi: 10.1038/s41598-024-54655-z (PMC10987645; doi:10.1038/s41598-024-54655-z)
Supplement: Supplementary file 1 — Supplementary Information 1. [file 41598_2024_54655_MOESM1_ESM.zip › Nature SREP/QC_AIMS_files/Proj110.pdf]

T5320643

MaxPeak: 100.00%
Ret_Time: 1.283 min

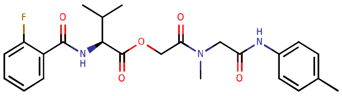

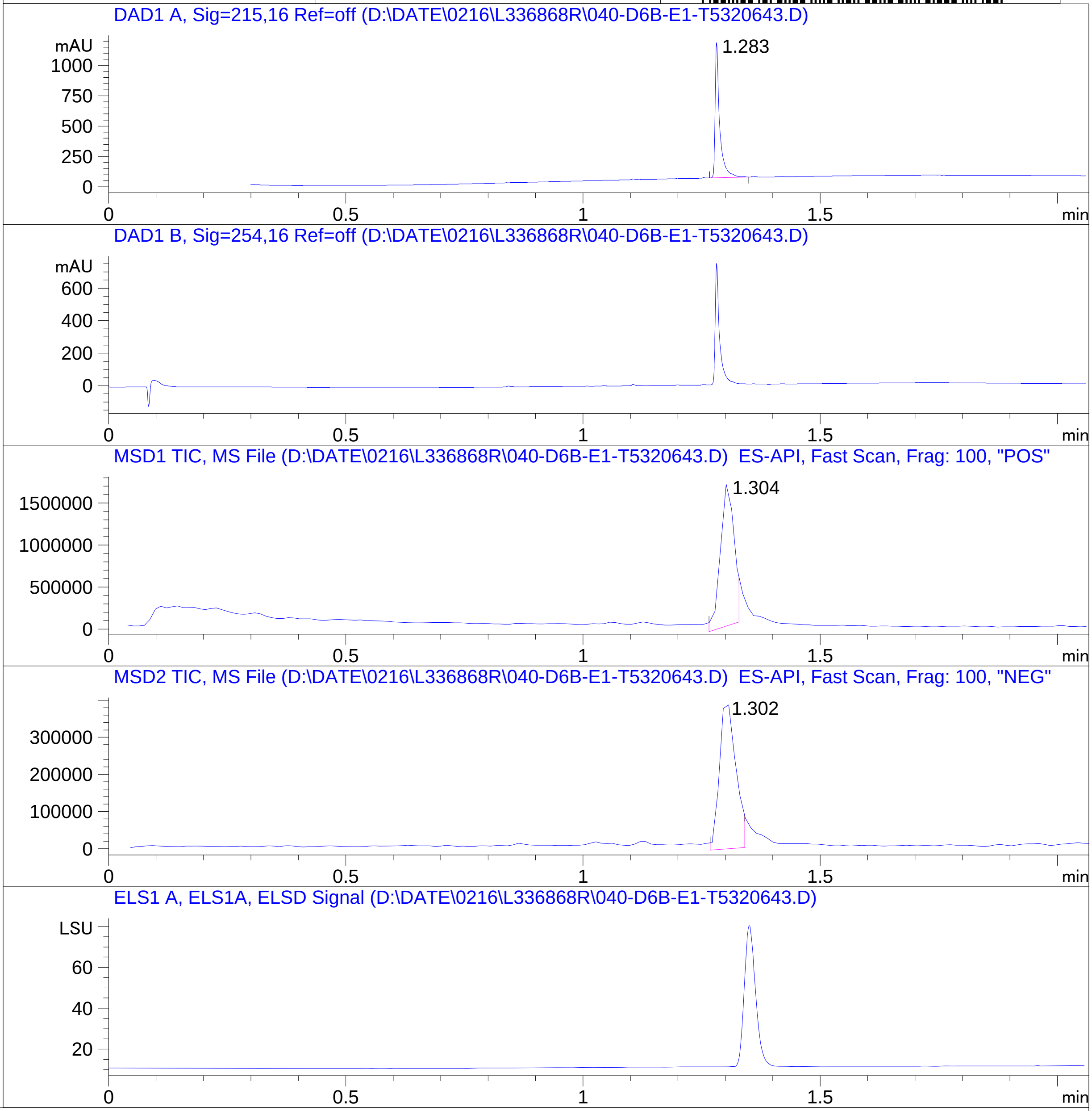

Mol Wt 457.5

Exact Mass 457.23

#	Time	Area%
1	1.283	100.00

RT 1.304

RT 1.302

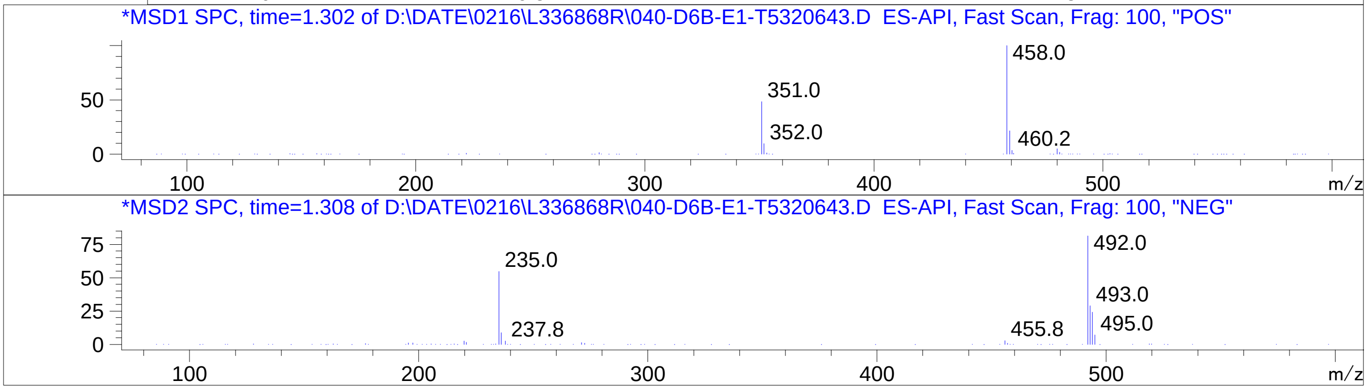

Inj.Date 2/16/2021 O Acq. Method C:\Users\ -> ->
