# Supplementary material for: AI is a viable alternative to high throughput screening: a 318-target study
Source: Sci Rep. 2024 Apr 2;14:7526. doi: 10.1038/s41598-024-54655-z (PMC10987645; doi:10.1038/s41598-024-54655-z)
Supplement: Supplementary file 1 — Supplementary Information 1. [file 41598_2024_54655_MOESM1_ESM.zip › Nature SREP/QC_AIMS_files/Proj111.pdf]

MaxPeak: 95.37%
Ret_Time: 1.017 min

T7202463

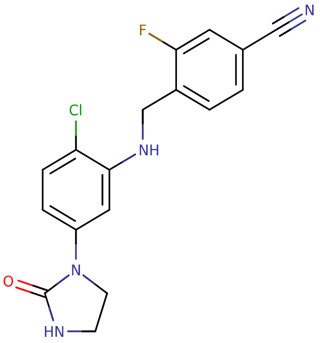

Mol Wt 344.77

Exact Mass 344.1

#	Time	Area%
1	0.666	4.63
2	1.017	95.37

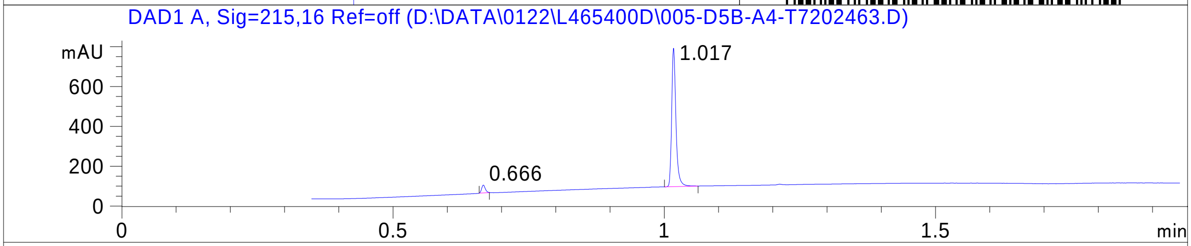

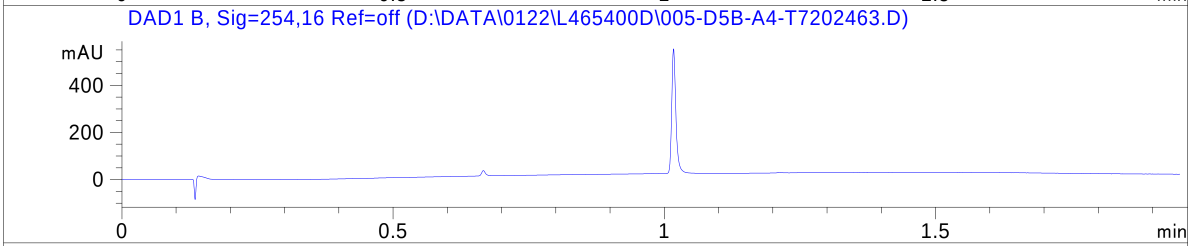

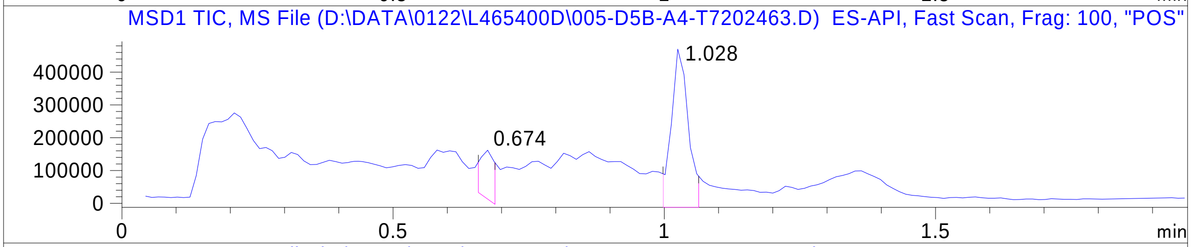

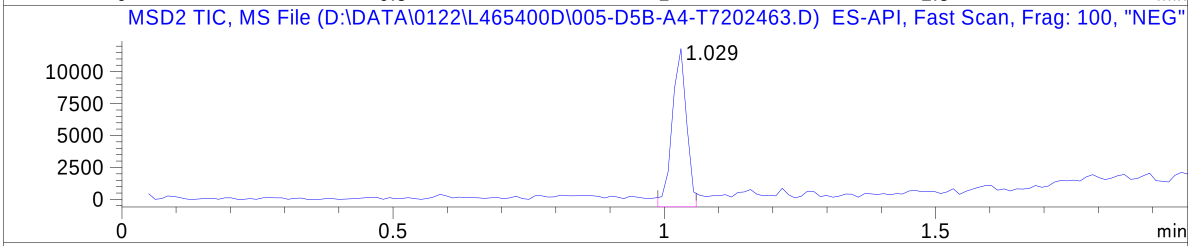

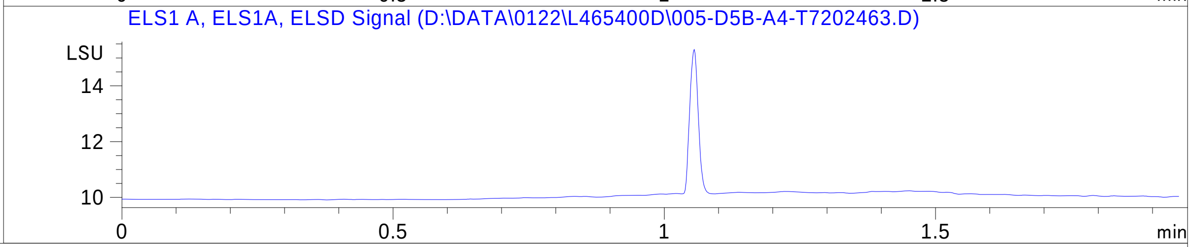

RT 0.674

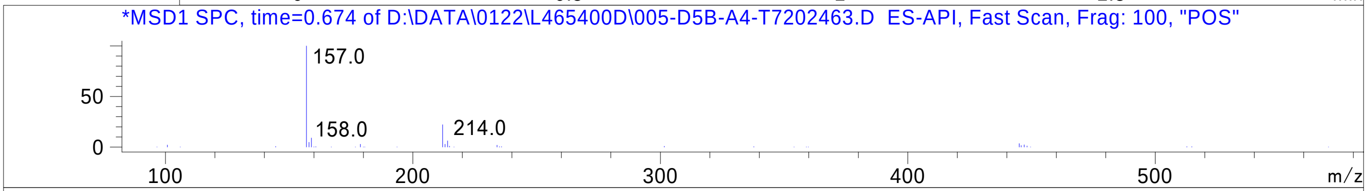

RT 1.028

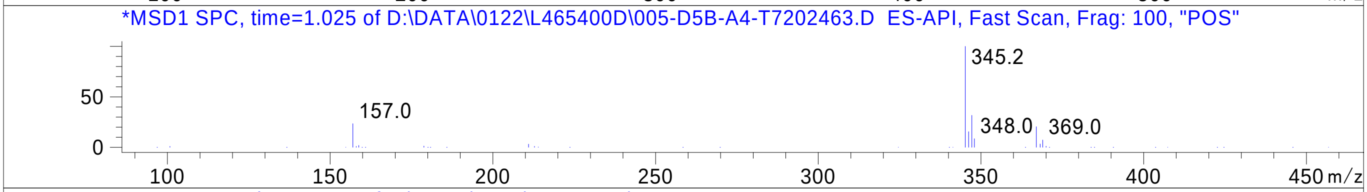

RT 1.029

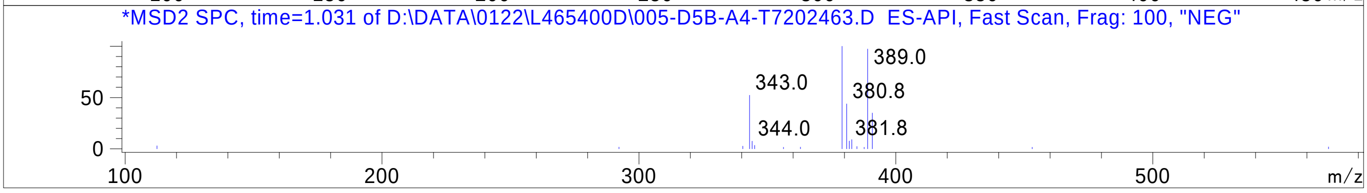

Inj.Date 1/22/2022 H Acq. Method C:\Users\ -> ->
