# Supplementary material for: AI is a viable alternative to high throughput screening: a 318-target study
Source: Sci Rep. 2024 Apr 2;14:7526. doi: 10.1038/s41598-024-54655-z (PMC10987645; doi:10.1038/s41598-024-54655-z)
Supplement: Supplementary file 1 — Supplementary Information 1. [file 41598_2024_54655_MOESM1_ESM.zip › Nature SREP/QC_AIMS_files/Proj119.pdf]

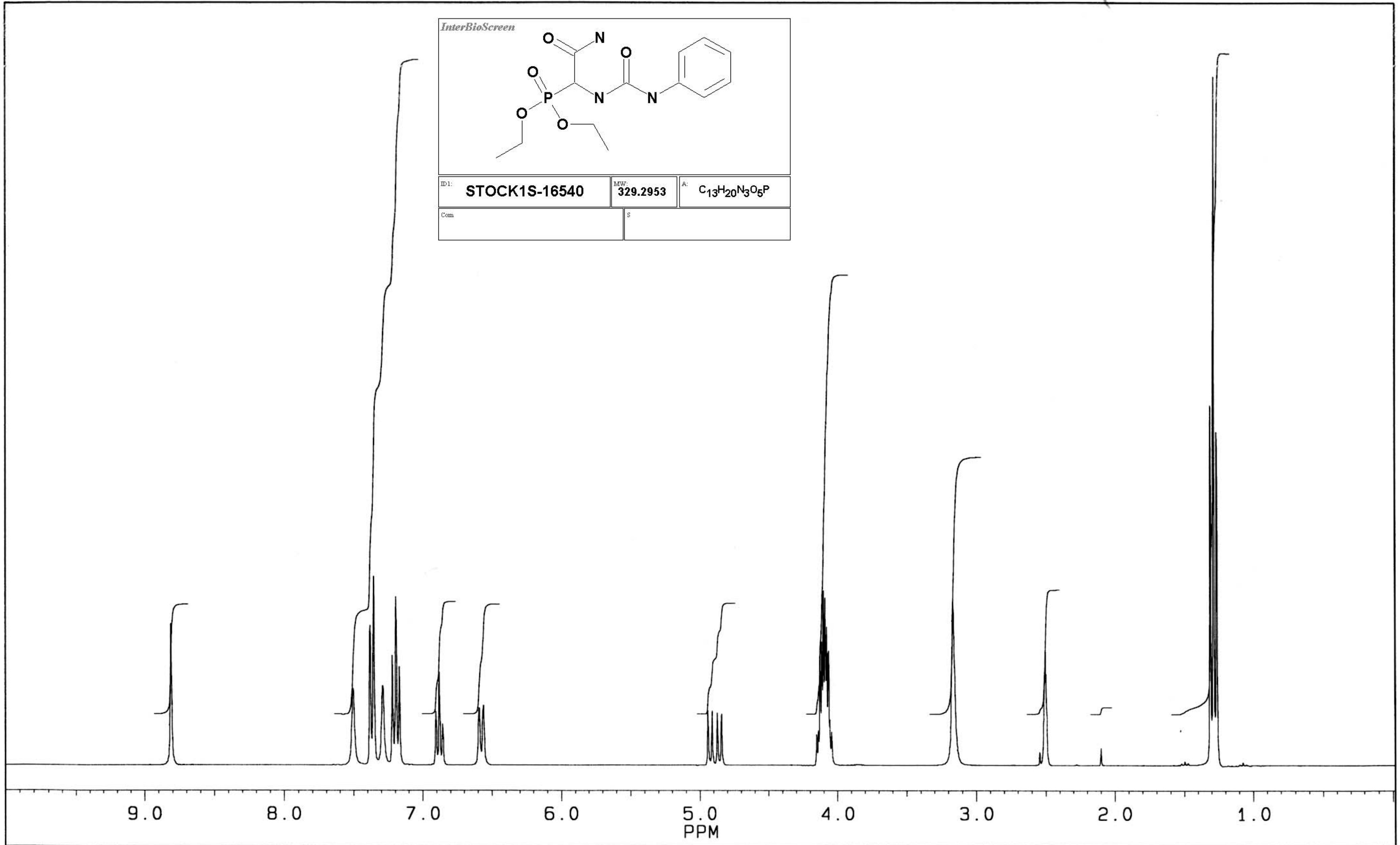

	Bruker AC-300 SF=300.13 MHz	SI=16K, SW=5376, PW=2.5	AQ=1.33, RD=3.0, NS=16	SR=4787.856, TE=313K	Est:
		Opr: VELIKIAN I.V.;	Solv: DMSO;	Prep: G-937;	Exp:
