# Supplementary material for: AI is a viable alternative to high throughput screening: a 318-target study
Source: Sci Rep. 2024 Apr 2;14:7526. doi: 10.1038/s41598-024-54655-z (PMC10987645; doi:10.1038/s41598-024-54655-z)

MaxPeak: 92.58%
Ret_Time: 1.089 min

T7675534

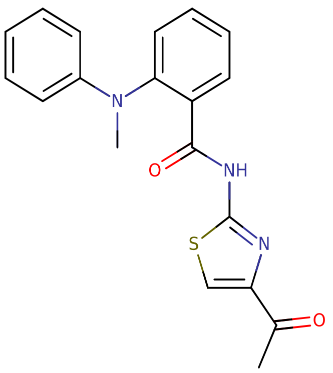

Mol Wt 351.42

Exact Mass 351.12

#	Time	Area%
1	0.954	4.89
2	0.993	2.53
3	1.089	92.58

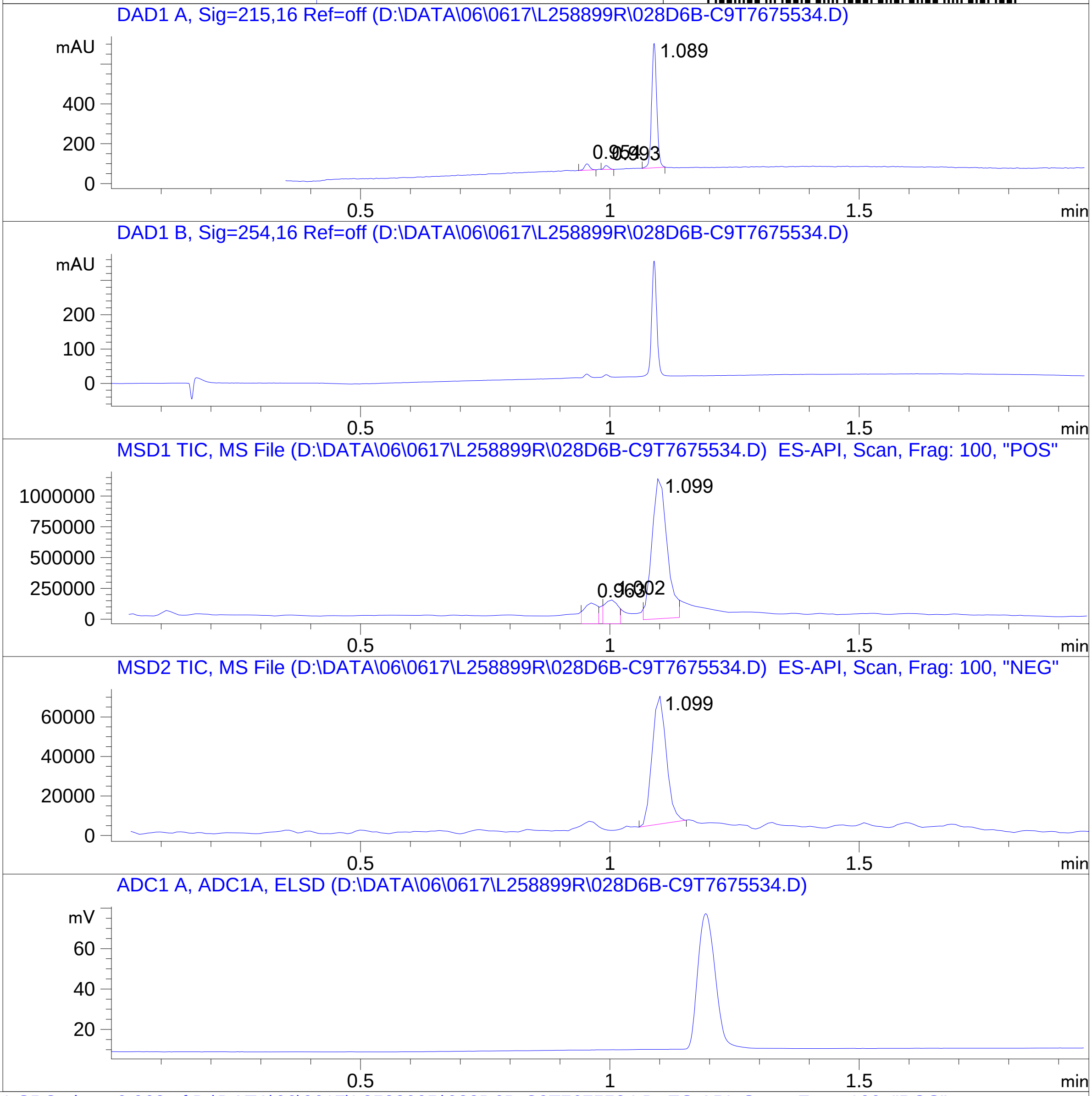

RT 0.963

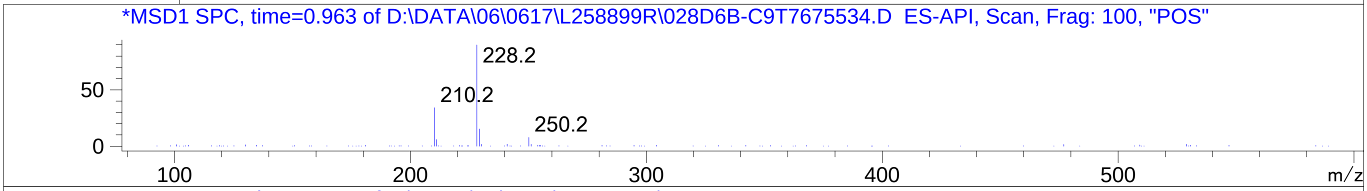

RT 1.002

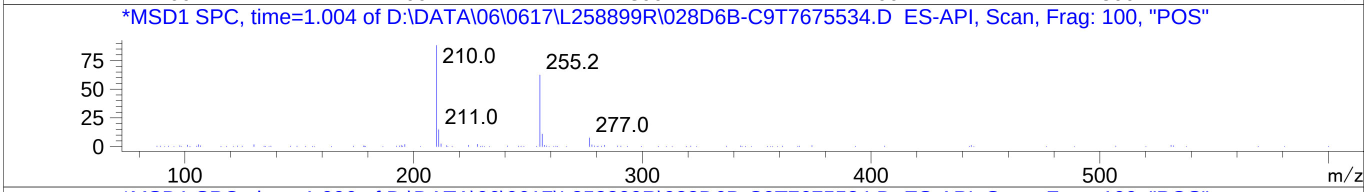

RT 1.099

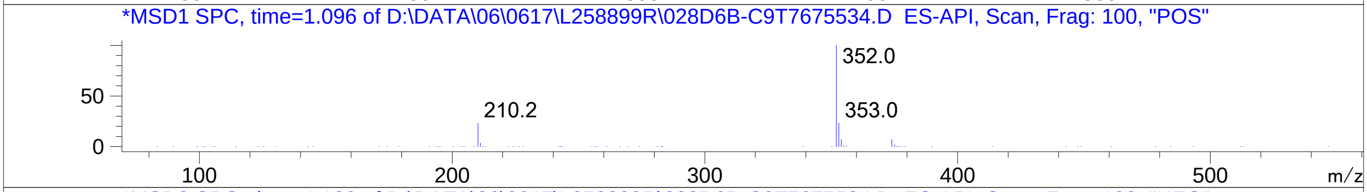

RT 1.099

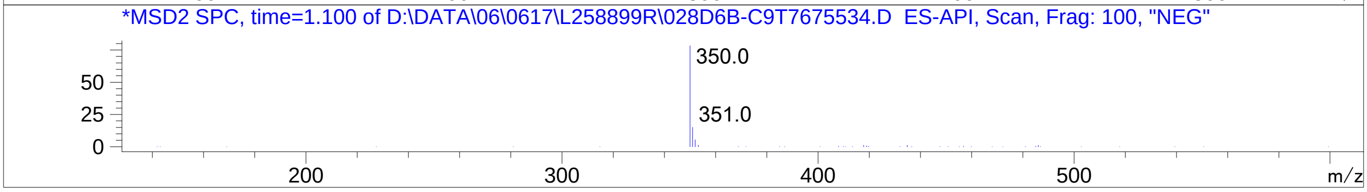

Supplement: Supplementary file 1 — Supplementary Information 1. [file 41598_2024_54655_MOESM1_ESM.zip › Nature SREP/QC_AIMS_files/Proj123.pdf]
