# Supplementary material for: AI is a viable alternative to high throughput screening: a 318-target study
Source: Sci Rep. 2024 Apr 2;14:7526. doi: 10.1038/s41598-024-54655-z (PMC10987645; doi:10.1038/s41598-024-54655-z)
Supplement: Supplementary file 1 — Supplementary Information 1. [file 41598_2024_54655_MOESM1_ESM.zip › Nature SREP/QC_AIMS_files/Proj124.pdf]

MaxPeak: 100.00%
Ret_Time: 1.347 min

5396534$2

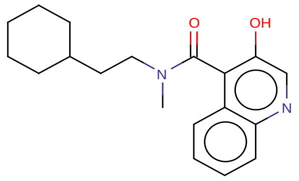

**Mol Wt**	**312.41**
**Exact Mass**	**312.22**

#	Time	Area%
1	1.347	100.00

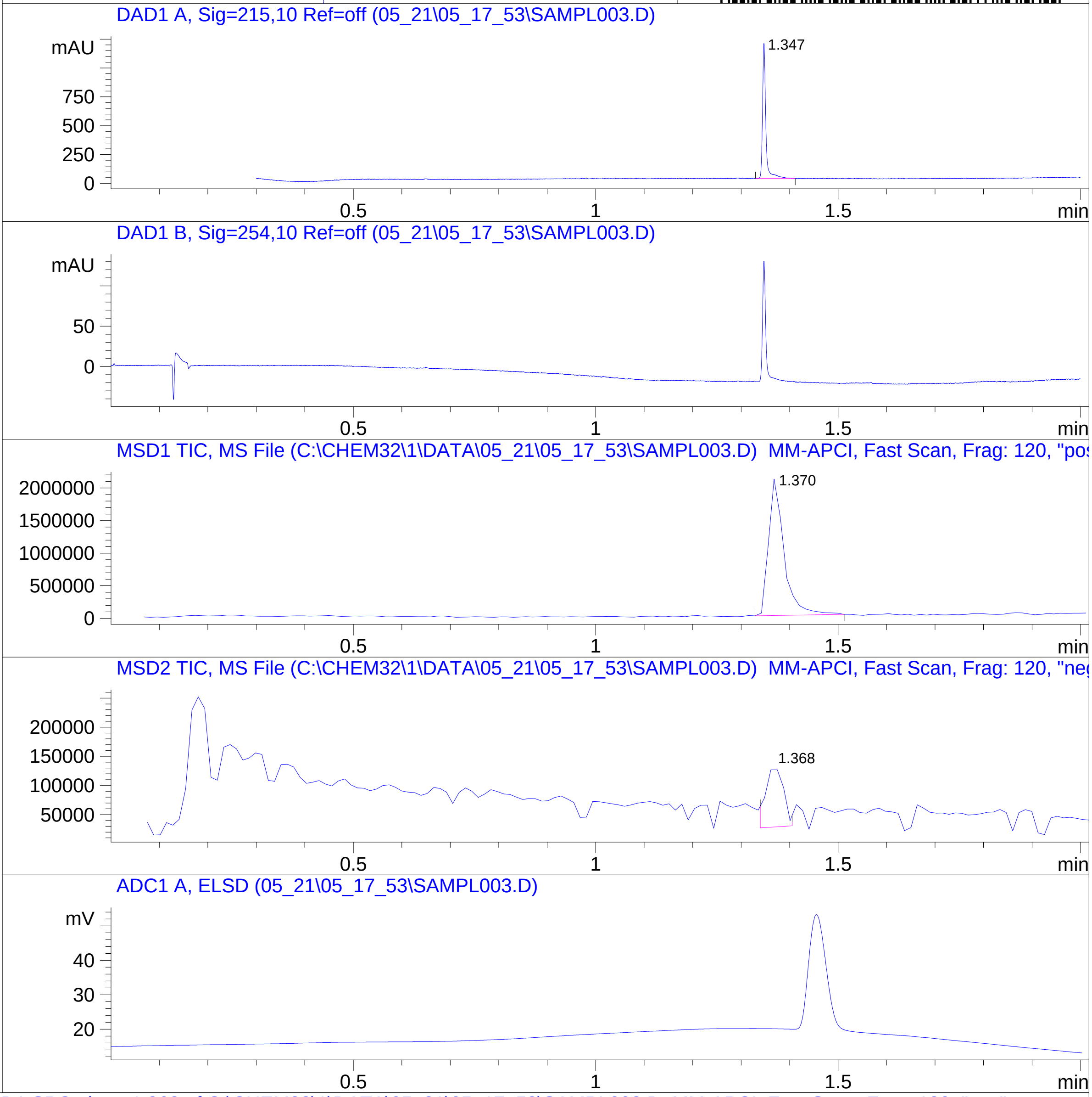

RT 1.370

RT 1.368

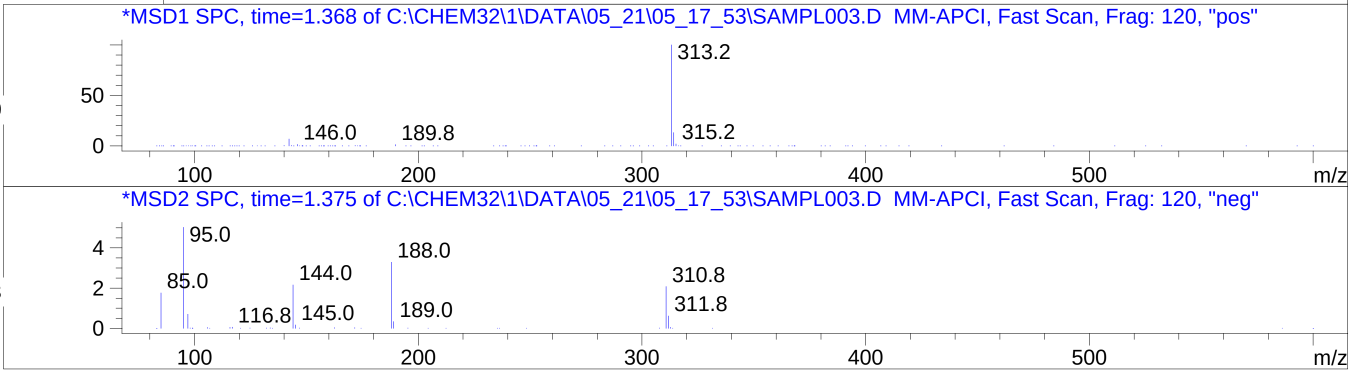

Inj.Date 5/20/2012 N P2-A-03 RR Acq. Method C:\CHEM32\-> ->
