# Supplementary material for: AI is a viable alternative to high throughput screening: a 318-target study
Source: Sci Rep. 2024 Apr 2;14:7526. doi: 10.1038/s41598-024-54655-z (PMC10987645; doi:10.1038/s41598-024-54655-z)
Supplement: Supplementary file 1 — Supplementary Information 1. [file 41598_2024_54655_MOESM1_ESM.zip › Nature SREP/QC_AIMS_files/Proj134.pdf]

```
MaxPeak:  100.00%
Ret_Time: 1.410 min
```

T6474255

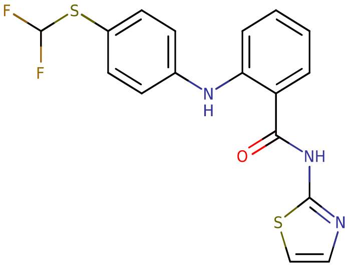

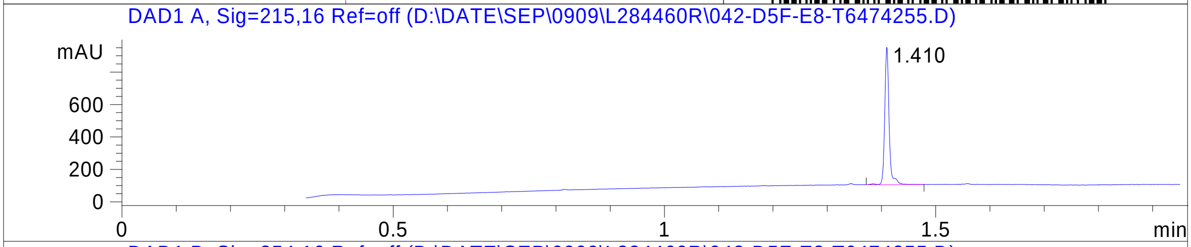

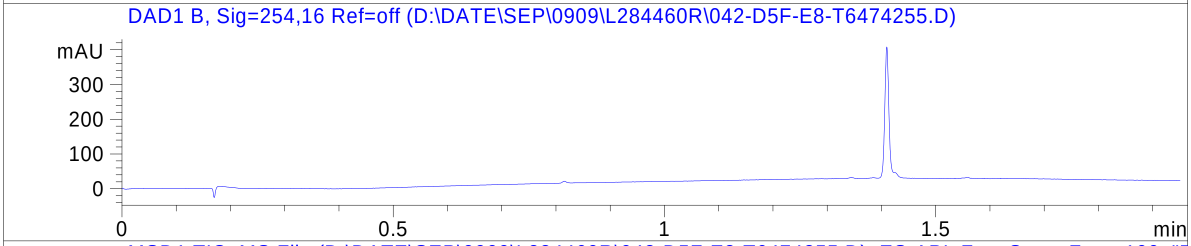

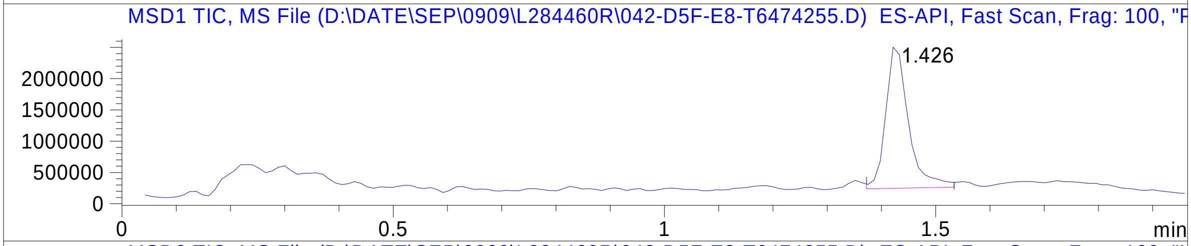

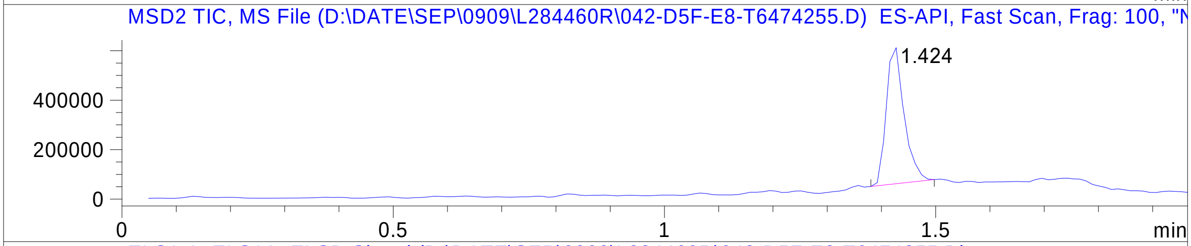

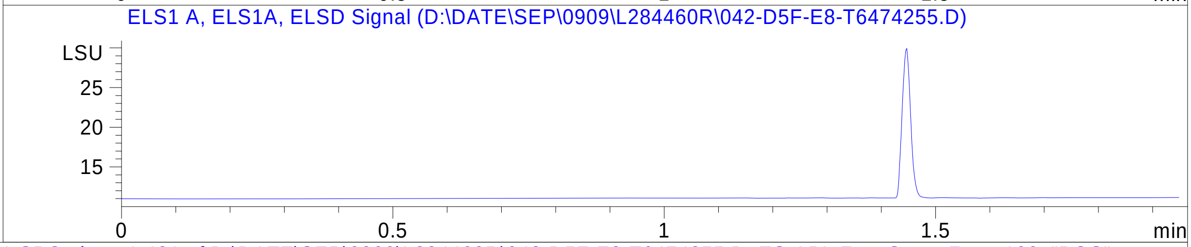

Mol Wt 377.43

Exact Mass 377.06

```
#  Time    Area%
------------------
1  1.410  100.00
```

RT 1.426

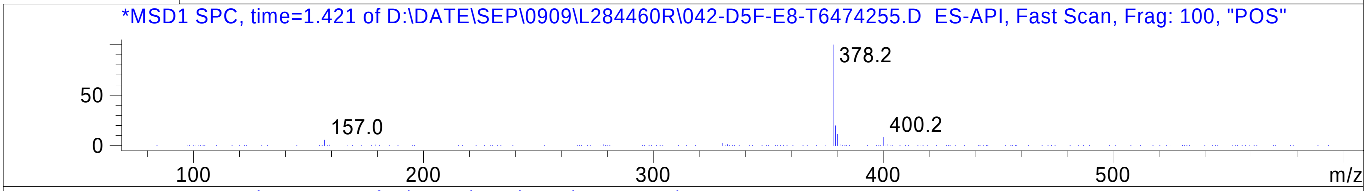

RT 1.424

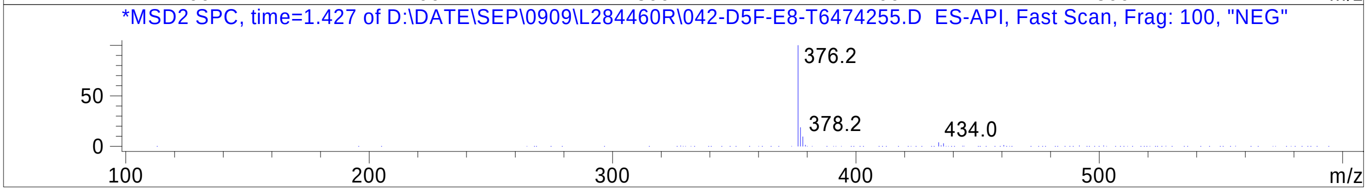


```
Inj.Date 9/8/2020          Y          Acq. Method C:\Users\ ->    ->
```
