# Supplementary material for: AI is a viable alternative to high throughput screening: a 318-target study
Source: Sci Rep. 2024 Apr 2;14:7526. doi: 10.1038/s41598-024-54655-z (PMC10987645; doi:10.1038/s41598-024-54655-z)
Supplement: Supplementary file 1 — Supplementary Information 1. [file 41598_2024_54655_MOESM1_ESM.zip › Nature SREP/QC_AIMS_files/Proj136.pdf]

MaxPeak: 94.45%
Ret_Time: 1.251 min

T7729171

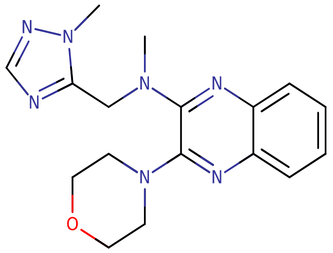

Mol Wt 339.4

Exact Mass 339.2

#	Time	Area%
1	1.074	5.55
2	1.251	94.45

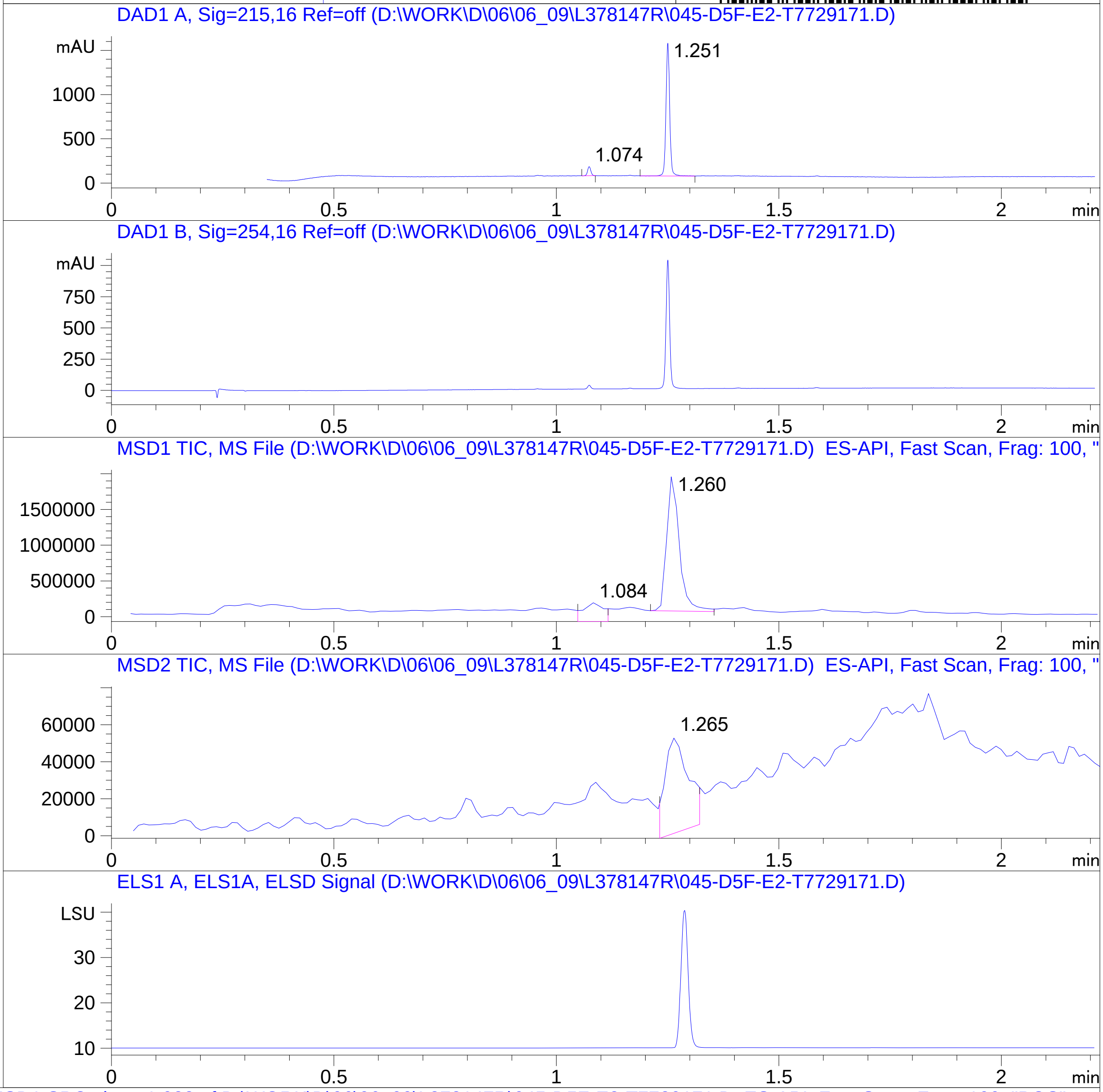

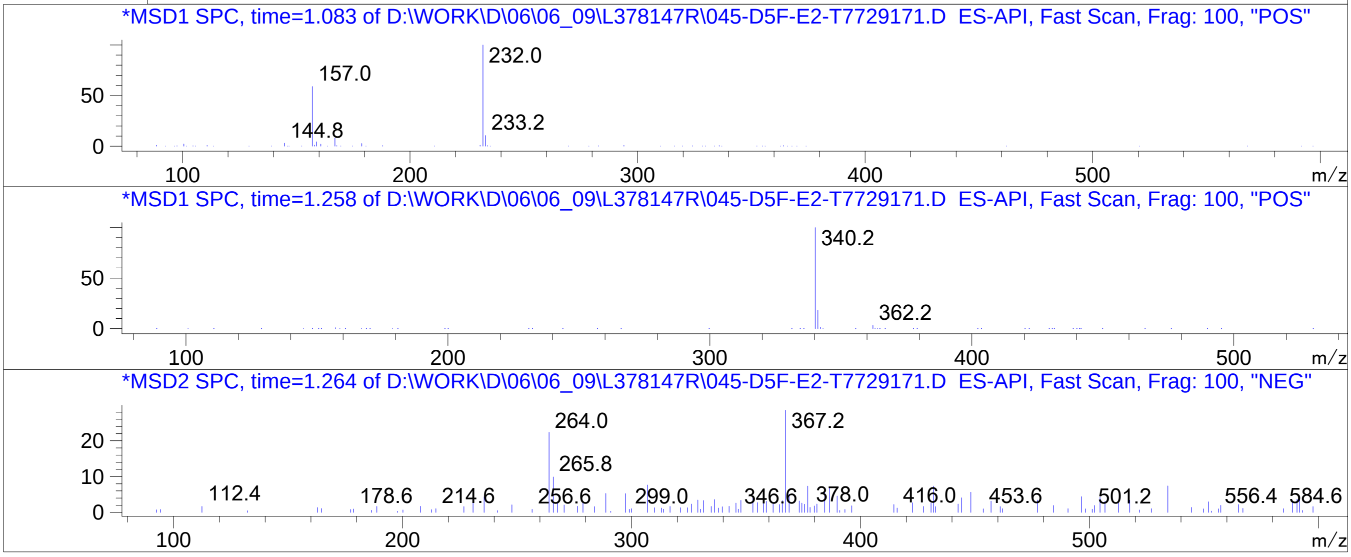

Inj.Date 6/9/2021 K Acq. Method C:\Users\ -> ->
