# Supplementary material for: AI is a viable alternative to high throughput screening: a 318-target study
Source: Sci Rep. 2024 Apr 2;14:7526. doi: 10.1038/s41598-024-54655-z (PMC10987645; doi:10.1038/s41598-024-54655-z)
Supplement: Supplementary file 1 — Supplementary Information 1. [file 41598_2024_54655_MOESM1_ESM.zip › Nature SREP/QC_AIMS_files/Proj143.pdf]

MaxPeak: 96.75%
Ret_Time: 1.261 min

6765448

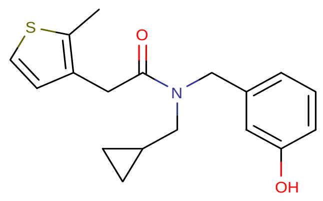

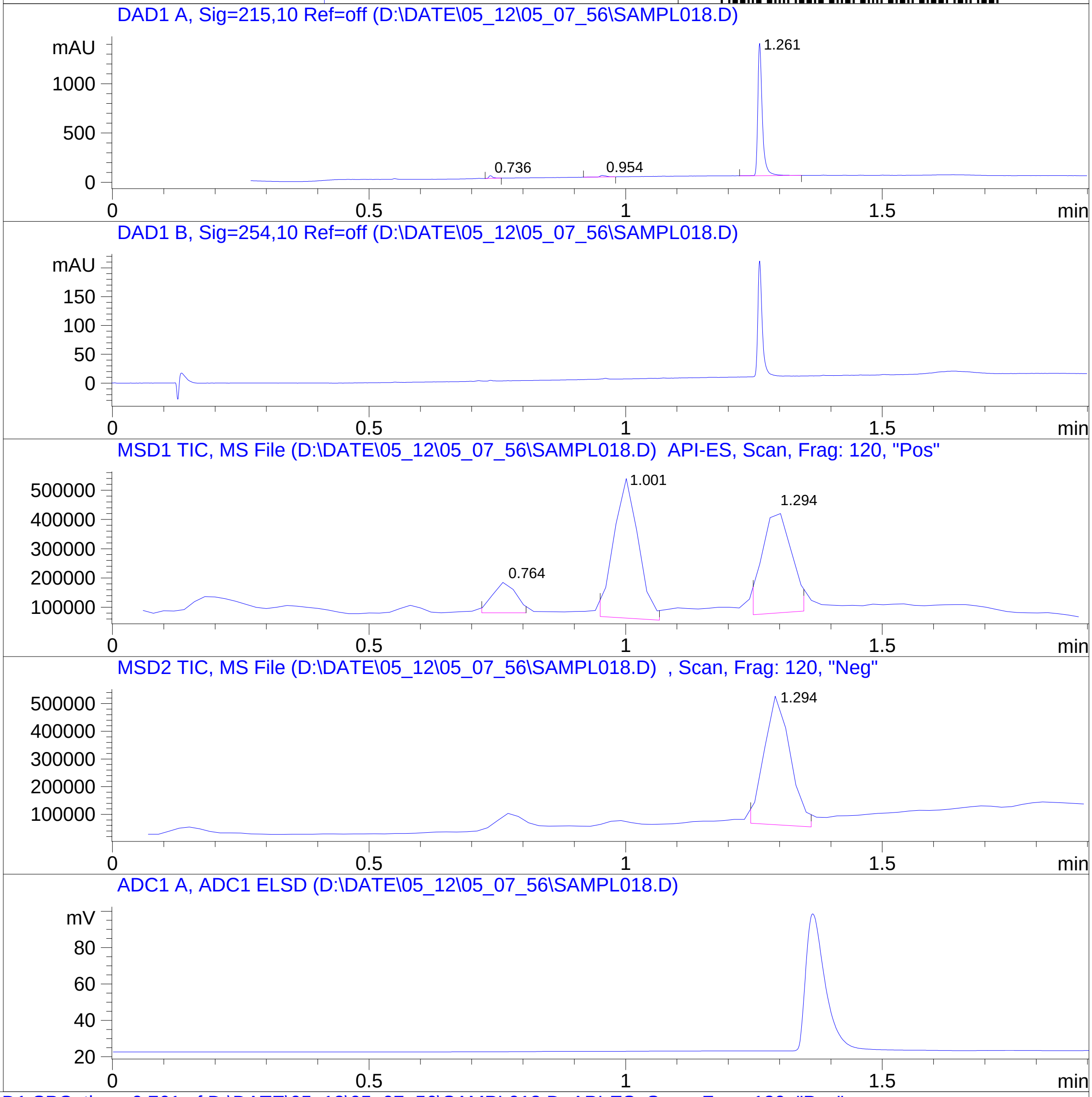

Mol Wt 315.43
Exact Mass 315.16

#	Time	Area%
1	0.736	1.65
2	0.954	1.60
3	1.261	96.75

RT 0.764

RT 1.001

RT 1.294

RT 1.294

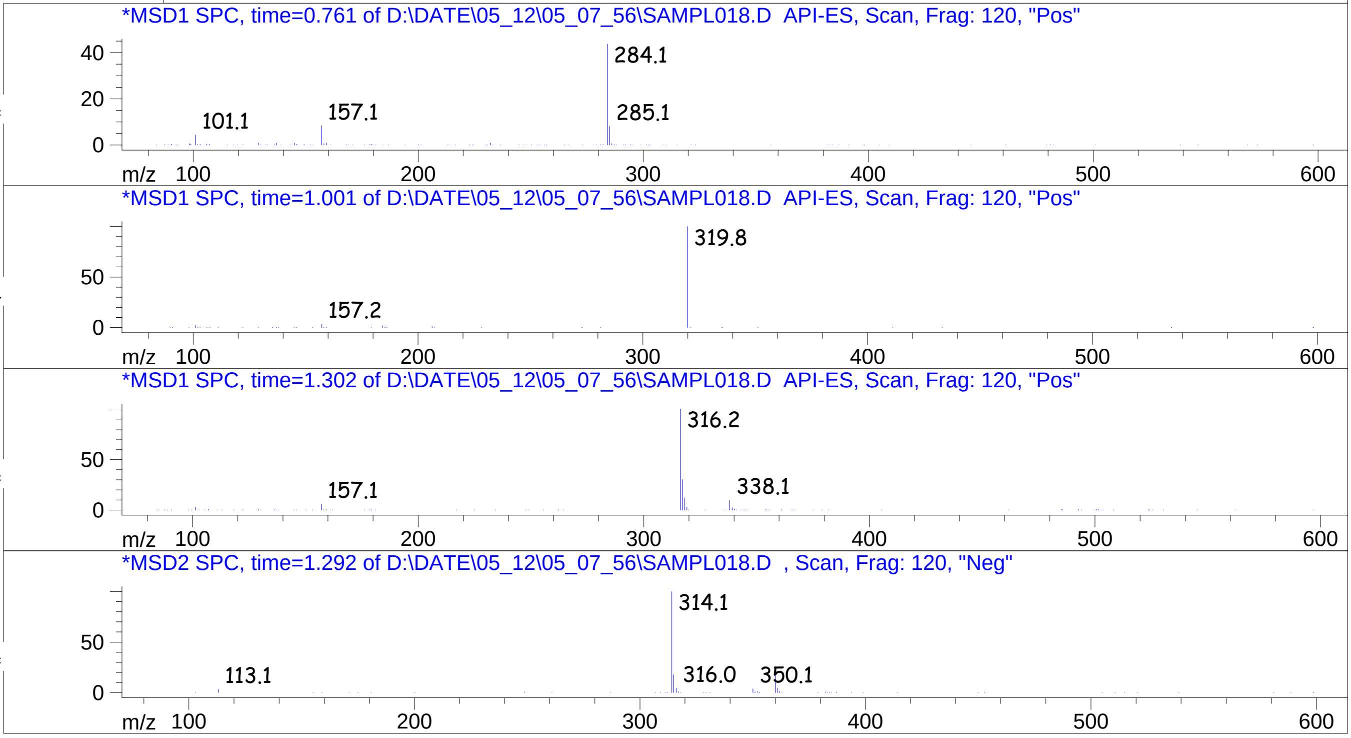

Inj.Date 5/10/2014 O P2-C-02 VL Acq. Method C:\HPCHEM\-> ->
