# Supplementary material for: AI is a viable alternative to high throughput screening: a 318-target study
Source: Sci Rep. 2024 Apr 2;14:7526. doi: 10.1038/s41598-024-54655-z (PMC10987645; doi:10.1038/s41598-024-54655-z)
Supplement: Supplementary file 1 — Supplementary Information 1. [file 41598_2024_54655_MOESM1_ESM.zip › Nature SREP/QC_AIMS_files/Proj145.pdf]

MaxPeak: 93.70%
Ret_Time: 1.261 min

T5497027

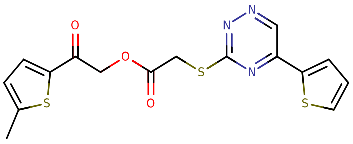

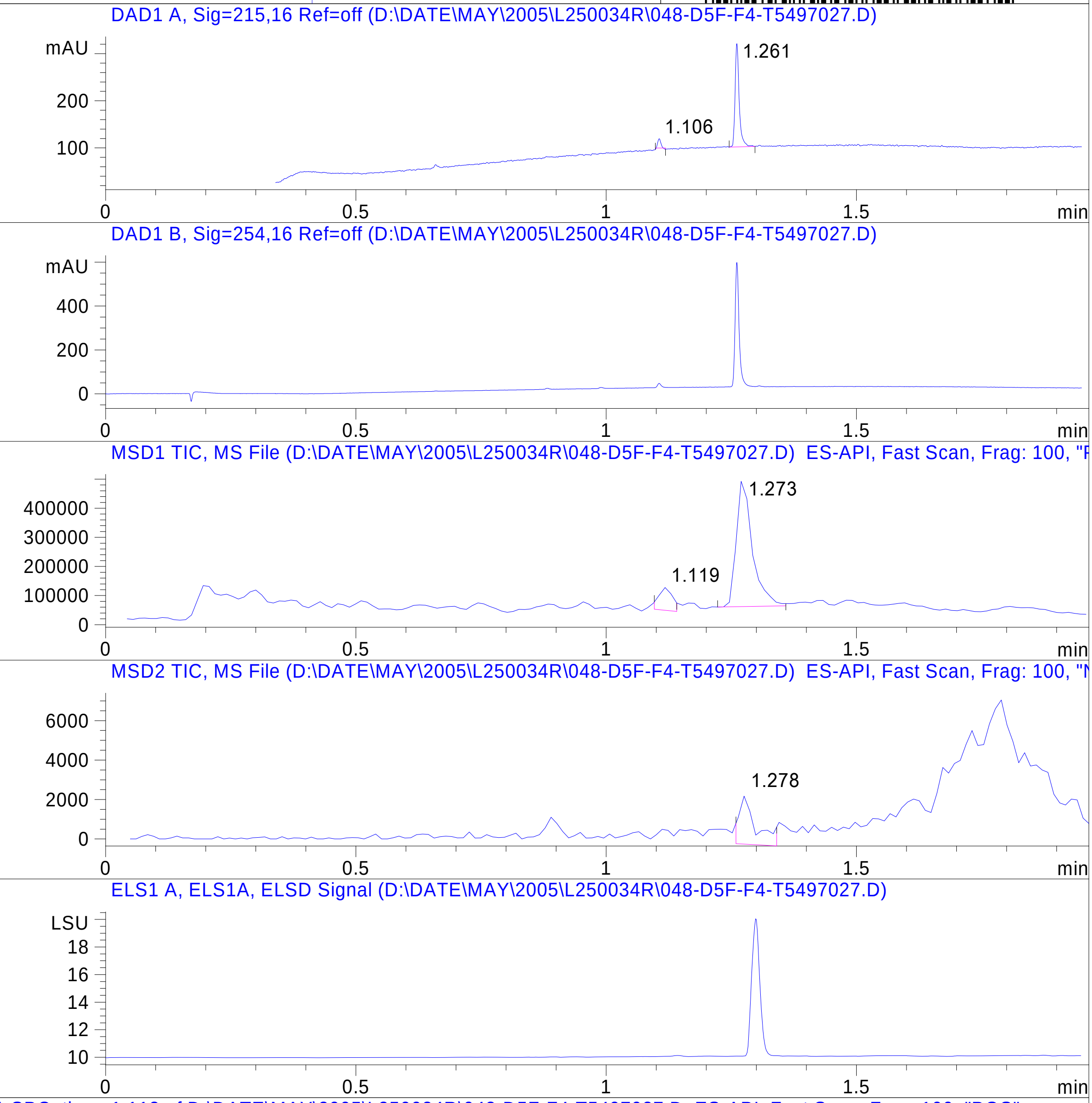

Mol Wt 391.49

Exact Mass 391.01

#	Time	Area%
1	1.106	6.30
2	1.261	93.70

RT 1.119

RT 1.273

RT 1.278

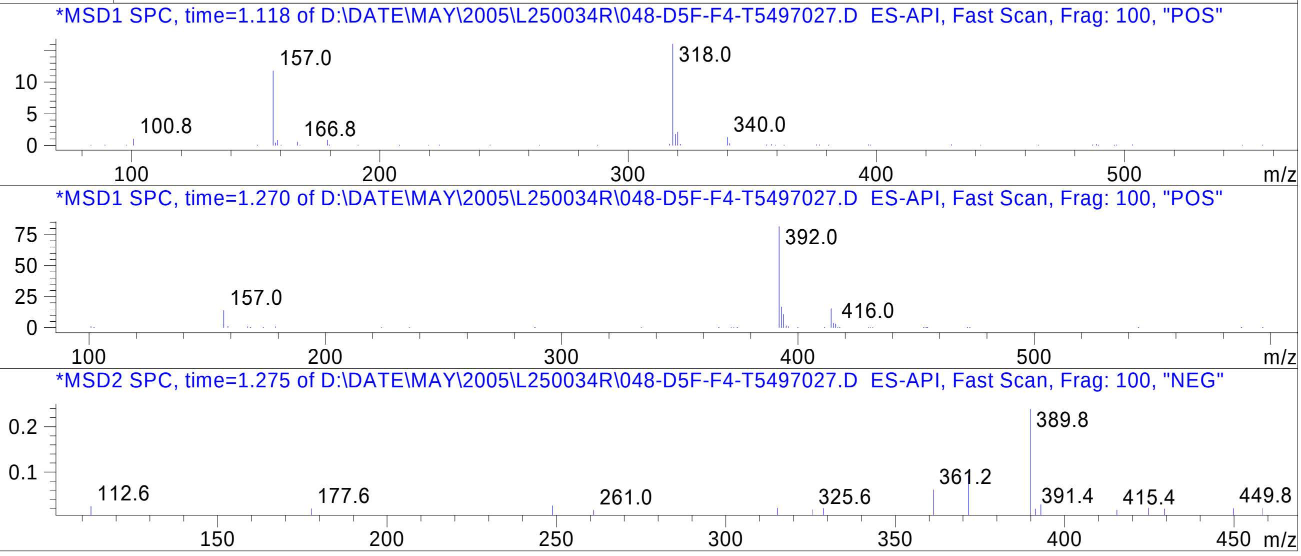

Inj.Date 5/19/2020 Y Acq. Method C:\Users\ -> ->
