# Supplementary material for: AI is a viable alternative to high throughput screening: a 318-target study
Source: Sci Rep. 2024 Apr 2;14:7526. doi: 10.1038/s41598-024-54655-z (PMC10987645; doi:10.1038/s41598-024-54655-z)
Supplement: Supplementary file 1 — Supplementary Information 1. [file 41598_2024_54655_MOESM1_ESM.zip › Nature SREP/QC_AIMS_files/Proj150.pdf]

MaxPeak: 90.18%
Ret_Time: 1.190 min

6841228

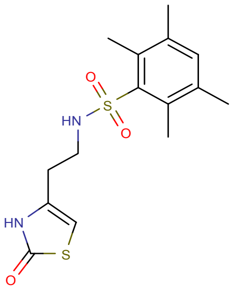

Mol Wt 340.46
Exact Mass 340.11

#	Time	Area%
1	0.994	2.78
2	1.190	90.18
3	1.346	3.38
4	1.634	3.66

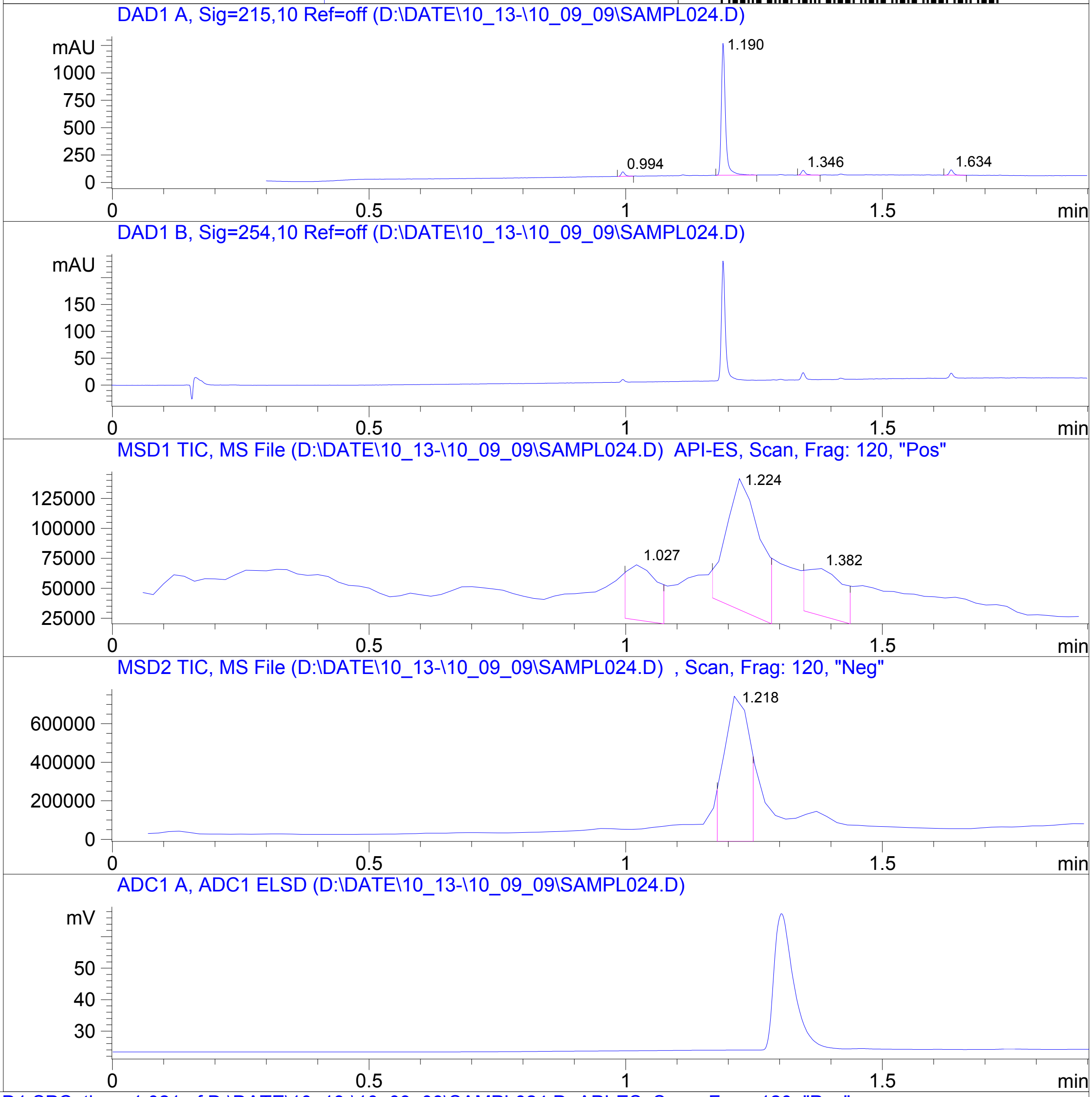

RT 1.027
RT 1.224
RT 1.382
RT 1.218

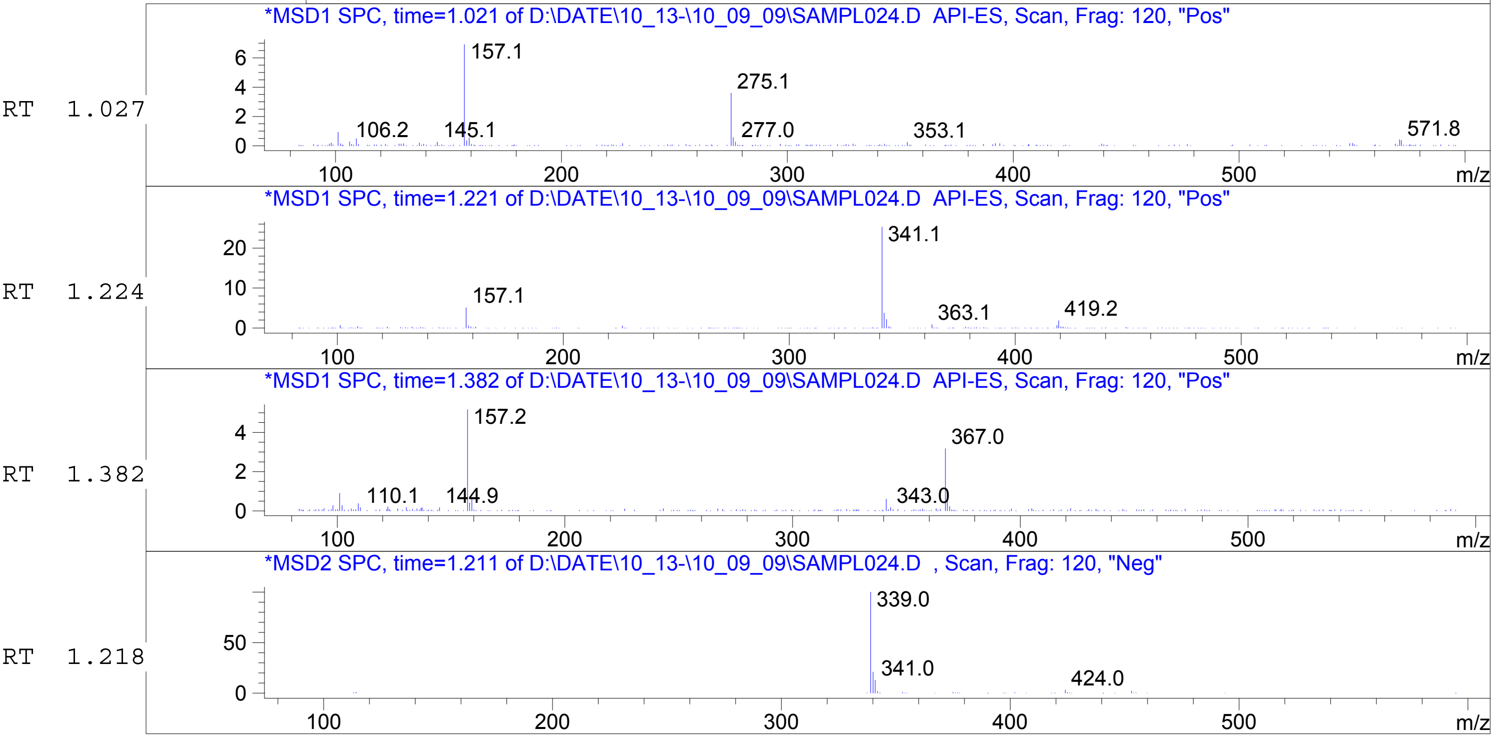

Inj.Date 10/13/2014 L P2-C-06 VL Acq. Method C:\HPCHEM\-> ->
