# Supplementary material for: AI is a viable alternative to high throughput screening: a 318-target study
Source: Sci Rep. 2024 Apr 2;14:7526. doi: 10.1038/s41598-024-54655-z (PMC10987645; doi:10.1038/s41598-024-54655-z)
Supplement: Supplementary file 1 — Supplementary Information 1. [file 41598_2024_54655_MOESM1_ESM.zip › Nature SREP/QC_AIMS_files/Proj151.pdf]

MaxPeak: 93.83%
Ret_Time: 1.390 min

6371989

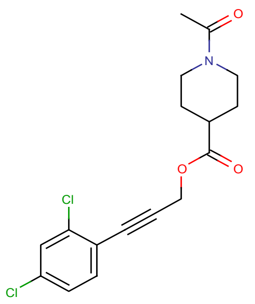

Mol Wt 354.23
Exact Mass 353.08

#	Time	Area%
1	1.205	6.17
2	1.390	93.83

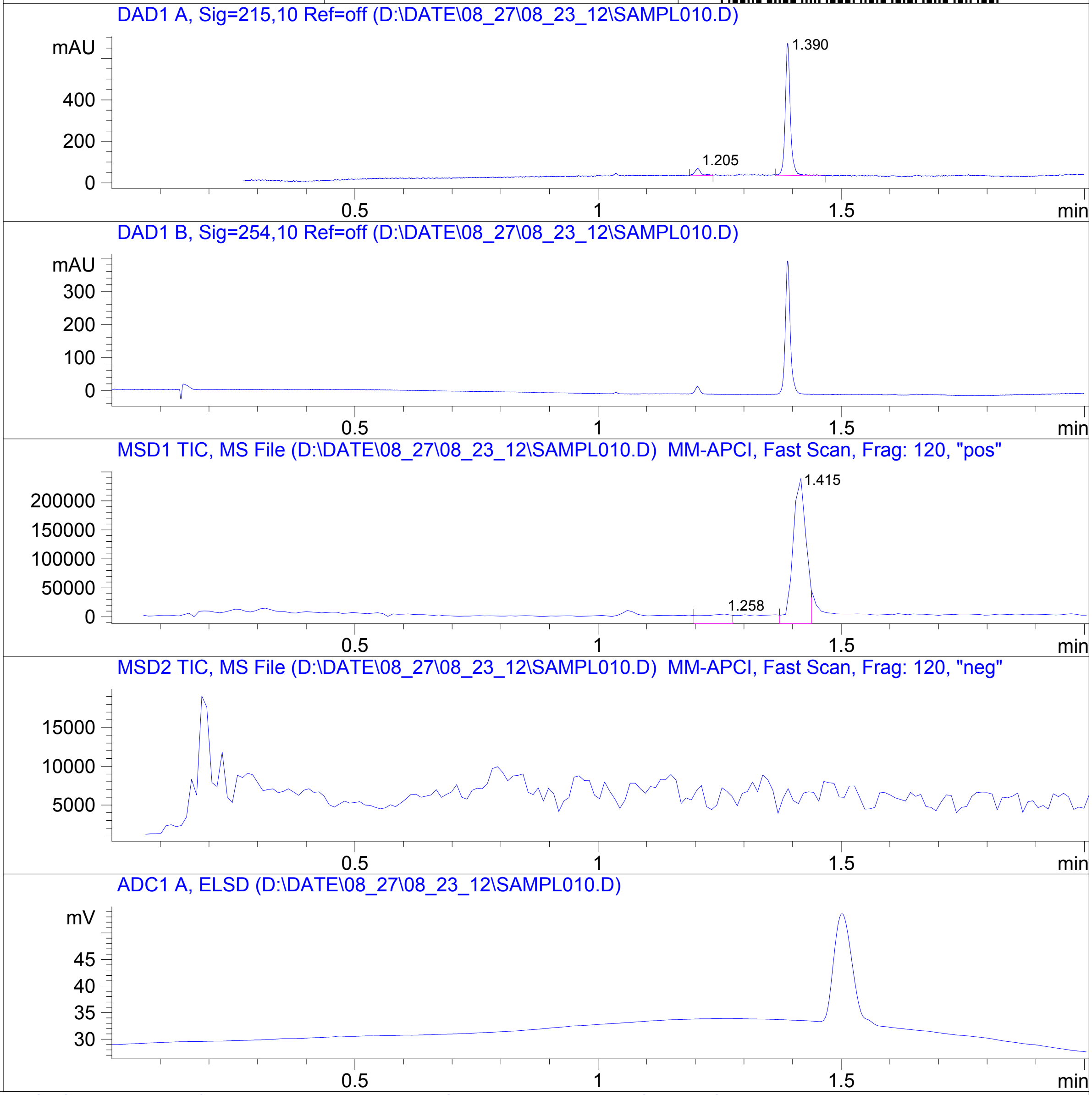

RT 1.258

RT 1.415

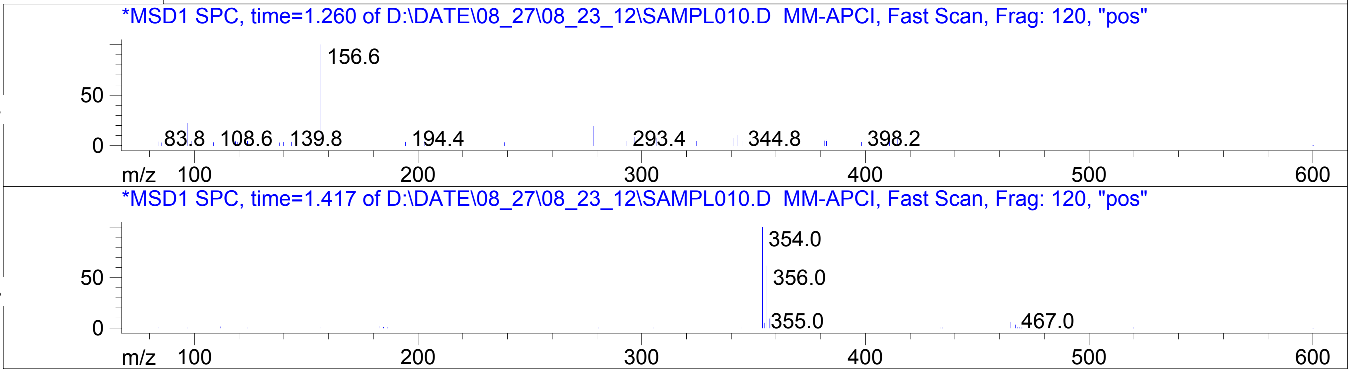

Inj.Date 8/24/2013 L P2-B-02 RR Acq. Method C:\CHEM32\-> ->
