# Supplementary material for: AI is a viable alternative to high throughput screening: a 318-target study
Source: Sci Rep. 2024 Apr 2;14:7526. doi: 10.1038/s41598-024-54655-z (PMC10987645; doi:10.1038/s41598-024-54655-z)
Supplement: Supplementary file 1 — Supplementary Information 1. [file 41598_2024_54655_MOESM1_ESM.zip › Nature SREP/QC_AIMS_files/Proj156.pdf]

T5802441

MaxPeak: 93.90%
Ret_Time: 1.396 min

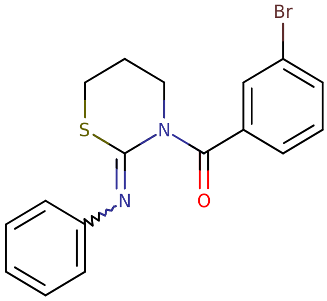

Mol Wt 375.28

Exact Mass 376.03

#	Time	Area%
1	1.282	6.10
2	1.396	93.90

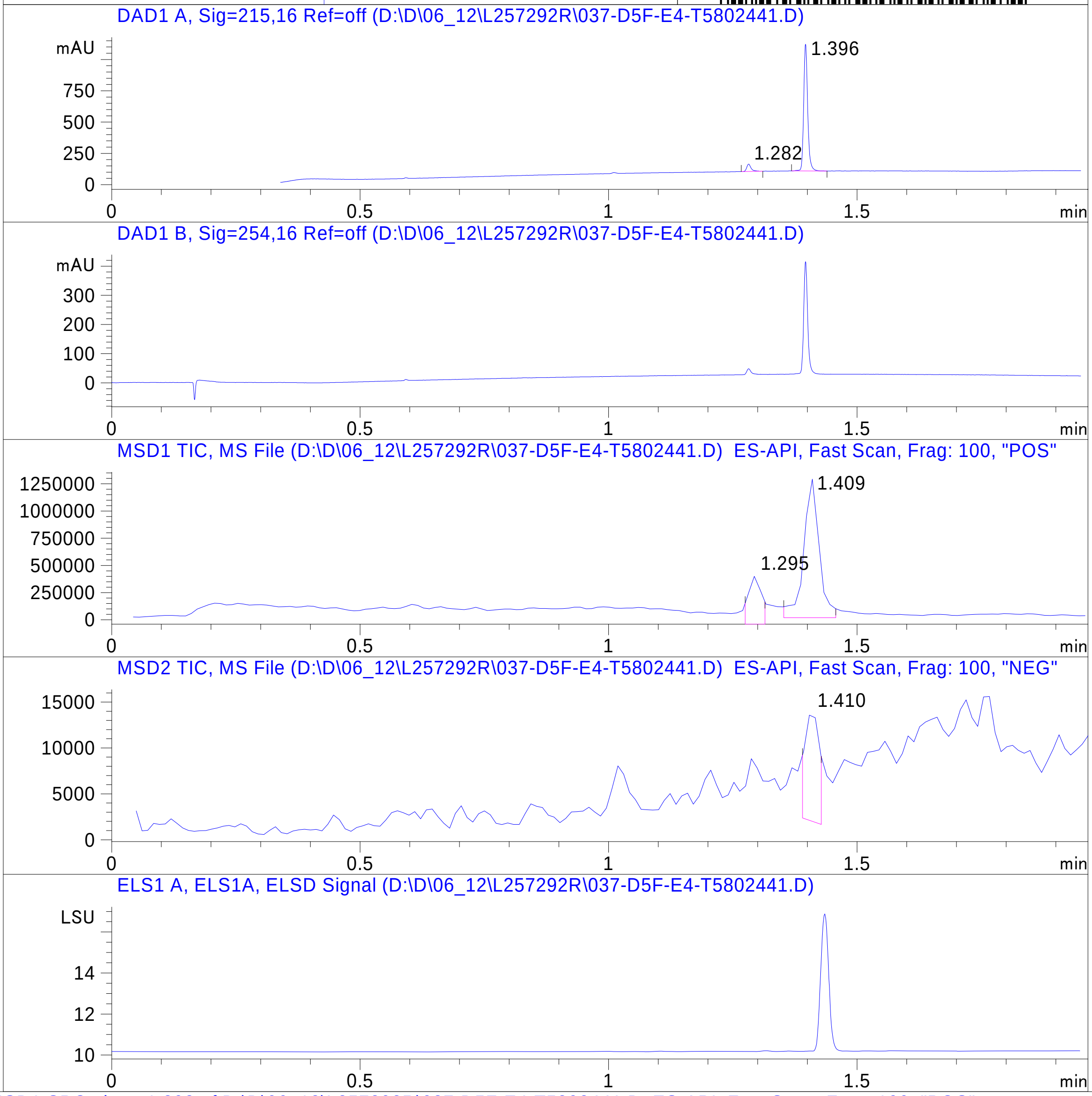

RT 1.295

RT 1.409

RT 1.410

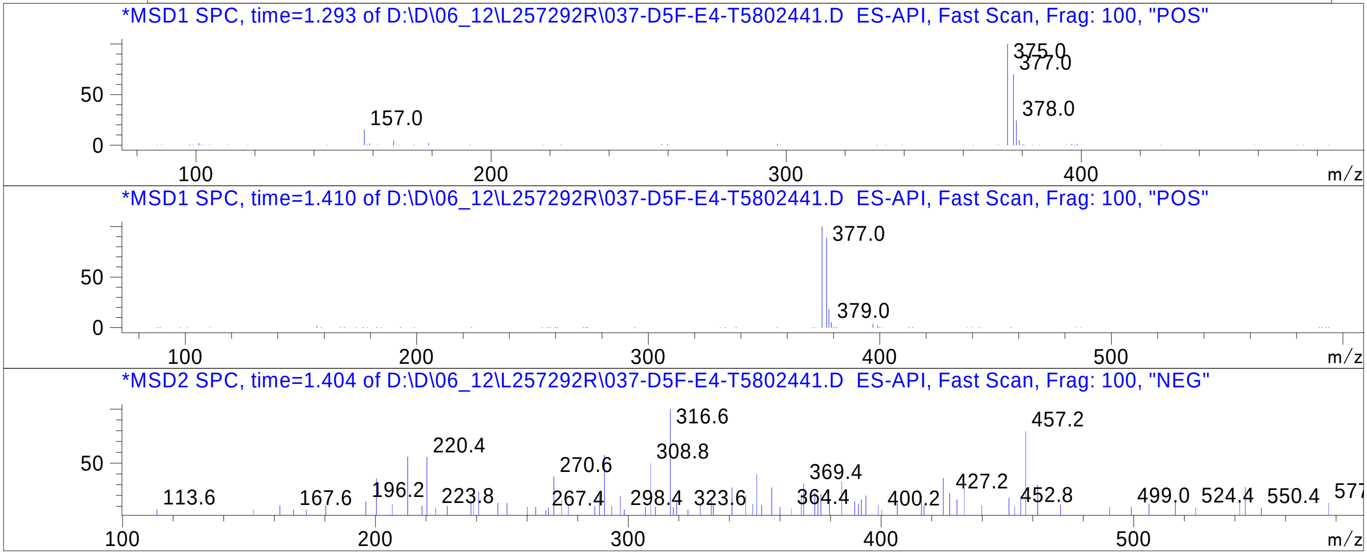

Inj.Date 6/12/2020 N Acq. Method C:\Users\ -> ->
