# Supplementary material for: AI is a viable alternative to high throughput screening: a 318-target study
Source: Sci Rep. 2024 Apr 2;14:7526. doi: 10.1038/s41598-024-54655-z (PMC10987645; doi:10.1038/s41598-024-54655-z)
Supplement: Supplementary file 1 — Supplementary Information 1. [file 41598_2024_54655_MOESM1_ESM.zip › Nature SREP/QC_AIMS_files/Proj160.pdf]

MaxPeak: 99.42%
Ret_Time: 1.417 min

T6066090

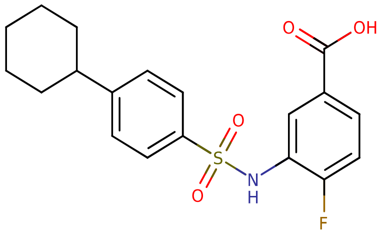

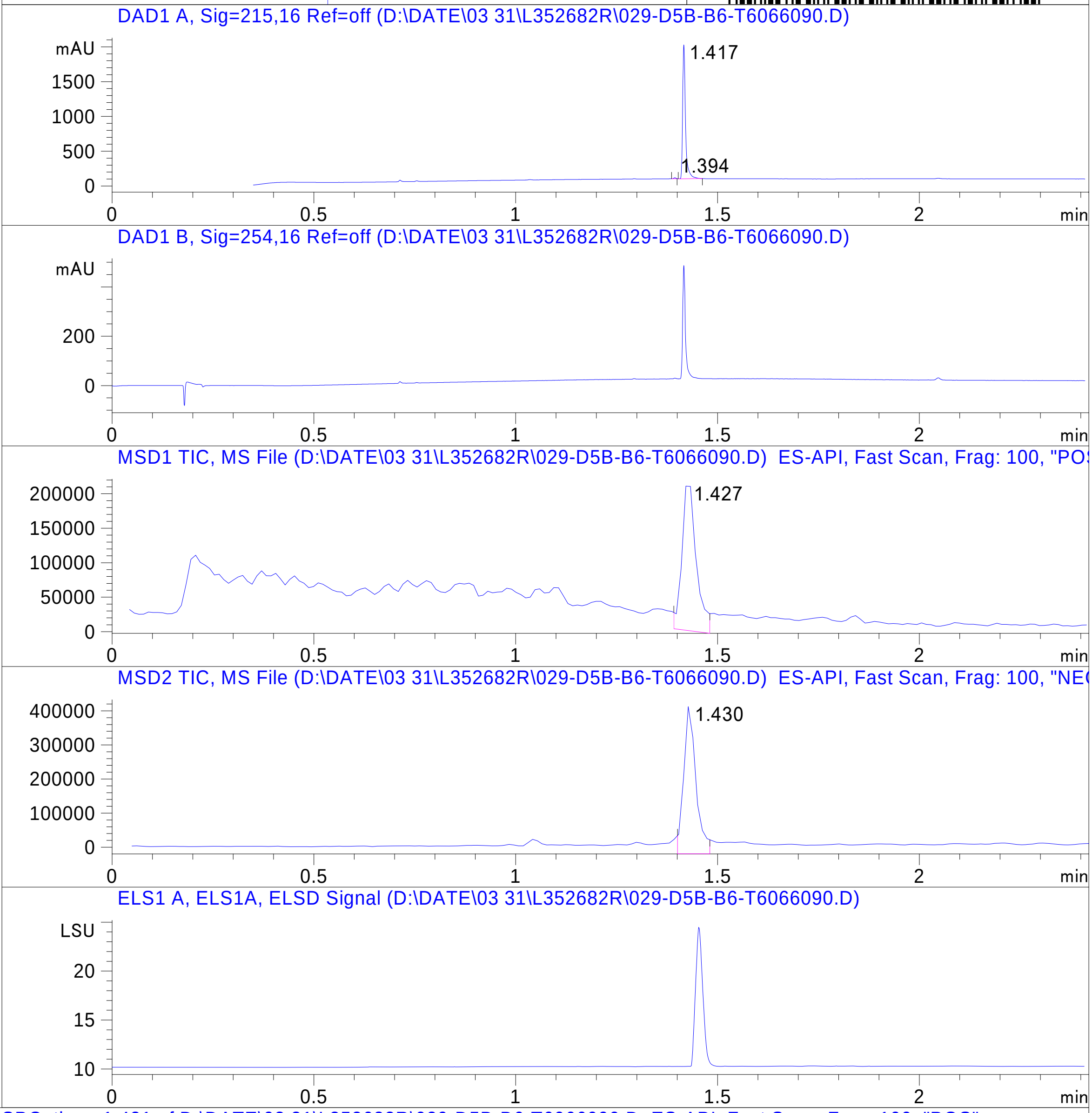

Mol Wt 377.43

Exact Mass 377.13

#	Time	Area%
1	1.394	0.58
2	1.417	99.42

RT 1.427

RT 1.430

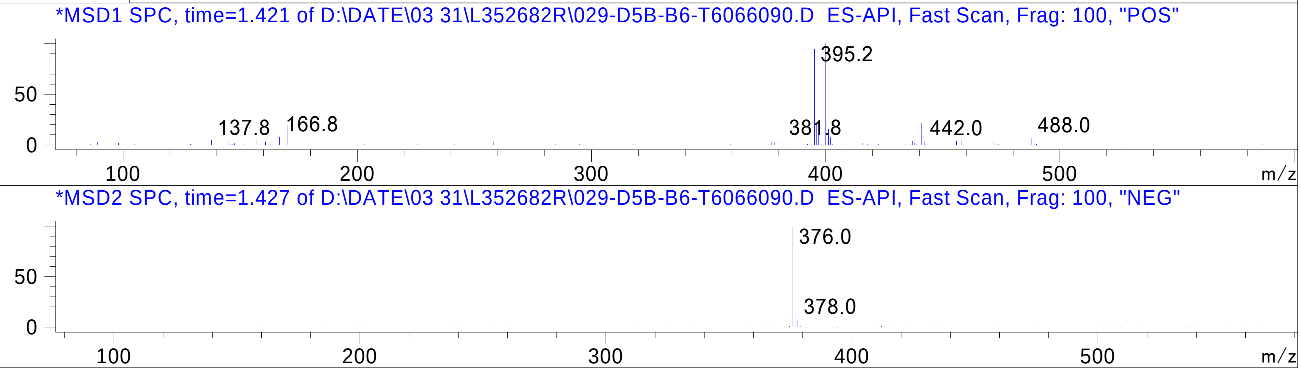

Inj.Date 3/31/2021 E Acq. Method C:\Users\ -> ->
