# Supplementary material for: AI is a viable alternative to high throughput screening: a 318-target study
Source: Sci Rep. 2024 Apr 2;14:7526. doi: 10.1038/s41598-024-54655-z (PMC10987645; doi:10.1038/s41598-024-54655-z)
Supplement: Supplementary file 1 — Supplementary Information 1. [file 41598_2024_54655_MOESM1_ESM.zip › Nature SREP/QC_AIMS_files/Proj161.pdf]

MaxPeak: 100.00%
Ret_Time: 1.415 min

T6922247

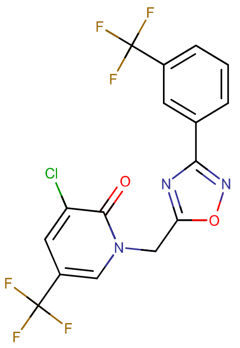

Mol Wt 423.7

Exact Mass 423.03

#	Time	Area%
1	1.415	100.00

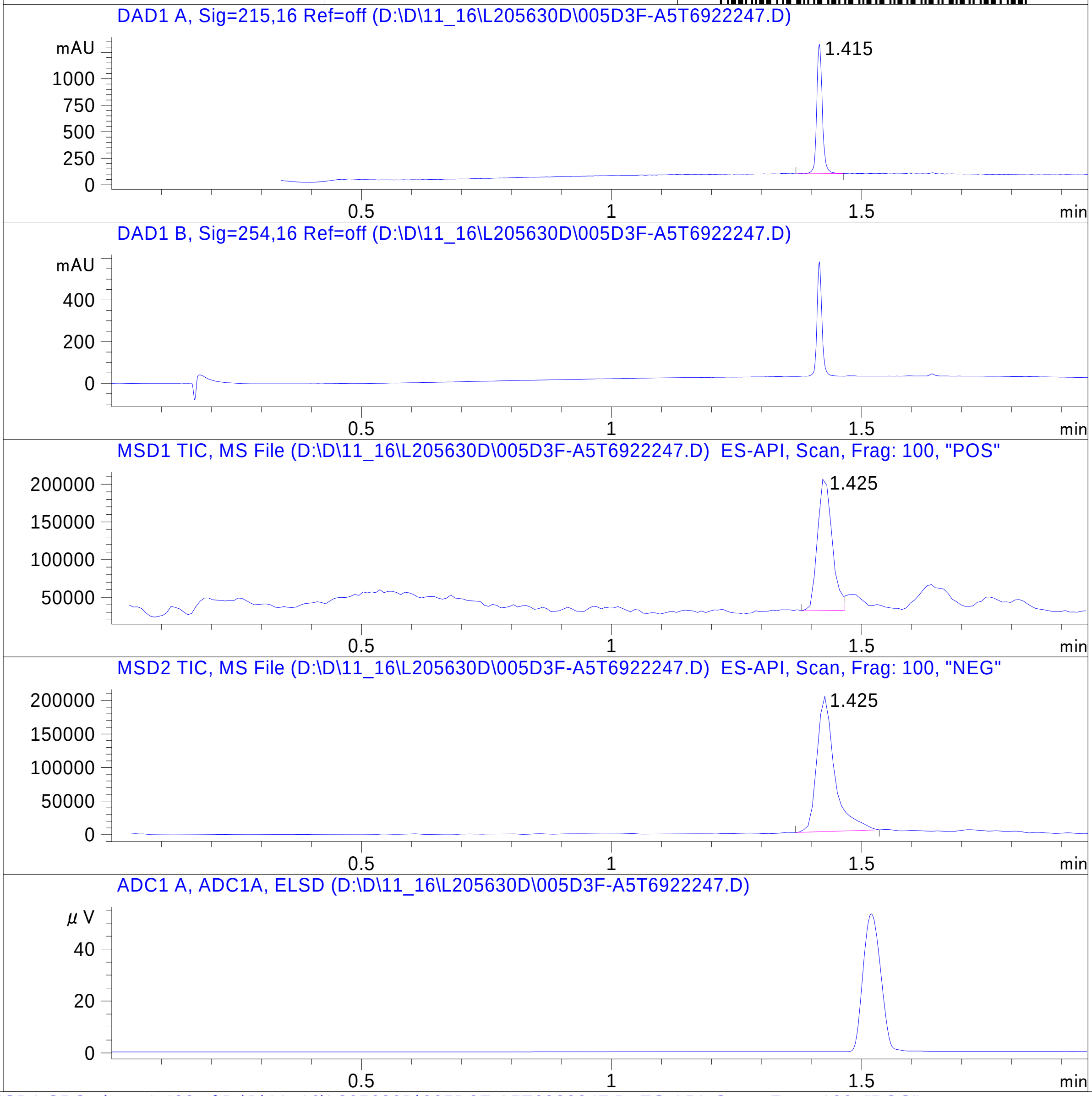

RT 1.425

RT 1.425

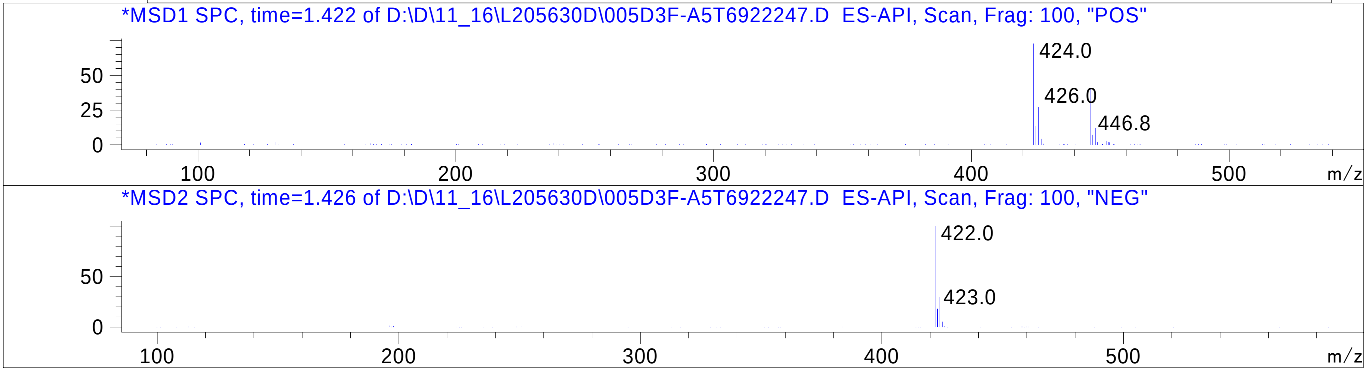

Inj.Date 11/15/2019   N   <invalid> -11-   Acq. Method C:\Chem32\-> ->
