# Supplementary material for: AI is a viable alternative to high throughput screening: a 318-target study
Source: Sci Rep. 2024 Apr 2;14:7526. doi: 10.1038/s41598-024-54655-z (PMC10987645; doi:10.1038/s41598-024-54655-z)

T6160453

MaxPeak: 97.48%
Ret_Time: 1.330 min

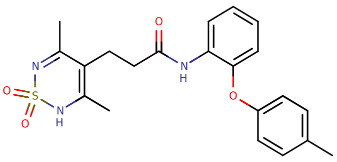

Mol Wt 413.49
Exact Mass 413.16

#	Time	Area%
1	0.674	1.85
2	0.851	0.67
3	1.330	97.48

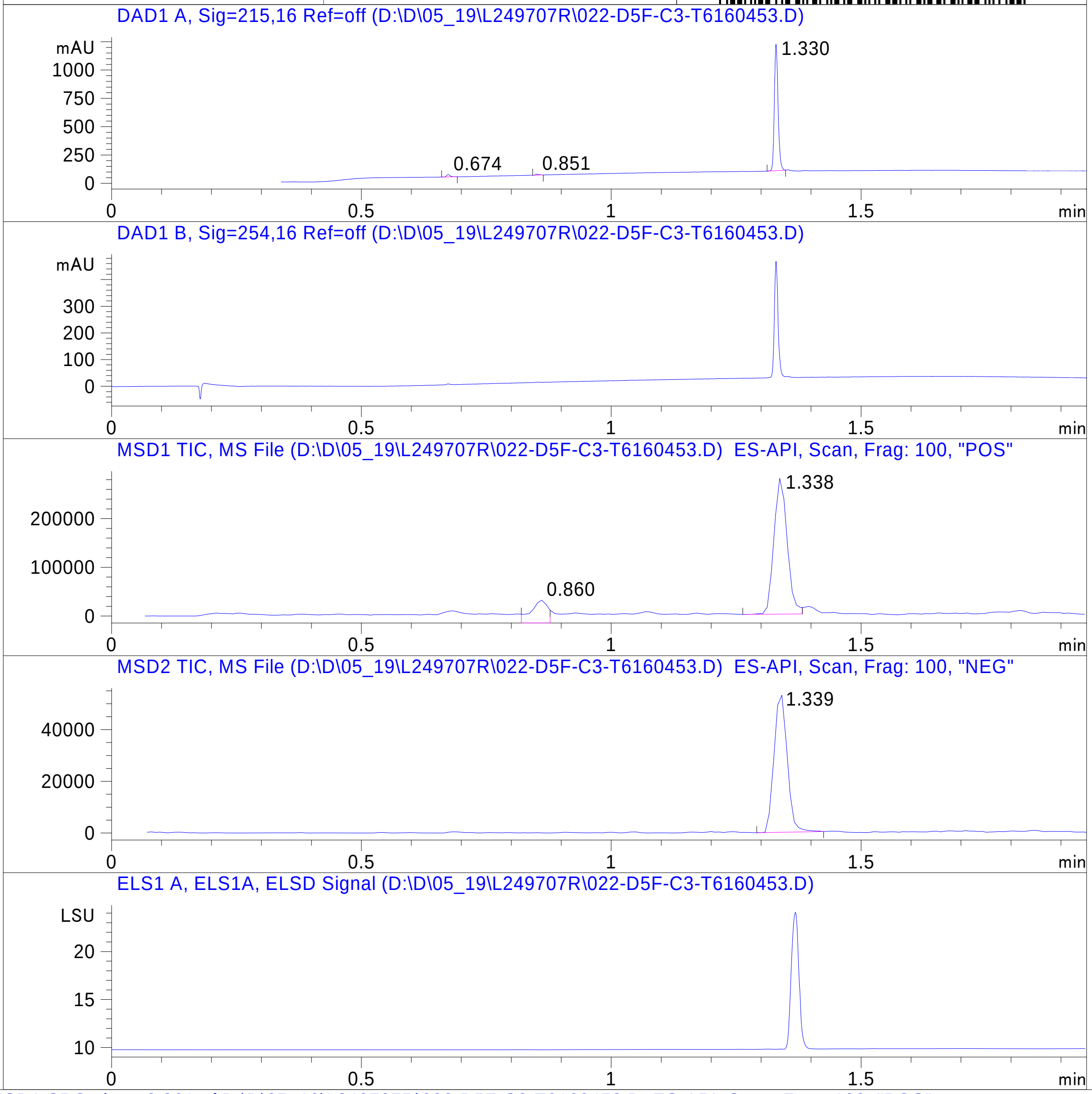

RT 0.860

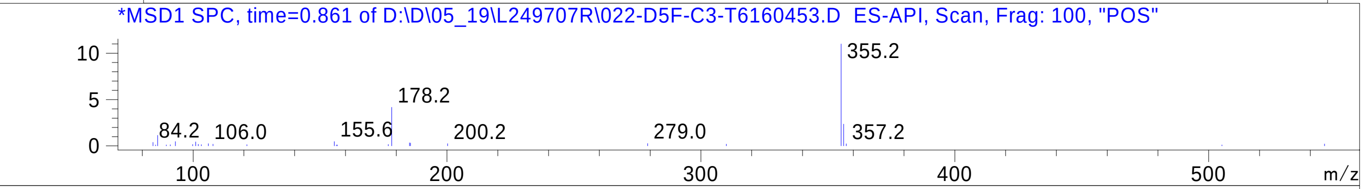

RT 1.338

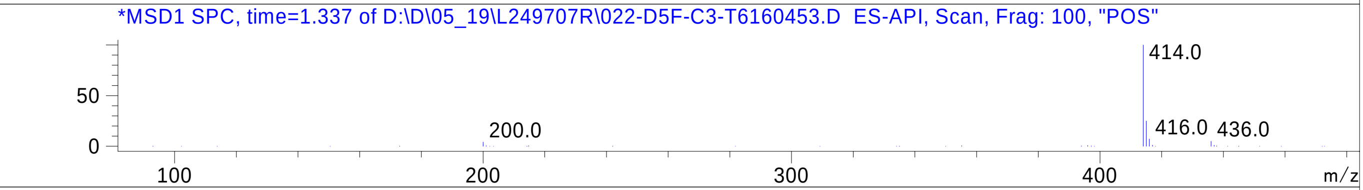

RT 1.339

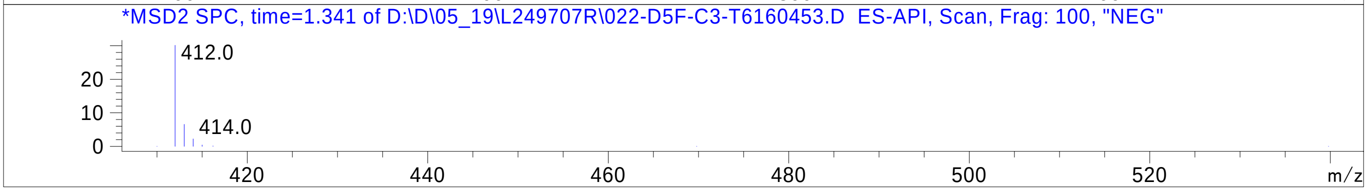

Supplement: Supplementary file 1 — Supplementary Information 1. [file 41598_2024_54655_MOESM1_ESM.zip › Nature SREP/QC_AIMS_files/Proj163.pdf]
