# Supplementary material for: AI is a viable alternative to high throughput screening: a 318-target study
Source: Sci Rep. 2024 Apr 2;14:7526. doi: 10.1038/s41598-024-54655-z (PMC10987645; doi:10.1038/s41598-024-54655-z)
Supplement: Supplementary file 1 — Supplementary Information 1. [file 41598_2024_54655_MOESM1_ESM.zip › Nature SREP/QC_AIMS_files/Proj164.pdf]

T5459815

MaxPeak: 95.50%
Ret_Time: 1.459 min

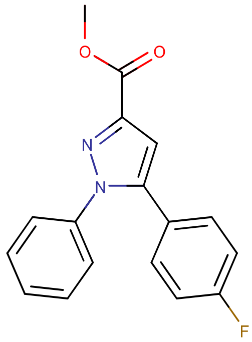

Mol Wt 296.3

Exact Mass 296.11

#	Time	Area%
1	1.459	95.50
2	1.526	4.50

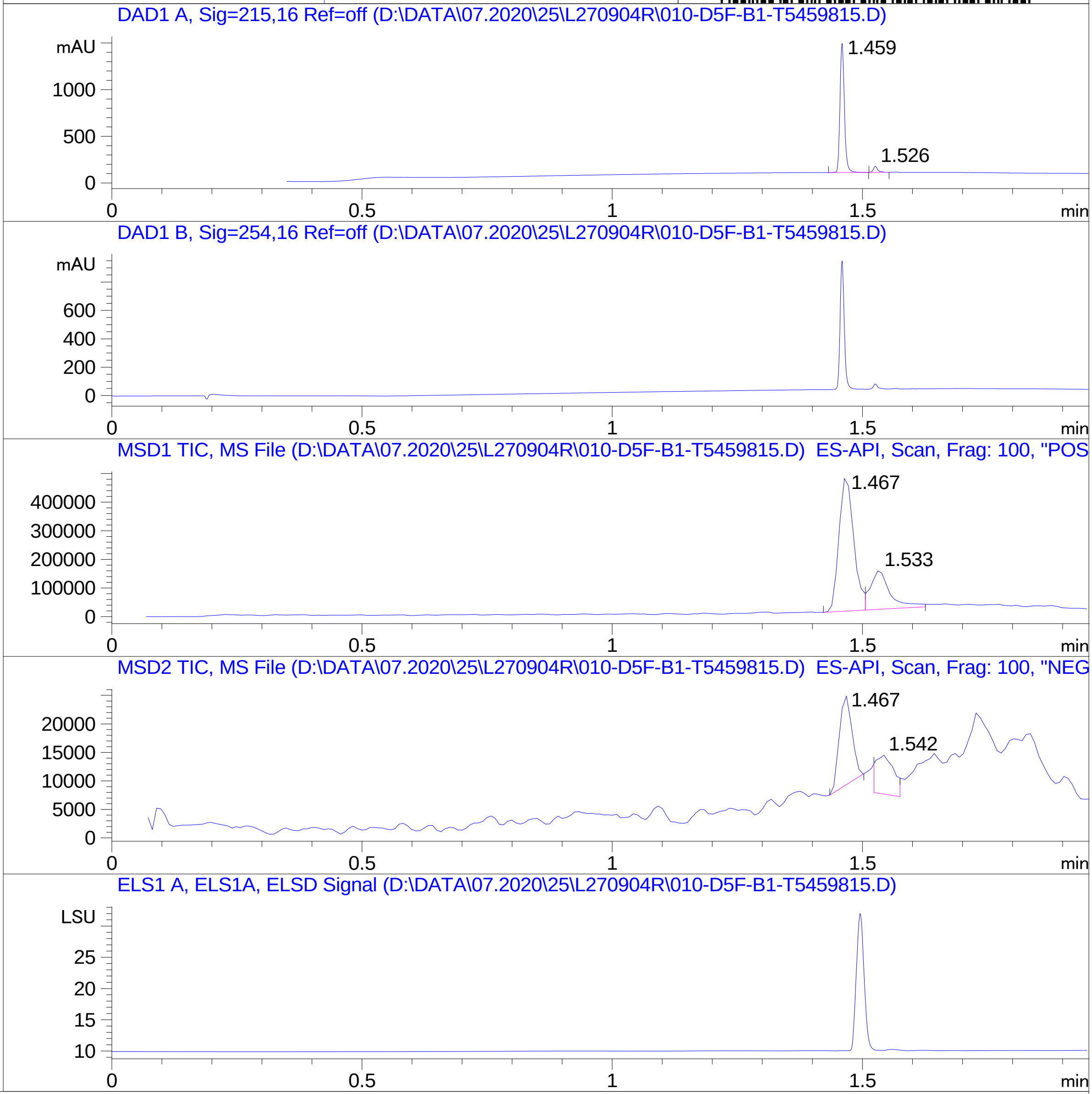

RT 1.467

RT 1.533

RT 1.467

RT 1.542

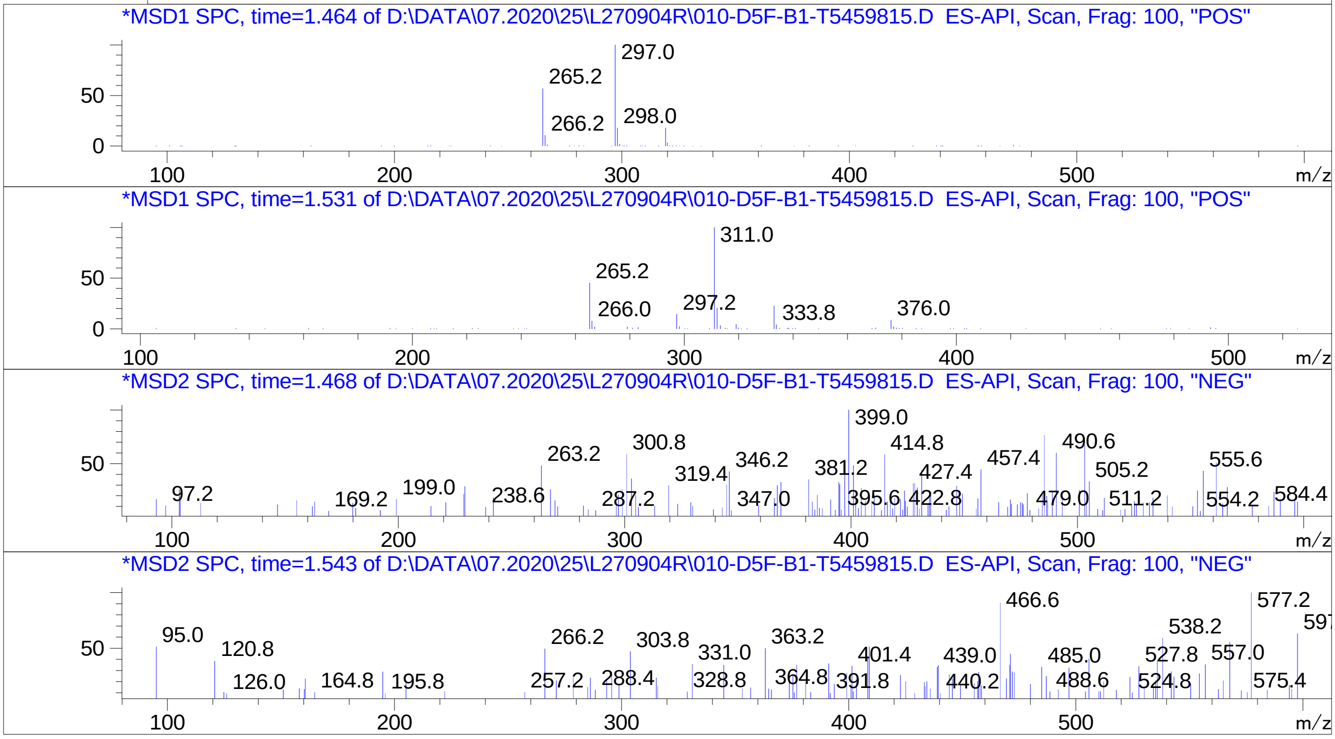

Inj.Date 7/24/2020 CH <invalid> -17- Acq. Method C:\Chem32\-> ->
