# Supplementary material for: AI is a viable alternative to high throughput screening: a 318-target study
Source: Sci Rep. 2024 Apr 2;14:7526. doi: 10.1038/s41598-024-54655-z (PMC10987645; doi:10.1038/s41598-024-54655-z)
Supplement: Supplementary file 1 — Supplementary Information 1. [file 41598_2024_54655_MOESM1_ESM.zip › Nature SREP/QC_AIMS_files/Proj180.pdf]

MaxPeak: 100.00%
Ret_Time: 1.562 min

T5280898

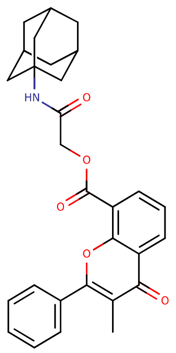

Mol Wt 471.54

Exact Mass 471.24

#	Time	Area%
1	1.562	100.00

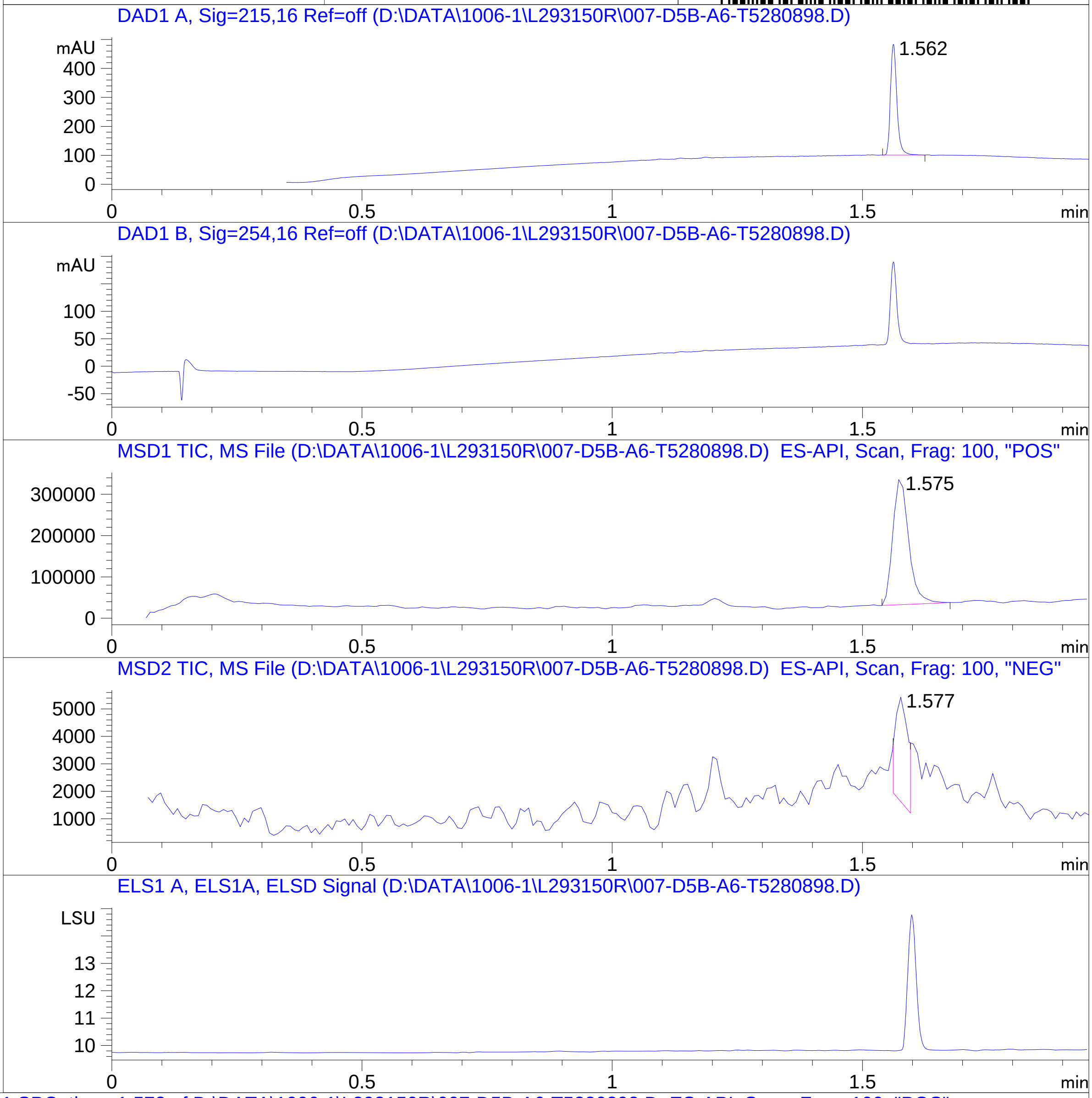

RT 1.575

RT 1.577

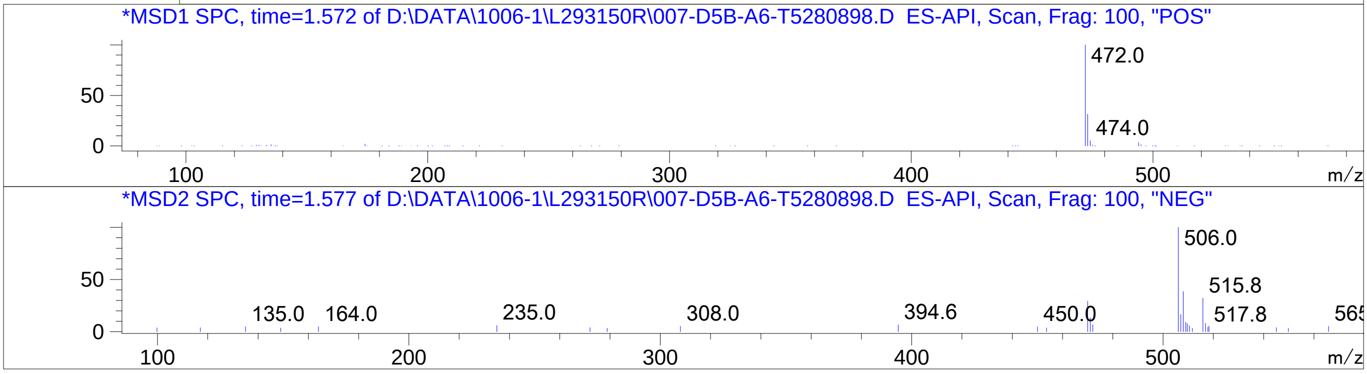

Inj.Date 10/6/2020 M 13 Acq. Method C:\Chem32\-> ->
