# Supplementary material for: AI is a viable alternative to high throughput screening: a 318-target study
Source: Sci Rep. 2024 Apr 2;14:7526. doi: 10.1038/s41598-024-54655-z (PMC10987645; doi:10.1038/s41598-024-54655-z)

Analysis number 337104
Compound Code HTS13968
Book Reference 600/38/2
Project Code QC-SCR
Chemist Name DaveC

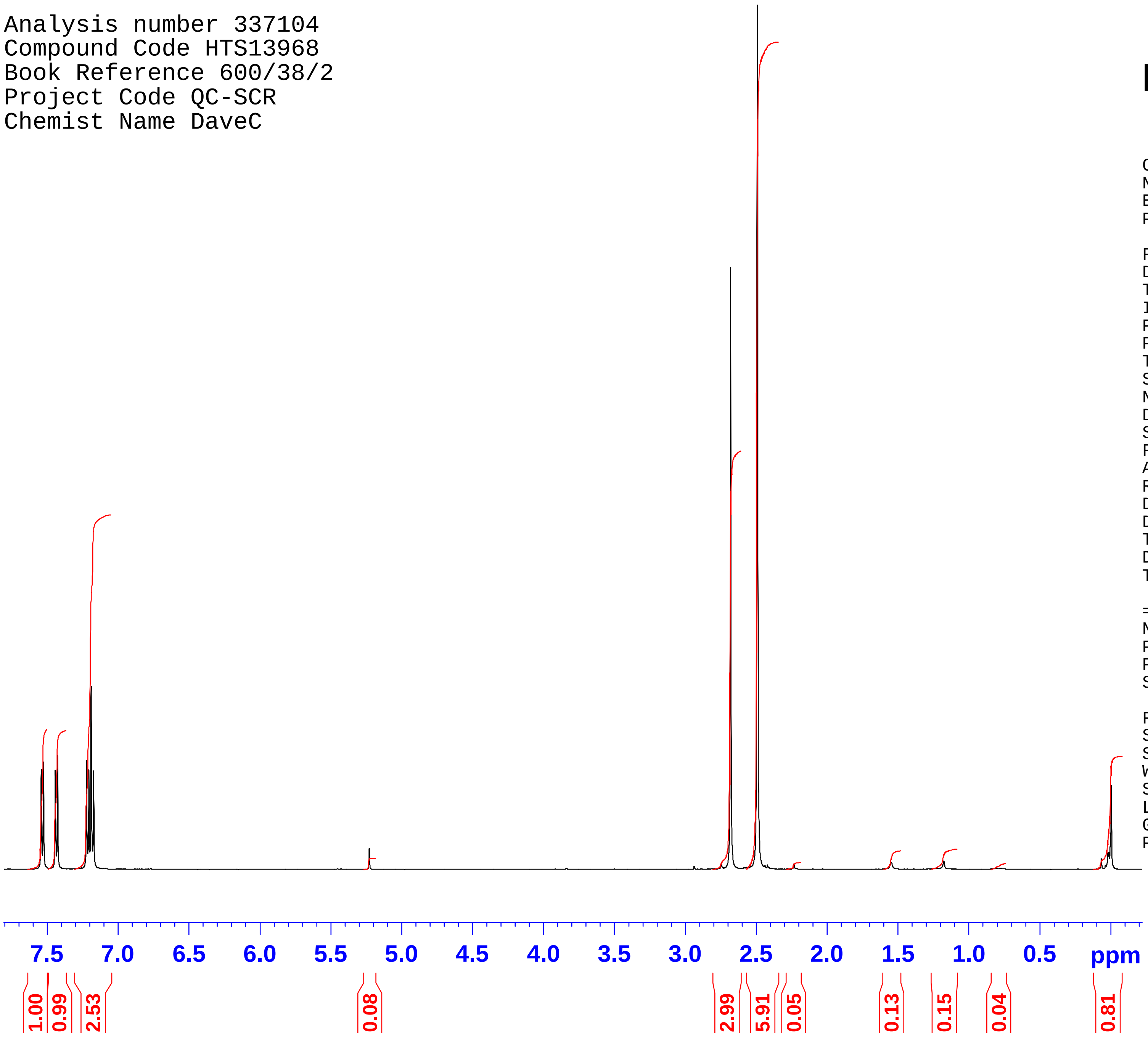

Current Data Parameters

NAME	ADRS
EXPNO	18430
PROCNO	1

F2 - Acquisition Parameters

Date_	20110318
Time	12.39
INSTRUM	dpx250
PROBHD	5 mm Dual 13C/
PULPROG	zg30
TD	65536
SOLVENT	CDCl3
NS	32
DS	2
SWH	5175.983 Hz
FIDRES	0.078979 Hz
AQ	6.3308277 sec
RG	912.3
DW	96.600 usec
DE	6.00 usec
TE	293.2 K
D1	1.00000000 sec
TD0	1

======== CHANNEL f1 ========

NUC1	1H
P1	9.00 usec
PL1	0.00 dB
SFO1	250.1315447 MHz

F2 - Processing parameters

SI	32768
SF	250.1300234 MHz
WDW	EM
SSB	0
LB	0.30 Hz
GB	0
PC	1.00

1 Signature Appended
MIDAS Raw Prints: 296029

1_HTS13968
2_600/38/2
3_DaveC
4_QC-SCR

===

Injection Date : 18/03/2011 12:45:50 PM Seq. Line : 15
Sample Name : 337104 Location : P1-A-05
Acq. Operator : Inj : 1
Inj Volume : 5 µl
Sequence File : C:\HPCHEM\HPLC_2\SEQUENCE\18MAR11.S
Method : C:\HPCHEM\HPLC_2\METHODS\HIGH_5.M
Last changed : 09/02/2011 14:19:29 PM
pH10 mobile phase (water +0.05% NH3, Acetonitrile +0.05% NH3)
5cm column
Flow-rate 1ml/min 5ul injection, 40C column temp, 215nm

===

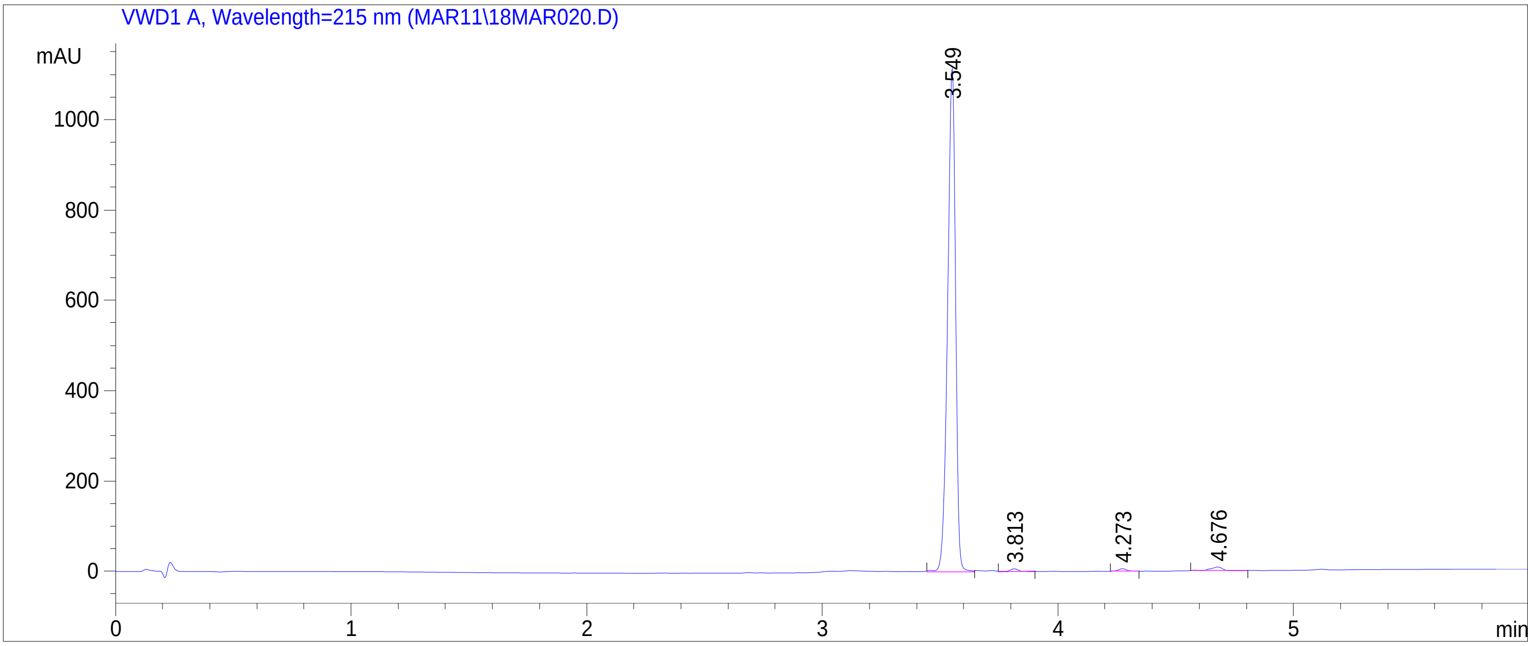

===

Area Percent Report

===

Sorted By : Signal
Multiplier : 1.0000
Dilution : 1.0000
Use Multiplier & Dilution Factor with ISTDs

Signal 1: VWD1 A, Wavelength=215 nm

Peak #	RetTime [min]	Type	Width [min]	Area mAU *s	Height [mAU]	Area %
1	3.549	BB	0.0377	2615.38208	1109.72876	97.6330
2	3.813	BB	0.0451	19.45452	6.50168	0.7262
3	4.273	PP	0.0382	13.49616	5.62426	0.5038
4	4.676	BB	0.0552	30.45693	8.16631	1.1370

Totals : 2678.78969 1130.02101

Results obtained with enhanced integrator!

===

*** End of Report ***

1 Signature Appended
MIDAS Raw Prints: 296028

Analysis number 337104
Compound Code HTS13968
Book Reference 600/38/2
Project Code QC-SCR
Chemist Name DaveC

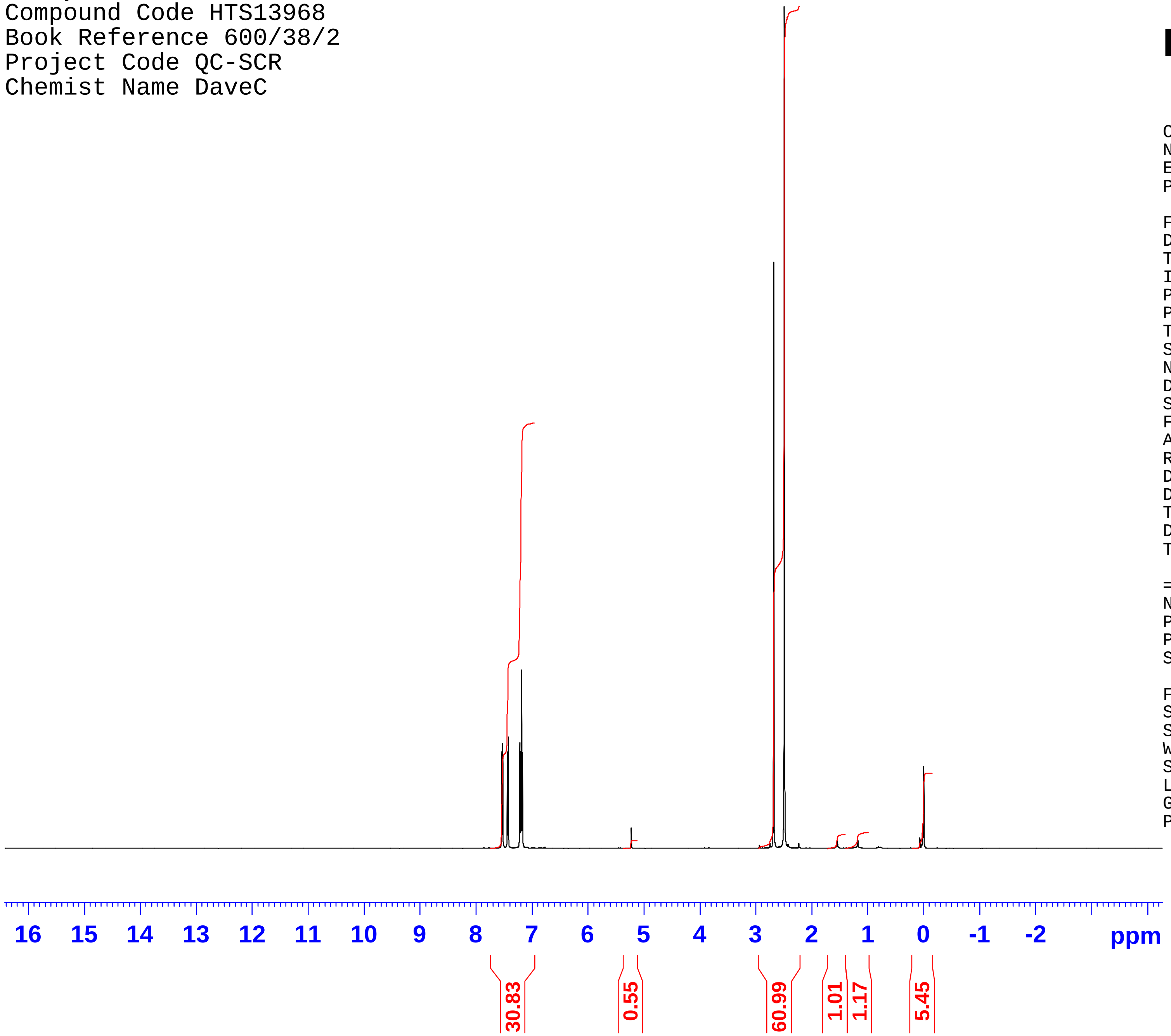

BRUKER

Current Data Parameters
NAME ADRS
EXPNO 18430
PROCNO 1

F2 - Acquisition Parameters
Date_ 20110318
Time 12.39
INSTRUM dpx250
PROBHD 5 mm Dual 13C/
PULPROG zg30
TD 65536
SOLVENT CDCl3
NS 32
DS 2
SWH 5175.983 Hz
FIDRES 0.078979 Hz
AQ 6.3308277 sec
RG 912.3
DW 96.600 usec
DE 6.00 usec
TE 293.2 K
D1 1.00000000 sec
TD0 1

======== CHANNEL f1 ========
NUC1 1H
P1 9.00 usec
PL1 0.00 dB
SFO1 250.1315447 MHz

F2 - Processing parameters
SI 32768
SF 250.1300234 MHz
WDW EM
SSB 0
LB 0.30 Hz
GB 0
PC 1.00

1 Signature Appended
MIDAS Raw Prints: 296025

Openlynx Report - davec

Sample: 5 | Vial:1,2:E,2 | ID:600/38/2
File:337104 | Date:18-Mar-2011 | Time:09:19:17
Description:1_HTS13968; 2_ | Submitter:davec

Printed: Fri Mar 18 09:32:57 2011

Sample Report:

Sample 5 Vial 1,2:E,2 ID 600/38/2 File 337104 Date 18-Mar-2011 Time 09:19:17 Description 1_HTS13968; 2_

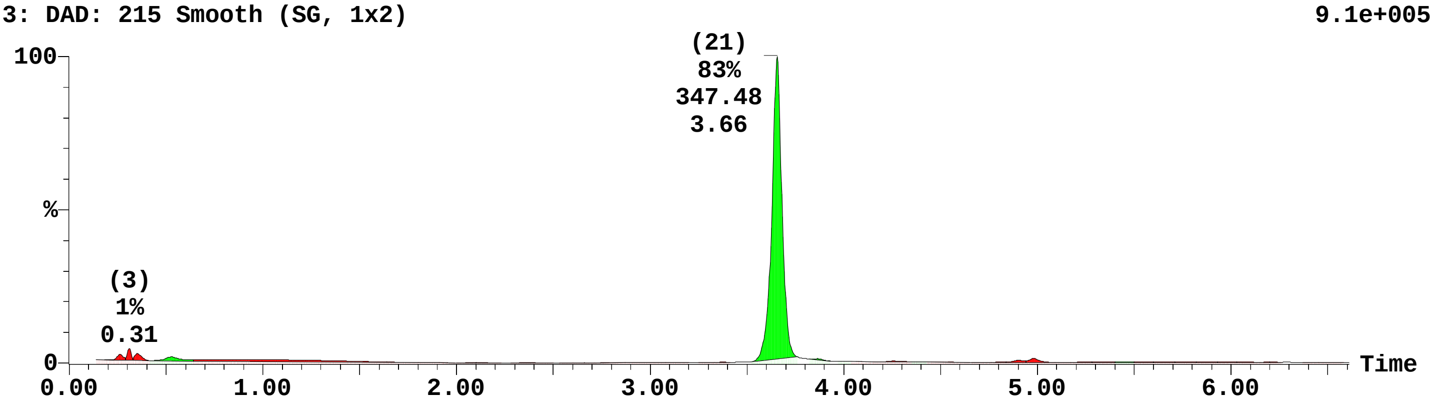

Peak Number	Compound	Time	AreaAbs	Area %Total	Width	Height	Mass Found
1		0.21	5e+001	0.07	0	1e+003	
2		0.26	6e+002	0.98	0	2e+004	
3		0.31	8e+002	1.29	0	4e+004	
4		0.35	9e+002	1.32	0	2e+004	
5	Found	0.53	1e+003	1.83	0	1e+004	347.48
6		0.88	4e+003	6.39	1	5e+003	
9		1.85	2e+001	0.03	0	3e+002	
10		1.91	6e+000	0.01	0	4e+001	
11		2.08	4e+001	0.05	0	6e+002	
12		2.11	4e+001	0.06	0	6e+002	
13		2.37	1e+002	0.18	0	1e+003	
14		2.60	2e+000	0.00	0		
15		2.66	3e+001	0.04	0	5e+002	
16		2.79	9e+001	0.14	0	1e+003	
17		3.02	8e+001	0.12	0	6e+002	
18		3.11	2e+001	0.04	0	4e+002	
19		3.29	9e+000	0.01	0	2e+002	
20		3.37	8e+000	0.01	0	2e+002	
21	Found	3.66	5e+004	83.23	0	9e+005	347.48
22	Found	3.86	1e+002	0.23	0	3e+003	347.48
23	Found	3.97	4e+000	0.01	0		347.48
24		4.08	2e+001	0.03	0	4e+002	
25		4.26	3e+002	0.40	0	3e+003	
26	Found	4.37	6e+001	0.10	0	1e+003	347.48
27		4.52	2e+002	0.24	0	2e+003	
28		4.71	3e+001	0.05	0	6e+002	
29		4.82	1e+002	0.15	0	2e+003	
30		4.90	4e+002	0.61	0	6e+003	
31		4.98	7e+002	1.14	0	1e+004	
32		5.13	2e+001	0.02	0	3e+002	
33		5.24	8e+001	0.12	0	1e+003	
34		5.34	1e+002	0.15	0	1e+003	
35	Found	5.45	8e+001	0.13	0	1e+003	347.48
36		5.55	9e+001	0.13	0	1e+003	
37		5.66	1e+002	0.21	0	2e+003	
38		5.76	1e+002	0.20	0	1e+003	
39		5.87	1e+002	0.16	0	2e+003	
40		5.96	3e+001	0.05	0	4e+002	
41		6.07	2e+001	0.03	0	3e+002	
42		6.20	5e+000	0.01	0	2e+002	
43		6.21	6e+000	0.01	0	2e+002	
44		6.41	6e+000	0.01	0	1e+002	
44		6.45	9e+000	0.01	0	1e+001	

1 Signature Appended
MIDAS Raw Prints: 295971

Sample: 5 Vial:1,2:E,2 ID:600/38/2
File:337104 Date:18-Mar-2011 Time:09:19:17
Description:1_HTS13968; 2_ Submitter:davec

Printed: Fri Mar 18 09:32:57 2011

Sample Report (continued):

Peak ID	Compound	Time	Mass Found
1		0.21	

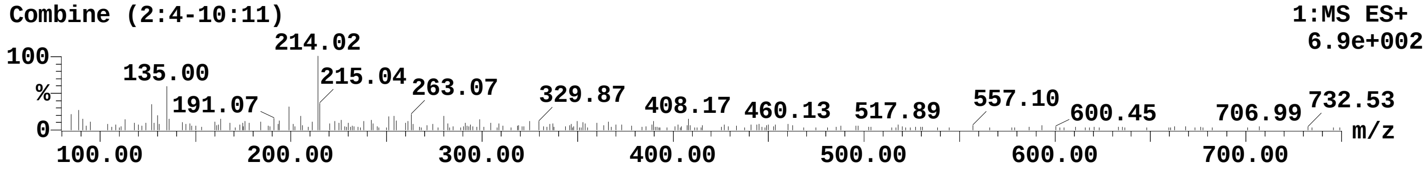

Peak ID	Compound	Time	Mass Found
2		0.26	

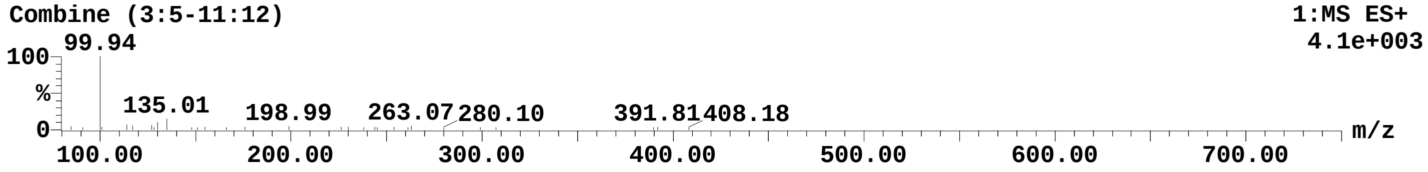

Peak ID	Compound	Time	Mass Found
3		0.31	

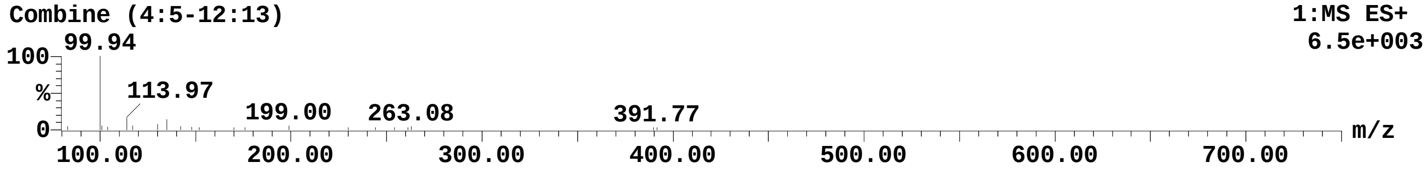

Peak ID	Compound	Time	Mass Found
4		0.35	

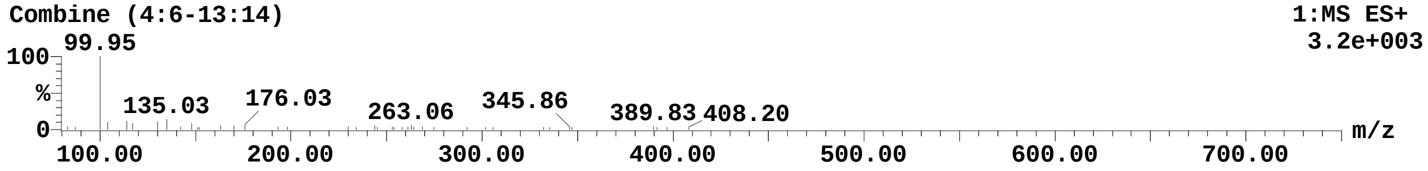

Peak ID	Compound	Time	Mass Found
5		0.53	

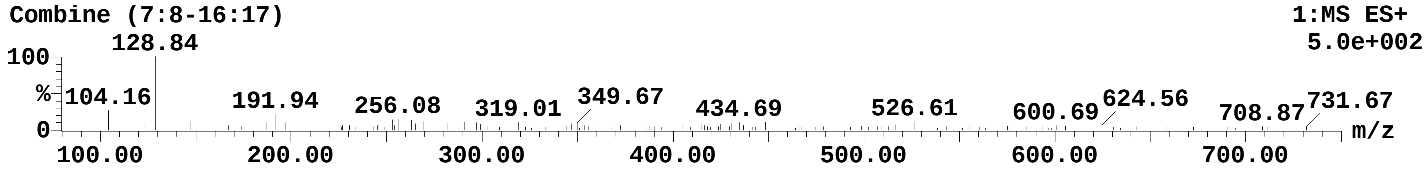

Peak ID	Compound	Time	Mass Found
6		0.88	

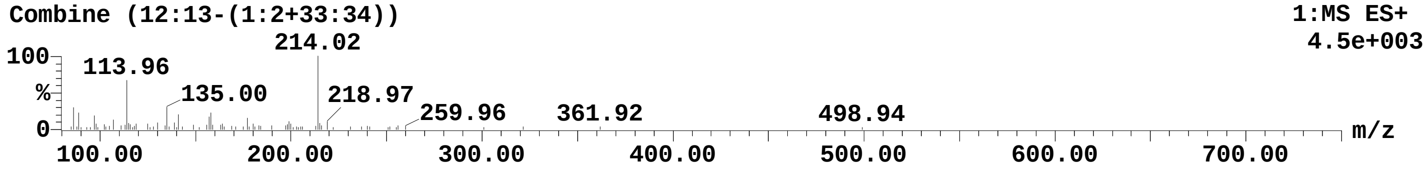

Sample: 5 | Vial:1,2:E,2 | ID:600/38/2
File:337104 | Date:18-Mar-2011 | Time:09:19:17
Description:1_HTS13968; 2_ | Submitter:davec

Printed: Fri Mar 18 09:32:57 2011

Sample Report (continued):

Peak ID	Compound	Time	Mass Found
7			

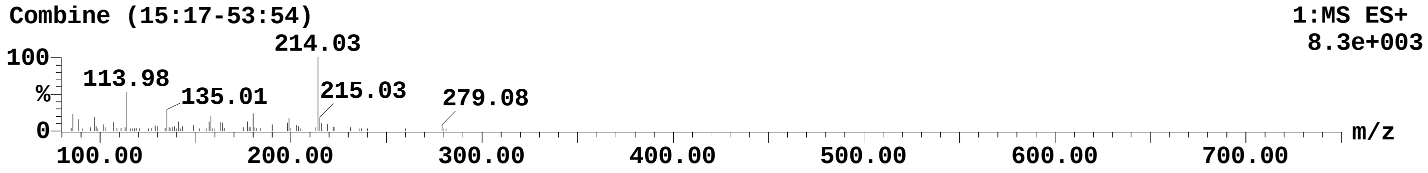

Peak ID	Compound	Time	Mass Found
9		1.85	

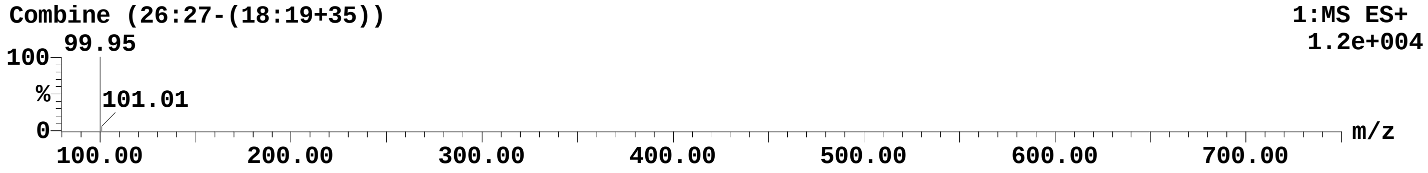

Peak ID	Compound	Time	Mass Found
10		1.91	

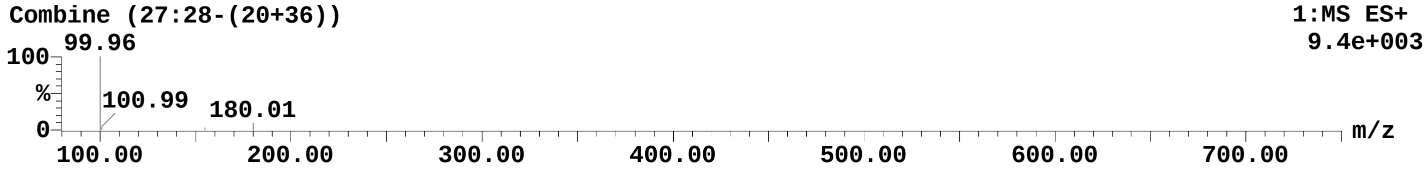

Peak ID	Compound	Time	Mass Found
11		2.08	

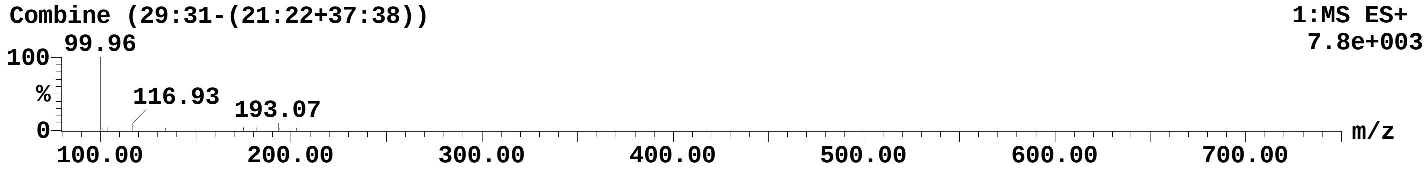

Peak ID	Compound	Time	Mass Found
12		2.11	

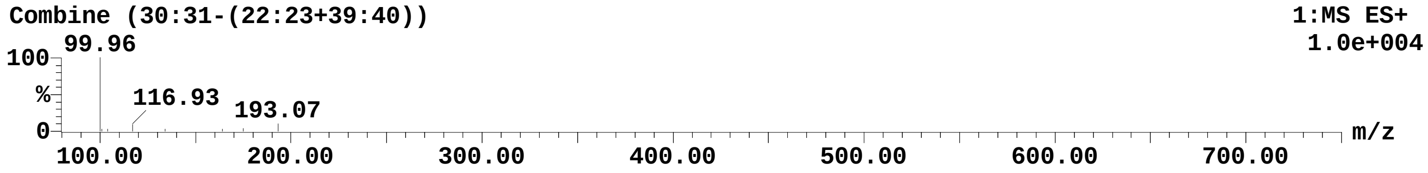

Peak ID	Compound	Time	Mass Found
13		2.37	

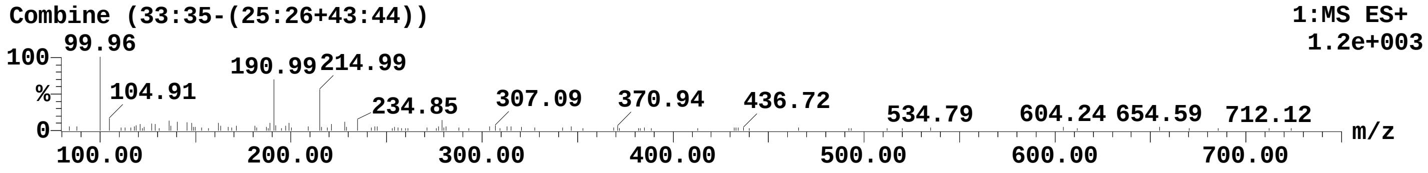

Sample: 5 | Vial:1,2:E,2 | ID:600/38/2
File:337104 | Date:18-Mar-2011 | Time:09:19:17
Description:1_HTS13968; 2_ | Submitter:davec

Printed: Fri Mar 18 09:32:57 2011

Sample Report (continued):

Peak ID	Compound	Time	Mass Found
14		2.60	

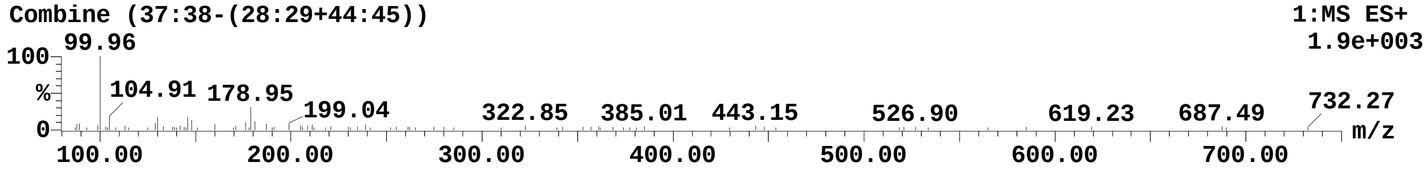

Peak ID	Compound	Time	Mass Found
15		2.66	

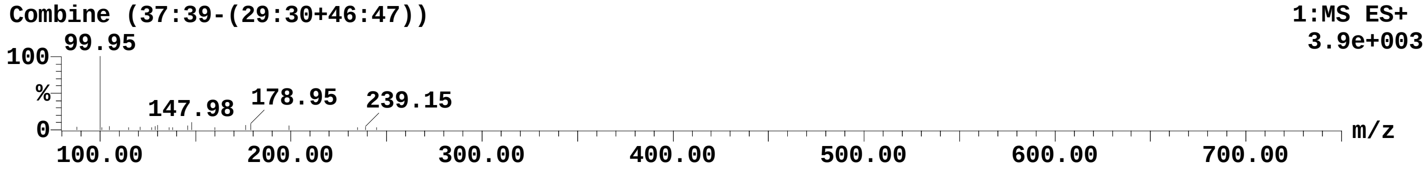

Peak ID	Compound	Time	Mass Found
16		2.79	

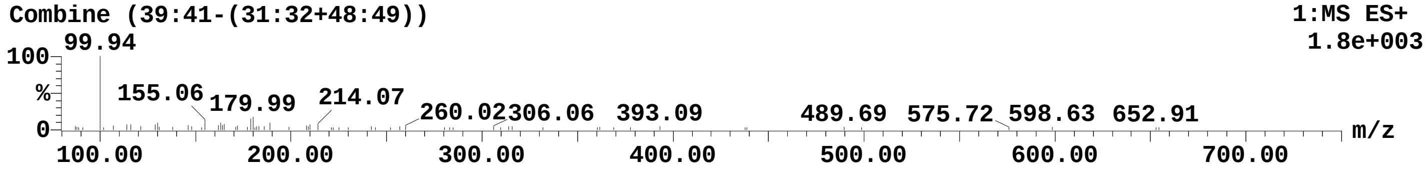

Peak ID	Compound	Time	Mass Found
17		3.02	

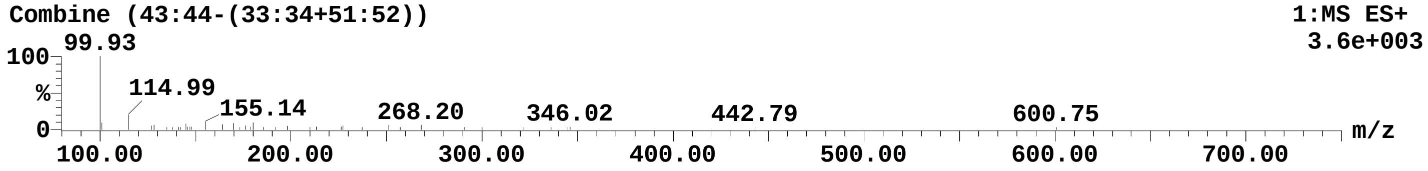

Peak ID	Compound	Time	Mass Found
18		3.11	

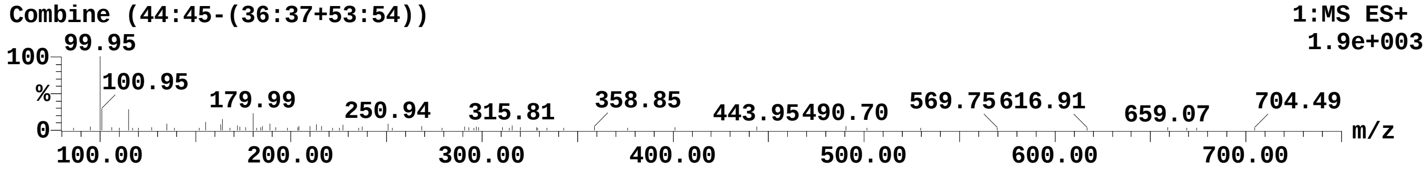

Peak ID	Compound	Time	Mass Found
19		3.29	

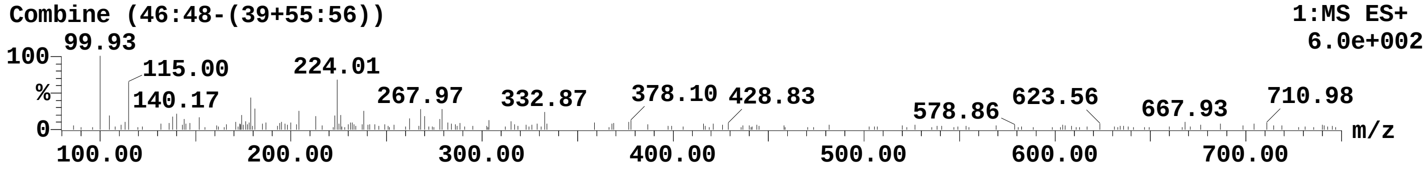

Sample Report (continued):

Peak ID	Compound	Time	Mass Found
20		3.37	

Combine (48:49-(40:41+56:57))

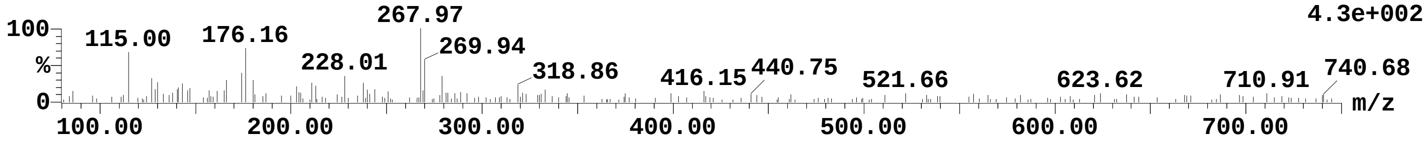

Peak ID	Compound	Time	Mass Found
21	Found	3.66	347.48

Combine (52:53-(43+61:62))

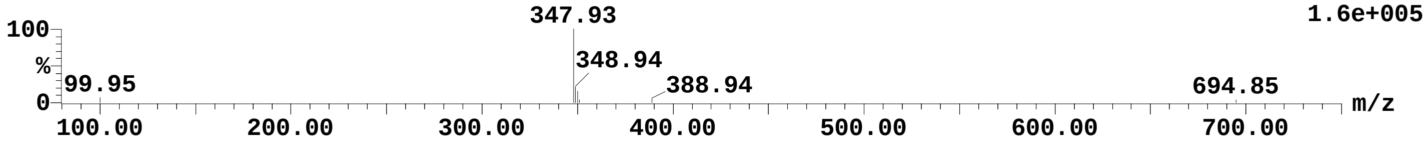

Peak ID	Compound	Time	Mass Found
22	Found	3.86	347.48

Combine (55:56-(47:48+63:64))

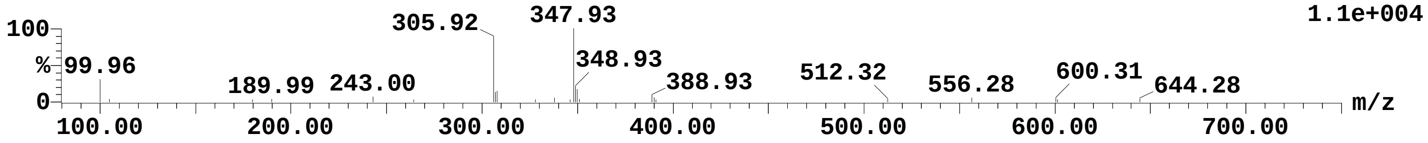

Peak ID	Compound	Time	Mass Found
23	Found	3.97	347.48

Combine (56:58-(49:50+65:66))

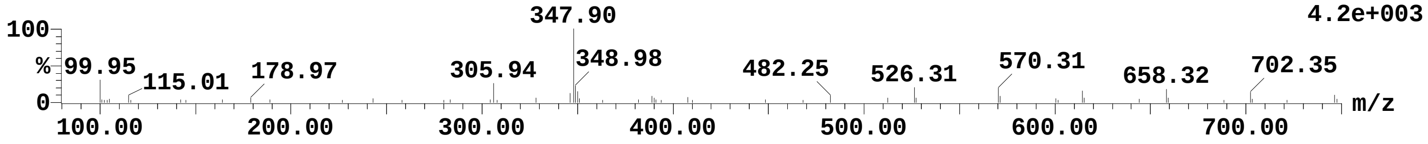

Peak ID	Compound	Time	Mass Found
24		4.08	

Combine (58:59-(50:51+67))

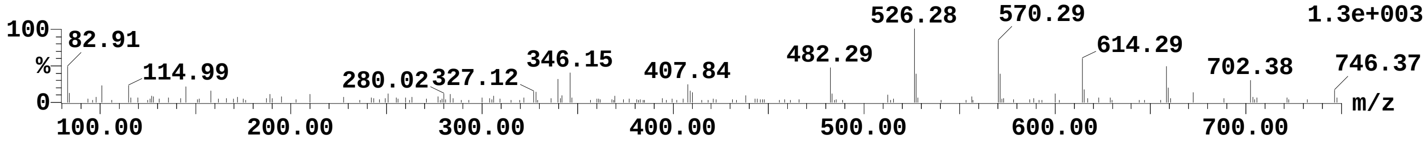

Peak ID	Compound	Time	Mass Found
25		4.26	

Combine (60:62-(52+69:70))

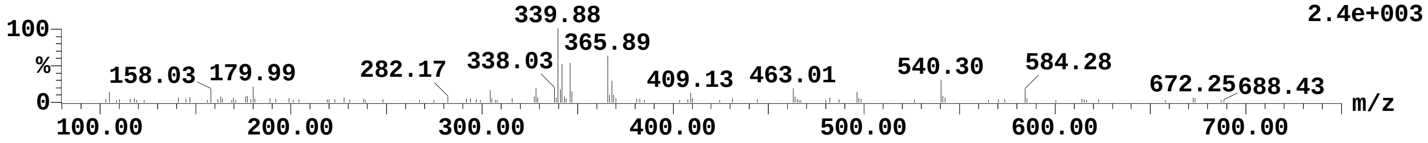

Sample: 5 | Vial:1,2:E,2 | ID:600/38/2
File:337104 | Date:18-Mar-2011 | Time:09:19:17
Description:1_HTS13968; 2_ | Submitter:davec

Printed: Fri Mar 18 09:32:57 2011

Sample Report (continued):

Peak ID	Compound	Time	Mass Found
26		4.37	

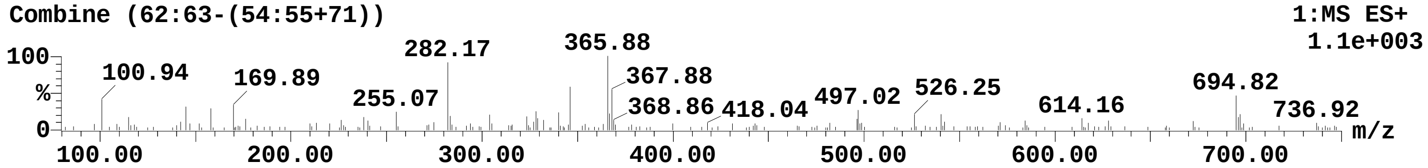

Peak ID	Compound	Time	Mass Found
27		4.52	

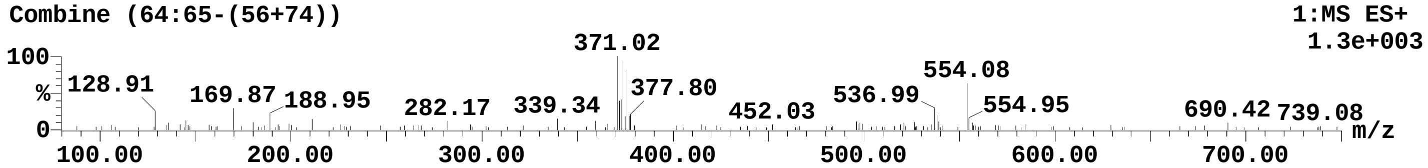

Peak ID	Compound	Time	Mass Found
28		4.71	

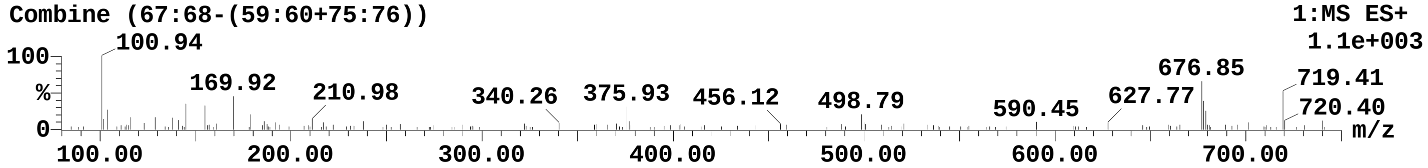

Peak ID	Compound	Time	Mass Found
29		4.82	

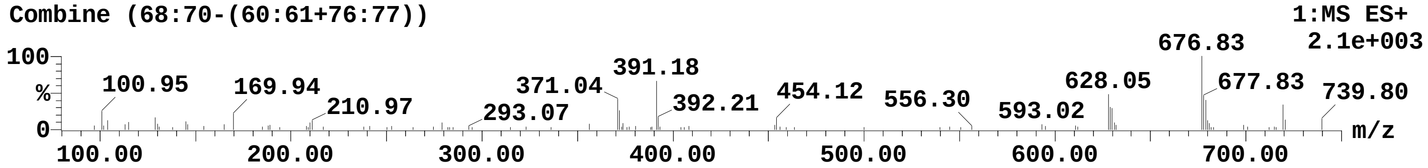

Peak ID	Compound	Time	Mass Found
30		4.90	

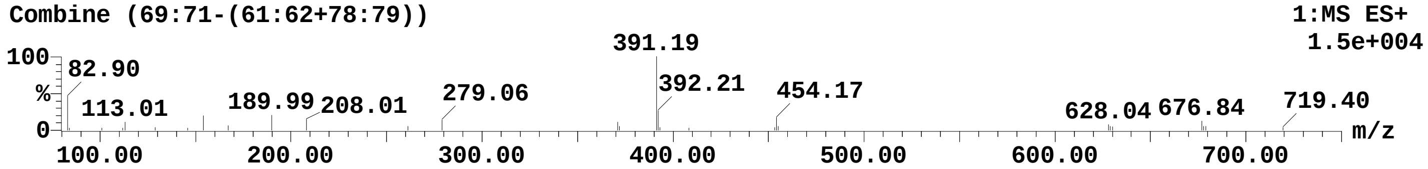

Peak ID	Compound	Time	Mass Found
31		4.98	

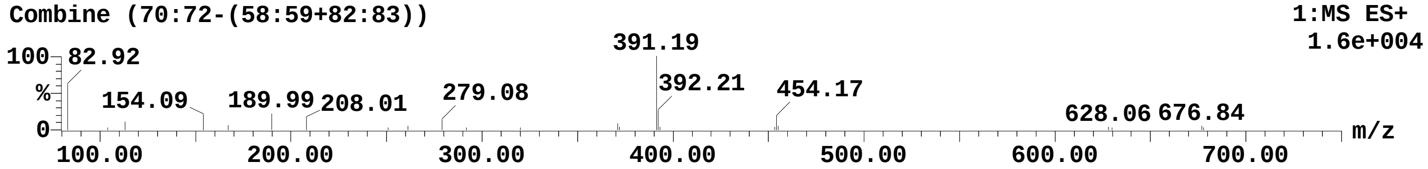

Sample: 5 | Vial:1,2:E,2 | ID:600/38/2
File:337104 | Date:18-Mar-2011 | Time:09:19:17
Description:1_HTS13968; 2_ | Submitter:davec

Printed: Fri Mar 18 09:32:57 2011

Sample Report (continued):

Peak ID	Compound	Time	Mass Found
32		5.13	

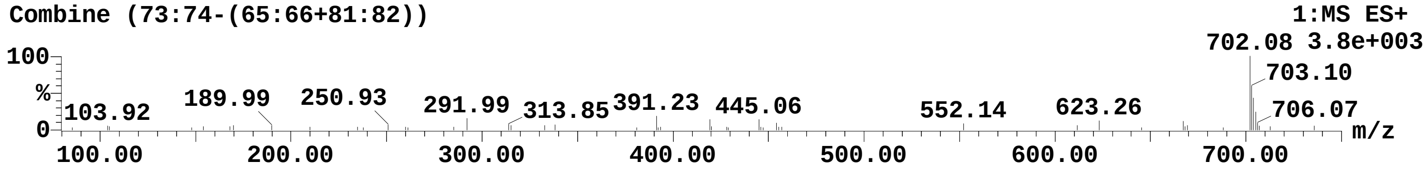

Peak ID	Compound	Time	Mass Found
33		5.24	

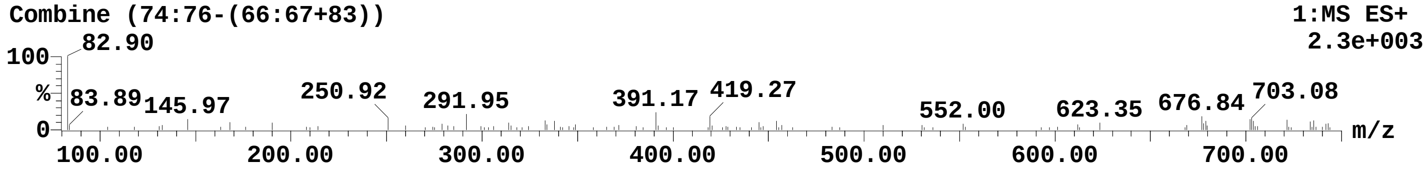

Peak ID	Compound	Time	Mass Found
34		5.34	

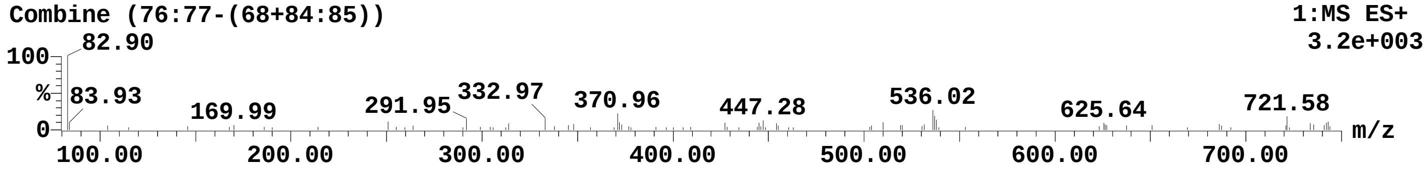

Peak ID	Compound	Time	Mass Found
35	Found	5.45	347.48

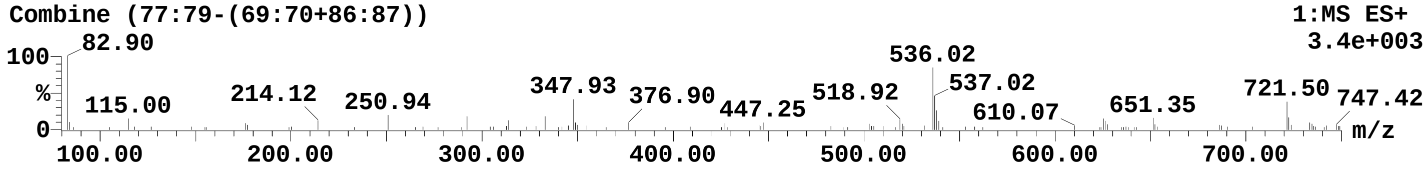

Peak ID	Compound	Time	Mass Found
36		5.55	

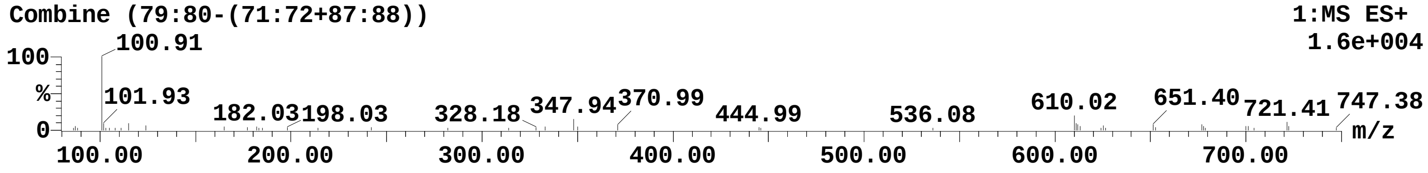

Peak ID	Compound	Time	Mass Found
37		5.66	

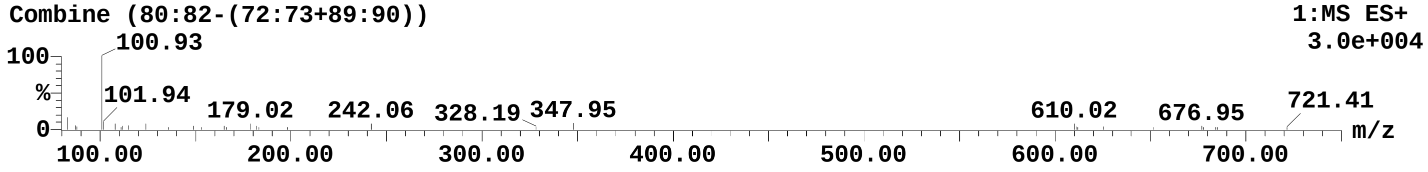

Sample Report (continued):

Peak ID	Compound	Time	Mass Found
38		5.76	

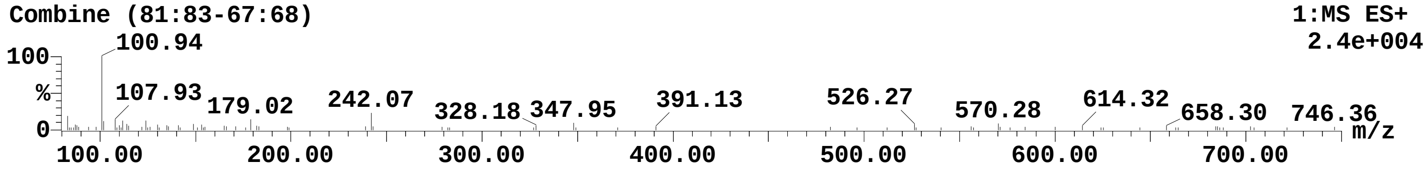

Peak ID	Compound	Time	Mass Found
39		5.87	

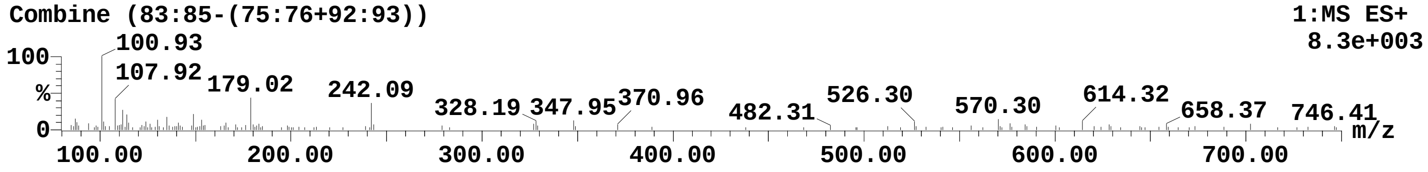

Peak ID	Compound	Time	Mass Found
40		5.96	

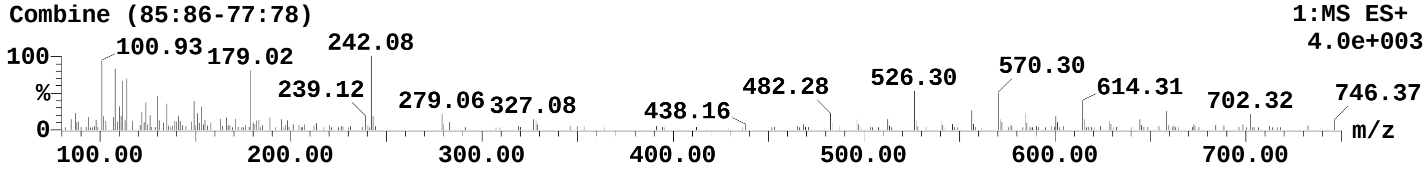

Peak ID	Compound	Time	Mass Found
41		6.07	

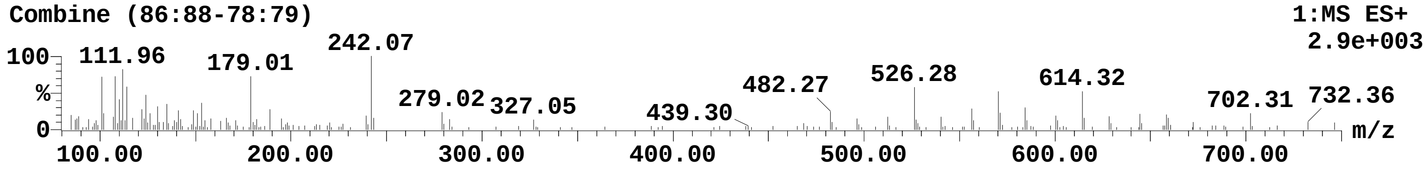

Peak ID	Compound	Time	Mass Found
42		6.20	

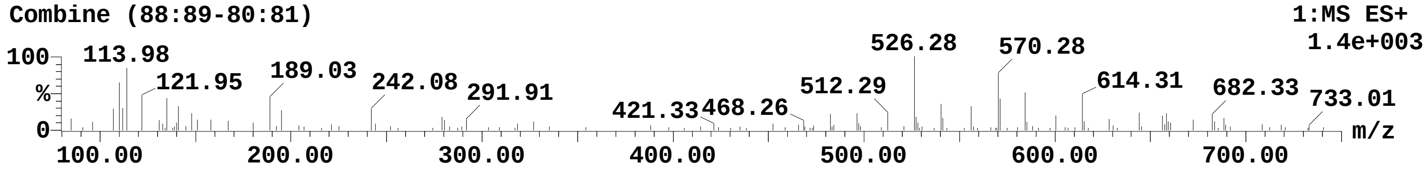

Peak ID	Compound	Time	Mass Found
43		6.21	

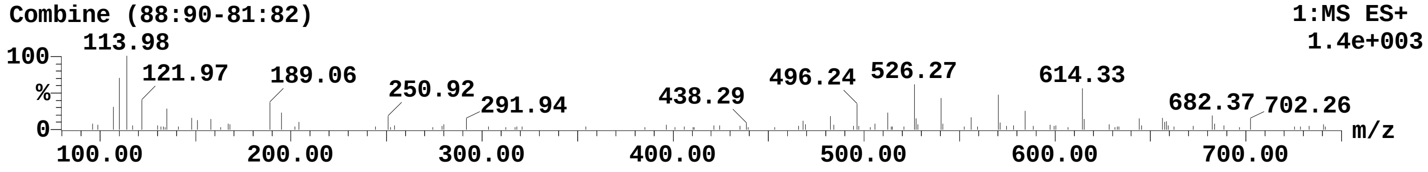

Sample: 5	Vial:1,2:E,2	ID:600/38/2
File:337104	Date:18-Mar-2011	Time:09:19:17
Description:1_HTS13968; 2_	Submitter:davec	

Printed: Fri Mar 18 09:32:57 2011

Sample Report (continued):

Peak ID	Compound	Time	Mass Found
44		6.41	

Combine (92:93-84:85)

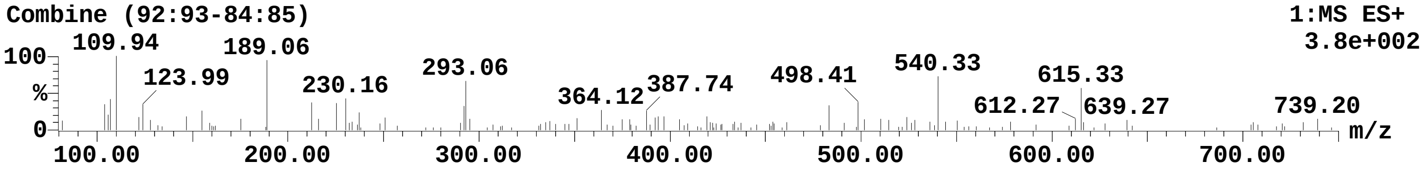

Peak ID	Compound	Time	Mass Found
1		0.21	

Combine (2:3-10:11)

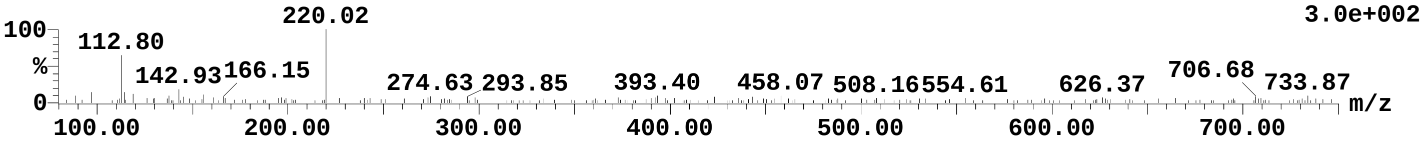

Peak ID	Compound	Time	Mass Found
2		0.26	

Combine (3:4-11:12)

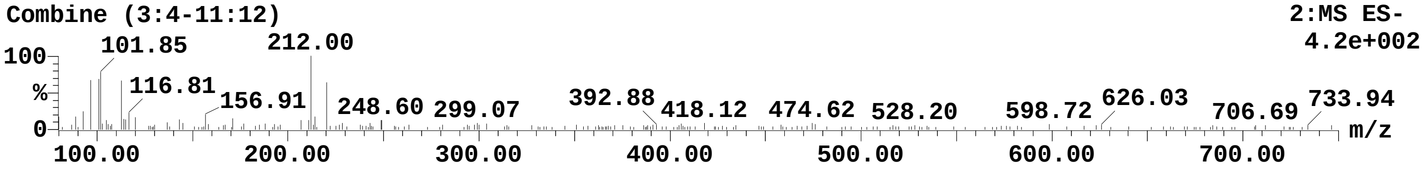

Peak ID	Compound	Time	Mass Found
3		0.31	

Combine (3:5-11:12)

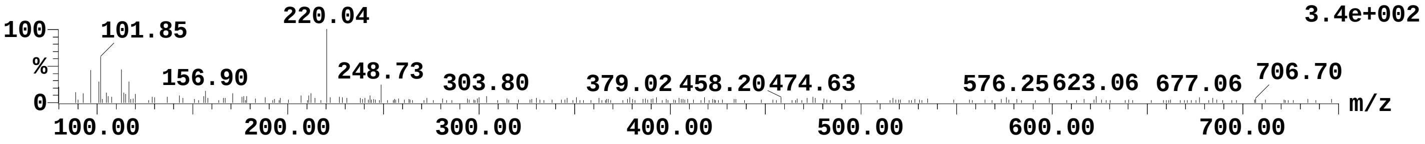

Peak ID	Compound	Time	Mass Found
4		0.35	

Combine (4:5-13)

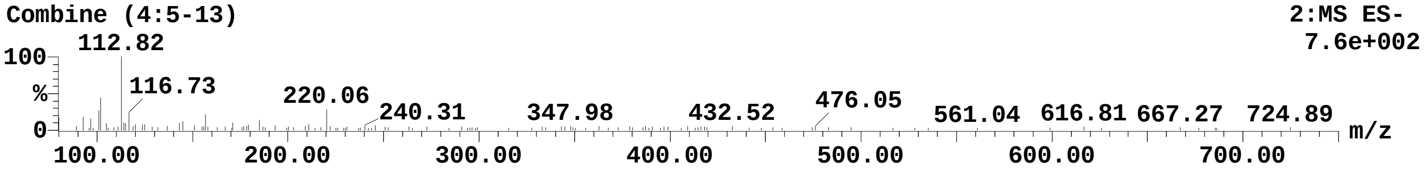

Peak ID	Compound	Time	Mass Found
5	Found	0.53	347.48

Combine (6:8-16:17)

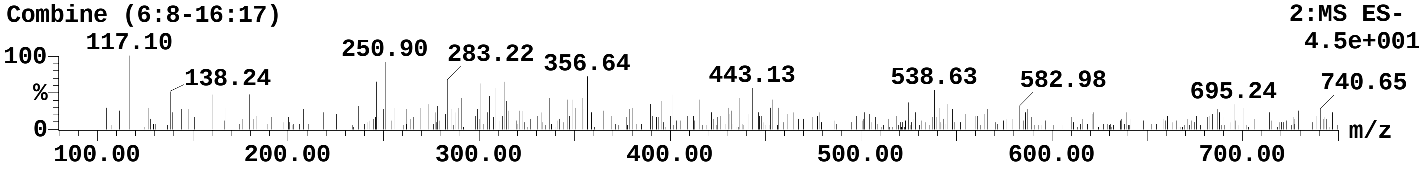

Sample: 5 | Vial:1,2:E,2 | ID:600/38/2
File:337104 | Date:18-Mar-2011 | Time:09:19:17
Description:1_HTS13968; 2_ | Submitter:davec

Printed: Fri Mar 18 09:32:57 2011

Sample Report (continued):

Peak ID	Compound	Time	Mass Found
6		0.88	

Combine (11:13-(1:2+33)) 2:MS ES-

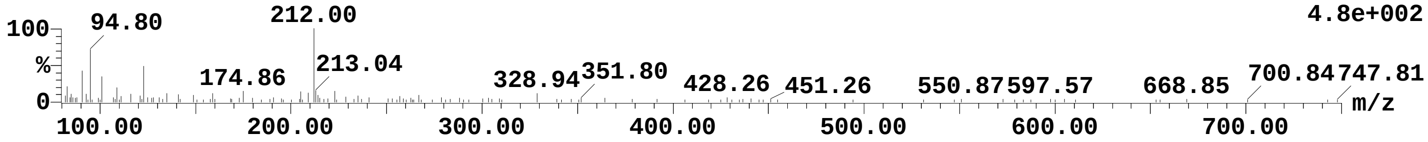

Peak ID	Compound	Time	Mass Found
8			

Combine (24:26-(3:4+38:39)) 2:MS ES-

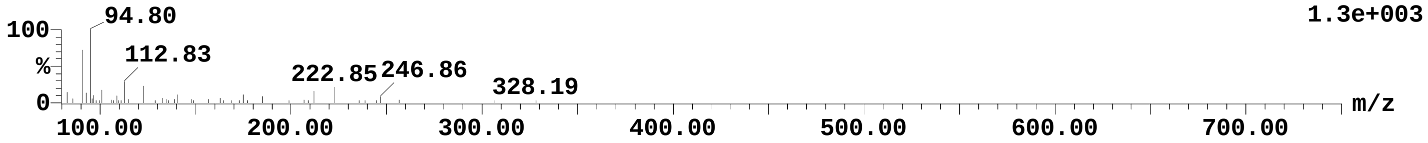

Peak ID	Compound	Time	Mass Found
9		1.85	

Combine (25:27-(18+34:35)) 2:MS ES-

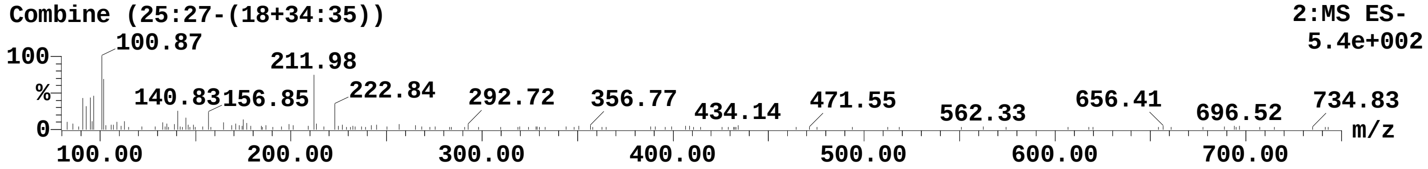

Peak ID	Compound	Time	Mass Found
10		1.91	

Combine (26:28-(19:20+35:36)) 2:MS ES-

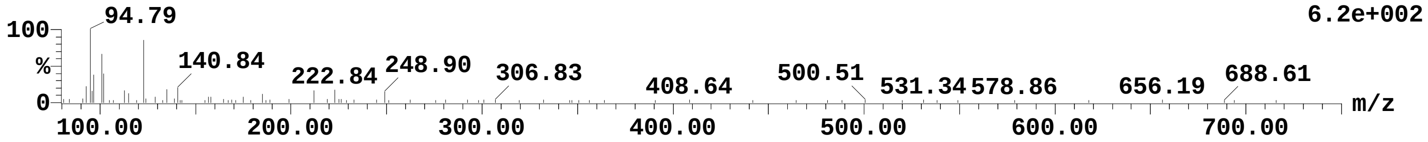

Peak ID	Compound	Time	Mass Found
11		2.08	

Combine (29:30-(20:21+37)) 2:MS ES-

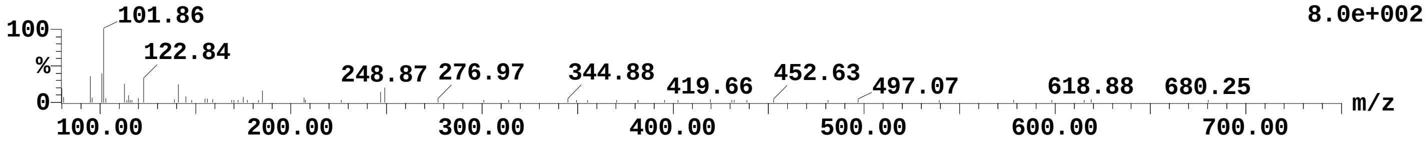

Peak ID	Compound	Time	Mass Found
12		2.11	

Combine (29:30-(22+39)) 2:MS ES-

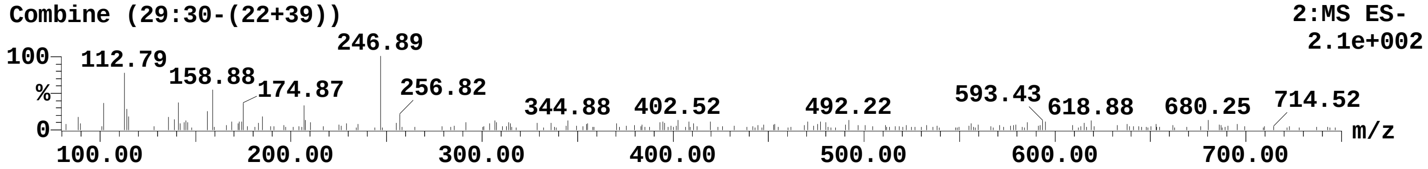

Sample: 5 | Vial:1,2:E,2 | ID:600/38/2
File:337104 | Date:18-Mar-2011 | Time:09:19:17
Description:1_HTS13968; 2_ | Submitter:davec

Printed: Fri Mar 18 09:32:57 2011

Sample Report (continued):

Peak ID	Compound	Time	Mass Found
13		2.37	

Combine (33:34-(24:25+42:43))

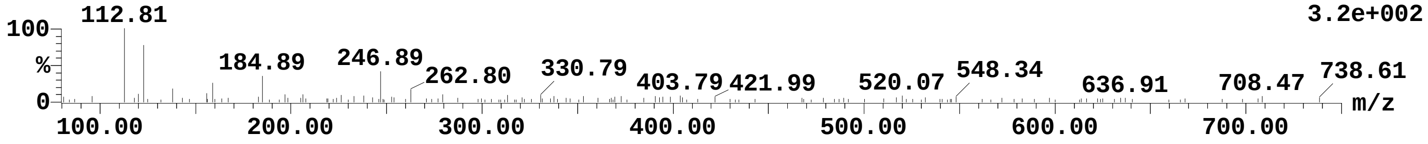

Peak ID	Compound	Time	Mass Found
14		2.60	

Combine (36:37-(28:29+44:45))

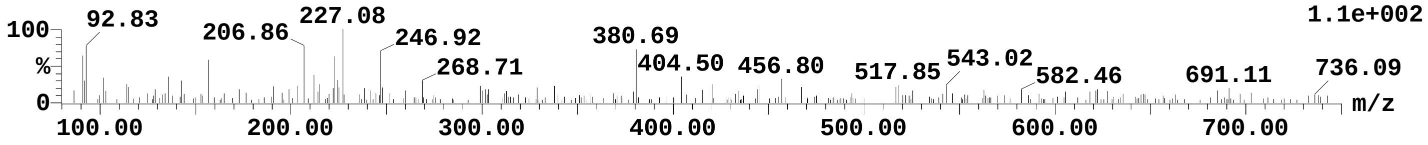

Peak ID	Compound	Time	Mass Found
15		2.66	

Combine (37:38-(29:30+45:46))

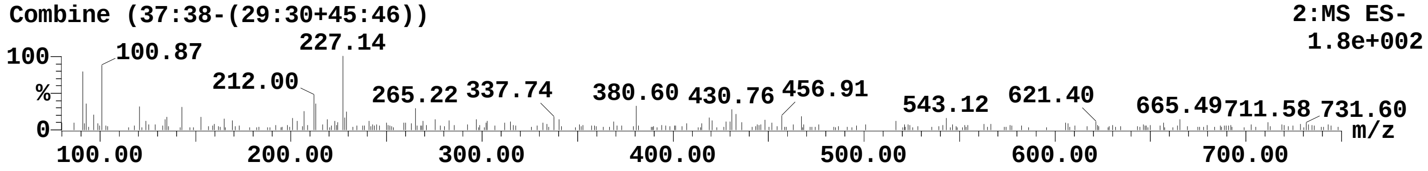

Peak ID	Compound	Time	Mass Found
16		2.79	

Combine (39:40-(30:31+48))

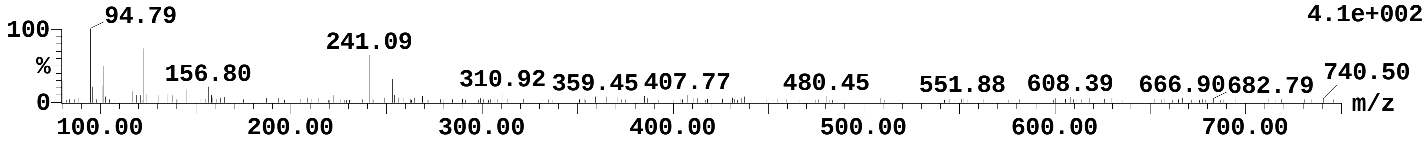

Peak ID	Compound	Time	Mass Found
17		3.02	

Combine (42:44-(33+51))

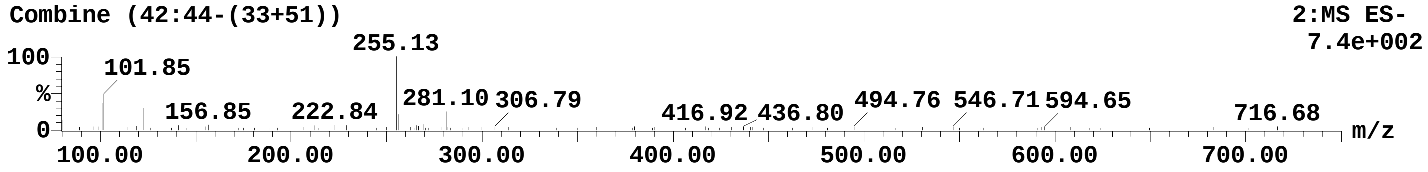

Peak ID	Compound	Time	Mass Found
18		3.11	

Combine (43:45-(36+52:53))

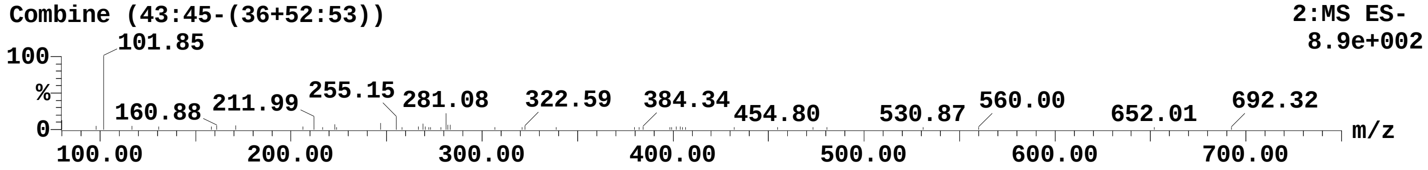

Sample: 5 | Vial:1,2:E,2 | ID:600/38/2
File:337104 | Date:18-Mar-2011 | Time:09:19:17
Description:1_HTS13968; 2_ | Submitter:davec

Printed: Fri Mar 18 09:32:57 2011

Sample Report (continued):

Peak ID	Compound	Time	Mass Found
19		3.29	

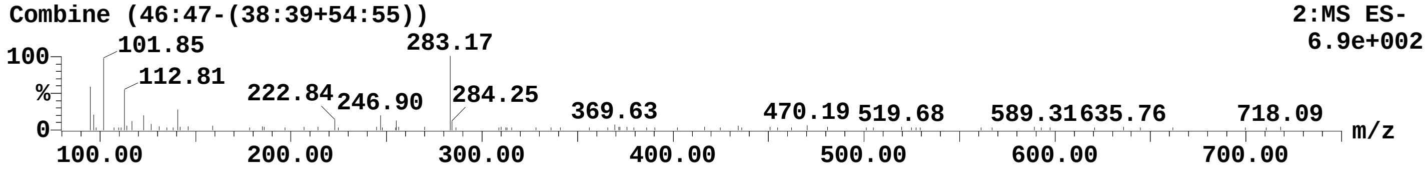

Peak ID	Compound	Time	Mass Found
20		3.37	

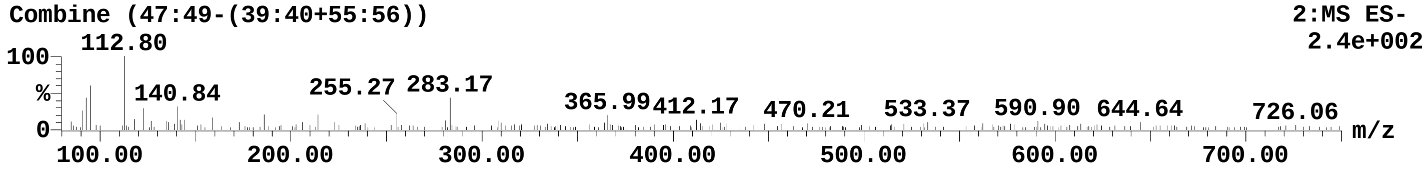

Peak ID	Compound	Time	Mass Found
21		3.66	

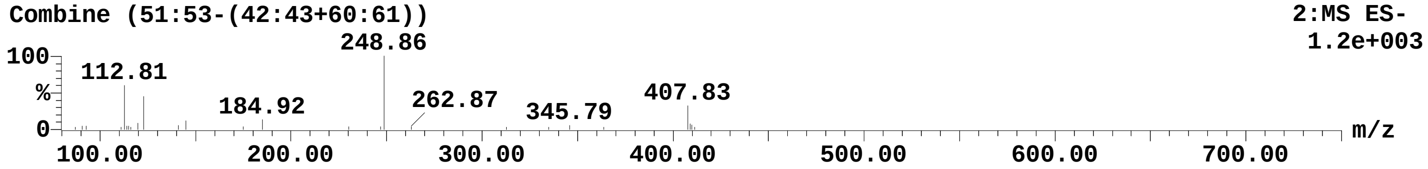

Peak ID	Compound	Time	Mass Found
22		3.86	

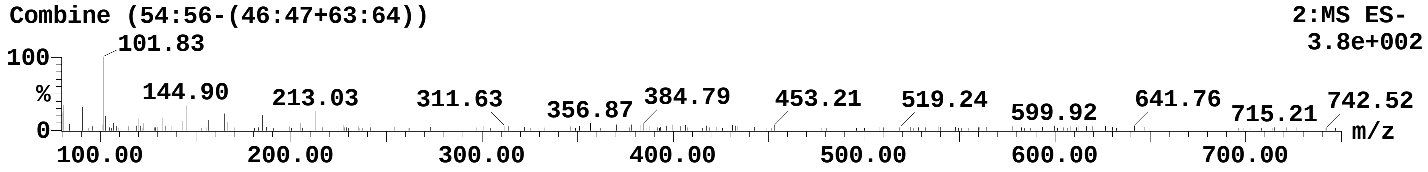

Peak ID	Compound	Time	Mass Found
23		3.97	

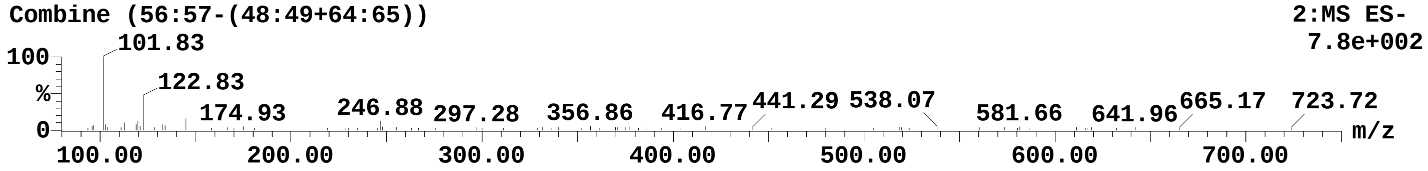

Peak ID	Compound	Time	Mass Found
24		4.08	

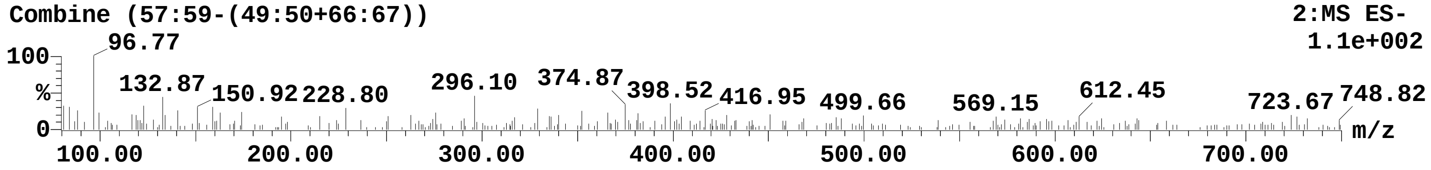

Sample Report (continued):

Peak ID	Compound	Time	Mass Found
25		4.26	

Combine (60:61-(51:52+69:70))

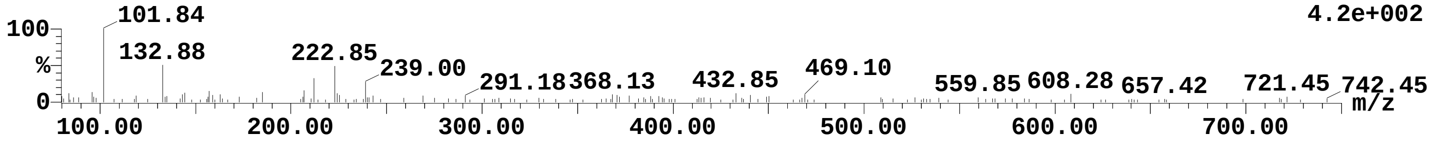

Peak ID	Compound	Time	Mass Found
26	Found	4.37	347.48

Combine (61:63-(54:55+70:71))

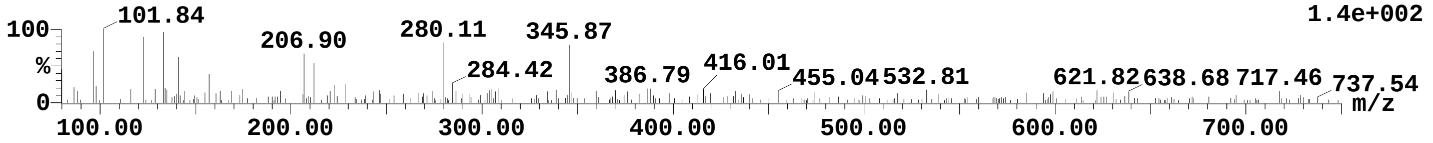

Peak ID	Compound	Time	Mass Found
27		4.52	

Combine (64:65-(55:56+73:74))

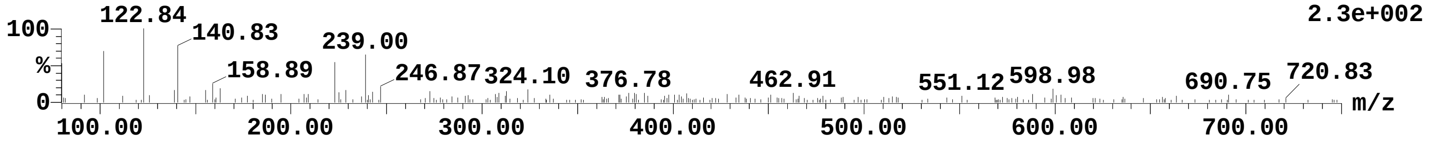

Peak ID	Compound	Time	Mass Found
28		4.71	

Combine (66:68-(58:59+75))

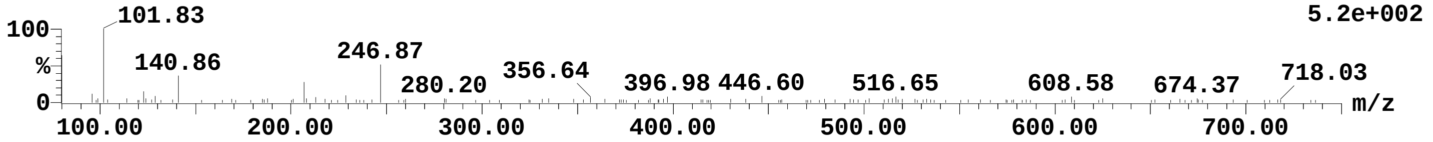

Peak ID	Compound	Time	Mass Found
29		4.82	

Combine (68:69-(60+76:77))

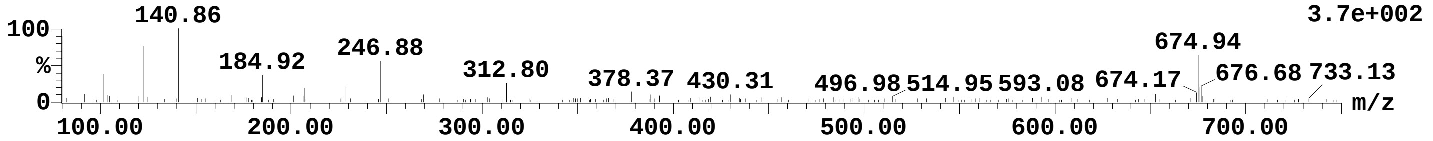

Peak ID	Compound	Time	Mass Found
30		4.90	

Combine (69:70-(61:62+77:78))

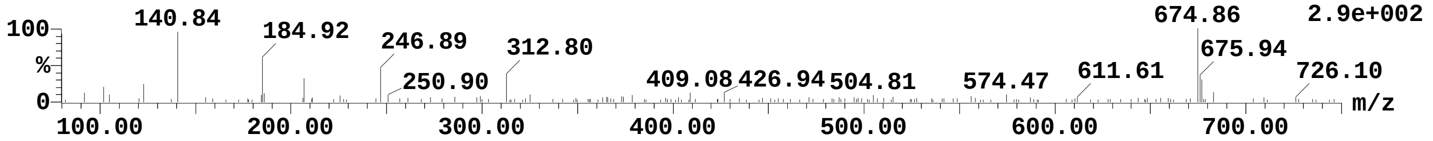

Sample: 5 Vial:1,2:E,2 ID:600/38/2
File:337104 Date:18-Mar-2011 Time:09:19:17
Description:1_HTS13968; 2_ Submitter:davec

Printed: Fri Mar 18 09:32:57 2011

Sample Report (continued):

Peak ID	Compound	Time	Mass Found
31		4.98	

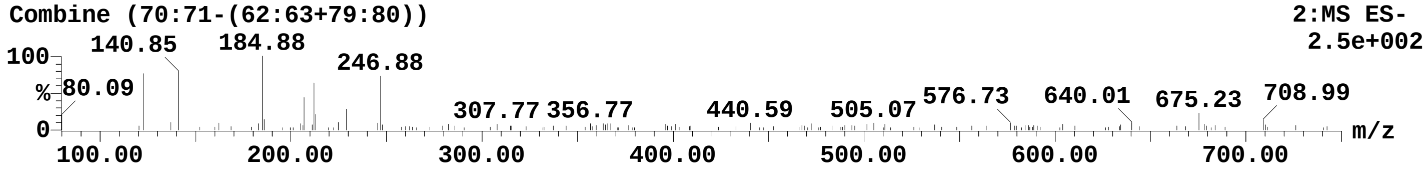

Peak ID	Compound	Time	Mass Found
32		5.13	

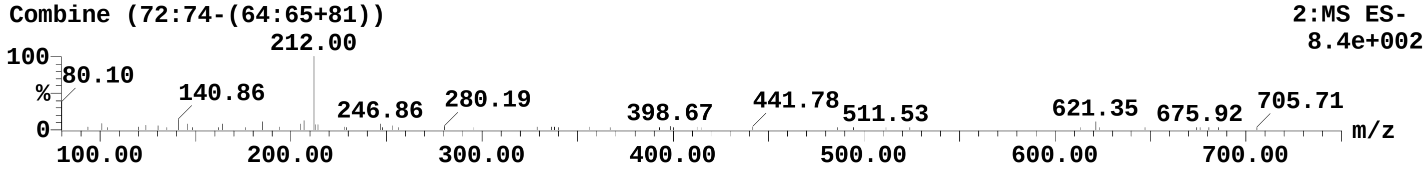

Peak ID	Compound	Time	Mass Found
33		5.24	

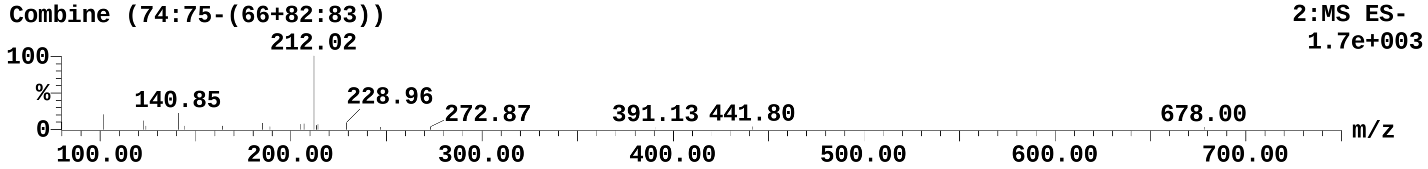

Peak ID	Compound	Time	Mass Found
34		5.34	

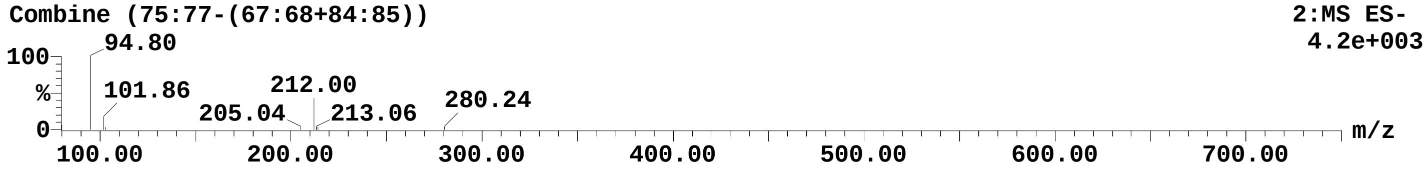

Peak ID	Compound	Time	Mass Found
35		5.45	

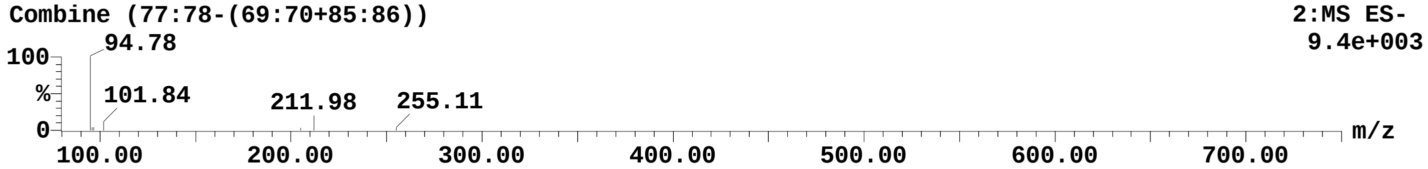

Peak ID	Compound	Time	Mass Found
36		5.55	

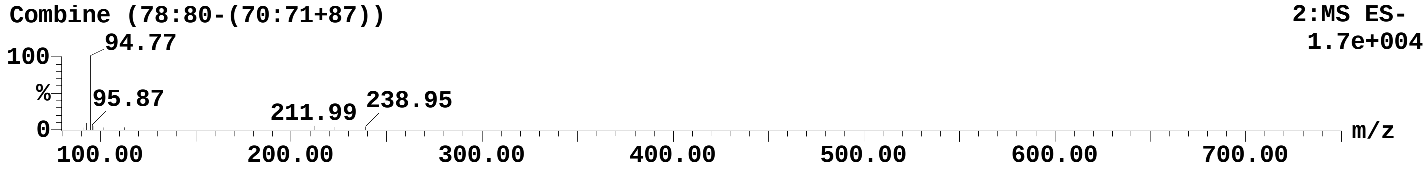

Sample: 5 | Vial:1,2:E,2 | ID:600/38/2
File:337104 | Date:18-Mar-2011 | Time:09:19:17
Description:1_HTS13968; 2_ | Submitter:davec

Printed: Fri Mar 18 09:32:57 2011

Sample Report (continued):

Peak ID	Compound	Time	Mass Found
37		5.66	

Combine (80:81-(72+88:89)) **2:MS ES-**

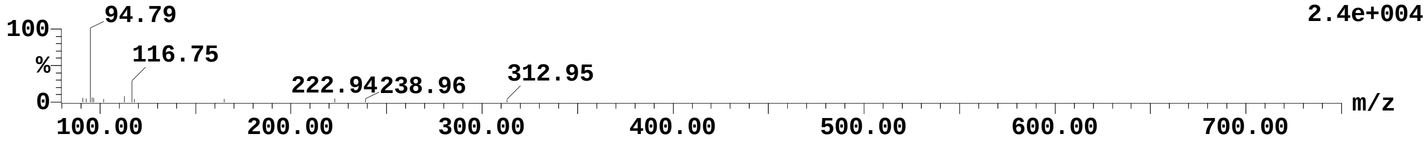

Peak ID	Compound	Time	Mass Found
38		5.76	

Combine (81:83-(73:74+90:91)) **2:MS ES-**

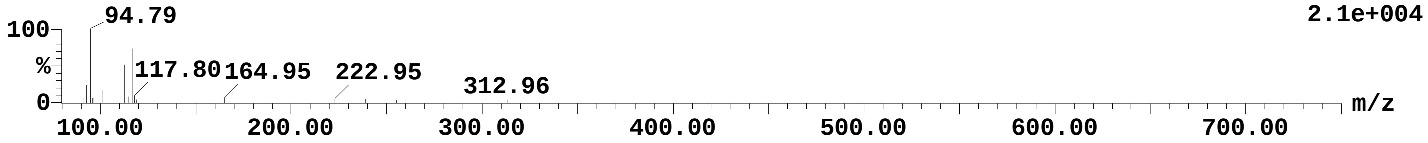

Peak ID	Compound	Time	Mass Found
39		5.87	

Combine (83:84-(75:76+92)) **2:MS ES-**

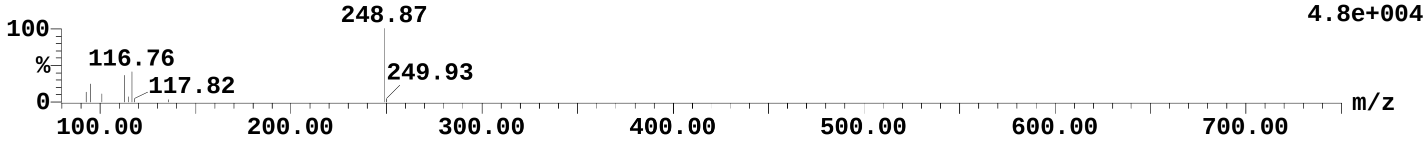

Peak ID	Compound	Time	Mass Found
40		5.96	

Combine (84:86-77) **2:MS ES-**

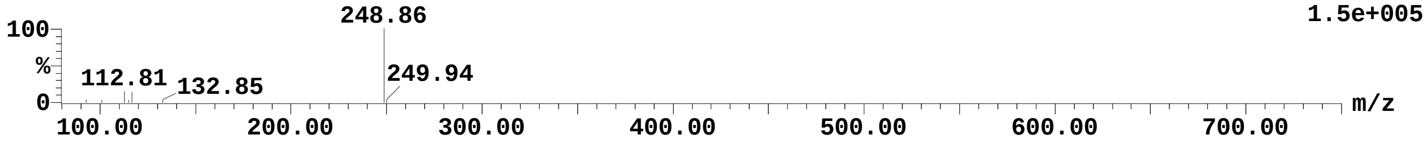

Peak ID	Compound	Time	Mass Found
41		6.07	

Combine (86:88-70:71) **2:MS ES-**

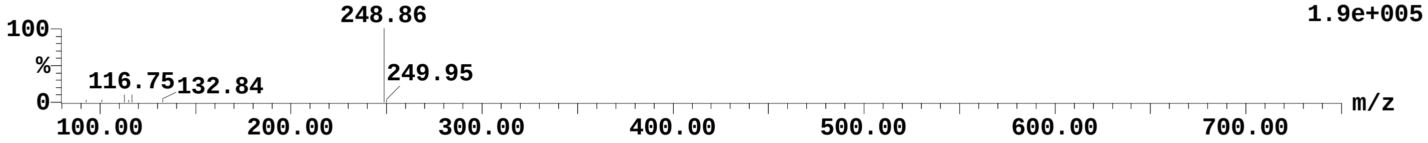

Peak ID	Compound	Time	Mass Found
42		6.20	

Combine (87:89-80) **2:MS ES-**

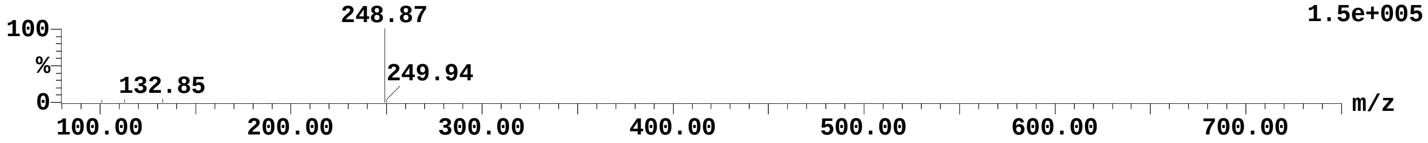

Sample: 5 | Vial:1,2:E,2 | ID:600/38/2
File:337104 | Date:18-Mar-2011 | Time:09:19:17
Description:1_HTS13968; 2_ | Submitter:davec

Printed: Fri Mar 18 09:32:57 2011

Sample Report (continued):

Peak ID	Compound	Time	Mass Found
43		6.21	

Combine (88:89-80:81) 2:MS ES- 1.4e+005

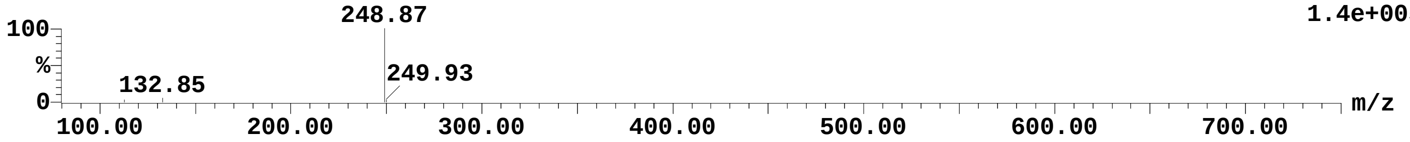

Peak ID	Compound	Time	Mass Found
44		6.41	

Combine (91:92-84:85) 2:MS ES- 6.5e+004

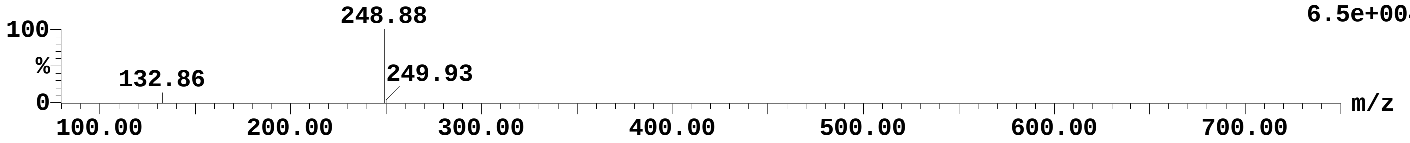

Supplement: Supplementary file 1 — Supplementary Information 1. [file 41598_2024_54655_MOESM1_ESM.zip › Nature SREP/QC_AIMS_files/Proj186.pdf]
