# Supplementary material for: AI is a viable alternative to high throughput screening: a 318-target study
Source: Sci Rep. 2024 Apr 2;14:7526. doi: 10.1038/s41598-024-54655-z (PMC10987645; doi:10.1038/s41598-024-54655-z)
Supplement: Supplementary file 1 — Supplementary Information 1. [file 41598_2024_54655_MOESM1_ESM.zip › Nature SREP/QC_AIMS_files/Proj198.pdf]

MaxPeak: 94.99%
Ret_Time: 1.092 min

6078047

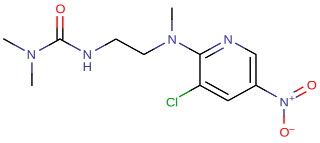

Mol Wt 301.73
Exact Mass 301.1

#	Time	Area%
1	1.092	94.99
2	1.263	3.61
3	1.334	1.41

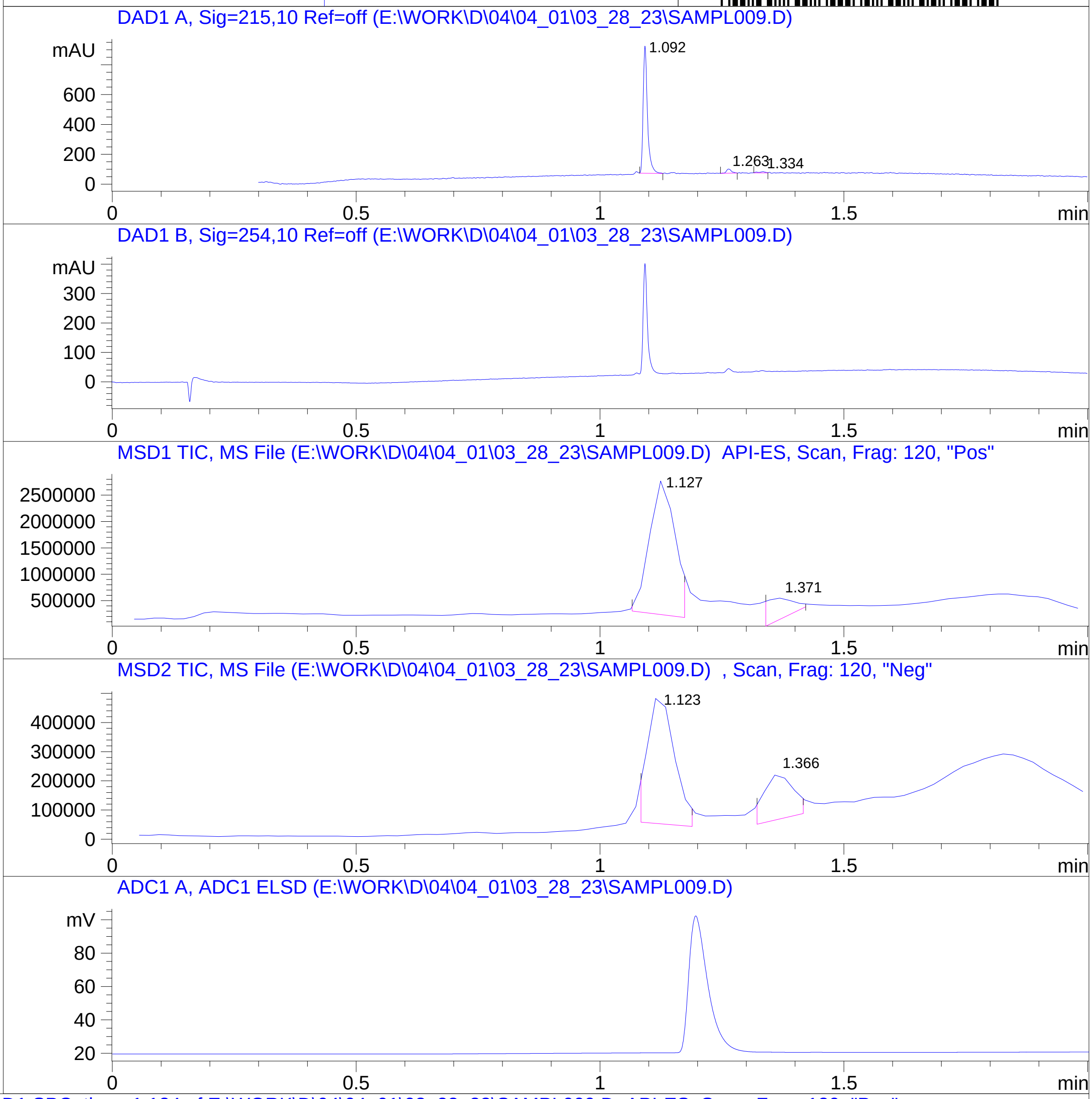

RT 1.127

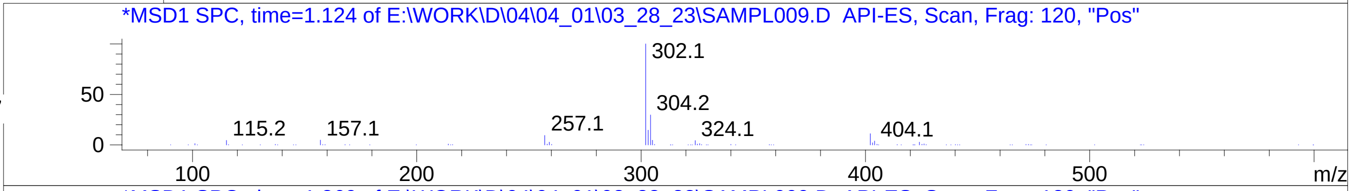

RT 1.371

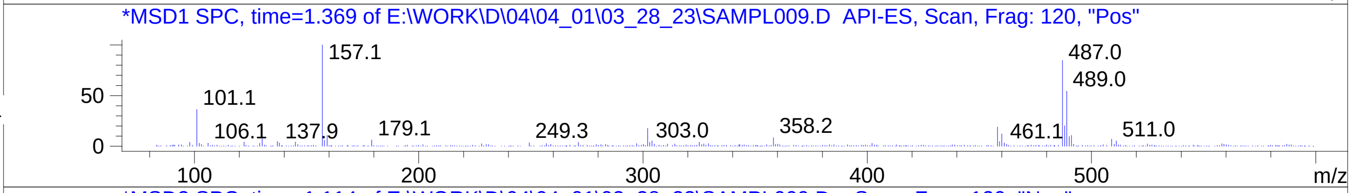

RT 1.123

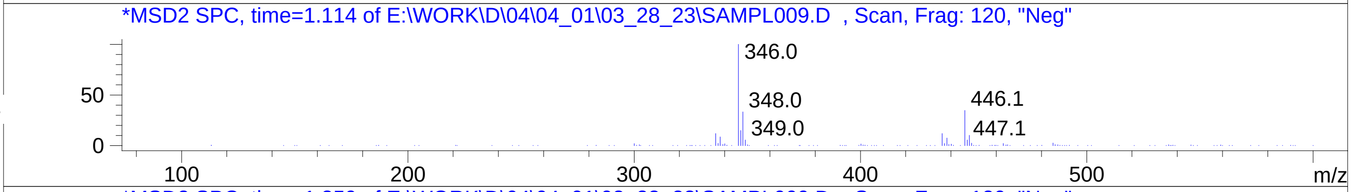

RT 1.366

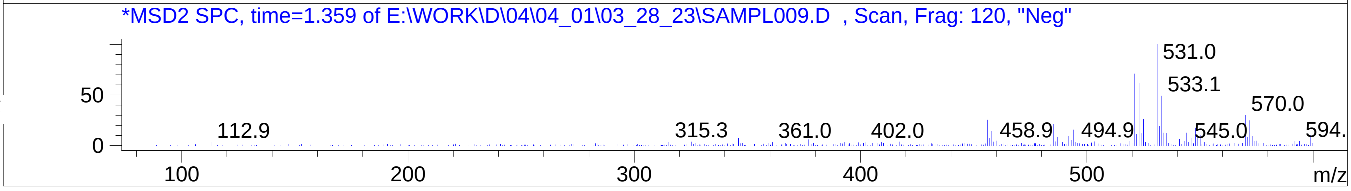

Inj.Date 3/31/2013 K P2-A-09 SL Acq. Method C:\HPCHEM\-> ->
