# Supplementary material for: AI is a viable alternative to high throughput screening: a 318-target study
Source: Sci Rep. 2024 Apr 2;14:7526. doi: 10.1038/s41598-024-54655-z (PMC10987645; doi:10.1038/s41598-024-54655-z)

T6068729

MaxPeak: 93.68%
Ret_Time: 1.557 min

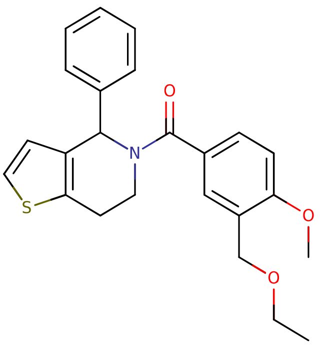

Mol Wt 407.52

Exact Mass 407.19

#	Time	Area%
1	1.299	1.62
2	1.432	2.54
3	1.489	2.15
4	1.557	93.68

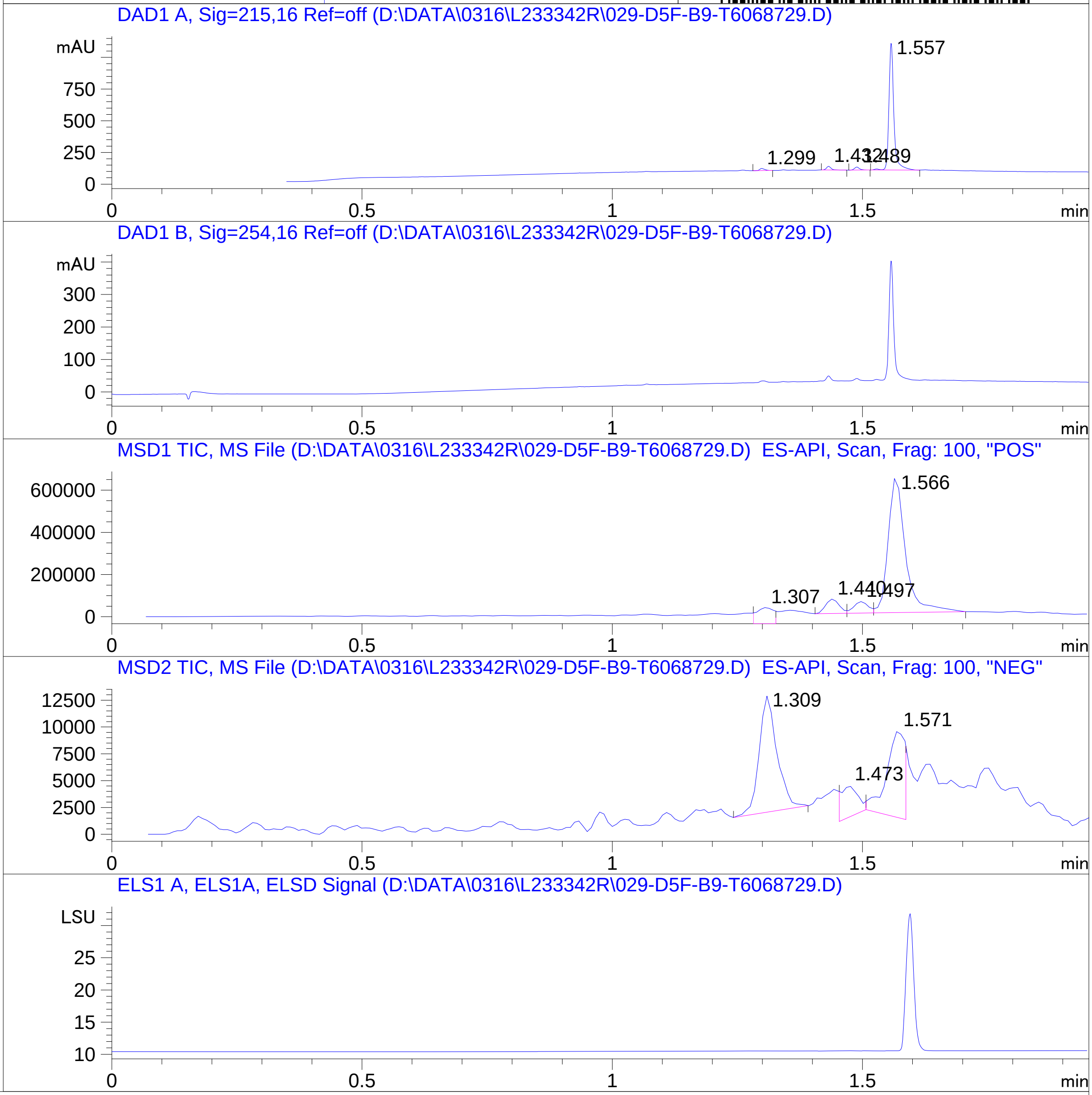

RT 1.307

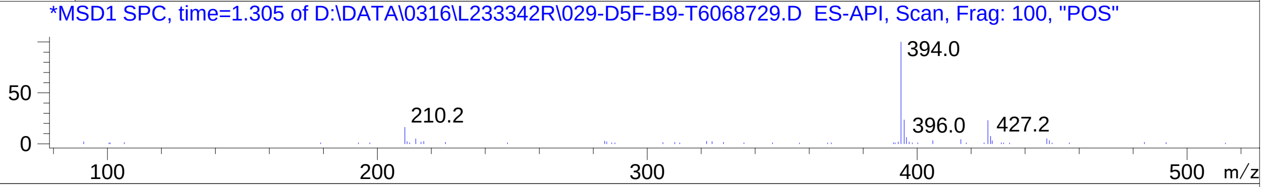

RT 1.440

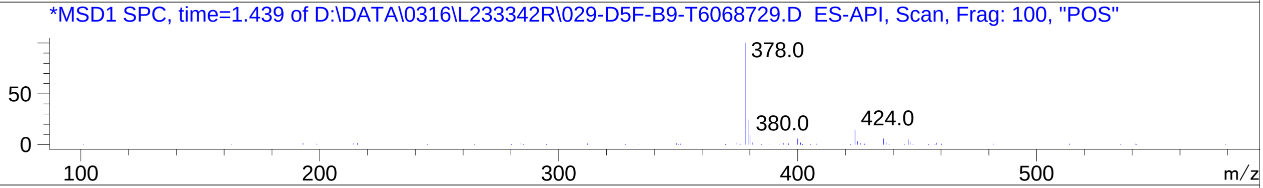

RT 1.497

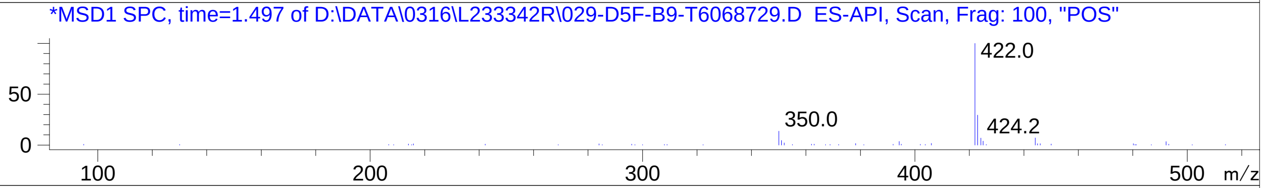

RT 1.566

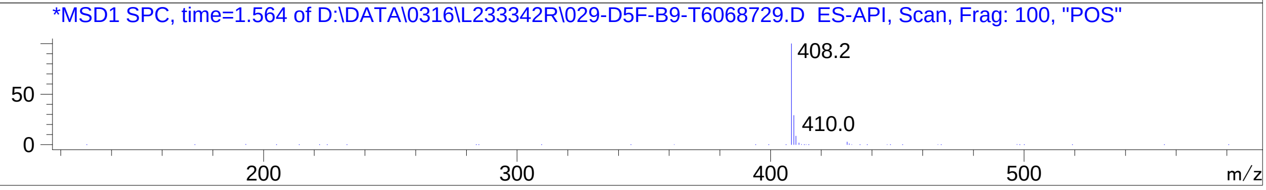

RT 1.309

RT 1.473

RT 1.571

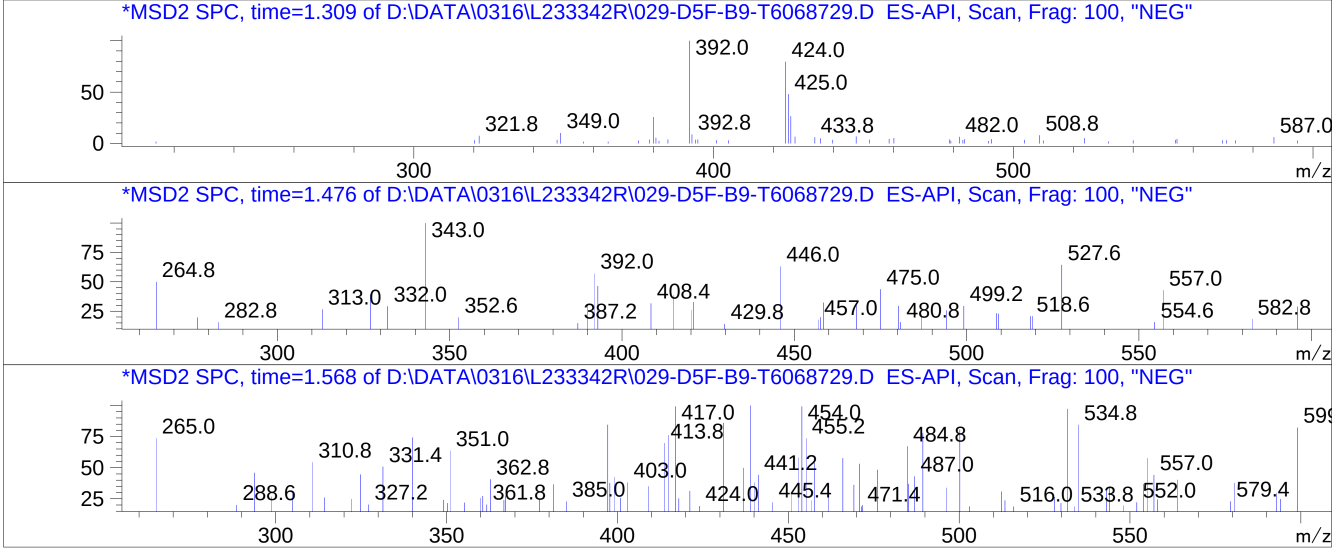

Supplement: Supplementary file 1 — Supplementary Information 1. [file 41598_2024_54655_MOESM1_ESM.zip › Nature SREP/QC_AIMS_files/Proj201.pdf]
