# Supplementary material for: AI is a viable alternative to high throughput screening: a 318-target study
Source: Sci Rep. 2024 Apr 2;14:7526. doi: 10.1038/s41598-024-54655-z (PMC10987645; doi:10.1038/s41598-024-54655-z)
Supplement: Supplementary file 1 — Supplementary Information 1. [file 41598_2024_54655_MOESM1_ESM.zip › Nature SREP/QC_AIMS_files/Proj205.pdf]

MaxPeak: 95.18%
Ret_Time: 1.514 min

T5893449

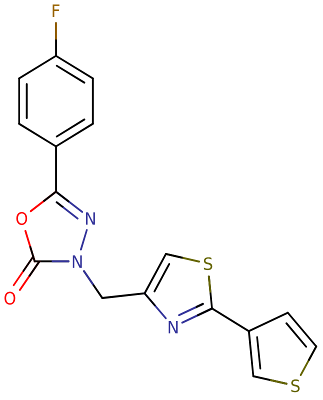

Mol Wt 359.4

Exact Mass 359.02

#	Time	Area%
1	1.514	95.18
2	1.576	1.85
3	1.618	1.62
4	1.680	1.35

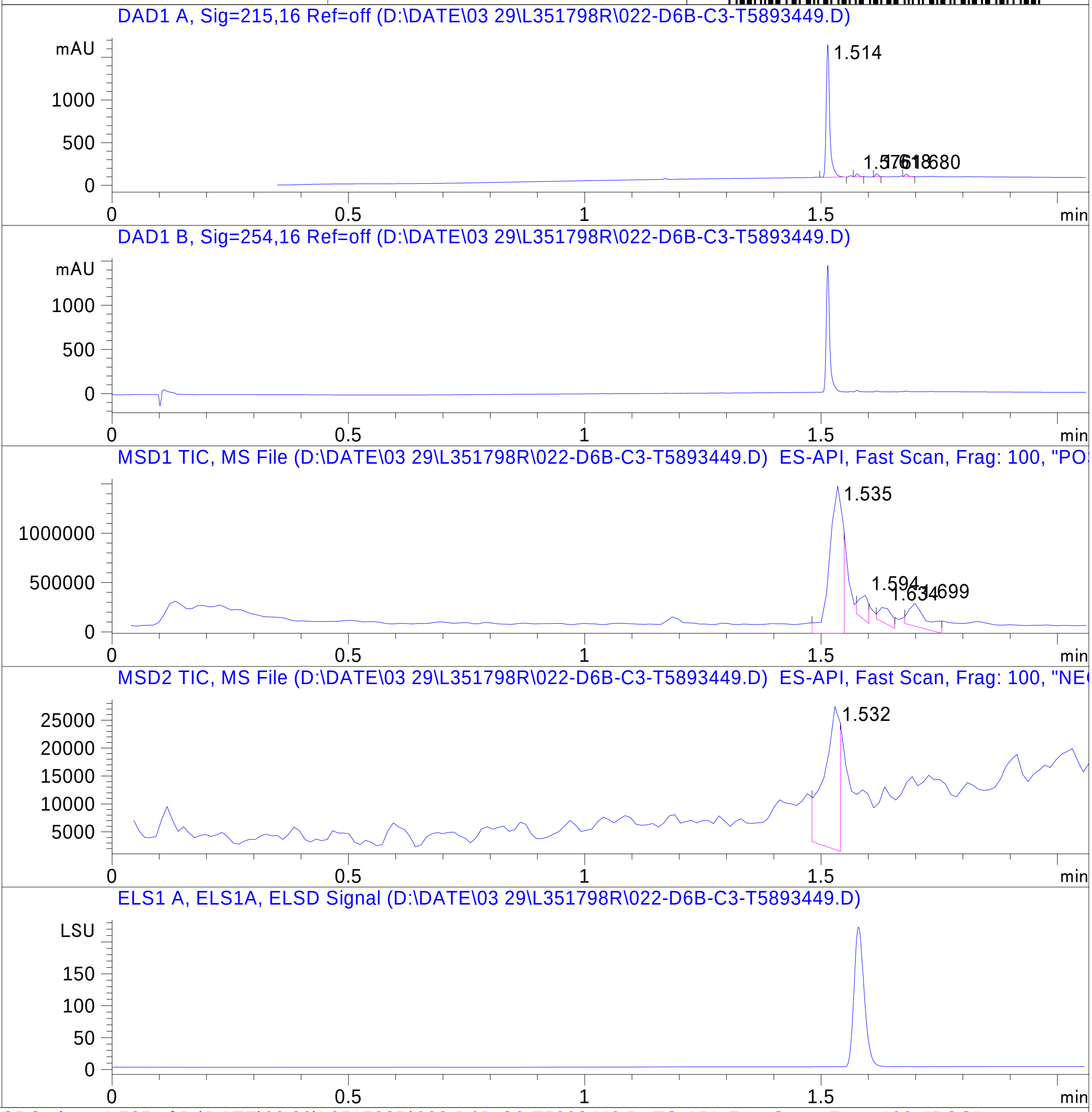

RT 1.535

RT 1.594

RT 1.634

RT 1.699

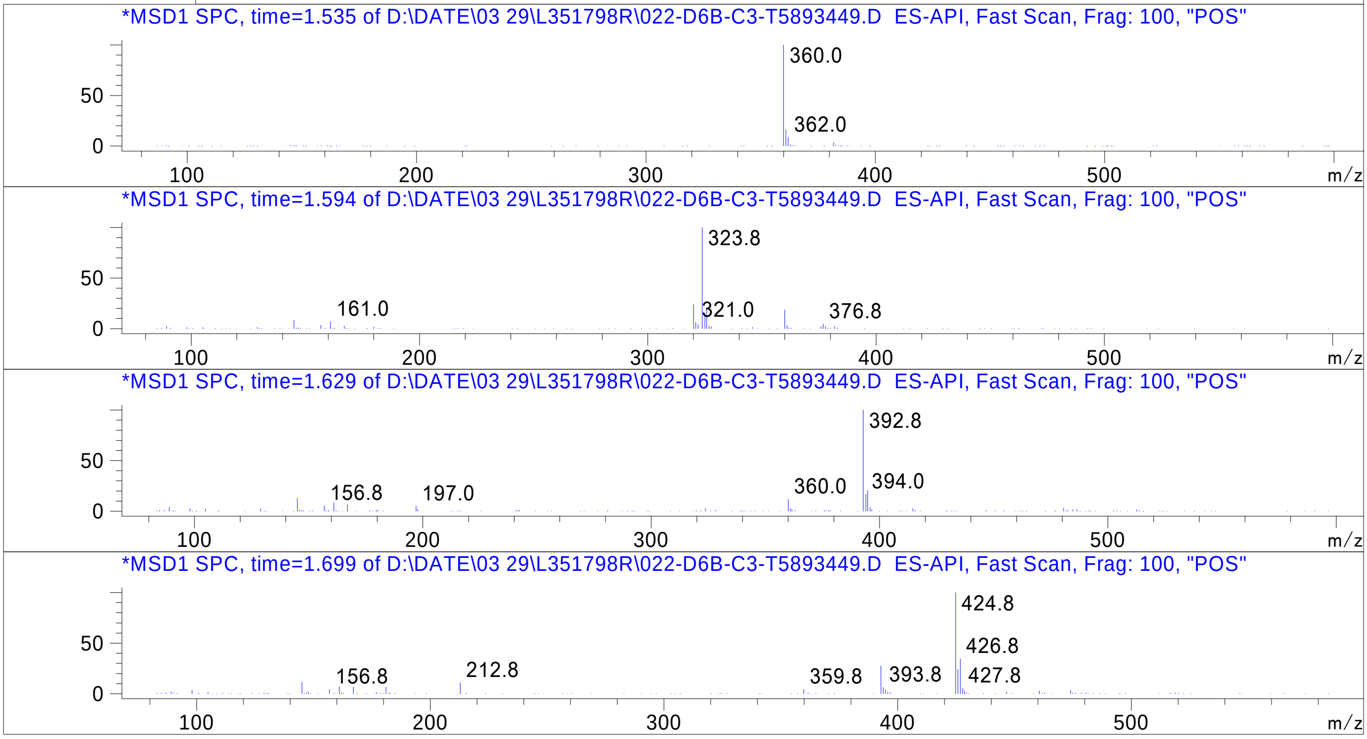

Inj.Date 3/29/2021 E Acq. Method C:\Users\ -> ->

RT 1.532

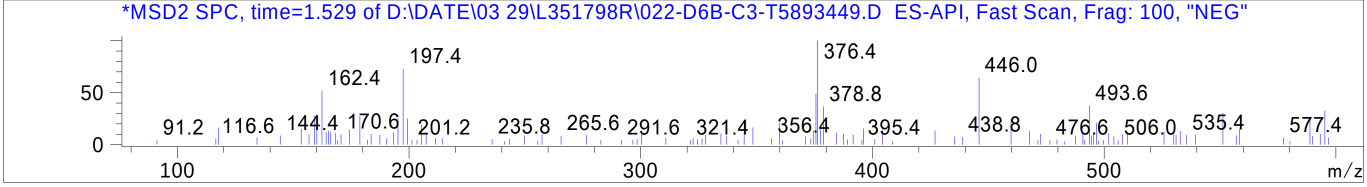

Inj.Date 3/29/2021 E Acq. Method C:\Users\ -> ->
