# Supplementary material for: AI is a viable alternative to high throughput screening: a 318-target study
Source: Sci Rep. 2024 Apr 2;14:7526. doi: 10.1038/s41598-024-54655-z (PMC10987645; doi:10.1038/s41598-024-54655-z)

MaxPeak: 95.55%
Ret_Time: 1.429 min

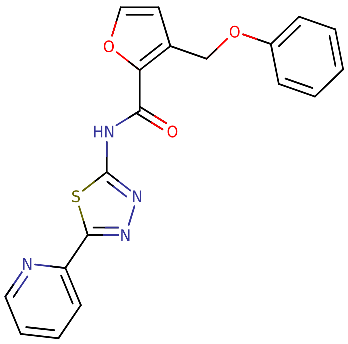

T6636654

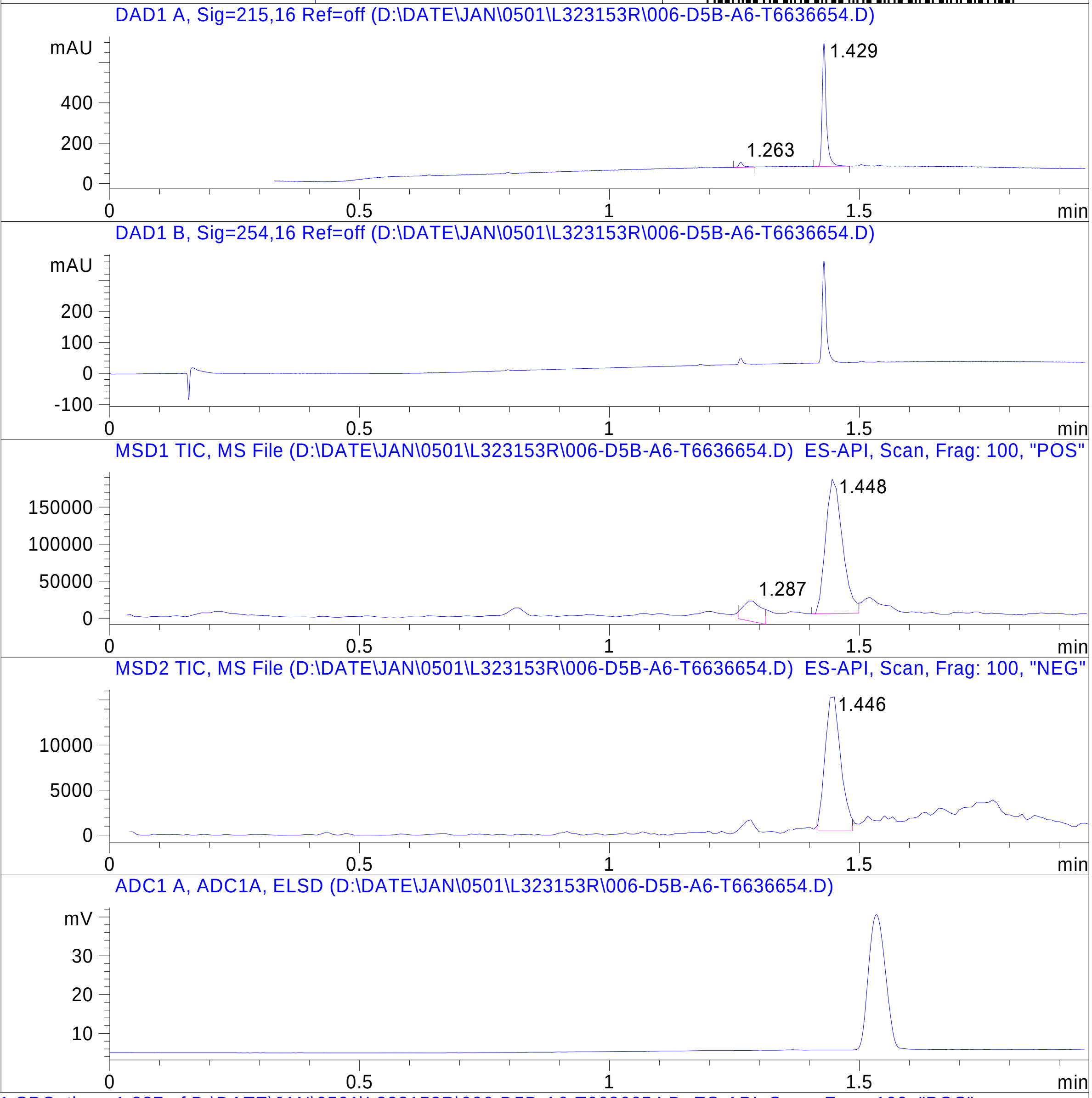

Mol Wt 378.4

Exact Mass 378.08

#	Time	Area%
1	1.263	4.45
2	1.429	95.55

RT 1.287

RT 1.448

RT 1.446

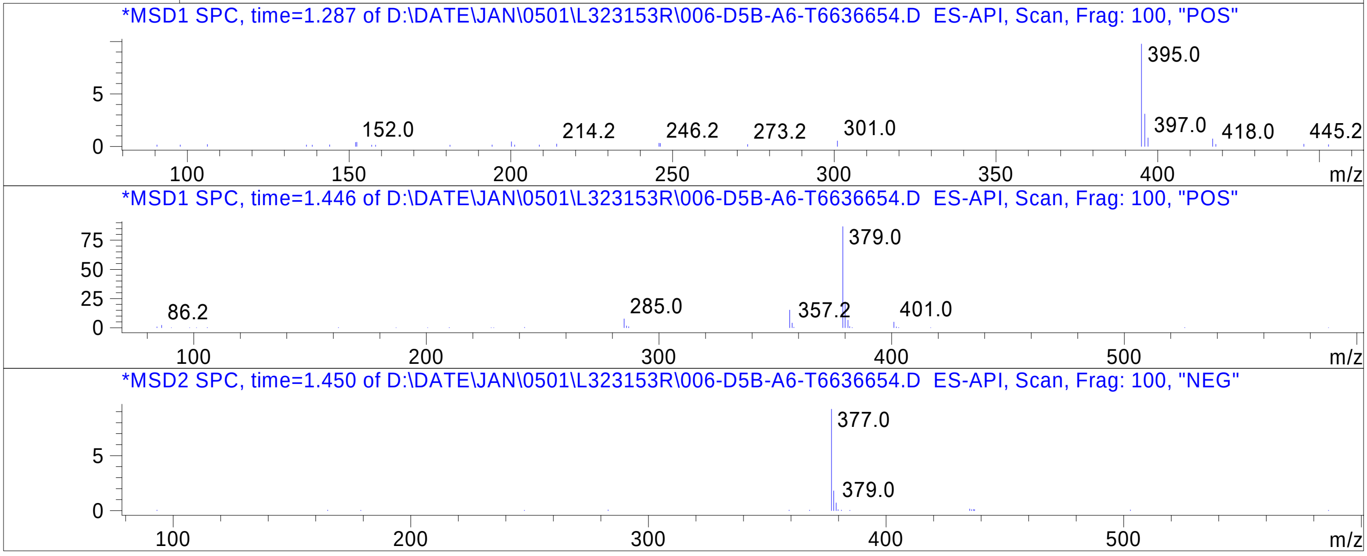

Supplement: Supplementary file 1 — Supplementary Information 1. [file 41598_2024_54655_MOESM1_ESM.zip › Nature SREP/QC_AIMS_files/Proj210.pdf]
