# Supplementary material for: AI is a viable alternative to high throughput screening: a 318-target study
Source: Sci Rep. 2024 Apr 2;14:7526. doi: 10.1038/s41598-024-54655-z (PMC10987645; doi:10.1038/s41598-024-54655-z)
Supplement: Supplementary file 1 — Supplementary Information 1. [file 41598_2024_54655_MOESM1_ESM.zip › Nature SREP/QC_AIMS_files/Proj221.pdf]

2956012

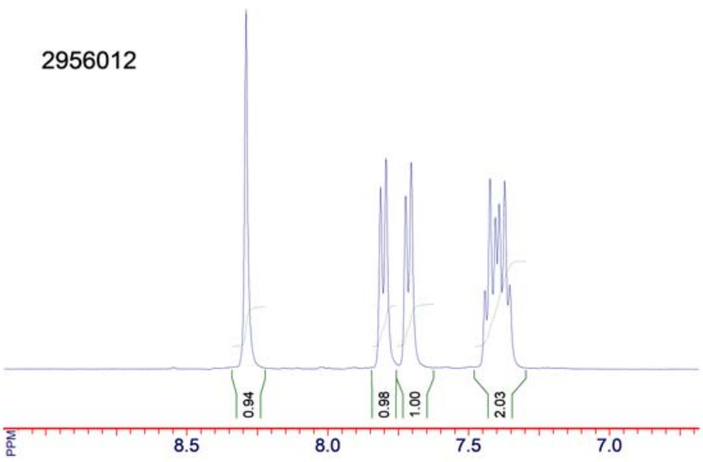

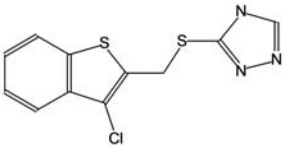

2956012 Zavod $C_{11}H_8ClN_3S_2$
281.78

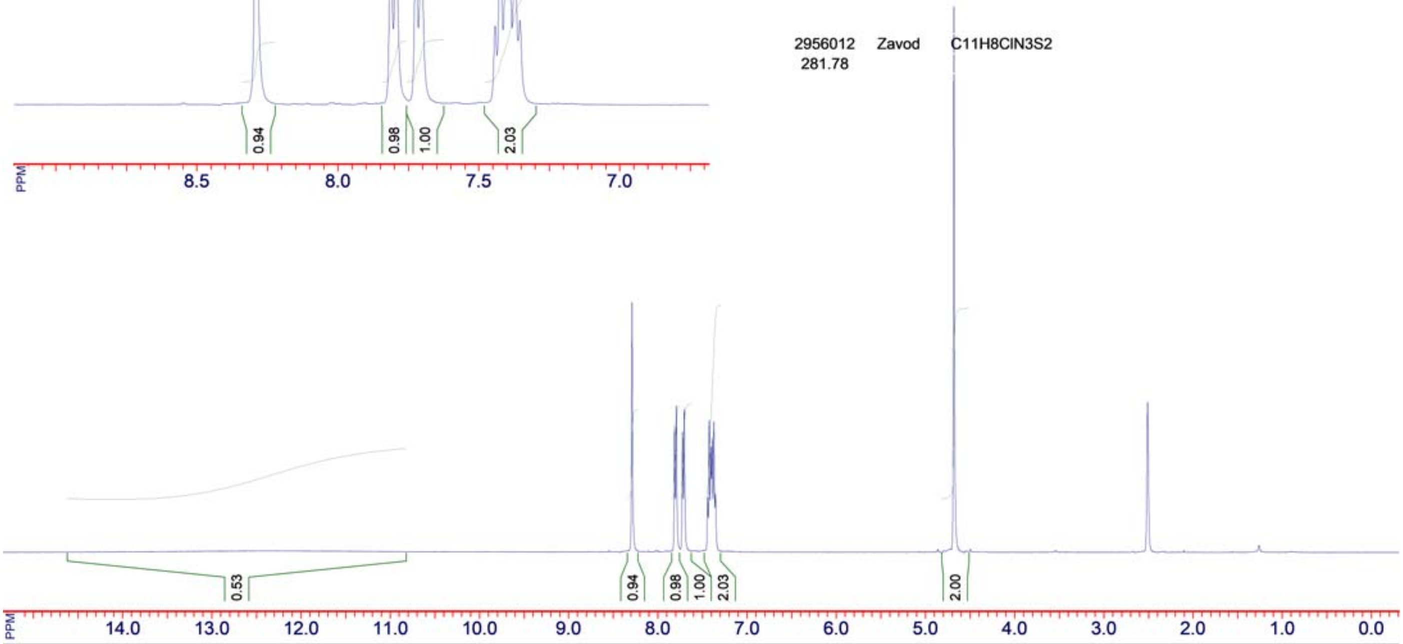

**File name: 2956012**	Operator: Reutova	SF: 399.9703 MHz	NSC: 0	PW: 15.00 usec, RG: 32	SI: 32768
Date: 15-Jul-2009	Solvent: dmso-d6+ccl4	SW: 6803 Hz	TE: 293 K	AQ: 1.33 sec, RD: 0.00 sec	
