# Supplementary material for: AI is a viable alternative to high throughput screening: a 318-target study
Source: Sci Rep. 2024 Apr 2;14:7526. doi: 10.1038/s41598-024-54655-z (PMC10987645; doi:10.1038/s41598-024-54655-z)
Supplement: Supplementary file 1 — Supplementary Information 1. [file 41598_2024_54655_MOESM1_ESM.zip › Nature SREP/QC_AIMS_files/Proj226.pdf]

MaxPeak: 96.22%
Ret_Time: 1.252 min

5633812$1

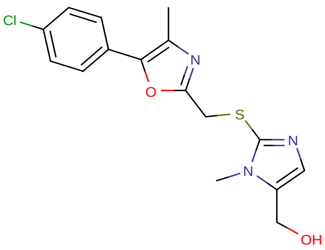

Mol Wt 349.84
Exact Mass 349.08

#	Time	Area%
1	1.221	3.78
2	1.252	96.22

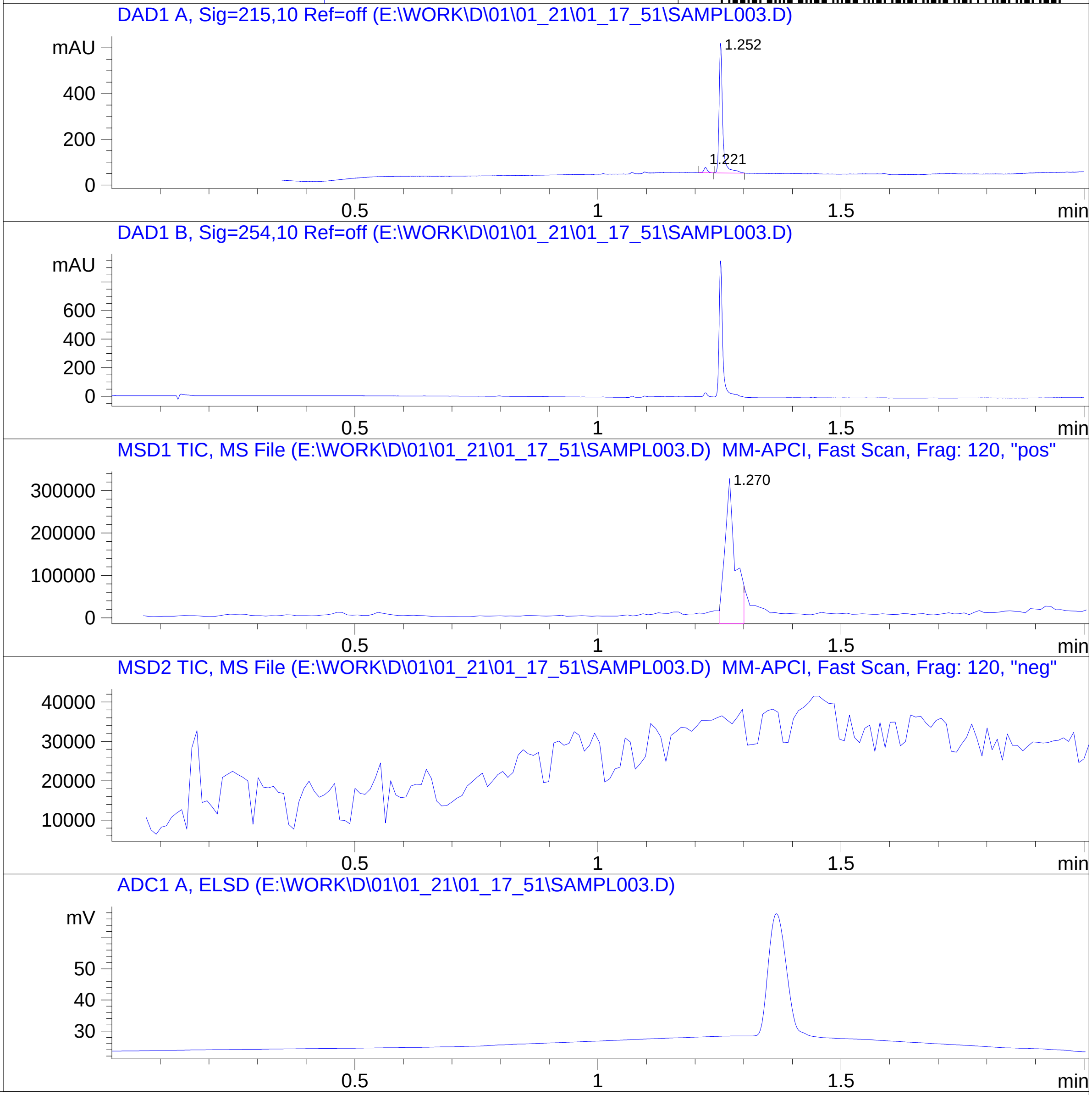

RT 1.270

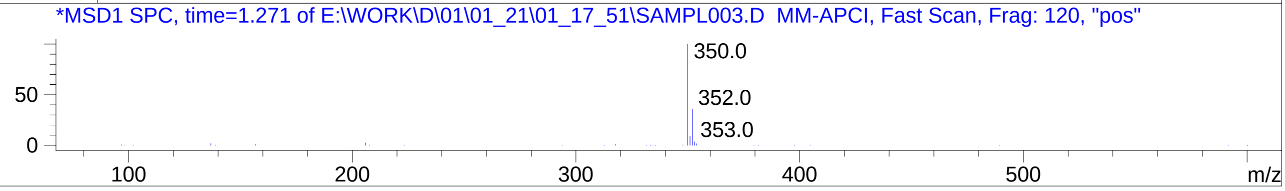

Inj.Date 1/20/2013 K P2-A-03 RR Acq. Method C:\CHEM32\-> ->
