# Supplementary material for: AI is a viable alternative to high throughput screening: a 318-target study
Source: Sci Rep. 2024 Apr 2;14:7526. doi: 10.1038/s41598-024-54655-z (PMC10987645; doi:10.1038/s41598-024-54655-z)

MaxPeak: 100.00%
Ret_Time: 1.620 min

T6382080

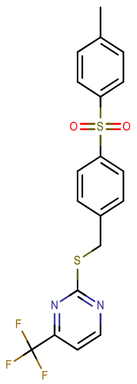

Mol Wt 424.46

Exact Mass 424.07

#	Time	Area%
1	1.620	100.00

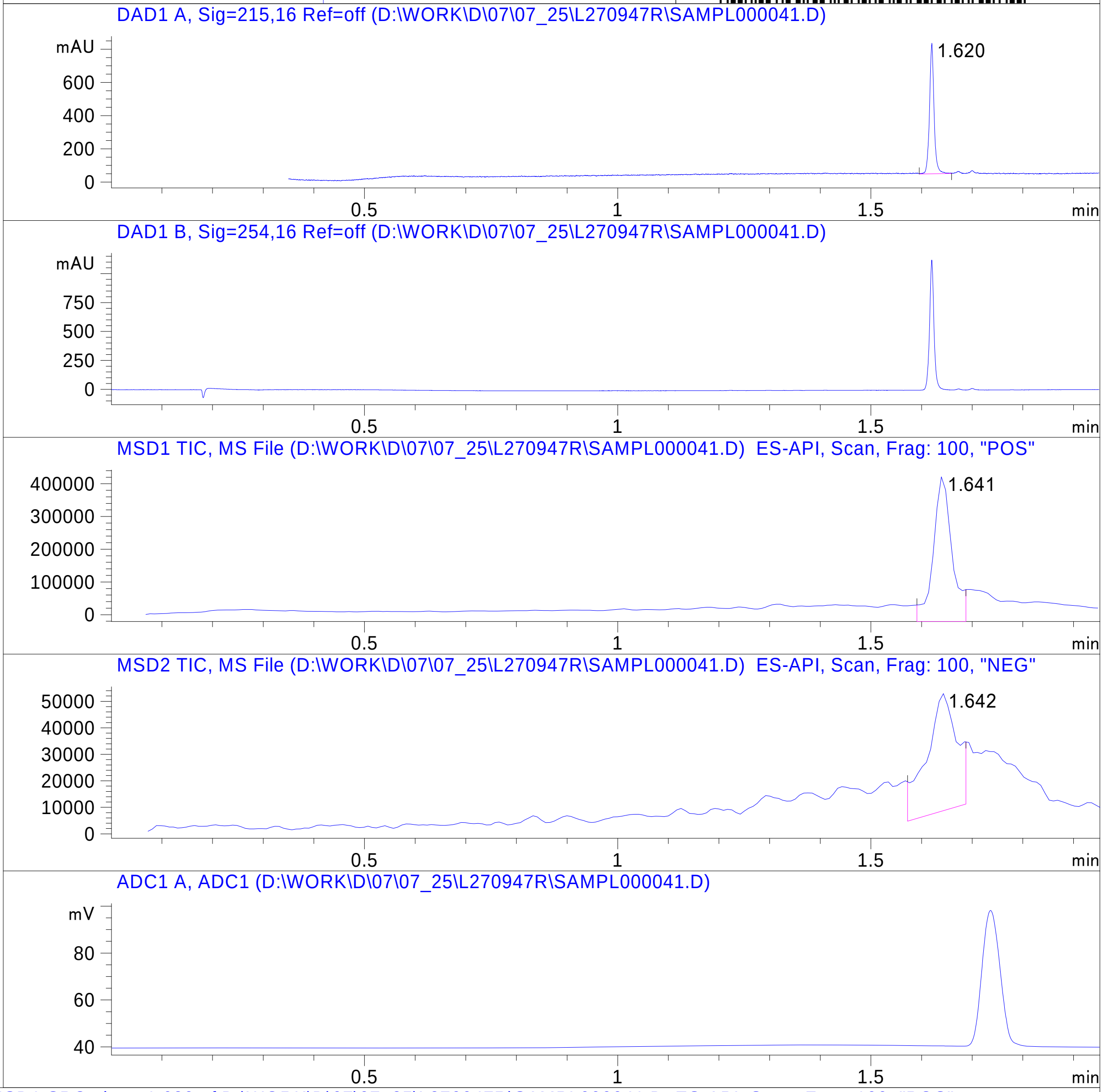

RT 1.641

RT 1.642

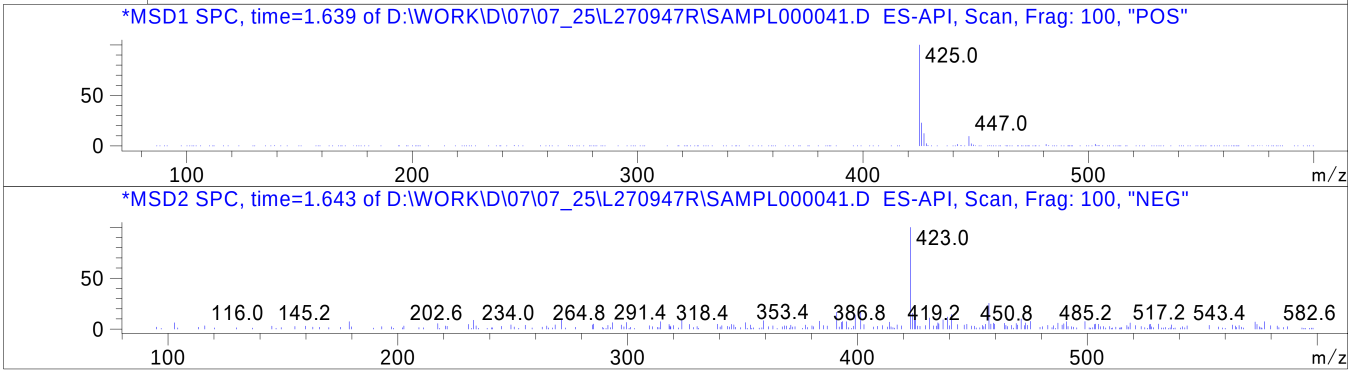

Supplement: Supplementary file 1 — Supplementary Information 1. [file 41598_2024_54655_MOESM1_ESM.zip › Nature SREP/QC_AIMS_files/Proj235.pdf]
