# Supplementary material for: AI is a viable alternative to high throughput screening: a 318-target study
Source: Sci Rep. 2024 Apr 2;14:7526. doi: 10.1038/s41598-024-54655-z (PMC10987645; doi:10.1038/s41598-024-54655-z)
Supplement: Supplementary file 1 — Supplementary Information 1. [file 41598_2024_54655_MOESM1_ESM.zip › Nature SREP/QC_AIMS_files/Proj239.pdf]

MaxPeak: 95.67%
Ret_Time: 0.752 min

5302953

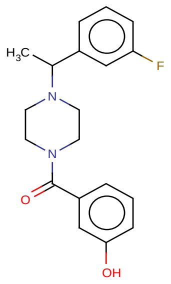

Mol Wt 328.381
Exact Mass 328.19

#	Time	Area%
1	0.628	2.87
2	0.752	95.67
3	1.052	1.47

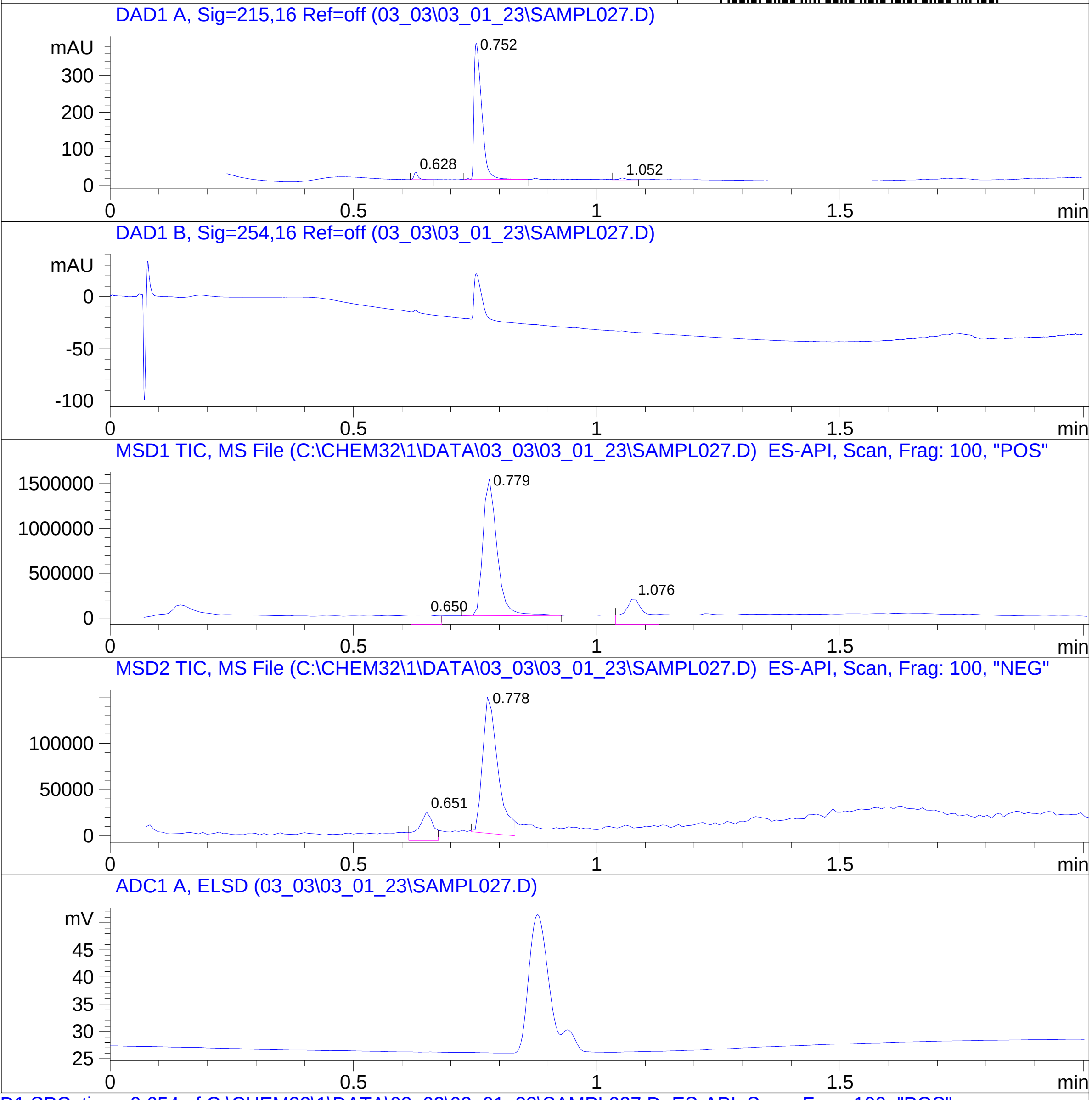

RT 0.650

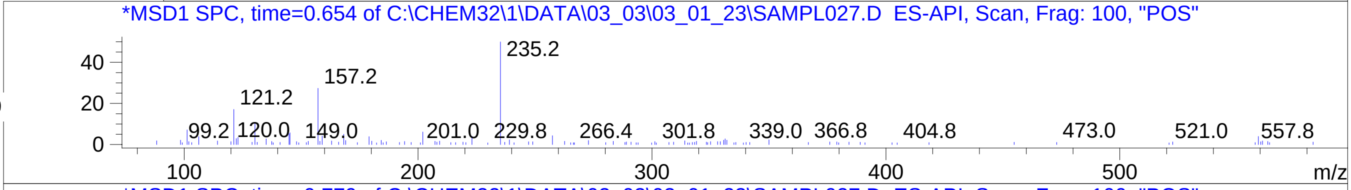

RT 0.779

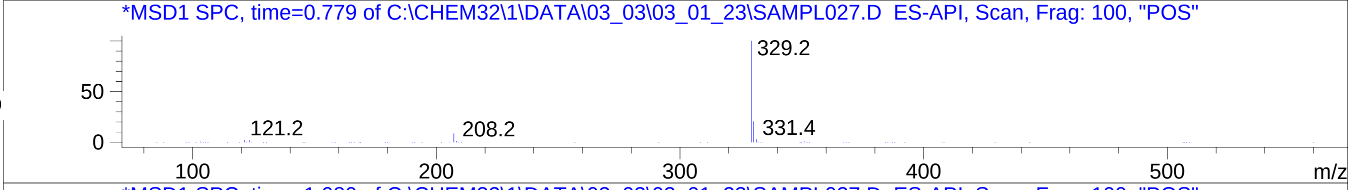

RT 1.076

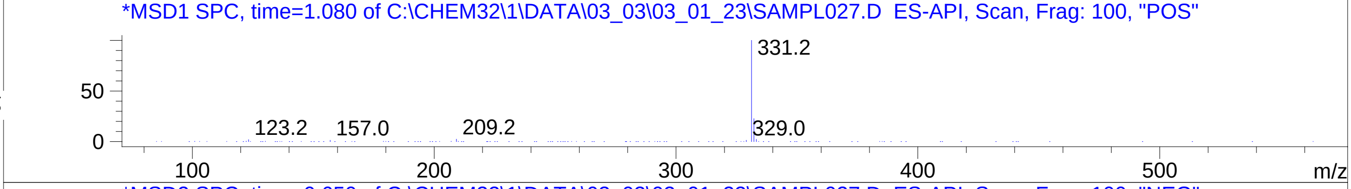

RT 0.651

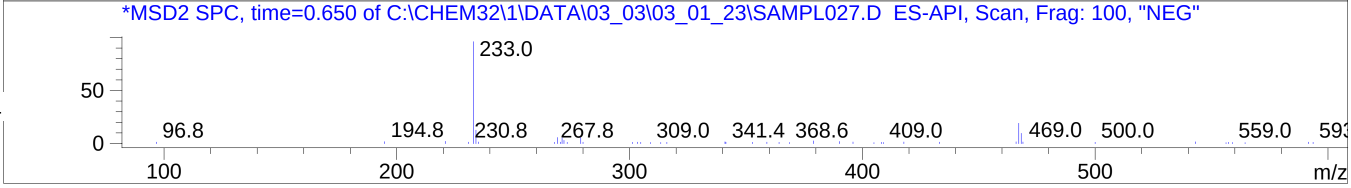

Inj.Date 3/3/2012 N P2-C-09 '4' Acq. Method C:\CHEM32\-> ->

RT 0.778

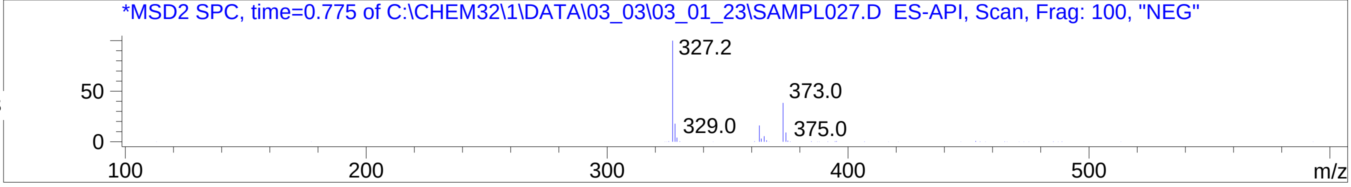
*MSD2 SPC, time=0.775 of C:\CHEM32\1\DATA\03_03\03_01_23\SAMPL027.D ES-API, Scan, Frag: 100, "NEG"
327.2
329.0
373.0
375.0
50
0
100
200
300
400
500
m/z
