# Supplementary figures and images for: AI is a viable alternative to high throughput screening: a 318-target study
Source: Sci Rep. 2024 Apr 2;14:7526. doi: 10.1038/s41598-024-54655-z (PMC10987645; doi:10.1038/s41598-024-54655-z)

Y662653\$5

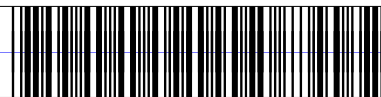

MaxPeak: 97.27%  
Ret\_Time: 0.606 min

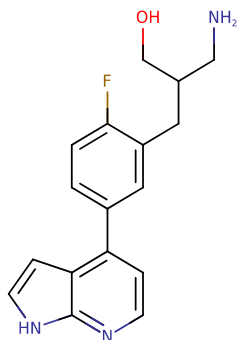

Mol Wt 299.34  
Exact Mass 299.17

| # | Time  | Area% |
|---|-------|-------|
| 1 | 0.606 | 97.27 |
| 2 | 0.759 | 2.30  |
| 3 | 1.356 | 0.43  |

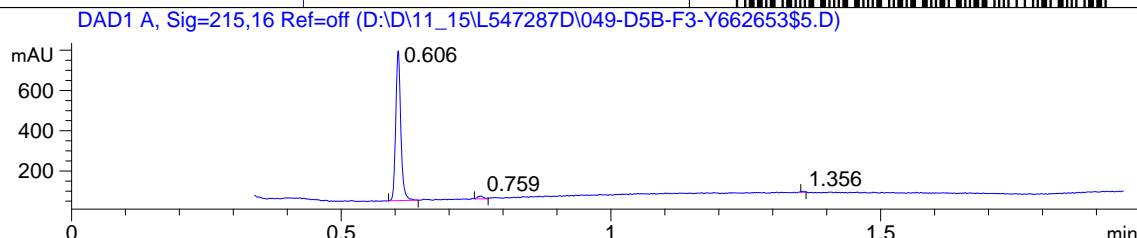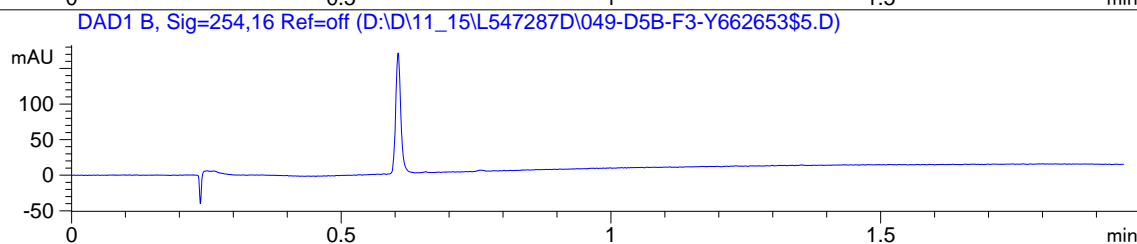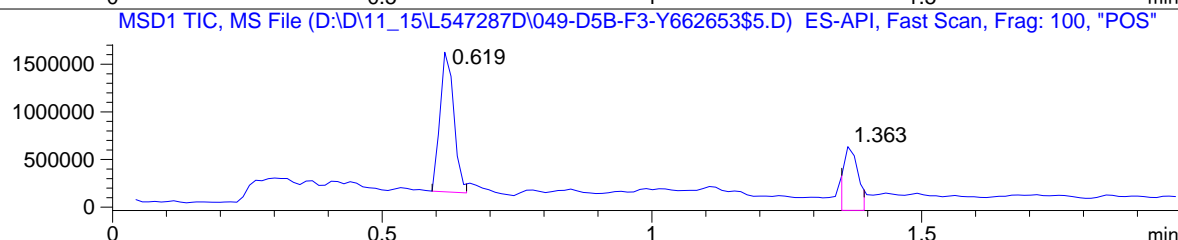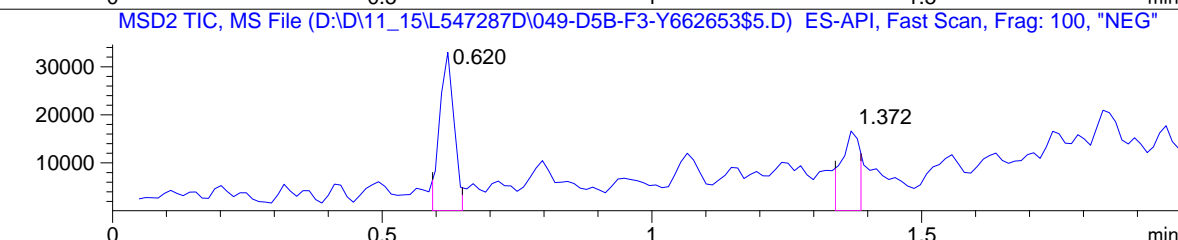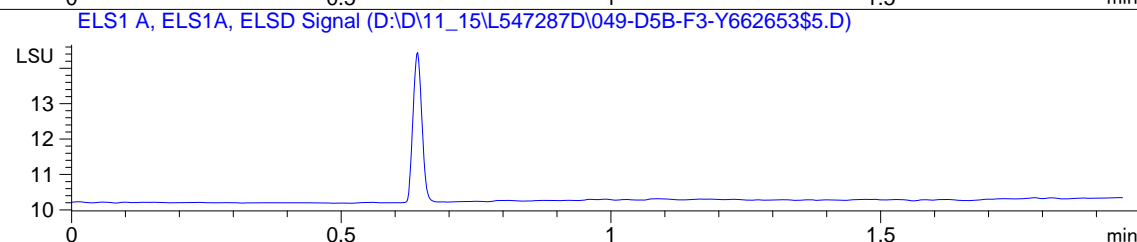

RT 0.619

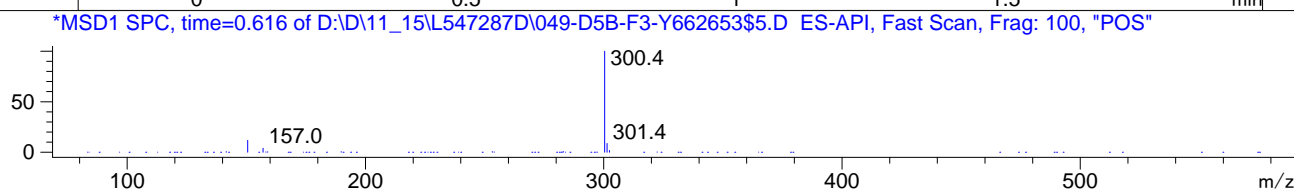

RT 1.363

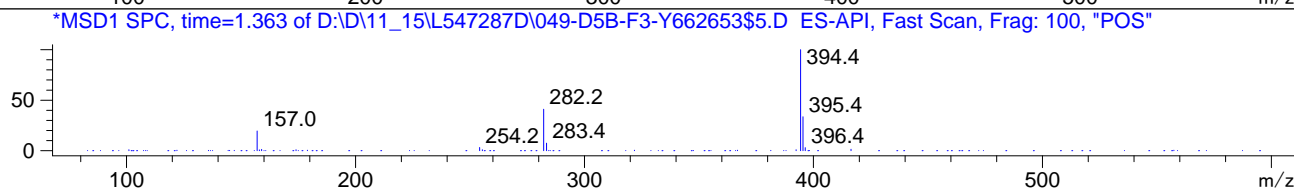

RT 0.620

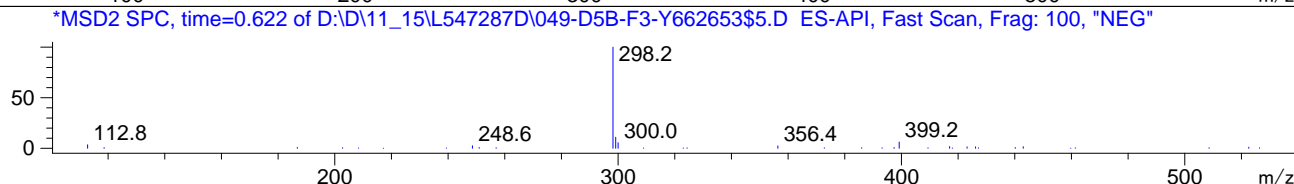

RT 1.372

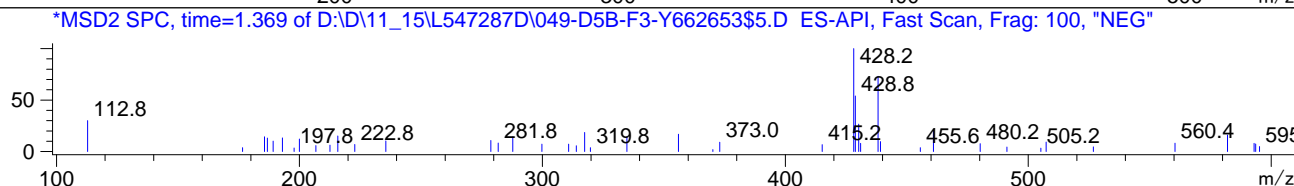

Supplement: Supplementary file 1 — Supplementary Information 1. [file 41598_2024_54655_MOESM1_ESM.zip › Nature SREP/QC_AIDD_cs_selected/LATS1_HID_10_LCMS.pdf]

MaxPeak: 99.40%  
Ret\_Time: 0.780 min

U268030\$11

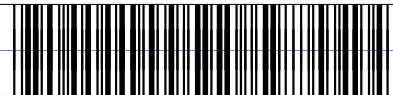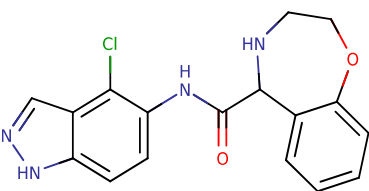

Mol Wt 342.78  
Exact Mass 342.1

| # | Time  | Area% |
|---|-------|-------|
| 1 | 0.780 | 99.40 |
| 2 | 1.266 | 0.60  |

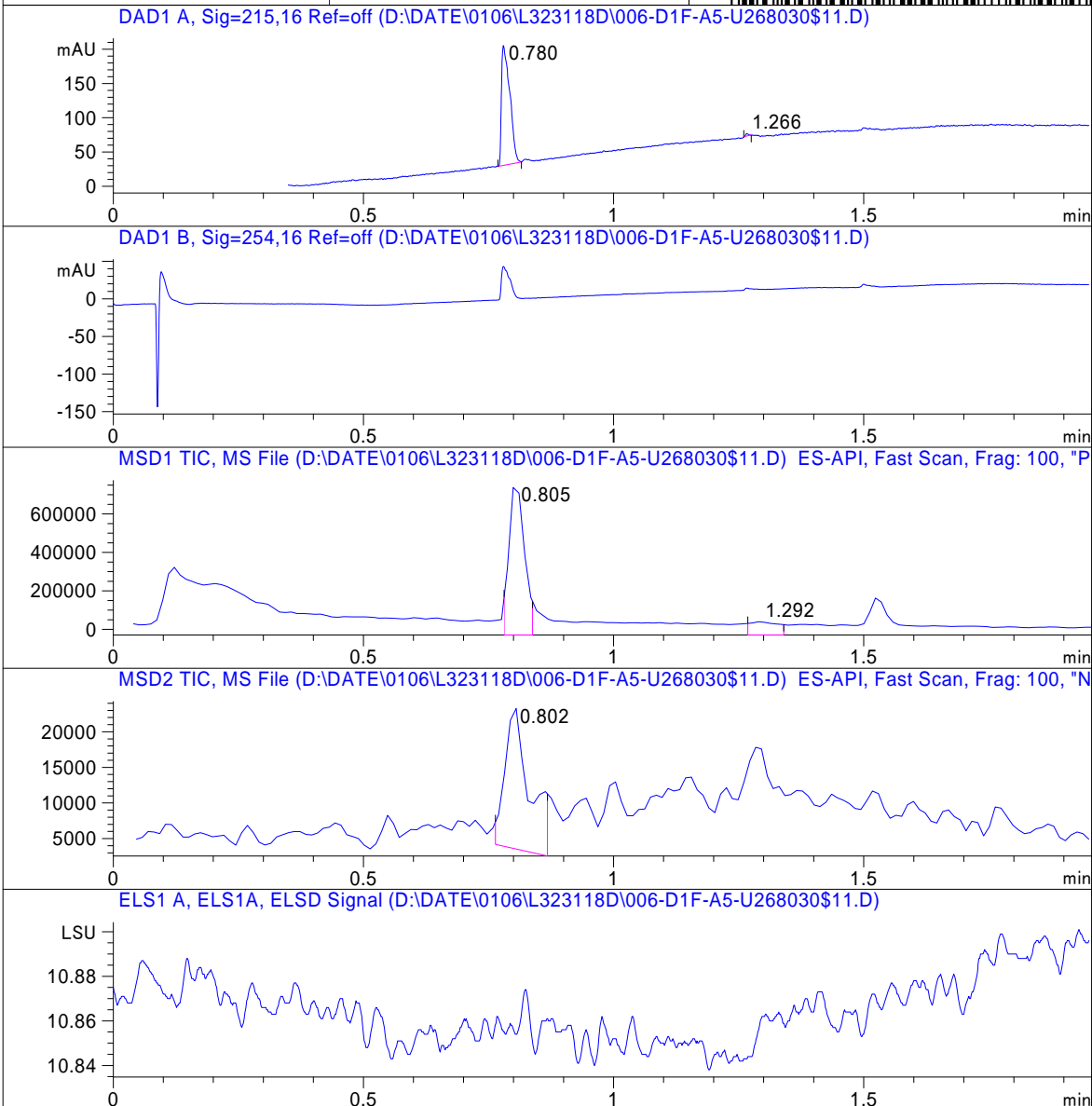

RT 0.805

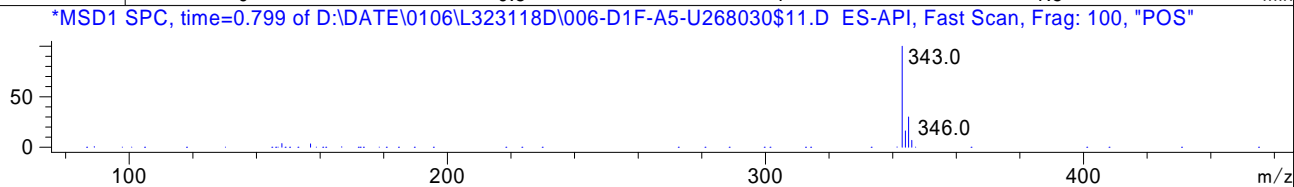

RT 1.292

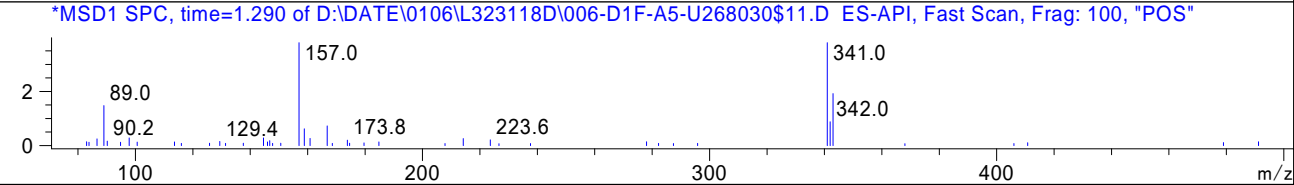

RT 0.802

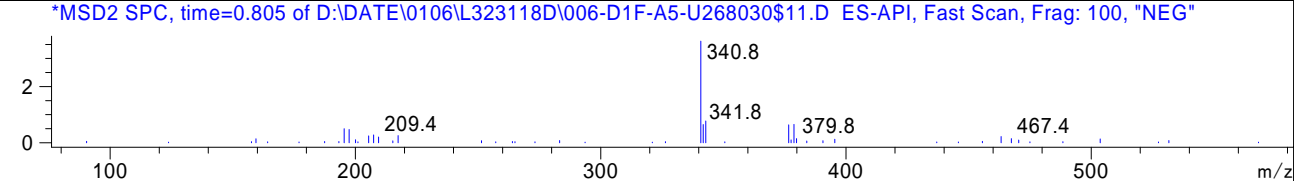

Supplement: Supplementary file 1 — Supplementary Information 1. [file 41598_2024_54655_MOESM1_ESM.zip › Nature SREP/QC_AIDD_cs_selected/LATS1_HID_1_LCMS.pdf]

U753246\$21

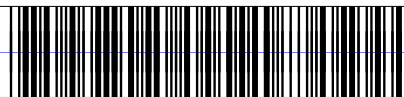

MaxPeak: 92.21%  
Ret\_Time: 0.760 min

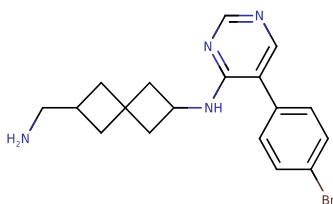

Mol Wt 373.29  
Exact Mass 372.13

| # | Time  | Area% |
|---|-------|-------|
| 1 | 0.760 | 92.21 |
| 2 | 1.005 | 2.75  |
| 3 | 1.046 | 3.09  |
| 4 | 1.125 | 1.95  |

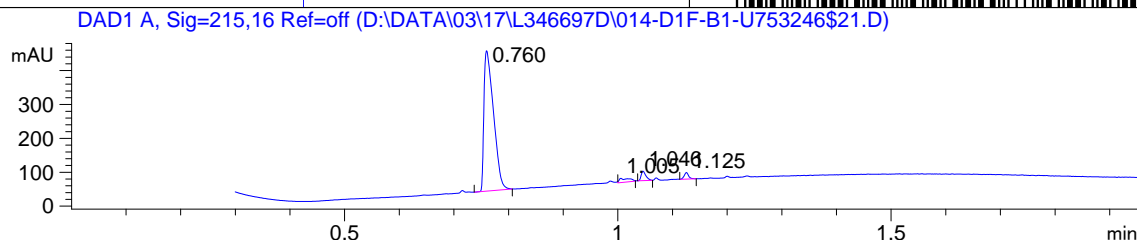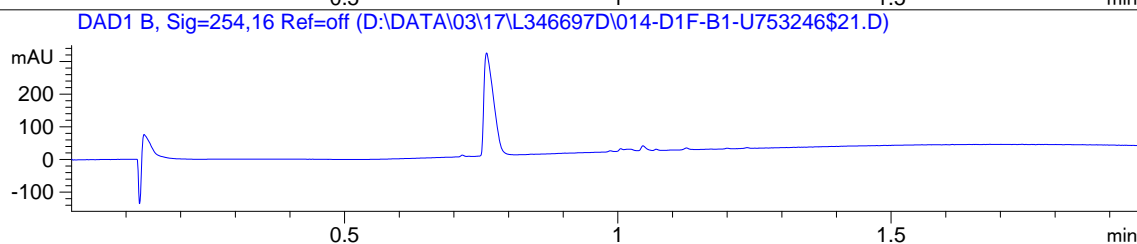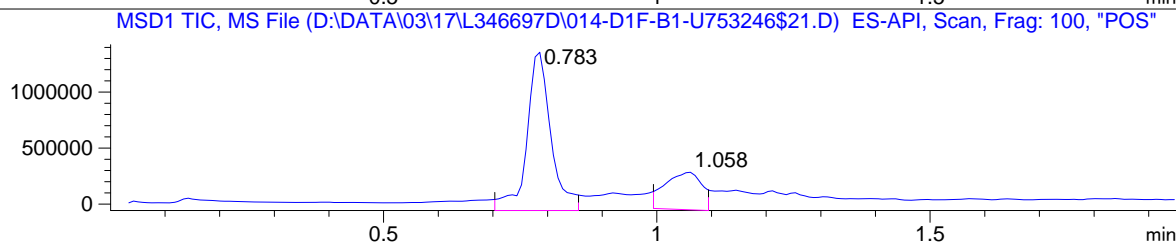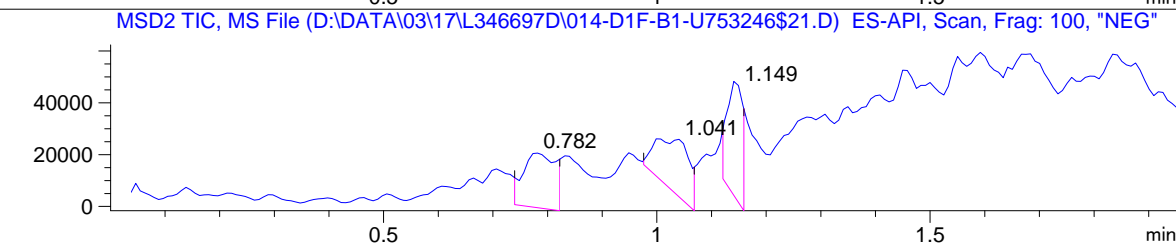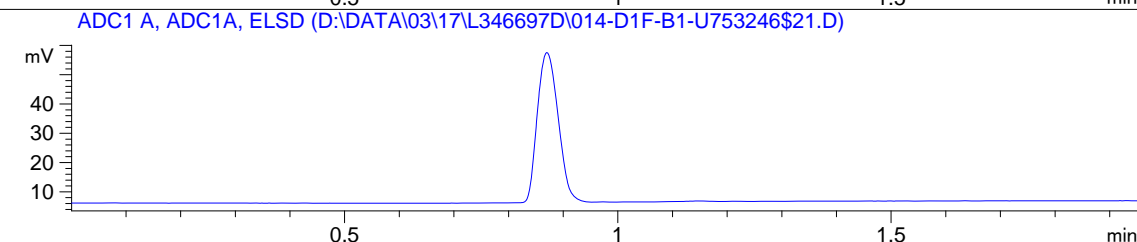

RT 0.783

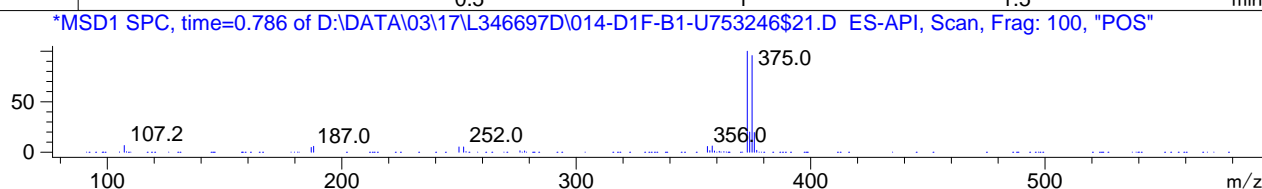

RT 1.058

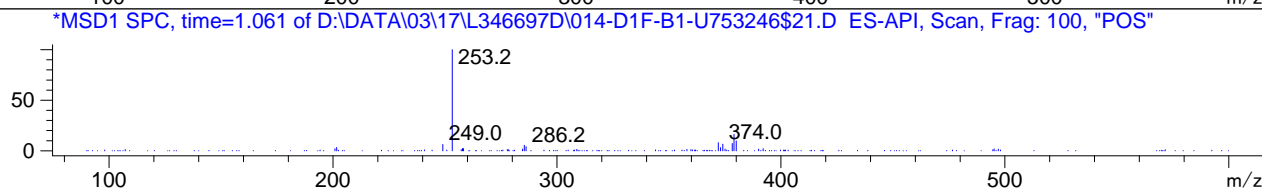

RT 0.782

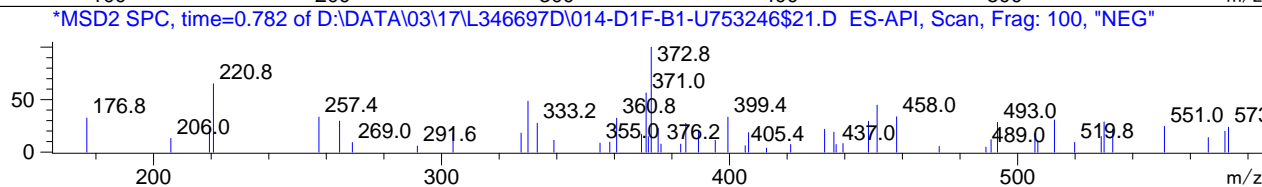

RT 1.041

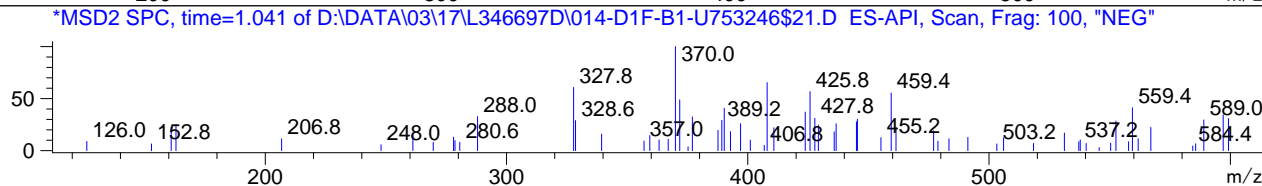

RT 1.149

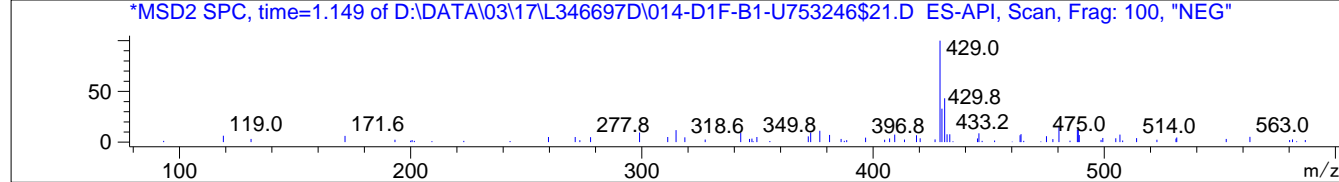

Supplement: Supplementary file 1 — Supplementary Information 1. [file 41598_2024_54655_MOESM1_ESM.zip › Nature SREP/QC_AIDD_cs_selected/LATS1_HID_3_LCMS.pdf]

W538597\$2

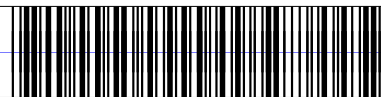

MaxPeak: 91.49%  
Ret\_Time: 0.731 min

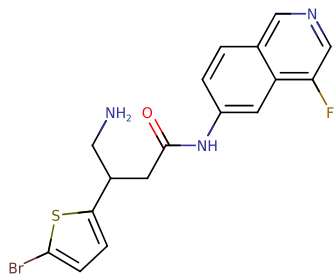

Mol Wt 408.29  
Exact Mass 409.03

| # | Time  | Area% |
|---|-------|-------|
| 1 | 0.623 | 1.85  |
| 2 | 0.731 | 91.49 |
| 3 | 0.819 | 4.82  |
| 4 | 0.913 | 1.83  |

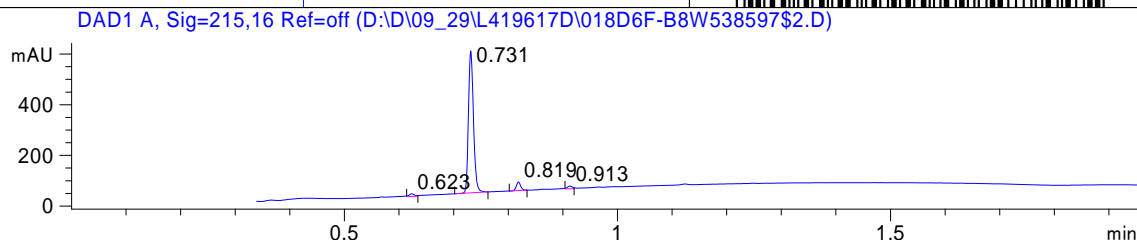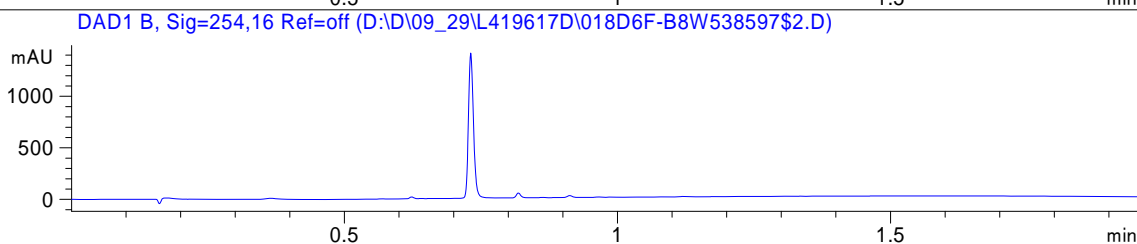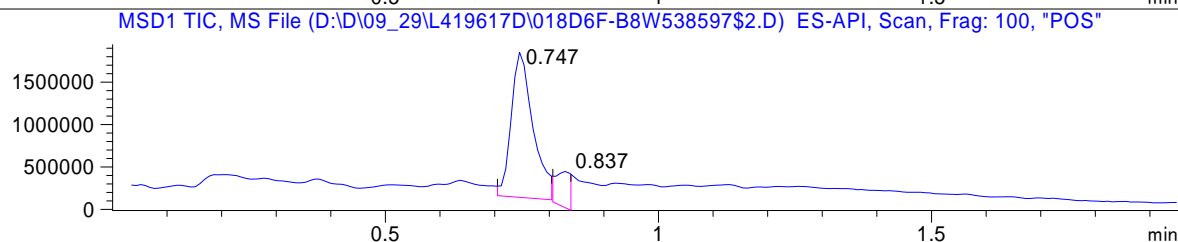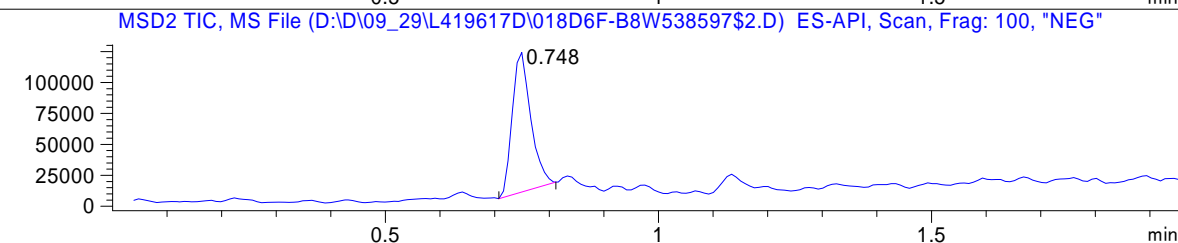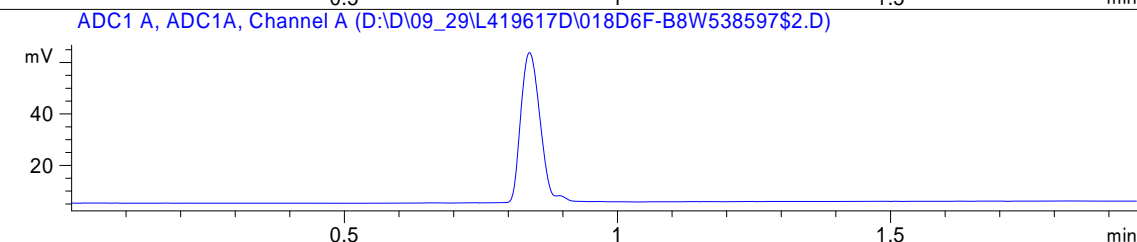

RT 0.747

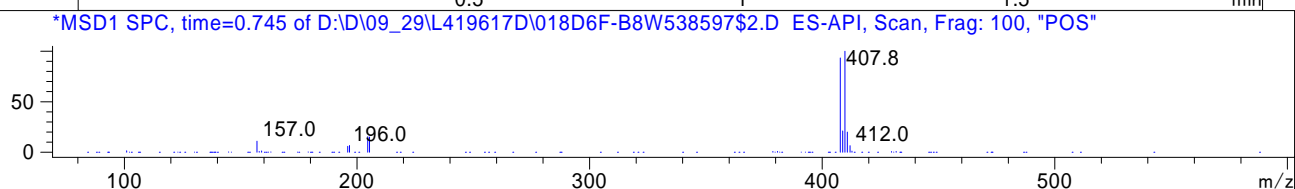

RT 0.837

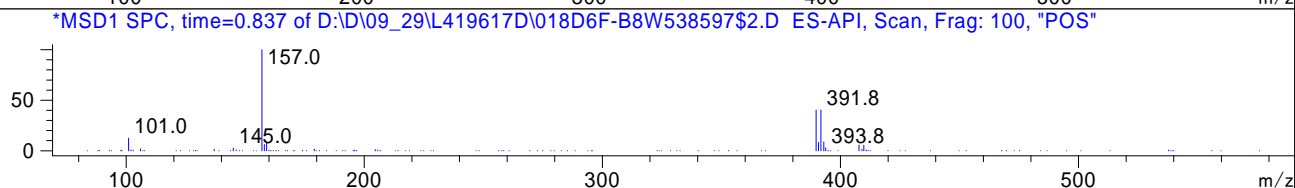

RT 0.748

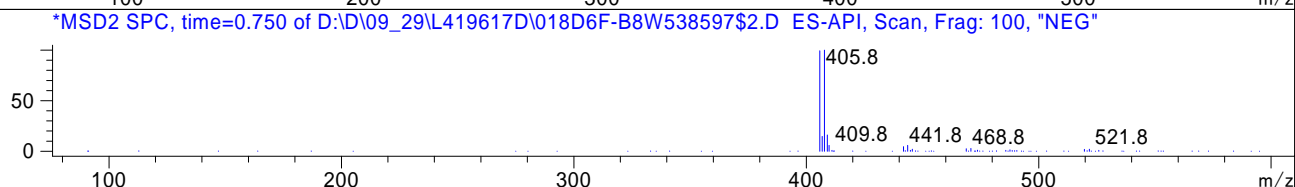

Supplement: Supplementary file 1 — Supplementary Information 1. [file 41598_2024_54655_MOESM1_ESM.zip › Nature SREP/QC_AIDD_cs_selected/LATS1_HVE_1_LCMS.pdf]

W538420\$2

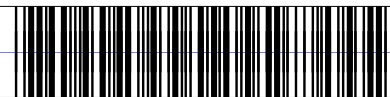

MaxPeak: 95.03%  
Ret\_Time: 0.981 min

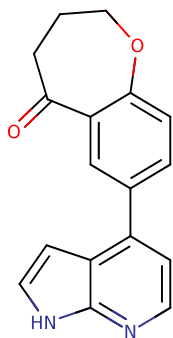

Mol Wt 278.3  
Exact Mass 278.12

| # | Time  | Area% |
|---|-------|-------|
| 1 | 0.962 | 1.77  |
| 2 | 0.981 | 95.03 |
| 3 | 1.287 | 3.20  |

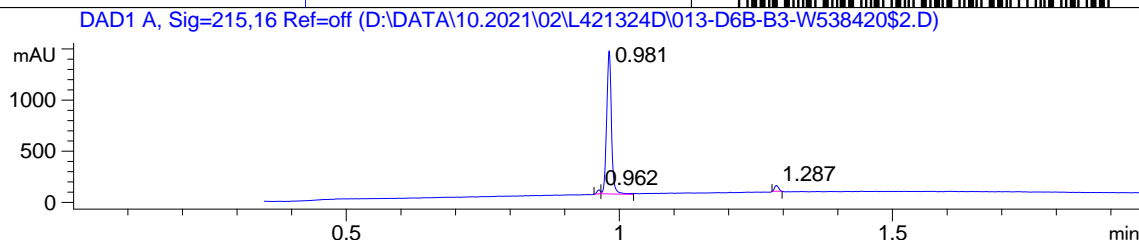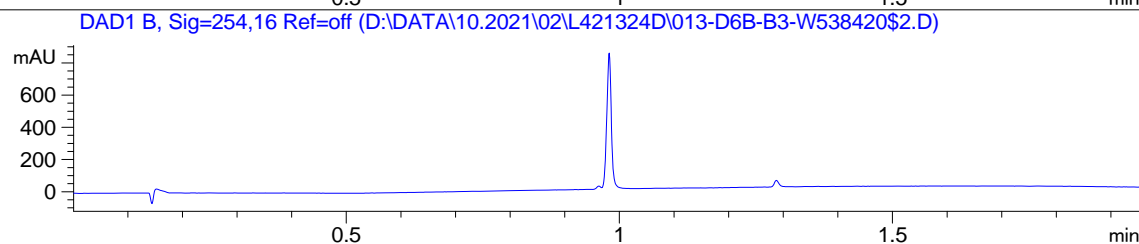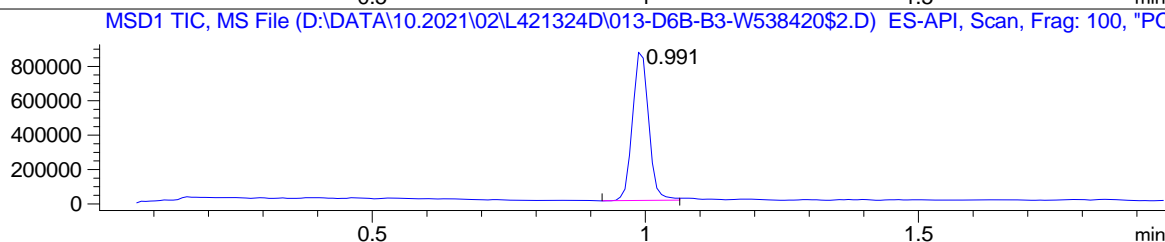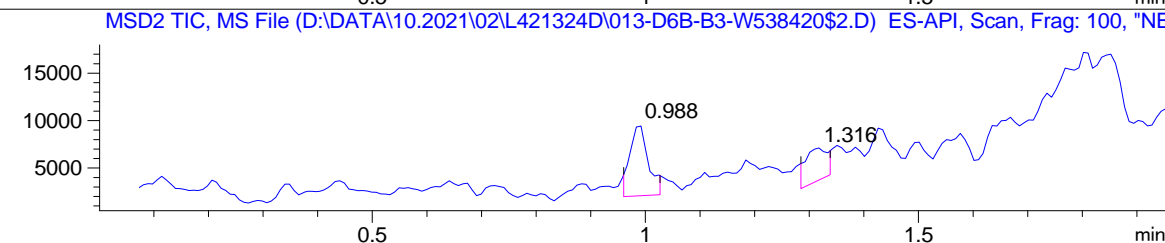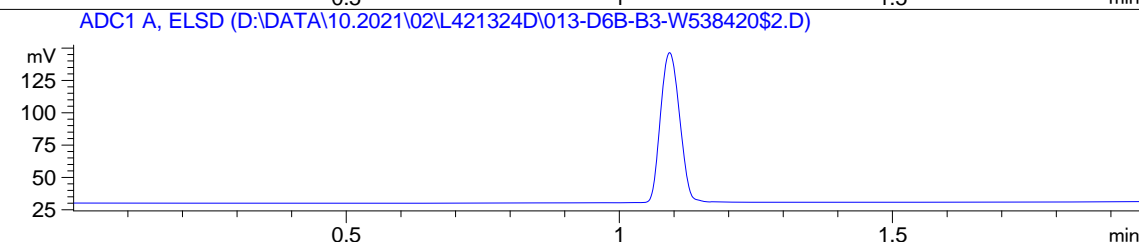

RT 0.991

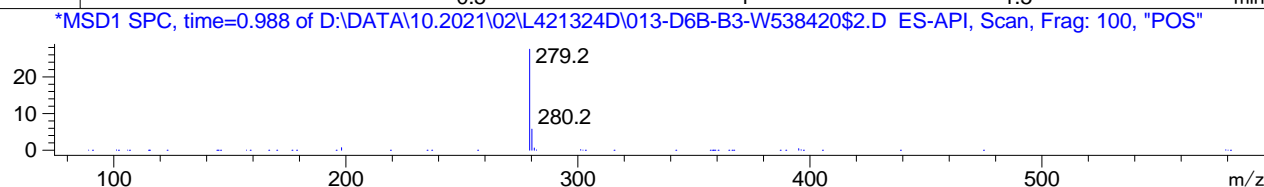

RT 0.988

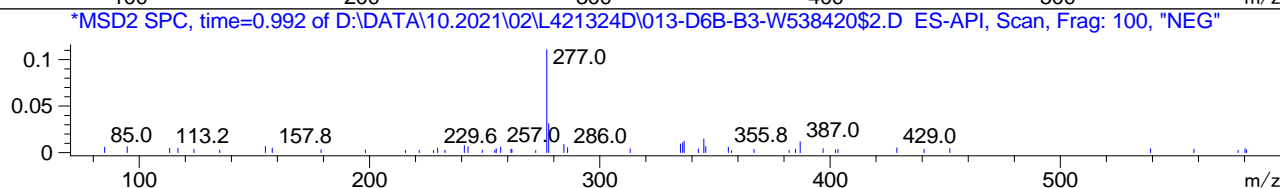

RT 1.316

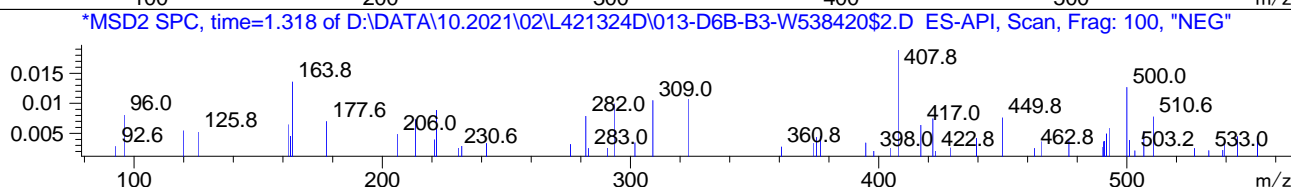

Supplement: Supplementary file 1 — Supplementary Information 1. [file 41598_2024_54655_MOESM1_ESM.zip › Nature SREP/QC_AIDD_cs_selected/LATS1_HVE_9_LCMS.pdf]

MaxPeak: 97.87%  
Ret\_Time: 0.795 min

U753256\$2

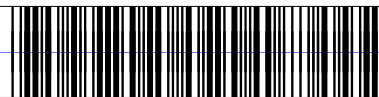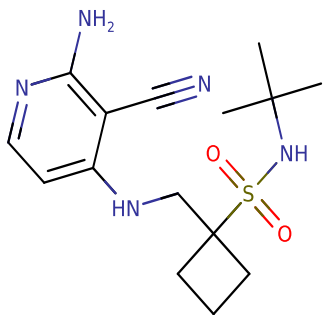

Mol Wt 337.44  
Exact Mass 337.18

| # | Time  | Area% |
|---|-------|-------|
| 1 | 0.795 | 97.87 |
| 2 | 1.126 | 2.13  |

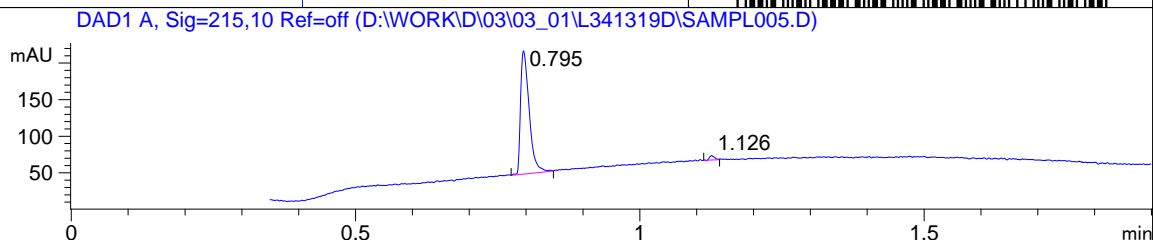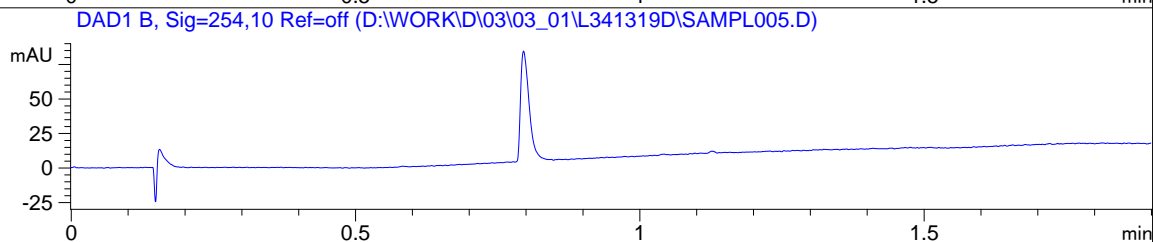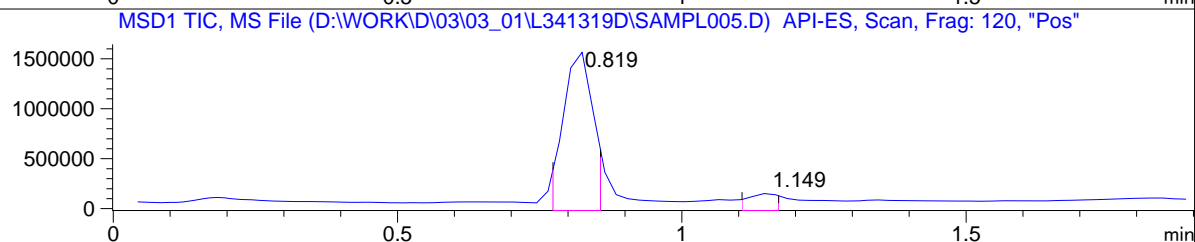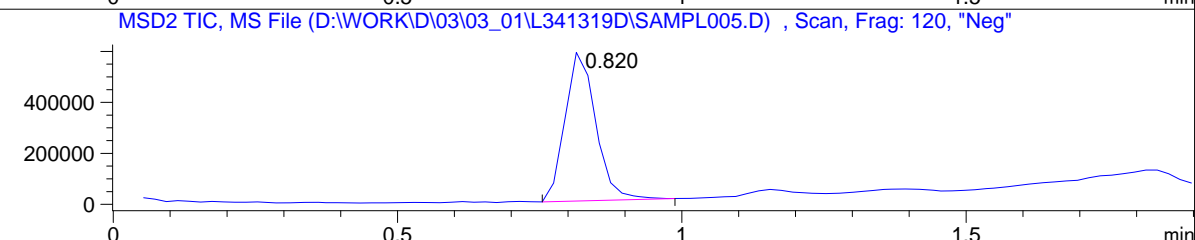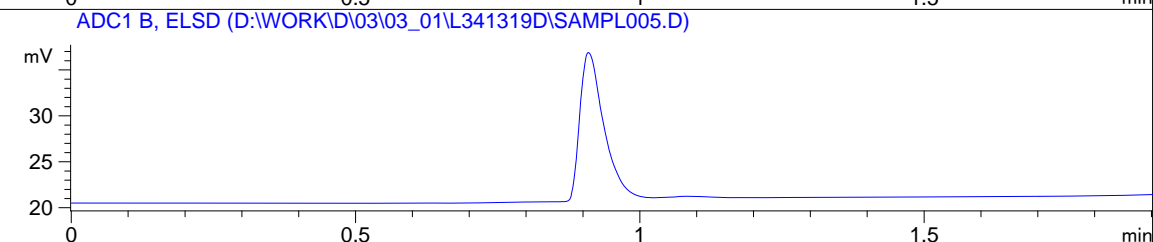

RT 0.819

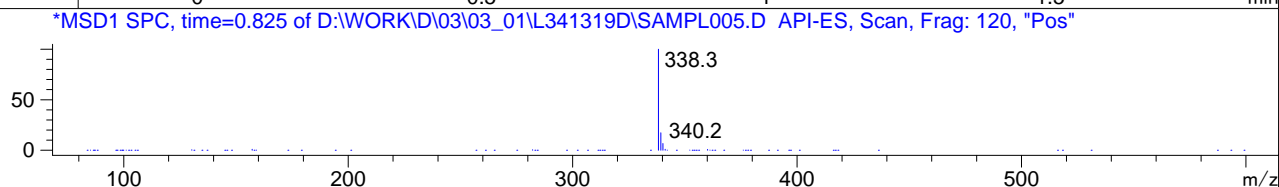

RT 1.149

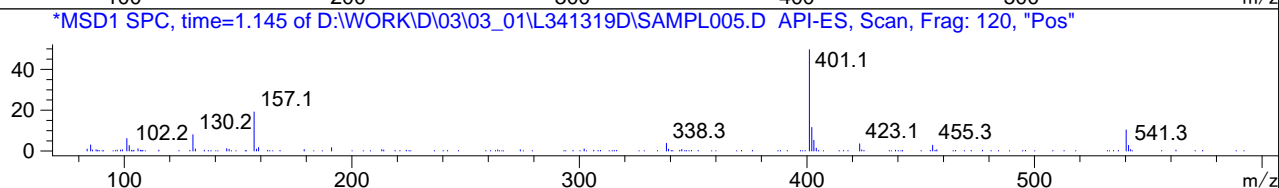

RT 0.820

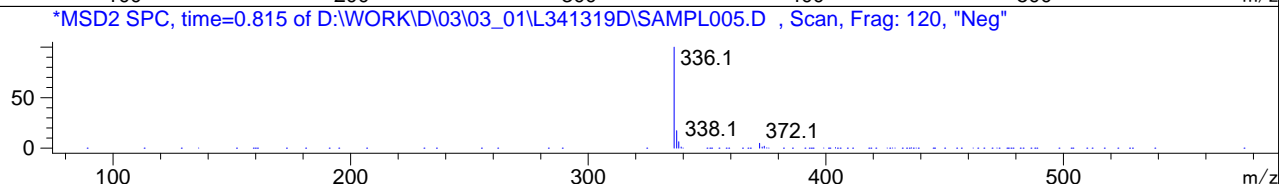

Supplement: Supplementary file 1 — Supplementary Information 1. [file 41598_2024_54655_MOESM1_ESM.zip › Nature SREP/QC_AIDD_cs_selected/LATS1_HVE_PARENT_4_LCMS.pdf]

V026485\$1

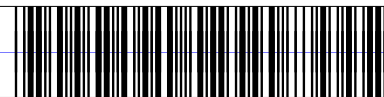

MaxPeak: 100.00%  
Ret\_Time: 1.462 min

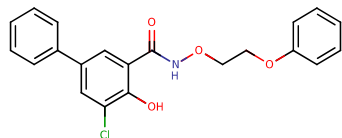

Mol Wt 383.82  
Exact Mass 383.11

| # | Time  | Area%  |
|---|-------|--------|
| 1 | 1.462 | 100.00 |

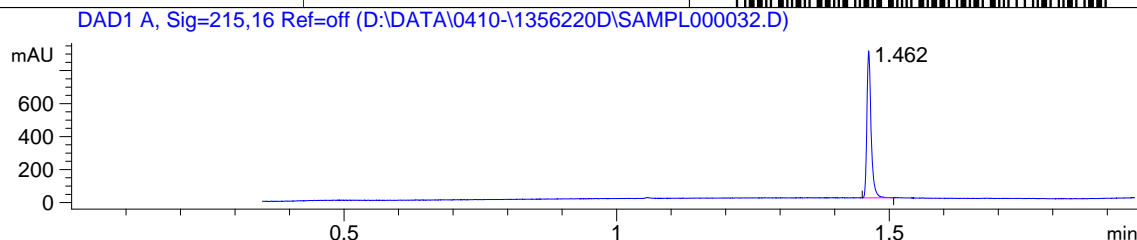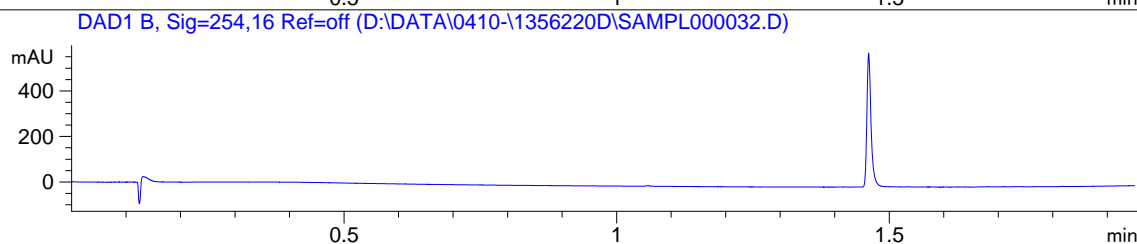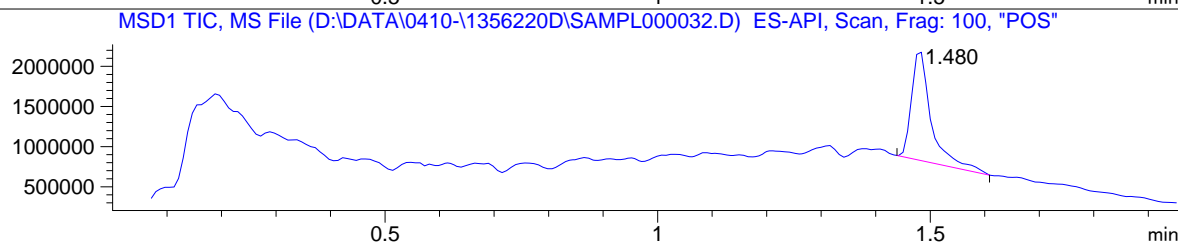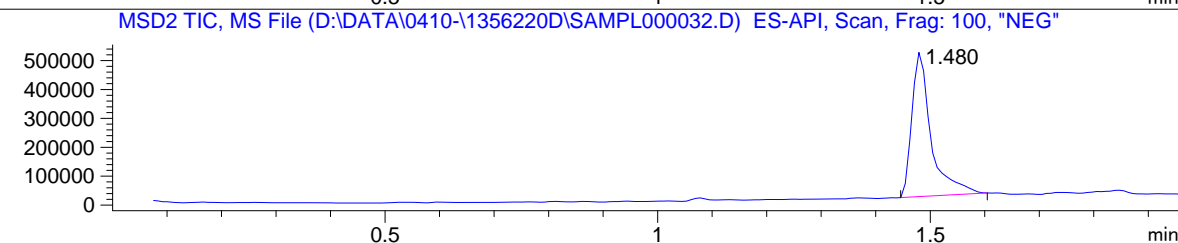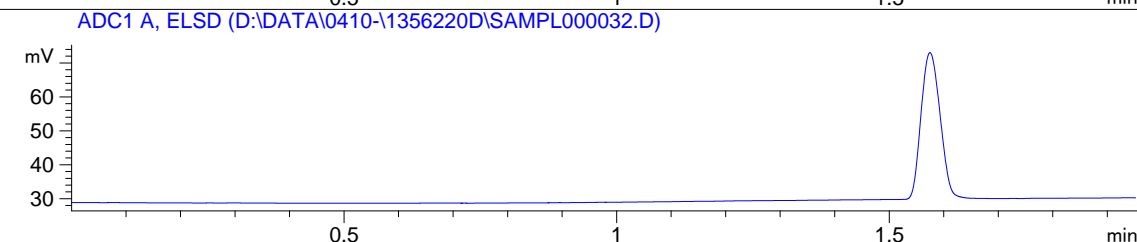

RT 1.480

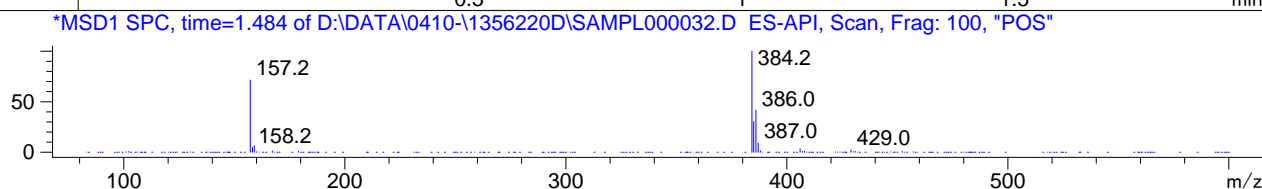

RT 1.480

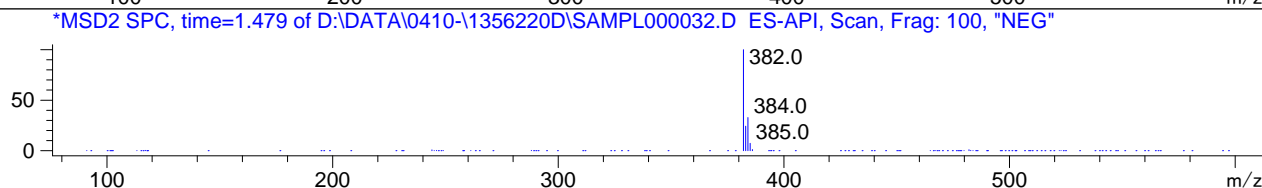

Supplement: Supplementary file 1 — Supplementary Information 1. [file 41598_2024_54655_MOESM1_ESM.zip › Nature SREP/QC_AIDD_cs_selected/VCP_HID_6_LCMS.pdf]

U035962\$3

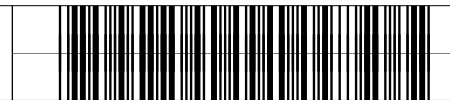

MaxPeak: 98.52%  
Ret\_Time: 0.881 min

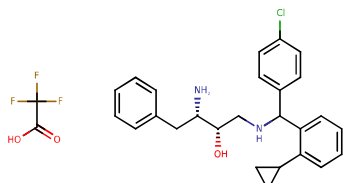

Mol Wt 535  
Exact Mass 420.25

| # | Time  | Area% |
|---|-------|-------|
| 1 | 0.881 | 98.52 |
| 2 | 1.190 | 1.48  |

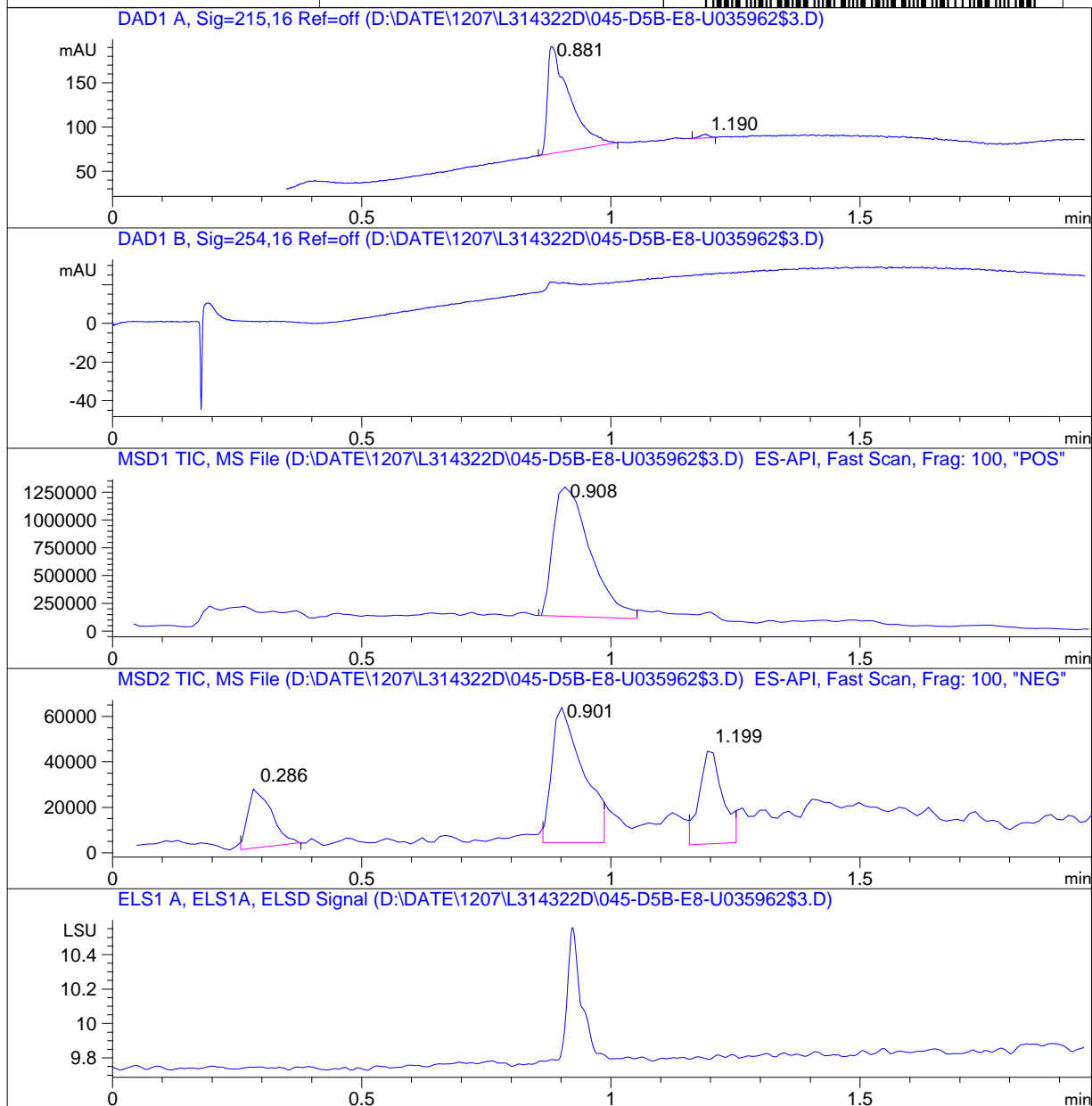

RT 0.908

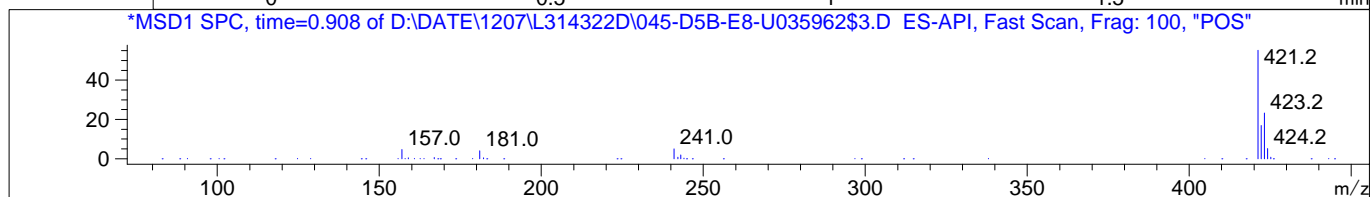

RT 0.286

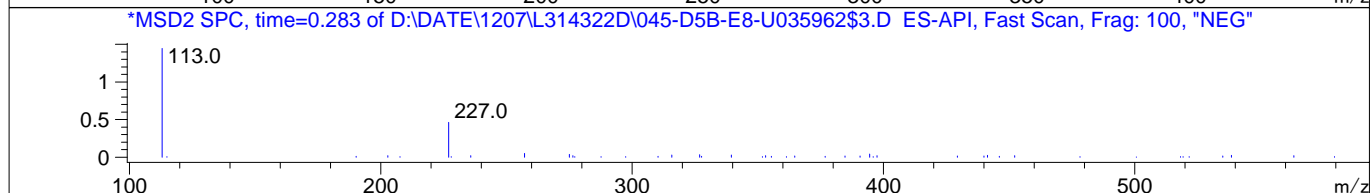

RT 0.901

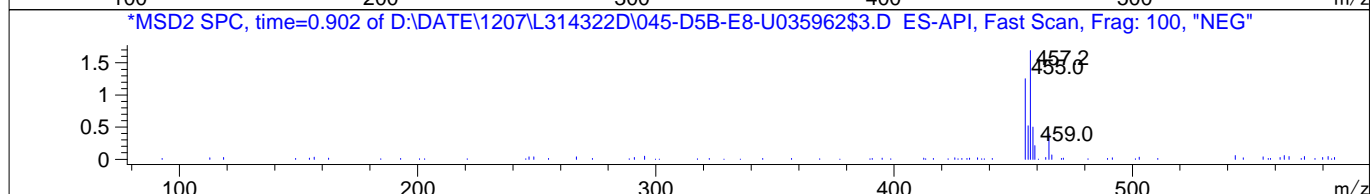

RT 1.199

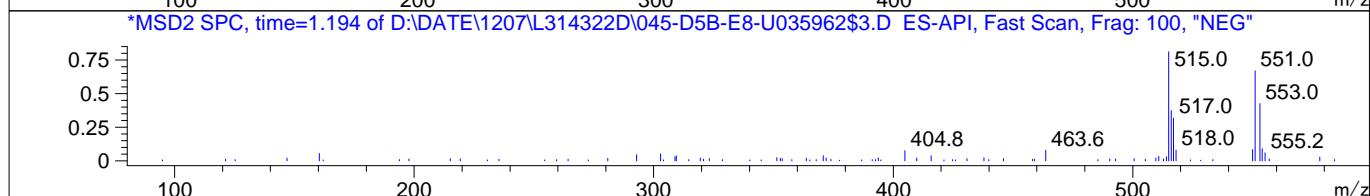

Supplement: Supplementary file 1 — Supplementary Information 1. [file 41598_2024_54655_MOESM1_ESM.zip › Nature SREP/QC_AIDD_selected/MC2R_DR_exemplar_LCMS.pdf]

MaxPeak: 100.00%  
Ret\_Time: 1.266 min

U110721\$1

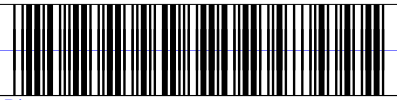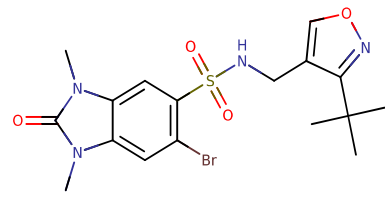

Mol Wt 457.34  
Exact Mass 458.06

| # | Time  | Area%  |
|---|-------|--------|
| 1 | 1.266 | 100.00 |

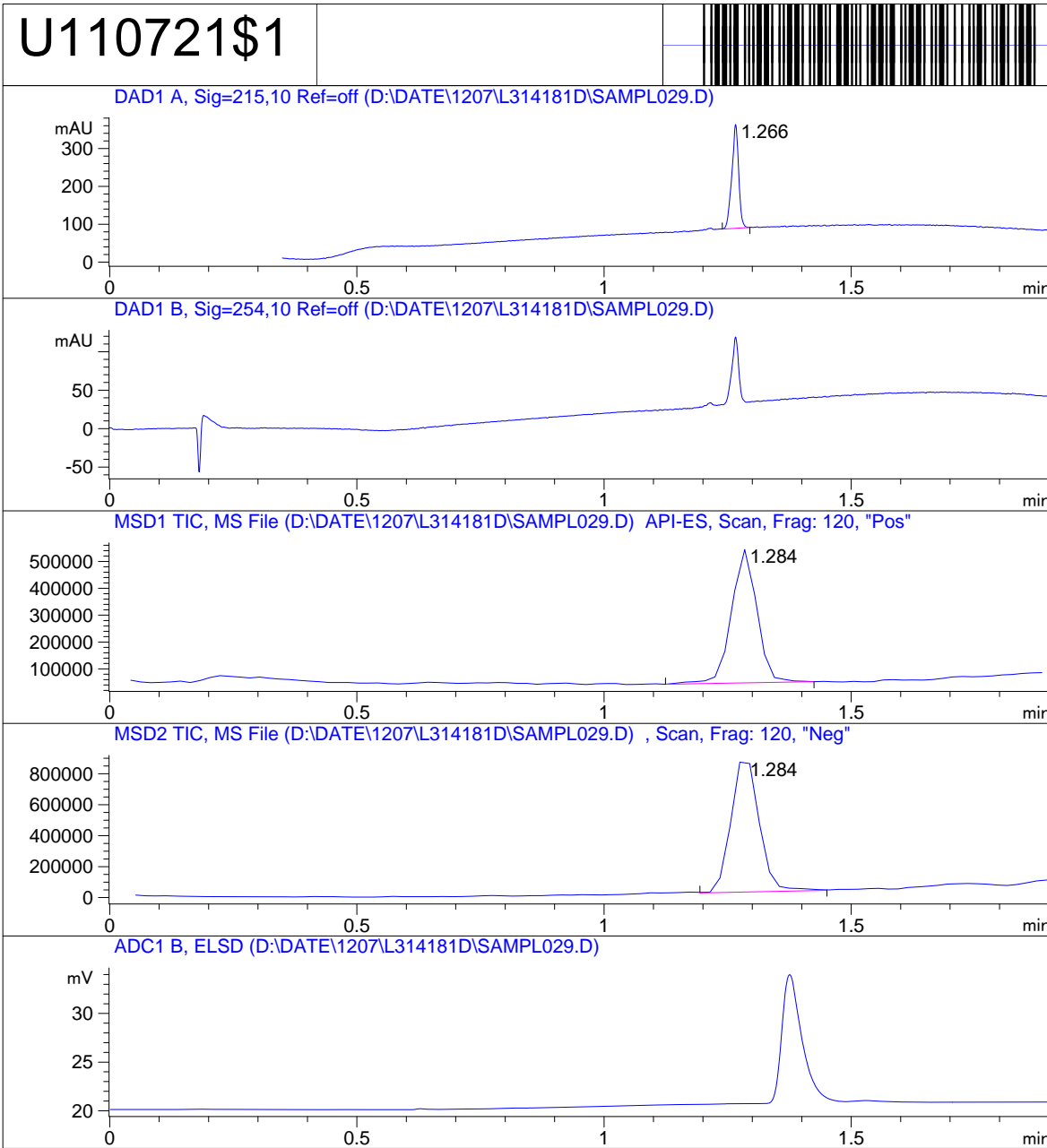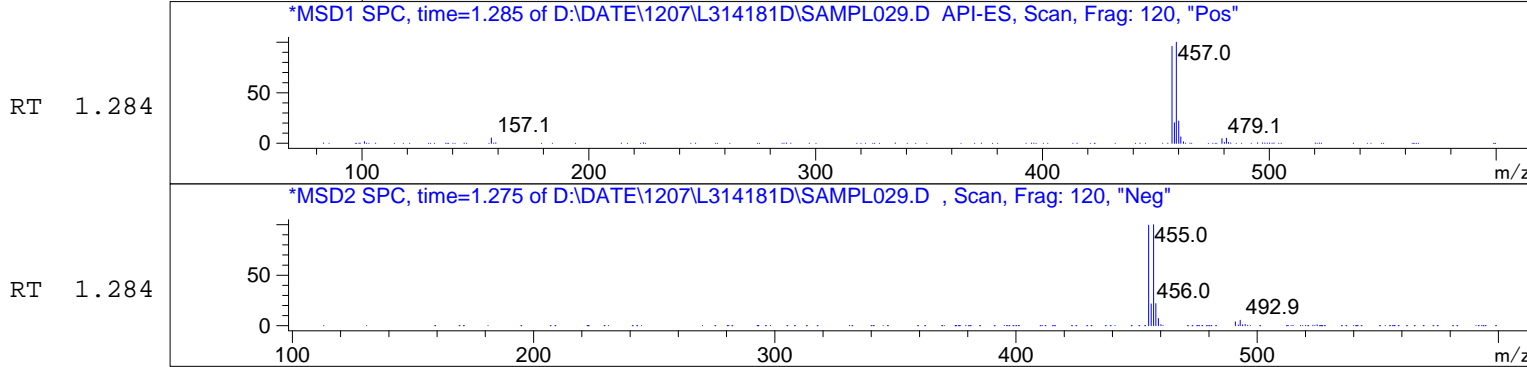

Supplement: Supplementary file 1 — Supplementary Information 1. [file 41598_2024_54655_MOESM1_ESM.zip › Nature SREP/QC_AIDD_selected/NT5E_DR_exemplar_LCMS.pdf]

W647390\$J

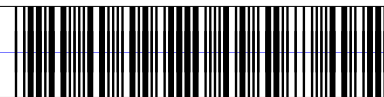

MaxPeak: 94.02%  
Ret\_Time: 0.900 min

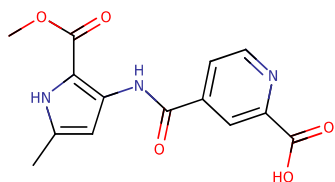

Mol Wt 303.27  
Exact Mass 303.08

| # | Time  | Area% |
|---|-------|-------|
| 1 | 0.900 | 94.02 |
| 2 | 0.982 | 2.70  |
| 3 | 1.057 | 1.39  |
| 4 | 1.298 | 1.89  |

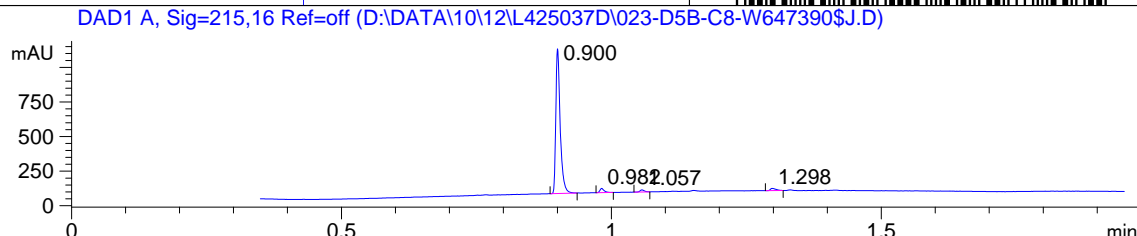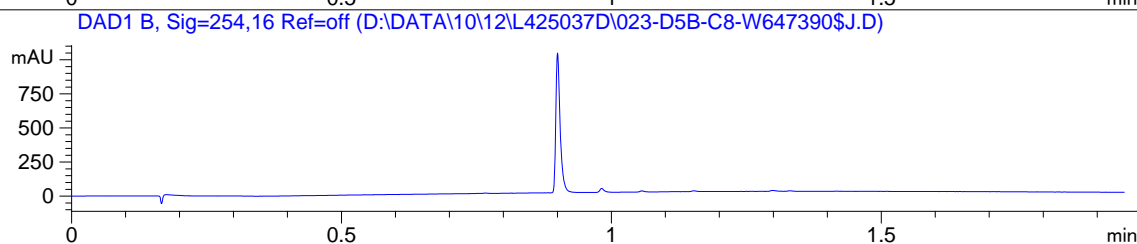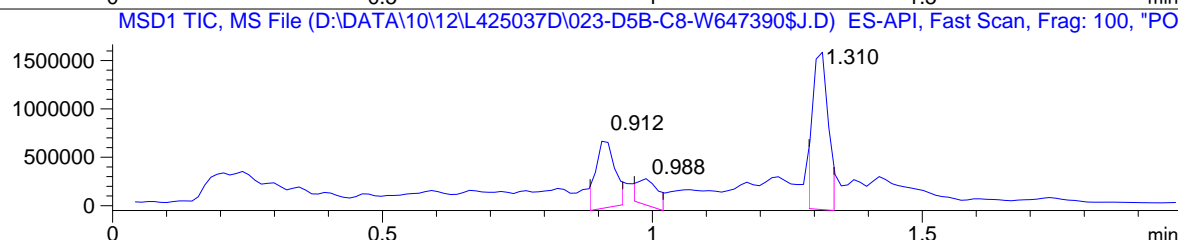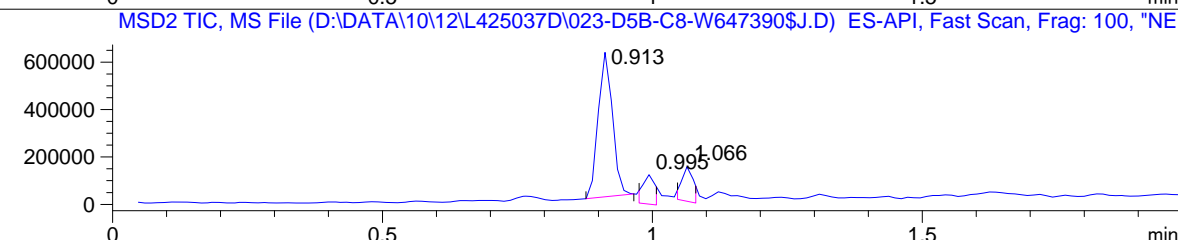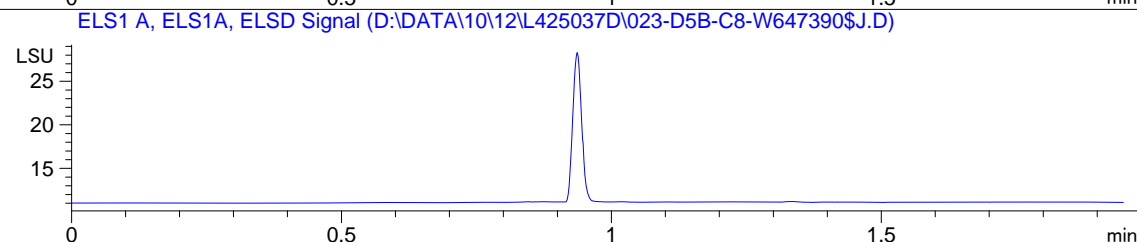

RT 0.912

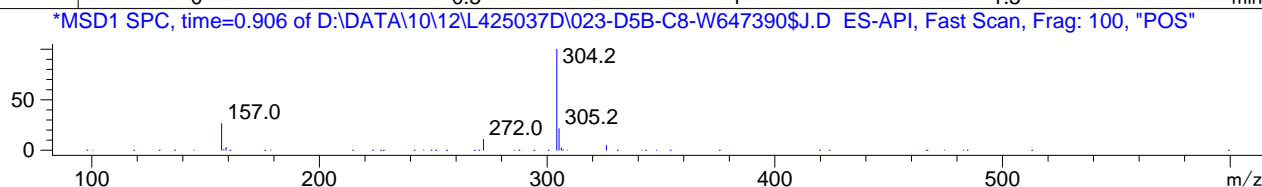

RT 0.988

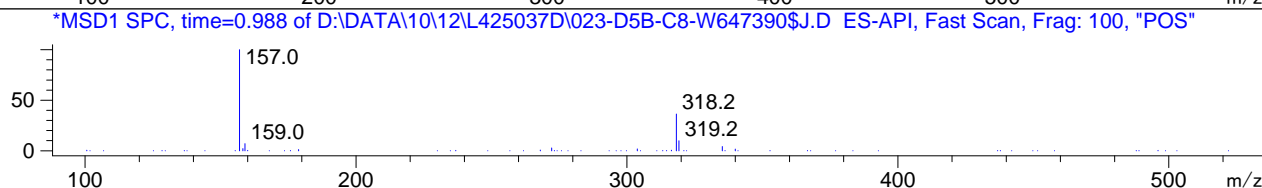

RT 1.310

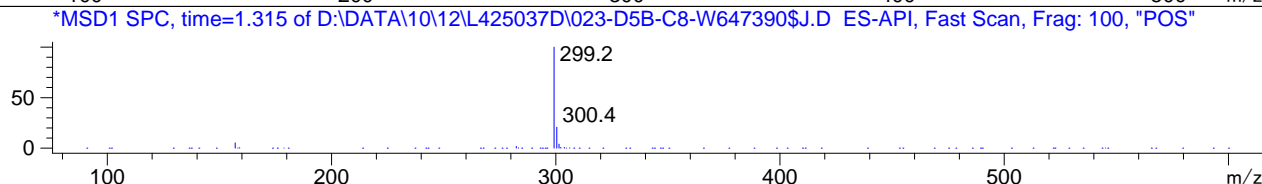

RT 0.913

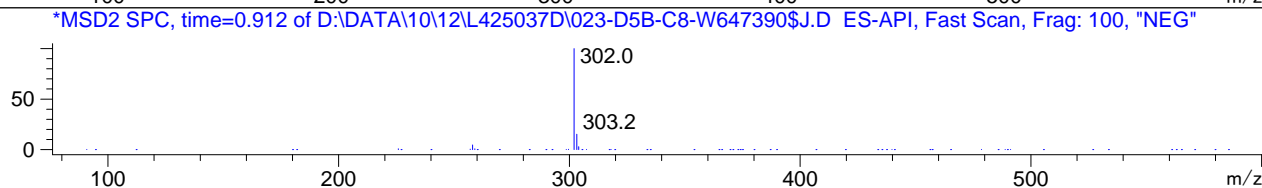

RT 0.995

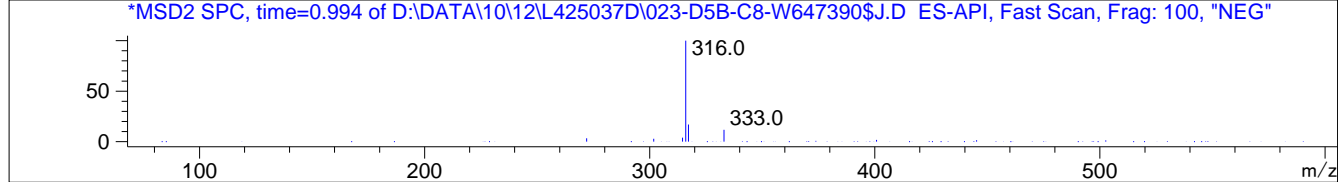

RT 1.066

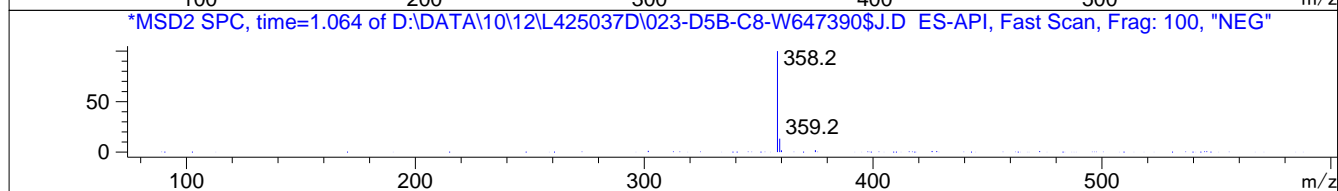

Supplement: Supplementary file 1 — Supplementary Information 1. [file 41598_2024_54655_MOESM1_ESM.zip › Nature SREP/QC_AIDD_selected/PARP14_DR_exemplar_LCMS.pdf]

U172020\$1

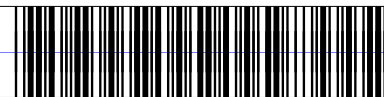

MaxPeak: 100.00%  
Ret\_Time: 0.492 min

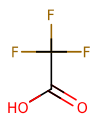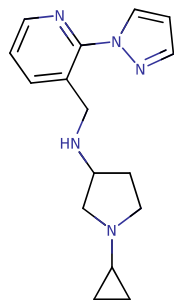

Mol Wt 397.4  
Exact Mass 283.21

| # | Time  | Area%  |
|---|-------|--------|
| 1 | 0.492 | 100.00 |

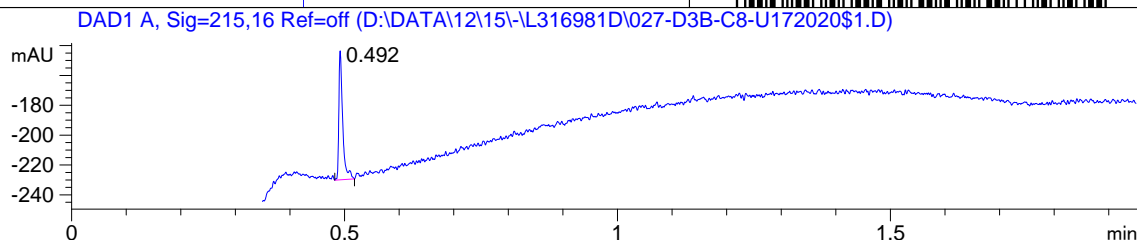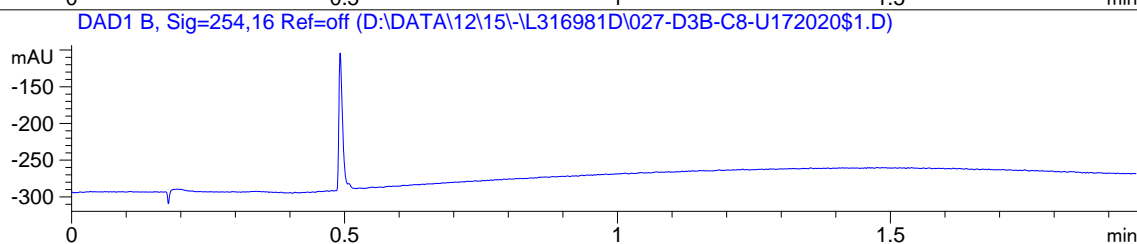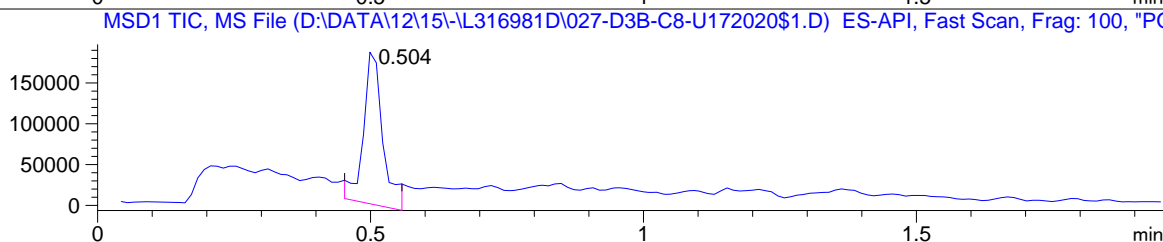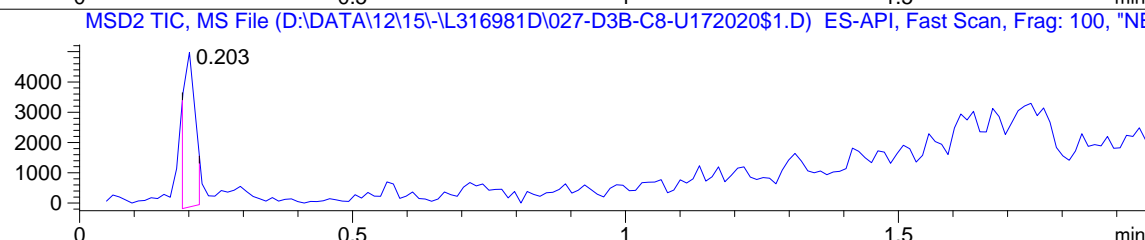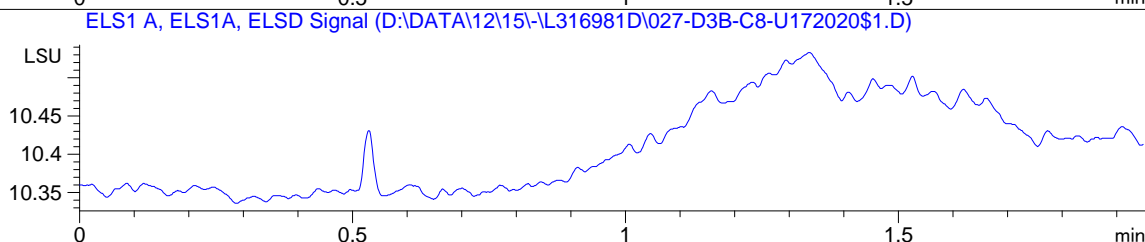

RT 0.504

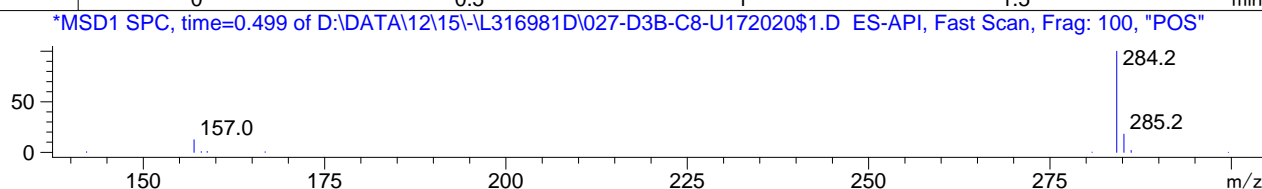

RT 0.203

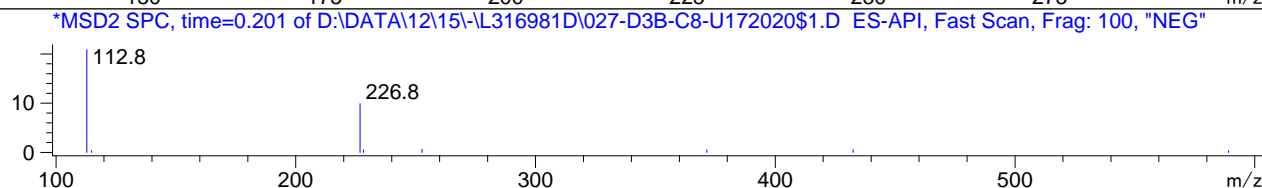

Supplement: Supplementary file 1 — Supplementary Information 1. [file 41598_2024_54655_MOESM1_ESM.zip › Nature SREP/QC_AIDD_selected/PRMT5_DR_exemplar_LCMS.pdf]

MaxPeak: 97.40%  
Ret\_Time: 1.566 min

T5147966

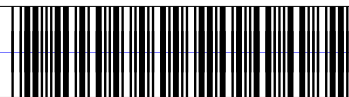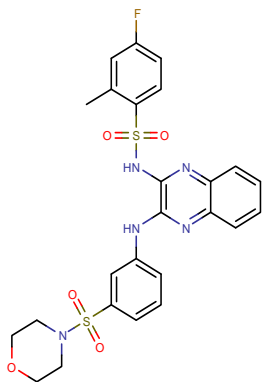

Mol Wt 557.62  
Exact Mass 557.13

| # | Time  | Area% |
|---|-------|-------|
| 1 | 0.927 | 2.60  |
| 2 | 1.566 | 97.40 |

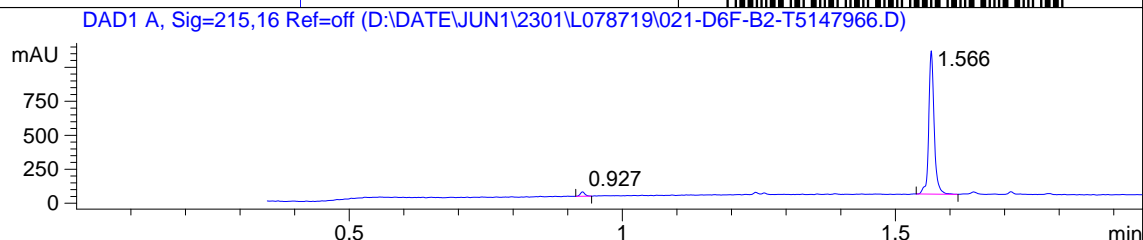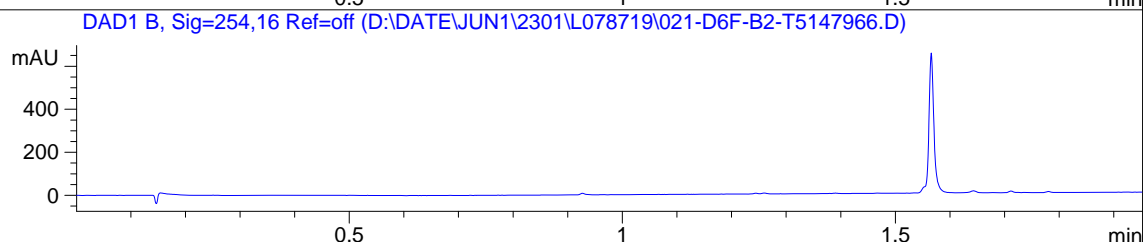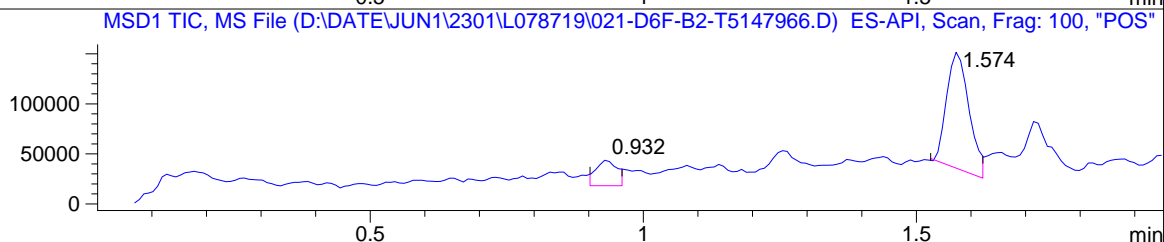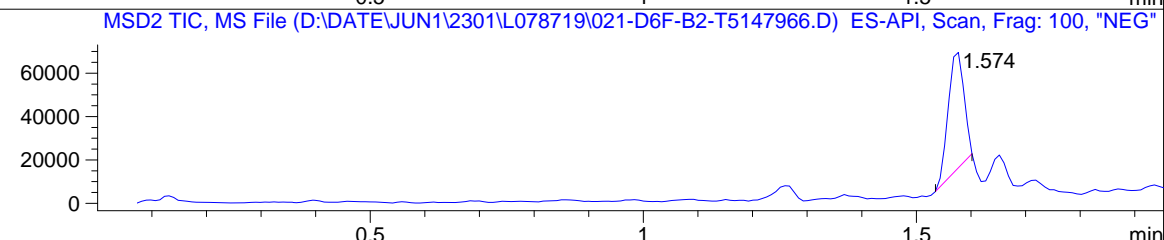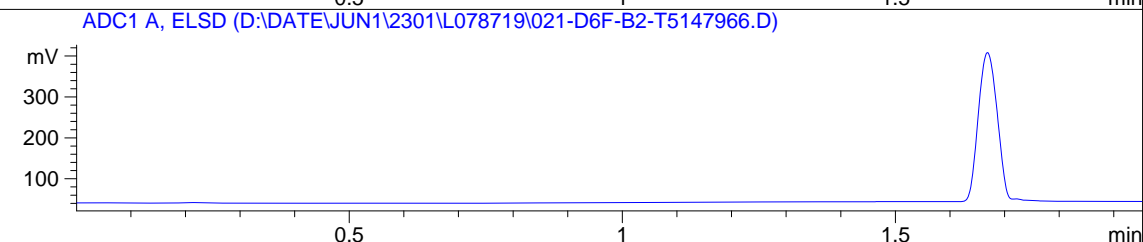

RT 0.932

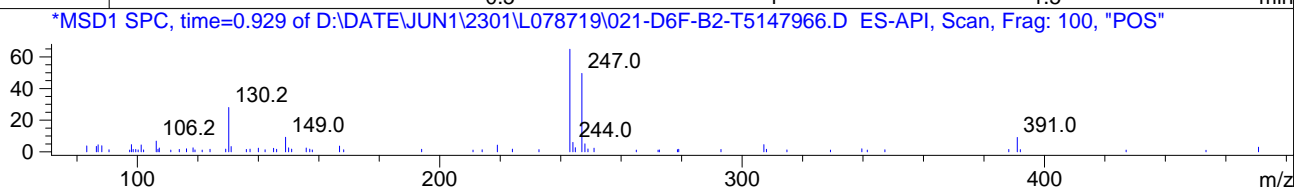

RT 1.574

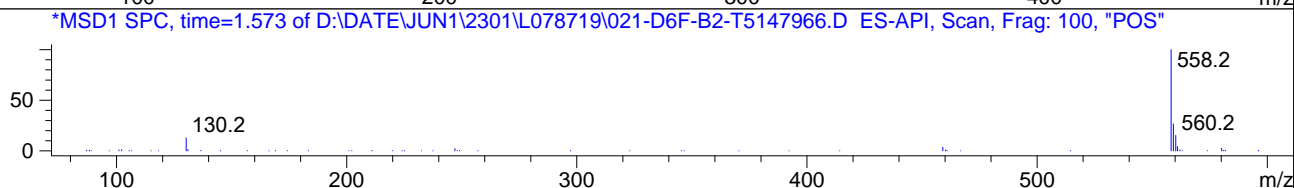

RT 1.574

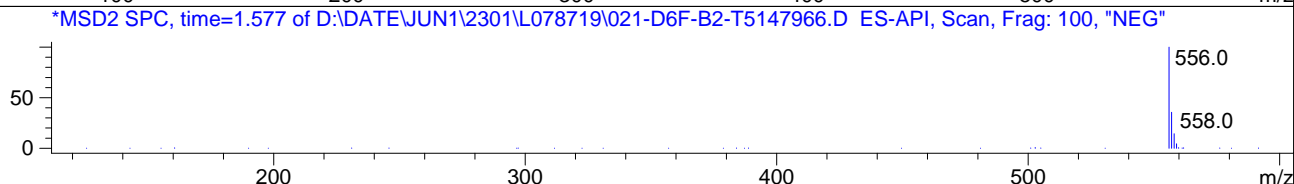

Supplement: Supplementary file 1 — Supplementary Information 1. [file 41598_2024_54655_MOESM1_ESM.zip › Nature SREP/QC_AIMS_files/Proj001.pdf]

MaxPeak: 94.78%  
Ret\_Time: 0.377 min

3848963

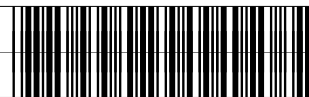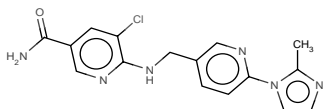

Mol Wt 342.783  
Exact Mass 342.11

| # | Time  | Area% |
|---|-------|-------|
| 1 | 0.377 | 94.78 |
| 2 | 0.560 | 0.69  |
| 3 | 0.626 | 1.04  |
| 4 | 0.666 | 1.86  |
| 5 | 0.829 | 0.74  |
| 6 | 1.125 | 0.89  |

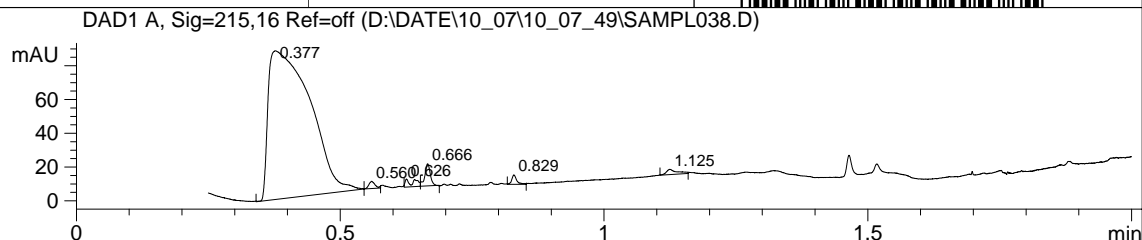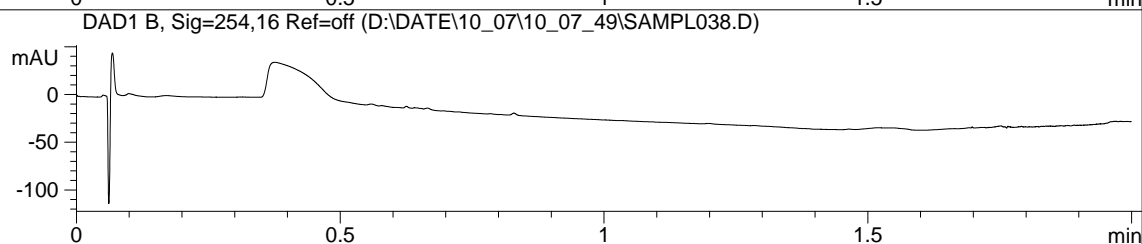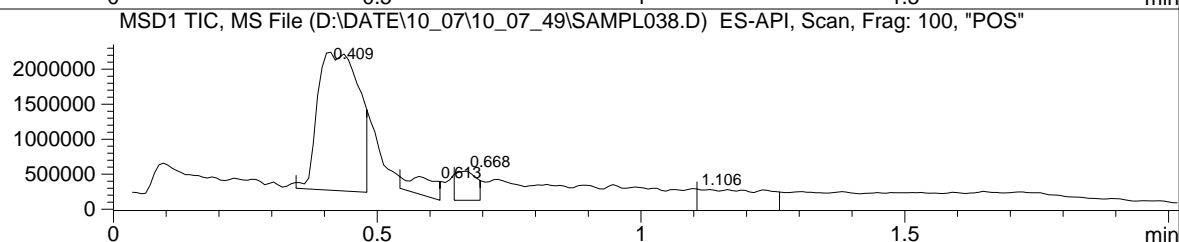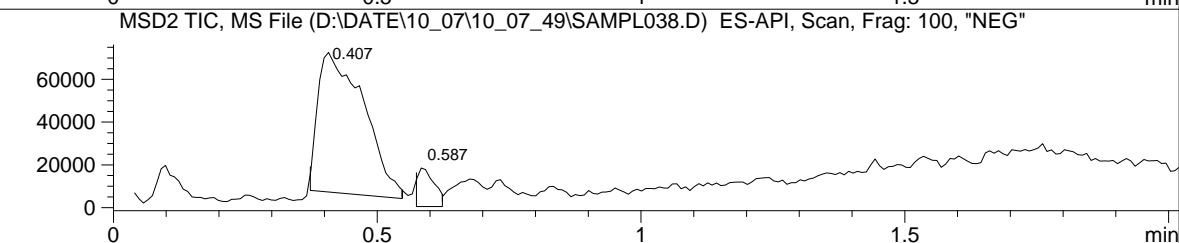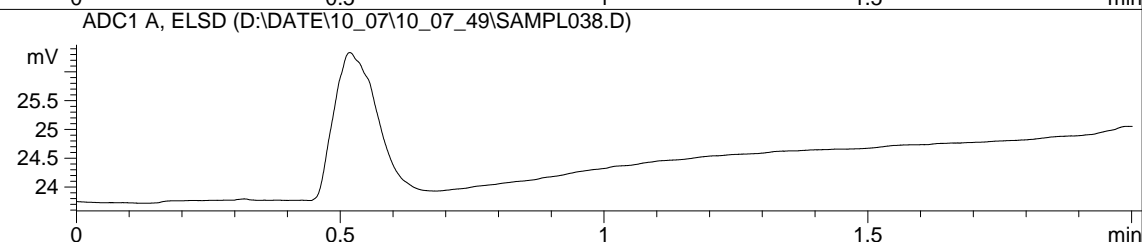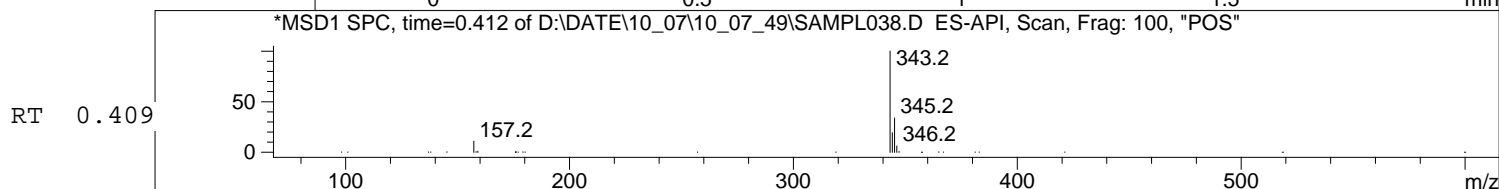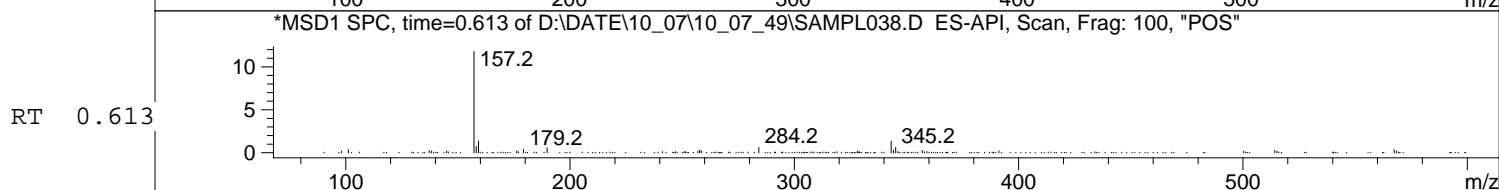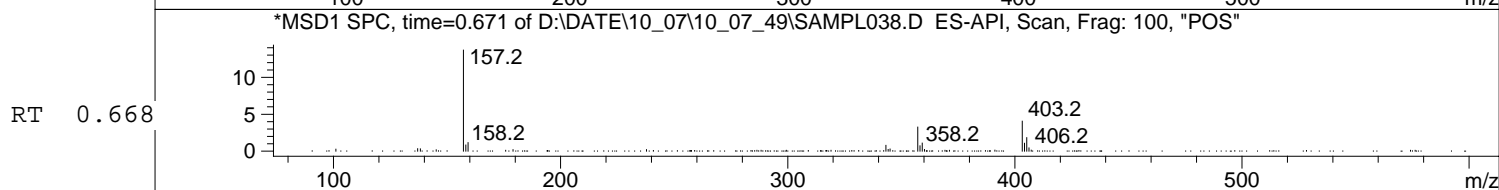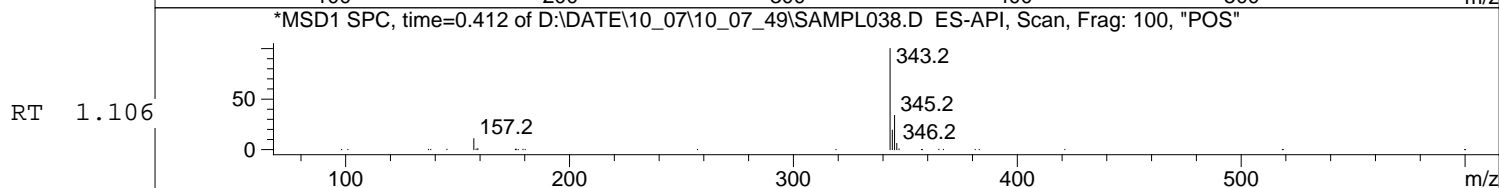

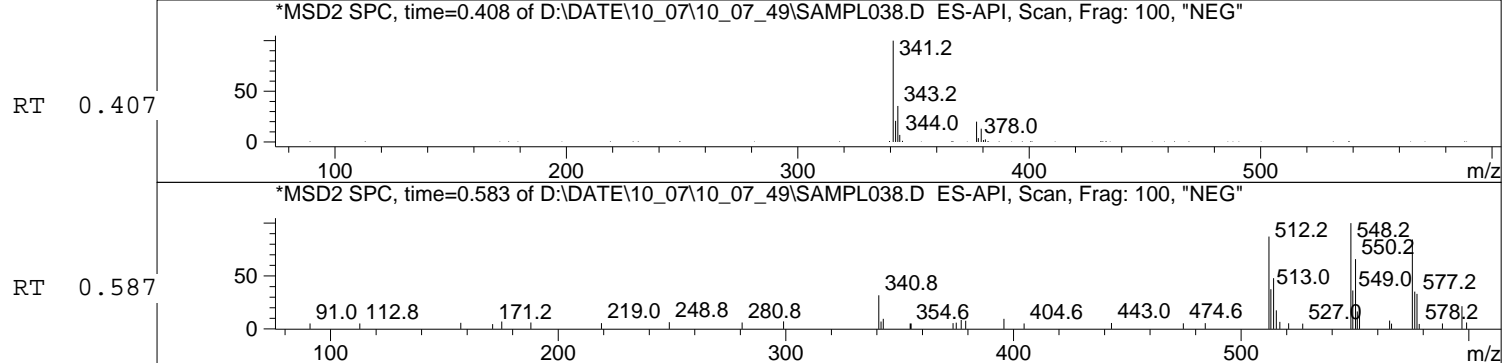

Supplement: Supplementary file 1 — Supplementary Information 1. [file 41598_2024_54655_MOESM1_ESM.zip › Nature SREP/QC_AIMS_files/Proj002.pdf]

Bruker AC-200, SF=200.13 MHz, 03-06-2022 Base: BBB8304-18

UGR35373 in DMSO-d6/CCl4

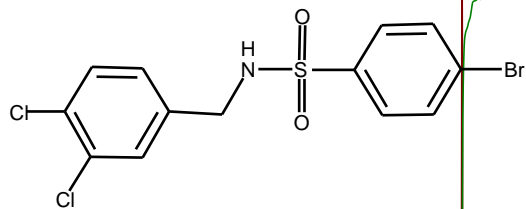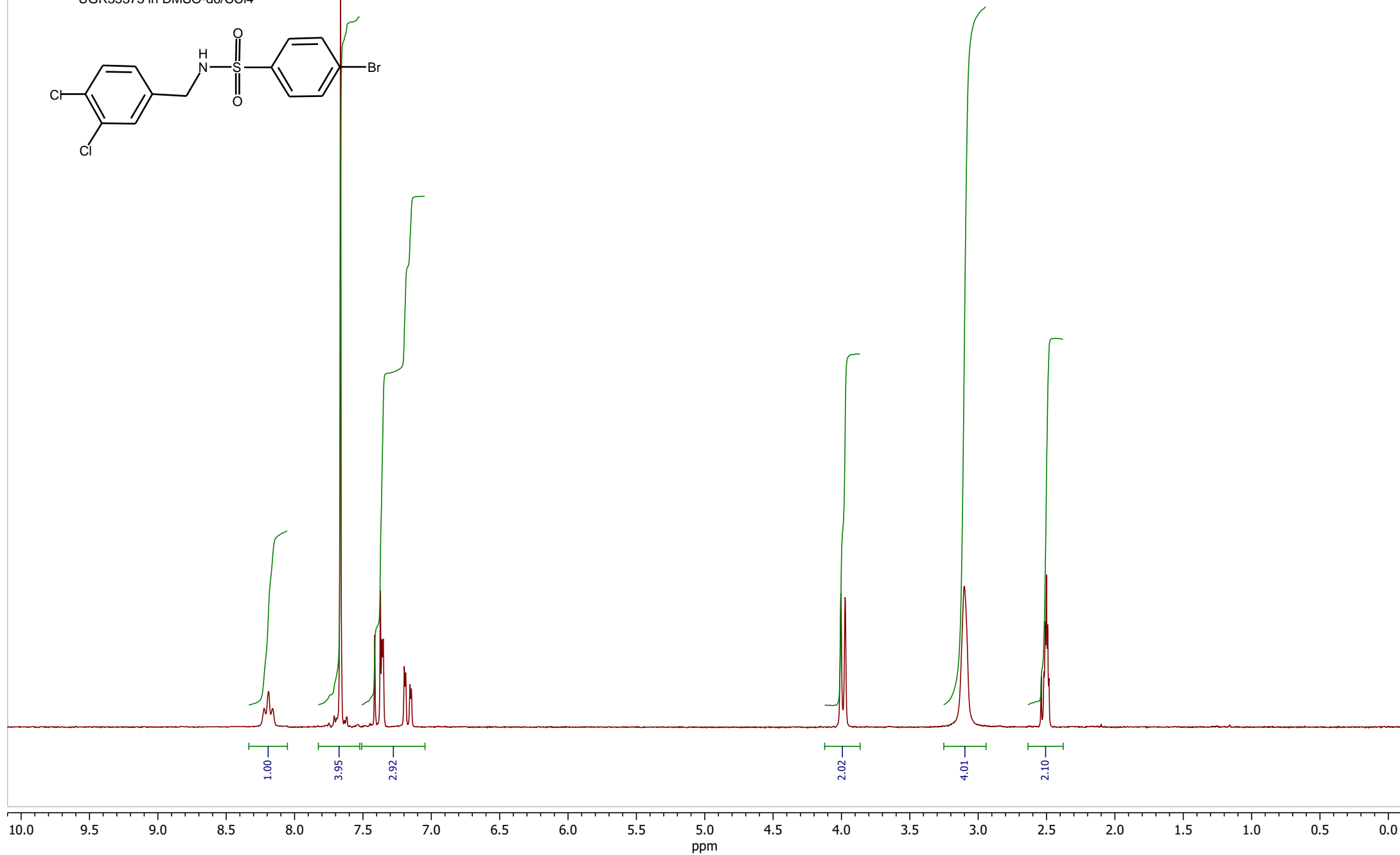

Supplement: Supplementary file 1 — Supplementary Information 1. [file 41598_2024_54655_MOESM1_ESM.zip › Nature SREP/QC_AIMS_files/Proj004.PDF]

T7549851

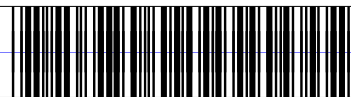

MaxPeak: 100.00%  
Ret\_Time: 0.828 min

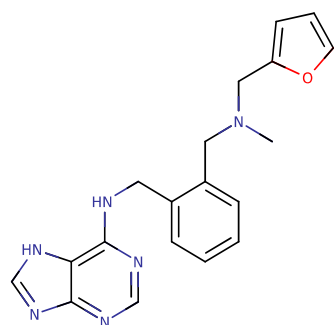

Mol Wt 348.4  
Exact Mass 348.19

| # | Time  | Area%  |
|---|-------|--------|
| 1 | 0.828 | 100.00 |

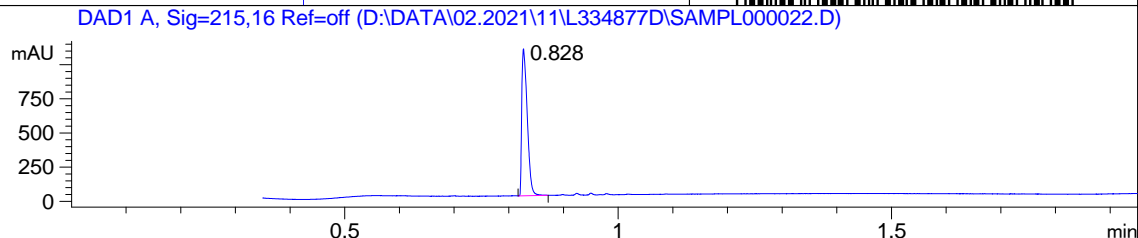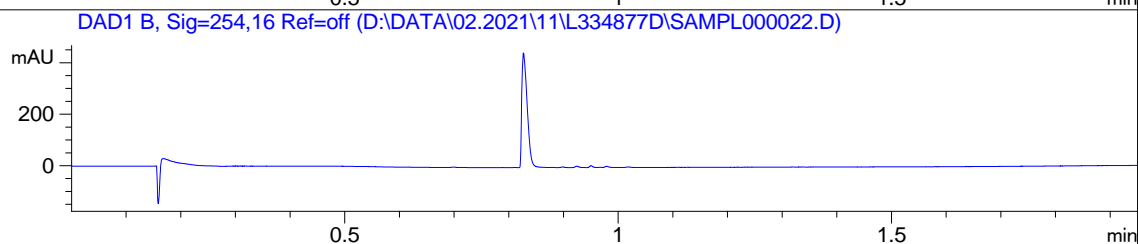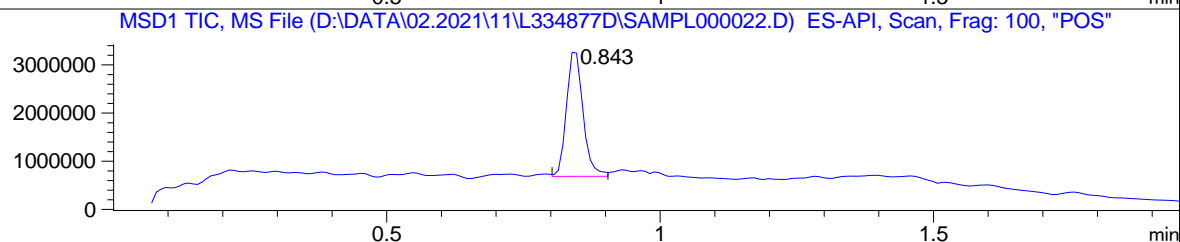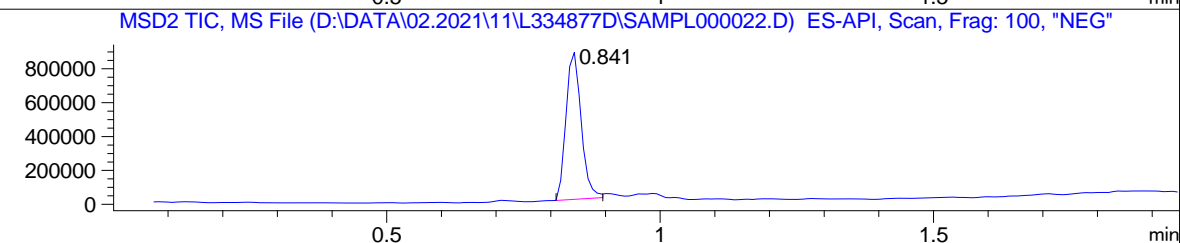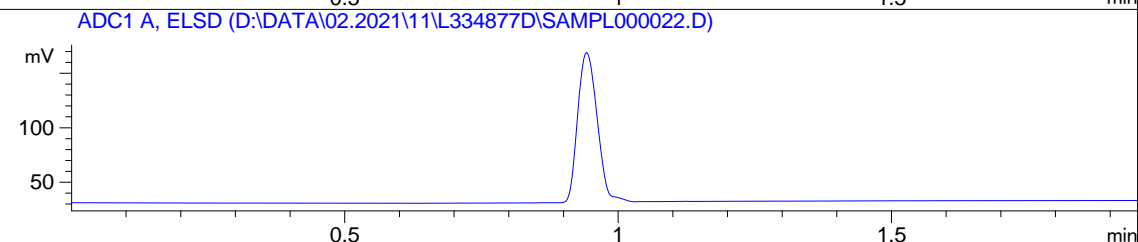

RT 0.843

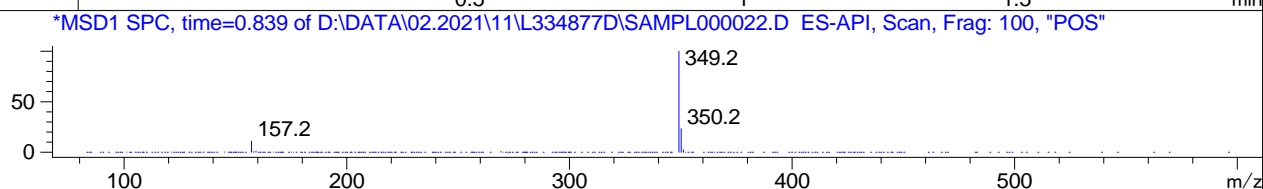

RT 0.841

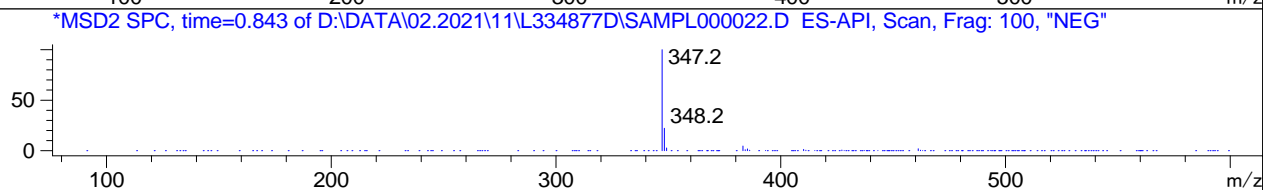

Supplement: Supplementary file 1 — Supplementary Information 1. [file 41598_2024_54655_MOESM1_ESM.zip › Nature SREP/QC_AIMS_files/Proj010.pdf]

MaxPeak: 100.00%  
Ret\_Time: 1.213 min

T6510957

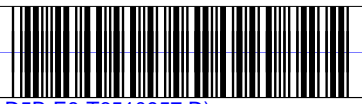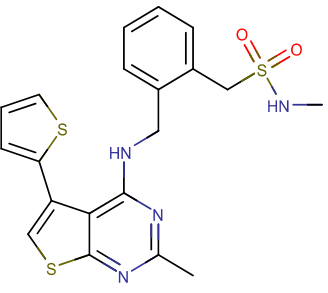

Mol Wt 444.59  
Exact Mass 444.09

| # | Time  | Area%  |
|---|-------|--------|
| 1 | 1.213 | 100.00 |

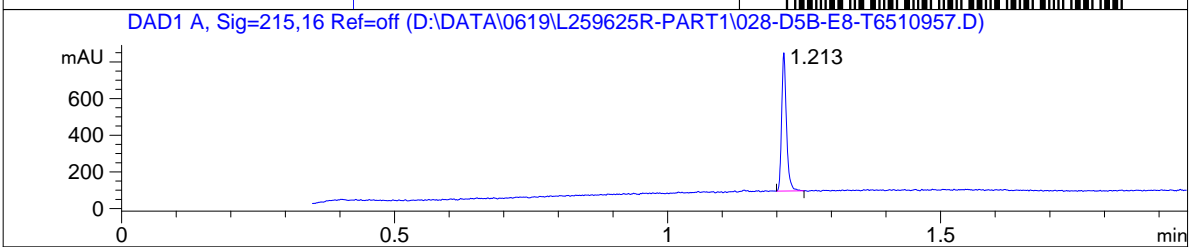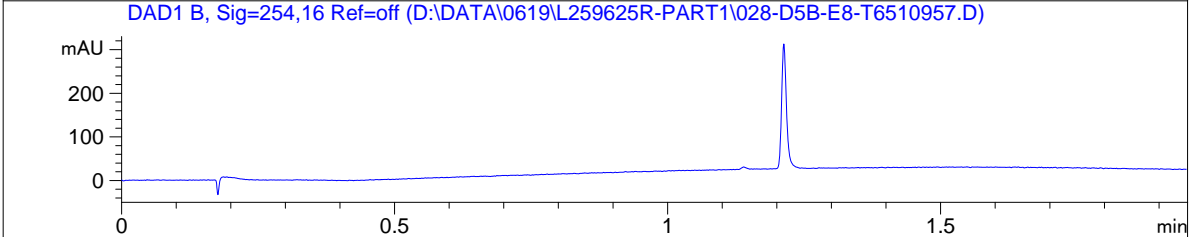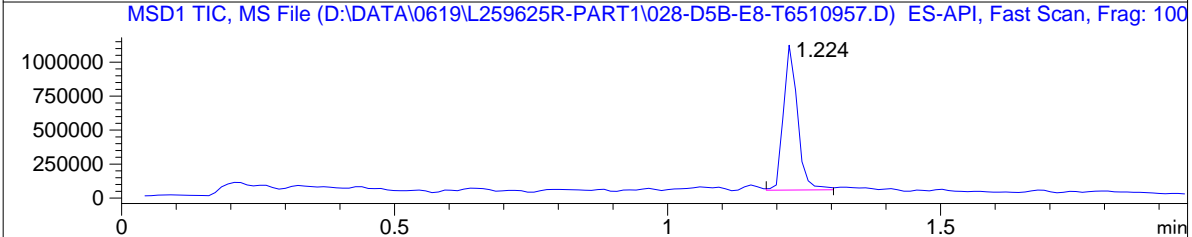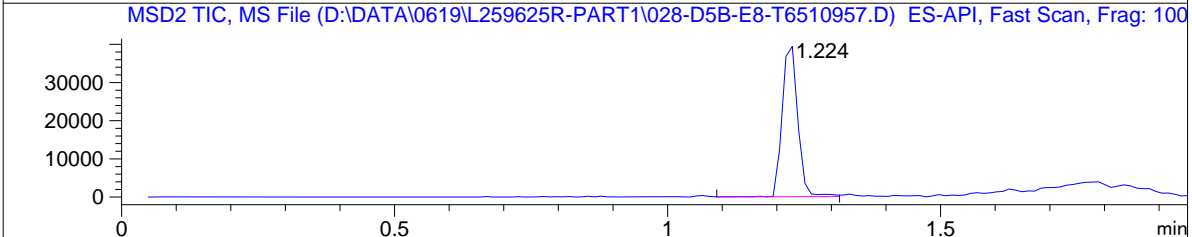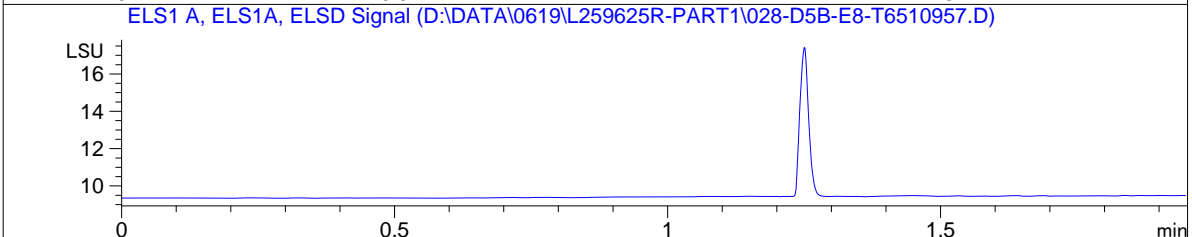

RT 1.224

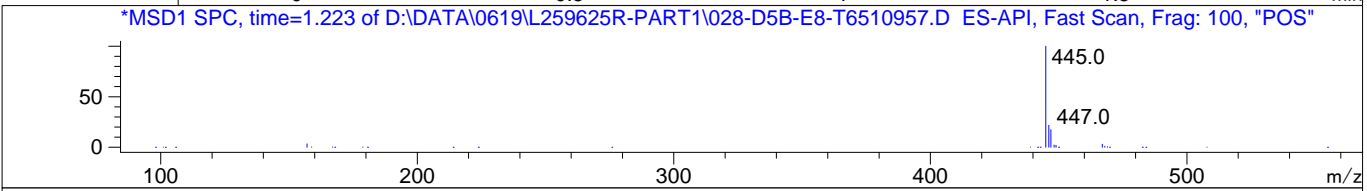

RT 1.224

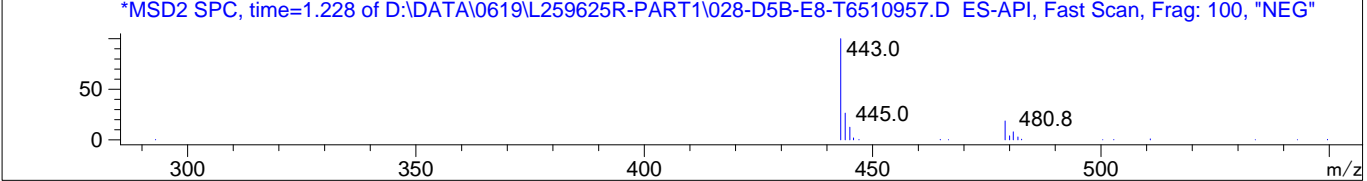

Supplement: Supplementary file 1 — Supplementary Information 1. [file 41598_2024_54655_MOESM1_ESM.zip › Nature SREP/QC_AIMS_files/Proj022.pdf]

UZI/1911604 ZU113

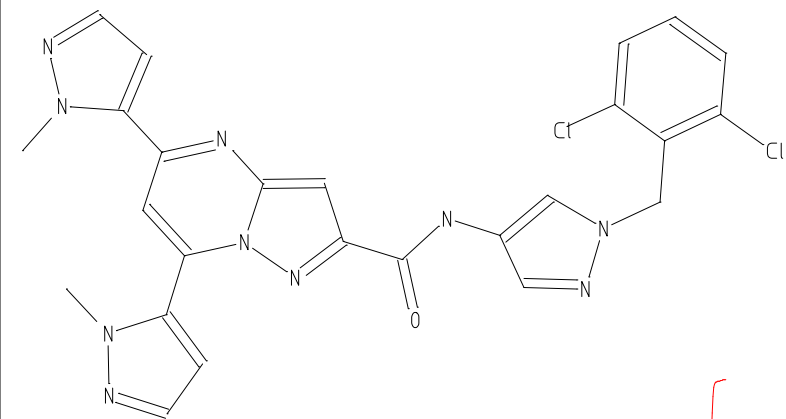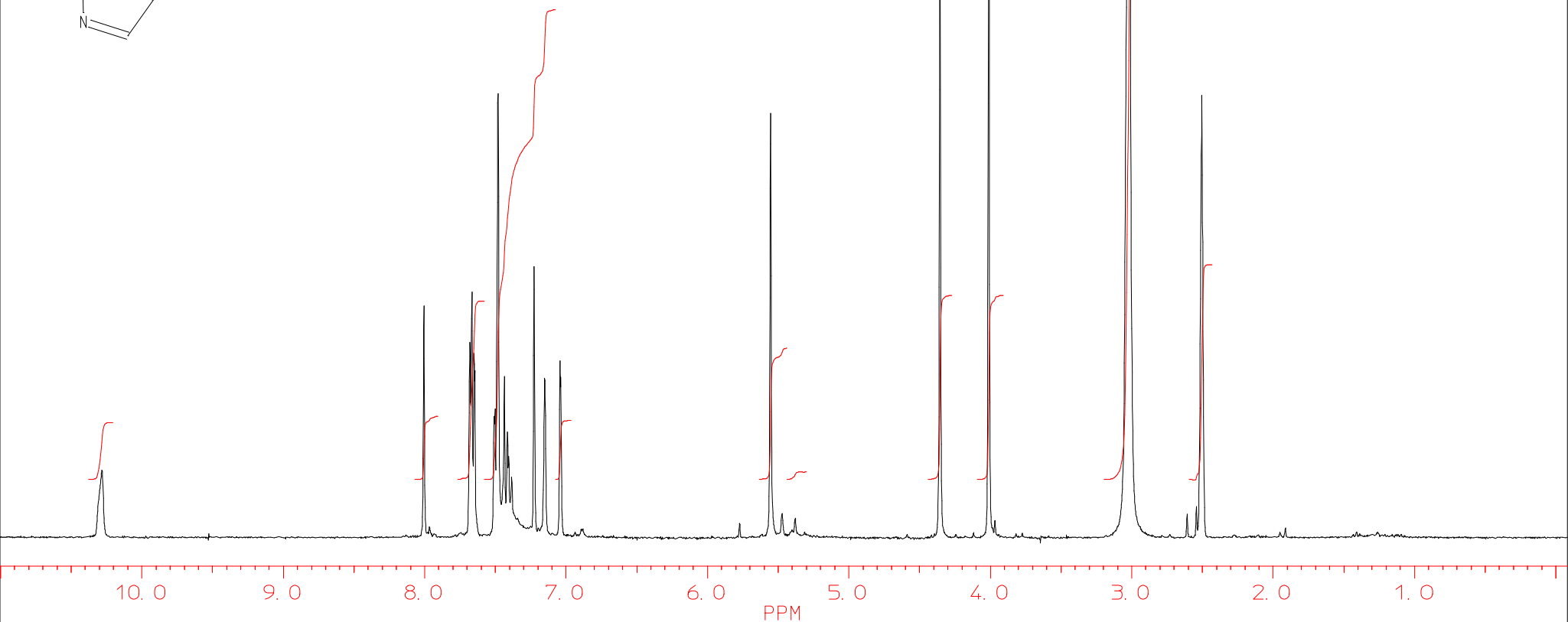

|                                                                                    |                      |                            |                          |                     |                                                                                       |
|------------------------------------------------------------------------------------|----------------------|----------------------------|--------------------------|---------------------|---------------------------------------------------------------------------------------|
| UZI/1911604 ZU113                                                                  | AC-300 SF=300.13 MHz | SI=16K, SW=5376.34, PW=2.5 | AQ=1.524, RD=3.00, NS=12 | SR=4788.46, TE=308K |                                                                                       |
| 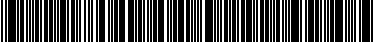 | Moscow, 16 June 2004 | Opr: A. N. SHUMSKY;        | Solv: DMSO-D6+CCL4;      | Prep: G-10325;      | 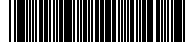 |

Supplement: Supplementary file 1 — Supplementary Information 1. [file 41598_2024_54655_MOESM1_ESM.zip › Nature SREP/QC_AIMS_files/Proj023.pdf]

MaxPeak: 96.52%  
Ret\_Time: 0.736 min

3479077

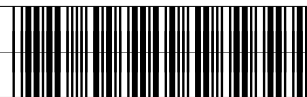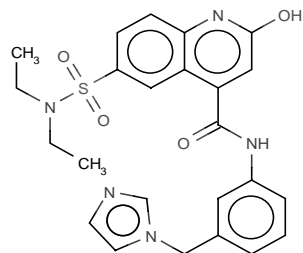

Mol Wt 479.551  
Exact Mass 479.18

| # | Time  | Area% |
|---|-------|-------|
| 1 | 0.718 | 2.00  |
| 2 | 0.736 | 96.52 |
| 3 | 1.132 | 1.48  |

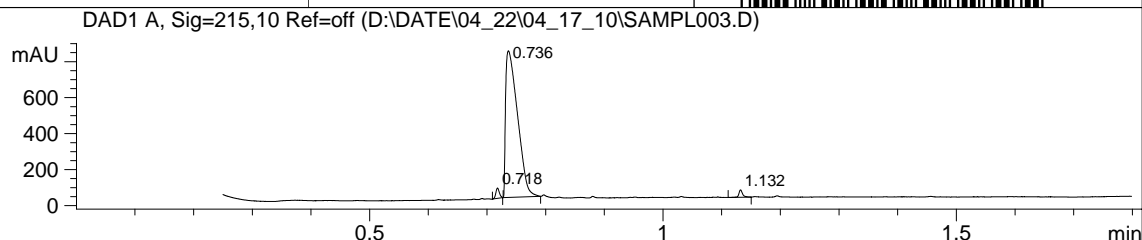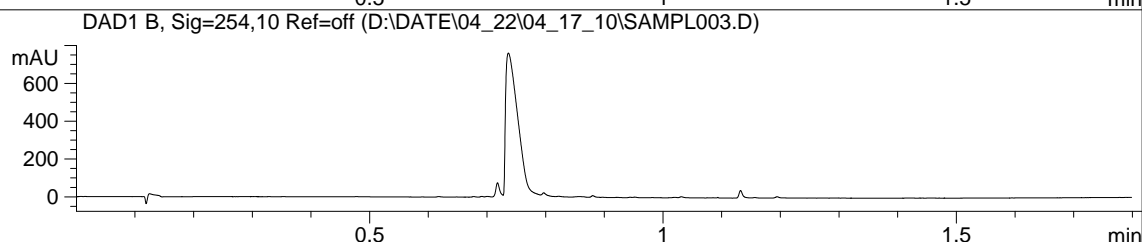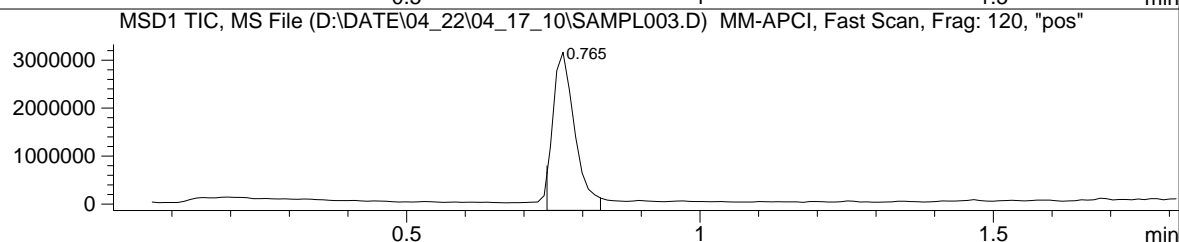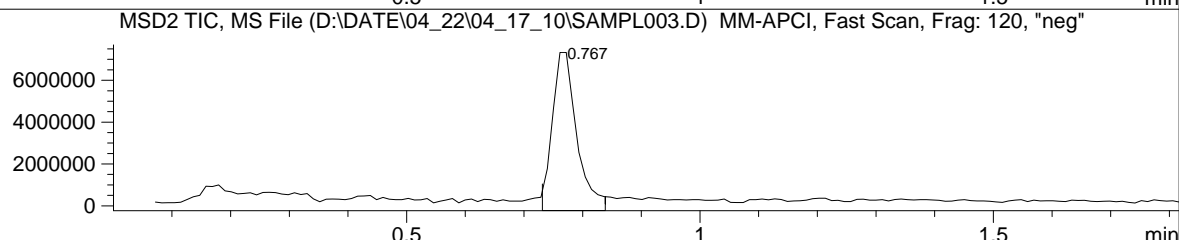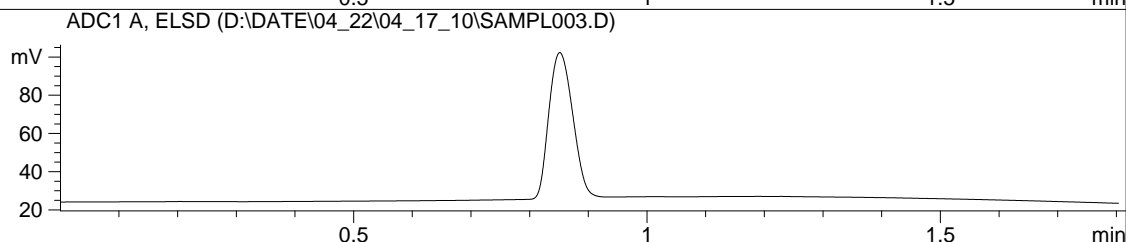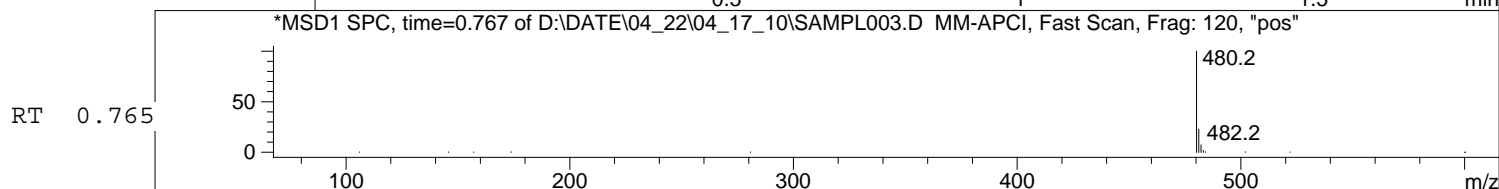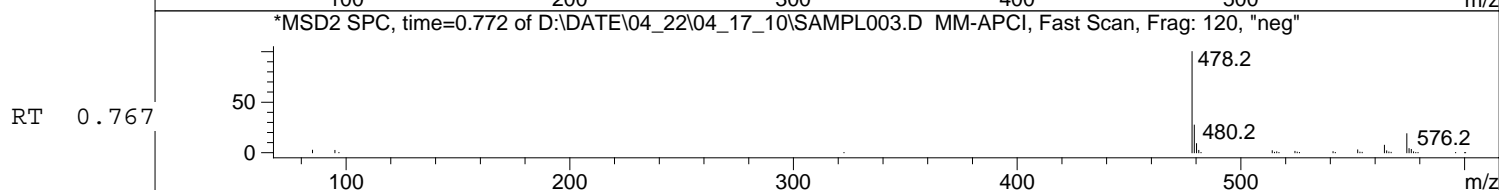

Supplement: Supplementary file 1 — Supplementary Information 1. [file 41598_2024_54655_MOESM1_ESM.zip › Nature SREP/QC_AIMS_files/Proj026.pdf]

T5949299

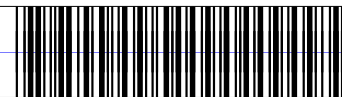

MaxPeak: 84.06%  
Ret\_Time: 1.271 min

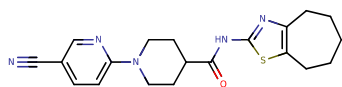

Mol Wt 381.5  
Exact Mass 381.19

| # | Time  | Area% |
|---|-------|-------|
| 1 | 0.845 | 2.22  |
| 2 | 0.898 | 2.16  |
| 3 | 1.230 | 1.76  |
| 4 | 1.248 | 9.81  |
| 5 | 1.271 | 84.06 |

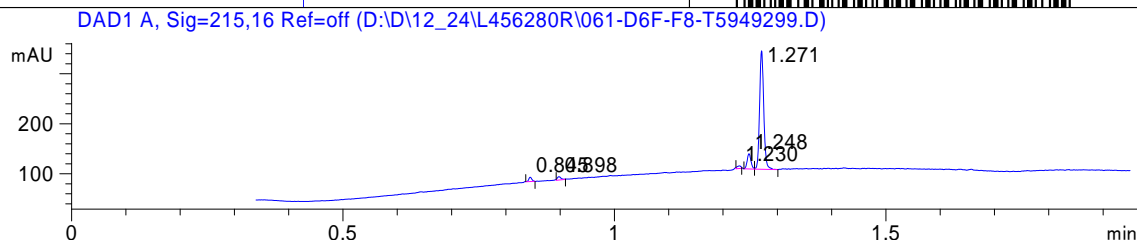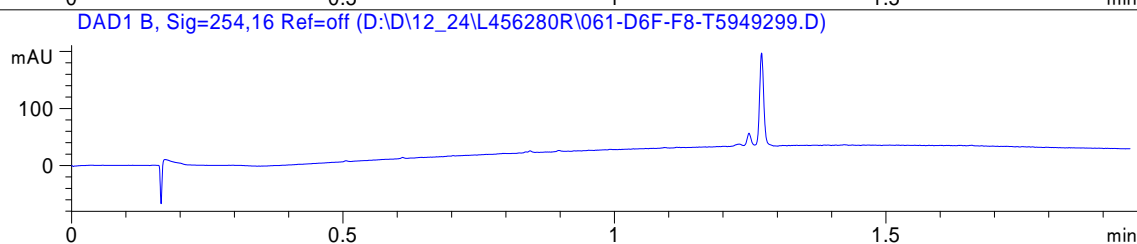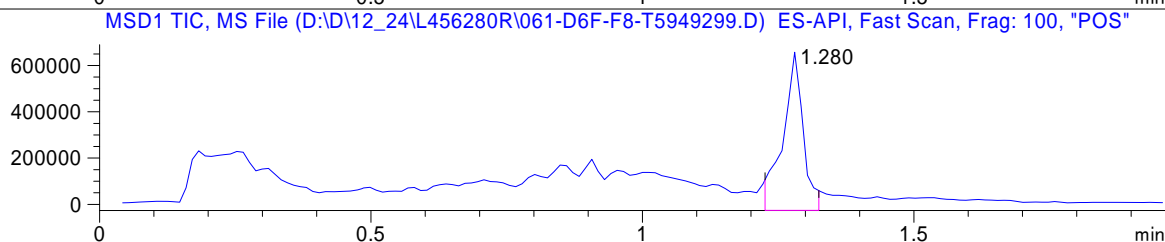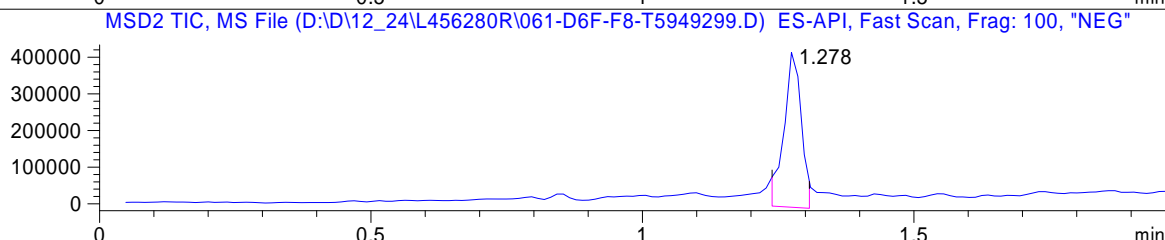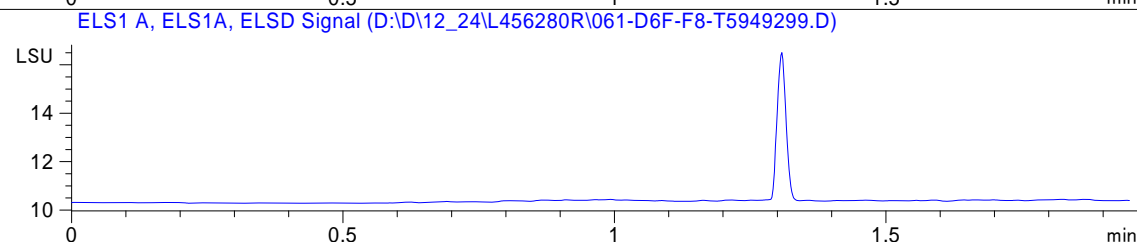

RT 1.280

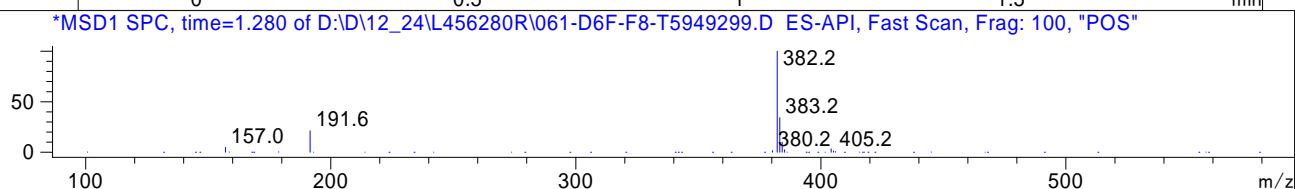

RT 1.278

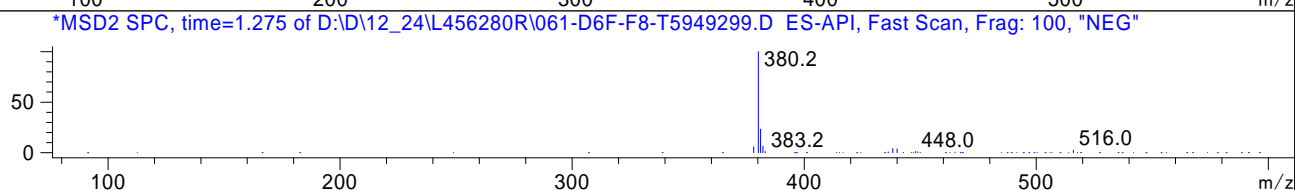

Supplement: Supplementary file 1 — Supplementary Information 1. [file 41598_2024_54655_MOESM1_ESM.zip › Nature SREP/QC_AIMS_files/Proj029.pdf]

$^1\text{H}$  NMR (400 MHz,  $\text{DMSO}-d_6$ )  $\delta$  ppm 1.40 (s, 9 H) 5.83 (d,  $J=5.13$  Hz, 1 H) 6.78 (s, 2 H) 7.13 - 7.33 (m, 3 H) 7.33 - 7.41 (m, 1 H) 7.41 - 7.52 (m, 1 H) 7.69 (tdd,  $J=9.41, 9.41, 6.05, 4.22$  Hz, 1 H) 7.98 (d,  $J=5.14$  Hz, 1 H) 10.90 (s, 1 H)

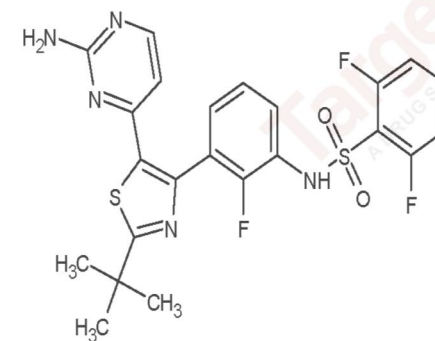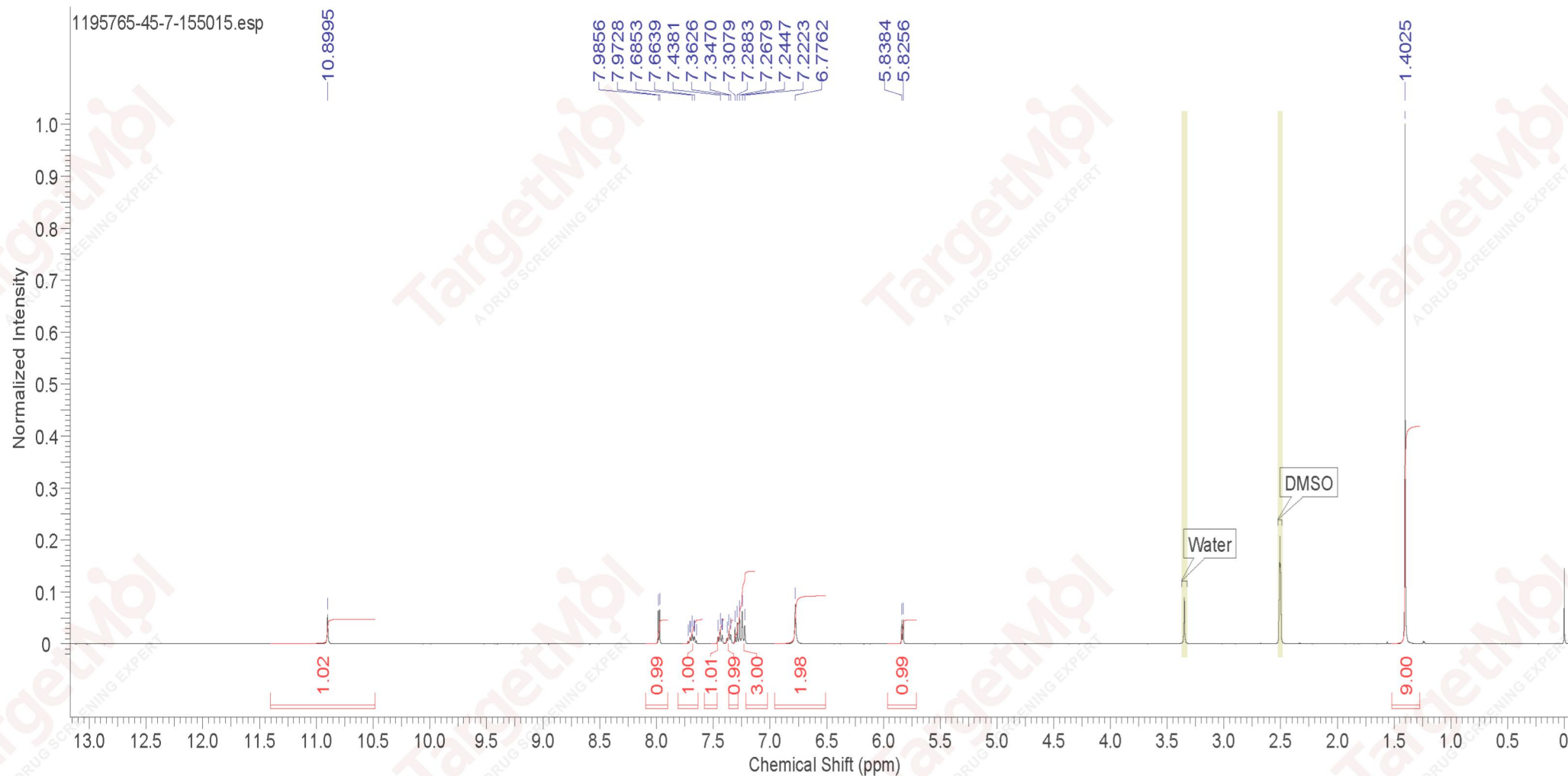

Supplement: Supplementary file 1 — Supplementary Information 1. [file 41598_2024_54655_MOESM1_ESM.zip › Nature SREP/QC_AIMS_files/Proj035.pdf]

NMR:S787101

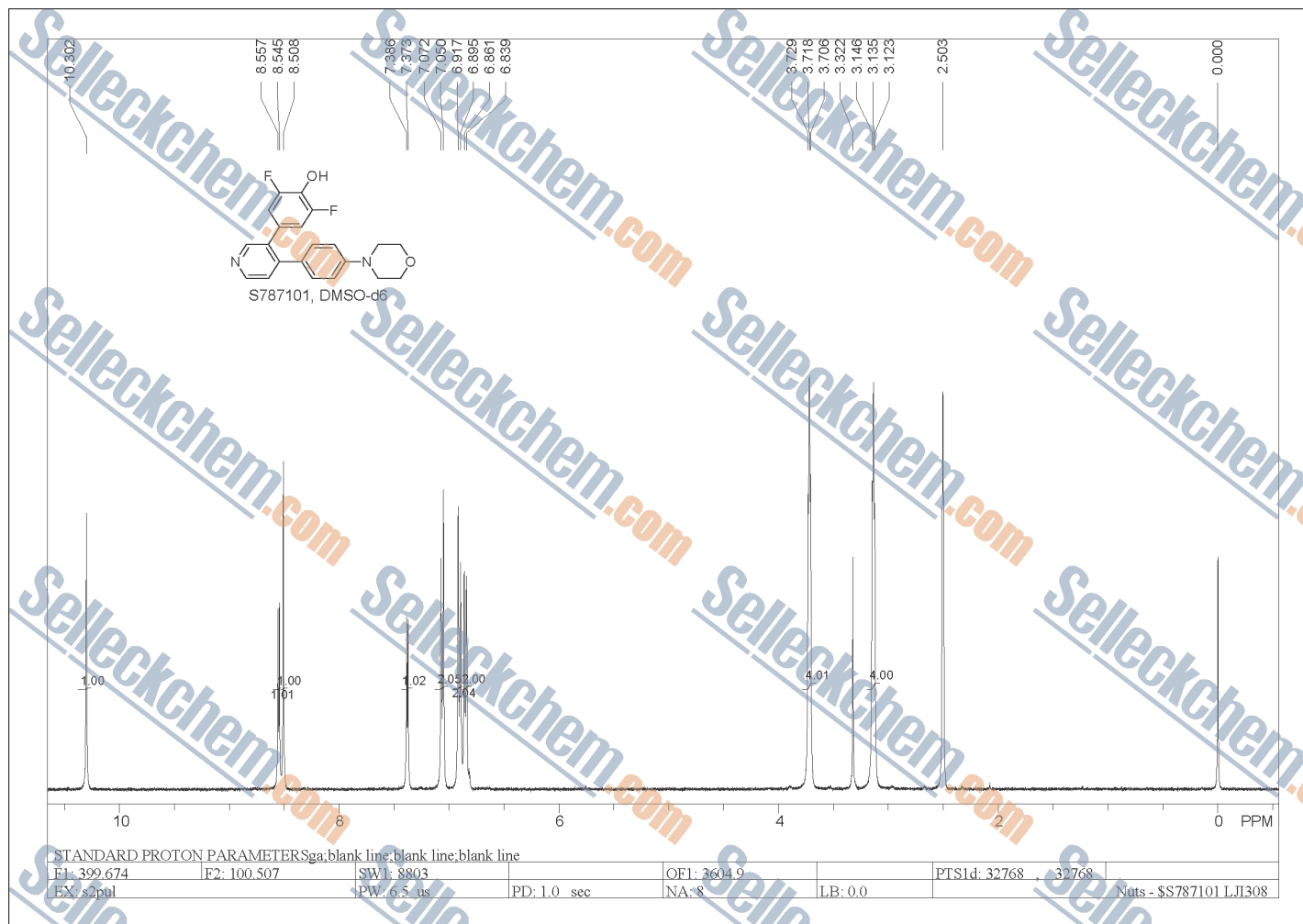

Supplement: Supplementary file 1 — Supplementary Information 1. [file 41598_2024_54655_MOESM1_ESM.zip › Nature SREP/QC_AIMS_files/Proj037.pdf]

MaxPeak: 96.35%  
Ret\_Time: 0.957 min

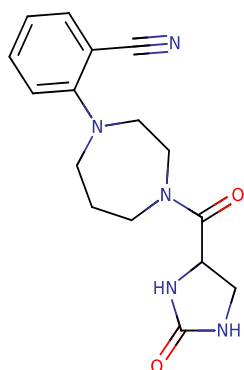

Mol Wt 313.35  
Exact Mass 313.17

| # | Time  | Area% |
|---|-------|-------|
| 1 | 0.957 | 96.35 |
| 2 | 1.422 | 3.65  |

T7569747

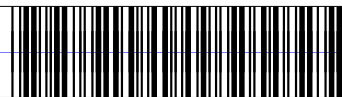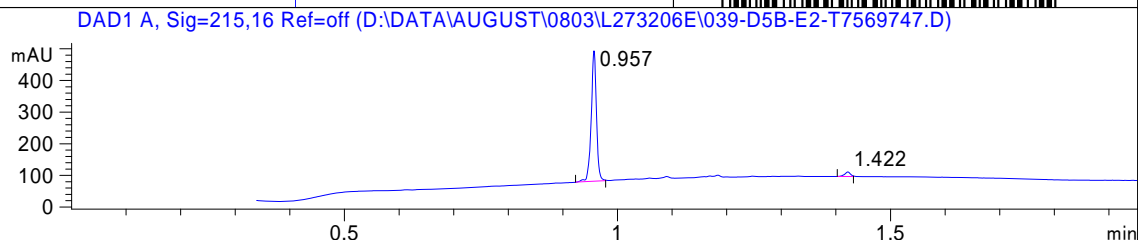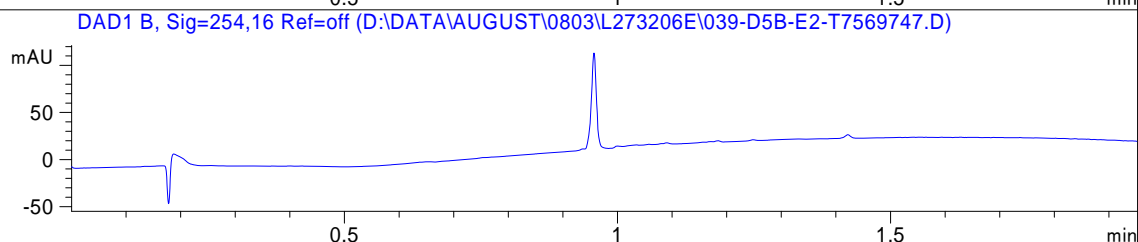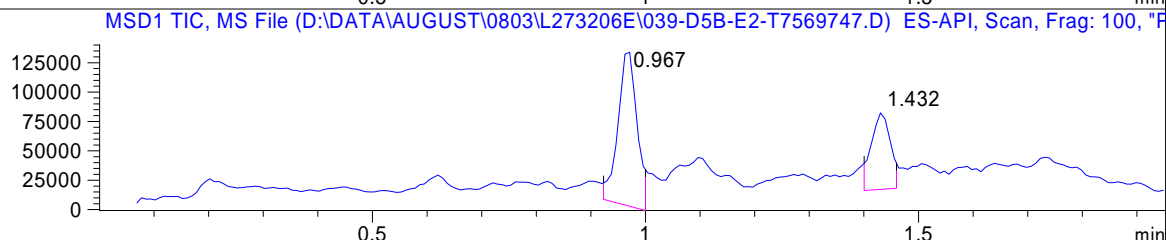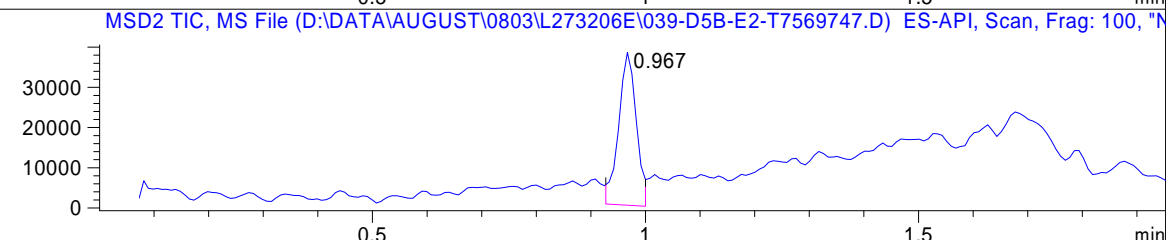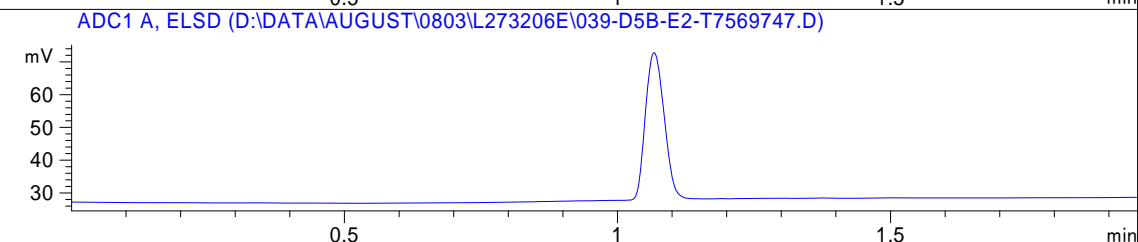

RT 0.967

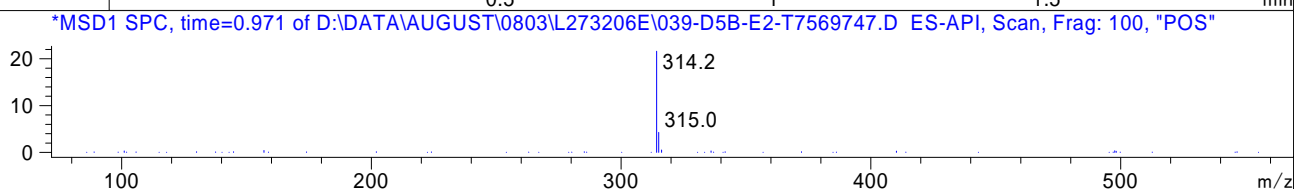

RT 1.432

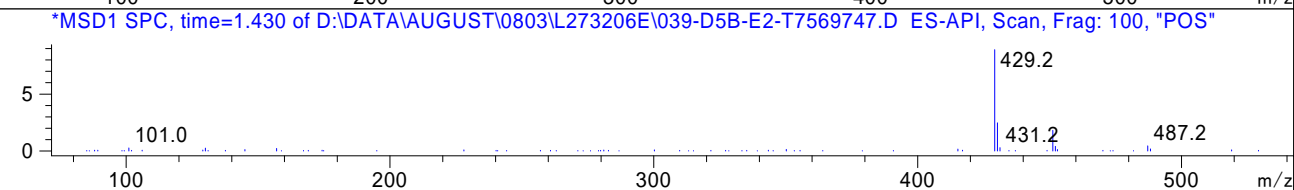

RT 0.967

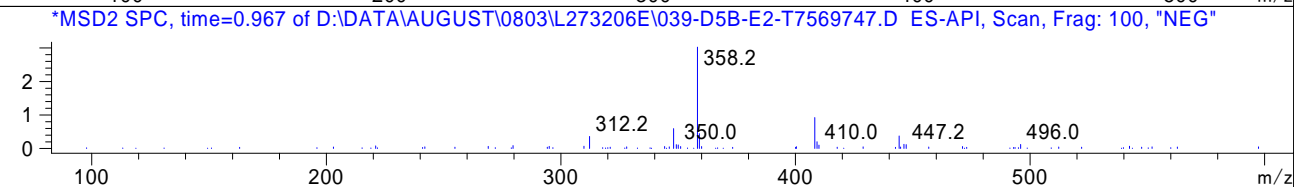

Supplement: Supplementary file 1 — Supplementary Information 1. [file 41598_2024_54655_MOESM1_ESM.zip › Nature SREP/QC_AIMS_files/Proj039.pdf]

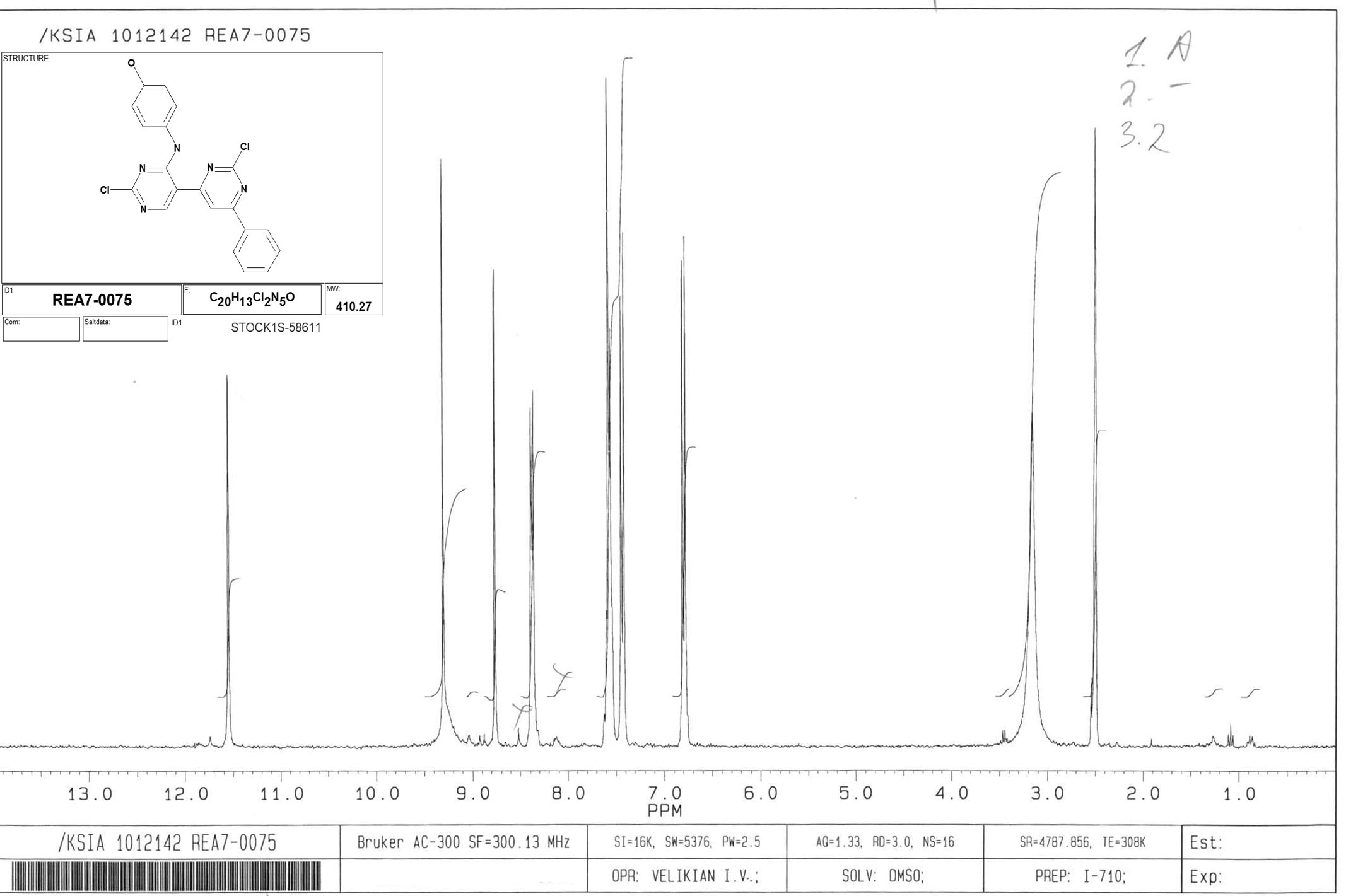

Supplement: Supplementary file 1 — Supplementary Information 1. [file 41598_2024_54655_MOESM1_ESM.zip › Nature SREP/QC_AIMS_files/Proj053.jpg]

MaxPeak: 95.93%  
Ret\_Time: 1.147 min

2462813

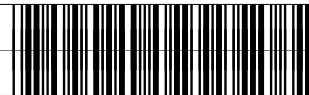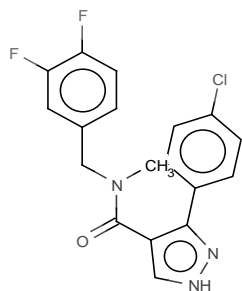

Mol Wt 361.773  
Exact Mass 361.1

| # | Time  | Area% |
|---|-------|-------|
| 1 | 0.867 | 4.07  |
| 2 | 1.147 | 95.93 |

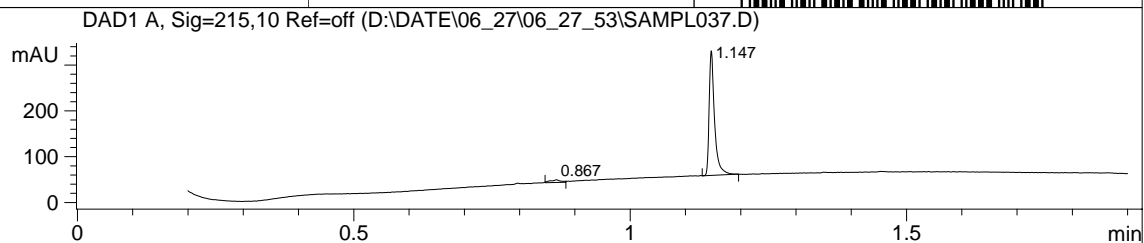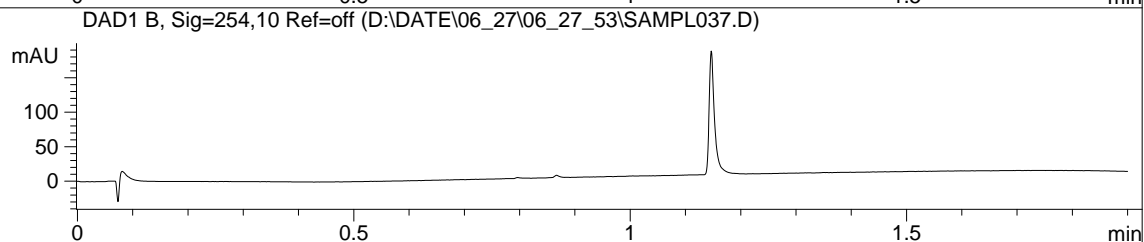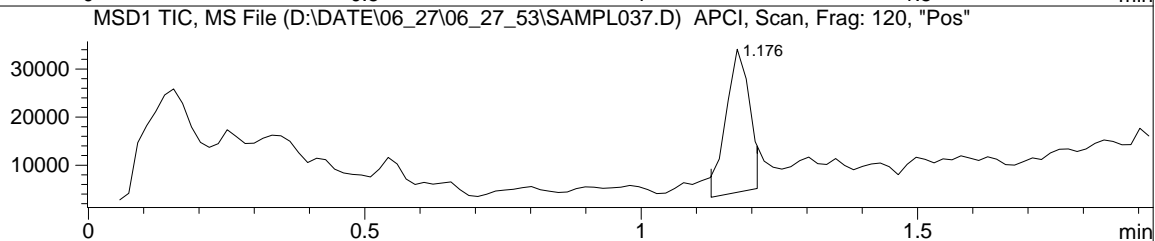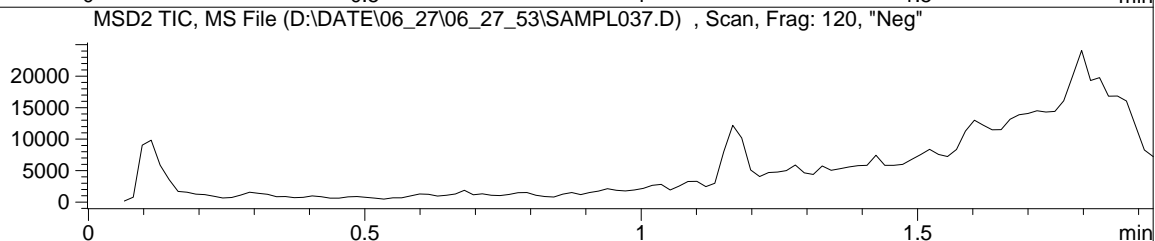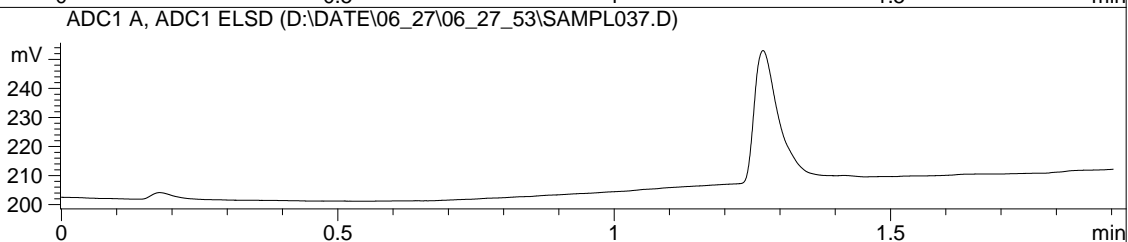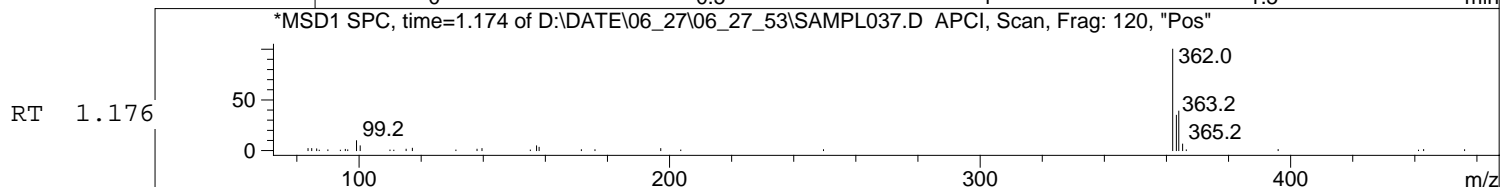

Supplement: Supplementary file 1 — Supplementary Information 1. [file 41598_2024_54655_MOESM1_ESM.zip › Nature SREP/QC_AIMS_files/Proj056.pdf]

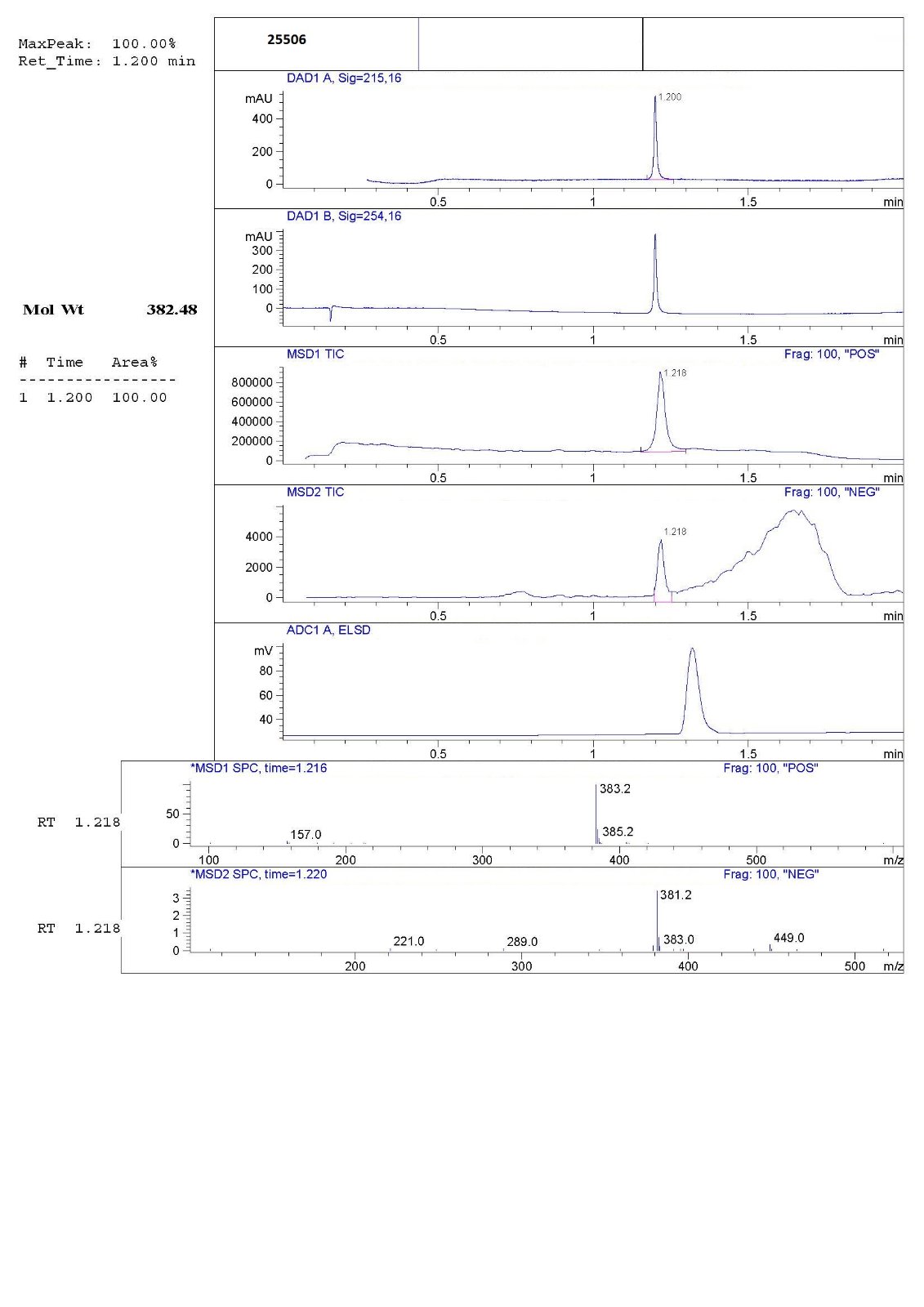

Supplement: Supplementary file 1 — Supplementary Information 1. [file 41598_2024_54655_MOESM1_ESM.zip › Nature SREP/QC_AIMS_files/Proj066.jpg]

T8342493

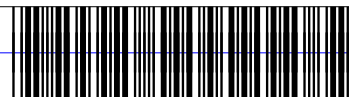

MaxPeak: 95.04%  
Ret\_Time: 0.379 min

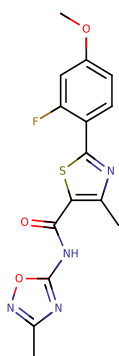

Mol Wt 348.35  
Exact Mass 348.07

| # | Time  | Area% |
|---|-------|-------|
| 1 | 0.352 | 4.96  |
| 2 | 0.379 | 95.04 |

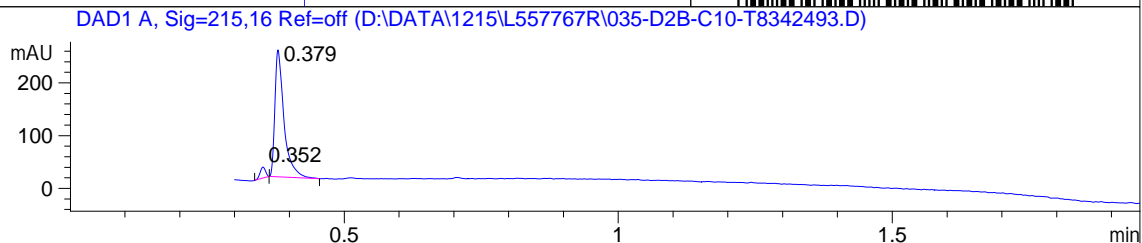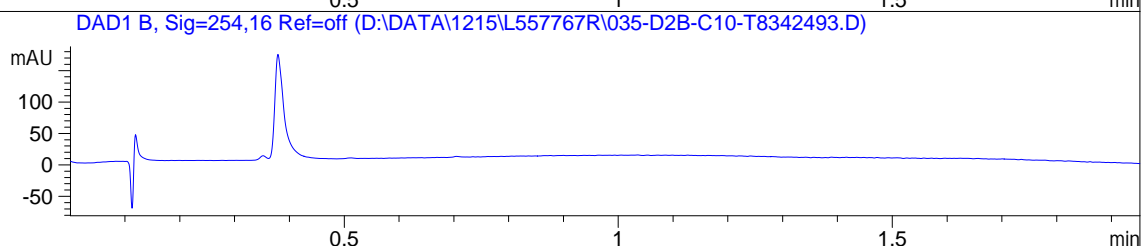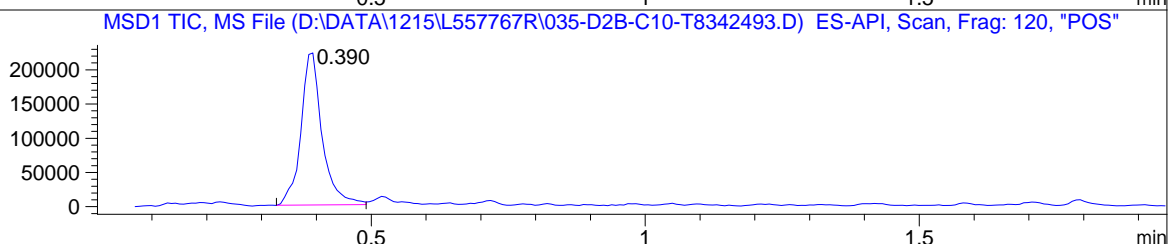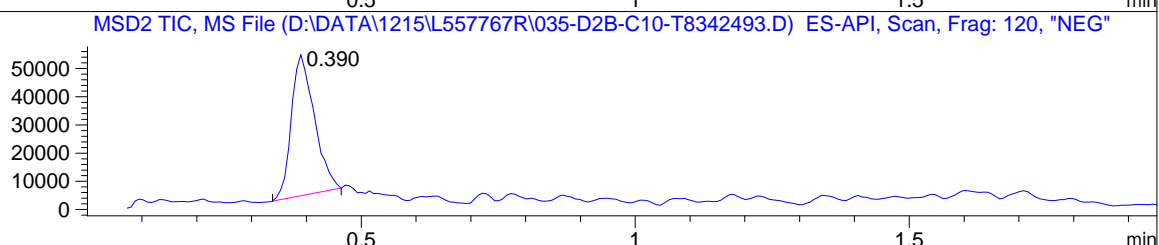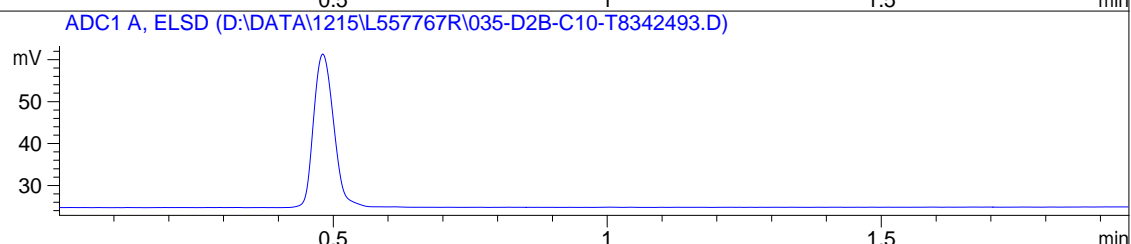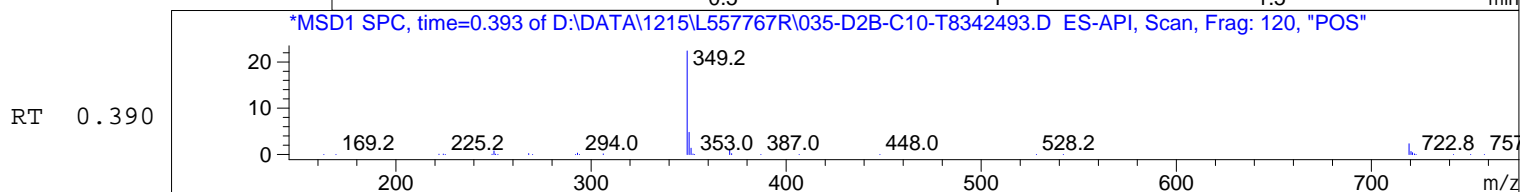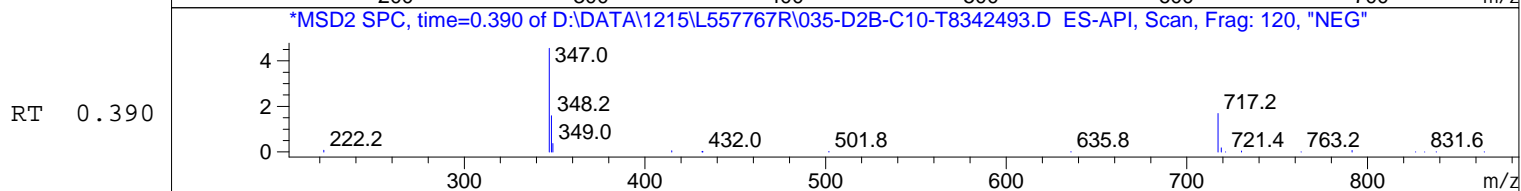

Supplement: Supplementary file 1 — Supplementary Information 1. [file 41598_2024_54655_MOESM1_ESM.zip › Nature SREP/QC_AIMS_files/Proj072.pdf]

T7630858

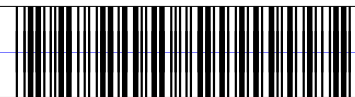

MaxPeak: 94.36%  
Ret\_Time: 1.208 min

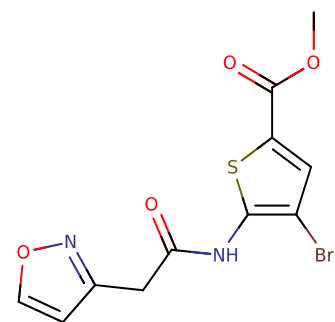

Mol Wt 345.17  
Exact Mass 345.94

| # | Time  | Area% |
|---|-------|-------|
| 1 | 1.121 | 4.59  |
| 2 | 1.208 | 94.36 |
| 3 | 1.345 | 1.05  |

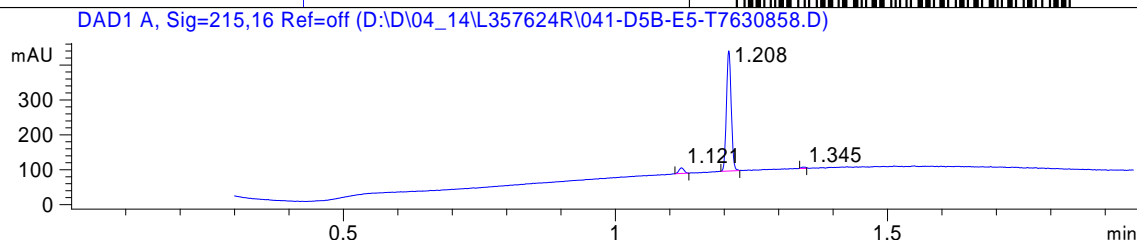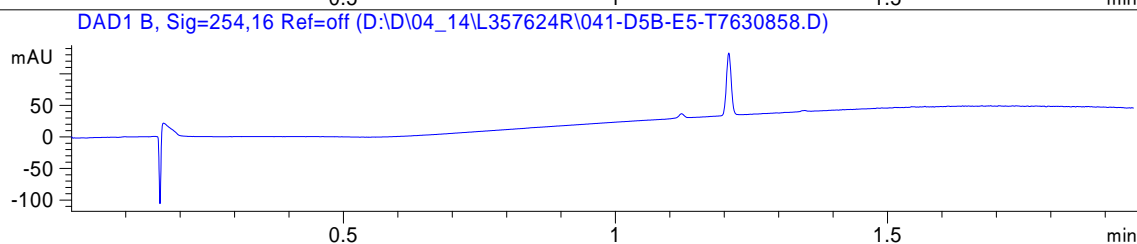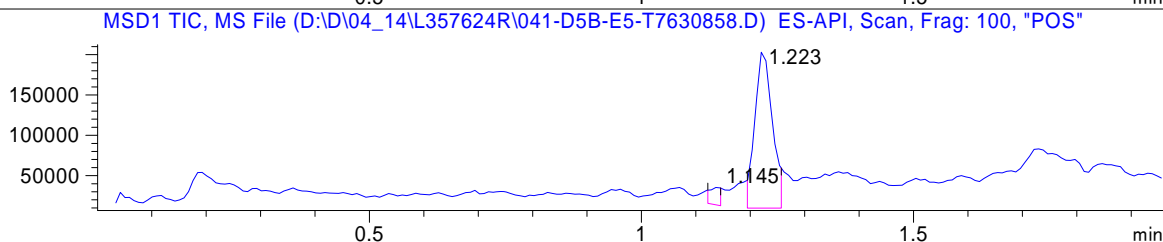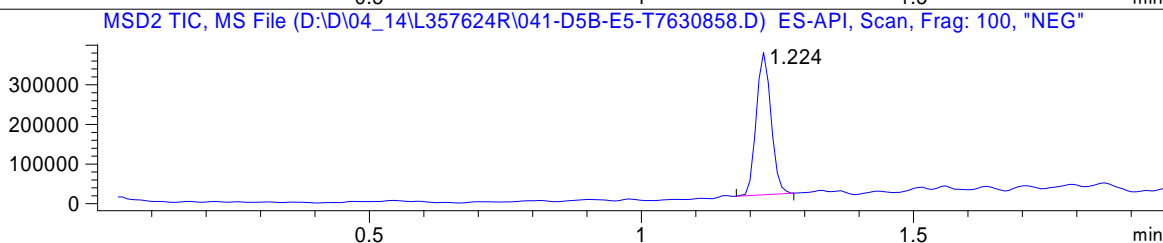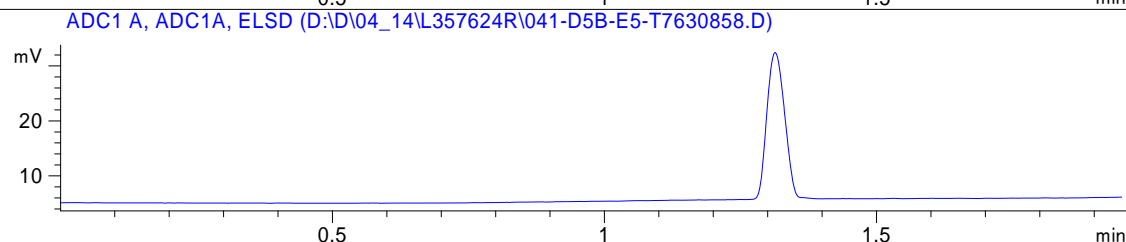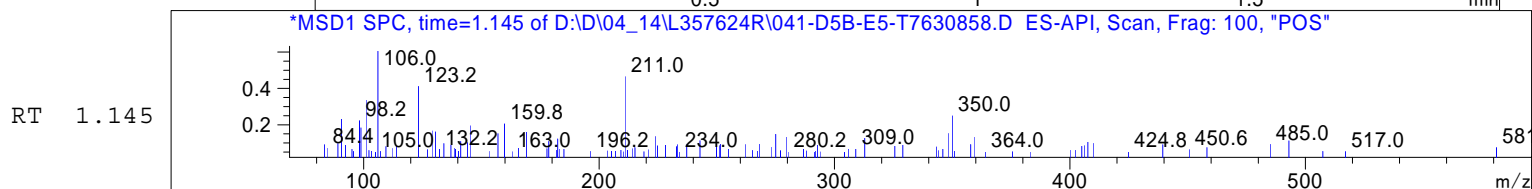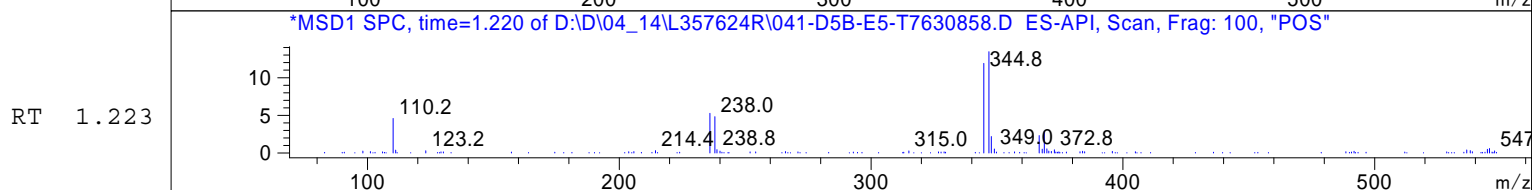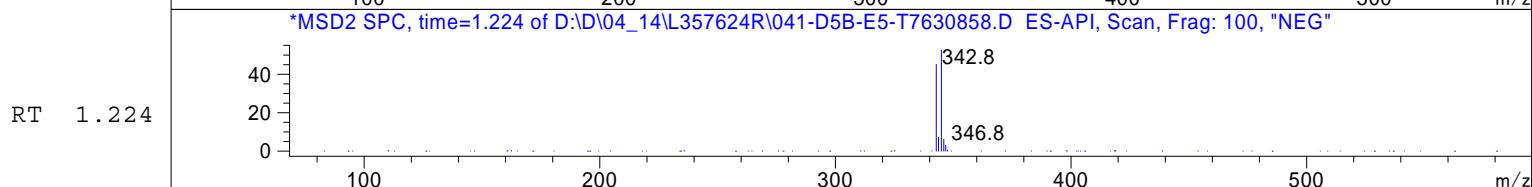

Supplement: Supplementary file 1 — Supplementary Information 1. [file 41598_2024_54655_MOESM1_ESM.zip › Nature SREP/QC_AIMS_files/Proj073.pdf]

T5587880

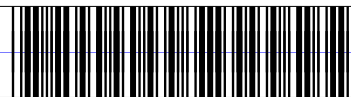

MaxPeak: 100.00%  
Ret\_Time: 1.049 min

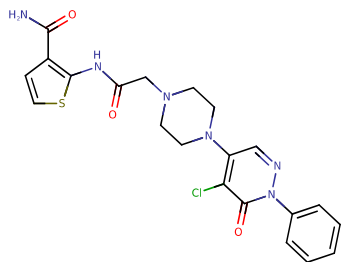

Mol Wt 472.95  
Exact Mass 472.12

| # | Time  | Area%  |
|---|-------|--------|
| 1 | 1.049 | 100.00 |

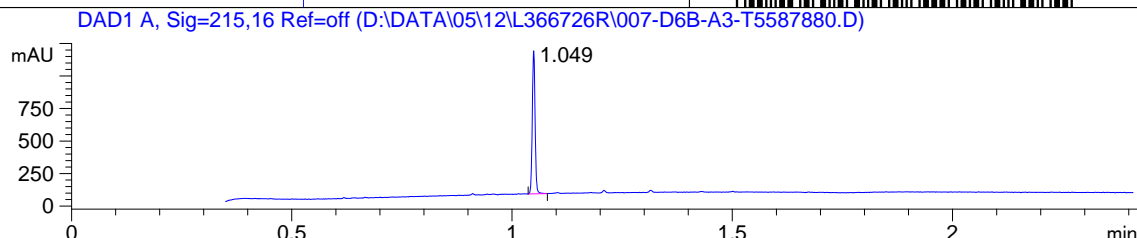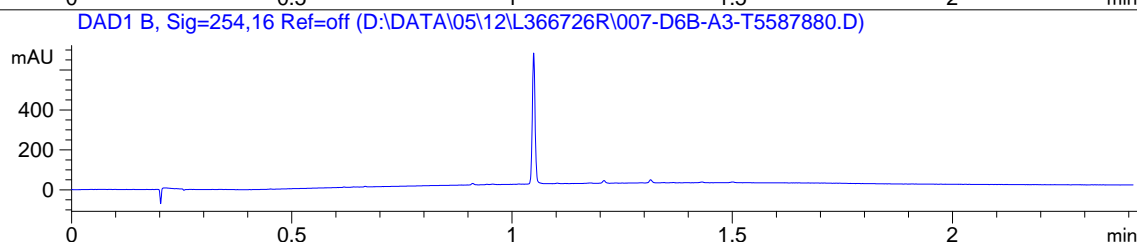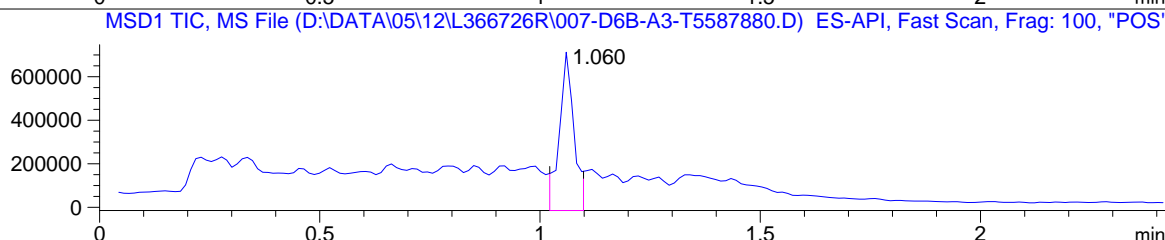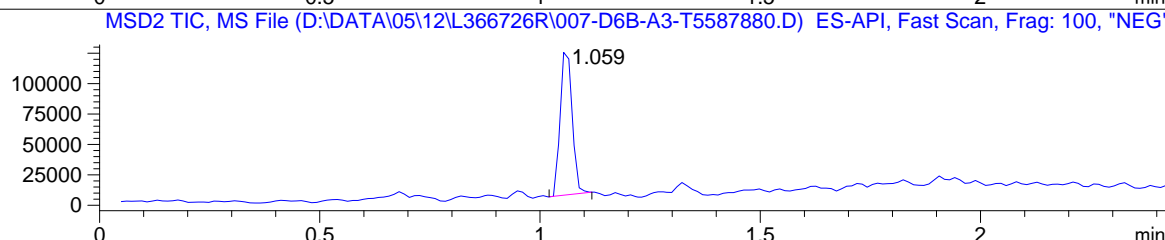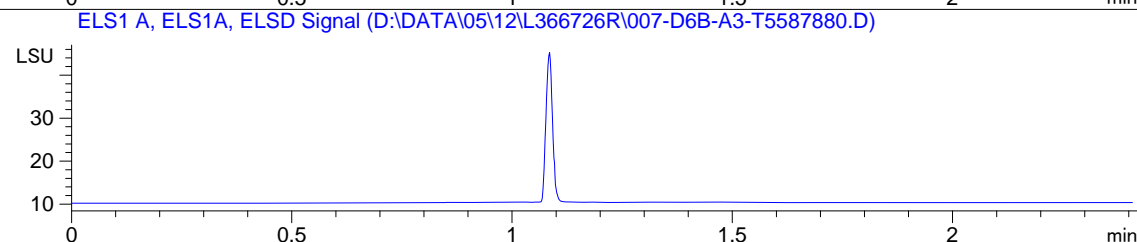

RT 1.060

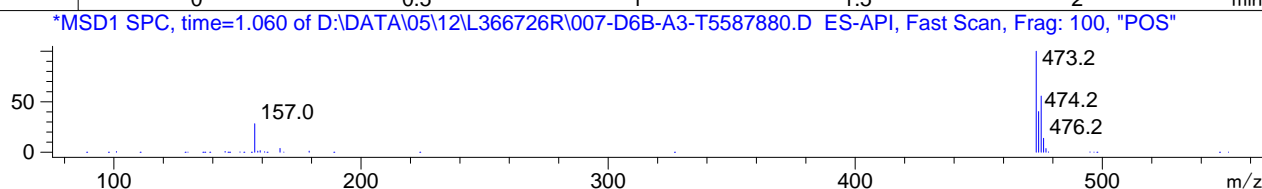

RT 1.059

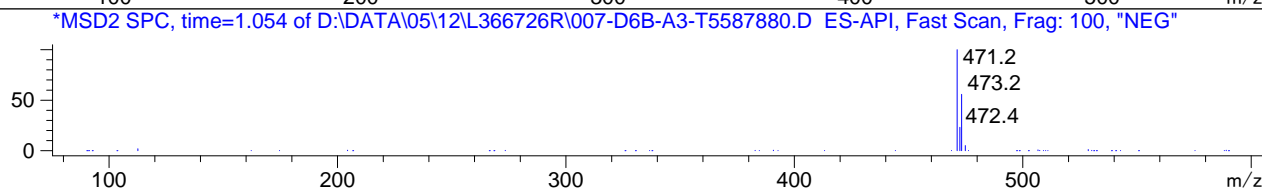

Supplement: Supplementary file 1 — Supplementary Information 1. [file 41598_2024_54655_MOESM1_ESM.zip › Nature SREP/QC_AIMS_files/Proj075.pdf]

T5572373

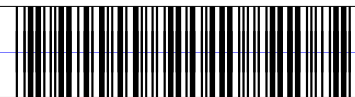

MaxPeak: 98.22%  
Ret\_Time: 1.283 min

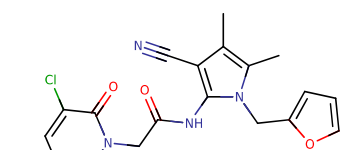

Mol Wt 452.81  
Exact Mass 452.1

| # | Time  | Area% |
|---|-------|-------|
| 1 | 1.283 | 98.22 |
| 2 | 1.378 | 1.78  |

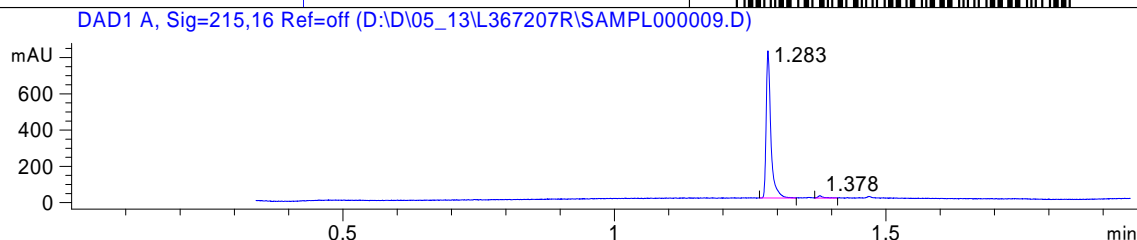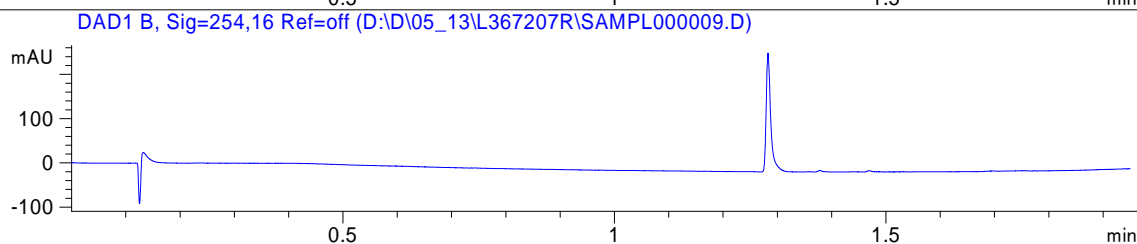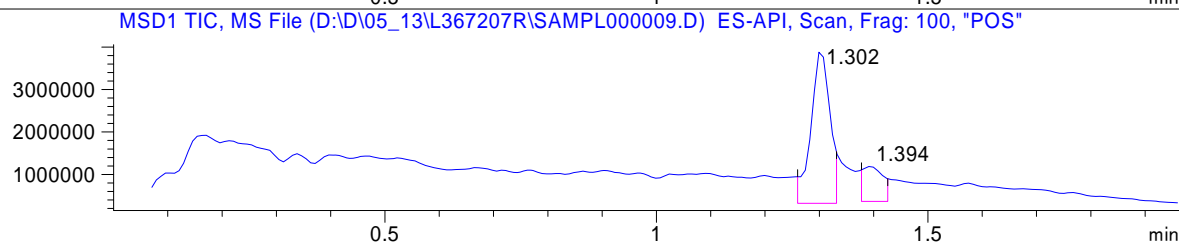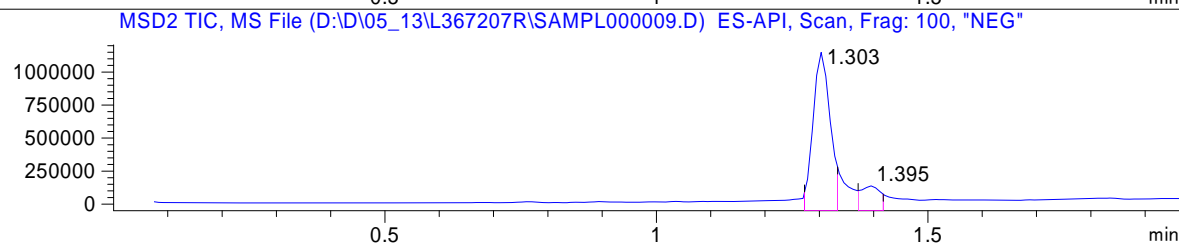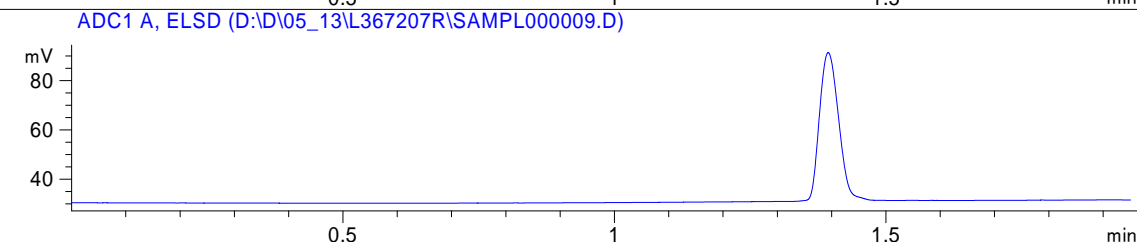

RT 1.302

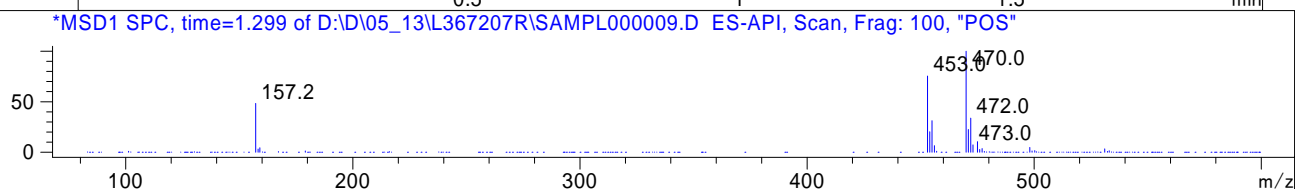

RT 1.394

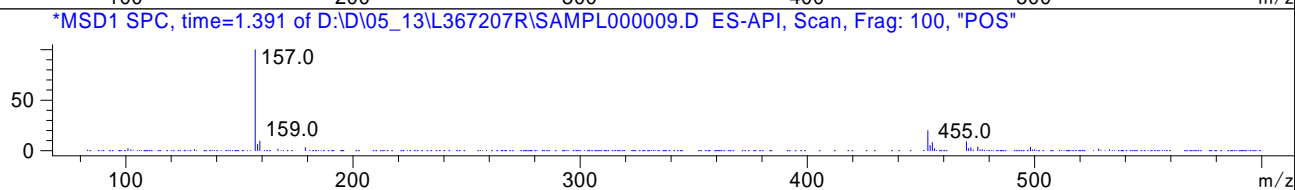

RT 1.303

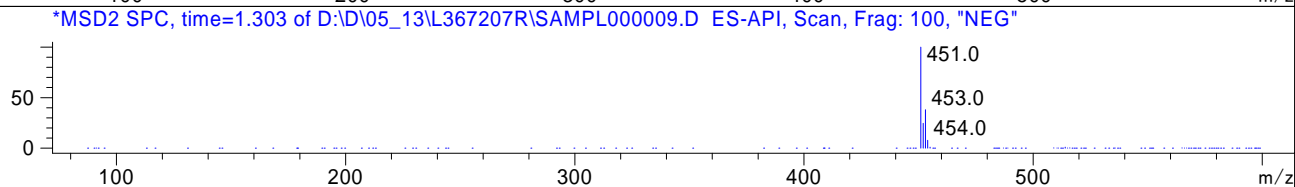

RT 1.395

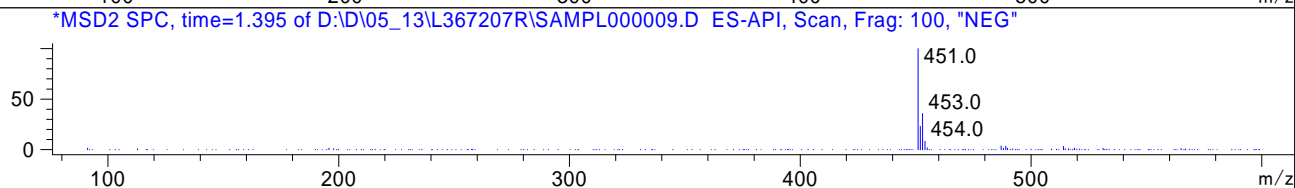

Supplement: Supplementary file 1 — Supplementary Information 1. [file 41598_2024_54655_MOESM1_ESM.zip › Nature SREP/QC_AIMS_files/Proj081.pdf]

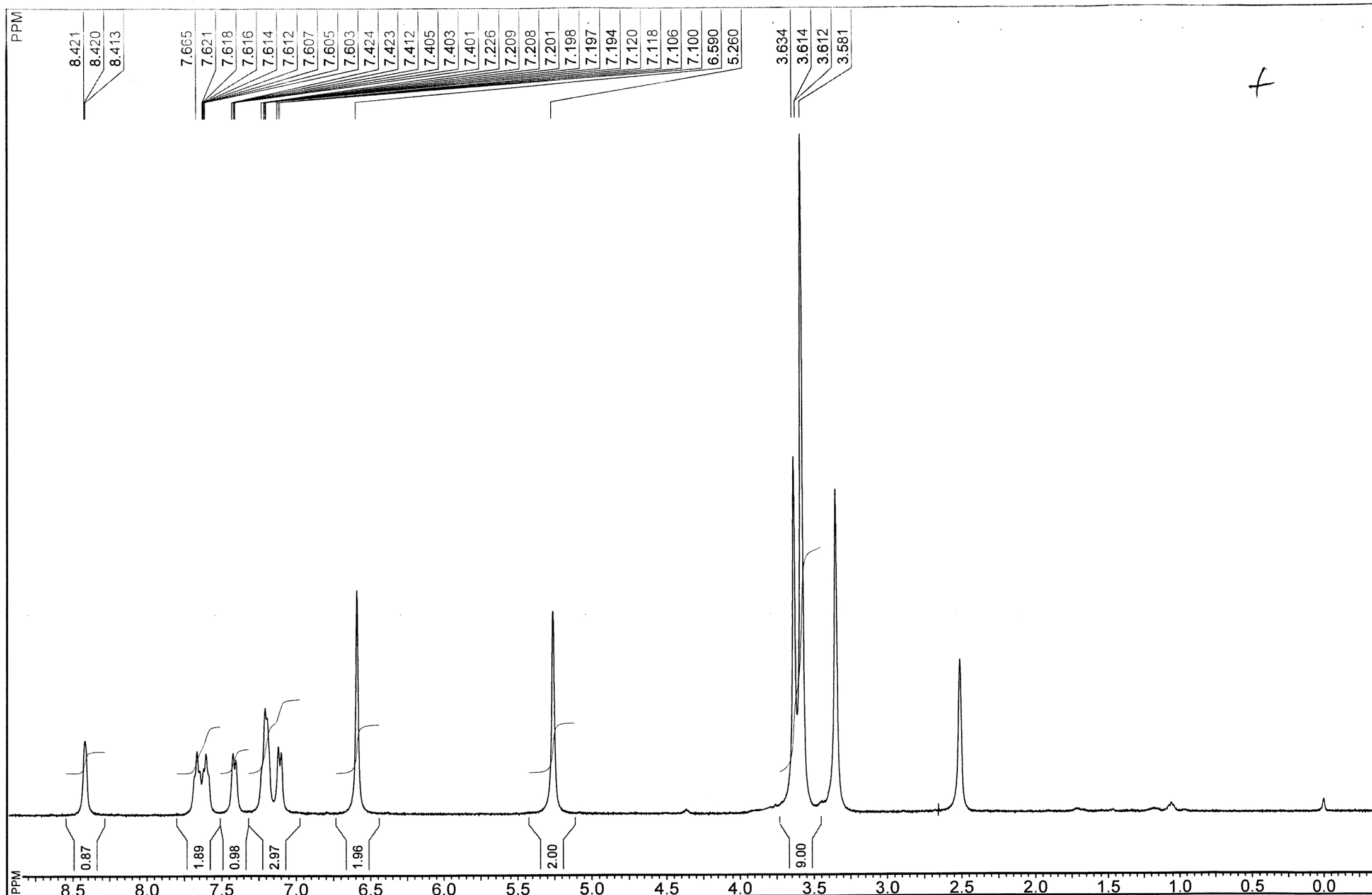

File name: 84039a

Owner:

SF: 400.4477 MHz

NS:

SI: 32768, TD: 24002

Date: 11-Feb-2007

Solvent: DMSO-d6

SW: 8000

TE: 293

Supplement: Supplementary file 1 — Supplementary Information 1. [file 41598_2024_54655_MOESM1_ESM.zip › Nature SREP/QC_AIMS_files/Proj091.pdf]

MaxPeak: 100.00%  
Ret\_Time: 1.133 min

2717133

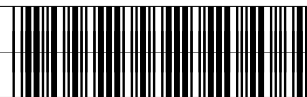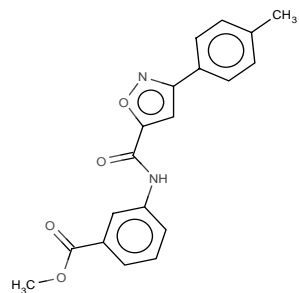

Mol Wt 336.341  
Exact Mass 336.12

| # | Time  | Area%  |
|---|-------|--------|
| 1 | 1.133 | 100.00 |

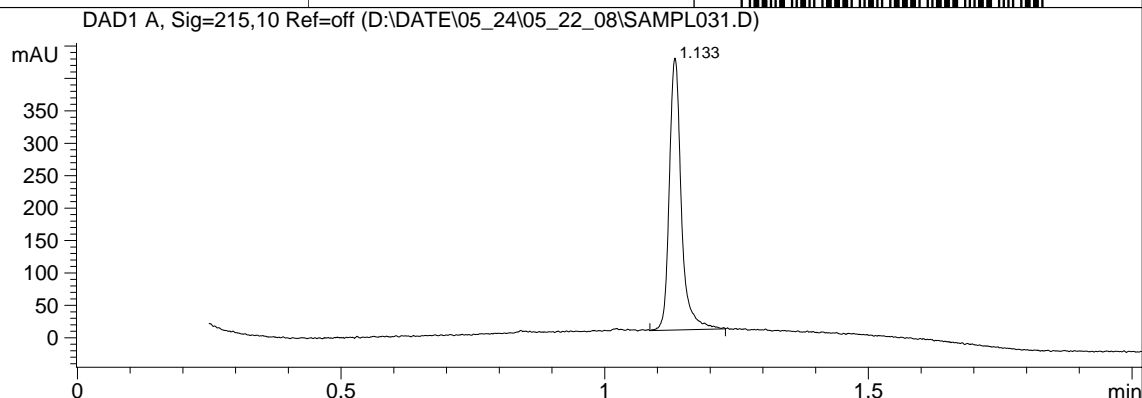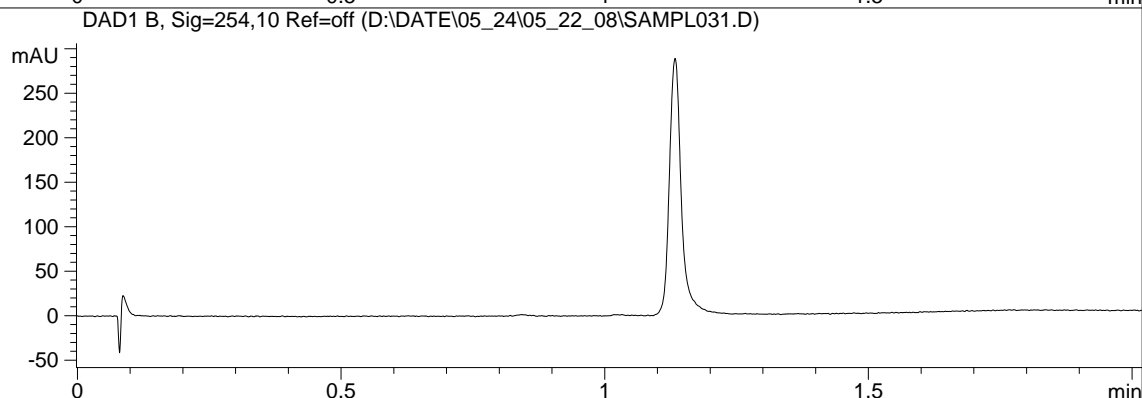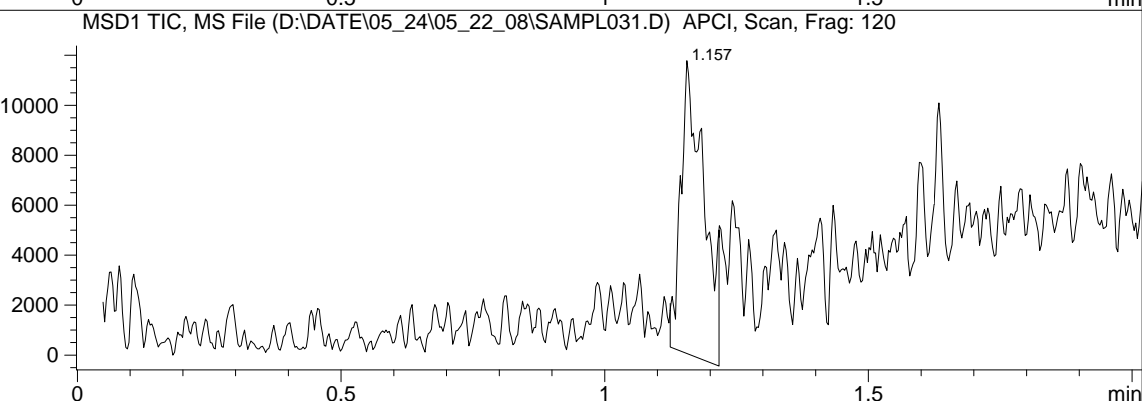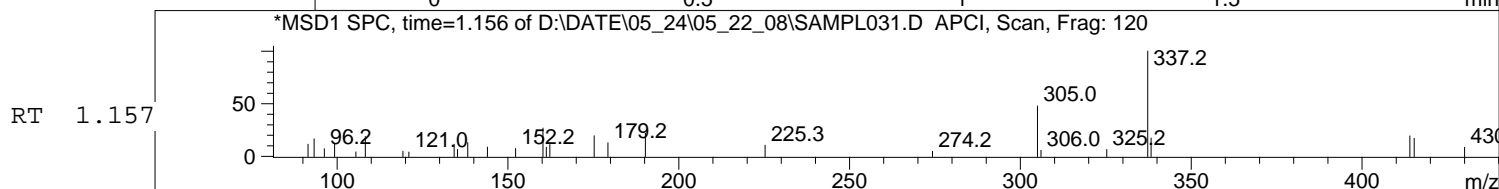

Supplement: Supplementary file 1 — Supplementary Information 1. [file 41598_2024_54655_MOESM1_ESM.zip › Nature SREP/QC_AIMS_files/Proj096.pdf]

$^1\text{H}$  NMR (400 MHz,  $\text{DMSO}-d_6$ )  $\delta$  ppm 10.10 (s, 1 H) 8.59 (d,  $J=5.14$  Hz, 1 H) 8.40 (s, 1 H) 8.29 - 8.38 (m, 2 H) 7.71 - 7.83 (m, 2 H) 7.16 (d,  $J=5.14$  Hz, 1 H) 7.00 (s, 2 H) 2.80 (d,  $J=6.85$  Hz, 2 H) 0.92 - 1.03 (m, 1 H) 0.28 - 0.40 (m, 2 H) -0.01 - 0.03 (m, 2 H)

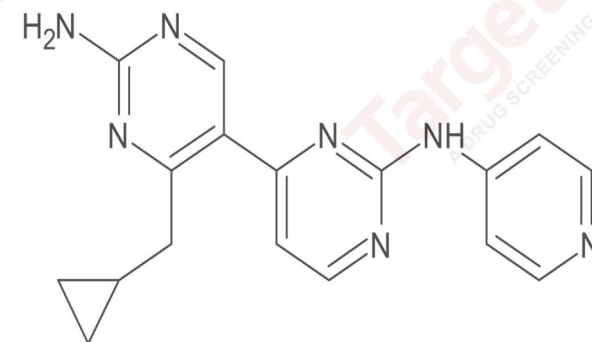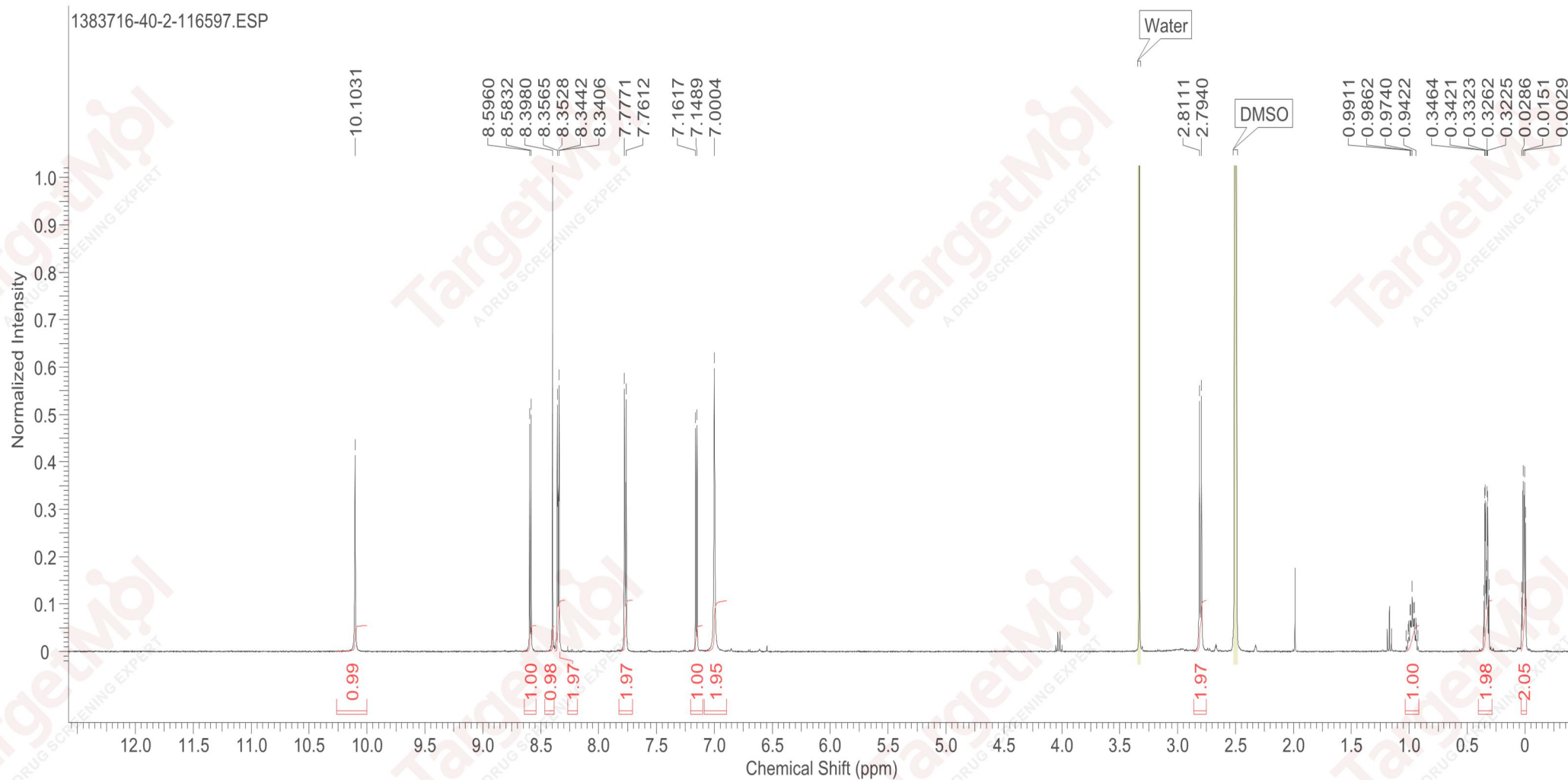

Supplement: Supplementary file 1 — Supplementary Information 1. [file 41598_2024_54655_MOESM1_ESM.zip › Nature SREP/QC_AIMS_files/Proj104.pdf]

MaxPeak: 98.65%  
Ret\_Time: 0.675 min

1818999

OK

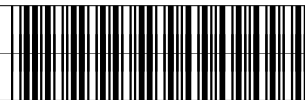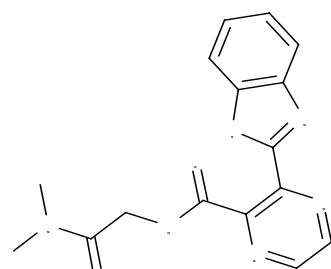

mw = 342.38

| # | Time  | Area% |
|---|-------|-------|
| 1 | 0.675 | 98.65 |
| 2 | 0.711 | 1.35  |

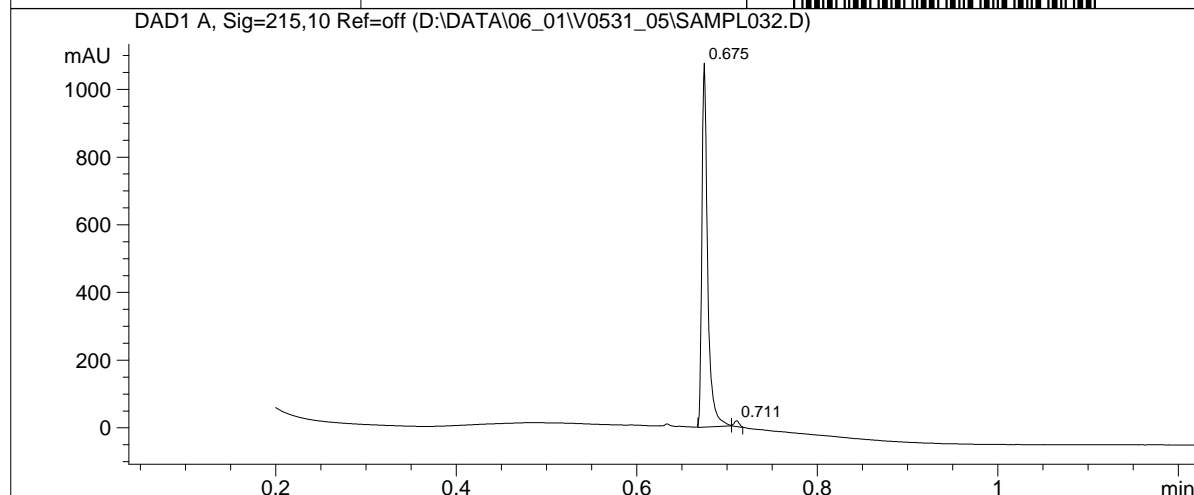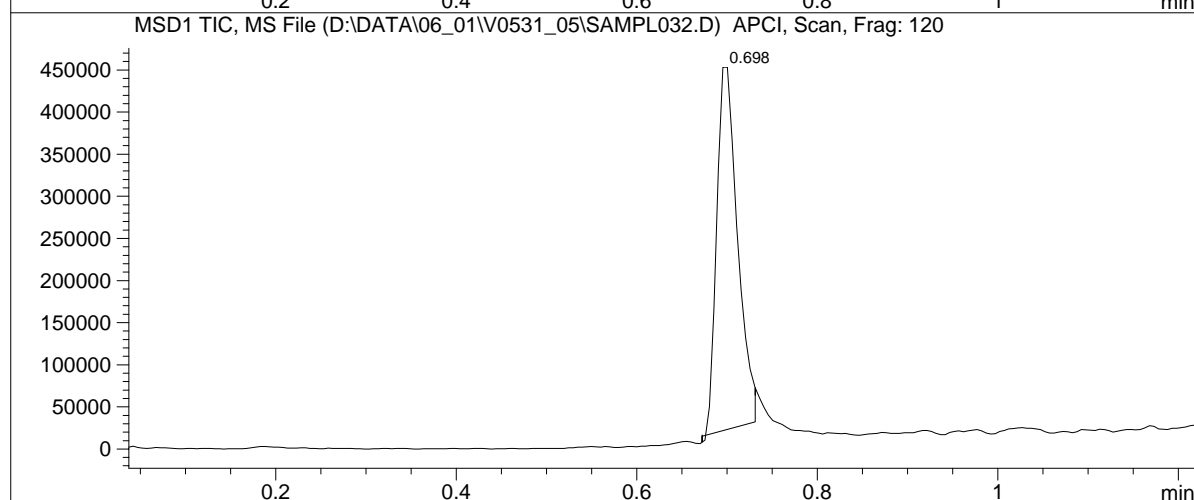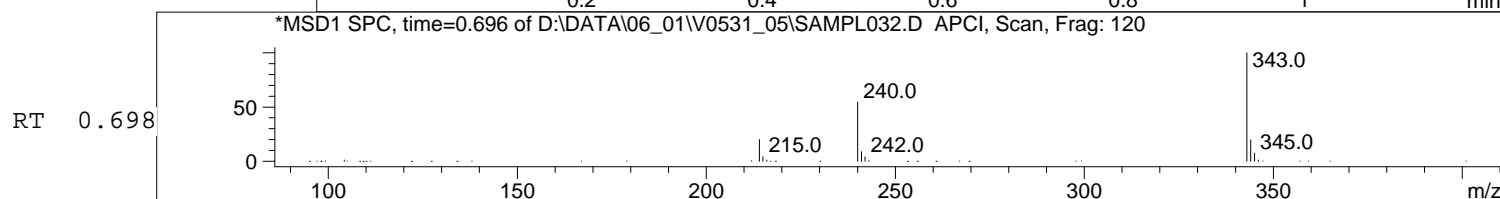

RT 0.698

Supplement: Supplementary file 1 — Supplementary Information 1. [file 41598_2024_54655_MOESM1_ESM.zip › Nature SREP/QC_AIMS_files/Proj120.pdf]

J975680\$1

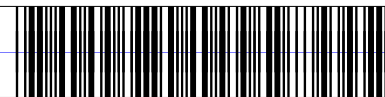

MaxPeak: 100.00%  
Ret\_Time: 1.119 min

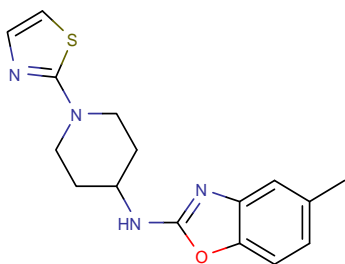

Mol Wt 314.4  
Exact Mass 314.14

| # | Time  | Area%  |
|---|-------|--------|
| 1 | 1.119 | 100.00 |

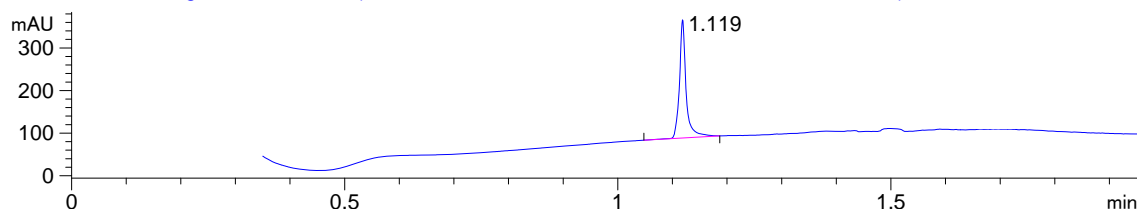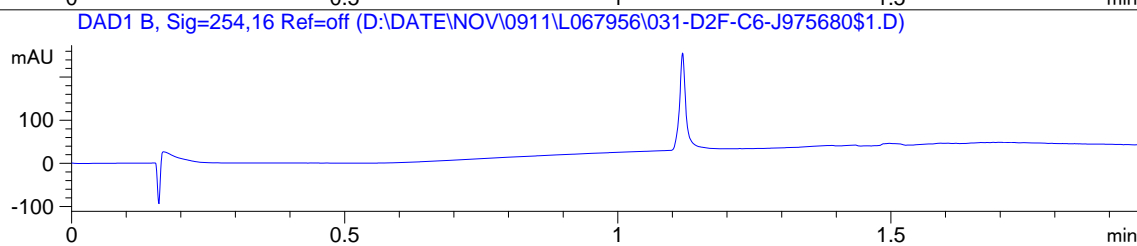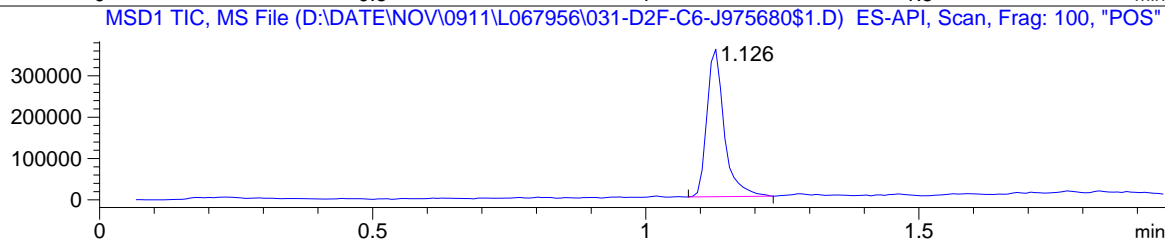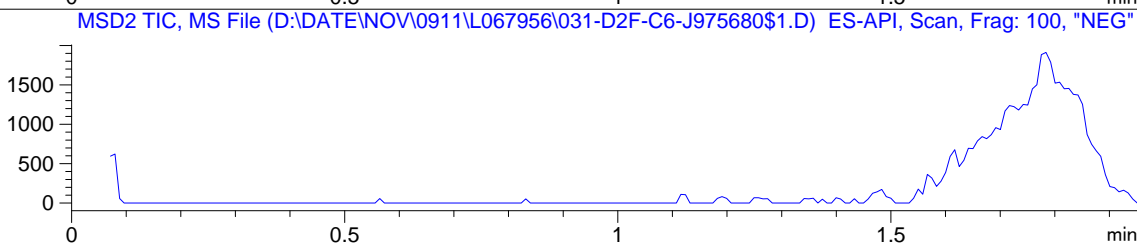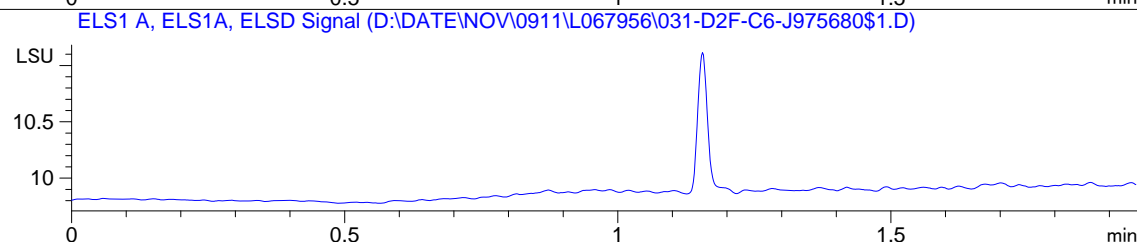

RT 1.126

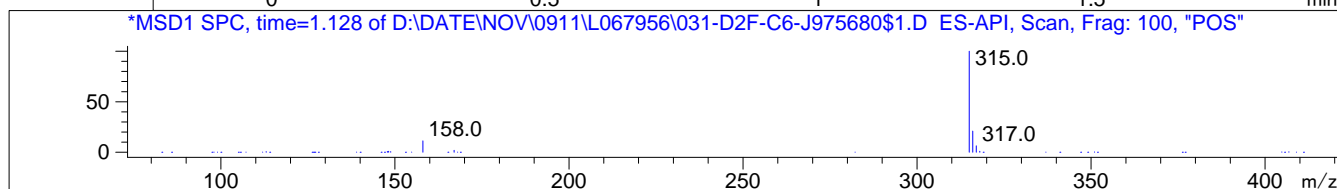

Supplement: Supplementary file 1 — Supplementary Information 1. [file 41598_2024_54655_MOESM1_ESM.zip › Nature SREP/QC_AIMS_files/Proj127.pdf]

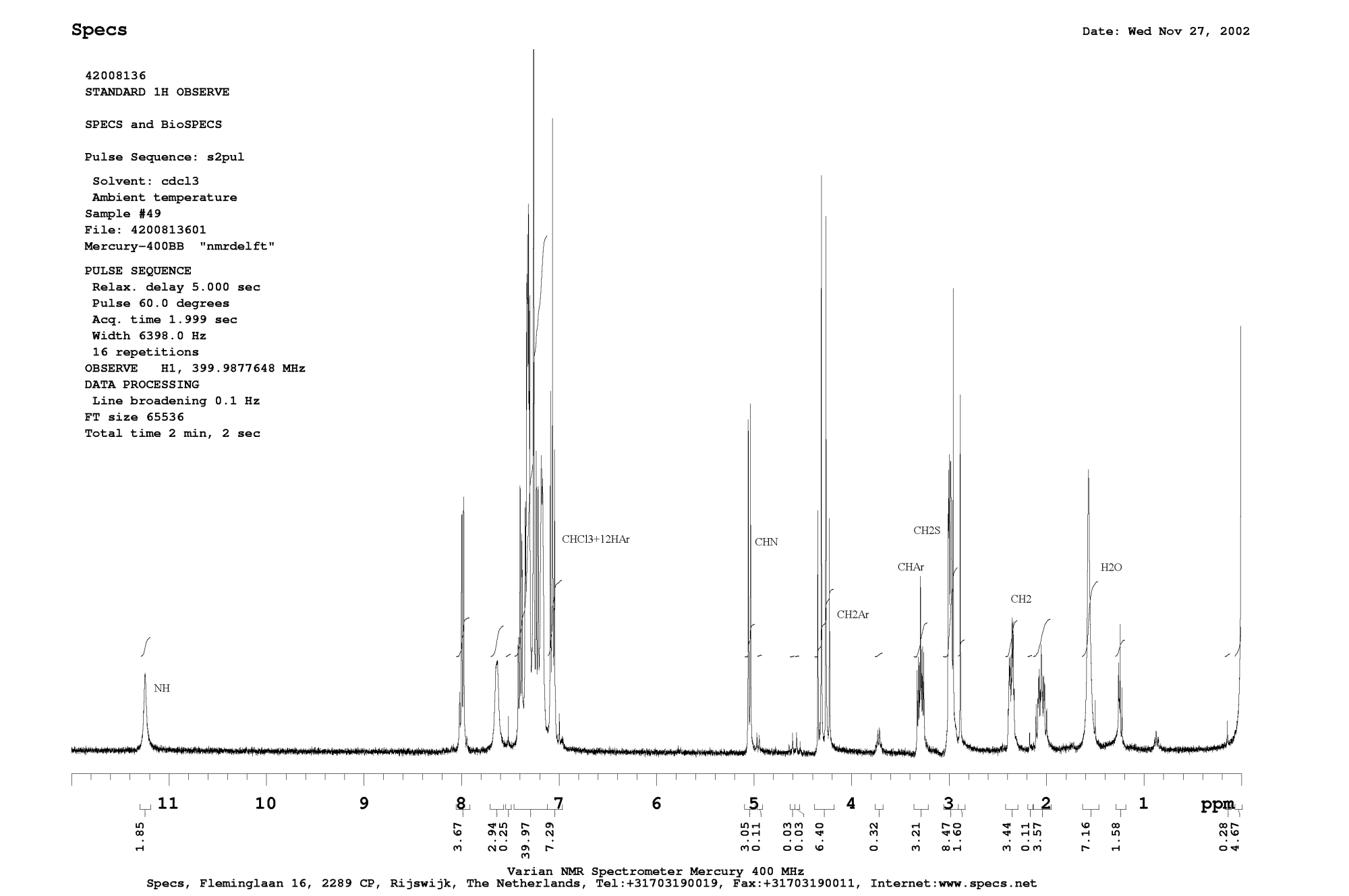

Supplement: Supplementary file 1 — Supplementary Information 1. [file 41598_2024_54655_MOESM1_ESM.zip › Nature SREP/QC_AIMS_files/Proj135.png]

T7371883

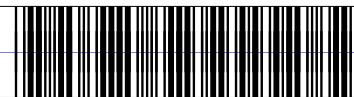

MaxPeak: 98.59%  
Ret\_Time: 1.530 min

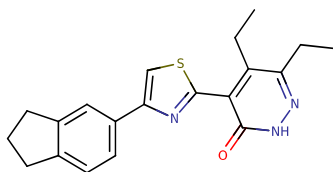

Mol Wt 351.46  
Exact Mass 351.17

| # | Time  | Area% |
|---|-------|-------|
| 1 | 1.223 | 1.41  |
| 2 | 1.530 | 98.59 |

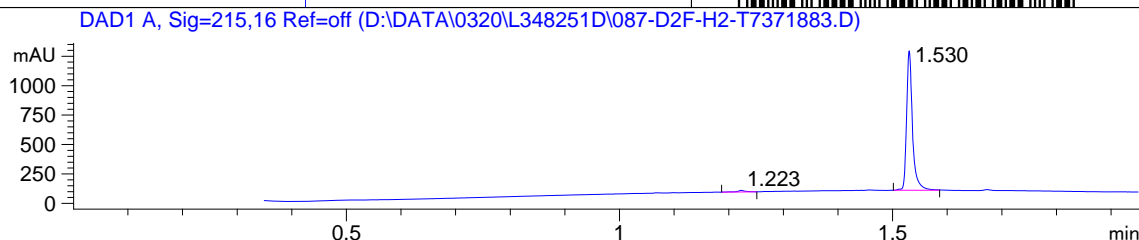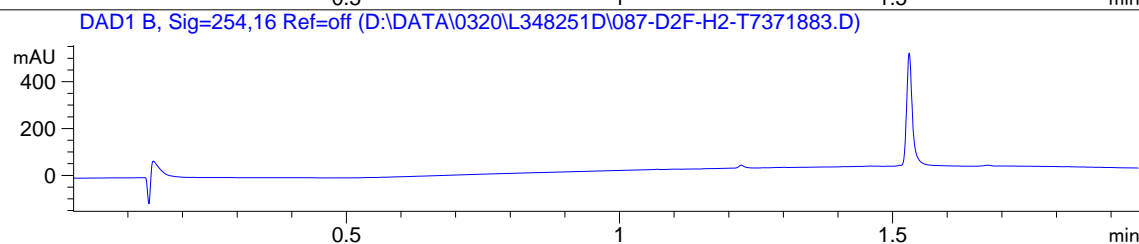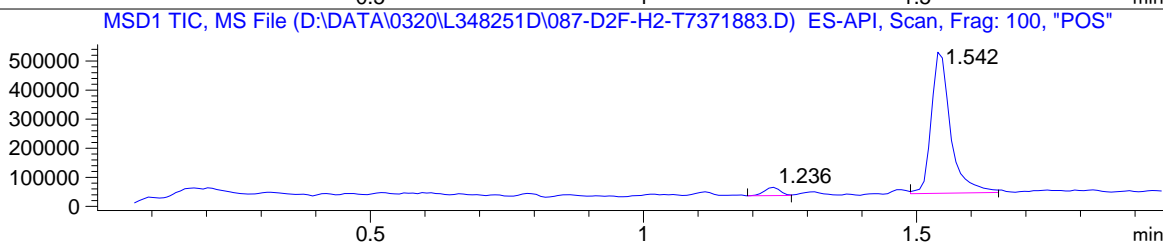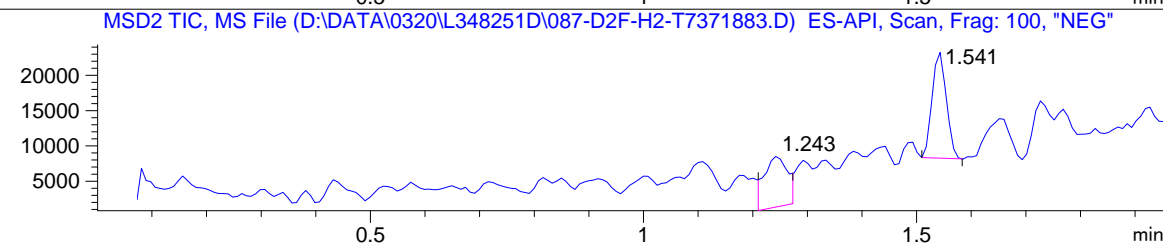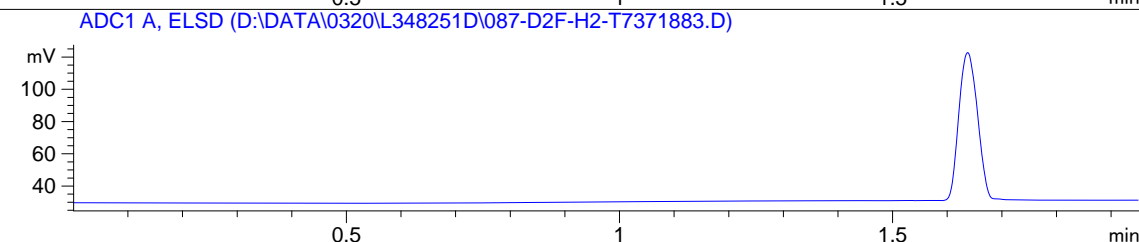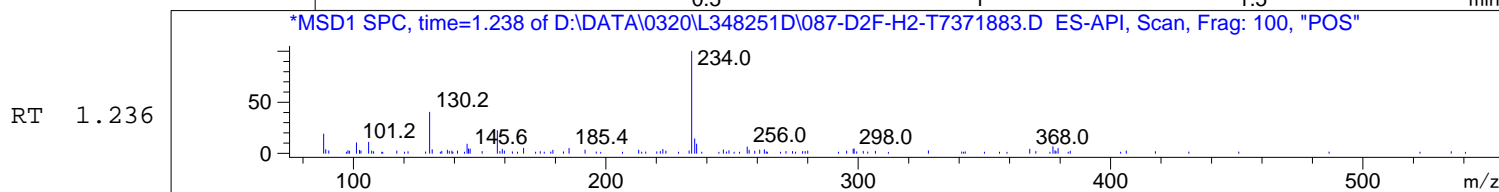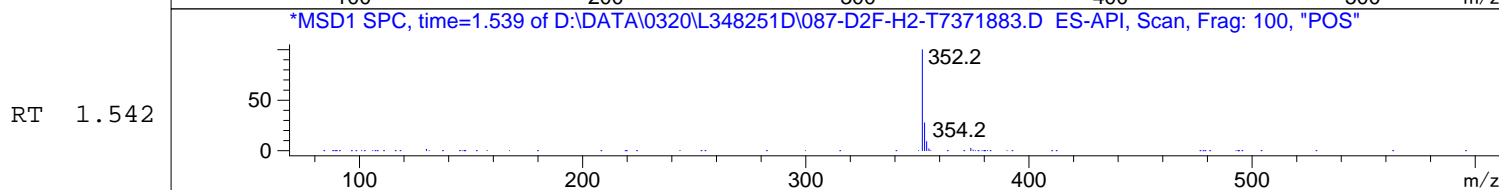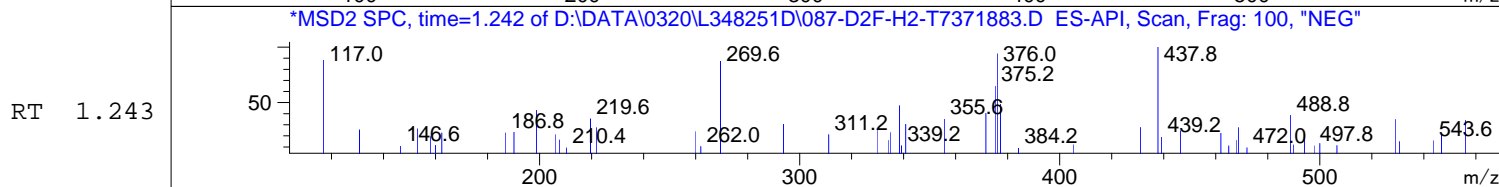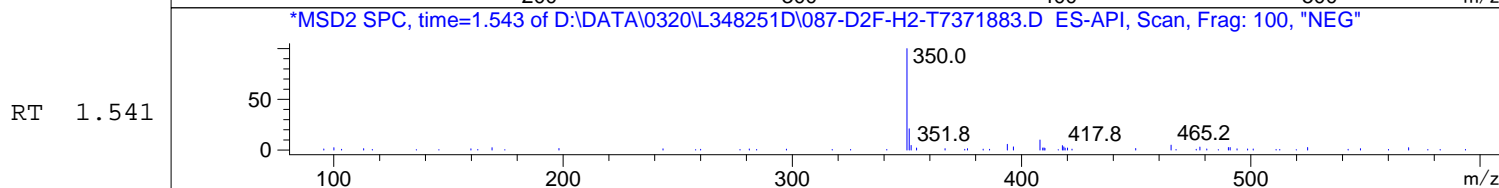

Supplement: Supplementary file 1 — Supplementary Information 1. [file 41598_2024_54655_MOESM1_ESM.zip › Nature SREP/QC_AIMS_files/Proj148.pdf]

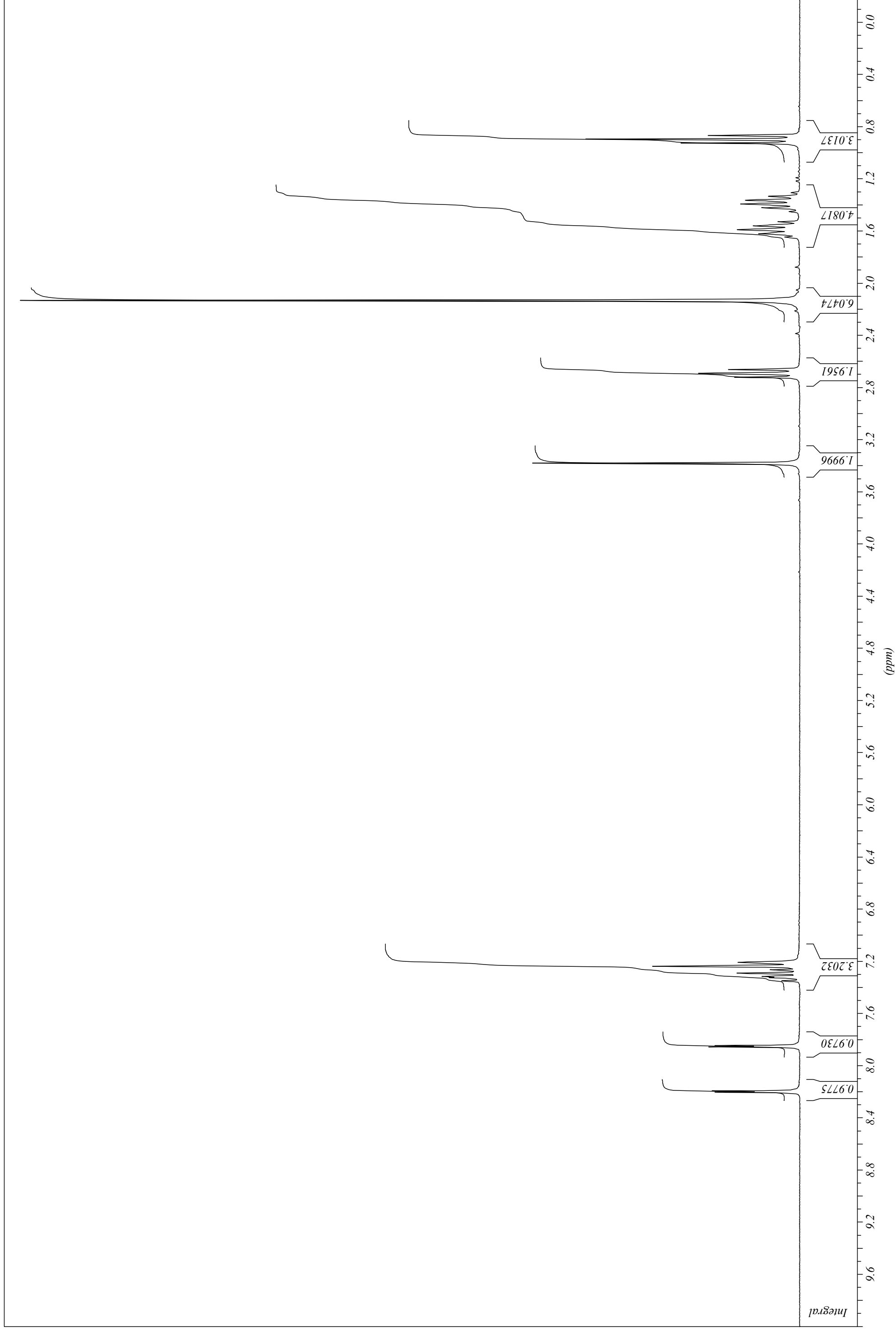

Supplement: Supplementary file 1 — Supplementary Information 1. [file 41598_2024_54655_MOESM1_ESM.zip › Nature SREP/QC_AIMS_files/Proj152.pdf]

MaxPeak: 97.25%  
Ret\_Time: 1.081 min

T7370122

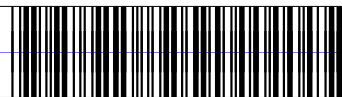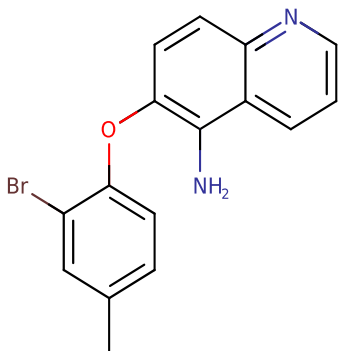

Mol Wt 329.19  
Exact Mass 328.04

| # | Time  | Area% |
|---|-------|-------|
| 1 | 0.973 | 2.75  |
| 2 | 1.081 | 97.25 |

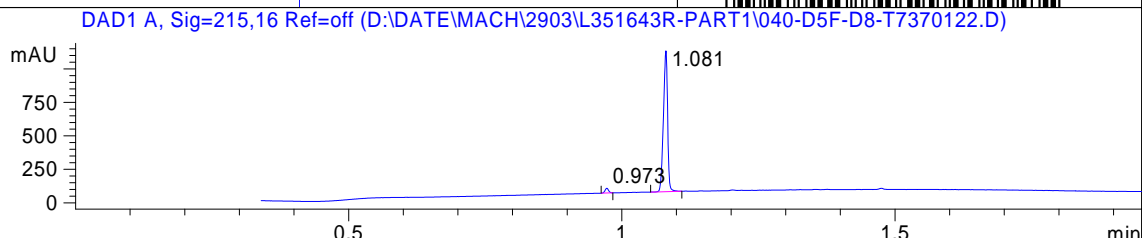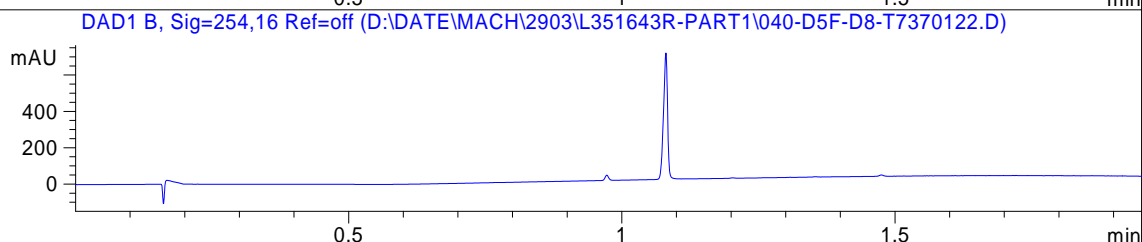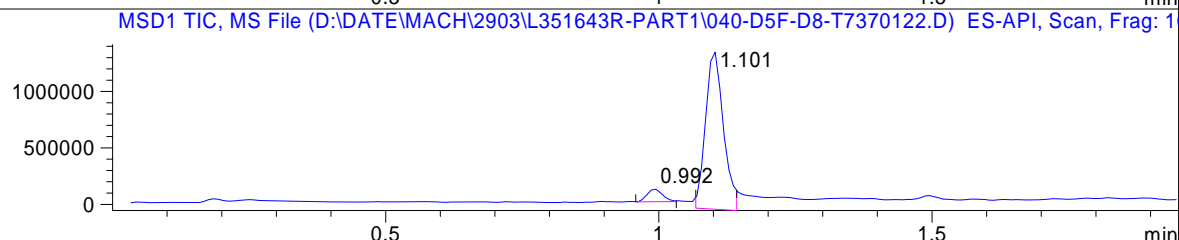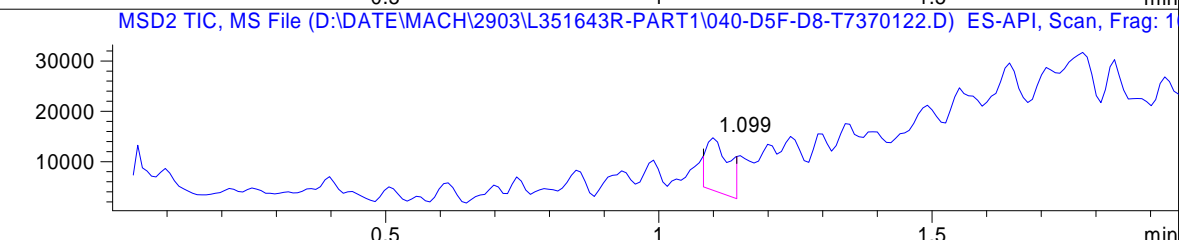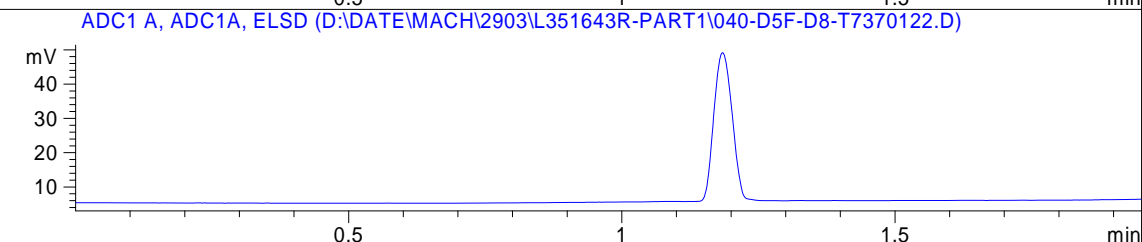

RT 0.992

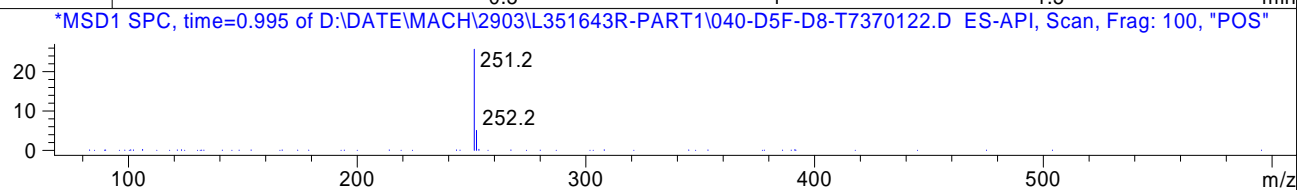

RT 1.101

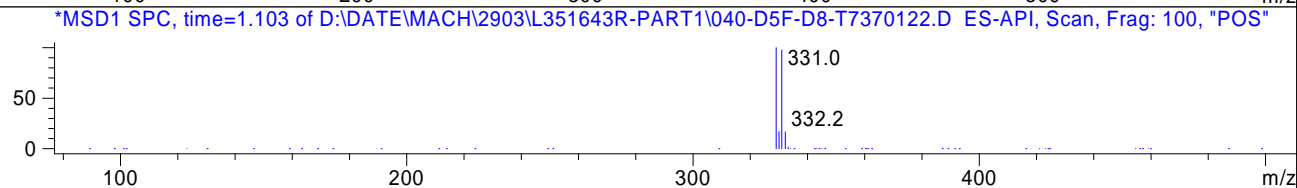

RT 1.099

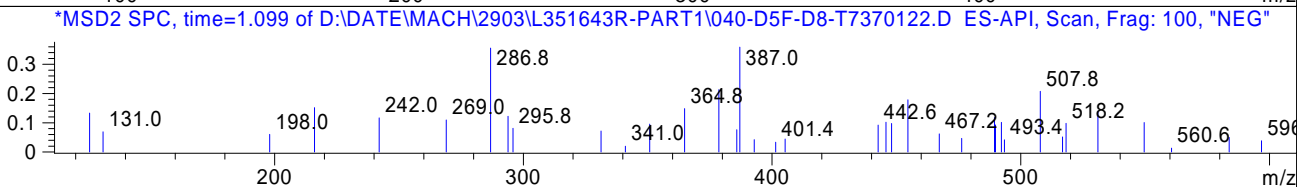

Supplement: Supplementary file 1 — Supplementary Information 1. [file 41598_2024_54655_MOESM1_ESM.zip › Nature SREP/QC_AIMS_files/Proj154.pdf]

T7676181

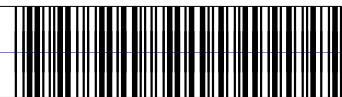

MaxPeak: 100.00%  
Ret\_Time: 1.384 min

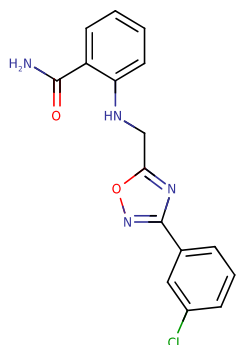

Mol Wt 328.75  
Exact Mass 328.08

| # | Time  | Area%  |
|---|-------|--------|
| 1 | 1.384 | 100.00 |

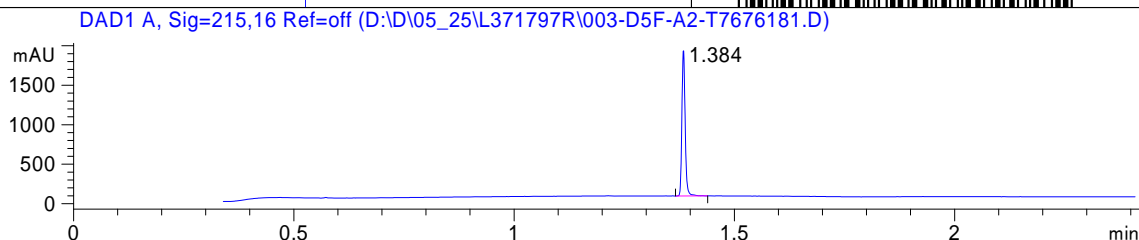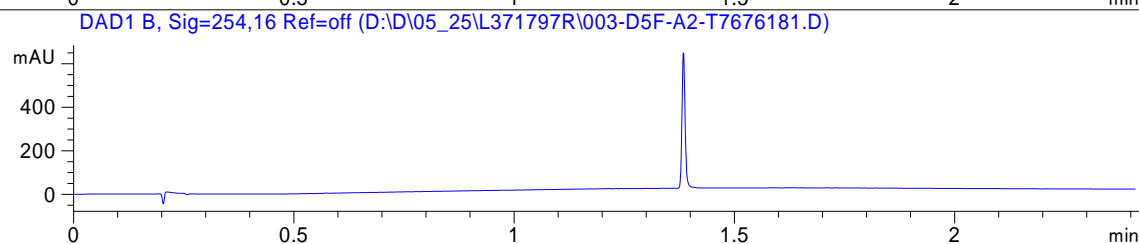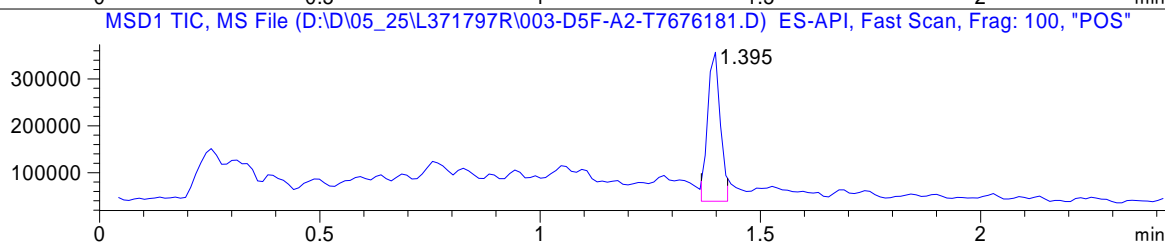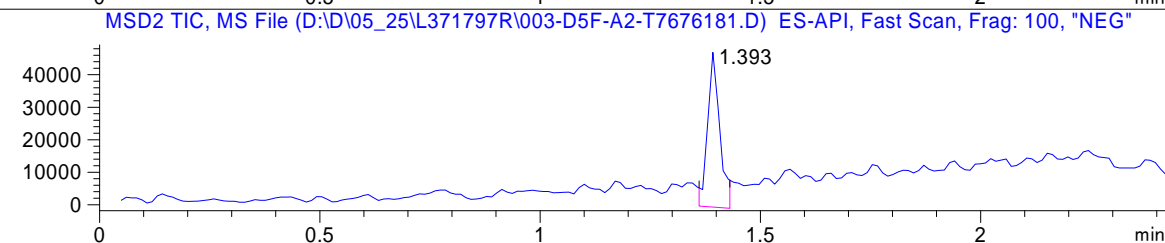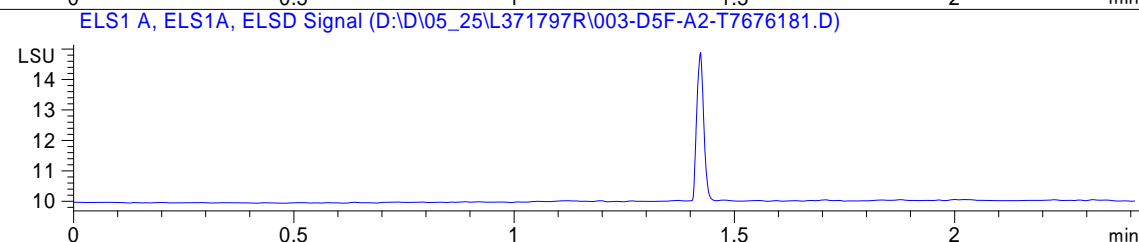

RT 1.395

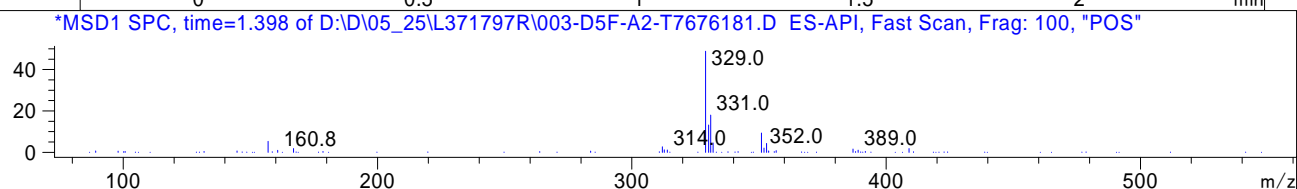

RT 1.393

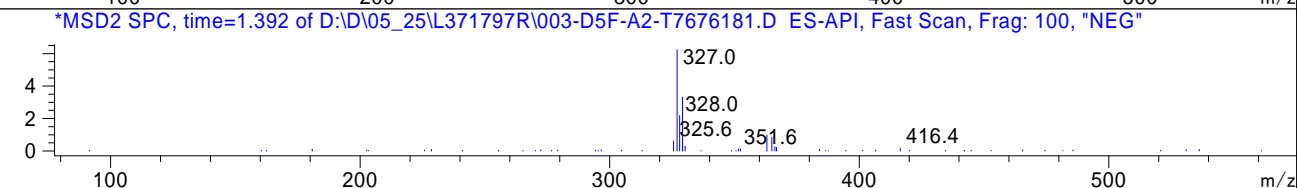

Supplement: Supplementary file 1 — Supplementary Information 1. [file 41598_2024_54655_MOESM1_ESM.zip › Nature SREP/QC_AIMS_files/Proj155.pdf]

MaxPeak: 100.00%  
Ret\_Time: 1.522 min

T5571835

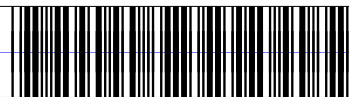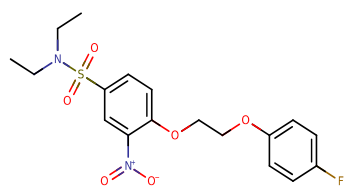

Mol Wt 412.43  
Exact Mass 412.12

| # | Time  | Area%  |
|---|-------|--------|
| 1 | 1.522 | 100.00 |

DAD1 A, Sig=215,16 Ref=off (D:\WORK\D\06\06\_09\L378257R\008-D5F-A7-T5571835.D)

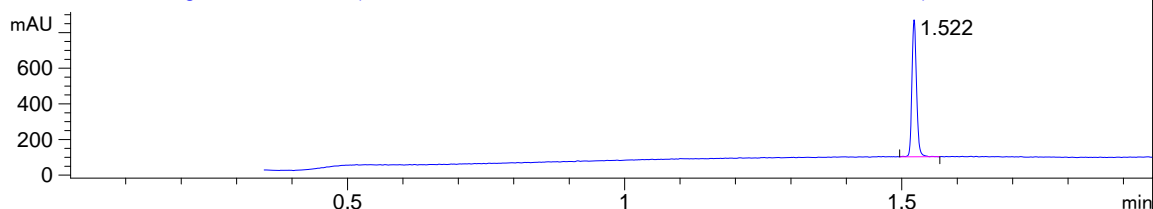

Supplement: Supplementary file 1 — Supplementary Information 1. [file 41598_2024_54655_MOESM1_ESM.zip › Nature SREP/QC_AIMS_files/Proj157.pdf]

T5767508

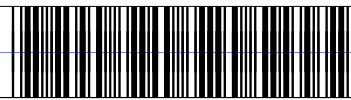

MaxPeak: 100.00%  
Ret\_Time: 1.631 min

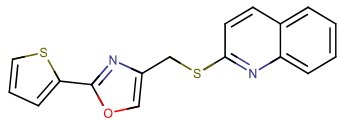

Mol Wt 324.42  
Exact Mass 324.05

| # | Time  | Area%  |
|---|-------|--------|
| 1 | 1.631 | 100.00 |

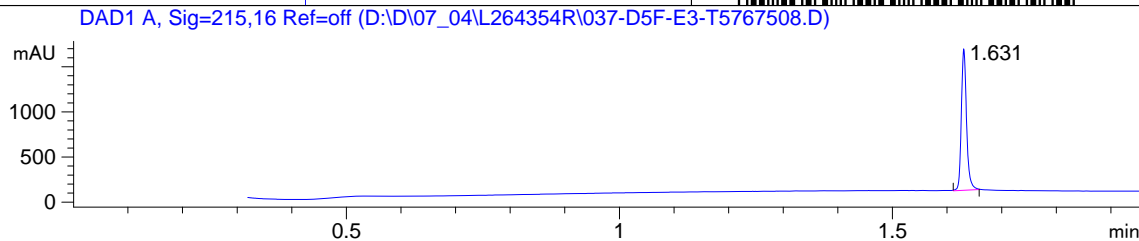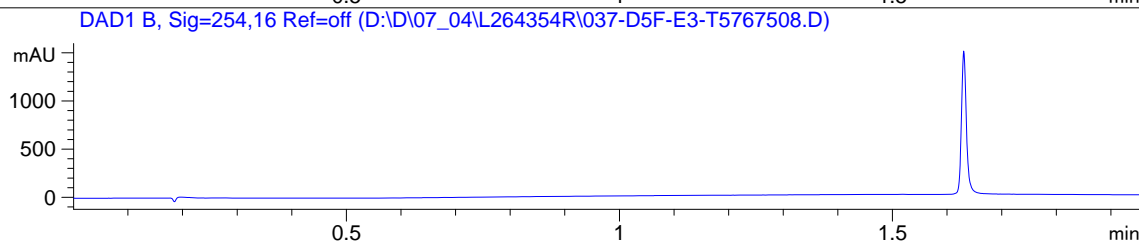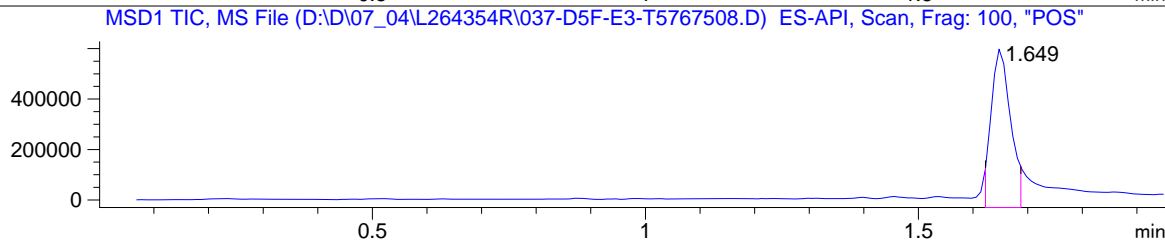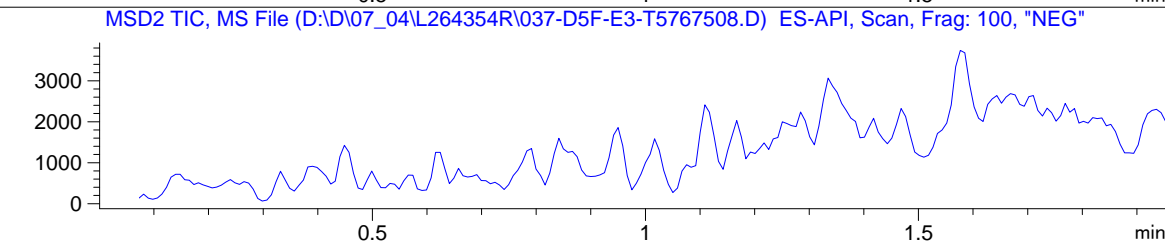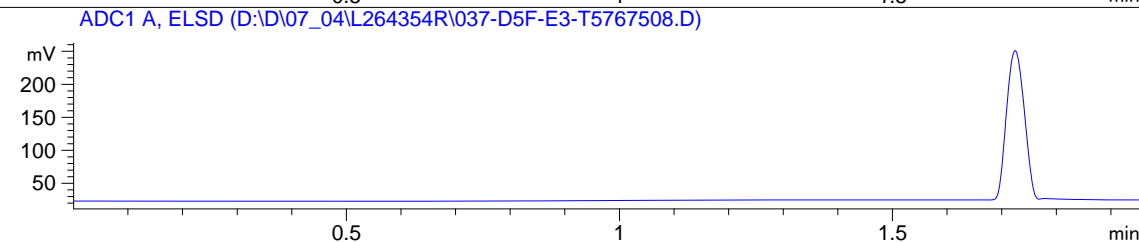

RT 1.649

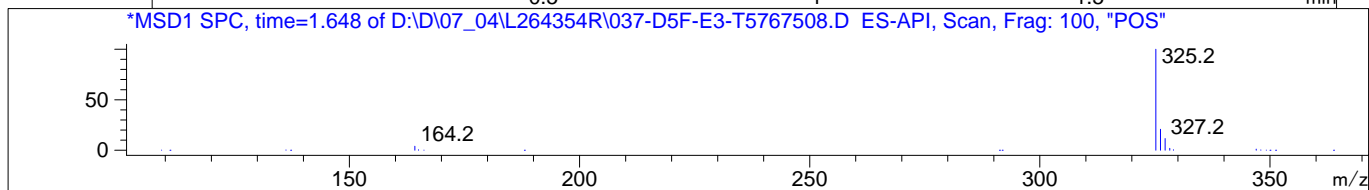

Supplement: Supplementary file 1 — Supplementary Information 1. [file 41598_2024_54655_MOESM1_ESM.zip › Nature SREP/QC_AIMS_files/Proj158.pdf]

T6125178

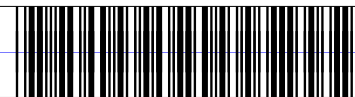

MaxPeak: 95.03%  
Ret\_Time: 1.449 min

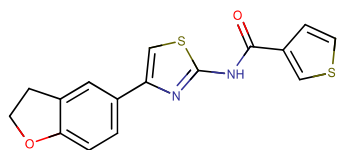

Mol Wt 328.41  
Exact Mass 328.04

| # | Time  | Area% |
|---|-------|-------|
| 1 | 1.061 | 2.39  |
| 2 | 1.449 | 95.03 |
| 3 | 1.508 | 2.59  |

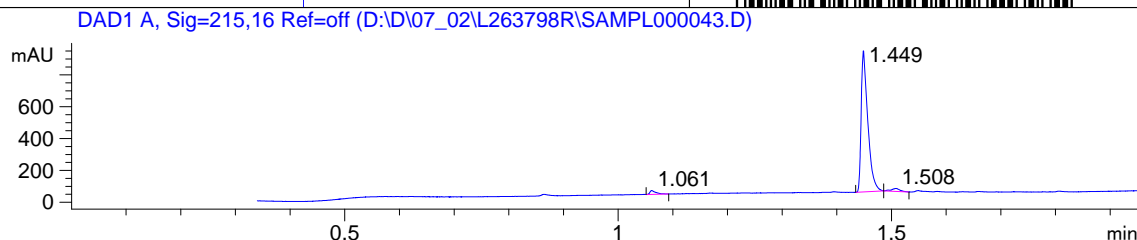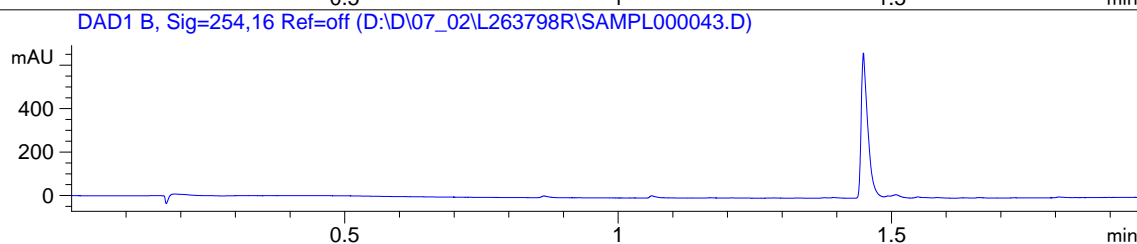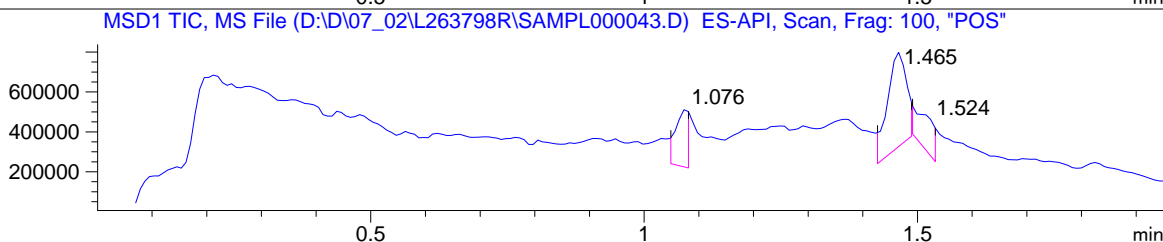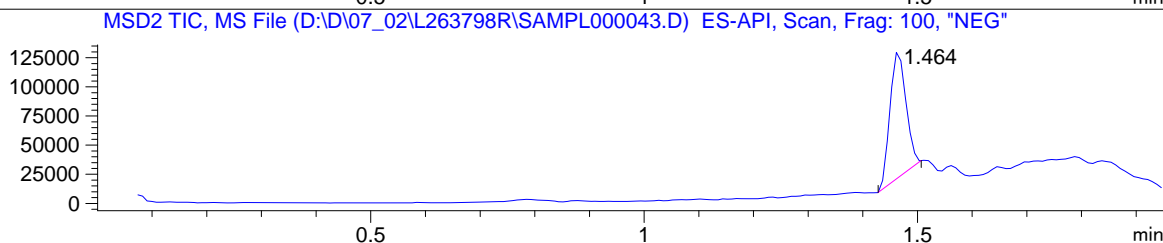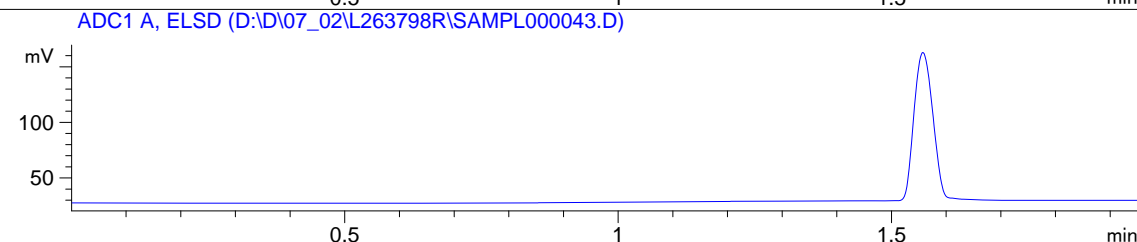

RT 1.076

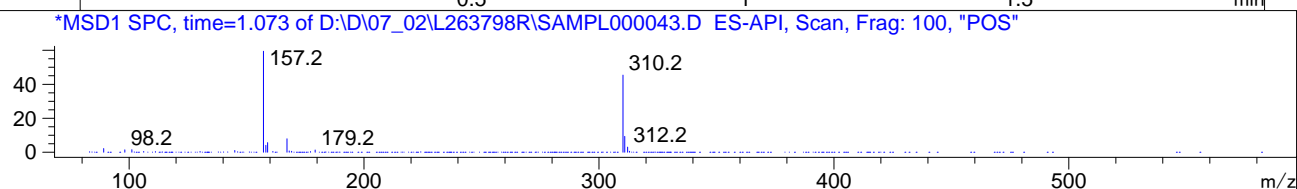

RT 1.465

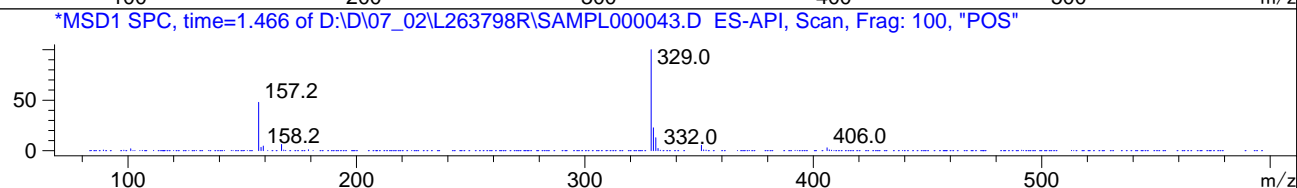

RT 1.524

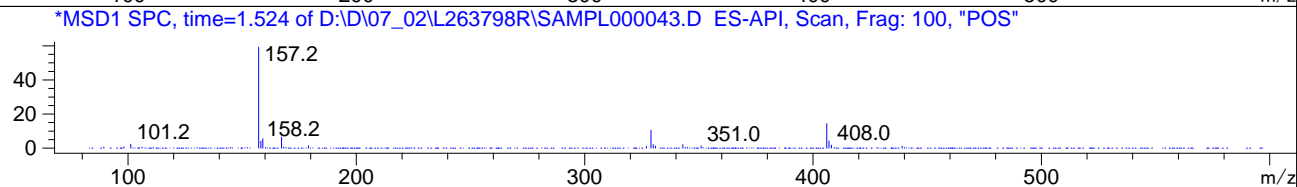

RT 1.464

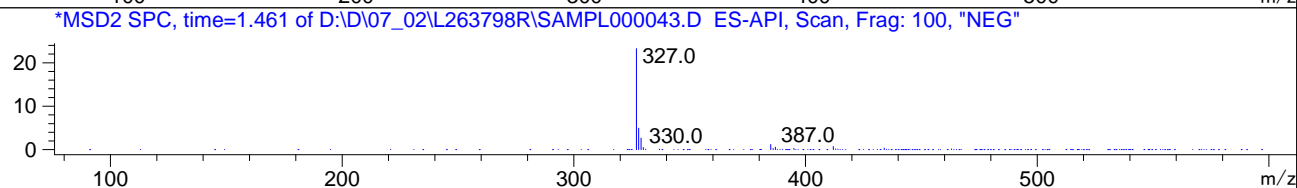

Supplement: Supplementary file 1 — Supplementary Information 1. [file 41598_2024_54655_MOESM1_ESM.zip › Nature SREP/QC_AIMS_files/Proj159.pdf]

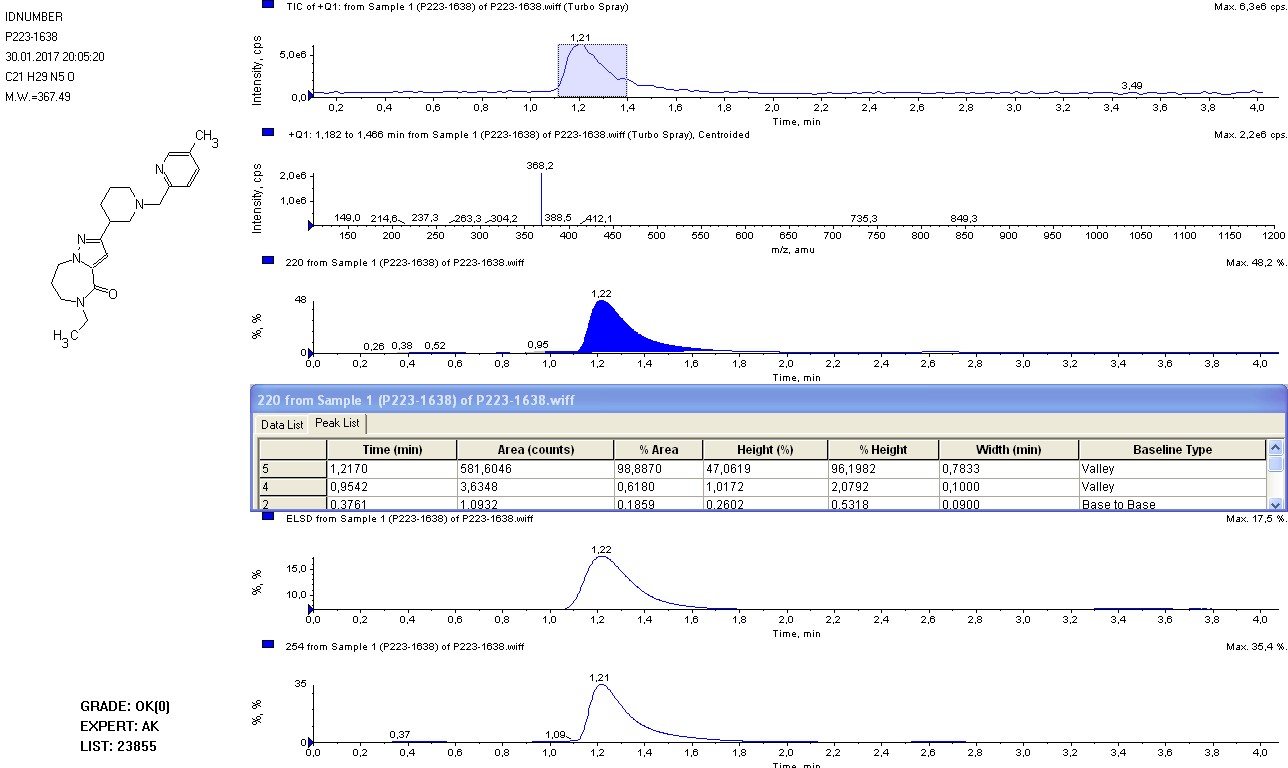

Supplement: Supplementary file 1 — Supplementary Information 1. [file 41598_2024_54655_MOESM1_ESM.zip › Nature SREP/QC_AIMS_files/Proj166.JPG]

MaxPeak: 92.60%  
Ret\_Time: 1.393 min

7508215

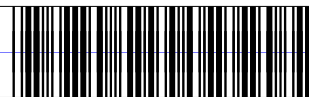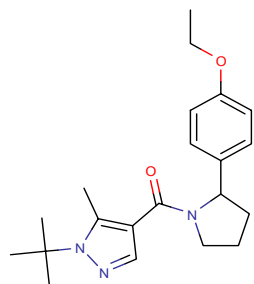

Mol Wt 355.47  
Exact Mass 355.27

| # | Time  | Area% |
|---|-------|-------|
| 1 | 0.520 | 2.54  |
| 2 | 1.023 | 2.29  |
| 3 | 1.357 | 2.56  |
| 4 | 1.393 | 92.60 |

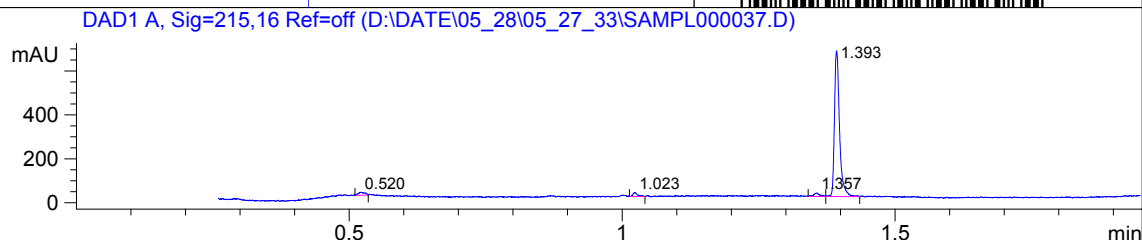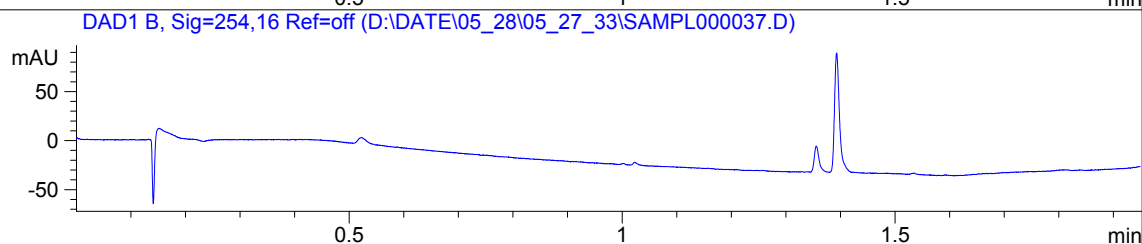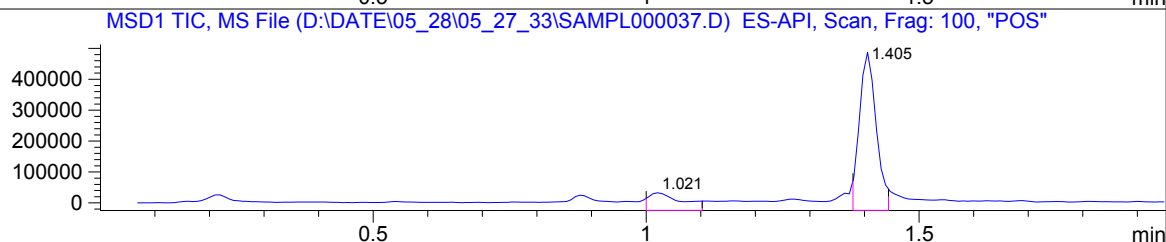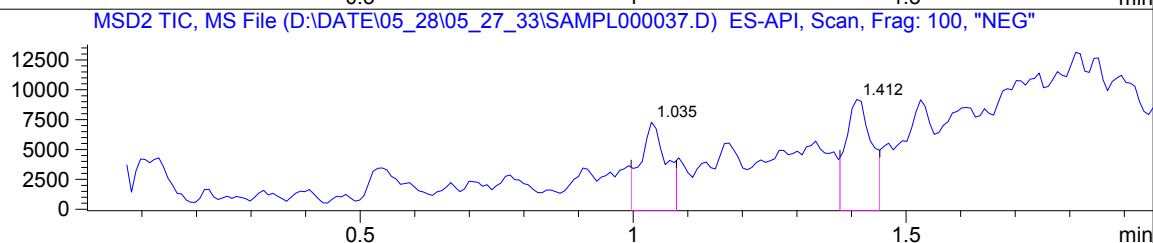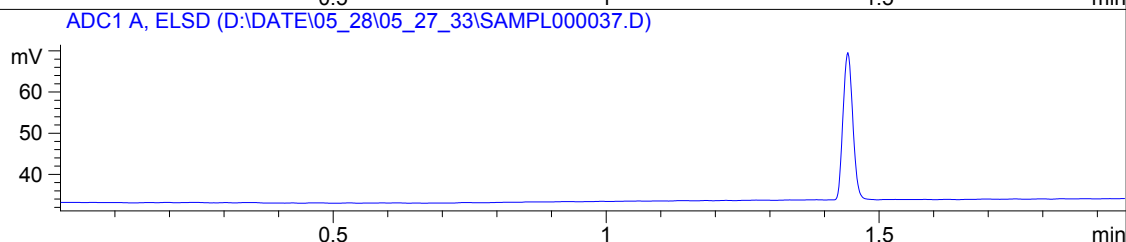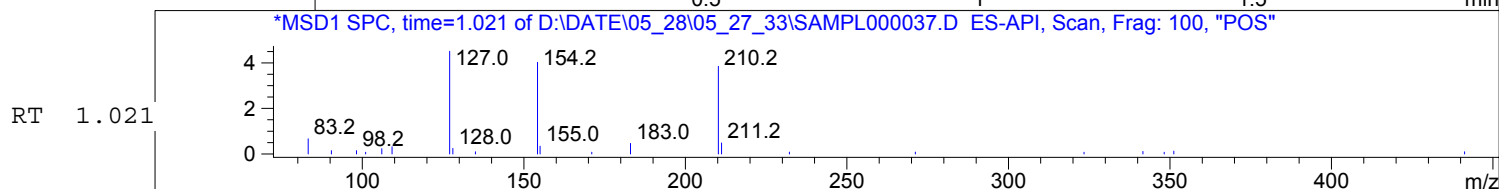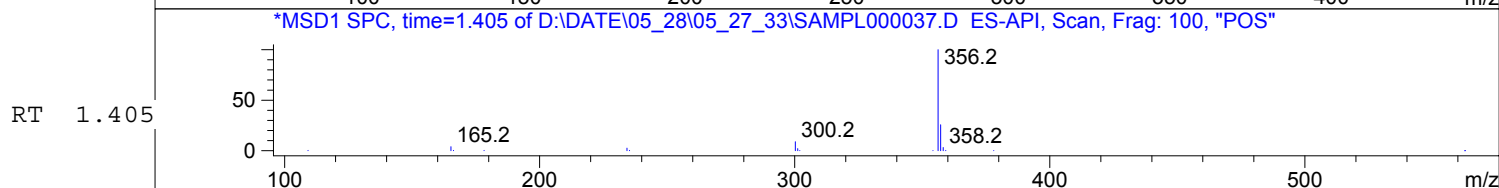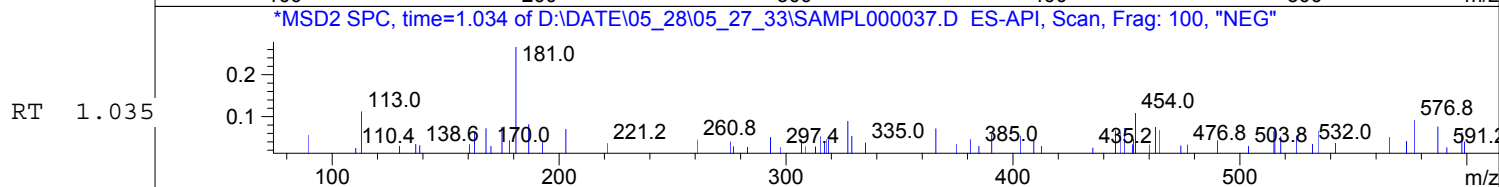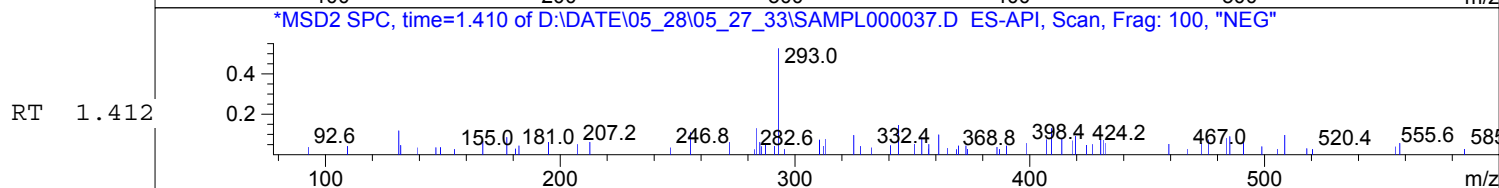

Supplement: Supplementary file 1 — Supplementary Information 1. [file 41598_2024_54655_MOESM1_ESM.zip › Nature SREP/QC_AIMS_files/Proj167.pdf]

A2195/0092340

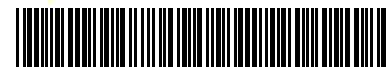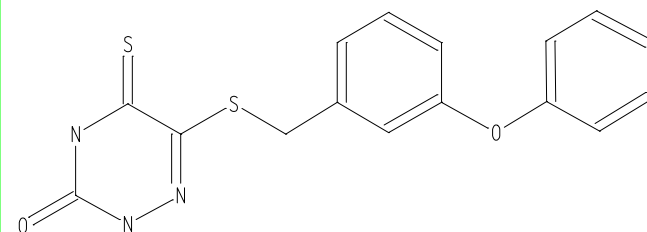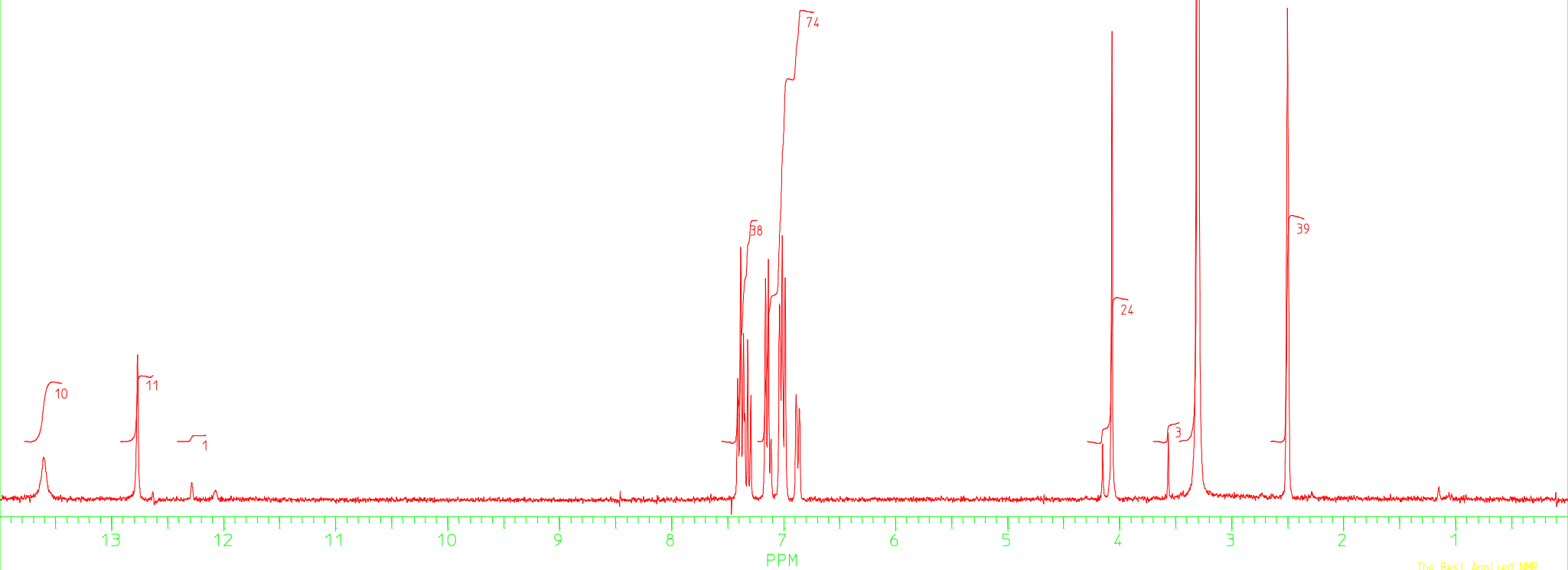

Supplement: Supplementary file 1 — Supplementary Information 1. [file 41598_2024_54655_MOESM1_ESM.zip › Nature SREP/QC_AIMS_files/Proj182.pdf]

MaxPeak: 100.00%  
Ret\_Time: 0.806 min

T8189223

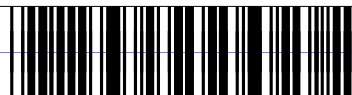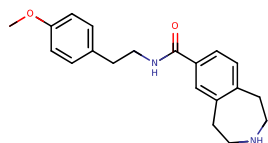

Mol Wt 360.88  
Exact Mass 324.22

| # | Time  | Area%  |
|---|-------|--------|
| 1 | 0.806 | 100.00 |

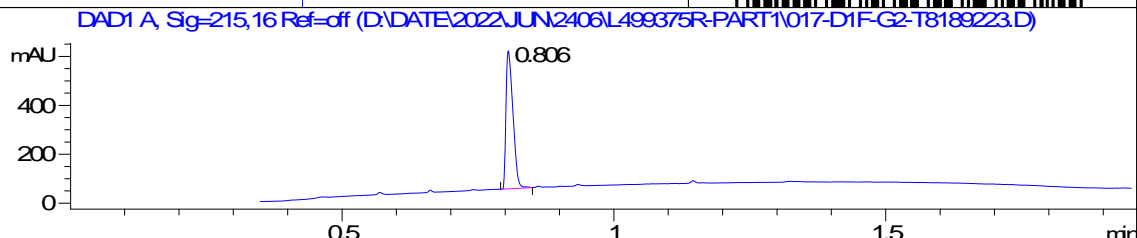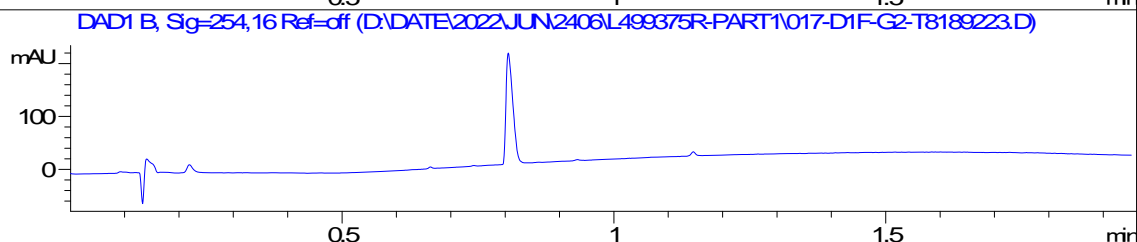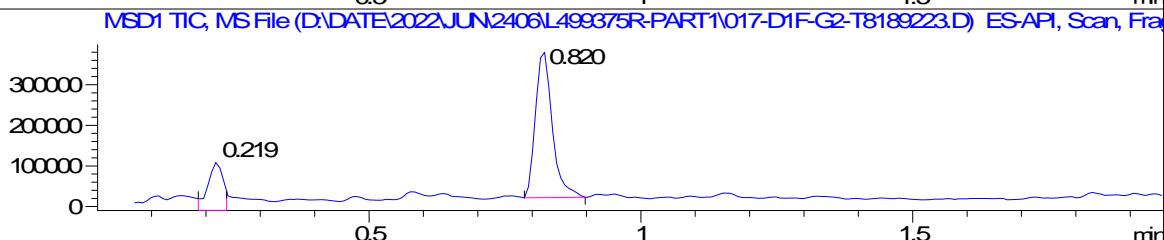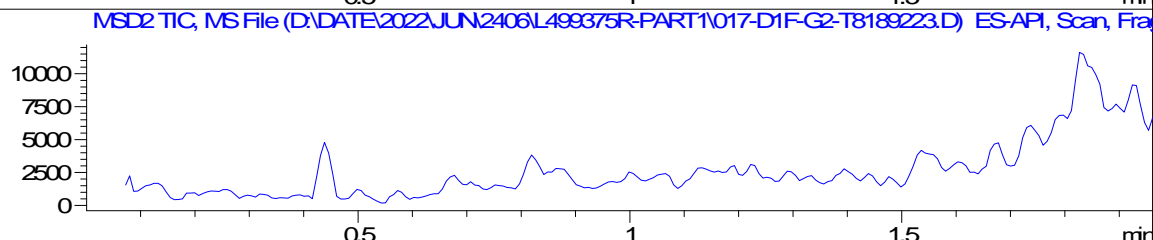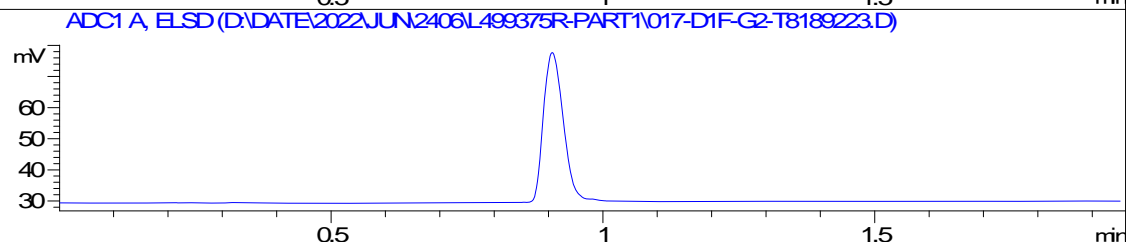

RT 0.219

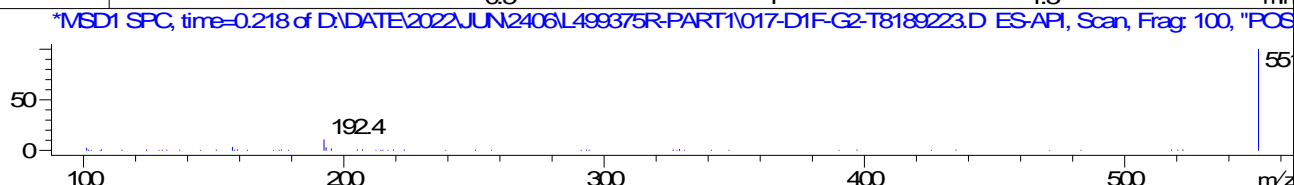

RT 0.820

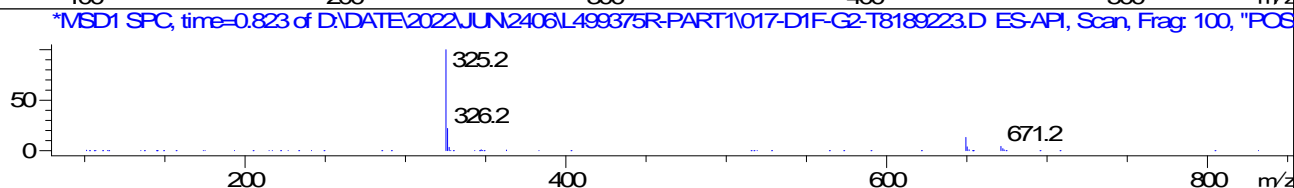

Supplement: Supplementary file 1 — Supplementary Information 1. [file 41598_2024_54655_MOESM1_ESM.zip › Nature SREP/QC_AIMS_files/Proj184.pdf]

T8020489

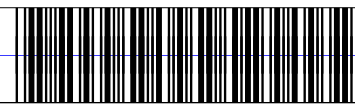

MaxPeak: 100.00%  
Ret\_Time: 0.986 min

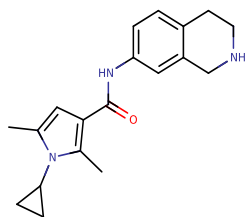

Mol Wt 345.87  
Exact Mass 309.22

| # | Time  | Area%  |
|---|-------|--------|
| 1 | 0.986 | 100.00 |

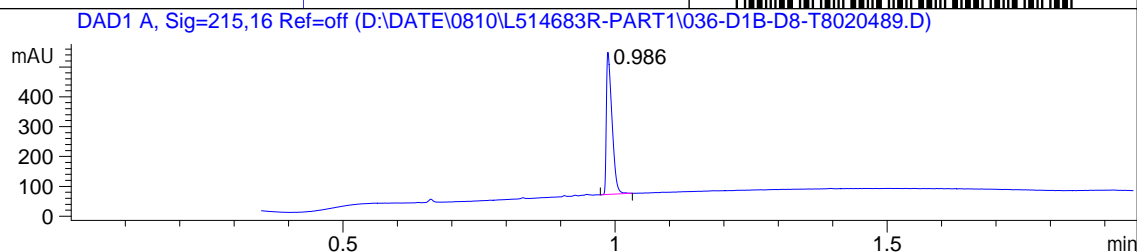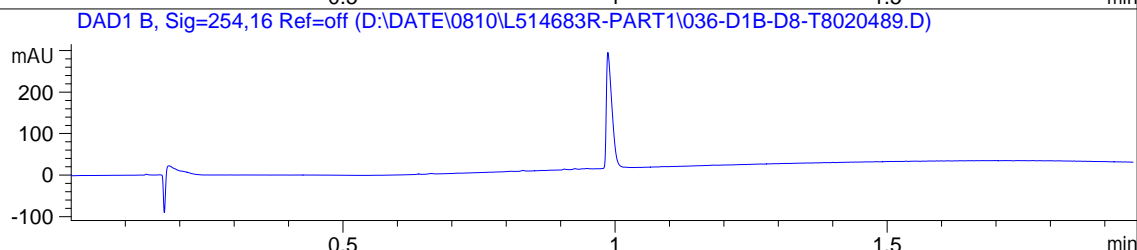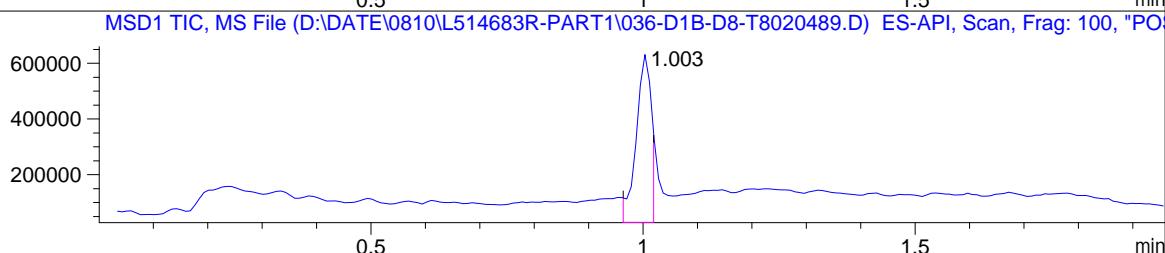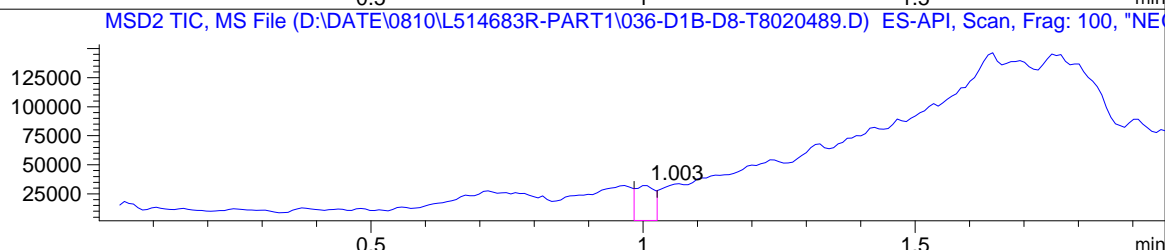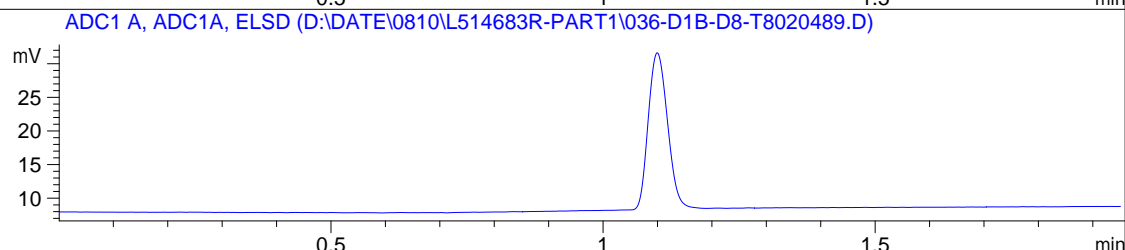

RT 1.003

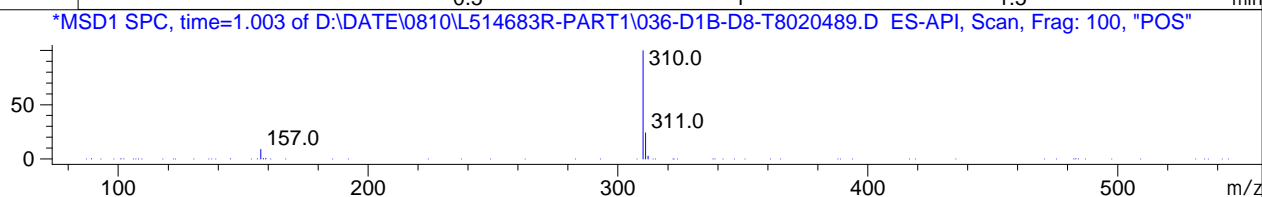

RT 1.003

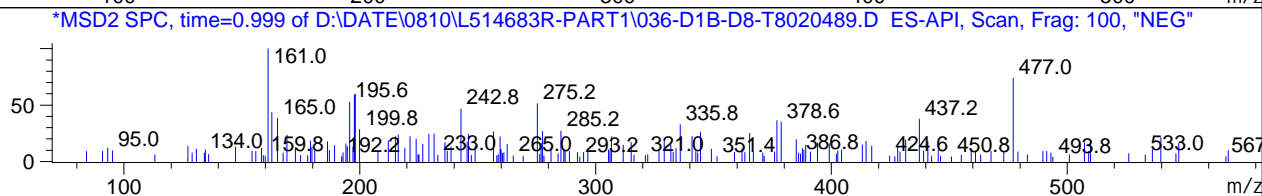

Supplement: Supplementary file 1 — Supplementary Information 1. [file 41598_2024_54655_MOESM1_ESM.zip › Nature SREP/QC_AIMS_files/Proj185.pdf]

MaxPeak: 92.37%  
Ret\_Time: 1.157 min

4758342

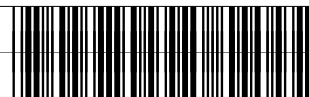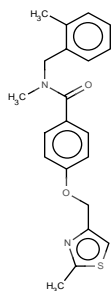

Mol Wt 366.477  
Exact Mass 366.17

| # | Time  | Area% |
|---|-------|-------|
| 1 | 0.343 | 4.86  |
| 2 | 0.825 | 2.77  |
| 3 | 1.157 | 92.37 |

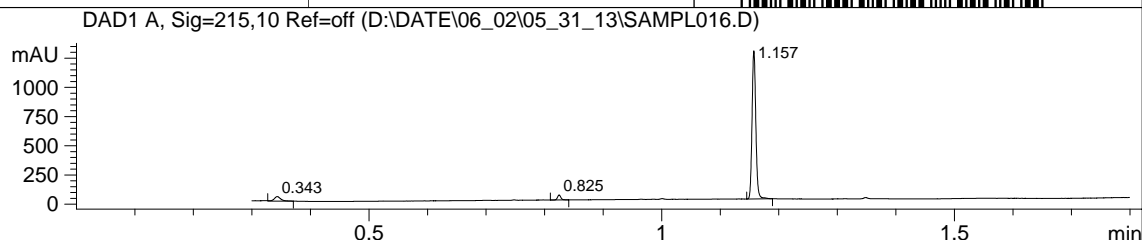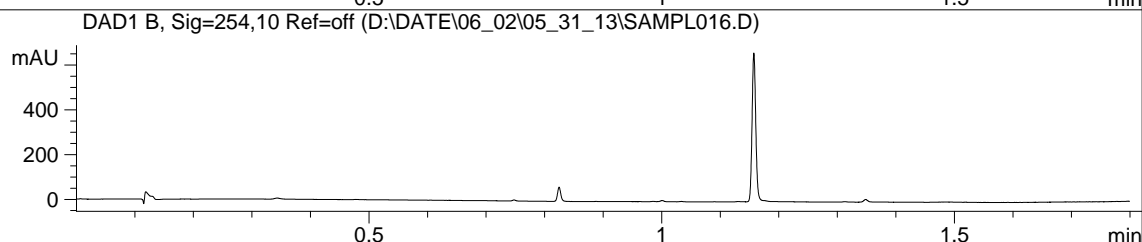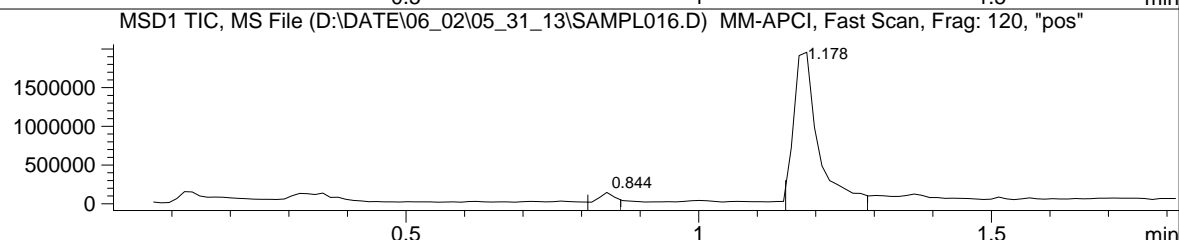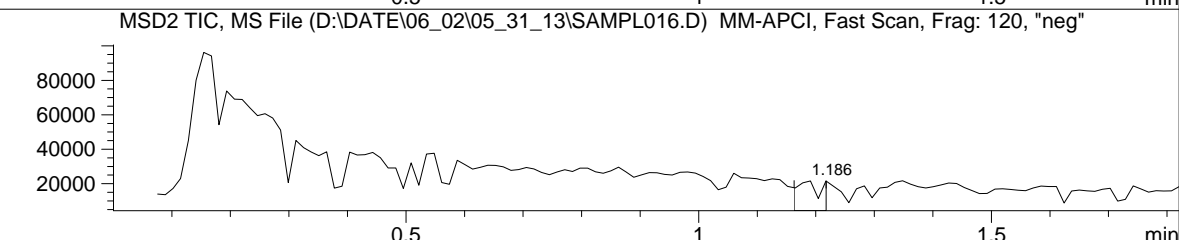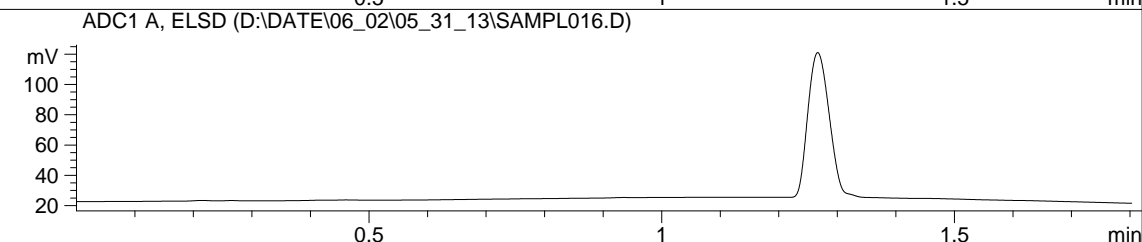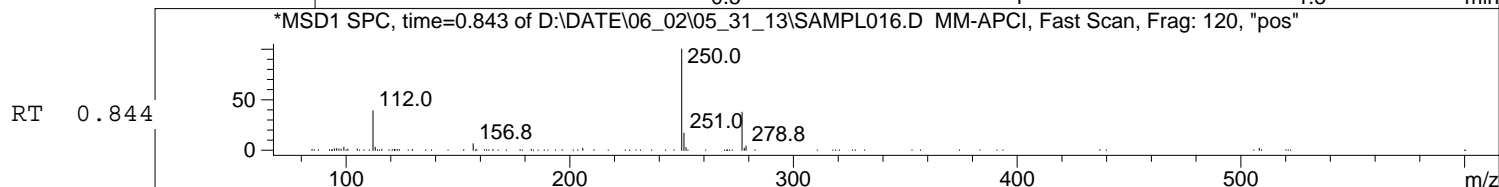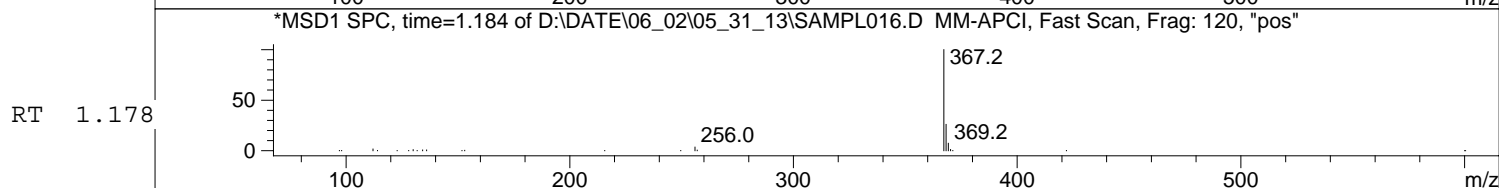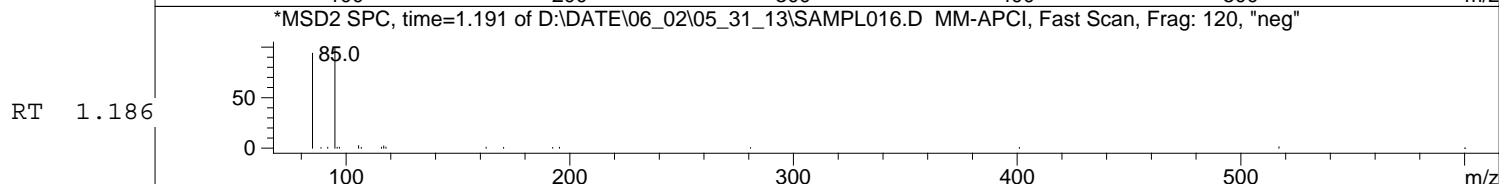

Supplement: Supplementary file 1 — Supplementary Information 1. [file 41598_2024_54655_MOESM1_ESM.zip › Nature SREP/QC_AIMS_files/Proj187.pdf]

T5358975

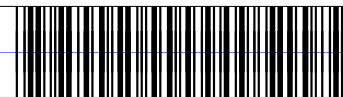

MaxPeak: 100.00%  
Ret\_Time: 1.186 min

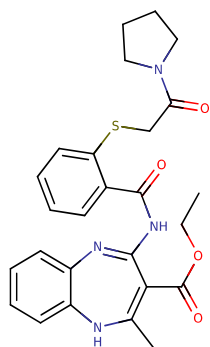

Mol Wt 492.59  
Exact Mass 492.21

| # | Time  | Area%  |
|---|-------|--------|
| 1 | 1.186 | 100.00 |

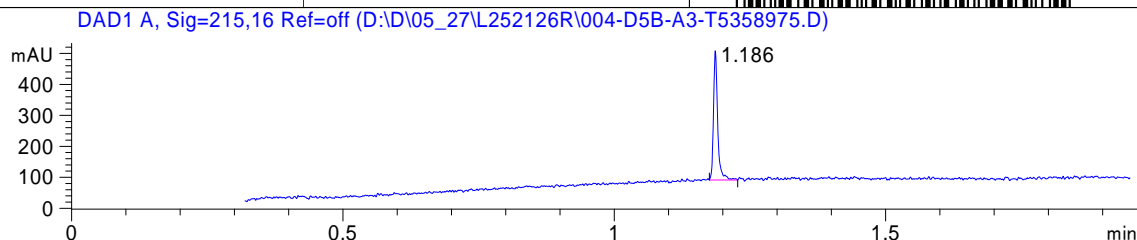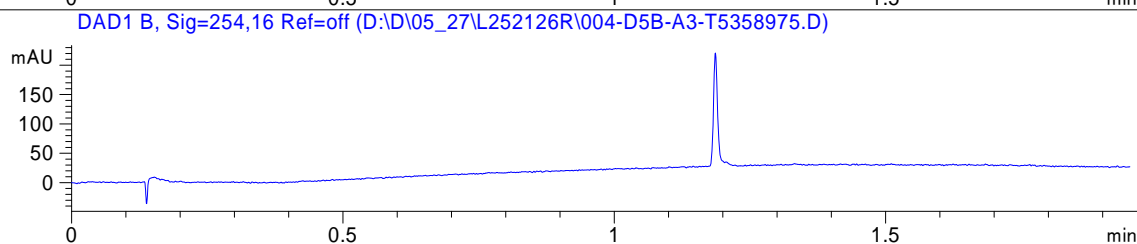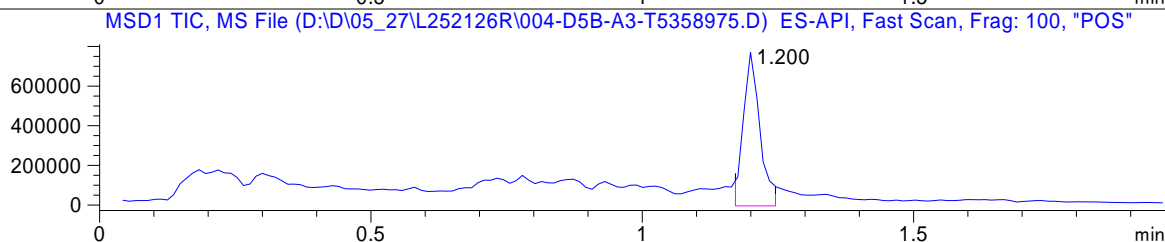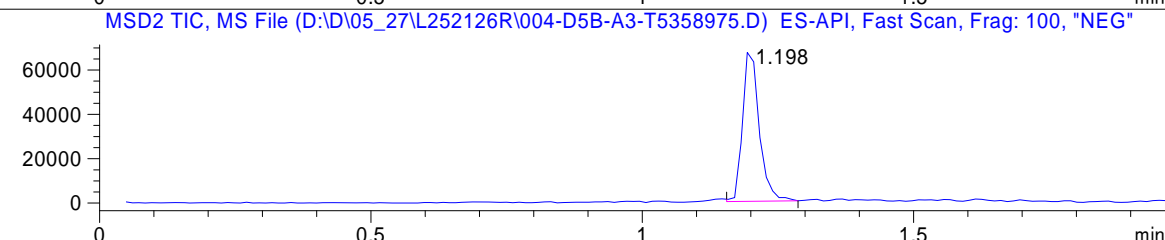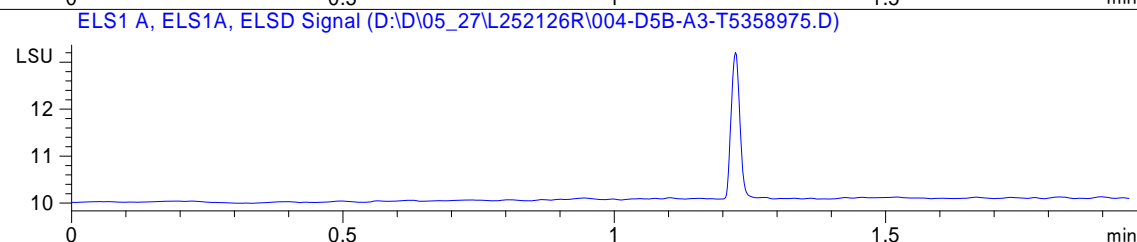

RT 1.200

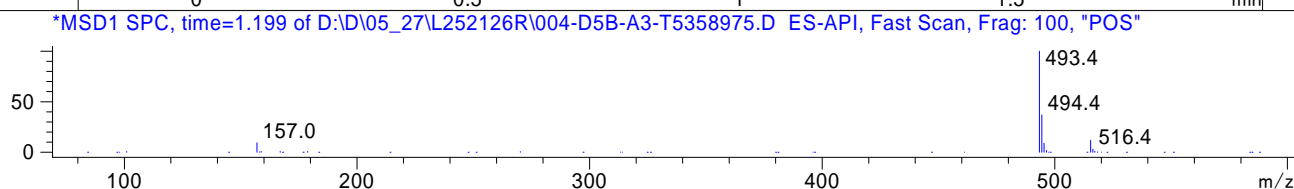

RT 1.198

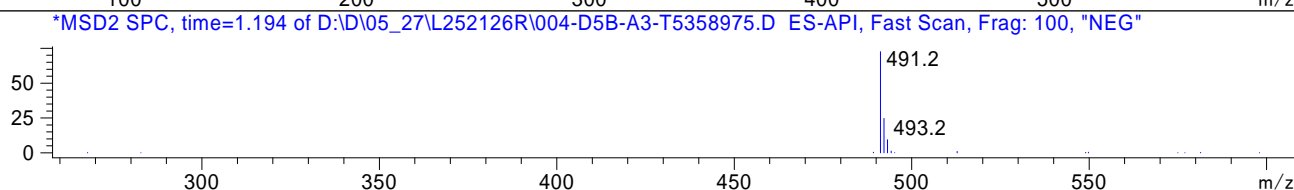

Supplement: Supplementary file 1 — Supplementary Information 1. [file 41598_2024_54655_MOESM1_ESM.zip › Nature SREP/QC_AIMS_files/Proj189.pdf]

MaxPeak: 91.42%  
Ret\_Time: 1.418 min

T5622662

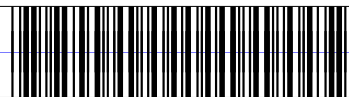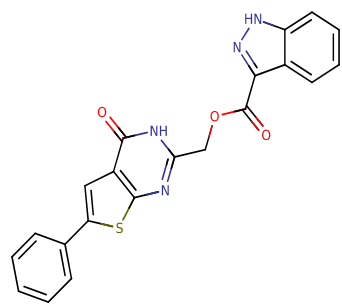

Mol Wt 402.43  
Exact Mass 402.08

| # | Time  | Area% |
|---|-------|-------|
| 1 | 1.173 | 3.90  |
| 2 | 1.418 | 91.42 |
| 3 | 1.594 | 4.68  |

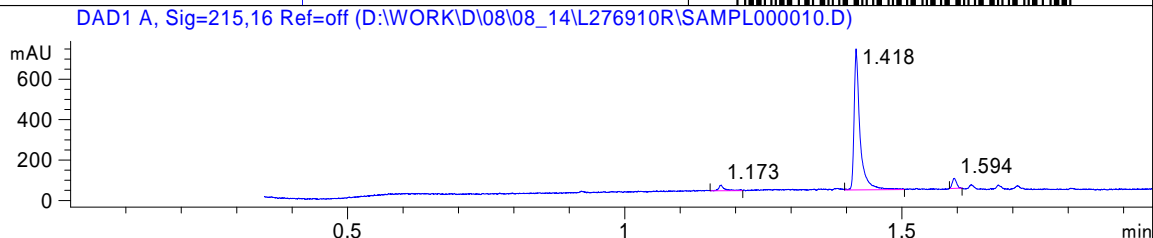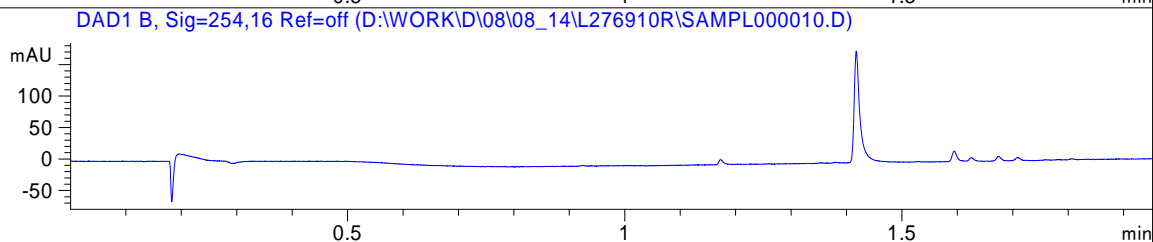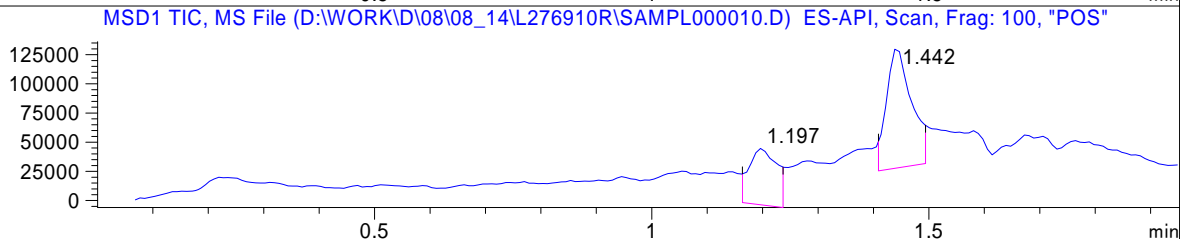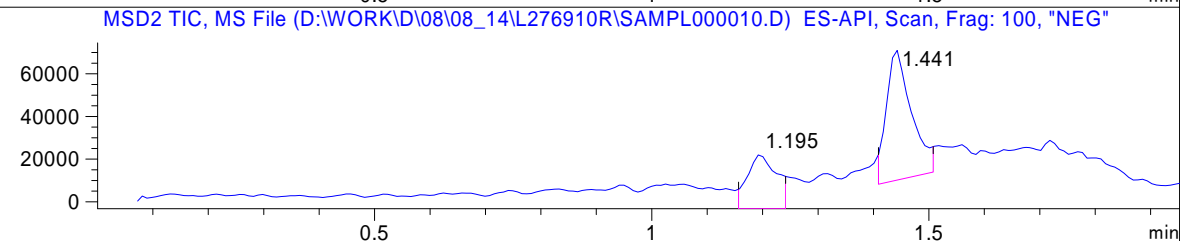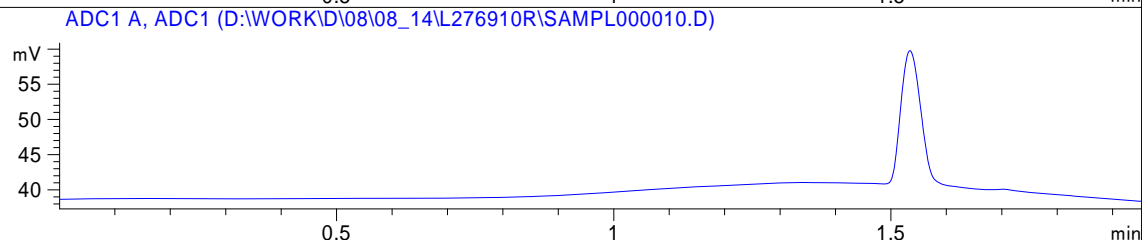

RT 1.197

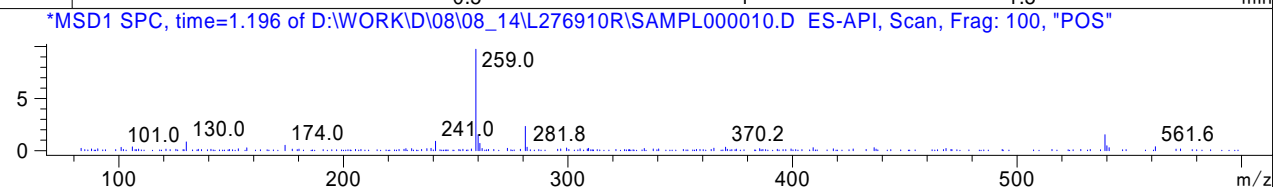

RT 1.442

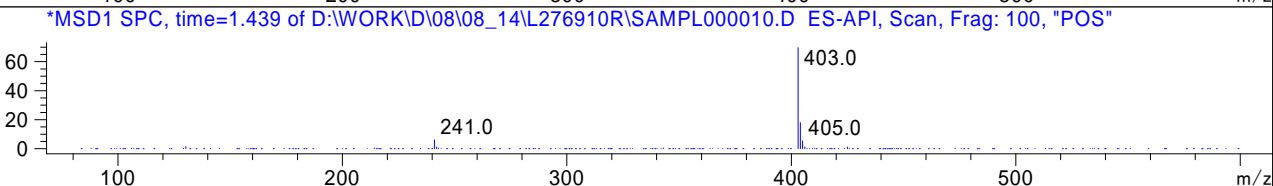

RT 1.195

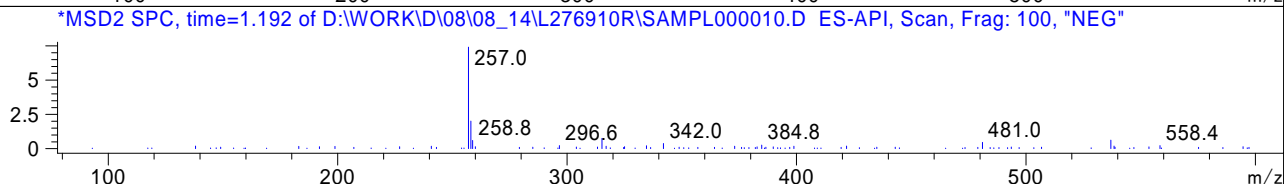

RT 1.441

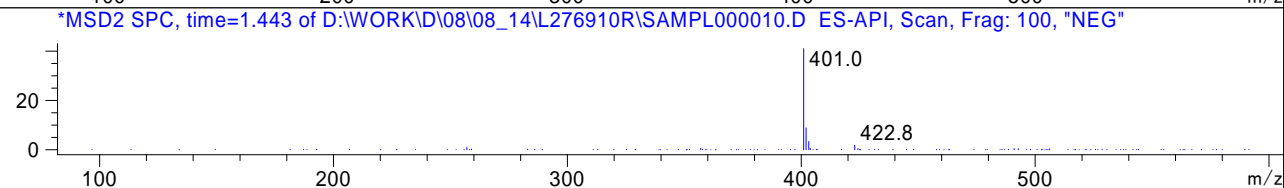

Supplement: Supplementary file 1 — Supplementary Information 1. [file 41598_2024_54655_MOESM1_ESM.zip › Nature SREP/QC_AIMS_files/Proj191.pdf]

T6914764

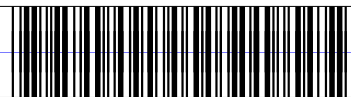

MaxPeak: 100.00%  
Ret\_Time: 1.329 min

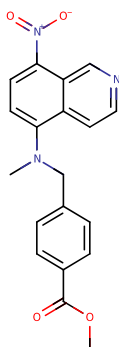

Mol Wt 351.36  
Exact Mass 351.13

| # | Time  | Area%  |
|---|-------|--------|
| 1 | 1.329 | 100.00 |

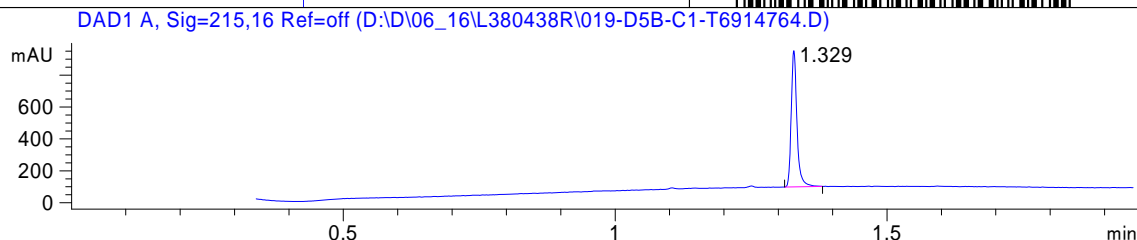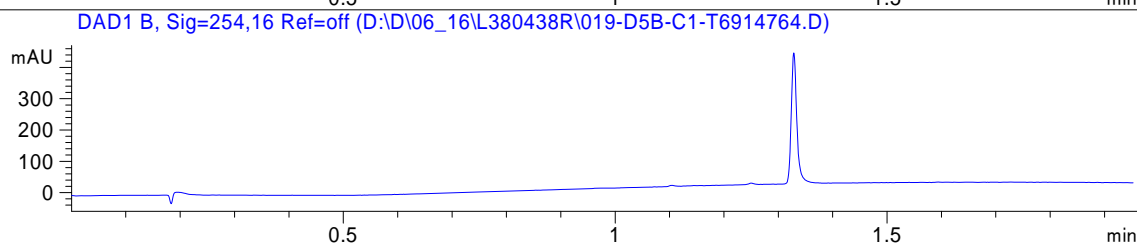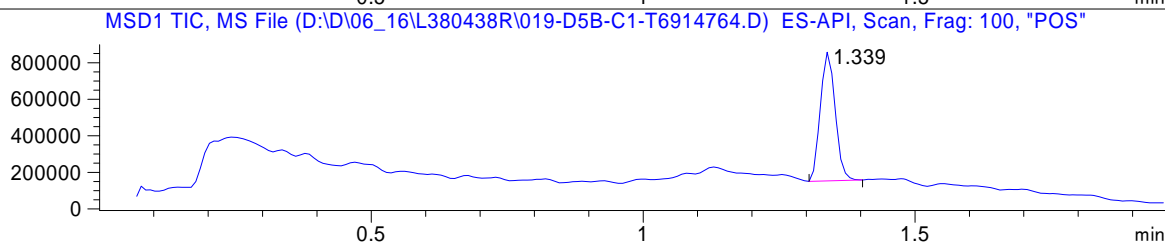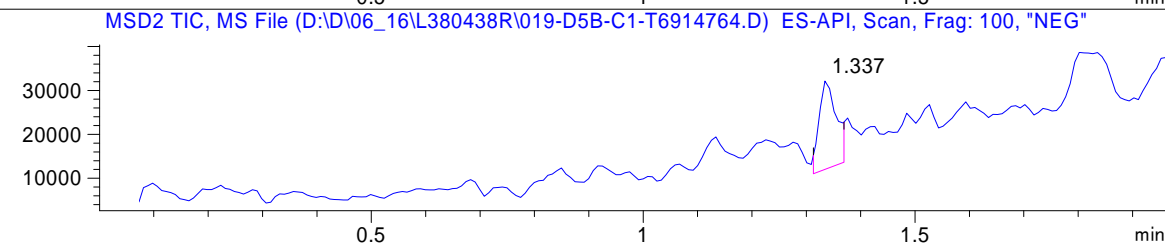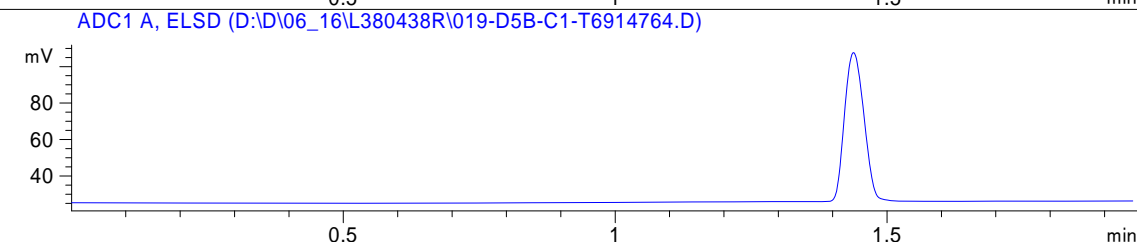

RT 1.339

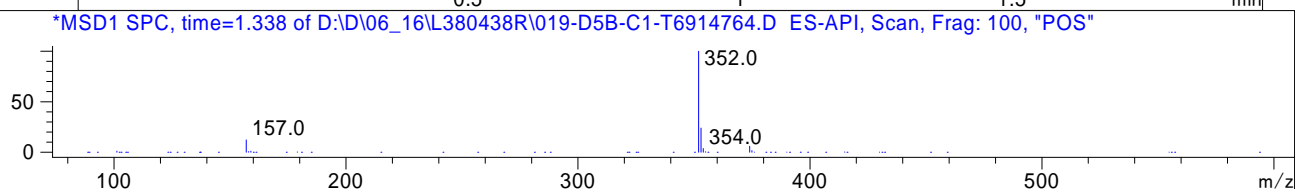

RT 1.337

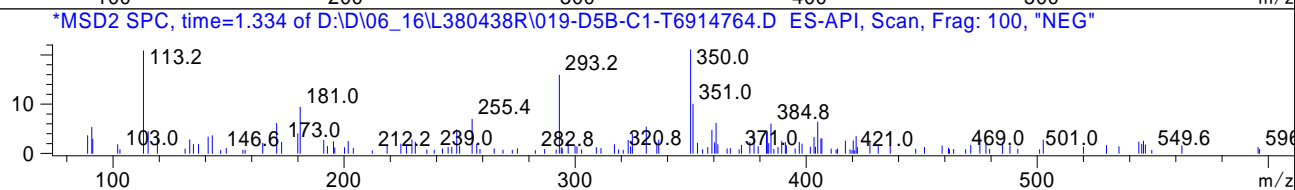

Supplement: Supplementary file 1 — Supplementary Information 1. [file 41598_2024_54655_MOESM1_ESM.zip › Nature SREP/QC_AIMS_files/Proj193.pdf]

A4015/0171245

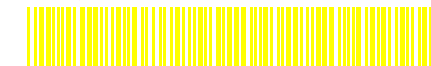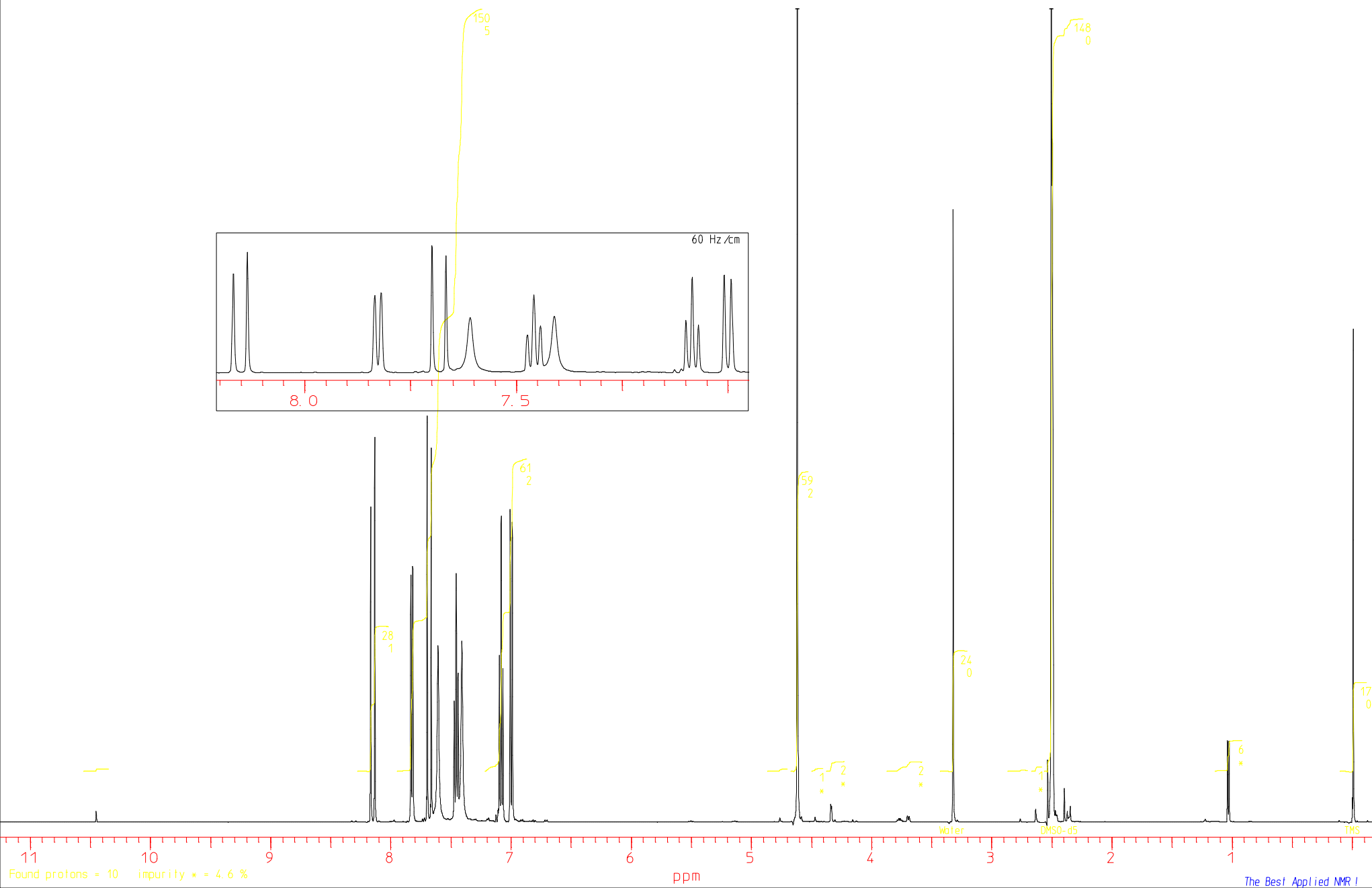

Supplement: Supplementary file 1 — Supplementary Information 1. [file 41598_2024_54655_MOESM1_ESM.zip › Nature SREP/QC_AIMS_files/Proj195.pdf]

T8626317

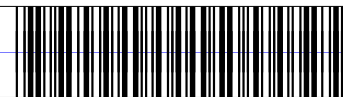

MaxPeak: 92.31%  
Ret\_Time: 0.724 min

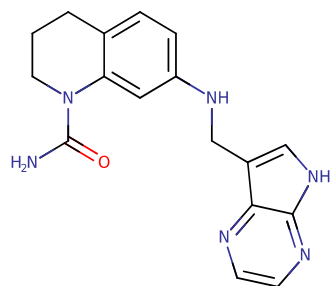

Mol Wt 322.36  
Exact Mass 322.17

| # | Time  | Area% |
|---|-------|-------|
| 1 | 0.724 | 92.31 |
| 2 | 0.785 | 7.69  |

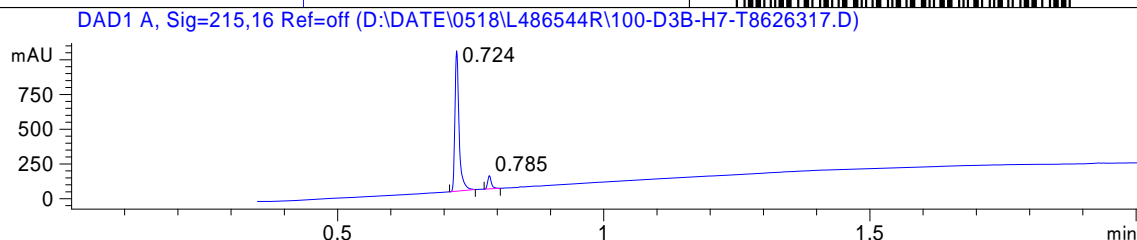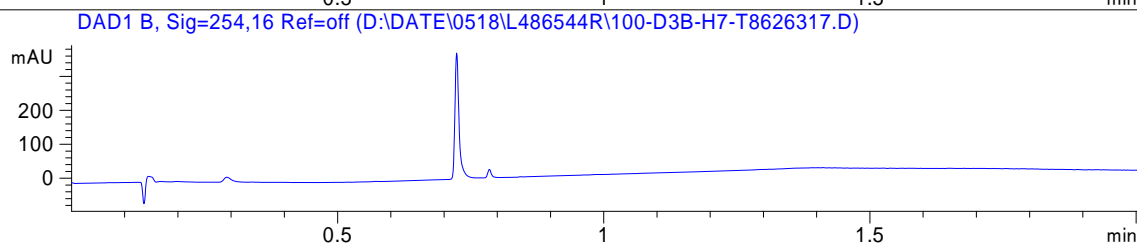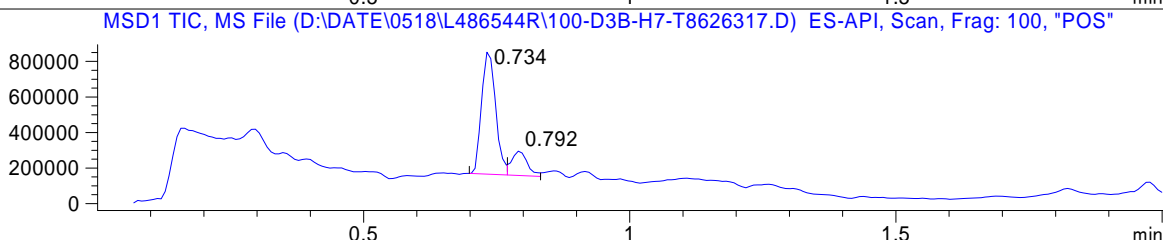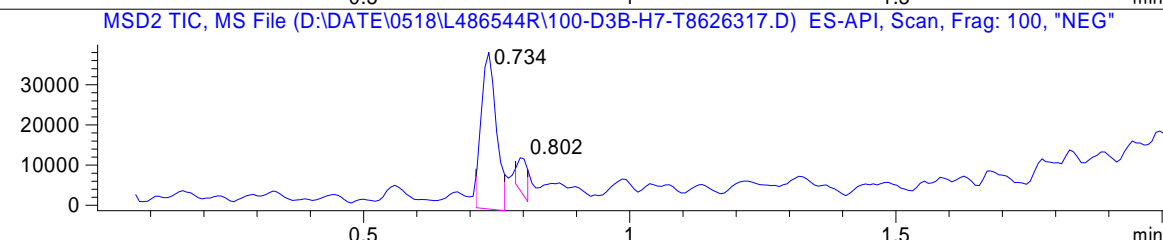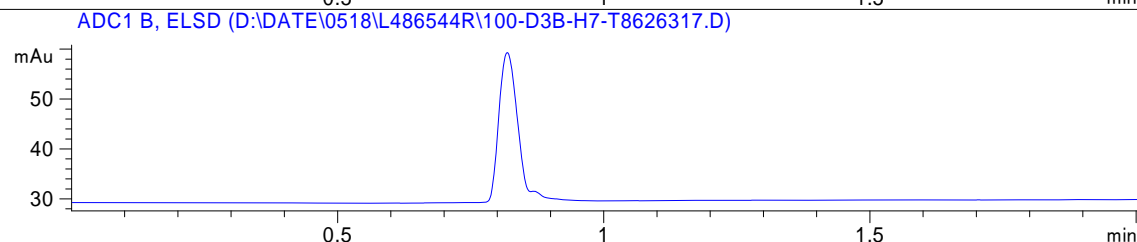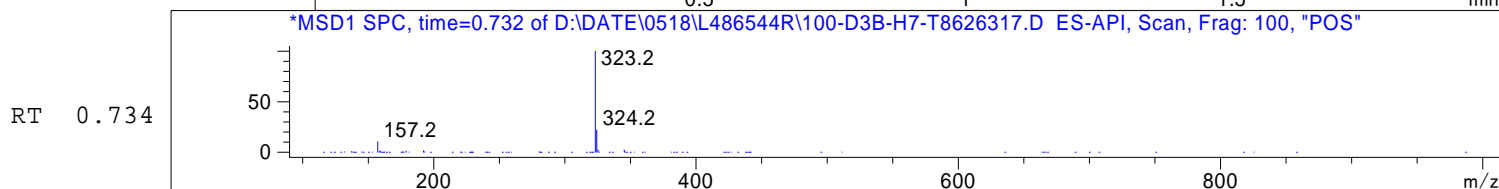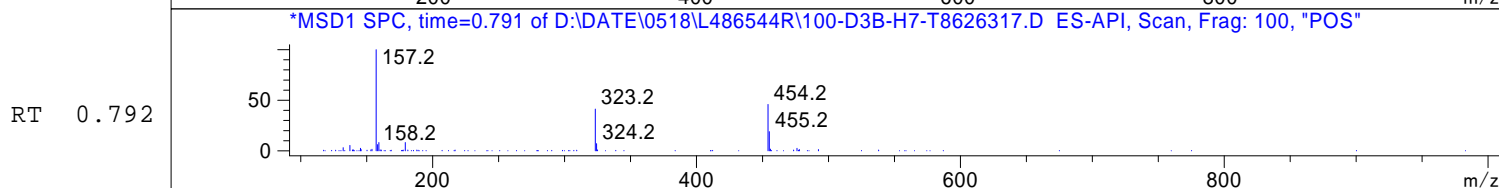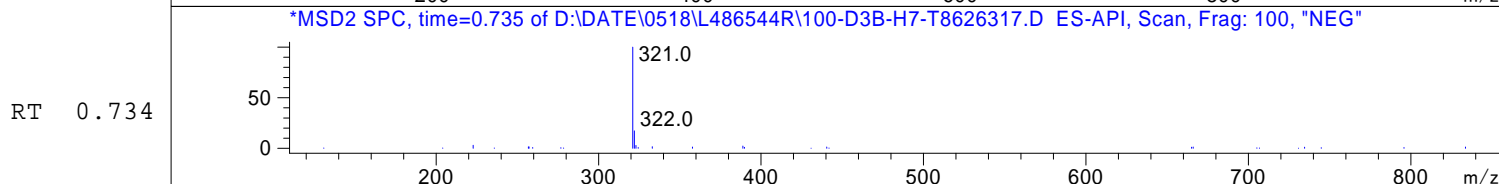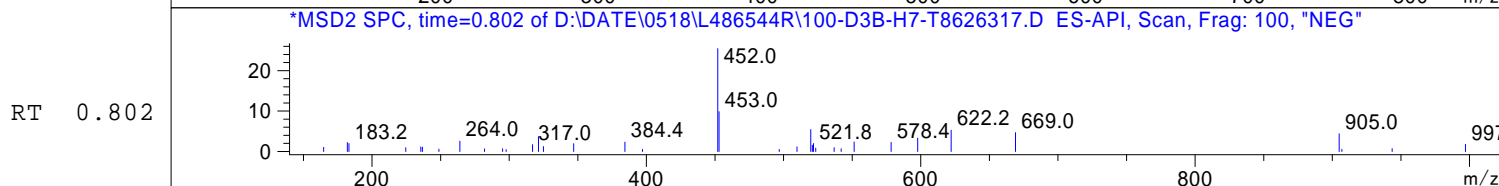

Supplement: Supplementary file 1 — Supplementary Information 1. [file 41598_2024_54655_MOESM1_ESM.zip › Nature SREP/QC_AIMS_files/Proj197.pdf]

MaxPeak: 94.34%  
Ret\_Time: 1.304 min

8681483\$13

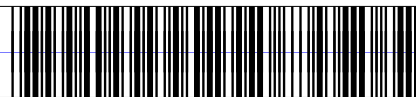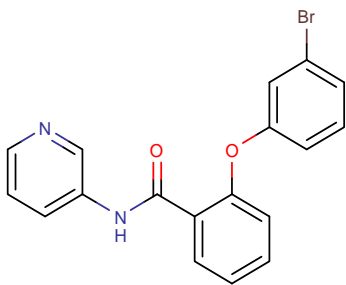

Mol Wt 369.21  
Exact Mass 368.03

| # | Time  | Area% |
|---|-------|-------|
| 1 | 1.304 | 94.34 |
| 2 | 1.400 | 5.66  |

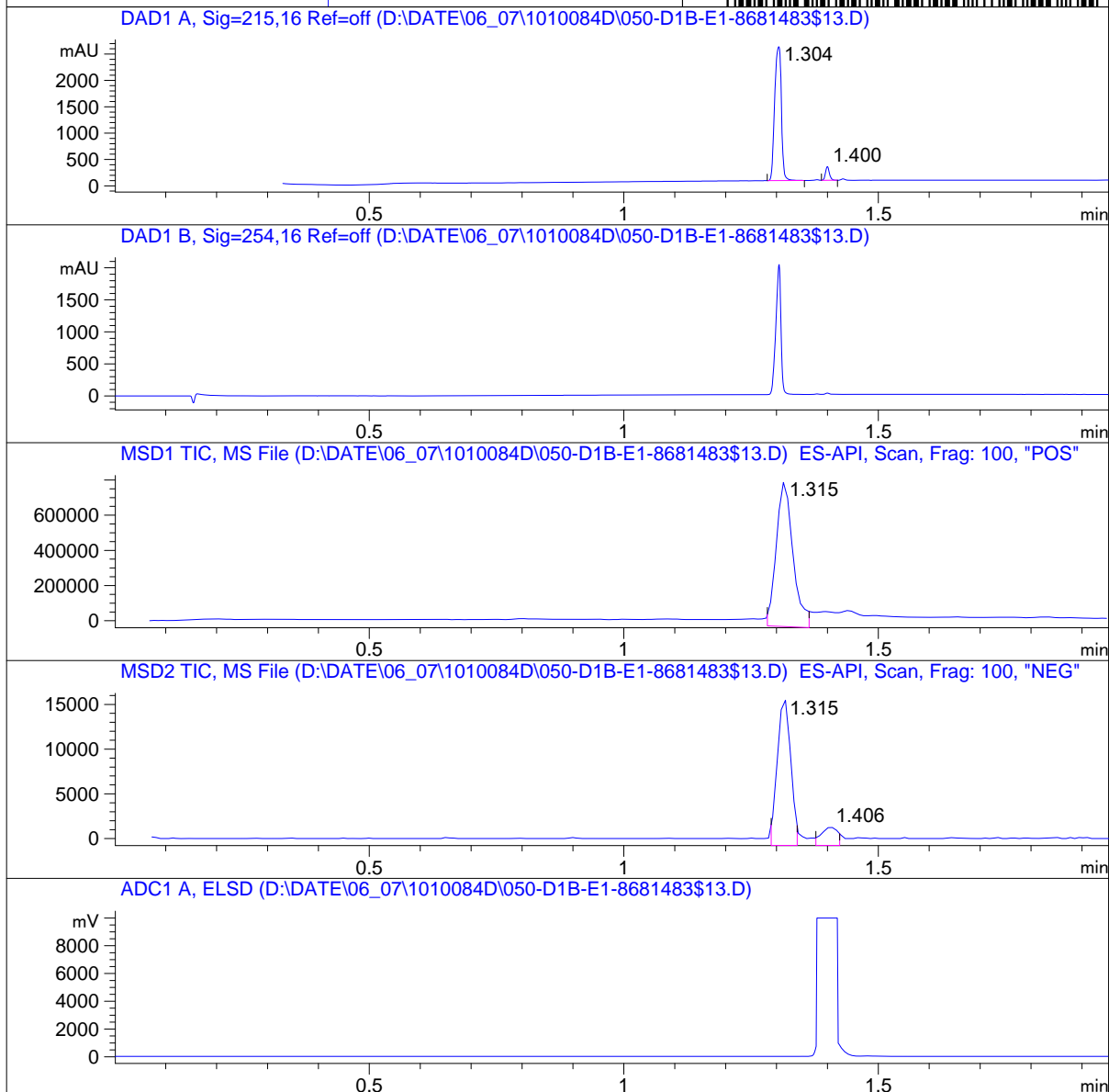

RT 1.315

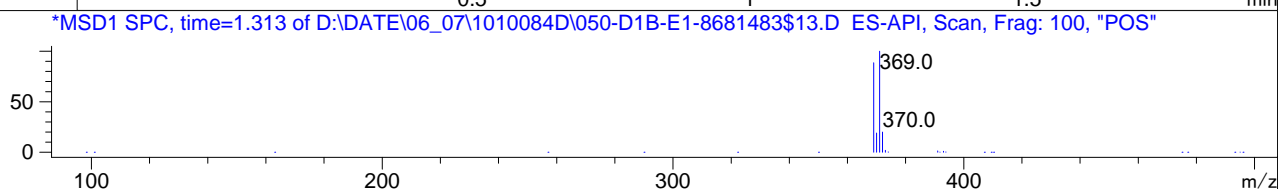

RT 1.315

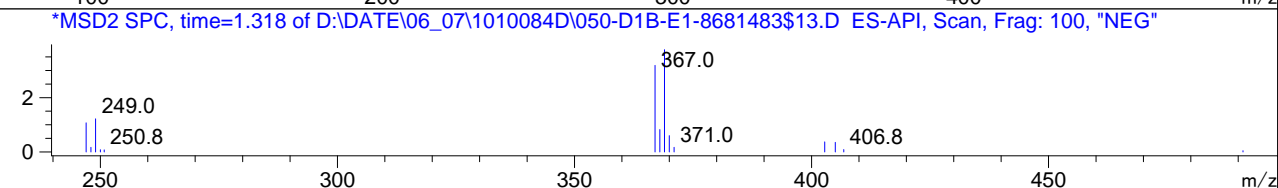

RT 1.406

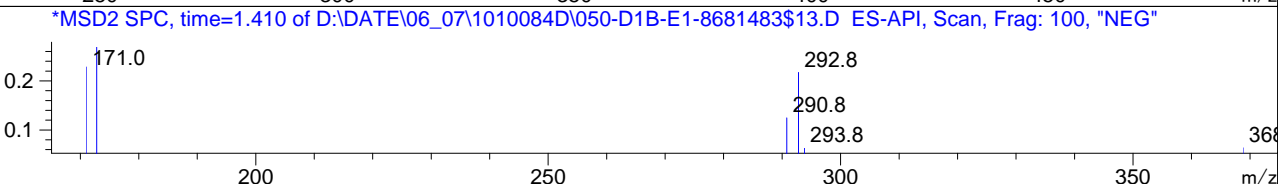

Supplement: Supplementary file 1 — Supplementary Information 1. [file 41598_2024_54655_MOESM1_ESM.zip › Nature SREP/QC_AIMS_files/Proj202.pdf]

MaxPeak: 100.00%  
Ret\_Time: 1.064 min

T6289157

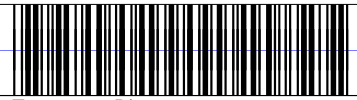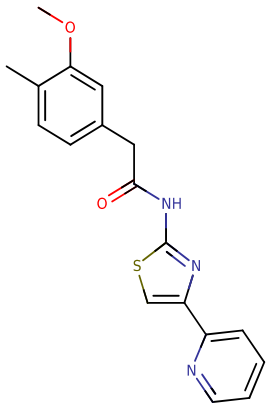

Mol Wt 339.41  
Exact Mass 339.12

| # | Time  | Area%  |
|---|-------|--------|
| 1 | 1.064 | 100.00 |

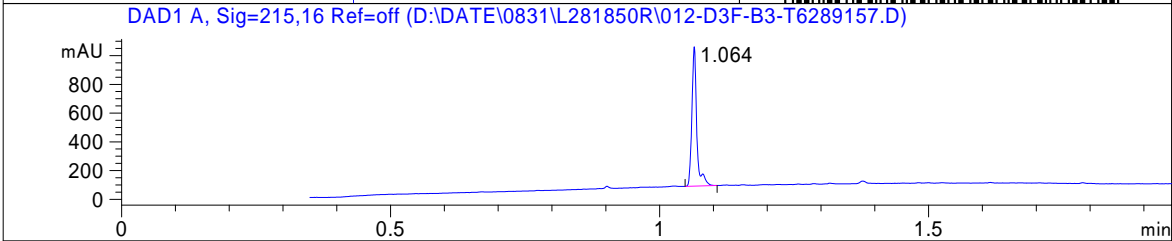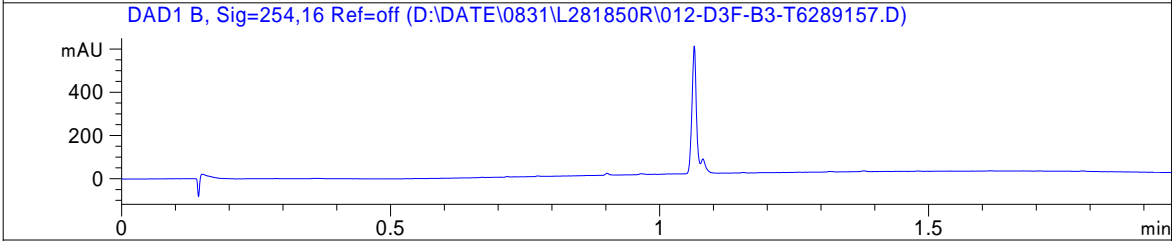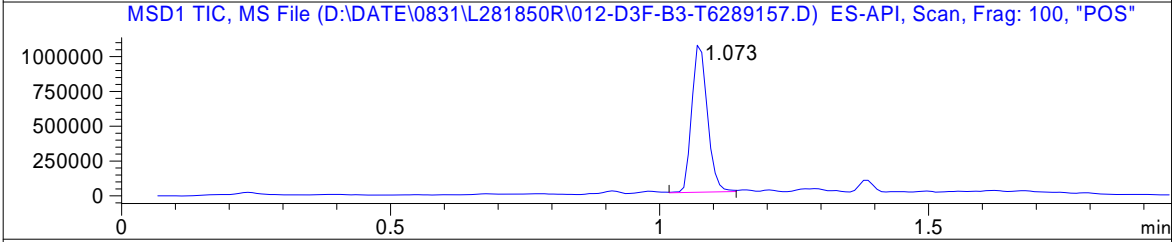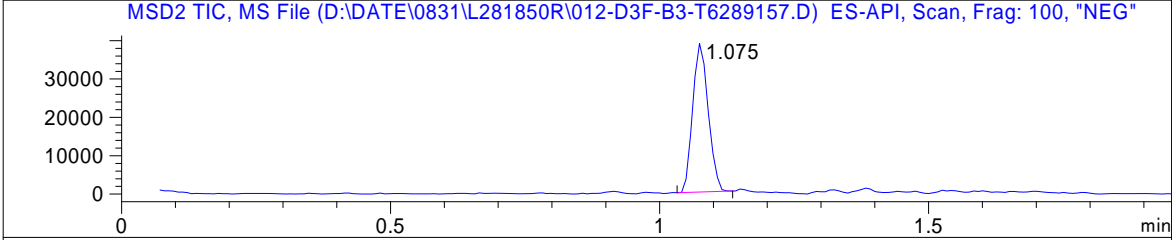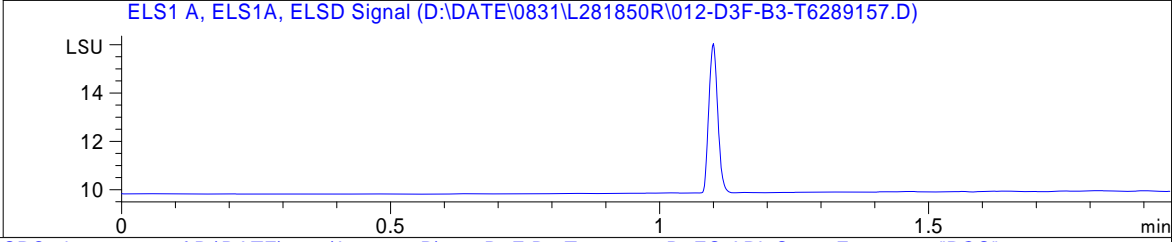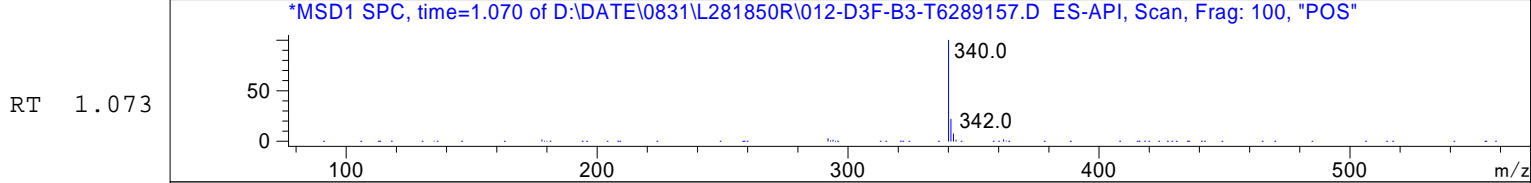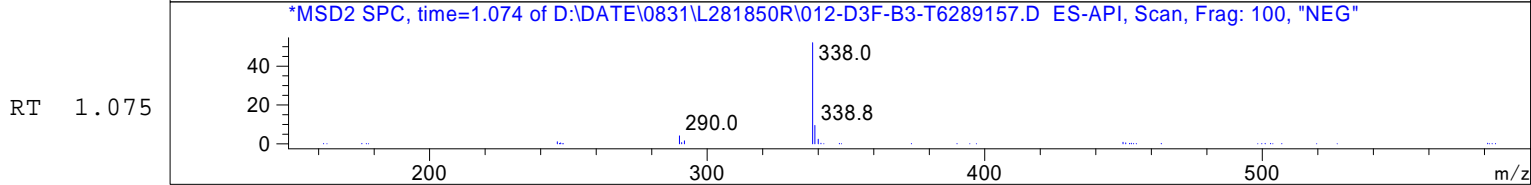

Supplement: Supplementary file 1 — Supplementary Information 1. [file 41598_2024_54655_MOESM1_ESM.zip › Nature SREP/QC_AIMS_files/Proj203.pdf]

T7374473

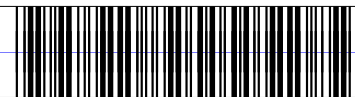

MaxPeak: 90.40%  
Ret\_Time: 1.525 min

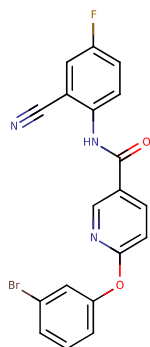

Mol Wt 412.21  
Exact Mass 411.01

| # | Time  | Area% |
|---|-------|-------|
| 1 | 0.610 | 3.11  |
| 2 | 0.772 | 4.51  |
| 3 | 1.340 | 1.98  |
| 4 | 1.525 | 90.40 |

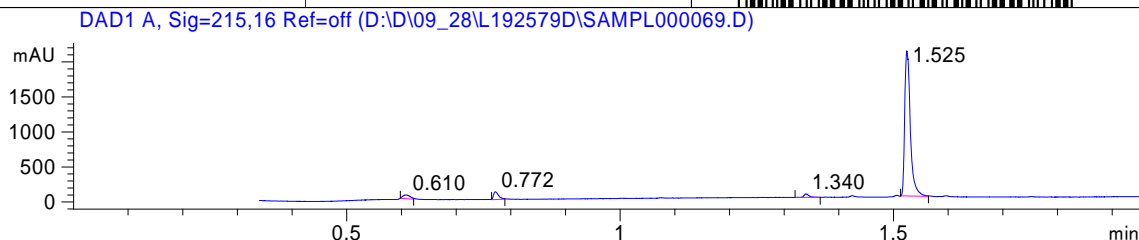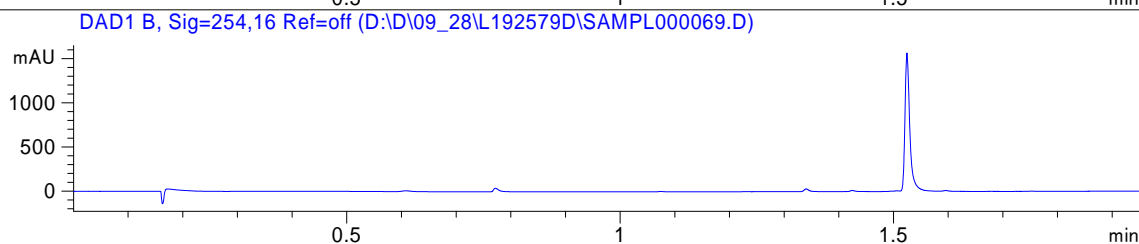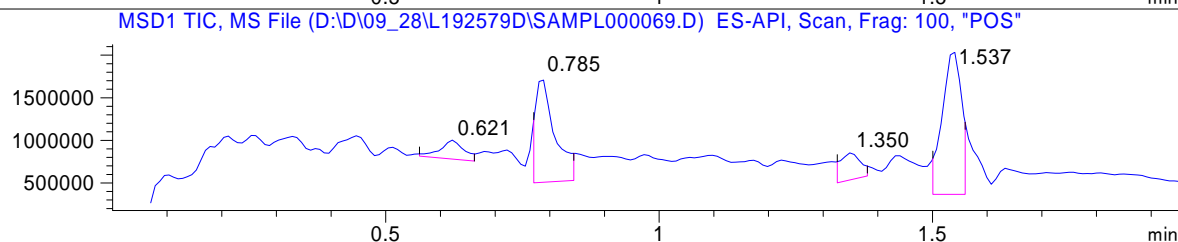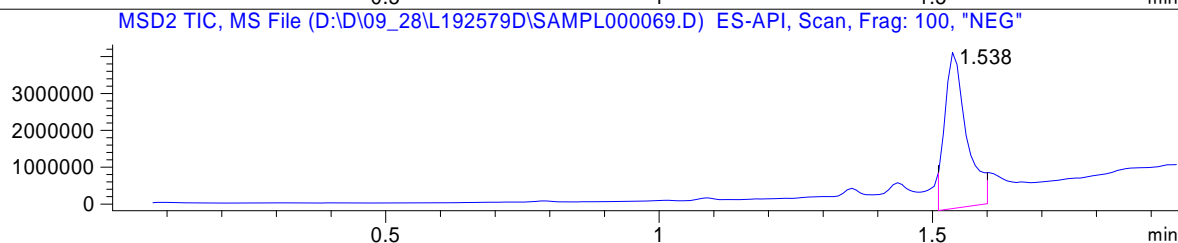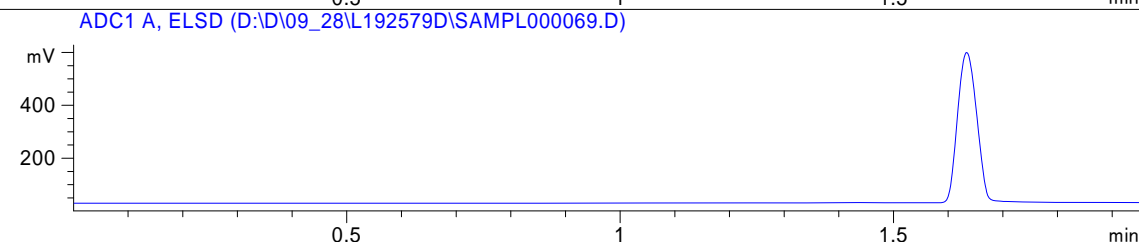

RT 0.621

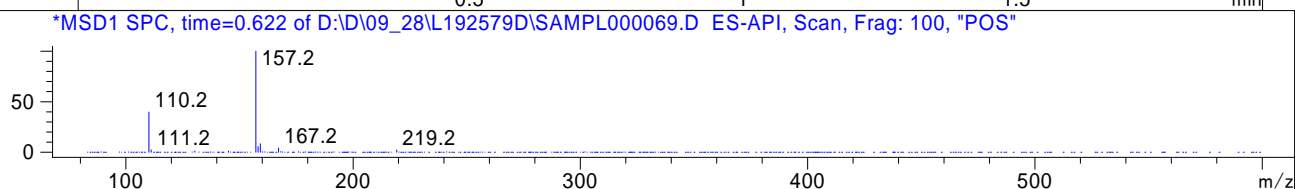

RT 0.785

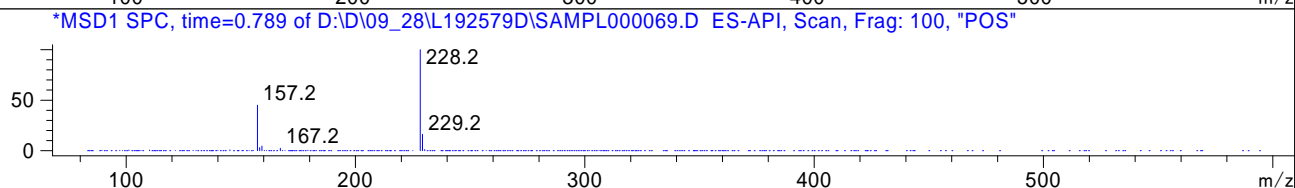

RT 1.350

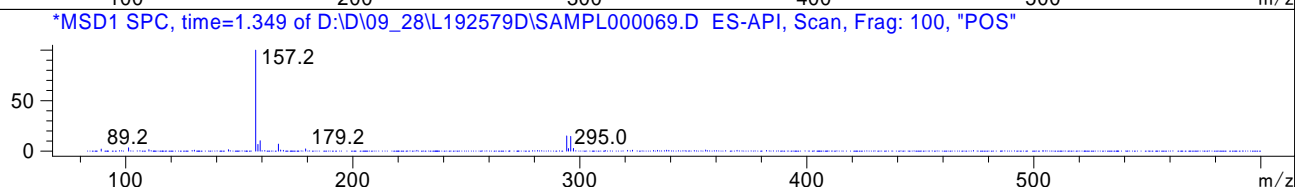

RT 1.537

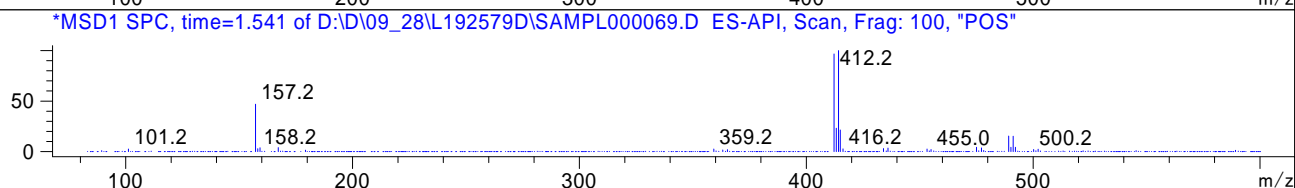

RT 1.538

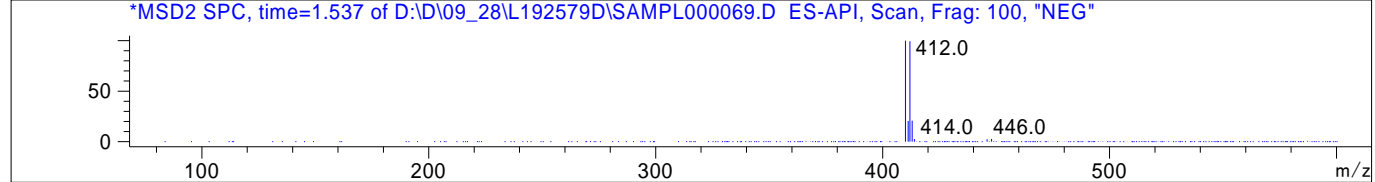

Supplement: Supplementary file 1 — Supplementary Information 1. [file 41598_2024_54655_MOESM1_ESM.zip › Nature SREP/QC_AIMS_files/Proj211.pdf]

T6818698

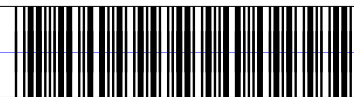

MaxPeak: 95.50%  
Ret\_Time: 1.106 min

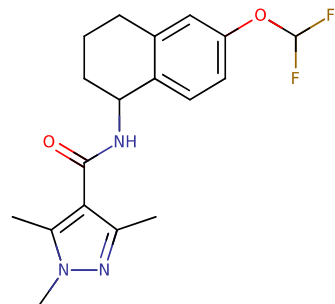

Mol Wt 349.38  
Exact Mass 349.19

| # | Time  | Area% |
|---|-------|-------|
| 1 | 0.515 | 4.50  |
| 2 | 1.106 | 95.50 |

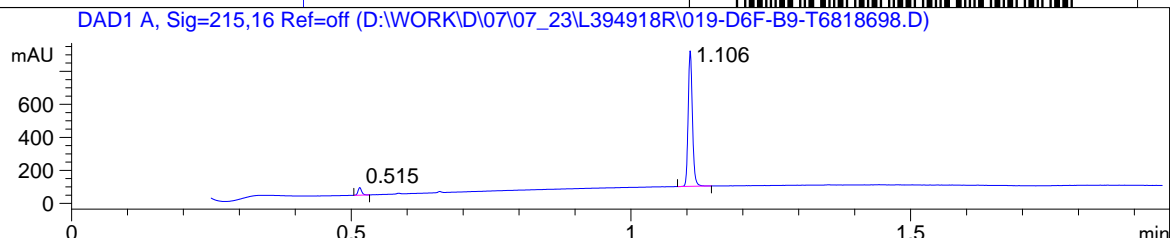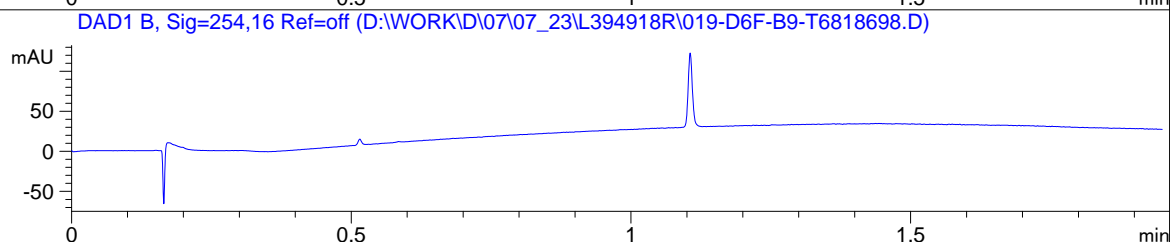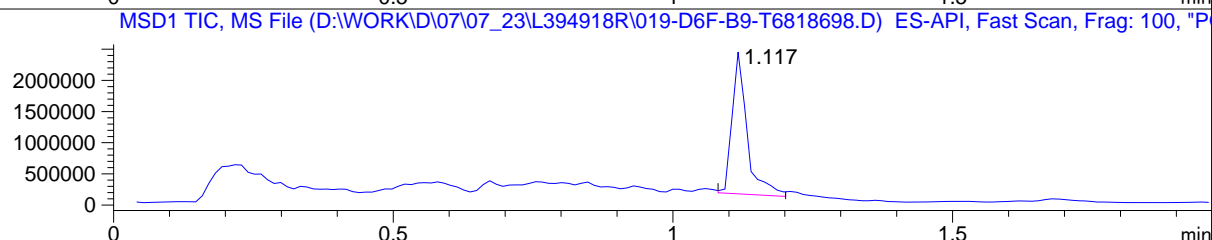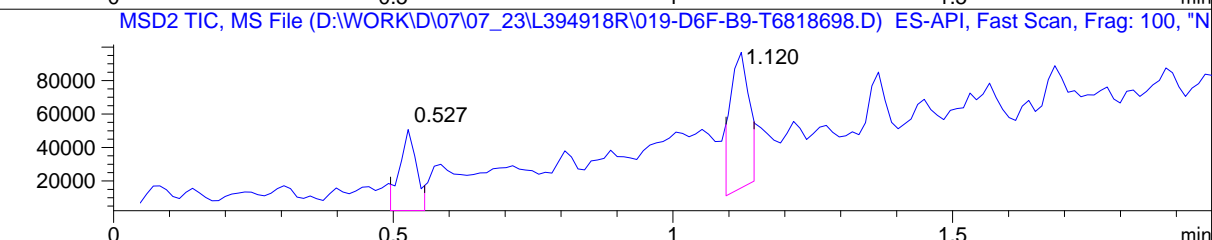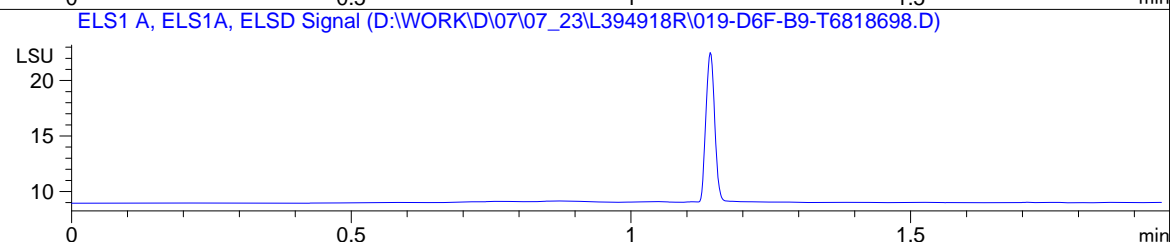

RT 1.117

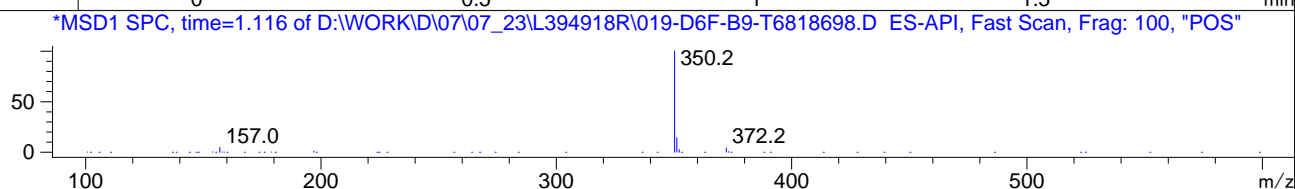

RT 0.527

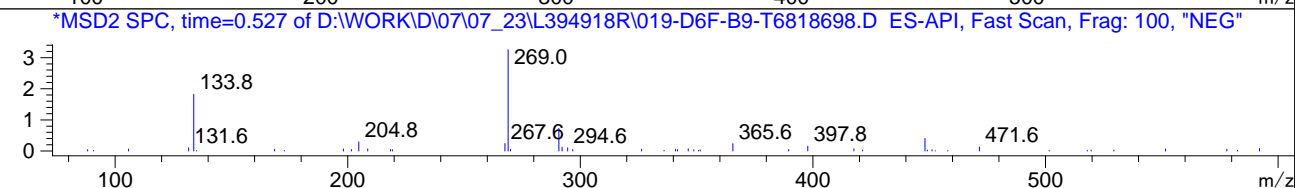

RT 1.120

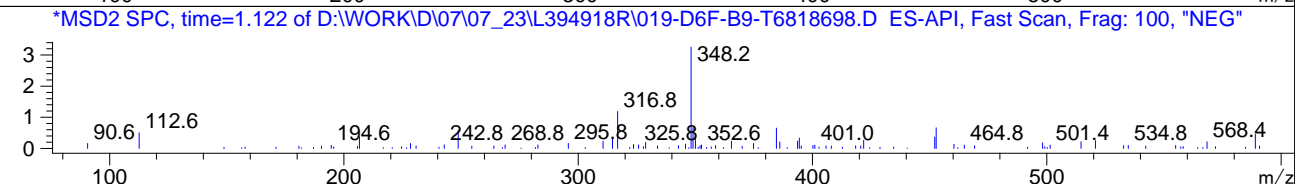

Supplement: Supplementary file 1 — Supplementary Information 1. [file 41598_2024_54655_MOESM1_ESM.zip › Nature SREP/QC_AIMS_files/Proj212.pdf]

IBS-E0350967

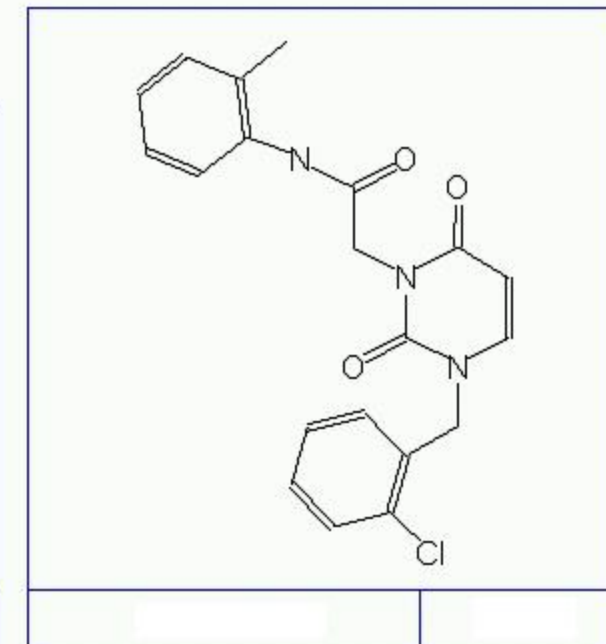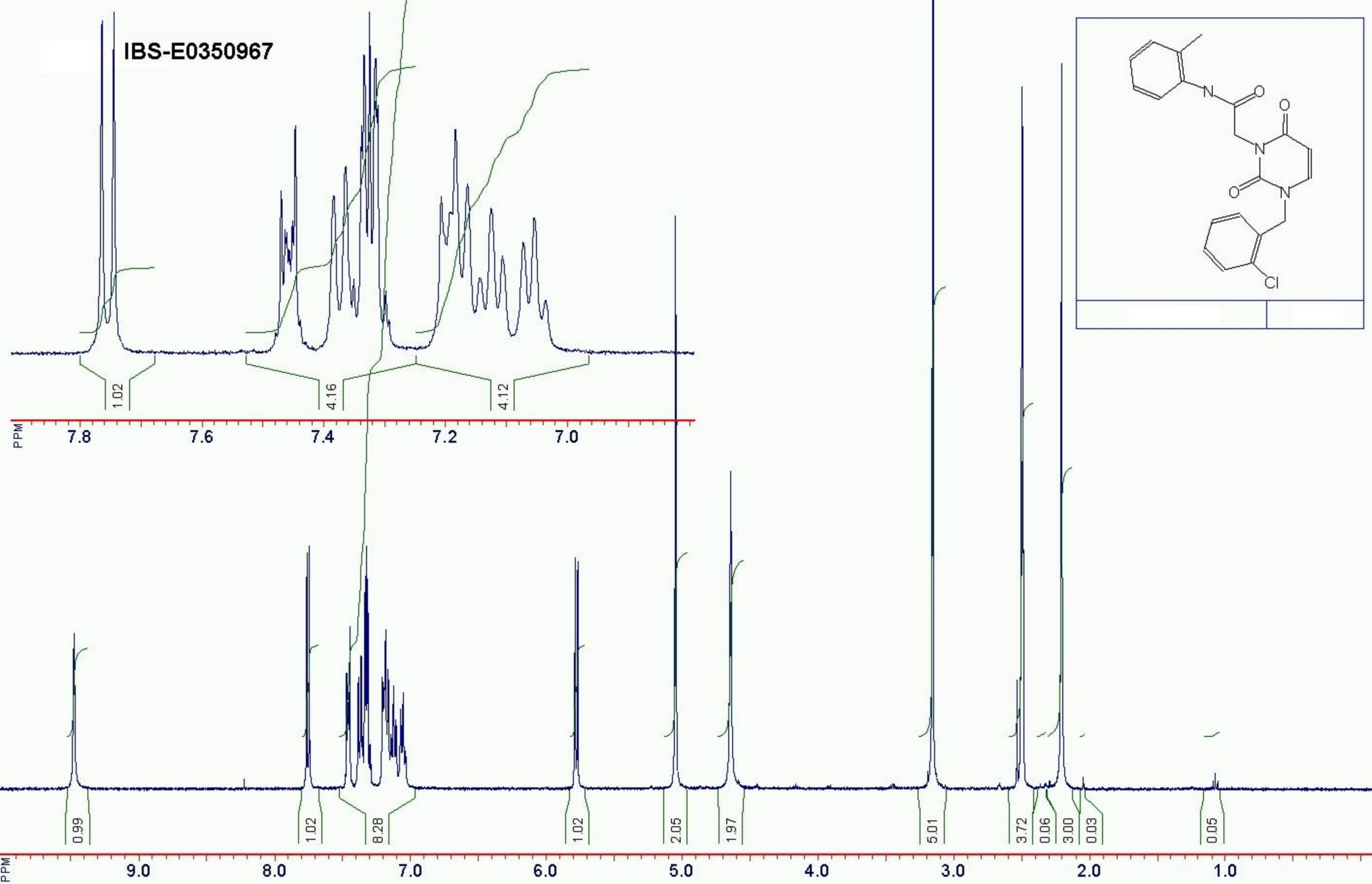

|               |                  |           |                            |                                        |
|---------------|------------------|-----------|----------------------------|----------------------------------------|
|               | SF: 400.4000 MHz | NSC: 1    | PW: 0.00 usec, RG: 128     |                                        |
| Solvent: DMSO | SW: 7246 Hz      | TE: 300 K | AQ: 2.26 sec, RD: 0.00 sec | Parameter file, XWIN-NMR == A14550_p3□ |

Supplement: Supplementary file 1 — Supplementary Information 1. [file 41598_2024_54655_MOESM1_ESM.zip › Nature SREP/QC_AIMS_files/Proj233.pdf]

T6780864

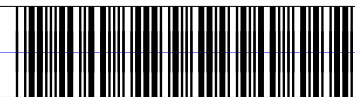

MaxPeak: 96.94%  
Ret\_Time: 1.357 min

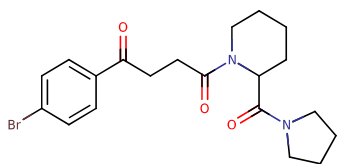

Mol Wt 421.33  
Exact Mass 420.14

| # | Time  | Area% |
|---|-------|-------|
| 1 | 1.273 | 3.06  |
| 2 | 1.357 | 96.94 |

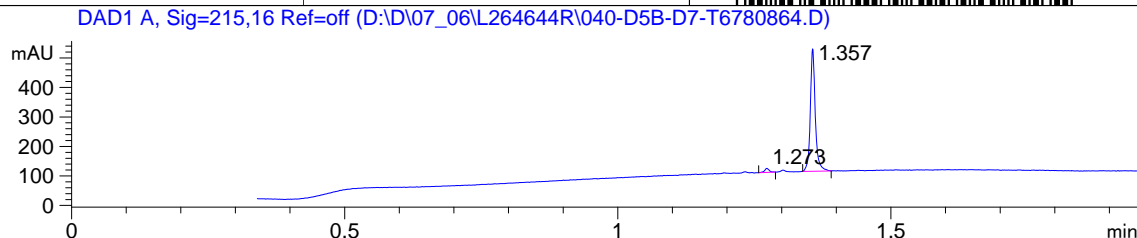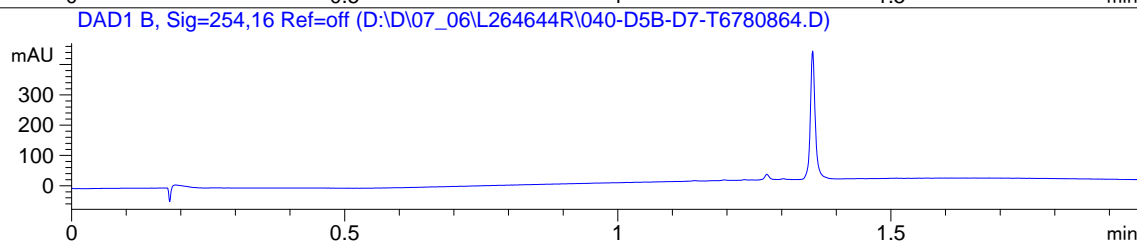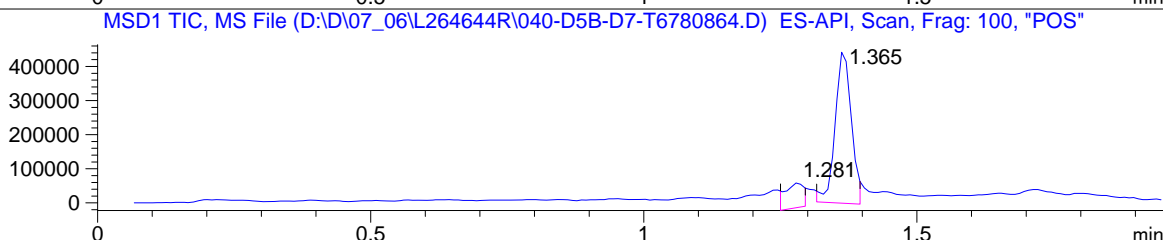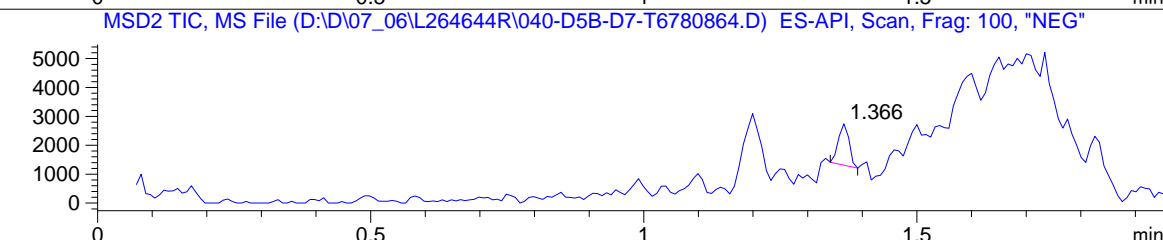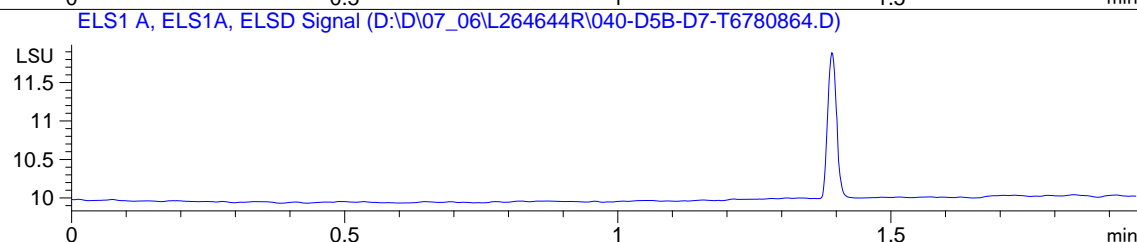

RT 1.281

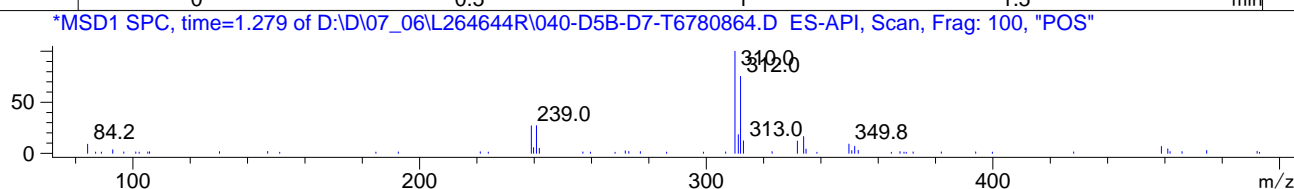

RT 1.365

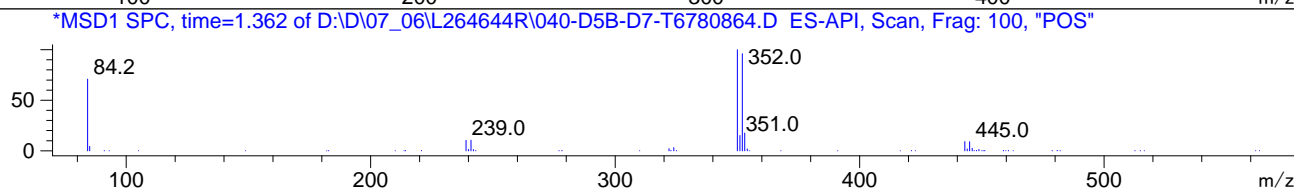

RT 1.366

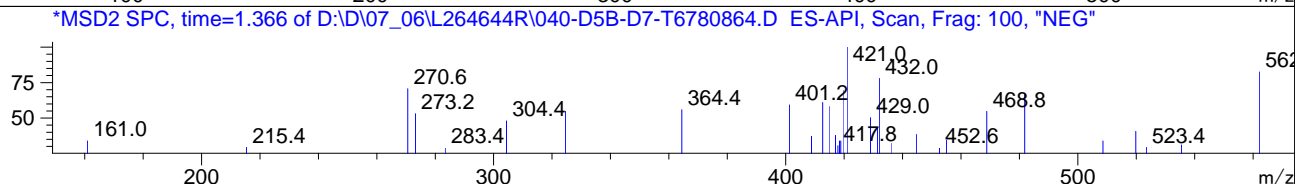

Supplement: Supplementary file 1 — Supplementary Information 1. [file 41598_2024_54655_MOESM1_ESM.zip › Nature SREP/QC_AIMS_files/Proj236.pdf]

MaxPeak: 97.84%  
Ret\_Time: 1.040 min

6340539

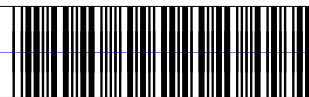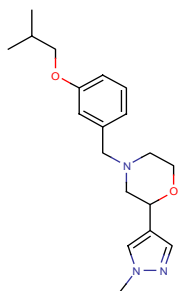

Mol Wt 329.44  
Exact Mass 329.25

| # | Time  | Area% |
|---|-------|-------|
| 1 | 1.040 | 97.84 |
| 2 | 1.433 | 2.16  |

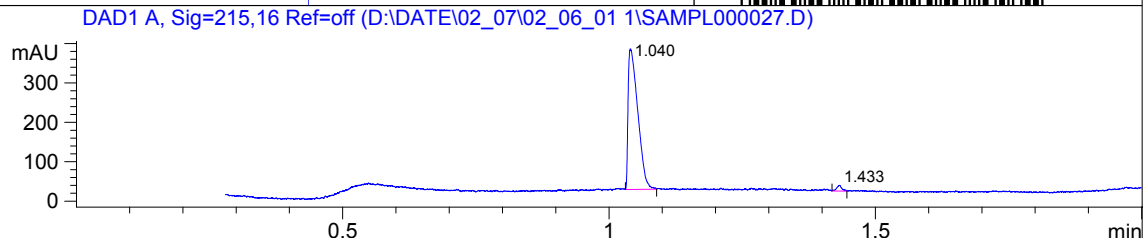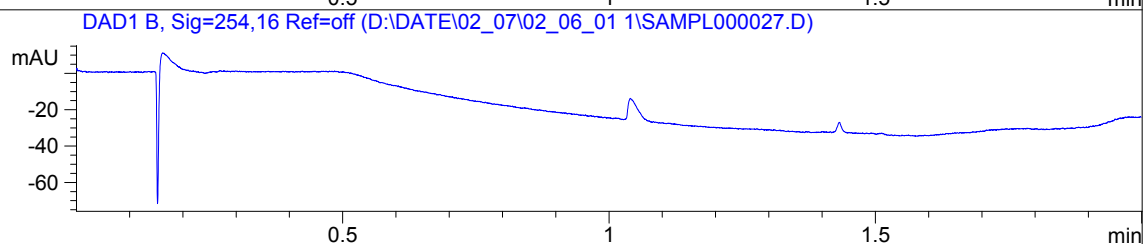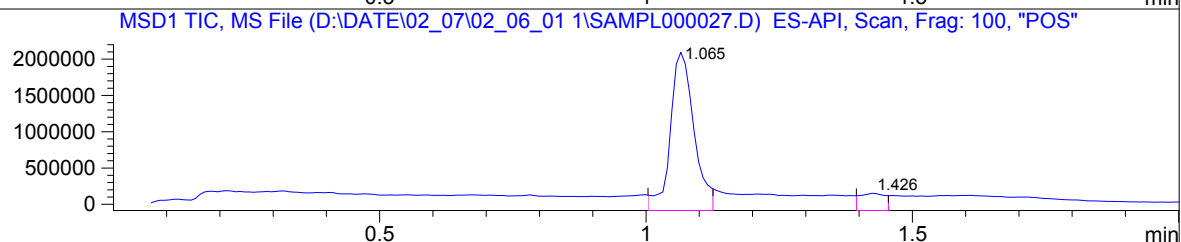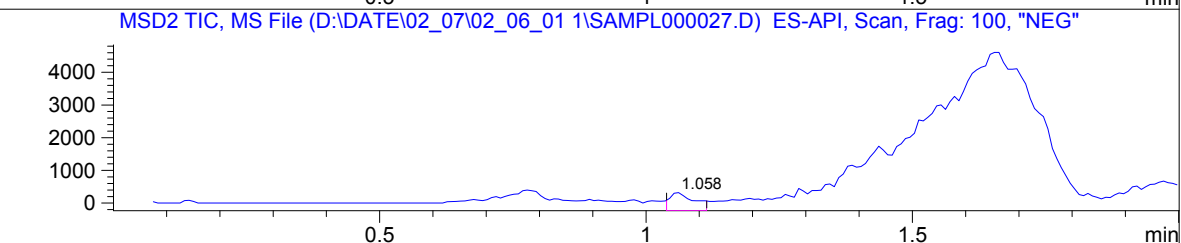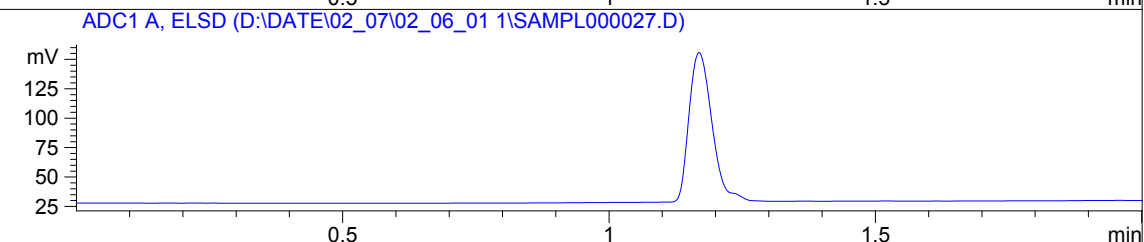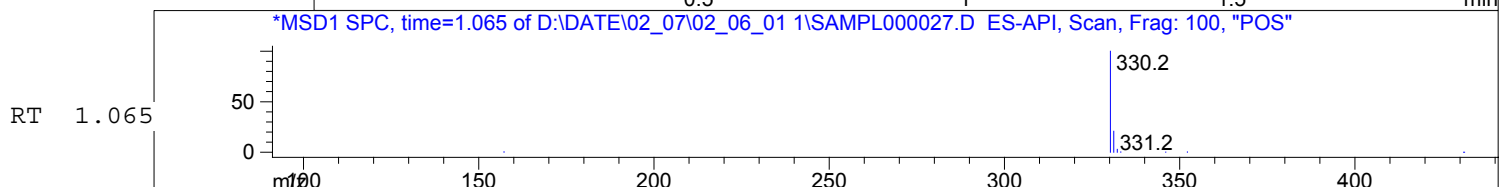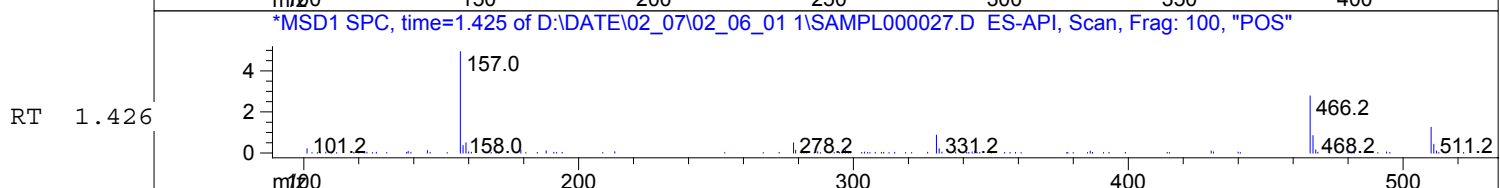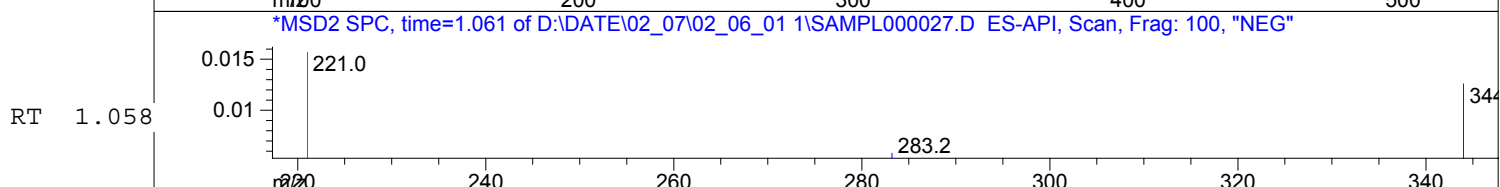

Supplement: Supplementary file 1 — Supplementary Information 1. [file 41598_2024_54655_MOESM1_ESM.zip › Nature SREP/QC_AIMS_files/Proj238.pdf]
