# Supplementary material for: AI is a viable alternative to high throughput screening: a 318-target study
Source: Sci Rep. 2024 Apr 2;14:7526. doi: 10.1038/s41598-024-54655-z (PMC10987645; doi:10.1038/s41598-024-54655-z)
Supplement: Supplementary file 2 — Supplementary Information 2. [file 41598_2024_54655_MOESM2_ESM.pdf]

AI is a Viable Alternative to High Throughput Screening: A 318-target study

The Atomwise AIMS Program*, Izhar Wallach[1ψ]

*Lists of participants and their affiliations appear in the manuscript. [1]Atomwise Inc.

[ψ]corresponding author: izhar@atomwise.com

Supplementary Material

Internal Portfolio Validation

LATS1

Large tumor suppressor homolog 1 (LATS1) is a negative regulator of YAP1 in the Hippo signaling pathway[1]. YAP1 degradation by Hippo pathway kinases, such as LATS1, is pivotal in organ size control and tumor suppression through restricting proliferation and promoting apoptosis[2]. There are numerous indications associating LATS1 with tumor immunogenicity. LATS1 deletion in tumors leads to tumor destruction by enhancing anti-tumor immune responses. Inactivation of the ‘‘tumor suppressor’’ LATS1/2 in tumor cells strongly suppresses tumor growth in immune-competent, but not immune-compromised, mice due to the induction of host anti-tumor immune responses[3]. ATP-competitive inhibitors of LATS kinases cause Yap-dependent proliferation of murine supporting cells in the inner ear, murine cardiomyocytes, and human Muller glia in retinal organoids[4].

Computationally targeting of the LATS1 ATP binding site posed a significant challenge because neither a 3-dimensional structure of the receptor nor any bioactivity binding data were available to us. We used SWISS-MODEL[5,6] to build a homology model of the receptor based on a homologous x-ray crystal structure sharing 42% sequence identity and a resolution of 3.15Å (PDB: 5NCL). We used the screening protocol described above to select a set of 418 diverse scaffolds, all dissimilar to any known kinase inhibitor in our training corpus.

We describe the assay protocol in the Methods section. We tested the 418 scaffolds in a KinomeScan competitive binding assay and identified 76 compounds that reduced positive control binding to <50% at 30uM. Subsequently, 75 of these showed dose-responsive binding behavior with K_D’s ranging from 0.077 - 82 µM (Supplementary Table 8 and Supplementary Table 9). We then chose 8 scaffolds for hit expansion and validation, performing analog expansion as described before, and selected a total of 834 analogs of these scaffolds. These compounds were tested in the KinaseProfiler activity assay. 435 analogs showed >50% inhibition at 50uM, out of which 382 demonstrated DR behavior with IC50s ranging from 0.034 - 76 µM (Supplementary Table 10). In all 8 analog expansions, we achieved sub-µM potencies and malleable structure-activity relationships (SAR) across the series (Supplementary Table 11) as well as improved potencies over the parent hit (Supplementary Figure 4).

VCP

Valosin-containing protein (VCP) is an AAA-type ATPase that governs diverse cellular processes, from the degradation of damaged proteins and organelles to key signaling events and chromatin regulation with thousands of client proteins[7]. VCP is a regulator in the ubiquitin-proteasome system, extracting misfolded proteins and chaperoning them to the proteasome for degradation. Elevated ATPase activity of VCP has been implicated in various neurological and oncological indications, such as inclusion body myopathy with early-onset Paget disease of bone and/or frontotemporal dementia (IBMPFD), amyotrophic lateral sclerosis, melanomas, non-small cell lung carcinoma, and breast cancer[8–12].

In this study, we sought to discover small-molecule inhibitors of VCP^{R155H} mutant responsible for IBMPFD, a rare autosomal dominant disorder. We used the AtomNet model to screen 16B Enamine REAL compounds against a relatively low-resolution crystal structure of VCP (PDB: 6MCK, 3.8Å). We chose a screening site that encompassed both the orthosteric ADP binding site[10,13–15] in the D2 domain and a nearby allosteric site[13,16] (Supplementary Fig. 5). Using the protocol described in the Methods section, we selected a set of 416 scaffolds for testing in VCP^{R155H} ADP-Glo bioluminescence assay[17,18]. Of the 20 scaffolds showing >40% inhibition at 50uM, 19 exhibited dose-responsive behavior, with IC50s ranging from 2.4μM to 99.7uM. ADP competition studies of the top 15 hits yielded 3 competitive, 1 uncompetitive, and 11 non-competitive scaffolds. We advanced 7 scaffolds (3 competitive, 1 uncompetitive, and 3 noncompetitive) to hit expansion based on their potency and the mechanism of action, that is, whether the compound competes with ADP for the binding site (Supplementary Table 12 and Supplementary Table 9). All of our compounds including the allosteric scaffolds were novel, having a maximal similarity of 0.47 (Tanimoto, ECFP4) to published VCP inhibitors.

Assay Protocols

See Supplementary file Assay_details.xlsx

Figures

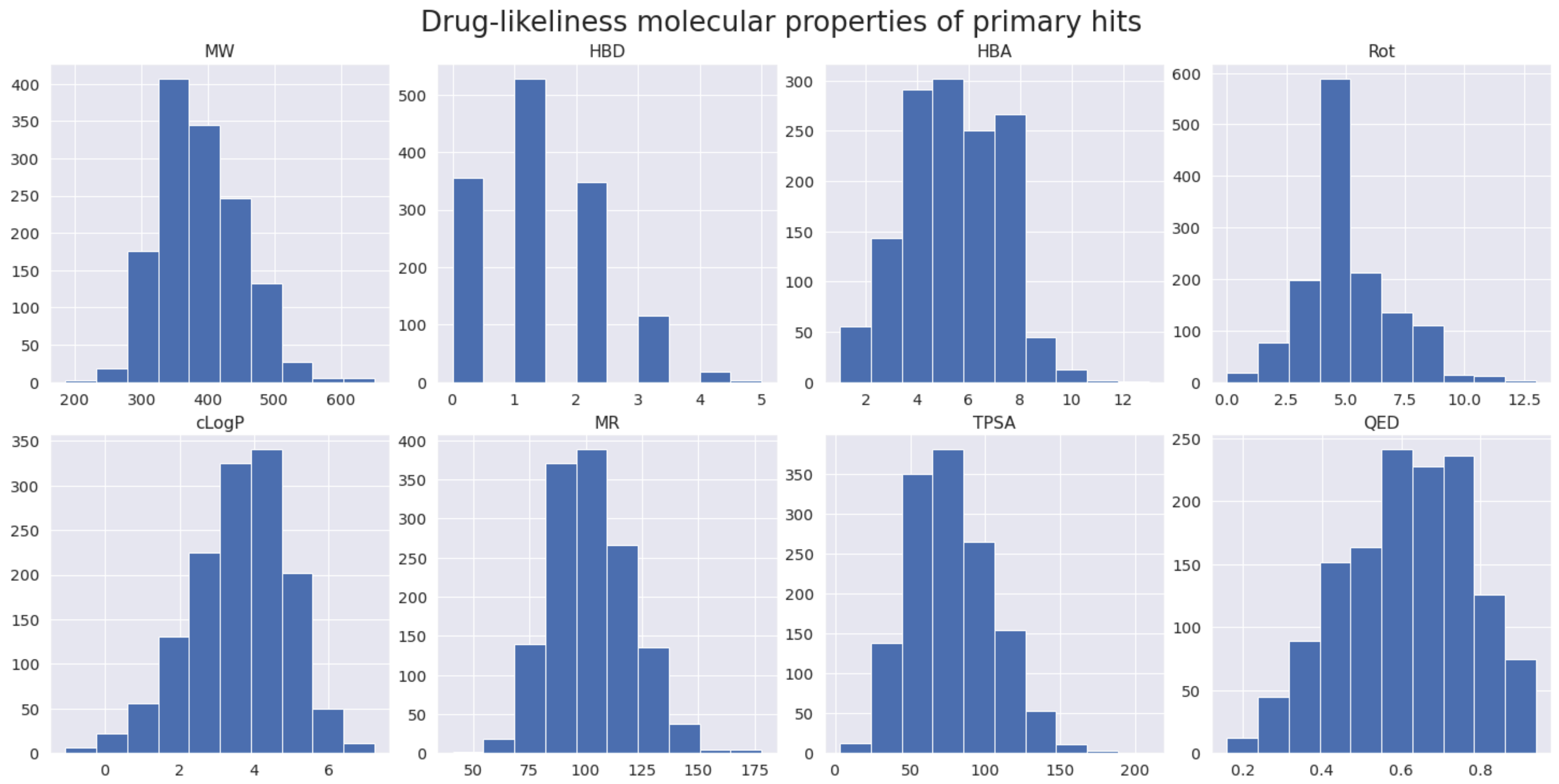

Supplementary Fig. 1. An illustration of the distributions of drug-like properties of the primary hits identified in the 296 AIMS projects. The molecular properties show that the compounds largely obey Lipinski rules and are amenable for further development. The number of hydrogen bond donors (HBD), number of hydrogen bond acceptors (HBA), number of rotational bonds, calculated logP (cLogP), molecular refractivity (MR), topological polar surface area (TPSA), and quantitative estimation of drug-likeness (QED[19]), were calculated using RDkit[20].

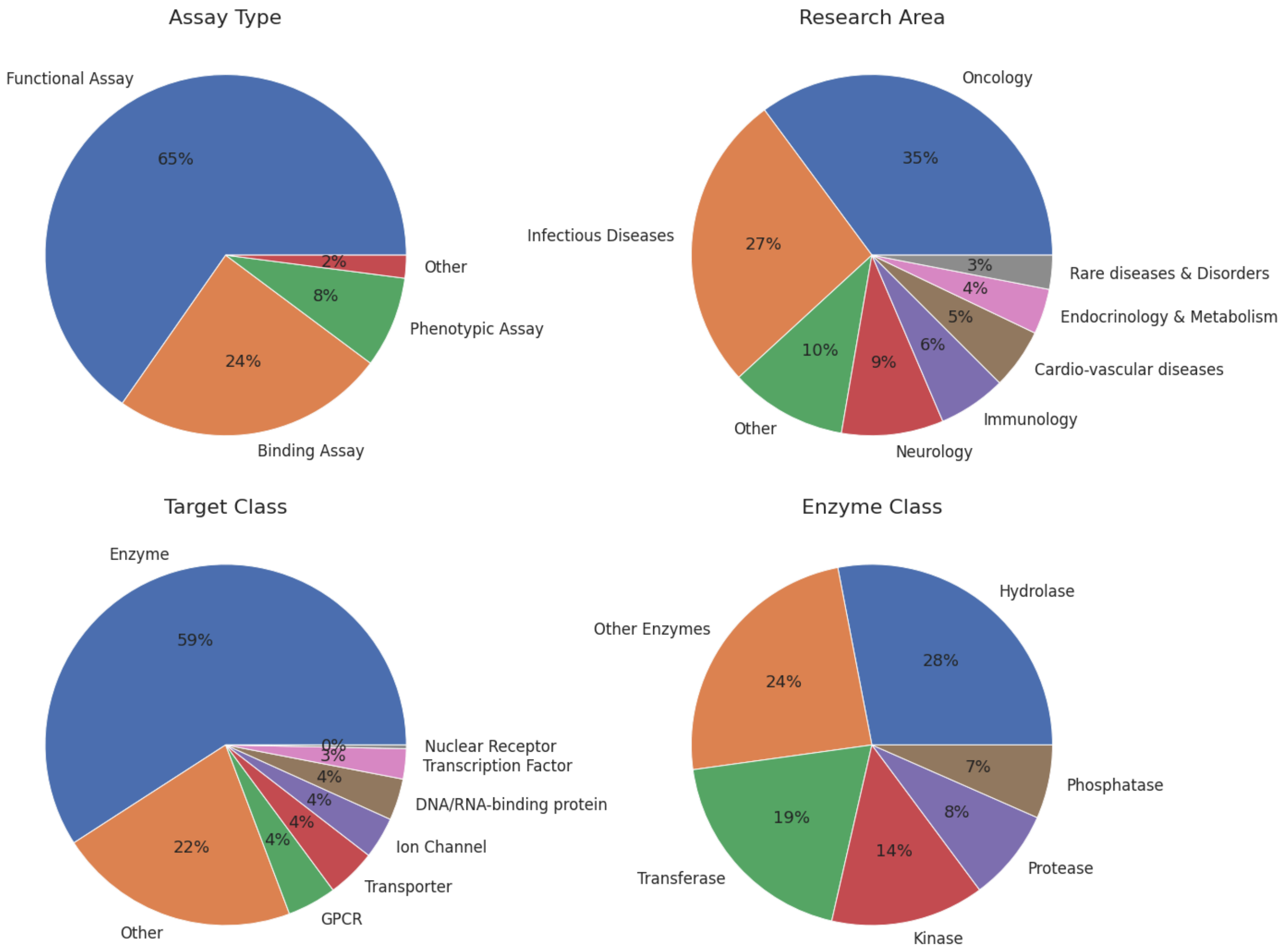

Supplementary Fig. 2. The distributions of 49 dose-response validation projects across assay types used in the primary screen, research area, target class, and further breakdown to enzyme class when applicable. The similarities of these distributions to that of the AIMS projects suggest that the DR validation set well represents the over set of AIMS projects.

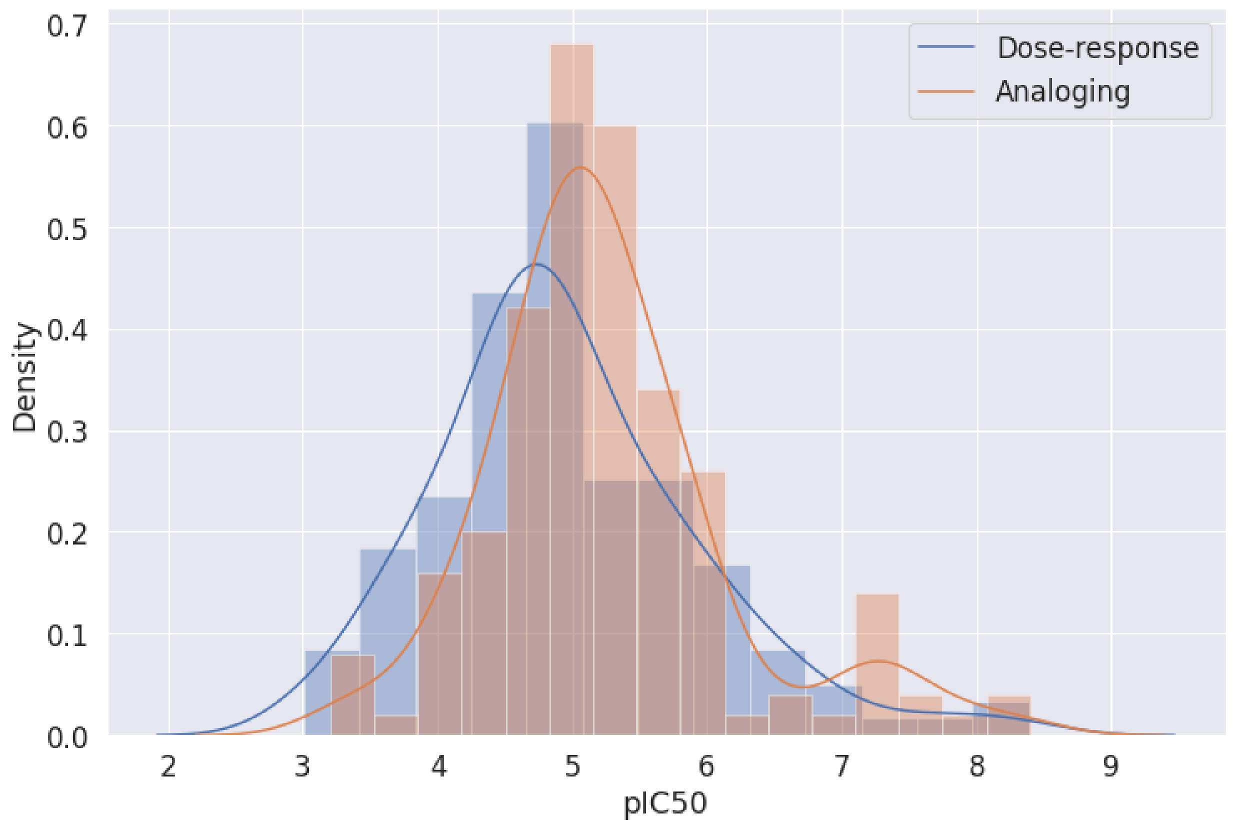

Supplementary Fig. 3. The distribution of potencies obtained in the dose-response and the analoging validation studies. As expected, the potencies tend to increase due to the exploration of analog space around the active scaffolds. The median DR activity of the 144 validated analogs was 7.4μM compared to the median of 15.4μM of the parent compound.

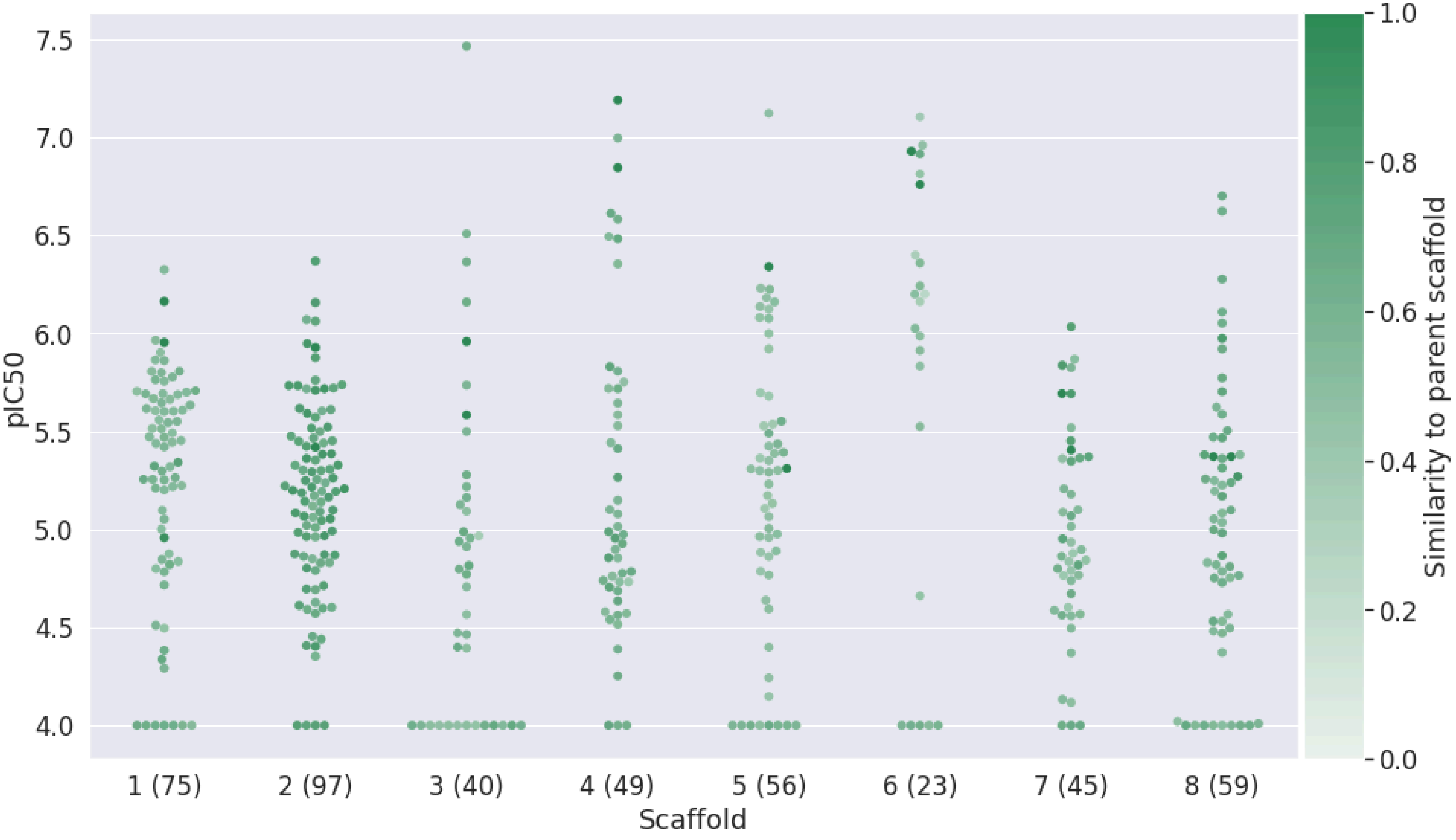

Supplementary Fig. 4. An illustration of the distribution of potencies for each of the analog sets selected for 8 LATS1 scaffolds identified in the first round of testing. In all 8 expansions, we achieved sub-μM potencies and malleable structure-activity relation (SAR). The color of the data points indicates the similarity of the analogs to their parent scaffold (Tanimoto similarity, ECFP4). The number of analogs of each scaffold is given in parentheses, e.g. there were 75 analogs of Scaffold 1.

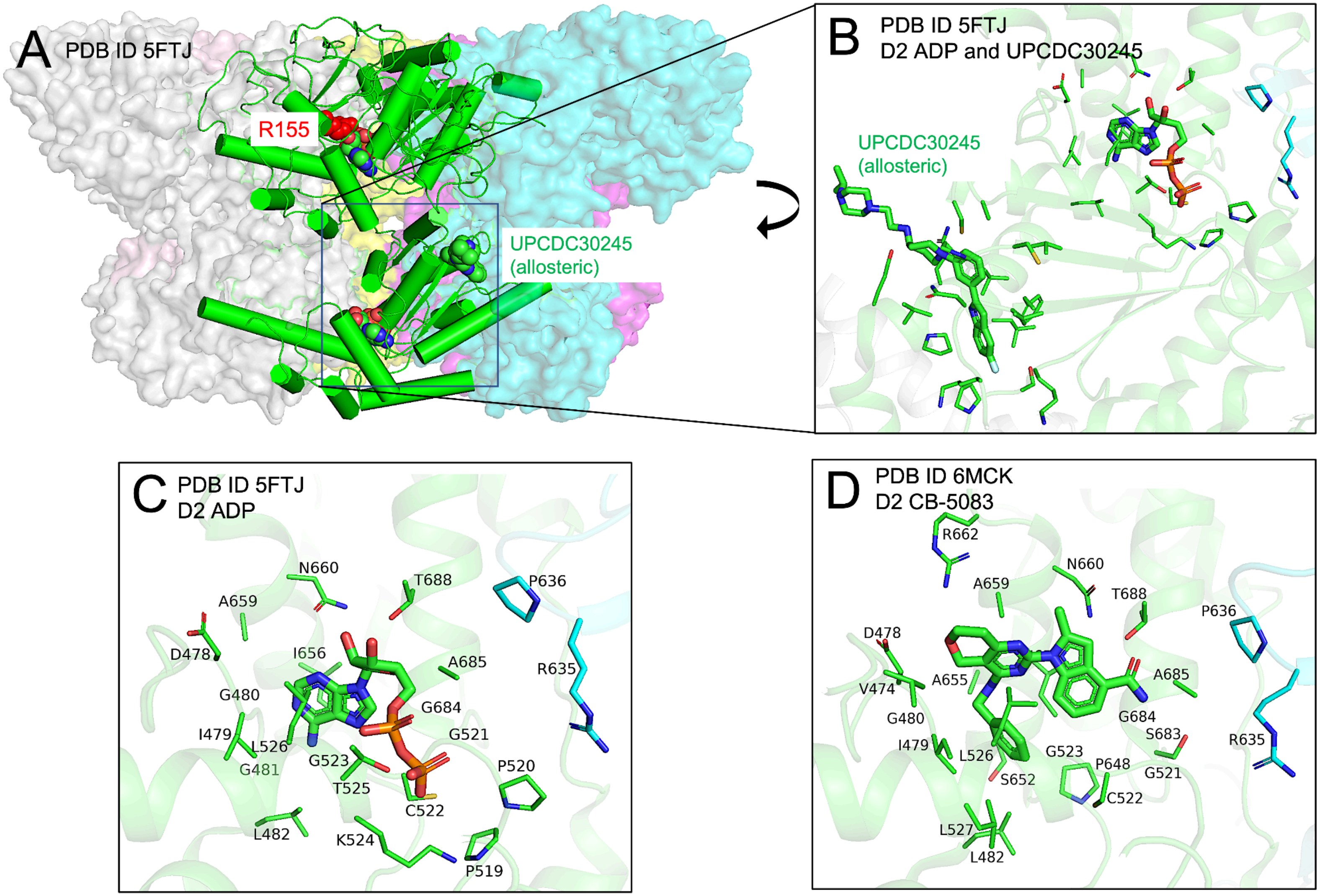

A
PDB ID 5FTJ
R155
UPCDC30245
(allosteric)
B
PDB ID 5FTJ
D2 ADP and UPCDC30245
UPCDC30245
(allosteric)
C
PDB ID 5FTJ
D2 ADP
N660
T688
P636
A659
D478
I656
A685
R635
G480
G684
I479
L526
G521
G481
G523
P520
T525
C522
L482
K524
P519
D
PDB ID 6MCK
D2 CB-5083
R662
N660
A659
T688
P636
D478
A655
A685
V474
G480
G684
S683
I479
R635
L526
G523
P648
G521
S652
C522
L527
L482

Supplementary Fig. 5. Illustration of the binding site of VCP used in the virtual screening. (A) The side view of VCP hexamer including N-terminal (residues 2-187), D1 (residues 209-462), and D2 (481-763) domains from PDB ID 5FTJ. The site of R155H mutation responsible for IBMPFD is adjacent to the D1 ADP site and is highlighted in red spheres on panel A. Each protomer of the hexamer is represented with a different color. D2 ADP and UPCDC30245 allosteric ligand-binding sites as well as a separate close-up view of D2 ADP site are displayed on (B) and (C), respectively. (D) The specific structure of the site used in the virtual screening is available via PDB ID 6MCK with bound CB-5083, an ADP-competitive inhibitor of VCP binding to D2 ADP site.

Tables

Target (Uniprot ID)	Identified hit	Potency (µM)	Nearest Neighbor (NN)	NN Similarity (ECFP4, Tanimoto)	NN potency (µM)
Miro1[21] (Q8IXI2)	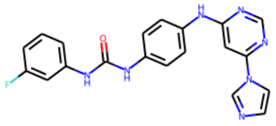	7.8	NA	NA	NA
pCD163[22] (Q2VL90)	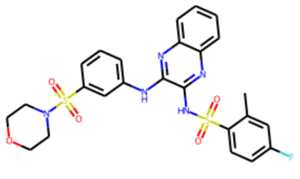	7.5	NA	NA	NA

TAAR5[23] (Q5QD14)	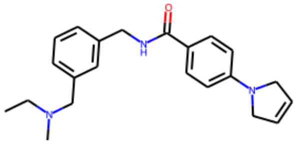	1.1	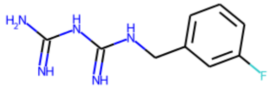	0.27	5
SHIP1[24] (Q92835)	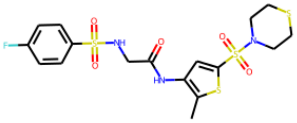	0.2	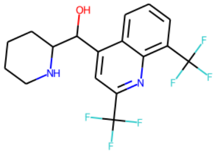	0.15	NA
SPOP and OTUD7A[25] (Q8TE49)	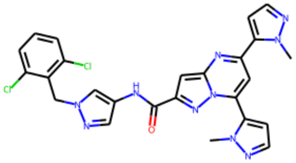	1.1	NA	NA	NA
Aspartate N-acetyltransferase[26] (Q8N9F0)	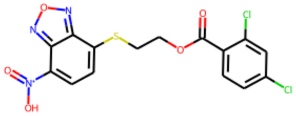	0.40	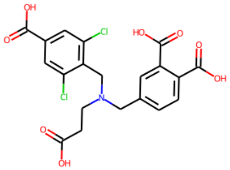	0.16	0.6
LATS1 (O95835)	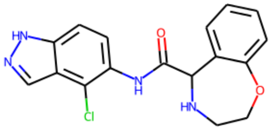	0.077	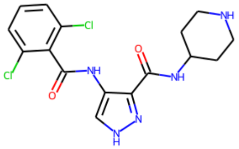	0.23	10
VCP R155H (P55072)	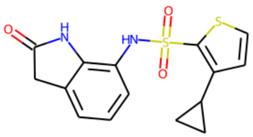	11.3	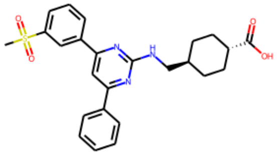	0.16	0.23

COB, CAWG (P0C8L0, C4YQJ6)	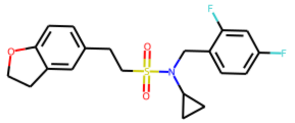	2.5	NA	NA	NA
DXR (O96693)	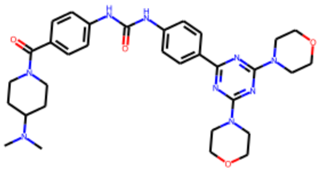	1.67	NA	NA	NA
RRM2 (P31350)	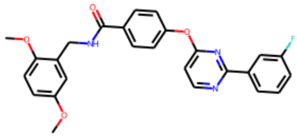	5	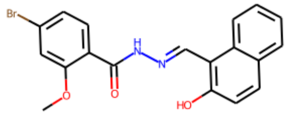	0.21	33.884
VP1 (Q9YLJ3)	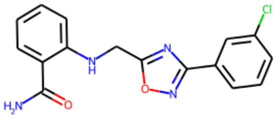	1.7	NA	NA	NA
DNMT1 (P26358)	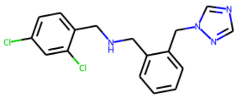	34.6	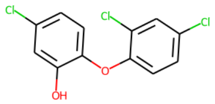	0.21	7.586
CREBBP (Q92793)	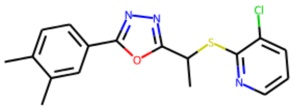	6	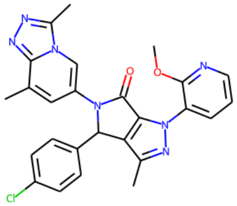	0.20	37.91

KRIT1/ HEG1 (O00522/ Q9ULI3)	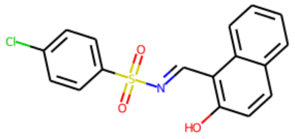	21	NA	NA	NA
EPHX2 (P34913)	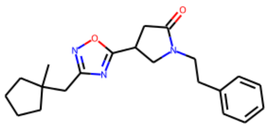	3.3	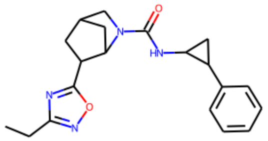	0.31	0.095

Supplementary Table 1. Examples of bioactive compounds discovered using the AtomNet screening system. Compounds for Miro1, pCD163, TAAR5, SHIP1, SPOP and OTUD7A, and Aspartate N-acetyltransferase were previously published. For each compound, we provide the Nearest Neighbor (NN) compound in the training data as defined by Tanimoto distance over ECFP4 fingerprints, and the corresponding annotated potency against the target of interest. "NA" denotes that the target lacked any active molecules in our training data or was labeled as active but without quantitative dose response potency.

Target	Structure	IC50 (μM)	Hill Slope	R^2	Dose-response curve
ASAH1	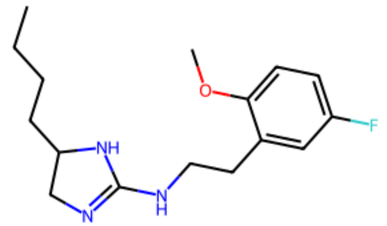	16.60	1.06	0.94	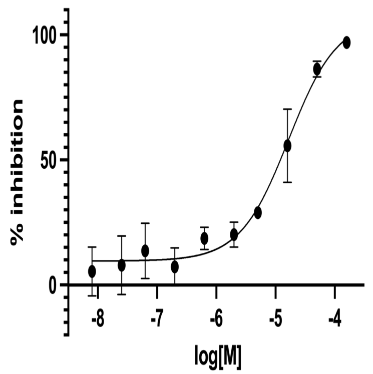
AXL	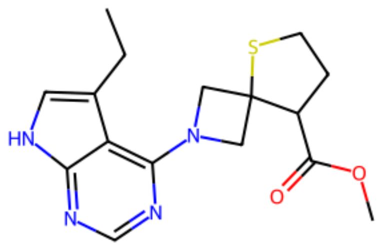	4.73	1.16	0.92	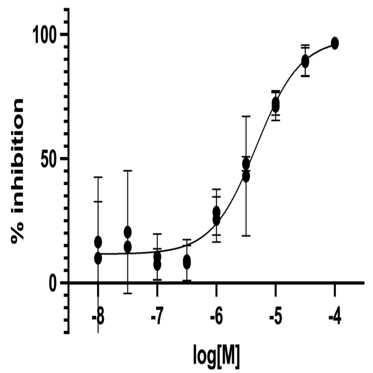

CDK5	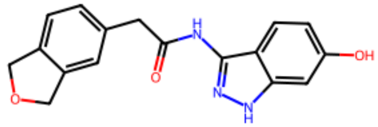	0.61	1.13	0.99	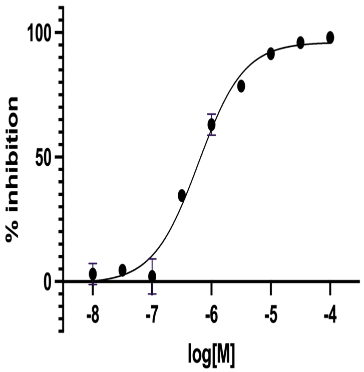
GFPT1	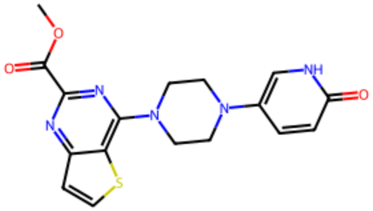	38.50	2.17	0.97	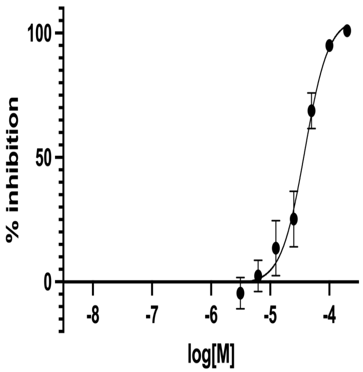

KCNT1	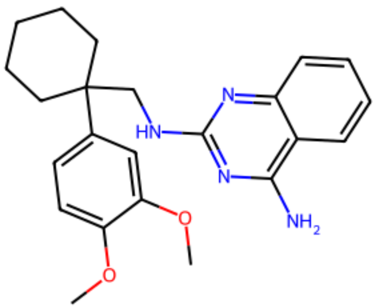	1.50	1.49	0.87	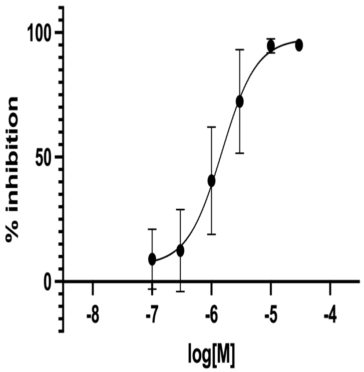
KDM6A	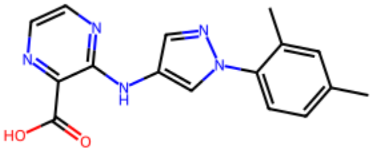	46.20	1.90	0.98	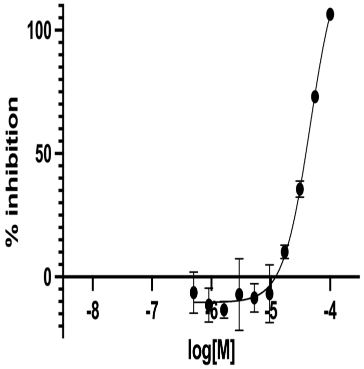

MC2R	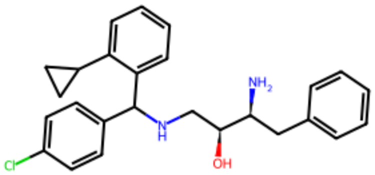	27.30	3.77	0.98	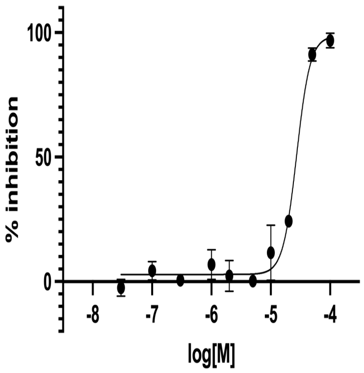
NT5E	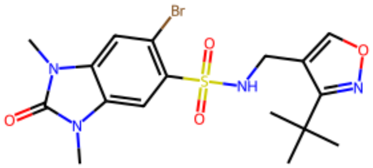	28.40	0.91	0.98	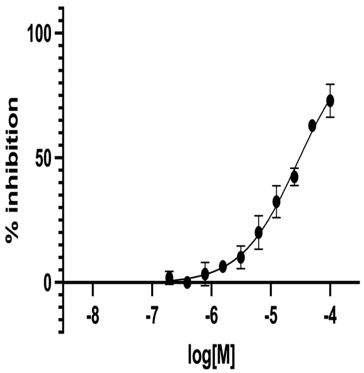

PARP14	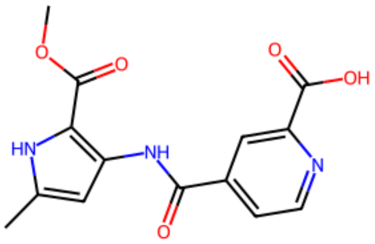	3.82	0.95	0.94	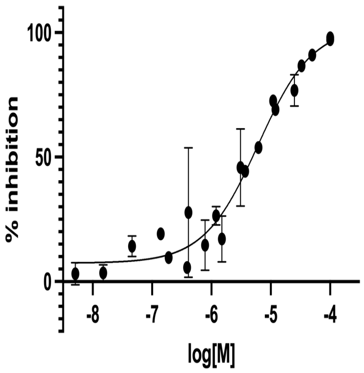
POLQ	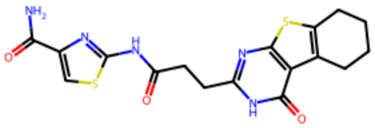	14.50	3.64	1.00	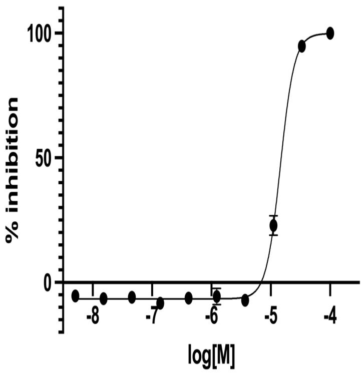

PPARA	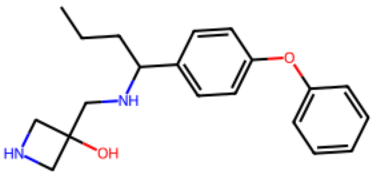	23.90	1.94	0.81	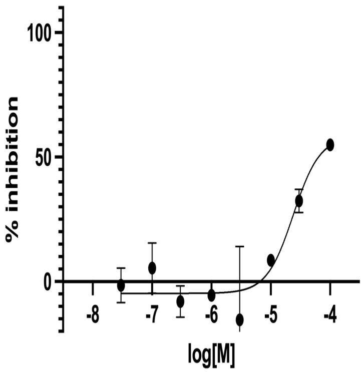
PPM1D	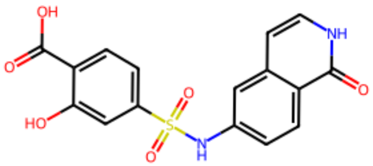	4.33	1.01	0.98	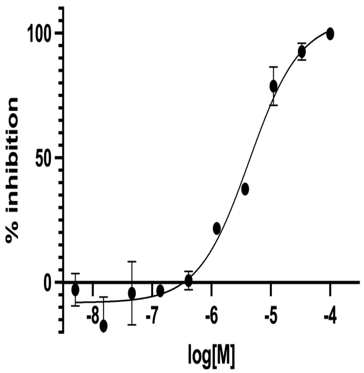

PRMT5	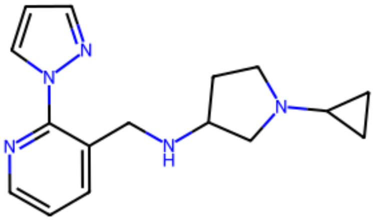	32.30	1.71	0.94	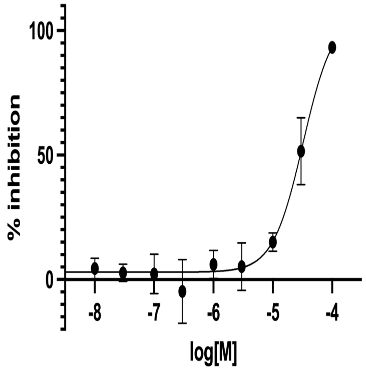
PRODH2	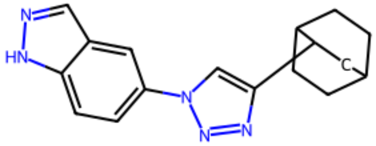	26.30	0.78	0.98	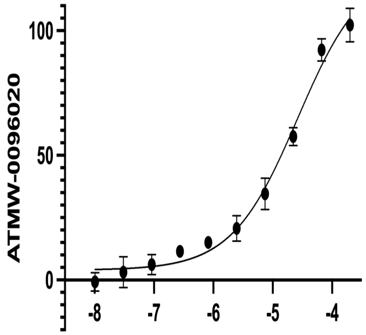

Supplementary Table 2. Dose-response curves of representative bioactive compounds reported in the Internal Portfolio Validation section. Curve plots, hill slopes, R^2, and IC50 or Kd values were generated using Prism[1].

[1] GraphPad Software, San Diego, California USA, www.graphpad.com

Project Number	UniProt ID	# Compounds Tested	No. Hits	Hit rate (%)	Assay Type	Model Type	Target Class	Enzyme Class	Research Area
001	Q9NZQ7	82	4	4.88	Binding	X-ray	Other	-	Oncology
002	Q9UBT2, Q9UBE0	73	2	2.74	Binding	X-ray	Enzyme	Other Enzymes	Oncology
003	Q9HBX9	141	37	26.2	Functional	Homology	GPCR	-	Other
004	Q96KS0	72	3	4.17	Phenotypic	Homology	Enzyme	Other Enzymes	Oncology
005	Q2VL90	90	1	1.11	Functional	X-ray	Other	-	Other
006	Q8IXL6	72	0	0.0	Functional	Homology	Enzyme	Kinase	Oncology
007	Q8I5R7	74	8	10.8	Functional	X-ray	Enzyme	Other Enzymes	Infectious Diseases
008	P35749	72	9	12.5	Functional	Homology	Other	-	Endocrinology & Metabolism
009	Q9Y2P5	77	0	0.0	Functional	Homology	Enzyme	Other Enzymes	Endocrinology & Metabolism
010	P28827	76	12	15.8	Phenotypic	Homology	Other	Phosphatase	Oncology
011	O75533	69	0	0.0	Phenotypic	X-ray	Other	-	Oncology
012	Q8NFI3	72	0	0.0	Functional	Homology	Enzyme	Hydrolase	Rare diseases & Disorders
013	Q12931	73	0	0.0	Binding	X-ray	Enzyme	Hydrolase	Oncology
014	Q14508	72	1	1.39	Phenotypic	Homology	Other	-	Oncology

015	Q13886	77	0	0.0	Phenotypic	Homology	Transcription Factor	-	Neurology
016	Q9UL51	55	3	5.45	Phenotypic	X-ray	Ion Channel	-	Other
017	Q92743	83	6	7.23	Binding	X-ray	Enzyme	Protease	Other
018	P14625	72	0	0.0	Binding	X-ray	Enzyme	Hydrolase	Oncology
019	Q9NVD7	76	15	19.7	Phenotypic	X-ray	Other	-	Cardio-vascula r diseases
020	Q8NBL1	75	1	1.33	Functional	Homology	Enzyme	Transferase	Other
021	Q12852	73	0	0.0	Phenotypic	X-ray	Enzyme	Kinase	Other
022	Q9Y623	88	4	4.55	Functional	Homology	Other	-	Endocrinology & Metabolism
023	Q8TE49	73	2	2.74	Phenotypic	Homology	Enzyme	Hydrolase	Other
024	Q8WZ42	76	7	9.21	Binding	X-ray	Enzyme	Kinase	Other
025	P9WJJ5	76	6	7.89	Functional	X-ray	Enzyme	Transferase	Infectious Diseases
026	Q92835	76	14	18.4	Functional	Homology	Enzyme	Phosphatase	Immunology
027	P07900	73	0	0.0	Binding	X-ray	Enzyme	Hydrolase	Oncology
028	P28827	72	14	19.4	Functional	X-ray	Enzyme	Phosphatase	Oncology
029	P25779	78	5	6.41	Other	X-ray	Enzyme	Hydrolase	Infectious Diseases
030	P44046	77	0	0.0	Functional	X-ray	Enzyme	Hydrolase	Infectious Diseases
031	P04156	81	0	0.0	Binding	Homology	Other	-	Rare diseases & Disorders

032	Q8IXI2	80	4	5.0	Phenotypic	X-ray	Enzyme	Hydrolase	Rare diseases & Disorders
033	P32322	82	0	0.0	Functional	X-ray	Enzyme	Other Enzymes	Oncology
034	C4YCM5	77	0	0.0	Functional	X-ray	Enzyme	Kinase	Infectious Diseases
035	Q13936	76	2	2.63	Binding	Homology	Ion Channel	-	Cardio-vascula r diseases
036	P49902	87	2	2.3	Functional	X-ray	Enzyme	Hydrolase	Oncology
037	Q4WUS9	71	2	2.82	Functional	X-ray	Enzyme	Transferase	Other
038	O60930	85	0	0.0	Functional	X-ray	Enzyme	Hydrolase	Oncology
039	Q9BRQ0	85	19	22.4	Binding	X-ray	Other	-	Oncology
040	Q7PRZ8	64	3	4.69	Functional	Homology	GPCR	-	Infectious Diseases
041	Q13009	80	8	10.0	Binding	X-ray	Other	-	Oncology
042	Q5TDH0	65	0	0.0	Functional	X-ray	Enzyme	Protease	Oncology
043	P09237	72	18	25.0	Binding	X-ray	Enzyme	Hydrolase	Oncology
044	P28223	57	6	10.5	Functional	Homology	GPCR	-	Infectious Diseases
045	Q04IN8	62	4	6.45	Other	X-ray	Other	-	Infectious Diseases
046	P18031	130	13	10.0	Functional	X-ray	Enzyme	Phosphatase	Endocrinology & Metabolism

047	Q7KWJ5	61	0	0.0	Binding	Homology	Transporter	-	Infectious Diseases
048	P05546, P00734	81	0	0.0	Functional	X-ray	Enzyme	Protease	Cardio-vascula r diseases
049	P50148	215	1	0.47	Binding	X-ray	Other	-	Immunology
050	O00308	83	2	2.41	Binding	Homology	Enzyme	Transferase	Oncology
051	P28827	77	17	22.1	Phenotypic	X-ray	Enzyme	Phosphatase	Oncology
052	P12004	59	5	8.47	Binding	X-ray	Other	-	Oncology
053	P55072	139	15	10.8	Functional	X-ray	Enzyme	Hydrolase	Other
054	Q5F7X0	155	0	0.0	Functional	Homology	Enzyme	Other Enzymes	Infectious Diseases
055	Q05086	141	19	13.5	Functional	X-ray	Enzyme	Transferase	Oncology
056	Q66479	77	20	26.0	Phenotypic	X-ray	Enzyme	Hydrolase	Infectious Diseases
057	O96693	76	11	14.5	Phenotypic	X-ray	Enzyme	Other Enzymes	Infectious Diseases
058	Q5HEB7	73	0	0.0	Phenotypic	X-ray	Enzyme	Other Enzymes	Infectious Diseases
059	Q16740	54	9	16.7	Functional	X-ray	Enzyme	Hydrolase	Endocrinology & Metabolism
060	O15178	57	6	10.5	Binding	X-ray	Transcription Factor	-	Oncology
061	Q9JJX6	73	6	8.22	Other	Homology	Ion Channel	-	Cardio-vascula r diseases

062	P31350	82	3	3.66	Phenotypic	X-ray	Enzyme	Other Enzymes	Oncology
063	O95631	68	4	5.88	Binding	X-ray	Other	-	Oncology
064	P9WHW5	57	0	0.0	Functional	X-ray	Enzyme	Phosphatase	Infectious Diseases
065	P11021	79	0	0.0	Functional	X-ray	Enzyme	Hydrolase	Cardio-vascula r diseases
066	Q13586	62	4	6.45	Functional	X-ray	Transporter	-	Immunology
067	Q969H0	73	9	12.3	Functional	X-ray	Other	-	Oncology
068	Q8IEW2	49	5	10.2	Phenotypic	Homology	Other	-	Infectious Diseases
069	P22513	63	0	0.0	Phenotypic	X-ray	Enzyme	Other Enzymes	Infectious Diseases
070	Q7DD94	80	0	0.0	Phenotypic	X-ray	Enzyme	Transferase	Infectious Diseases
071	Q9Y265, Q9Y230	81	0	0.0	Functional	X-ray	Enzyme	Hydrolase	Oncology
072	Q763K9	59	8	13.6	Functional	X-ray	Enzyme	Transferase	Infectious Diseases
073	Q9NQ25	69	4	5.8	Other	Homology	Other	-	Immunology
074	Q8RQE8	78	0	0.0	Functional	Cryo-EM	Enzyme	Transferase	Infectious Diseases
075	O75385	58	6	10.3	Functional	X-ray	Enzyme	Kinase	Oncology
076	Q9GZU7	60	0	0.0	Functional	X-ray	Enzyme	Hydrolase	Oncology

077	P22314	75	5	6.67	Functional	X-ray	Enzyme	Other Enzymes	Neurology
078	O75365	69	2	2.9	Functional	X-ray	Enzyme	Hydrolase	Oncology
079	Q9Y345	96	2	2.08	Functional	Homology	Transporter	-	Neurology
080	Q07812	87	1	1.15	Other	X-ray	Other	-	Neurology
081	P62873	76	5	6.58	Binding	X-ray	Other	-	Other
082	P25789	65	0	0.0	Functional	Cryo-EM	Enzyme	Hydrolase	Neurology
083	Q9NY59	78	0	0.0	Functional	X-ray	Enzyme	Hydrolase	Oncology
084	O43353	56	0	0.0	Other	X-ray	Enzyme	Kinase	Immunology
085	Q9Y572	54	2	3.7	Functional	Homology	Enzyme	Kinase	Immunology
086	P9WGZ1	167	3	1.8	Binding	X-ray	Enzyme	Transferase	Infectious Diseases
087	Q13526	81	0	0.0	Phenotypic	X-ray	Enzyme	Other Enzymes	Oncology
088	P32783	58	0	0.0	Binding	X-ray	Enzyme	Transferase	Infectious Diseases
089	Q9UBN7	86	0	0.0	Functional	X-ray	Enzyme	Hydrolase	Endocrinology & Metabolism
090	Q9UKK9	51	1	1.96	Functional	X-ray	Enzyme	Transferase	Oncology
091	Q12923	75	4	5.33	Functional	X-ray	Enzyme	Phosphatase	Other
092	P30613	67	7	10.4	Functional	X-ray	Enzyme	Kinase	Oncology
093	Q16740	84	0	0.0	Functional	X-ray	Enzyme	Hydrolase	Oncology
094	P9WKI7	54	7	13.0	Functional	X-ray	Enzyme	Other Enzymes	Infectious Diseases

095	Q91MB8	72	5	6.94	Functional	X-ray	Other	-	Infectious Diseases
096	Q9Y251	45	9	20.0	Functional	X-ray	Enzyme	Hydrolase	Oncology
097	P14340	76	2	2.63	Functional	Homology	Enzyme	Hydrolase	Infectious Diseases
098	O60603, Q15399	73	1	1.37	Phenotypic	X-ray	Other	-	Immunology
099	P9WPC3	72	2	2.78	Functional	X-ray	Enzyme	Hydrolase	Infectious Diseases
100	O15294	57	2	3.51	Functional	X-ray	Enzyme	Transferase	Oncology
101	P18177	46	0	0.0	Functional	X-ray	Enzyme	Hydrolase	Infectious Diseases
102	P32322	93	0	0.0	Functional	X-ray	Enzyme	Other Enzymes	Oncology
103	B1LMQ5	82	7	8.54	Phenotypic	X-ray	Enzyme	Hydrolase	Infectious Diseases
104	P15056	60	2	3.33	Binding	X-ray	Enzyme	Kinase	Oncology
105	P31153	96	0	0.0	Functional	X-ray	Enzyme	Transferase	Oncology
106	P26358	66	2	3.03	Binding	X-ray	Enzyme	Transferase	Oncology
107	P11086	64	0	0.0	Functional	X-ray	Enzyme	Transferase	Other
108	Q99ZW2	80	3	3.75	Functional	Homology	Enzyme	Hydrolase	Other
109	Q9NRL2	74	5	6.76	Functional	X-ray	Other	-	Rare diseases & Disorders
110	P25779	94	2	2.13	Phenotypic	X-ray	Enzyme	Hydrolase	Other

111	Q02880	63	2	3.17	Functional	X-ray	Enzyme	Other Enzymes	Other
112	Q9H1Y0	67	4	5.97	Phenotypic	X-ray	Other	-	Oncology
113	Q9Y4P1	72	0	0.0	Phenotypic	X-ray	Enzyme	Hydrolase	Oncology
114	Q8TDX7	46	0	0.0	Phenotypic	X-ray	Enzyme	Kinase	Immunology
115	Q8FCR6	83	9	10.8	Binding	X-ray	Enzyme	Other Enzymes	Infectious Diseases
116	P63092	63	0	0.0	Functional	Homology	Other	-	Endocrinology & Metabolism
117	Q12834	68	0	0.0	Phenotypic	X-ray	Other	-	Oncology
118	Q13257	77	0	0.0	Phenotypic	X-ray	Other	-	Oncology
119	Q96HS1	51	1	1.96	Functional	X-ray	Enzyme	Hydrolase	Neurology
120	P05121	65	13	20.0	Functional	X-ray	Other	-	Rare diseases & Disorders
121	Q9BUX1	73	7	9.59	Functional	Homology	Enzyme	Other Enzymes	Cardio-vascula r diseases
122	P38398	74	0	0.0	Phenotypic	X-ray	Other	-	Oncology
123	O14929	94	10	10.6	Functional	X-ray	Enzyme	Transferase	Oncology
124	P32322	80	4	5.0	Functional	X-ray	Enzyme	Other Enzymes	Oncology
125	P21554	95	0	0.0	Functional	Cryo-EM	GPCR	-	Neurology
126	Q96LB1	71	1	1.41	Functional	Homology	GPCR	-	Immunology
127	P9WQ17	94	6	6.38	Functional	X-ray	Enzyme	Transferase	Infectious Diseases

128	Q9NRX4	79	0	0.0	Functional	X-ray	Enzyme	Phosphatase	Oncology
129	Q9UF12	94	0	0.0	Functional	Homology	Enzyme	Other Enzymes	Other
130	Q5CYE4	94	5	5.32	Phenotypic	X-ray	Other	Other Enzymes	Infectious Diseases
131	P03418	96	0	0.0	Binding	X-ray	Other	-	Infectious Diseases
132	Q5TKA1, Q52LA3	79	0	0.0	Functional	X-ray	Other	-	Oncology
133	P9WI81	94	0	0.0	Phenotypic	X-ray	Enzyme	Kinase	Infectious Diseases
134	P09874	94	6	6.38	Binding	X-ray	Enzyme	Transferase	Oncology
135	P55072	87	14	16.1	Functional	X-ray	Enzyme	Hydrolase	Oncology
136	O60264	83	4	4.82	Other	X-ray	Enzyme	Hydrolase	Rare diseases & Disorders
137	P14867	75	0	0.0	Functional	X-ray	Ion Channel	-	Other
138	E9QYP0	94	0	0.0	Functional	X-ray	Enzyme	Other Enzymes	Infectious Diseases
139	P9WG47	94	0	0.0	Functional	X-ray	Enzyme	Transferase	Infectious Diseases
140	Q15303	94	0	0.0	Functional	X-ray	Enzyme	Kinase	Cardio-vascula r diseases
141	P31483	83	2	2.41	Phenotypic	Homology	DNA/RNA-bin ding protein	-	Neurology

142	P12931	82	5	6.1	Functional	X-ray	Enzyme	Kinase	Oncology
143	P0A031	96	10	10.4	Functional	X-ray	Other	-	Infectious Diseases
144	P34913	85	6	7.06	Functional	X-ray	Enzyme	Hydrolase	Other
145	P9WG49	96	4	4.17	Functional	X-ray	Enzyme	Other Enzymes	Infectious Diseases
146	Q9NZC2	82	11	13.4	Binding	Homology	Nuclear Receptor	-	Neurology
147	P49354, P49356	177	14	7.91	Functional	X-ray	Enzyme	Transferase	Neurology
148	Q15717	83	1	1.2	Binding	X-ray	DNA/RNA-binding protein	-	Neurology
149	Q9L5C8	94	0	0.0	Functional	X-ray	Enzyme	Hydrolase	Infectious Diseases
150	P31539	84	6	7.14	Functional	Cryo-EM	Other	-	Neurology
151	O15554	82	2	2.44	Functional	Cryo-EM	Ion Channel	-	Immunology
152	Q5W0Z9	64	1	1.56	Binding	X-ray	Enzyme	Transferase	Cardio-vascular diseases
153	P55851	182	8	4.4	Binding	Homology	Transporter	-	Oncology
154	P42345	94	30	31.9	Binding	Cryo-EM	Enzyme	Kinase	Oncology
155	Q9YLJ3	84	2	2.38	Phenotypic	X-ray	Other	-	Infectious Diseases
156	Q99836	94	9	9.57	Functional	Homology	Other	-	Oncology

157	P04637	83	3	3.61	Phenotypic	X-ray	DNA/RNA-binding protein	-	Oncology
158	P9WNM7	94	4	4.26	Phenotypic	Homology	DNA/RNA-binding protein	-	Infectious Diseases
159	I6Y9J2	94	4	4.26	Phenotypic	X-ray	Enzyme	Transferase	Infectious Diseases
160	Q15118	164	2	1.22	Functional	Homology	Enzyme	Kinase	Oncology
161	P0DTC2	100	20	20.0	Functional	X-ray	Other	-	Infectious Diseases
162	P0DTC2	190	1	0.53	Binding	X-ray	Enzyme	Protease	Infectious Diseases
163	P0DTD1	94	11	11.7	Functional	Homology	Enzyme	Protease	Infectious Diseases
164	P0DTD1	94	15	16.0	Functional	Cryo-EM	Transporter	Transferase	Infectious Diseases
165	Q8ZNV8	80	2	2.5	Phenotypic	Homology	Transporter	-	Infectious Diseases
166	P68135	83	7	8.43	Functional	X-ray	Other	-	Oncology
167	O14842	111	29	26.1	Phenotypic	X-ray	GPCR	-	Endocrinology & Metabolism
168	P15559	36	8	22.2	Functional	X-ray	Enzyme	Other Enzymes	Oncology
169	P52270	68	20	29.4	Functional	X-ray	Enzyme	Other Enzymes	Infectious Diseases
170	P43490	94	3	3.19	Phenotypic	X-ray	Enzyme	Transferase	Oncology

171	Q99538	86	4	4.65	Functional	X-ray	Enzyme	Hydrolase	Neurology
172	P28335	94	5	5.32	Functional	X-ray	GPCR	-	Neurology
173	P12931	100	0	0.0	Functional	X-ray	Enzyme	Kinase	Oncology
174	A0A6L0XSJ0	83	8	9.64	Phenotypic	Cryo-EM	Enzyme	Protease	Infectious Diseases
175	Q9H4A3	94	0	0.0	Binding	X-ray	Enzyme	Kinase	Oncology
176	P0C0V0	87	3	3.45	Functional	X-ray	Enzyme	Protease	Infectious Diseases
177	Q9H9Z2	58	9	15.5	Binding	X-ray	DNA/RNA-binding protein	-	Oncology
178	Q9R1M7	159	17	10.7	Functional	X-ray	Ion Channel	-	Neurology
179	Q9NQU5	94	0	0.0	Functional	X-ray	Enzyme	Kinase	Oncology
180	A0A1D3GEK4	74	3	4.05	Phenotypic	Homology	Enzyme	Protease	Infectious Diseases
181	A0A066SSQ2	84	3	3.57	Phenotypic	Homology	Enzyme	Protease	Infectious Diseases
182	Q13469	85	8	9.41	Functional	X-ray	Transcription Factor	-	Immunology
183	P9WNS3	94	5	5.32	Binding	X-ray	Enzyme	Transferase	Infectious Diseases
184	Q2FD70	94	3	3.19	Phenotypic	Homology	Transporter	-	Infectious Diseases
185	P31224	94	1	1.06	Phenotypic	X-ray	Transporter	-	Infectious Diseases

186	Q06330	67	7	10.4	Functional	Homology	Transcription Factor	-	Oncology
187	P61157	90	10	11.1	Other	X-ray	Other	-	Infectious Diseases
188	Q3HNM4	94	0	0.0	Functional	X-ray	Enzyme	Kinase	Infectious Diseases
189	Q15907	94	3	3.19	Functional	X-ray	Enzyme	Hydrolase	Other
190	P9WNZ1	94	0	0.0	Functional	Homology	Enzyme	Transferase	Infectious Diseases
191	P9WJY9	94	12	12.8	Functional	X-ray	Enzyme	Transferase	Infectious Diseases
192	Q9GPZ9	78	0	0.0	Functional	X-ray	Enzyme	Other Enzymes	Infectious Diseases
193	O15392	93	7	7.53	Phenotypic	X-ray	Other	-	Oncology
194	Q96D96	94	23	24.5	Binding	NMR	Transporter	-	Oncology
195	P35340	80	5	6.25	Phenotypic	X-ray	Enzyme	Other Enzymes	Infectious Diseases
196	P52732	96	2	2.08	Functional	X-ray	Other	-	Oncology
197	P52333	170	2	1.18	Binding	Homology	Enzyme	Kinase	Immunology
198	P47895	82	4	4.88	Functional	X-ray	Enzyme	Other Enzymes	Oncology
199	P08631	87	3	3.45	Phenotypic	X-ray	Enzyme	Kinase	Oncology
200	O43791	80	0	0.0	Binding	X-ray	Enzyme	Other Enzymes	Oncology

201	Q15796	77	4	5.19	Binding	X-ray	DNA/RNA-binding protein	-	Oncology
202	P21796	94	18	19.1	Binding	X-ray	Transporter	-	Oncology
203	Q9LAU2	96	11	11.5	Functional	Homology	Enzyme	Phosphatase	Infectious Diseases
204	Q9H6Y7	86	0	0.0	Functional	Homology	Enzyme	Other Enzymes	Other
205	K9WT99	96	2	2.08	Functional	X-ray	Enzyme	Other Enzymes	Oncology
206	P50454	96	0	0.0	Binding	X-ray	Transporter	-	Other
207	P0AGC3	83	0	0.0	Functional	X-ray	Enzyme	Transferase	Infectious Diseases
208	Q16822	84	5	5.95	Functional	Homology	Enzyme	Other Enzymes	Oncology
209	O35295	74	1	1.35	Binding	Homology	DNA/RNA-binding protein	-	Oncology
210	Q32ZE1	85	2	2.35	Functional	X-ray	Enzyme	Hydrolase	Infectious Diseases
211	B0FLN1	86	6	6.98	Phenotypic	Homology	Enzyme	Other Enzymes	Infectious Diseases
212	P61073	90	8	8.89	Functional	X-ray	GPCR	-	Oncology
213	P07949, Q62997	88	2	2.27	Functional	Cryo-EM	Enzyme	Kinase	Neurology
214	Q16186	94	1	1.06	Binding	X-ray	Enzyme	Protease	Oncology

215	P11498	94	4	4.26	Functional	X-ray	Enzyme	Other Enzymes	Endocrinology & Metabolism
216	P9WMH1	88	8	9.09	Phenotypic	X-ray	Transcription Factor	-	Infectious Diseases
217	Q8NEB9	78	5	6.41	Phenotypic	X-ray	Enzyme	Kinase	Oncology
218	P11474	73	4	5.48	Functional	X-ray	Other	-	Oncology
219	P03518	97	3	3.09	Functional	X-ray	Other	-	Infectious Diseases
220	P49821	87	0	0.0	Functional	Cryo-EM	Enzyme	Other Enzymes	Oncology
221	P08134	85	1	1.18	Phenotypic	X-ray	Other	-	Oncology
222	O15294	94	2	2.13	Binding	X-ray	Enzyme	Transferase	Oncology
223	O15554	83	3	3.61	Functional	Cryo-EM	Ion Channel	-	Immunology
224	Q02127	94	11	11.7	Functional	X-ray	Enzyme	Other Enzymes	Oncology
225	Q5QD14	94	2	2.13	Functional	Homology	GPCR	-	Neurology
226	Q76R61	94	3	3.19	Phenotypic	X-ray	DNA/RNA-binding protein	-	Other
227	P0C8L0	94	1	1.06	Functional	Homology	Other	-	Infectious Diseases
228	P07237	94	5	5.32	Functional	X-ray	Enzyme	Other Enzymes	Cardio-vascular diseases
229	Q15311	94	0	0.0	Phenotypic	NMR	Transporter	-	Oncology
230	Q5VWK5	94	0	0.0	Functional	X-ray	Other	-	Immunology

231	Q9UH17	85	0	0.0	Binding	X-ray	Enzyme	Hydrolase	Oncology
232	Q8JUX6	104	0	0.0	Functional	X-ray	Enzyme	Protease	Infectious Diseases
233	Q5UB51	68	3	4.41	Binding	X-ray	Enzyme	Protease	Infectious Diseases
234	Q5CVN0	83	0	0.0	Functional	Homology	Enzyme	Other Enzymes	Infectious Diseases
235	P13569	94	9	9.57	Functional	Cryo-EM	Ion Channel	-	Rare diseases & Disorders
236	Q8NBP7	94	5	5.32	Functional	X-ray	Enzyme	Hydrolase	Cardio-vascula r diseases
237	P09960	76	9	11.8	Functional	X-ray	Enzyme	Hydrolase	Immunology
238	Q99720	81	19	23.5	Binding	X-ray	Other	-	Neurology
239	P13945	84	16	19.1	Other	Homology	GPCR	-	Endocrinology & Metabolism
240	P12883	84	1	1.19	Functional	X-ray	Enzyme	Hydrolase	Cardio-vascula r diseases
241	Q6P988	87	0	0.0	Functional	X-ray	Enzyme	Hydrolase	Neurology
242	Q16526	177	6	3.39	Functional	Homology	Transcription Factor	-	Other
243	O00522, Q9ULI3	94	2	2.13	Binding	X-ray	Other	-	Cardio-vascula r diseases
244	Q96PN6	85	0	0.0	Functional	X-ray	Enzyme	Other Enzymes	Other

245	O55143	74	4	5.41	Binding	Homology	Enzyme	Hydrolase	Other
246	J9QQY6	94	0	0.0	Functional	Homology	Other	-	Infectious Diseases
247	P13501	83	1	1.2	Binding	X-ray	Other	Other Enzymes	Cardio-vascula r diseases
248	O00429	81	6	7.41	Binding	X-ray	Enzyme	Hydrolase	Neurology
249	Q99497	96	1	1.04	Functional	X-ray	Other	-	Oncology
250	Q15758	94	12	12.8	Functional	Cryo-EM	Transporter	-	Oncology
251	Q16548	94	35	37.2	Functional	X-ray	Other	Other Enzymes	Immunology
252	Q92793	89	10	11.2	Functional	X-ray	Enzyme	Transferase	Oncology
253	Q09427	96	3	3.12	Functional	Cryo-EM	Ion Channel	-	Endocrinology & Metabolism
254	O95786	94	2	2.13	Functional	X-ray	Enzyme	Hydrolase	Immunology
255	P16220	94	5	5.32	Other	X-ray	Transcription Factor	-	Oncology
256	A1Z1Q3	82	2	2.44	Binding	X-ray	Enzyme	Hydrolase	Oncology
257	Q814Y5	83	5	6.02	Functional	X-ray	Other	-	Infectious Diseases
258	O14924, O43566	96	2	2.08	Functional	NMR	GPCR	-	Neurology
259	A0QTK6	84	2	2.38	Functional	Cryo-EM	Other	-	Infectious Diseases
260	C6TCZ5	85	5	5.88	Functional	X-ray	Other	Other Enzymes	Other

261	Q9Y251	81	5	6.17	Functional	X-ray	Enzyme	Hydrolase	Oncology
262	Q9H255	94	10	10.6	Functional	Homology	GPCR	-	Oncology
263	P06400	94	15	16.0	Binding	X-ray	Other	-	Neurology
264	Q04206	84	7	8.33	Functional	X-ray	Transcription Factor	-	Oncology
265	P14778	82	5	6.1	Binding	X-ray	Other	-	Immunology
266	Q9Y2I7	73	0	0.0	Phenotypic	Homology	Enzyme	Kinase	Infectious Diseases
267	P0DTC2	94	1	1.06	Functional	Cryo-EM	Other	-	Infectious Diseases
268	Q13255	82	5	6.1	Functional	Homology	GPCR	-	Neurology
269	Q99986	81	3	3.7	Functional	X-ray	Enzyme	Kinase	Oncology
270	P16885	85	1	1.18	Binding	Homology	Enzyme	Hydrolase	Neurology
271	Q8IY81	85	3	3.53	Phenotypic	Homology	Enzyme	Transferase	Oncology
272	Q93096	84	0	0.0	Functional	X-ray	Enzyme	Phosphatase	Cardio-vascular diseases
273	Q8WTS1	70	6	8.57	Binding	Homology	Enzyme	Transferase	Other
274	Q14498	80	1	1.25	Phenotypic	X-ray	DNA/RNA-binding protein	Other Enzymes	Oncology
275	P0DTD1	94	0	0.0	Functional	X-ray	Enzyme	Protease	Infectious Diseases
276	Q8N9F0	63	5	7.94	Functional	X-ray	Enzyme	Transferase	Rare diseases & Disorders

277	P47870	84	0	0.0	Functional	Cryo-EM	Ion Channel	-	Other
278	P0A1S2	93	5	5.38	Binding	X-ray	DNA/RNA-binding protein	-	Infectious Diseases
279	P9WG83	73	10	13.7	Functional	Homology	Enzyme	Other Enzymes	Infectious Diseases
280	A0A0H3JDV8	81	1	1.23	Functional	X-ray	Enzyme	Transferase	Infectious Diseases
281	Q9NZC9	81	0	0.0	Functional	Homology	DNA/RNA-binding protein	-	Oncology
282	Q9Y5X0	84	0	0.0	Binding	X-ray	Enzyme	Transferase	Other
283	P14222	72	4	5.56	Functional	Homology	Other	-	Infectious Diseases
284	Q9Y265, Q9Y230	94	0	0.0	Functional	Cryo-EM	Enzyme	Hydrolase	Oncology
285	Q92835	83	13	15.7	Functional	X-ray	Enzyme	Phosphatase	Neurology
286	Q9H008	68	5	7.35	Binding	X-ray	Enzyme	Phosphatase	Oncology
287	Q13501	94	17	18.1	Functional	X-ray	Other	-	Cardio-vascular diseases
288	P37231	87	6	6.9	Phenotypic	X-ray	Other	-	Endocrinology & Metabolism
289	P25787, P25789	82	2	2.44	Functional	Cryo-EM	Other	-	Neurology
290	Q99638	82	0	0.0	Phenotypic	X-ray	Other	-	Oncology

291	P04062	62	7	11.3	Phenotypic	X-ray	Enzyme	Hydrolase	Rare diseases & Disorders
292	P10265	83	5	6.02	Phenotypic	Homology	Enzyme	Protease	Neurology
293	P22830	81	1	1.23	Functional	X-ray	Enzyme	Other Enzymes	Other
294	P18887	94	1	1.06	Phenotypic	X-ray	Other	-	Oncology
295	O95833	94	3	3.19	Functional	X-ray	Ion Channel	-	Oncology
296	Q8NBP7	94	1	1.06	Functional	X-ray	Enzyme	Protease	Cardio-vascula r diseases

Supplementary Table 3. Hit identification results for 296 AIMS projects.

Project Number	Uniprot ID	Structure	Mass	Purity (%)	MW	HBD	HBA	Rot	cLogP	MR	TPSA	QED
001	Q9NZQ7	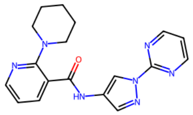	349.39	92.3	349	1	7	4	2.3	97	89	0.78
002	Q9UBT2, Q9UBE0	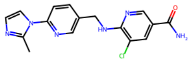	342.78	100	343	2	6	5	2.34	91	99	0.74
003	Q9HBX9	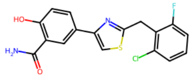	362.81	100	363	2	4	4	4.0	92	76	0.74
004	Q96KS0	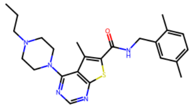	437.61	90.012	438	1	6	6	4.08	128	61	0.63

005	Q2VL90	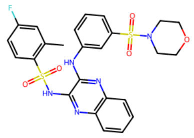	557.62	94.8	558	2	8	7	3.64	141	131	0.35
007	Q8I5R7	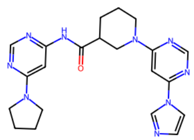	419.48	100	419	1	9	5	1.91	116	105	0.67
008	P35749	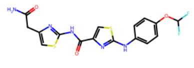	425.44	100	425	3	8	8	3.22	101	119	0.51
010	P28827	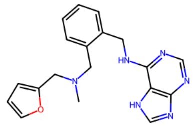	348.40	100	348	2	6	7	3.19	99	83	0.53
014	Q14508	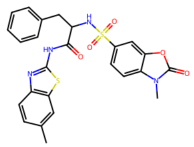	522.61	98	523	2	8	7	3.58	139	123	0.34

016	Q9UL51	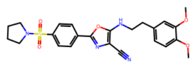	482.56	95	483	1	8	9	3.67	126	118	0.49
017	Q92743	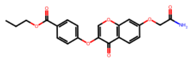	397.38	100	397	1	7	8	3.02	104	118	0.58
019	Q9NVD7	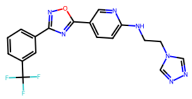	401.35	100	401	1	8	6	3.52	96	95	0.53
020	Q8NBL1	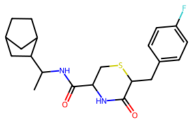	390.51	100	391	2	3	5	2.91	105	58	0.81
022	Q9Y623	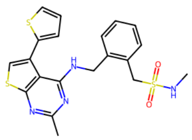	444.60	100	445	2	7	7	4.39	121	84	0.44

023	Q8TE49	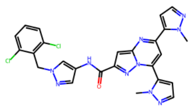	547.40	86.9	547	1	10	6	4.33	143	113	0.33
024	Q8WZ42	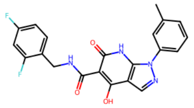	410.38	100	410	3	5	4	2.94	106	100	0.48
025	P9WJJ5	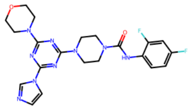	471.46	97.3	471	1	9	4	1.53	119	105	0.61
026	Q92835	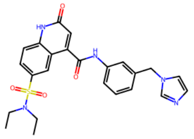	479.55	94	480	2	6	8	3.06	130	117	0.4
028	P28827	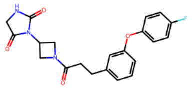	397.40	94	397	1	4	6	2.31	102	79	0.76

029	P25779	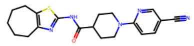	381.50	100	382	1	6	3	3.53	106	82	0.82
032	Q8IXI2	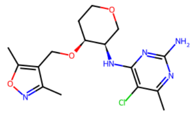	367.83	90	368	2	8	5	2.41	94	108	0.83
035	Q13936	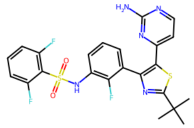	519.57	93	520	2	7	5	5.36	129	111	0.37
036	P49902	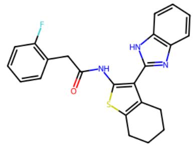	405.49	99	405	2	3	4	5.49	115	58	0.47
037	Q4WUS9	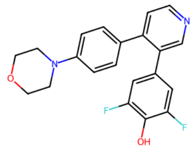	368.38	100	368	1	4	3	4.24	100	46	0.75

039	Q9BRQ0	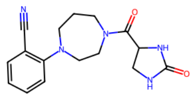	313.35	93	313	2	4	2	0.28	85	88	0.82
040	Q7PRZ8	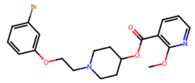	435.31	97	435	0	6	7	3.55	105	61	0.62
041	Q13009	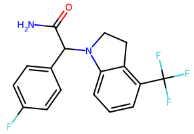	338.30	93	338	1	2	3	3.43	81	46	0.87
043	P09237	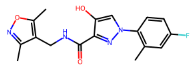	344.34	100	344	2	6	4	2.56	87	93	0.76
044	P28223	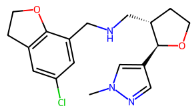	347.84	90	348	1	5	5	2.88	92	48	0.9

045	Q04IN8	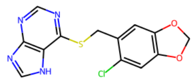	320.76	99	321	1	6	3	3.03	79	73	0.59
046	P18031	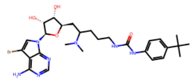	618.57	96	619	5	9	9	3.61	159	151	0.23
049	P50148	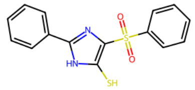	316.40	94	316	2	4	3	3.2	83	63	0.73
050	O00308	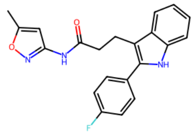	363.38	93	363	2	3	5	4.84	102	71	0.53
051	P28827	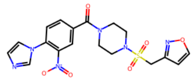	446.44	90	446	0	9	6	1.06	107	145	0.4

**052**	**P12004**	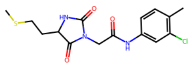	**355.84**	**97**	**356**	**2**	**4**	**6**	**2.26**	**92**	**79**	**0.77**
**053**	**P55072**	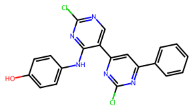	**410.27**	**90**	**410**	**2**	**6**	**4**	**5.36**	**110**	**84**	**0.35**
**055**	**Q05086**	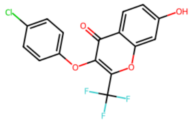	**356.68**	**99**	**357**	**1**	**4**	**2**	**4.96**	**81**	**60**	**0.7**
**056**	**Q66479**	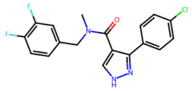	**361.77**	**97**	**362**	**1**	**2**	**4**	**4.28**	**91**	**49**	**0.75**
**057**	**O96693**	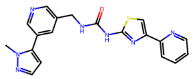	**391.45**	**95**	**391**	**2**	**7**	**5**	**3.32**	**108**	**98**	**0.54**

059	Q16740	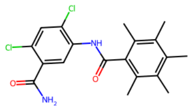	379.29	96	379	2	2	3	4.89	103	72	0.8
060	O15178	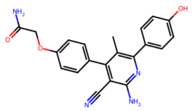	374.40	92	374	3	6	5	2.75	105	135	0.63
061	Q9JJX6	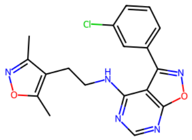	369.81	100	370	1	7	5	4.2	98	90	0.56
062	P31350	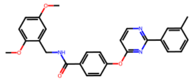	455.52	100	456	1	6	8	5.19	129	83	0.39
063	O95631	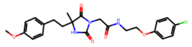	459.92	92	460	2	5	10	2.79	120	97	0.42

066	Q13586	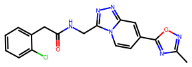	382.80	95	383	1	7	5	2.6	98	98	0.57
067	Q969H0	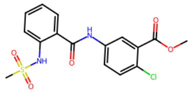	382.82	97	383	2	5	5	2.75	96	102	0.77
068	Q8IEW2	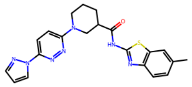	419.50	99	420	1	8	4	3.44	117	89	0.55
072	Q763K9	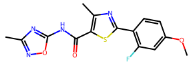	348.35	100	348	1	7	4	3.21	86	90	0.78
073	Q9NQ25	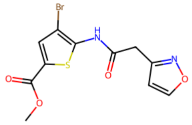	345.17	95	345	1	6	4	2.47	72	81	0.86

075	O75385	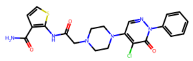	472.95	95	473	2	8	6	1.81	125	114	0.57
077	P22314	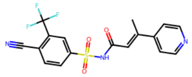	395.36	100	395	1	5	4	2.88	89	100	0.8
078	O75365	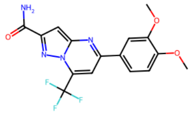	366.29	92	366	1	6	4	2.53	85	92	0.77
079	Q9Y345	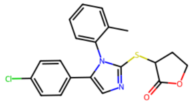	384.88	100	385	0	5	4	4.91	104	44	0.6
080	Q07812	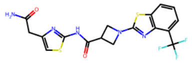	441.46	97	441	2	7	5	2.87	104	101	0.63

081	P62873	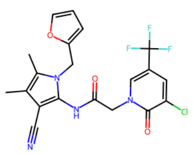	452.81	97	453	1	6	5	4.09	106	93	0.63
085	Q9Y572	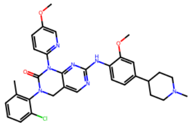	600.11	99	600	1	8	7	6.68	168	96	0.25
086	P9WGZ1	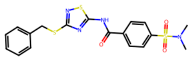	434.56	100	435	1	7	7	3.33	111	92	0.57
090	Q9UKK9	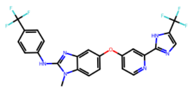	518.41	100	518	2	6	5	6.93	122	81	0.25
091	Q12923	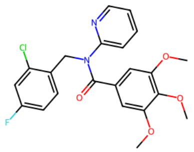	430.85	98	431	0	5	7	4.75	112	61	0.54

092	P30613	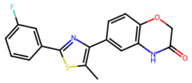	340.37	97	340	1	4	2	4.26	92	51	0.76
094	P9WKI7	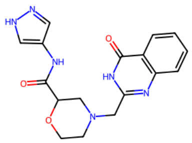	354.36	99	354	3	6	4	0.49	95	116	0.63
095	Q91MB8	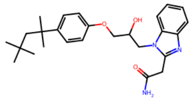	437.57	98	438	2	5	9	4.22	128	90	0.52
096	Q9Y251	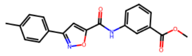	336.34	99	336	1	5	4	3.69	92	81	0.73
097	P14340	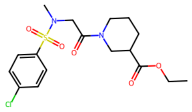	402.89	90	403	0	5	6	1.76	97	84	0.68

098	O60603, Q15399	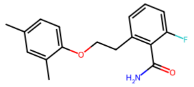	287.33	98	287	1	2	5	3.16	80	52	0.92
099	P9WPC3	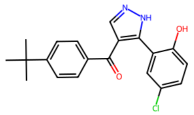	354.84	100	355	2	3	3	4.96	99	66	0.65
100	O15294	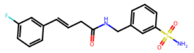	348.39	95	348	2	3	6	2.19	90	89	0.84
103	B1LMQ5	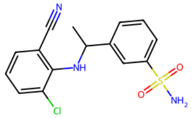	335.81	92	336	2	4	4	3.03	86	96	0.9
104	P15056	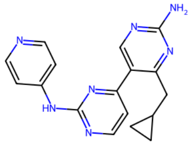	319.36	100	319	2	7	5	2.61	91	102	0.74

106	P26358	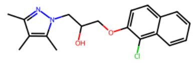	344.83	100	345	1	4	5	4.05	97	47	0.76
108	Q99ZW2	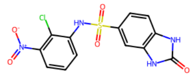	368.75	90	369	3	5	4	2.22	88	138	0.48
109	Q9NRL2	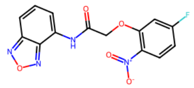	332.25	96	332	1	7	5	2.29	79	120	0.56
110	P25779	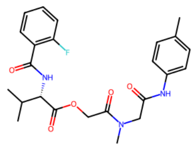	457.49	90	458	2	5	9	2.53	121	105	0.56
111	Q02880	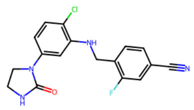	344.77	100	345	2	3	4	3.49	91	68	0.89

112	Q9H1Y0	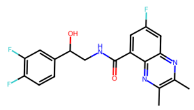	375.34	98	375	2	4	4	3.13	93	75	0.73
115	Q8FCR6	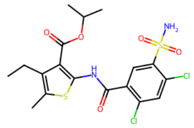	479.40	94	479	2	6	6	4.39	115	116	0.6
119	Q96HS1	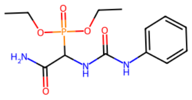	329.29	100	329	3	5	8	1.89	82	120	0.63
120	P05121	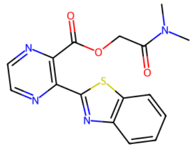	342.37	97	342	0	7	4	2.0	90	85	0.67
121	Q9BUX1	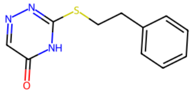	233.29	99	233	1	4	4	1.5	64	59	0.81

123	O14929	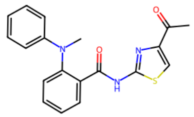	351.42	93	351	1	5	5	4.37	101	62	0.69
124	P32322	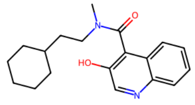	312.40	100	312	1	3	4	3.98	92	53	0.93
126	Q96LB1	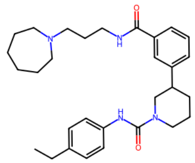	490.68	99	491	2	3	8	5.66	147	65	0.47
127	P9WQ17	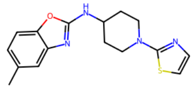	314.40	100	314	1	6	3	3.67	90	54	0.8
130	Q5CYE4	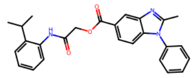	427.50	100	428	1	5	6	5.25	125	73	0.43

**134**	**P09874**	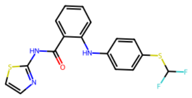	**377.43**	**100**	**377**	**2**	**5**	**6**	**5.45**	**98**	**54**	**0.56**
**135**	**P55072**	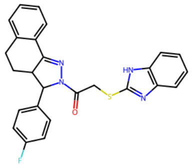	**456.54**	**90**	**457**	**1**	**4**	**4**	**5.34**	**128**	**61**	**0.42**
**136**	**O60264**	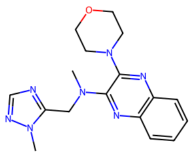	**339.39**	**94**	**339**	**0**	**8**	**4**	**1.23**	**96**	**72**	**0.71**
**141**	**P31483**	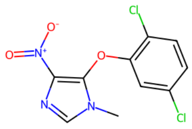	**288.08**	**100**	**288**	**0**	**5**	**3**	**3.43**	**66**	**70**	**0.64**
**142**	**P12931**	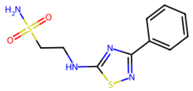	**284.36**	**90**	**284**	**2**	**6**	**5**	**0.91**	**72**	**98**	**0.85**

143	P0A031	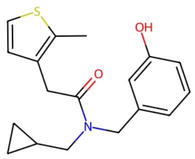	315.43	97	315	1	3	6	3.74	89	41	0.88
144	P34913	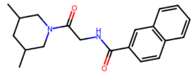	324.42	90	324	1	2	3	3.07	96	49	0.94
145	P9WG49	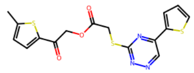	391.49	94	391	0	9	7	3.49	98	82	0.35
146	Q9NZC2	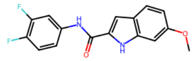	302.27	91	302	2	2	3	3.71	79	54	0.77
147	P49354, P49356	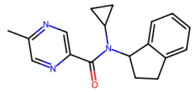	293.36	91	293	0	3	3	3.08	83	46	0.87

148	Q15717	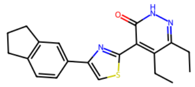	351.46	90	351	1	4	4	4.17	102	59	0.77
150	P31539	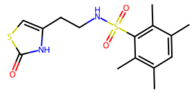	340.46	90	340	2	4	5	2.19	89	79	0.88
151	O15554	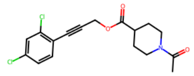	354.23	90	354	0	3	2	3.15	89	47	0.61
152	Q5W0Z9	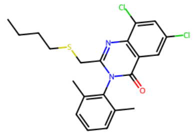	421.38	100	421	0	4	6	6.34	118	35	0.44
153	P55851	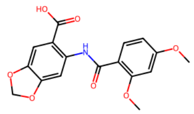	345.30	90	345	2	6	5	2.38	87	103	0.86

154	P42345	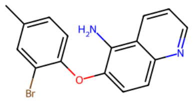	329.19	95	329	1	3	2	4.68	85	48	0.69
155	Q9YLJ3	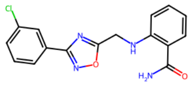	328.75	90	329	2	5	5	3.1	87	94	0.75
156	Q99836	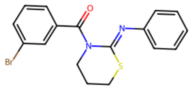	375.28	94	375	0	3	2	4.72	96	33	0.76
157	P04637	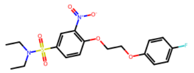	412.43	90	412	0	6	10	3.22	101	99	0.34
158	P9WNM7	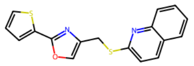	324.42	100	324	0	5	4	5.24	91	39	0.48

159	I6Y9J2	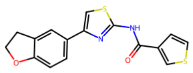	328.41	95	328	1	5	3	4.06	89	51	0.79
160	Q15118	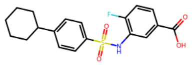	377.43	90	377	2	3	5	4.37	96	83	0.81
161	P0DTC2	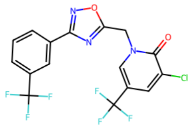	423.70	100	424	0	5	3	4.64	85	61	0.58
162	P0DTC2	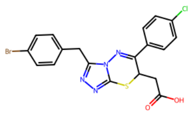	477.76	100	478	1	6	5	4.49	112	80	0.59
163	P0DTD1	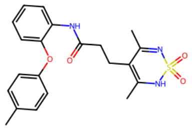	413.49	97	413	2	4	6	4.09	113	97	0.75

164	P0DTD1	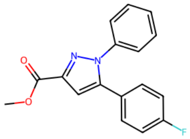	296.30	96	296	0	4	3	3.47	80	44	0.7
165	Q8ZNV8	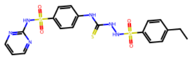	492.60	90	493	4	7	8	2.02	125	142	0.27
166	P68135	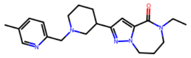	367.49	97	367	0	5	4	2.83	105	54	0.83
167	O14842	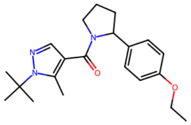	355.47	90	355	0	4	4	4.32	103	47	0.82
168	P15559	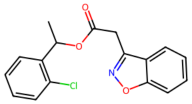	315.75	98	316	0	4	4	4.33	84	52	0.67

169	P52270	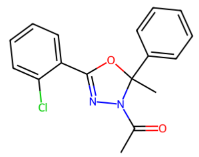	314.77	90	315	0	3	2	3.75	85	42	0.85
170	P43490	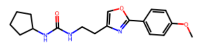	329.39	95	329	2	4	6	3.13	91	76	0.85
171	Q99538	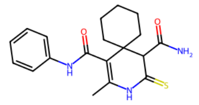	357.47	97	357	3	3	3	2.88	102	84	0.73
172	P28335	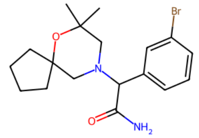	381.31	97	381	1	3	3	3.4	94	56	0.87
174	A0A6L0XSJ0	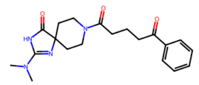	370.50	90	370	1	5	5	1.45	102	82	0.8

176	P0C0V0	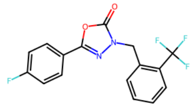	338.25	90	338	0	4	3	3.71	77	48	0.68
177	Q9H9Z2	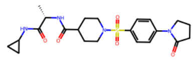	462.57	92	463	2	5	7	1.0	118	116	0.63
178	Q9R1M7	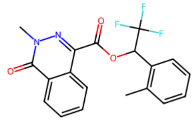	376.33	100	376	0	5	3	3.7	92	61	0.65
180	A0A1D3GEK4	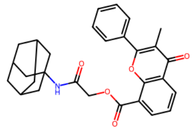	471.54	90	472	1	5	5	5.01	132	86	0.53
181	A0A066SSQ2	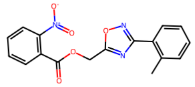	339.30	90	339	0	7	5	3.31	87	108	0.4

182	Q13469	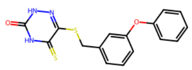	343.43	90	343	2	5	5	3.91	93	71	0.54
183	P9WNS3	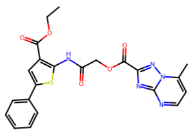	465.48	100	465	1	10	7	3.13	120	125	0.41
184	Q2FD70	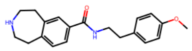	360.88	100	324	2	3	5	2.36	96	50	0.89
185	P31224	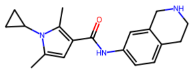	345.87	94	309	2	3	3	3.34	92	46	0.91
186	Q06330	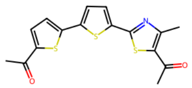	347.48	100	347	0	6	4	5.31	93	47	0.6

187	P61157	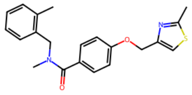	366.48	90	366	0	4	6	4.61	105	42	0.64
189	Q15907	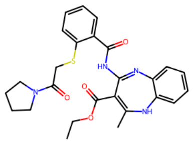	492.58	100	493	2	7	6	4.12	137	100	0.46
191	P9WJY9	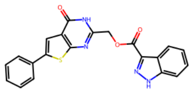	402.42	91	402	2	6	4	3.88	111	101	0.44
193	O15392	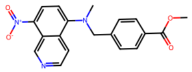	351.36	100	351	0	6	5	3.57	98	86	0.4
194	Q96D96	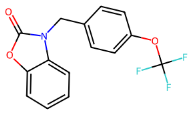	309.24	100	309	0	4	3	3.54	73	44	0.74

195	P35340	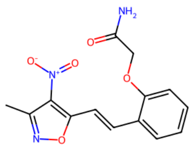	303.27	90	303	1	6	6	1.93	78	121	0.64
196	P52732	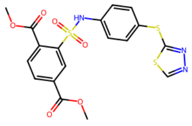	465.52	100	466	1	10	7	3.06	110	125	0.52
197	P52333	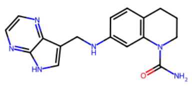	322.36	100	322	3	4	3	2.4	93	100	0.69
198	P47895	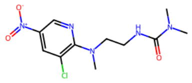	301.73	91	302	1	5	5	1.35	76	92	0.65
199	P08631	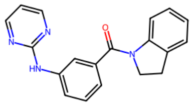	316.36	98	316	1	4	3	3.42	94	58	0.8

201	Q15796	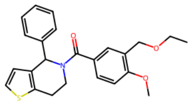	407.53	90	408	0	4	6	5.08	116	39	0.57
202	P21796	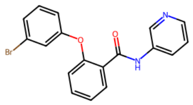	369.21	94	369	1	3	4	4.89	93	51	0.71
203	Q9LAU2	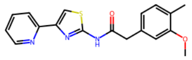	339.41	100	339	1	5	5	3.7	95	64	0.77
205	K9WT99	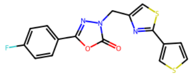	359.40	95	359	0	7	4	3.88	91	61	0.55
208	Q16822	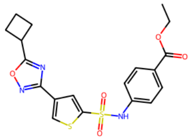	433.50	100	434	1	8	7	4.04	107	111	0.56

209	O35295	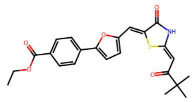	425.50	90	426	1	6	5	3.1	117	89	0.63
210	Q32ZE1	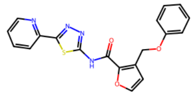	378.41	90	378	1	7	6	4.02	101	90	0.54
211	B0FLN1	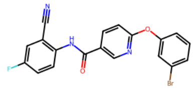	412.21	90	412	1	4	4	4.9	98	75	0.66
212	P61073	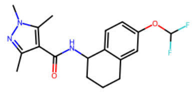	349.37	100	349	1	4	4	3.45	89	56	0.92
213	P07949, Q62997	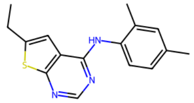	283.40	90	283	1	4	3	4.61	86	38	0.76

**214**	**Q16186**	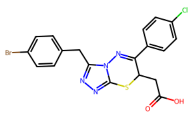	**477.76**	**100**	**478**	**1**	**6**	**5**	**4.49**	**112**	**80**	**0.59**
**215**	**P11498**	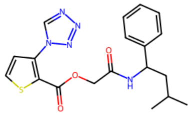	**399.47**	**91**	**399**	**1**	**8**	**8**	**2.78**	**104**	**99**	**0.58**
**216**	**P9WMH1**	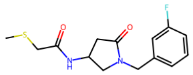	**296.36**	**100**	**296**	**1**	**3**	**5**	**1.41**	**77**	**49**	**0.89**
**217**	**Q8NEB9**	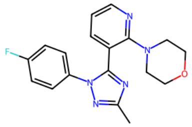	**339.37**	**100**	**339**	**0**	**6**	**3**	**2.61**	**92**	**56**	**0.73**
**218**	**P11474**	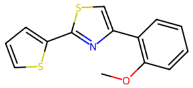	**273.37**	**99**	**273**	**0**	**4**	**3**	**4.55**	**77**	**22**	**0.7**

**219**	**P03518**	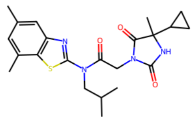	**428.55**	**99**	**429**	**1**	**5**	**6**	**3.62**	**118**	**83**	**0.71**
**221**	**P08134**	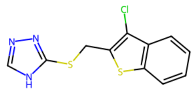	**281.78**	**90**	**282**	**1**	**4**	**3**	**3.97**	**73**	**42**	**0.74**
**222**	**O15294**	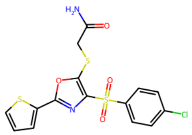	**414.91**	**100**	**415**	**1**	**7**	**6**	**3.47**	**97**	**103**	**0.62**
**223**	**O15554**	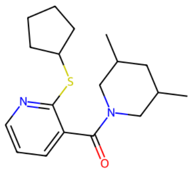	**318.48**	**90**	**318**	**0**	**3**	**3**	**4.23**	**91**	**33**	**0.84**
**224**	**Q02127**	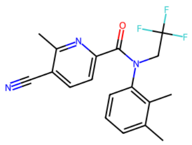	**347.33**	**99**	**347**	**0**	**3**	**3**	**4.09**	**87**	**57**	**0.84**

225	Q5QD14	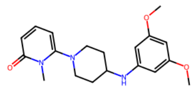	343.42	100	343	1	6	5	2.48	100	56	0.9
226	Q76R61	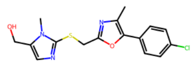	349.84	96	350	1	6	5	3.82	90	64	0.71
227	P0C8L0	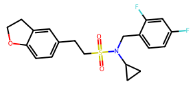	393.45	91	393	0	3	7	3.44	98	47	0.72
228	P07237	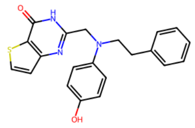	377.45	100	377	2	5	6	3.94	110	69	0.53
233	Q5UB51	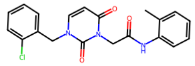	383.83	98	384	1	5	5	2.66	105	73	0.74

235	P13569	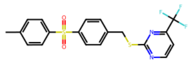	424.46	100	424	0	5	5	4.93	100	60	0.43
236	Q8NBP7	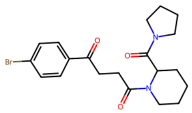	421.32	97	421	0	3	5	3.42	103	58	0.69
237	P09960	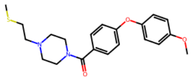	386.51	90	387	0	5	7	3.61	110	42	0.73
238	Q99720	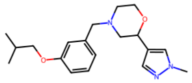	329.44	90	329	0	5	6	3.03	94	40	0.82
239	P13945	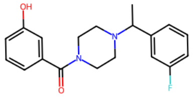	328.38	90	328	1	3	3	3.05	90	44	0.94

240	P12883	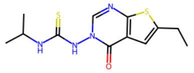	296.42	90	296	2	5	3	1.85	83	59	0.85
242	Q16526	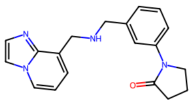	320.39	97	320	1	4	5	2.75	94	50	0.79
243	O00522, Q9ULI3	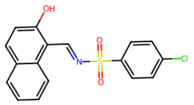	345.80	90	346	1	3	3	4.01	92	67	0.73
245	O55143	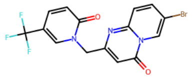	400.15	100	400	0	5	2	2.69	84	56	0.67
247	P13501	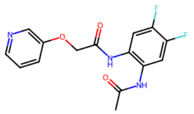	321.28	97	321	2	4	5	2.34	79	80	0.89

248	O00429	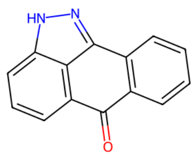	220.23	95	220	1	2	0	2.77	65	46	0.49
249	Q99497	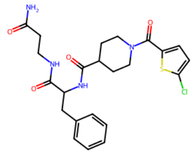	491.00	100	491	3	5	9	1.97	127	122	0.5
250	Q15758	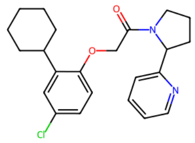	398.92	91	399	0	3	5	5.53	111	42	0.66
251	Q16548	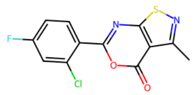	296.70	98	297	0	5	1	3.41	71	56	0.69
252	Q92793	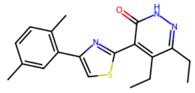	339.45	90	339	1	4	4	4.3	100	59	0.77

**253**	**Q09427**	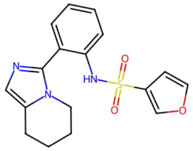	**343.40**	**90**	**343**	**1**	**5**	**4**	**3.28**	**90**	**77**	**0.79**
**254**	**O95786**	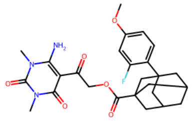	**499.53**	**92**	**500**	**1**	**9**	**6**	**2.08**	**128**	**123**	**0.48**
**255**	**P16220**	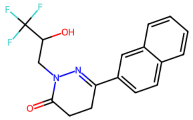	**336.31**	**96**	**336**	**1**	**3**	**3**	**3.09**	**83**	**53**	**0.94**
**256**	**A1Z1Q3**	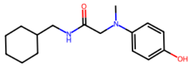	**276.37**	**97**	**276**	**2**	**3**	**5**	**2.52**	**81**	**53**	**0.87**
**257**	**Q814Y5**	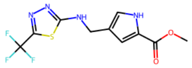	**306.27**	**90**	**306**	**2**	**6**	**4**	**2.28**	**64**	**80**	**0.85**

258	O14924, O43566	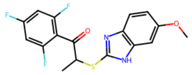	366.36	94	366	1	4	5	4.35	89	55	0.54
259	A0QTK6	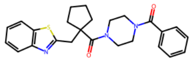	433.56	90	434	0	4	4	4.38	123	54	0.61
260	C6TCZ5	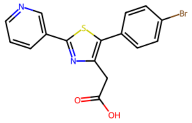	375.24	90	375	1	4	4	4.26	90	63	0.74
261	Q9Y251	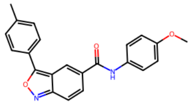	358.39	90	358	1	4	4	5.06	105	64	0.55
262	Q9H255	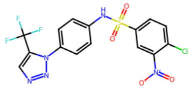	447.78	97	448	1	7	5	3.65	95	120	0.47

263	P06400	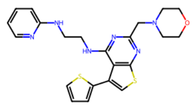	452.60	100	453	2	9	8	4.17	128	75	0.39
264	Q04206	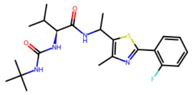	434.57	90	435	3	4	6	4.56	119	83	0.62
265	P14778	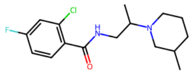	312.81	90	313	1	2	4	3.33	83	32	0.92
267	P0DTC2	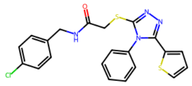	440.97	100	441	1	6	7	5.06	119	60	0.41
268	Q13255	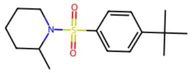	295.44	90	295	0	2	2	3.55	82	37	0.84

269	Q99986	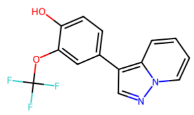	294.23	90	294	1	4	2	3.61	69	47	0.79
270	P16885	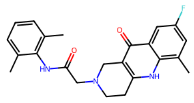	393.45	90	393	2	3	3	3.59	113	65	0.71
271	Q8IY81	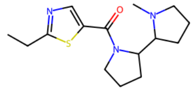	293.43	90	293	0	4	3	2.4	81	36	0.86
273	Q8WTS1	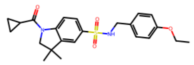	428.55	90	429	1	4	7	3.6	116	76	0.73
274	Q14498	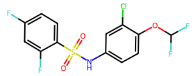	369.72	90	370	1	3	5	4.02	75	55	0.81

276	Q8N9F0	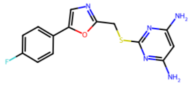	317.34	98	317	2	7	4	2.73	82	104	0.56
278	P0A1S2	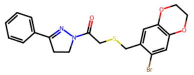	447.34	100	447	0	5	5	4.09	111	51	0.69
279	P9WG83	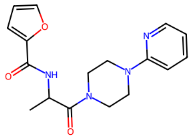	328.36	100	328	1	5	4	1.14	89	79	0.91
280	A0A0H3JDV8	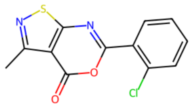	278.71	90	279	0	5	1	3.27	71	56	0.69
283	P14222	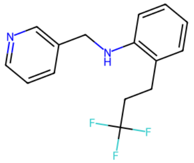	280.29	98	280	1	2	5	4.19	72	25	0.89

285	Q92835	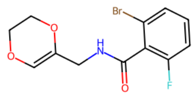	316.12	90	316	1	3	3	2.21	66	48	0.93
286	Q9H008	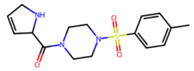	395.48	90	335	1	4	3	0.36	87	70	0.81
287	Q13501	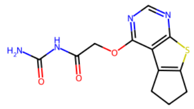	292.31	100	292	2	6	3	0.75	73	107	0.87
288	P37231	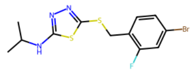	362.28	90	362	1	5	5	4.55	82	38	0.79
289	P25787, P25789	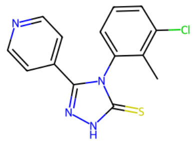	302.78	90	303	1	4	2	3.95	82	46	0.73

291	P04062	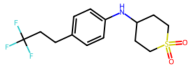	321.36	100	321	1	3	4	3.17	76	46	0.93
292	P10265	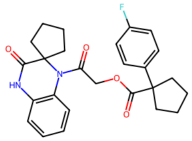	455.62	90	451	1	4	4	4.48	121	76	0.7
293	P22830	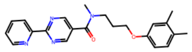	376.45	90	376	0	5	7	3.7	108	68	0.59
294	P18887	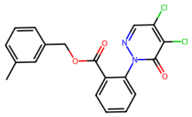	389.23	94	389	0	5	4	4.2	100	61	0.63
295	O95833	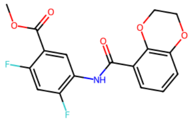	349.28	98	349	1	5	3	2.77	83	74	0.86

**296**	**Q8NBP7**	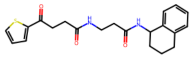	**384.49**	**96**	**385**	**2**	**4**	**8**	**3.41**	**106**	**75**	**0.69**

Supplementary Table 4. Structures of representative bioactive compounds along with their QC data and calculated physical properties from each of the 215 projects with at least one discovered bioactive compound. All compounds reported in this study are off-the-self catalog compounds. The Mass and Purity values are reported from the purchase order received from the supplier, and account for salts if applicable. Values not received from the supplier are indicated by a '-' sign. The number of hydrogen bond donors (HBD), number of hydrogen bond acceptors (HBA), number of rotational bonds (Rot), calculated logP (cLogP), molecular refractivity (MR), topological polar surface area (TPSA), and quantitative estimation of drug-likeness (QED[19]), were calculated using RDkit[20].

Project Number	UniProt ID	Research Area	# of SD hits	# of DR hits	Potency Range (μM)	Units
017	Q92743	Other	8	0	-	-
044	P28223	Infectious Diseases	7	3	0.5306 - 1.574	IC50
046	P18031	Endocrinology & Metabolism	13	3	0.2665 - 42.29	Kd
049	P50148	Immunology	1	1	55.2	IC50
050	O00308	Oncology	2	0	-	-
053	P55072	Other	15	2	1.186 - 2.176	IC50
093	Q16740	Oncology	4	0	-	-
094	P9WKI7	Infectious Diseases	7	1	22.1	IC50
103	B1LMQ5	Infectious Diseases	7	0	-	-
104	P15056	Oncology	2	2	2.297 - 118.2	IC50
120	P05121	Rare diseases & Disorders	13	8	21.33 - 865.7	IC50
135	P55072	Oncology	14	7	5.731 - 428.4	IC50
142	P12931	Oncology	5	3	0.401 - 75.85	EC50
143	P0A031	Infectious Diseases	10	2	2.21 - 31.55	IC50

144	P34913	Other	6	4	0.004 - 0.642	IC50
148	Q15717	Neurology	1	1	51.14	IC50
151	O15554	Immunology	2	1	5.766	IC50, EC50
153	P55851	Oncology	5	1	993.1	IC50
155	Q9YLJ3	Infectious Diseases	2	0	-	-
163	P0DTD1	Infectious Diseases	11	7	21.31 - 44.06	IC50
169	P52270	Infectious Diseases	20	8	12.2 - 34.54	IC50
174	A0A6L0XSJ0	Infectious Diseases	8	5	0.08519 -17.5	IC50
178	Q9R1M7	Neurology	17	8	3.077 - 18.99	IC50
182	Q13469	Immunology	8	1	0.58	IC50
183	P9WNS3	Infectious Diseases	5	1	195.6	IC50
186	Q06330	Oncology	7	3	1.657 - 22.71	IC50
189	Q15907	Other	3	0	-	-
191	P9WJY9	Infectious Diseases	12	3	25.64 - 51.42	IC50
196	P52732	Oncology	2	0	-	-
197	P52333	Immunology	2	2	36.98 - 69.76	IC50

198	P47895	Oncology	4	4	19.25 - 190.5	IC50
199	P08631	Oncology	3	3	0.2554 - 0.6474	IC50
205	K9WT99	Oncology	1	2	0.9516 - 5.01	IC50
209	O35295	Oncology	1	1	2.78	IC50
219	P03518	Infectious Diseases	3	0	-	-
228	P07237	Cardio-vascular diseases	1	1	3.59	IC50
235	P13569	Rare diseases & Disorders	9	9	0.1715 - 16.56	IC50
237	P09960	Immunology	9	9	1.229 - 33.57	IC50
240	P12883	Cardio-vascular diseases	1	1	20.87	IC50
242	Q16526	Other	6	2	122.2	IC50
243	O00522,Q9ULI3	Cardio-vascular diseases	2	1	862.5	IC50
248	O00429	Neurology	6	1	261.6	Kd
251	Q16548	Immunology	35	2	117.2 - 217.4	IC50
255	P16220	Oncology	5	2	1.014 - 14.26	IC50
258	O14924,O43566	Neurology	3	1	64.17	IC50
261	Q9Y251	Oncology	5	4	44.96 - 220.9	IC50

262	Q9H255	Oncology	10	7	0.01797 - 81.72	IC50
264	Q04206	Oncology	7	3	0.8557 - 2.982	IC50
273	Q8WTS1	Other	6	6	6.4965 - 46.985	IC50

Supplementary Table 5. Dose-response results for 49 AIMS projects in the AIMS-validation follow up studies. In the AIMS-validation dose-response (DR) studies we attempted to reproduce the primary single-dose (SD) hits and follow up with DR measurements, using the same assay at the same academic lab.

Project Number	UniProt ID	Structure	IC_{50}	Hill Slope	R^2	Dose-response curve
044	P28223	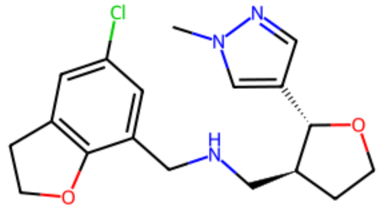	1.574	1.971	0.9965	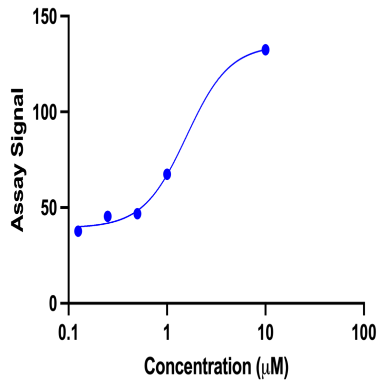
046	P18031	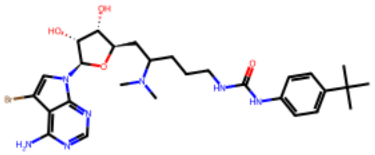	42.29*	-	0.7915	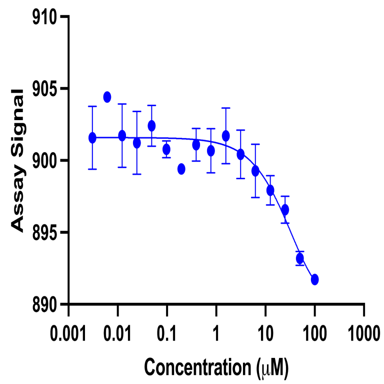

049	P50148	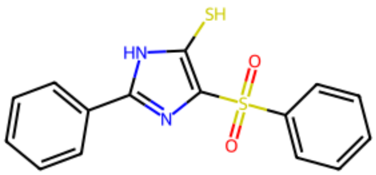	55.2	-1.77	0.9945	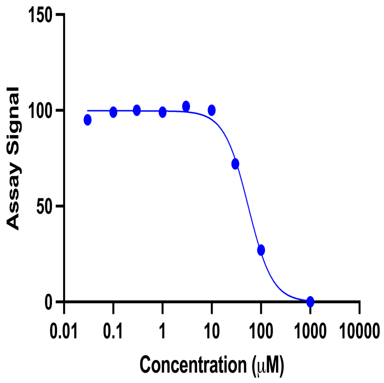
053	P55072	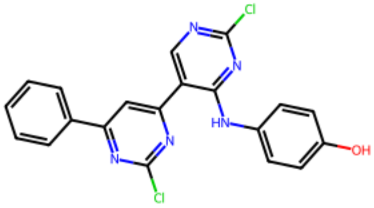	1.186	-1.563	0.8988	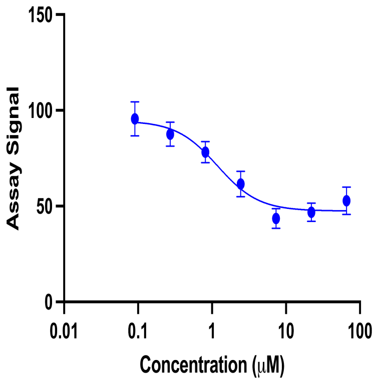

094	P9WKI7	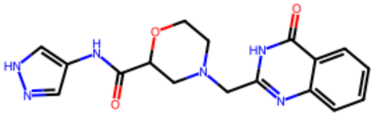	22.1	-0.956	0.9994	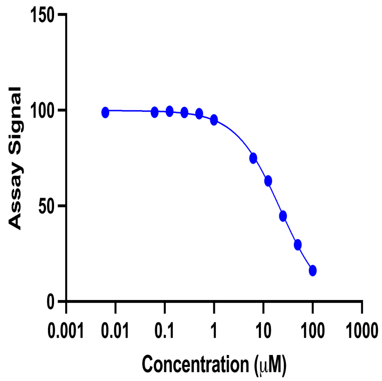
104	P15056	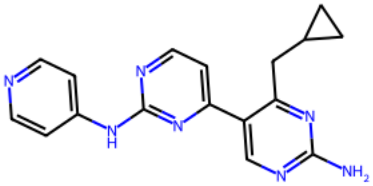	2.297	0.8474	0.9979	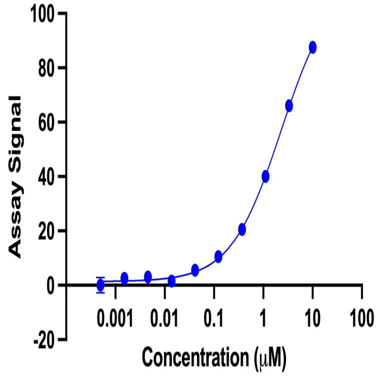

120	P05121	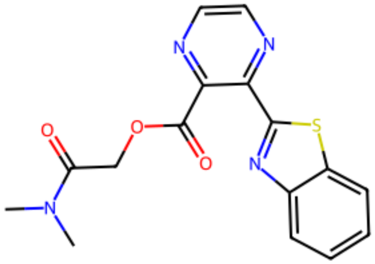	198.5	-1.082	0.9325	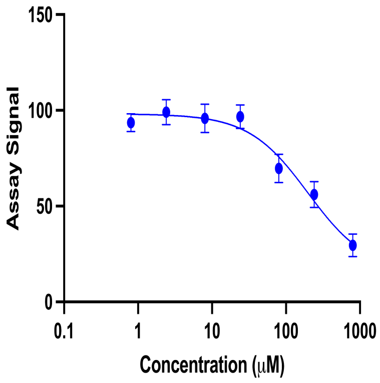
135	P55072	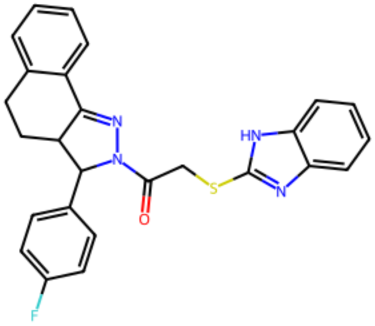	67.17	-1.065	0.9921	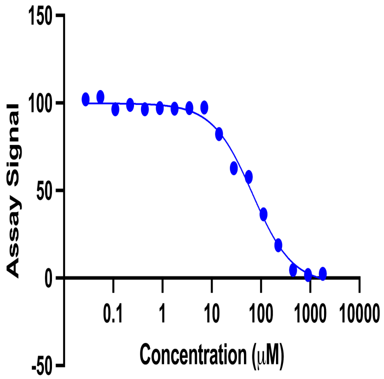

142	P12931	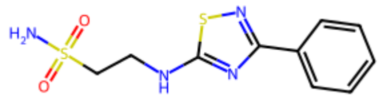	0.401	-1.042	0.9897	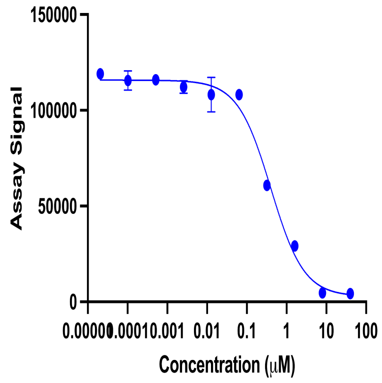
143	P0A031	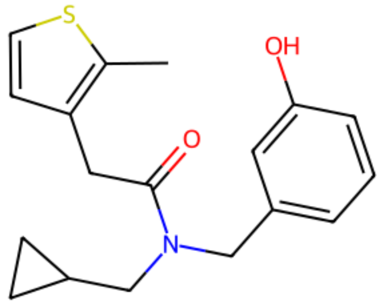	2.21	-3.475	0.9862	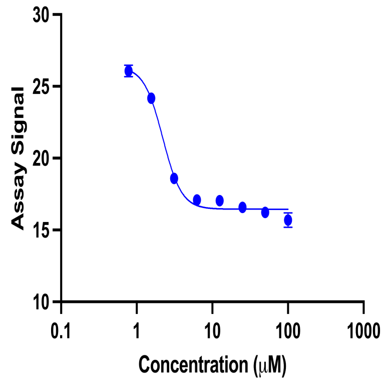

144	P34913	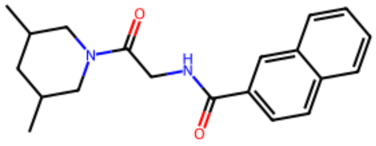	0.037	0.9207	0.9809	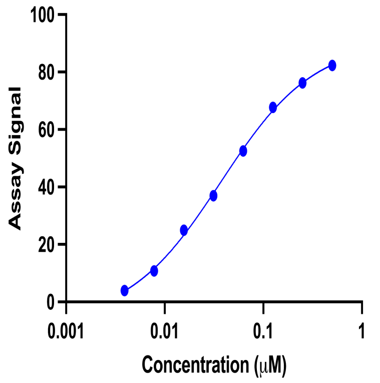
148	Q15717	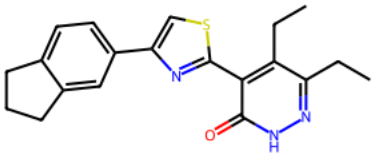	51.14	-2.467	0.9914	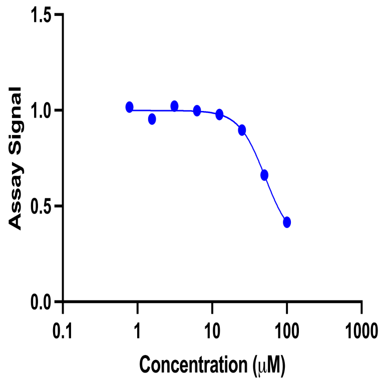

151	O15554	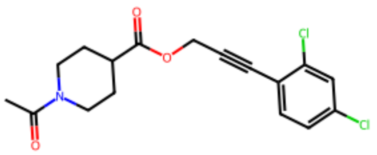	5.766	2.509	0.9775	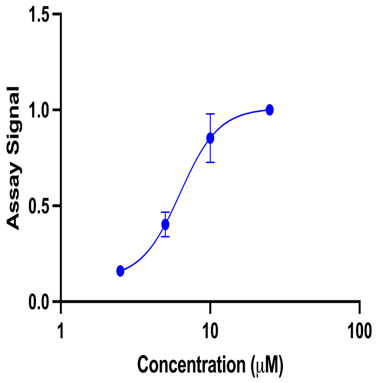
153	P55851	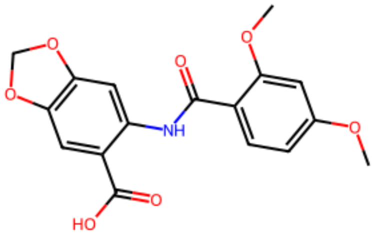	993.1	-0.9156	0.9803	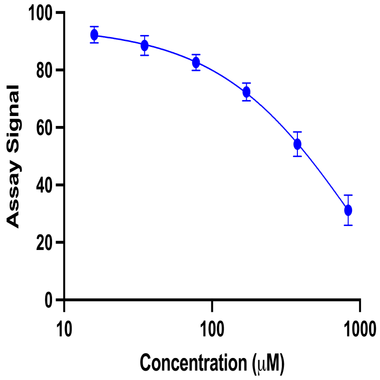

163	P0DTD1	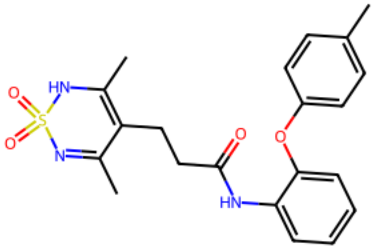	30.11	-2.854	0.9938	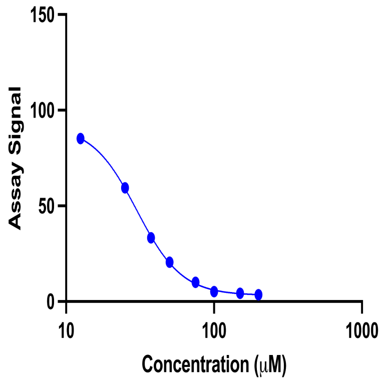
169	P52270	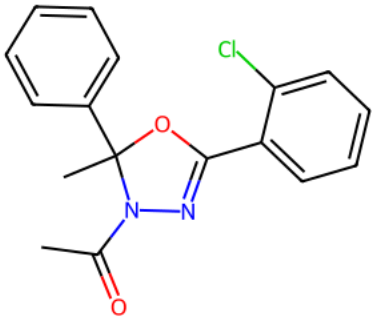	16.43	3.979	0.9899	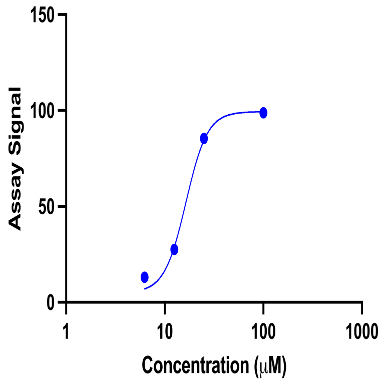

174	A0A6L0XSJ0	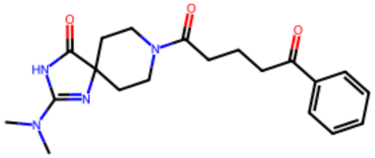	1.583	-1.331	0.896	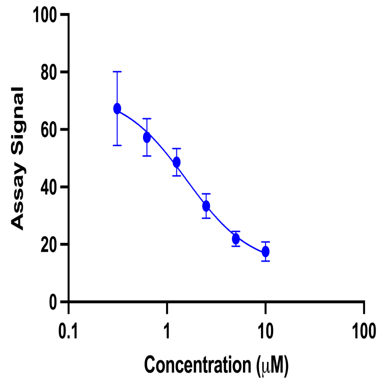
178	Q9R1M7	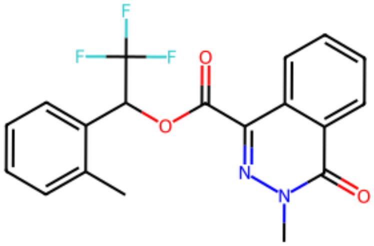	6.215	1.984	0.9989	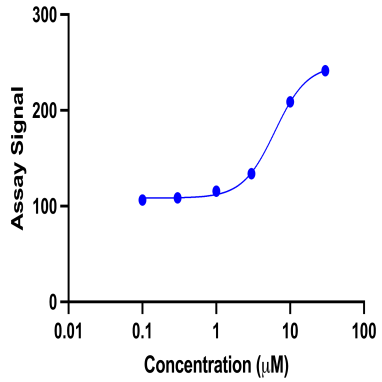

182	Q13469	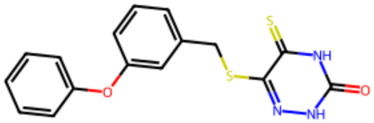	0.58	-1.313	0.9711	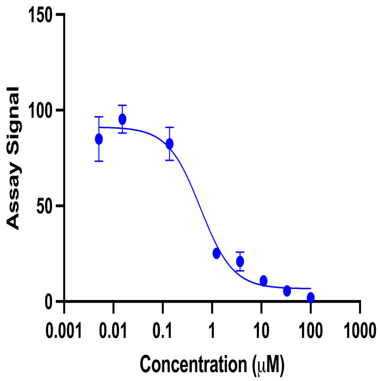
183	P9WNS3	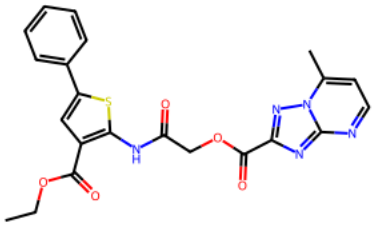	195.6	1.848	0.9564	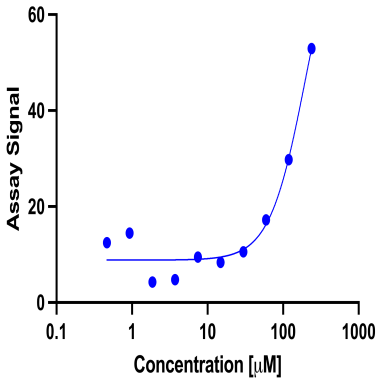

186	Q06330	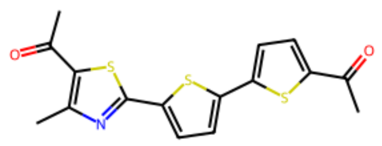	1.657	2.806	1.0	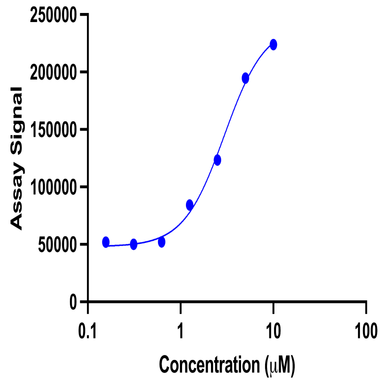
191	P9WJY9	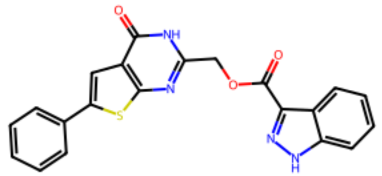	25.64	-2.987	0.9906	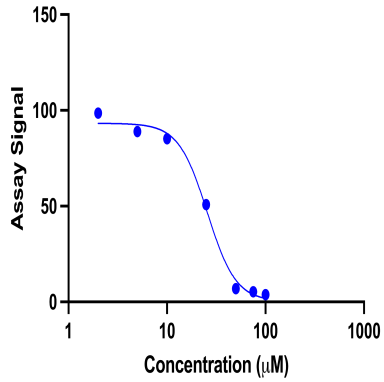

197	P52333	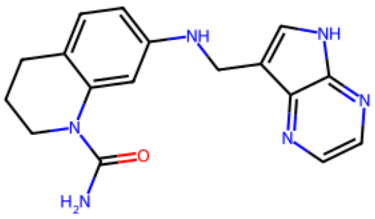	36.98	-1.934	0.8727	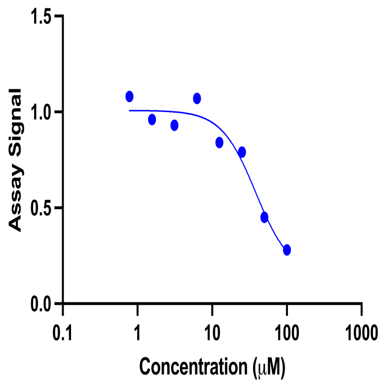
198	P47895	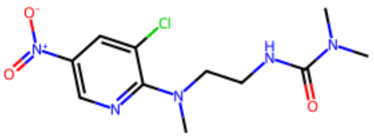	19.25	-0.8342	0.976	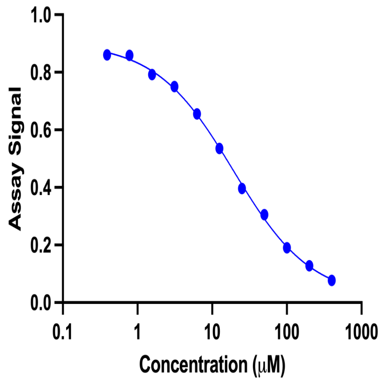

199	P08631	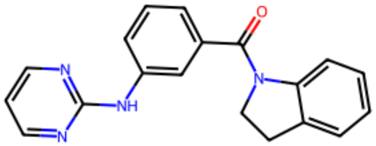	0.25	-1.061	0.8506	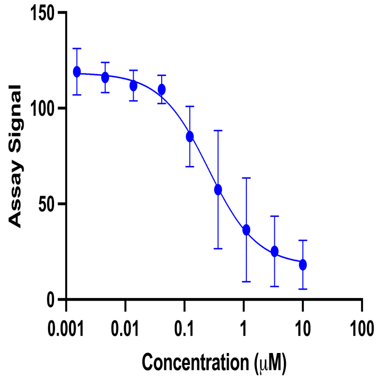
205	K9WT99	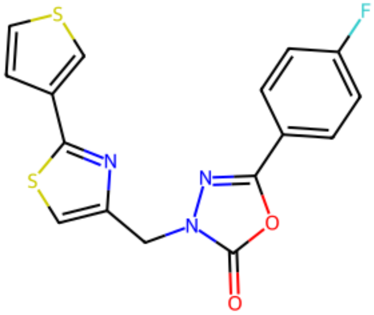	5.01	1.047	0.9885	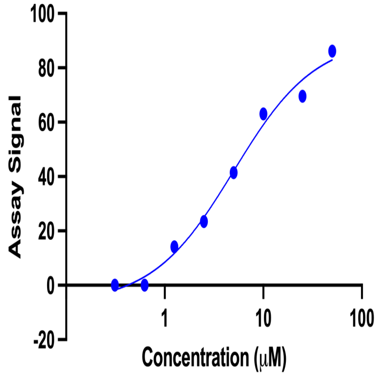

209	O35295	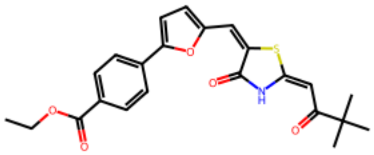	2.78	-1.331	0.9762	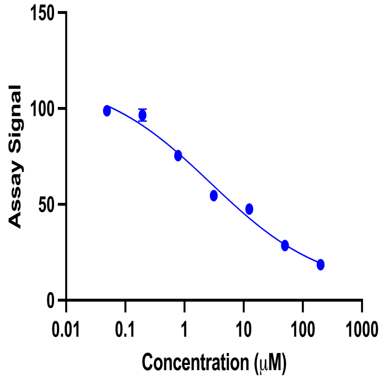
228	P07237	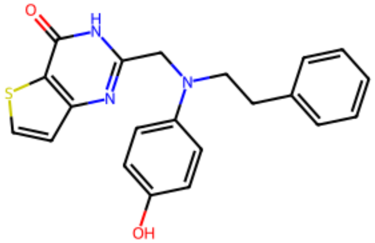	3.59	0.5685	0.9486	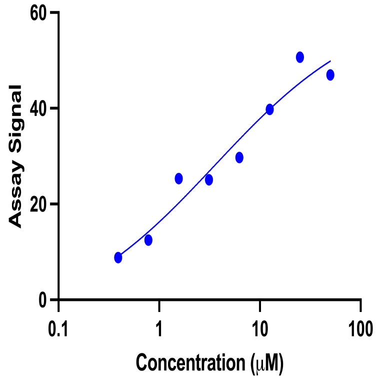

235	P13569	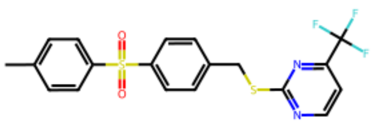	0.171	1.333	0.9728	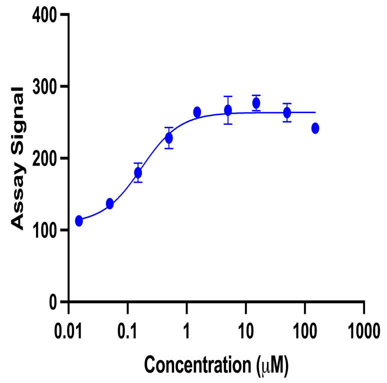
237	P09960	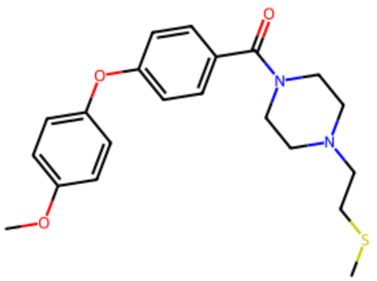	6.789	-0.8651	0.9864	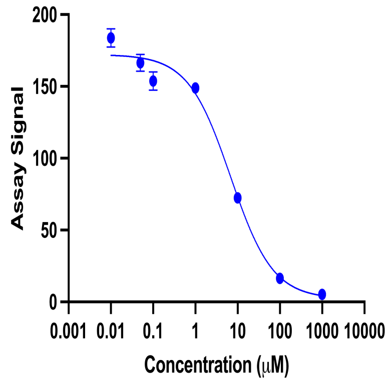

240	P12883	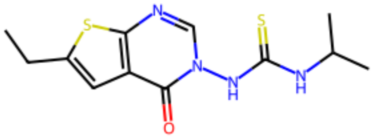	20.87	-1.015	0.9149	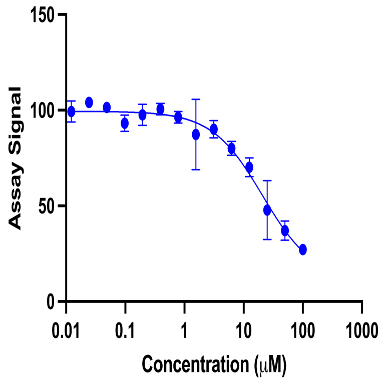
242	Q16526	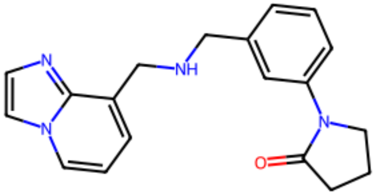	122.2	-1.193	0.9964	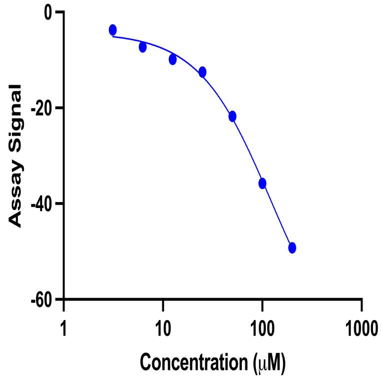

243	O00522,Q9UL I3	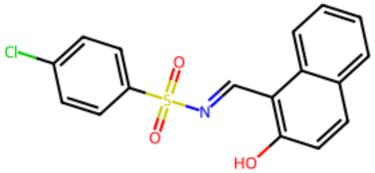	862.5	-0.7202	0.9399	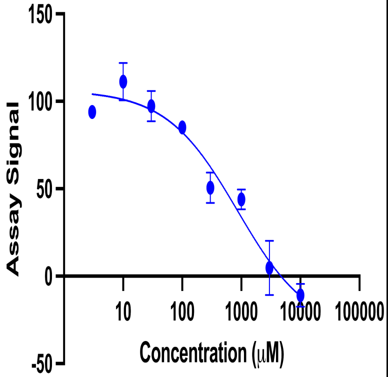
248	O00429	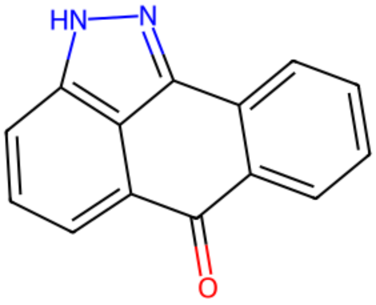	261.6*	-	0.9048	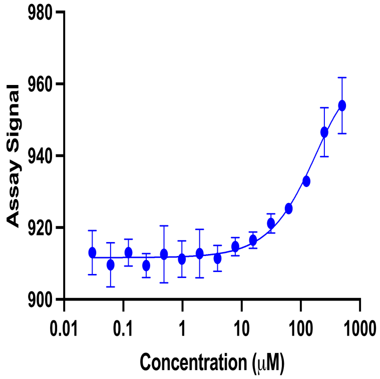

251	Q16548	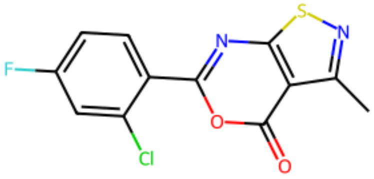	117.2	-2.874	0.9984	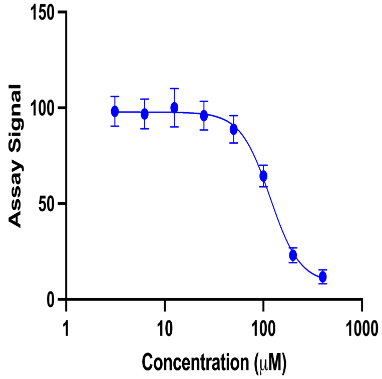
255	P16220	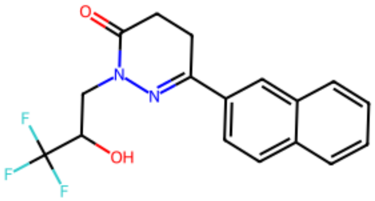	14.26	1.335	0.9542	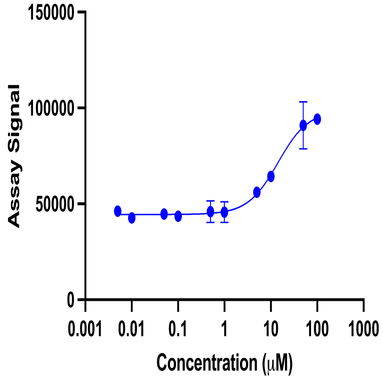

258	O14924,O435 66	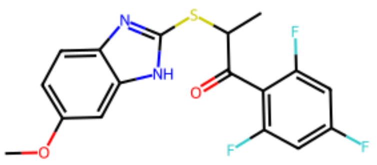	64.17	-0.9562	0.9863	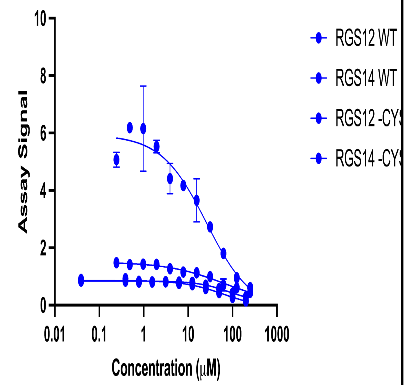
261	Q9Y251	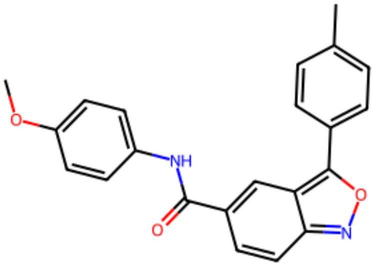	71.29	2.296	0.979	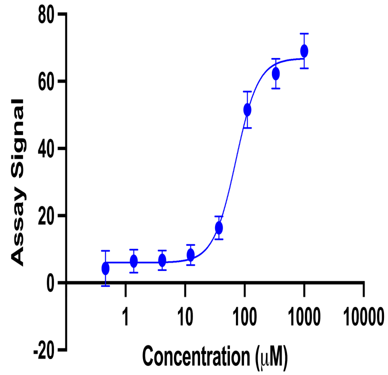

262	Q9H255	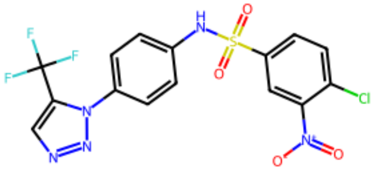	12.99	-1.719	0.8538	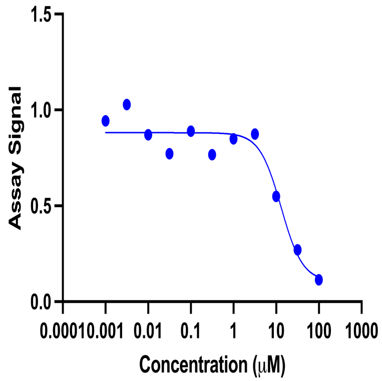
264	Q04206	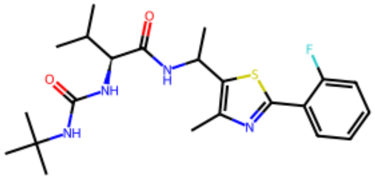	2.982	1.307	0.9714	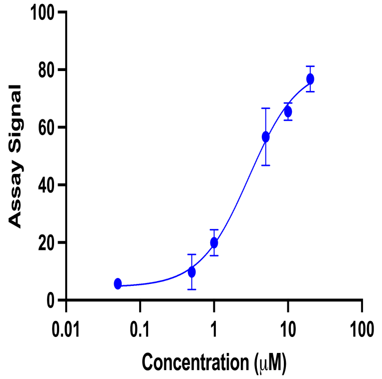

273	Q8WTS1	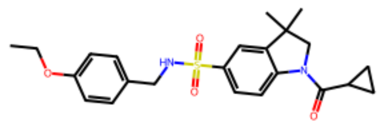	13.62	-1.555	0.9489	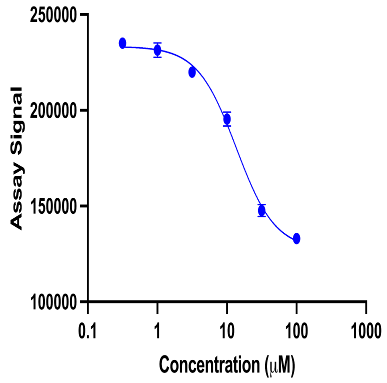

Supplementary Table 6 Dose-response curves of a representative bioactive compound for each of the projects that were followed up with positive dose-response experiments. All dose response data were plotted and analyzed using Prism 9.4.1 from GraphPad. Data (IC50/EC50) were fit using variable slope (four parameter) fits. and plotted as log[inhibitor] (μM) vs response. All Kd data were fit using One Site – Total (saturation) fits and plotted as log[inhibitor] (μM) vs response. '*' indicates Kd rather than IC_{50}.

Project ID	UniProt ID	Research Area	# of hits advanced to analog expansion	# hits validated	# analogs validated	Potency range (IC50/Ki, uM)	Units
056	Q66479	Infectious Diseases	2	0	0	-	IC50
057	O96693	Infectious Diseases	5	2	10	3.42 - 13.83	IC50
062	P31350	Oncology	3	2	9	3 - 40	IC50

080	Q07812	Neurotoxicity, cardiotoxicity, Alzheimer's Disease. ALS	4	2	6	-	Other
120	P05121	Rheumatology	5	2	7	89.12 - 626.8	IC50
126	Q96LB1	Immunology	1	1	6	5.9 - 78.9	IC50
141	P31483	Neurology	1	0	0	-	IC50
144	P34913	Neurology	3	2	12	0.004 - 0.068	IC50
158	P9WNM7	Infectious Diseases	3	3	31	1 - 23	IC50
159	I6Y9J2	Infectious Diseases	3	2	17	4.3 - 30.3	MIC
166	P68133	Oncology	1	0	0	-	Other
182	Q9BZS1 / Q13469	Immunology	3	2	15	1.053 - 8.263	IC50
198	P47895	Oncology	4	3	3	0.25 - 1.9	IC50
222	O15294	Oncology	2	2	5	15 - 38	IC50
227	P0C8L0	Infectious Diseases	1	1	3	10 - 80	IC50
243	O00522	Cardiology and Vascular Diseases	1	0	0	-	IC50
252	Q92793	Oncology	6	3	14	0.3 - 78.6	IC50

254	O95786	Infectious Diseases	2	1	1	0.083	IC50
258	O14924	Pain	2	2	13	10 - 104	IC50
267	P0DTC2	Infectious Diseases	1	0	0	-	IC50
276	Q8N9F0	Rare Diseases and Disorders	5	3	8	1.61 - 5.1	Ki

Supplementary Table 7. Dose-response results from the analog expansion for 21 AIMS projects in the AIMS-validation follow up studies. In addition to establishing dose-responsive behavior, additional measurable activities of analogous compounds further validate the primary hits.

Compound ID	Identified hit	Hit potency (Kd, nM)	Nearest Neighbor (NN)	NN Similarity (ECFP4, Tanimoto)	NN potency (Kd/IC50,nM)
LATS1_HID_1	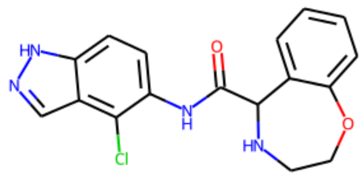	77	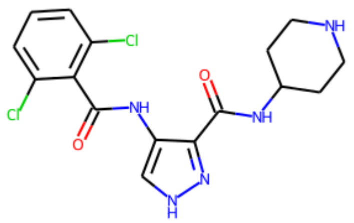	0.23	10000.0
LATS1_HID_2	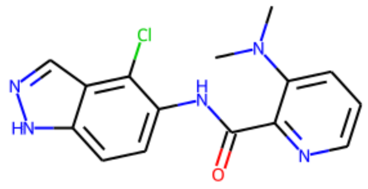	86	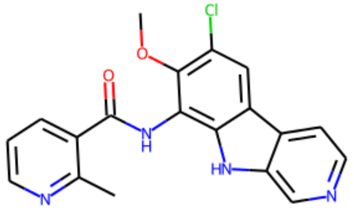	0.29	10000.0

LATS1_HID_3	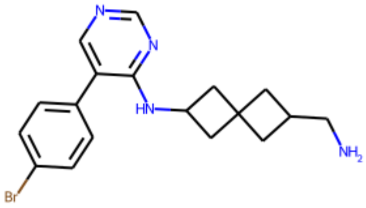	120	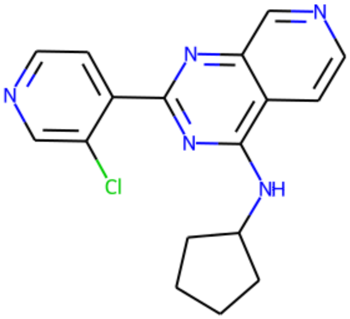	0.23	105.7
LATS1_HID_4	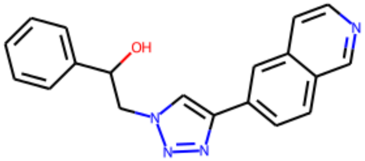	130	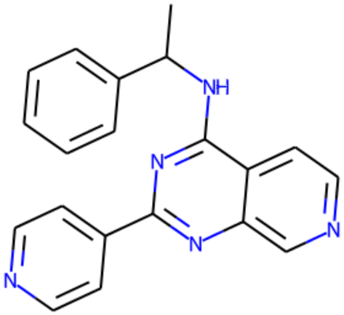	0.25	2691.5

LATS1_HID_5	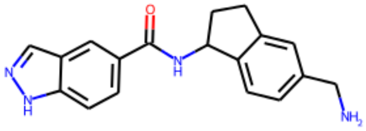	210	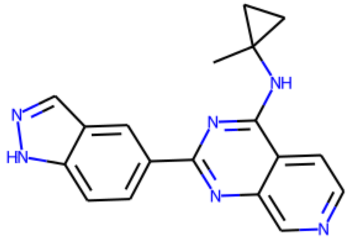	0.26	14.0
LATS1_HID_6	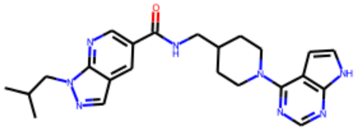	290	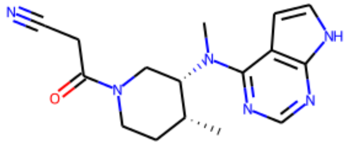	0.31	10000.0

LATS1_HID_7	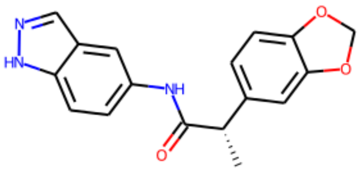	360	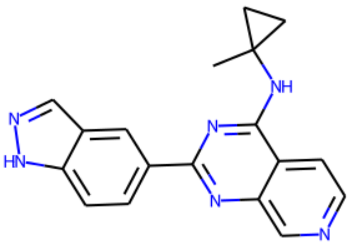	0.30	14.0
LATS1_HID_8	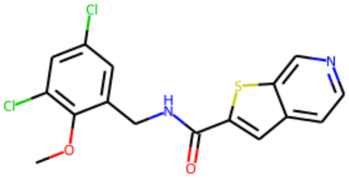	400	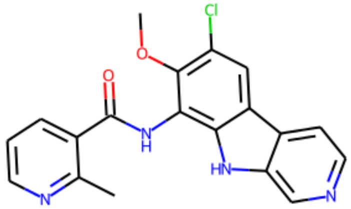	0.32	10000.0

LATS1_HID_9	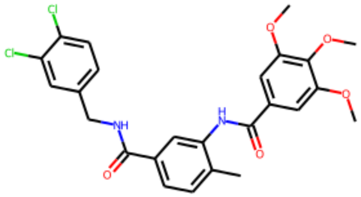	460	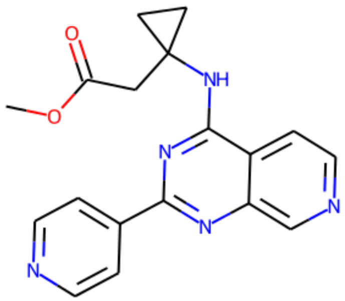	0.23	251.2
LATS1_HID_10	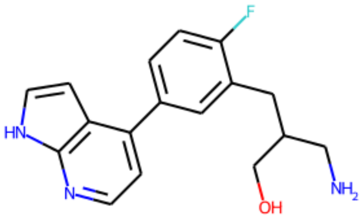	560	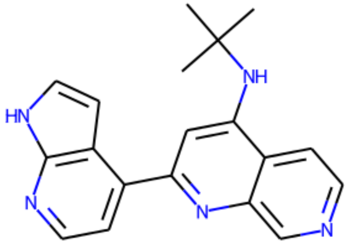	0.30	4.0

Supplementary Table 8. Top 10 active compounds identified for LATS1 in the hit-identification screen. 16 billion Enamine REAL compounds were screened with the AtomNet® model and 418 top-scoring diverse scaffolds were selected for testing in a KinomeScan binding assay. Of the 76 compounds with more than 50% inhibition at 30μM, 75 showed dose-responsive behavior ranging from 0.077μM to 82μM (KinomeScan binding assay). Most similar LATS1 compounds in the Atomwise database for each of the compounds are shown. The similarity between hits and database compounds (NN) is based on the Tanimoto similarity and ECFP4 fingerprints. NN Potencies are the median activities in the Atomwise database.

Compound ID	IC50 / Kd (μM)	Hill Slope	R^2	Dose-response curve
LATS1_HID_1	0.08381	-1.2020	0.9872	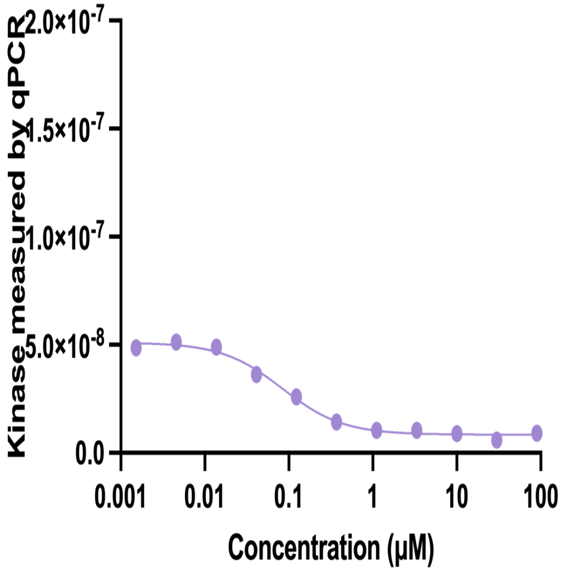

LATS1_HID_2	0.05546	-1.7780	0.7738	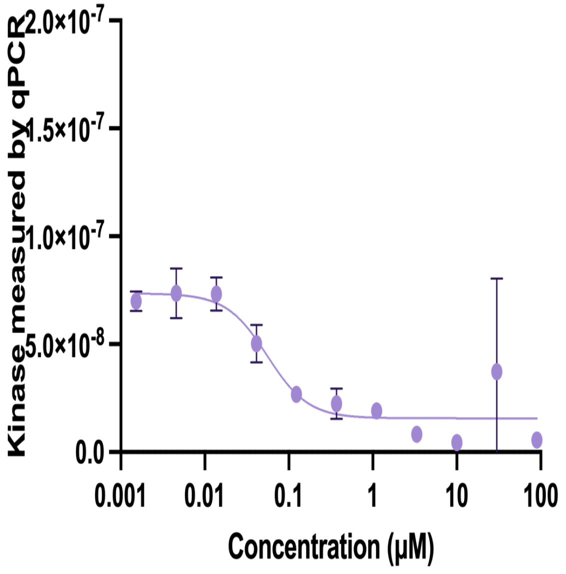

LATS1_HID_3	0.07242	-0.5208	0.9387	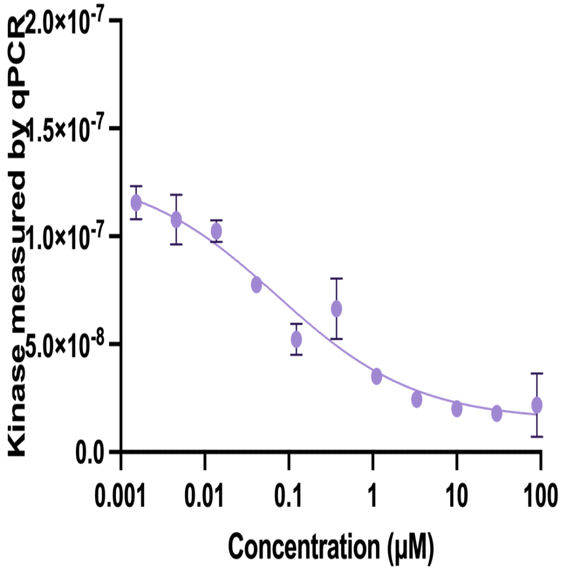

LATS1_HID_4	0.12520	-1.0700	0.9071	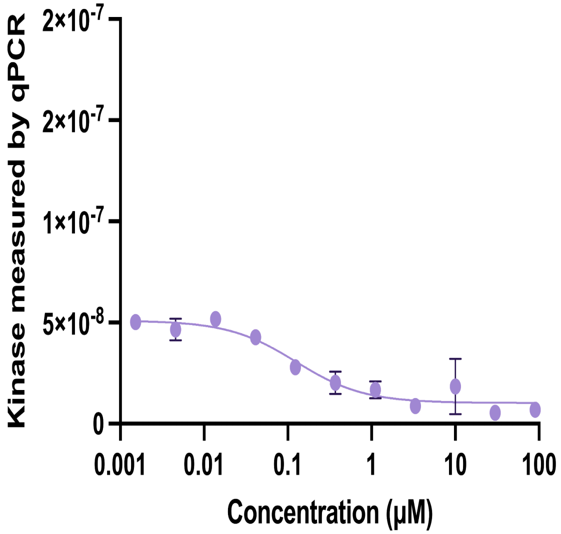

LATS1_HID_5	0.31550	-0.6241	0.8222	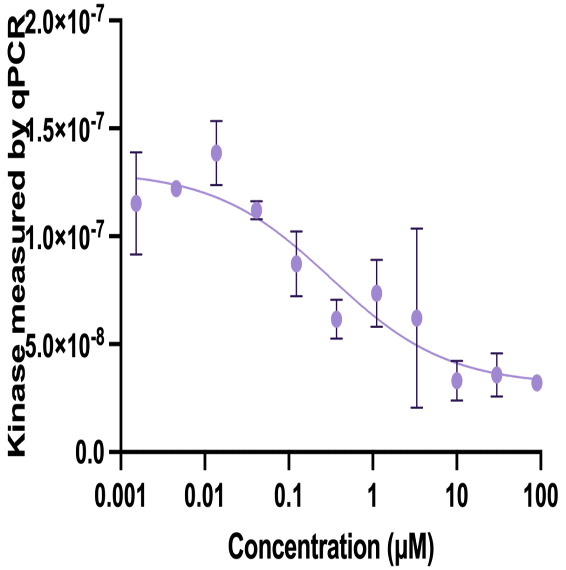

LATS1_HID_6	0.26590	-0.7033	0.9387	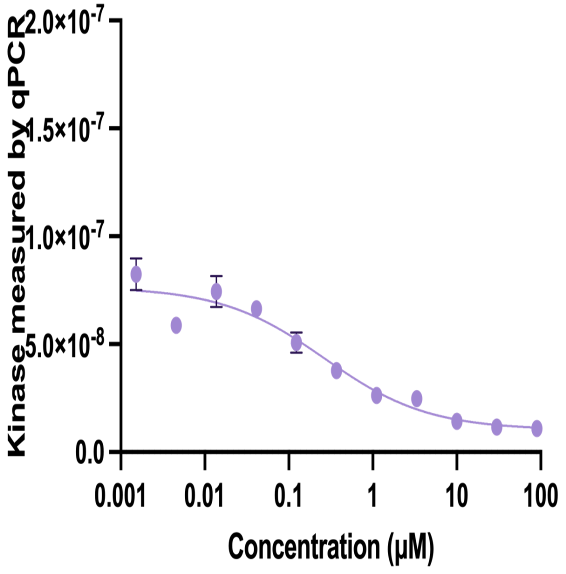

LATS1_HID_7	0.22750	-0.9714	0.8887	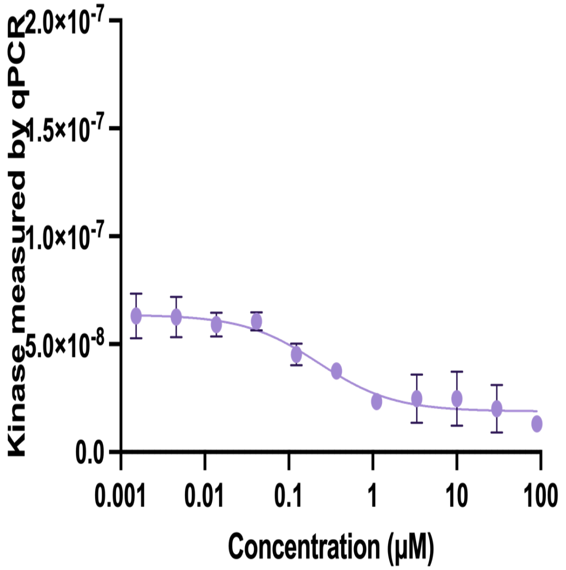

LATS1_HID_8	0.41860	-1.1130	0.9471	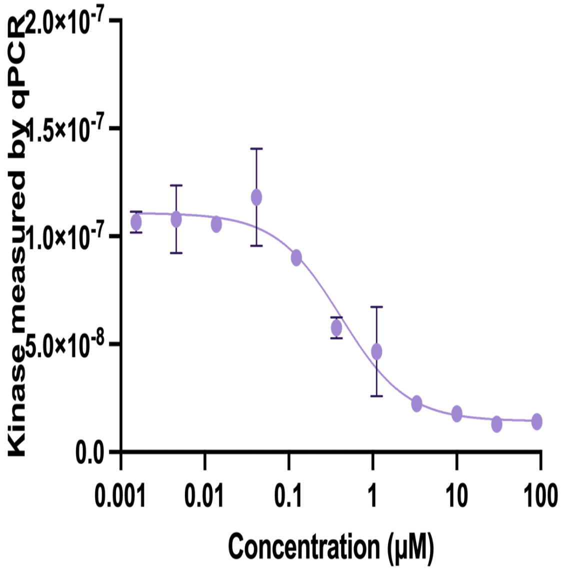

LATS1_HID_9	0.34360	-0.9266	0.9214	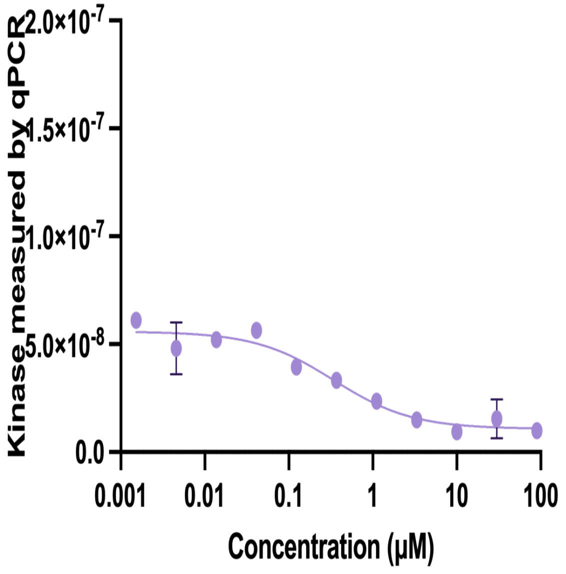

LATS1_HID_10	0.31120	-1.8580	0.8428	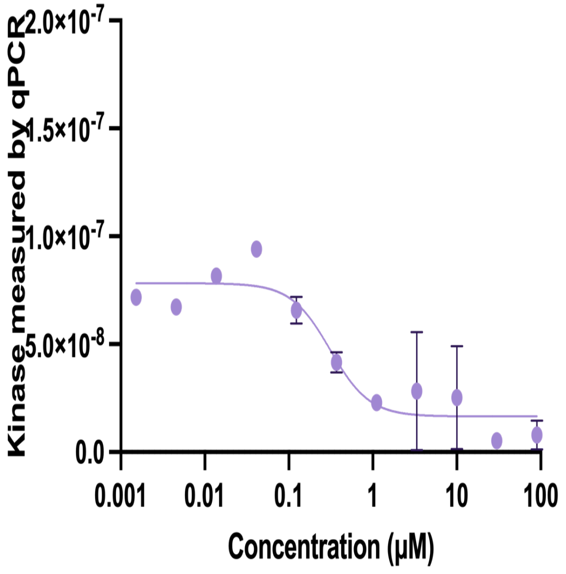

LATS1_HVE_1	0.03417	-2.2440	0.9913	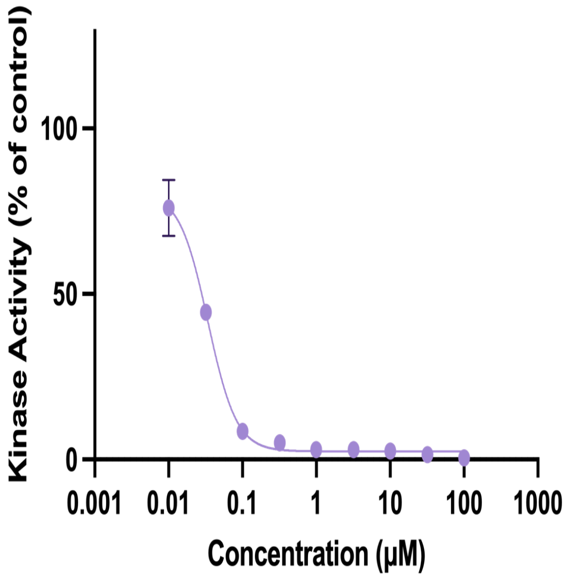

LATS1_HVE_2	0.02283	-0.6026	0.9925	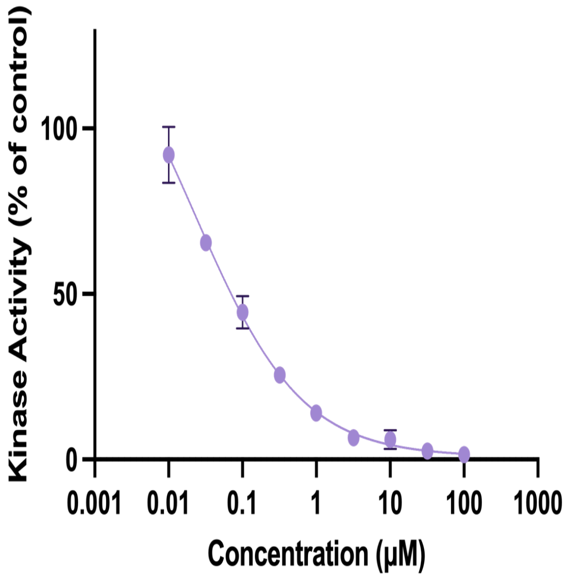

LATS1_HVE_3	0.07637	-1.0110	0.9969	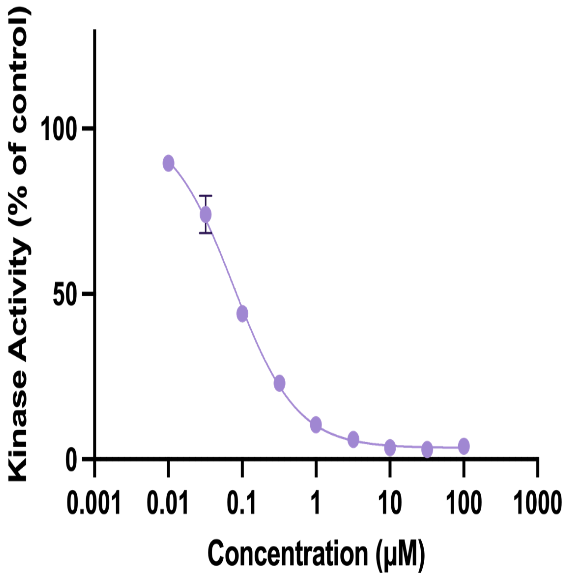

LATS1_HVE_4	0.10070	-1.4560	0.9551	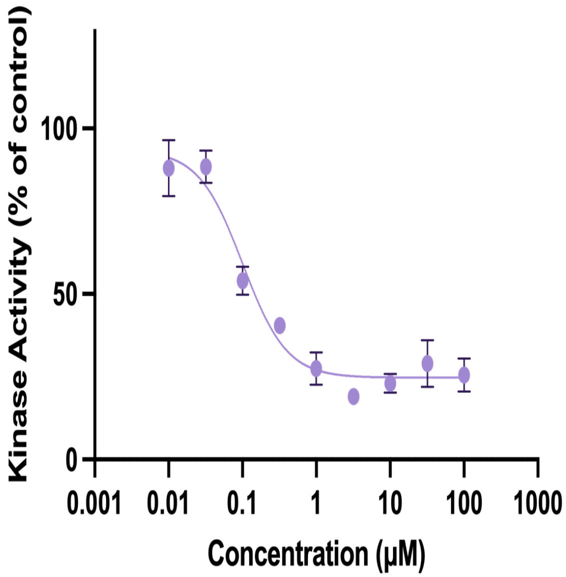

LATS1_HVE_5	0.10960	-1.2570	0.9887	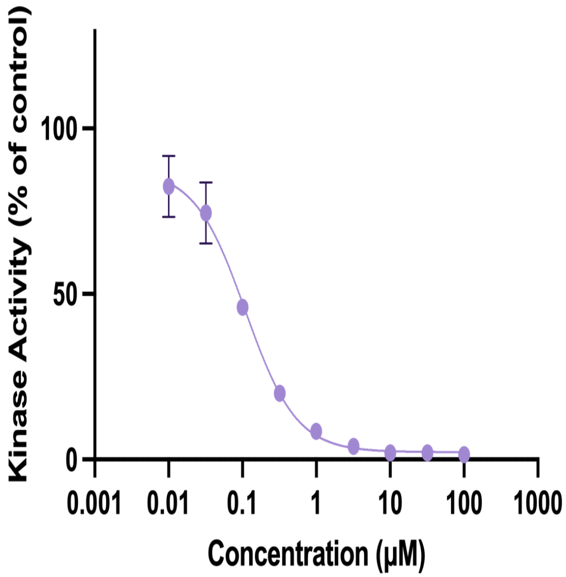

LATS1_HVE_6	0.01963	-0.5483	0.9822	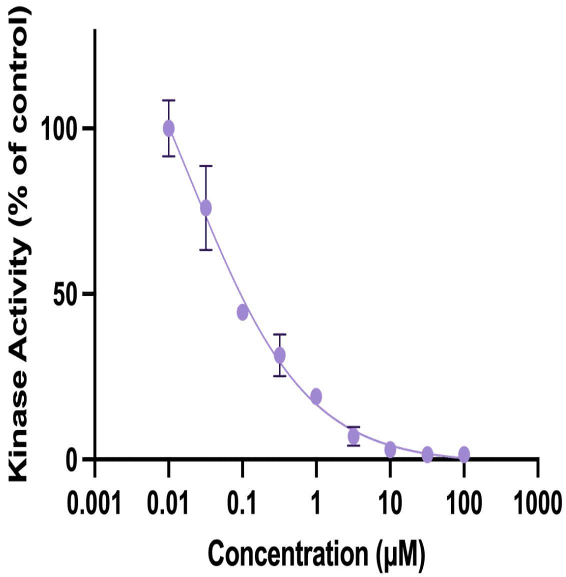

LATS1_HVE_7	0.11830	-0.6160	0.9848	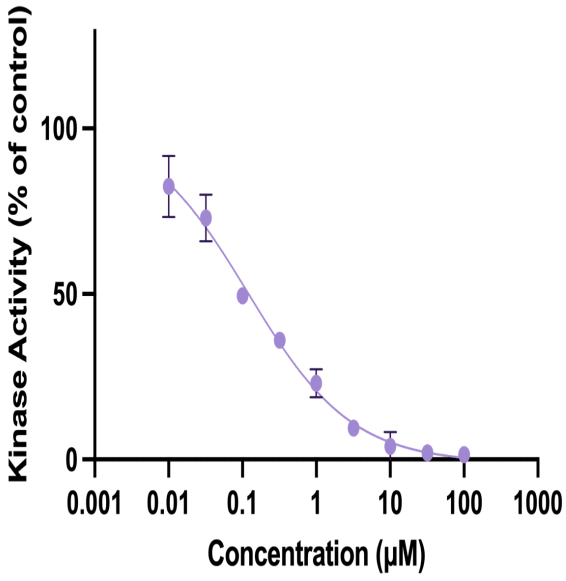

LATS1_HVE_8	0.14250	-1.1920	0.9913	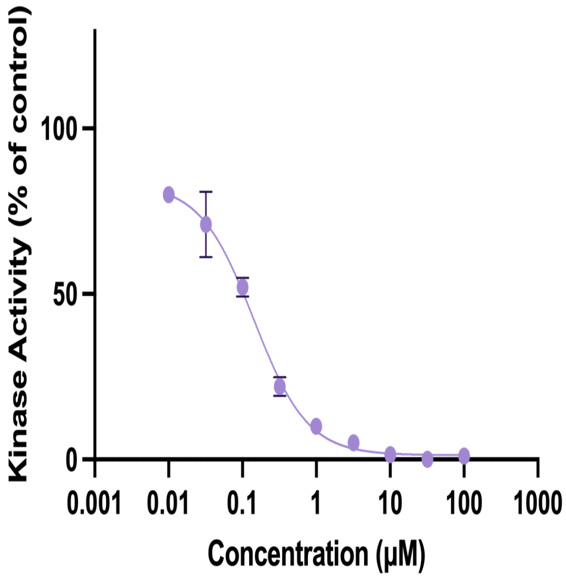

LATS1_HVE_9	0.15350	-0.9336	0.9971	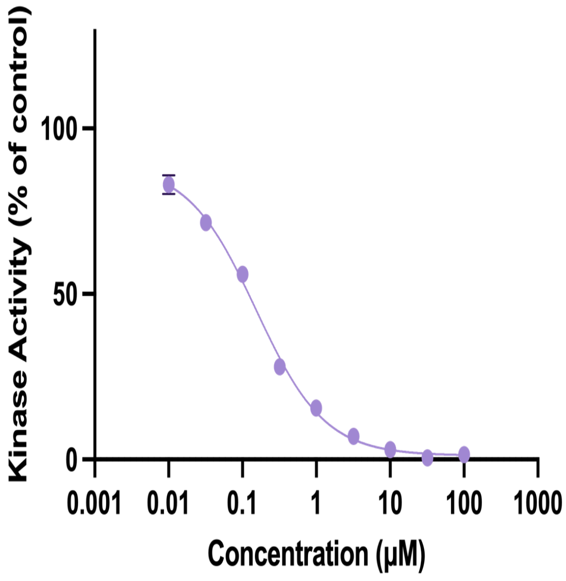

LATS1_HVE_10	0.19890	-1.0870	0.9814	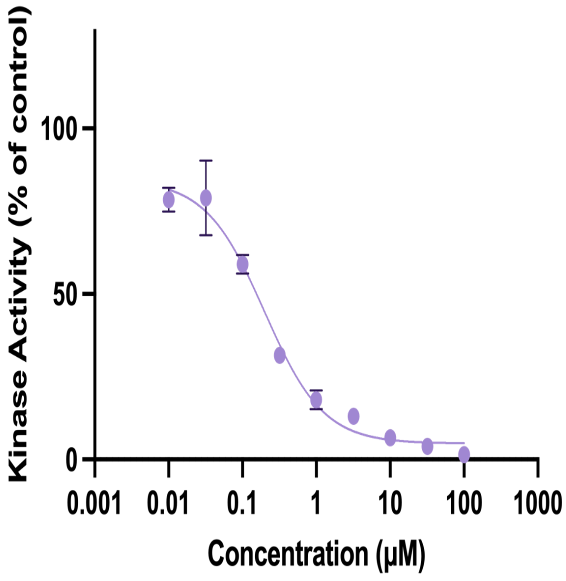

VCP_HID_1	4.31500	1.2110	0.9957	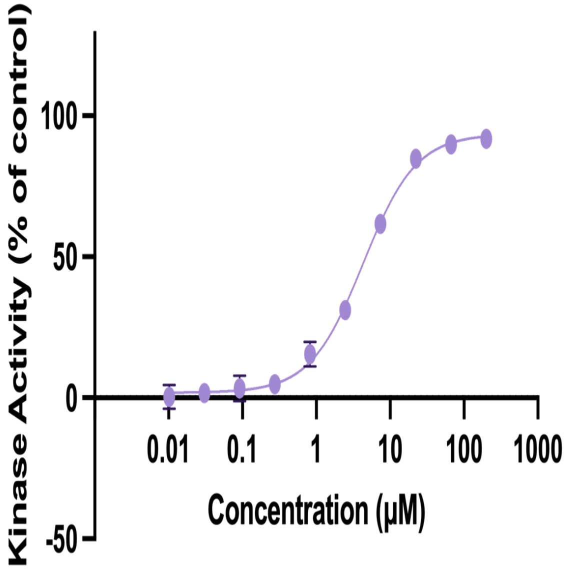

VCP_HID_2	20.81000	1.1450	0.9927	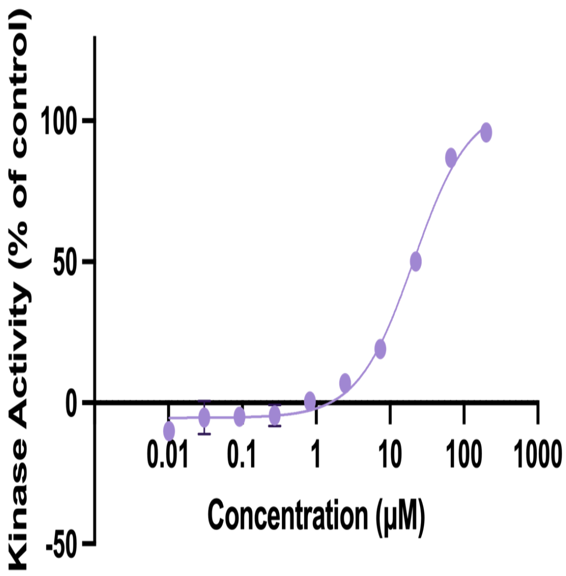

VCP_HID_3	42.31000	1.6770	0.9923	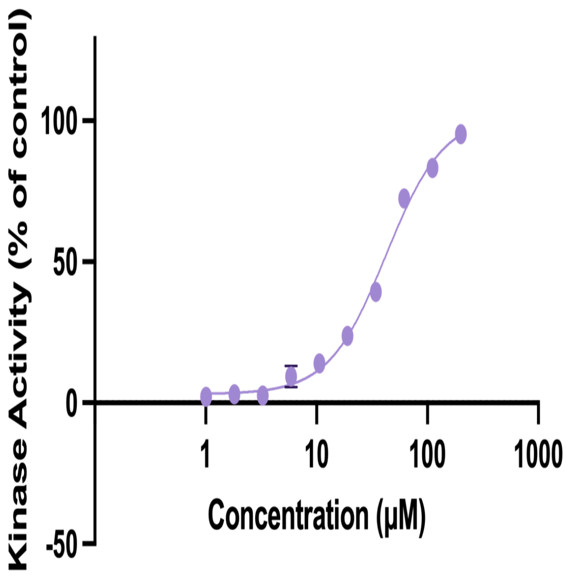

VCP_HID_4	14.08000	1.3260	0.9840	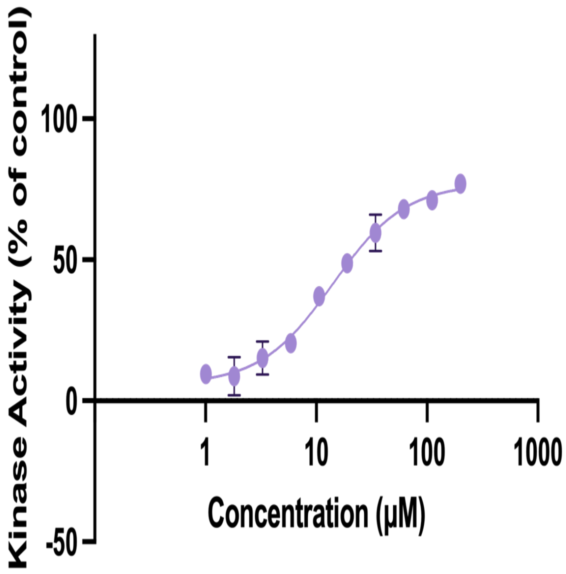

VCP_HID_5	7.46800	2.0280	0.9967	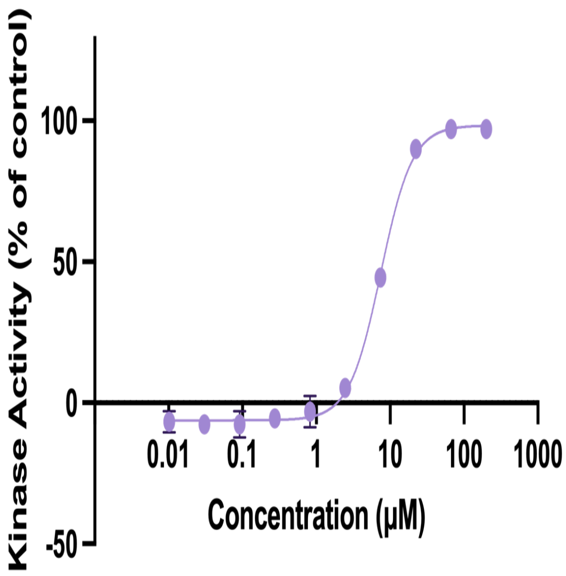

VCP_HID_6	12.58000	1.1450	0.9966	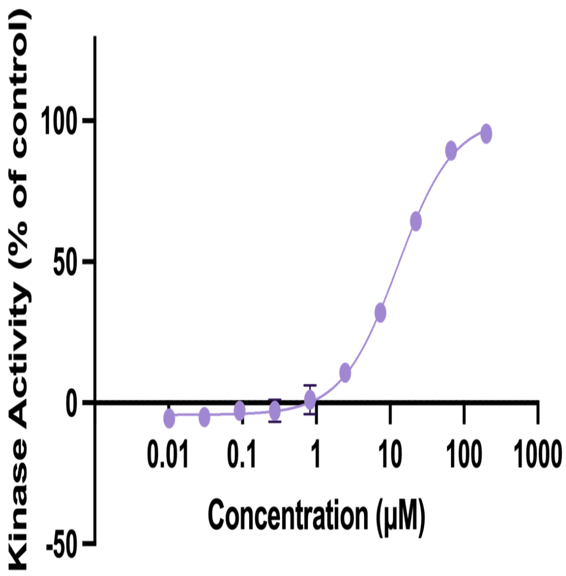

VCP_HID_7	22.92000	1.9530	0.9905	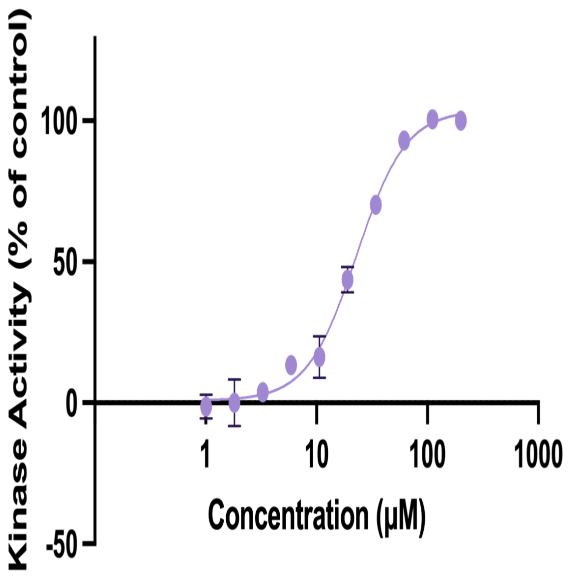

LATS1_HVE_PARENT_1	0.14250	-1.1920	0.9913	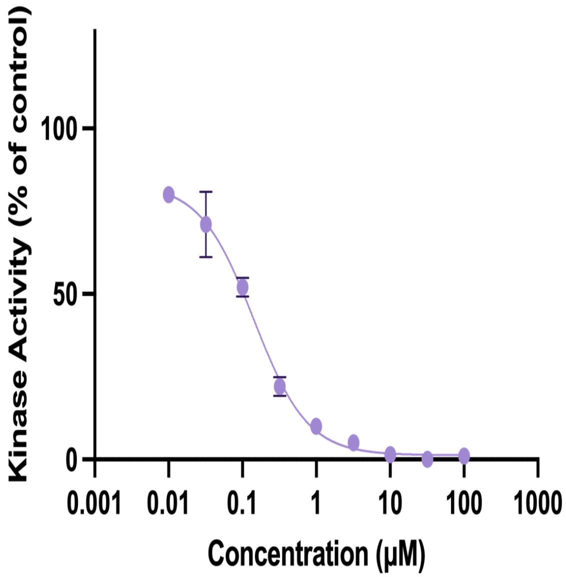

LATS1_HVE_PARENT_2	0.01963	-0.5483	0.9822	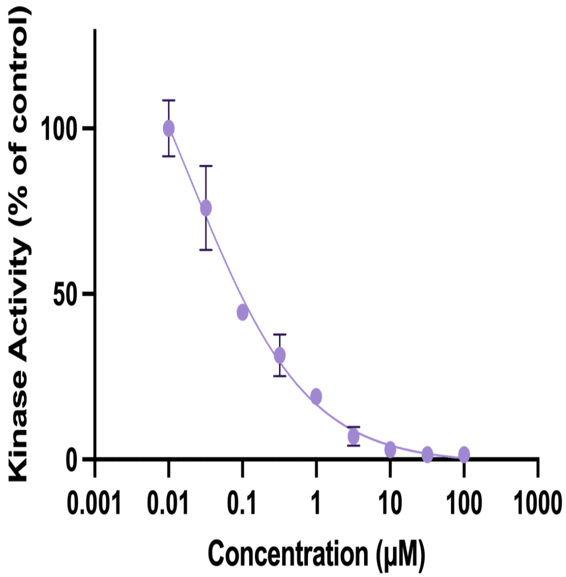

LATS1_HVE_PARENT_3	4.81600	-0.7635	0.9851	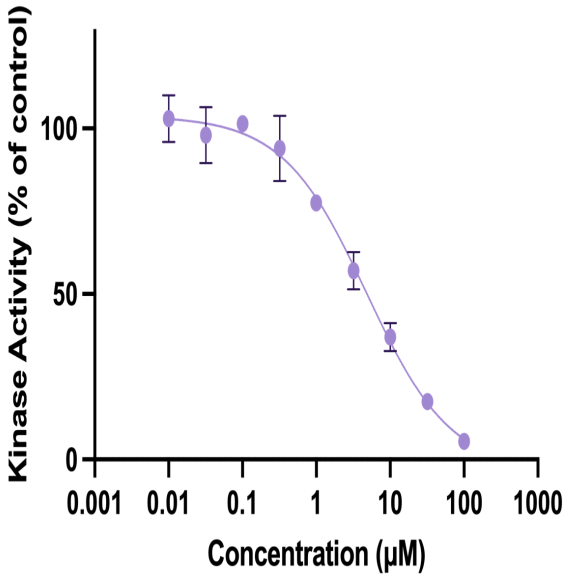

LATS1_HVE_PARENT_4	0.94910	-1.0730	0.9777	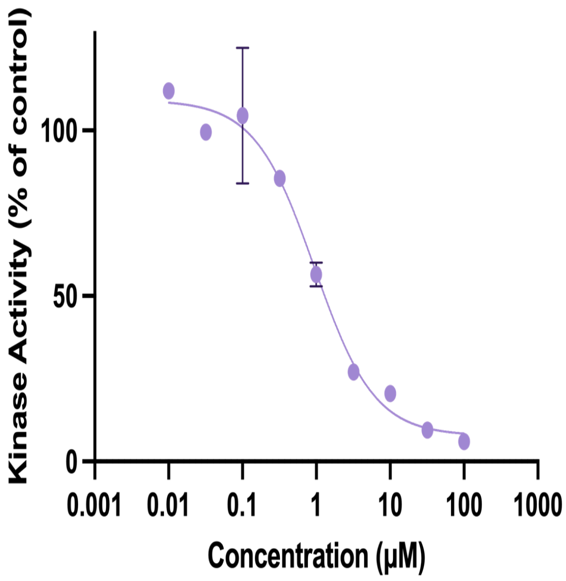

LATS1_HVE_PARENT_5	2.20600	-0.9670	0.9625	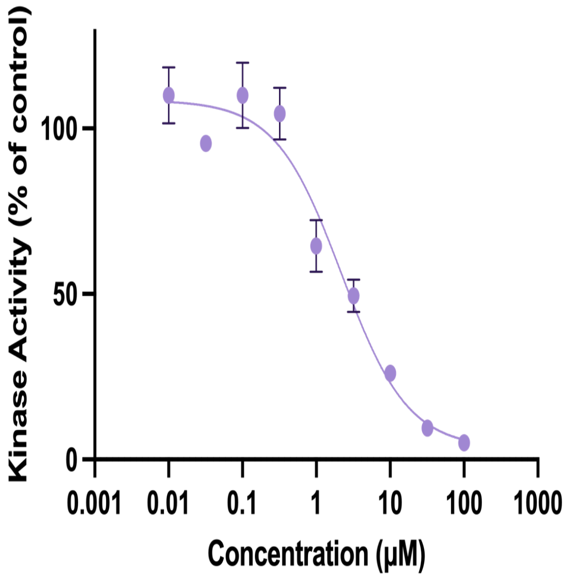

LATS1_HVE_PARENT_6	3.80900	-1.1620	0.9700	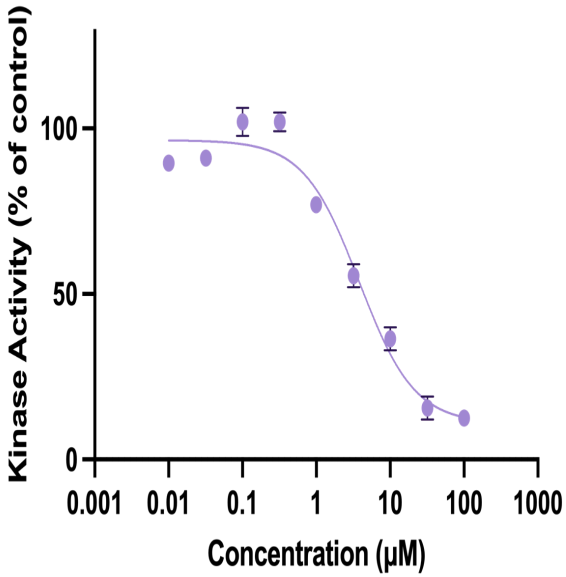

LATS1_HVE_PARENT_7	2.94700	-0.9894	0.9876	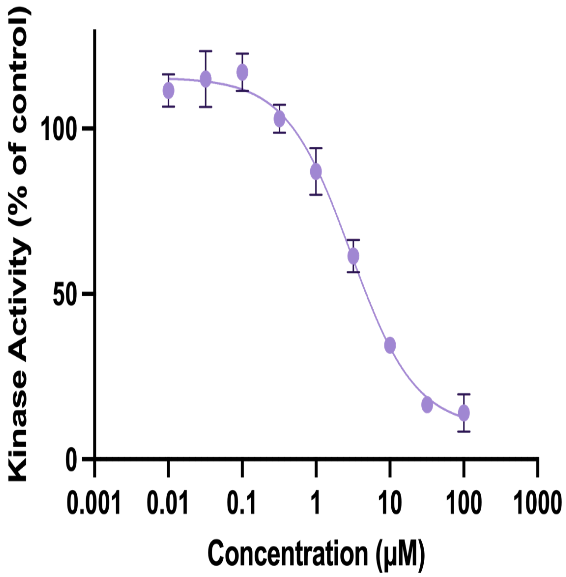

LATS1_HVE_PARENT_8	4.26100	-1.1340	0.9559	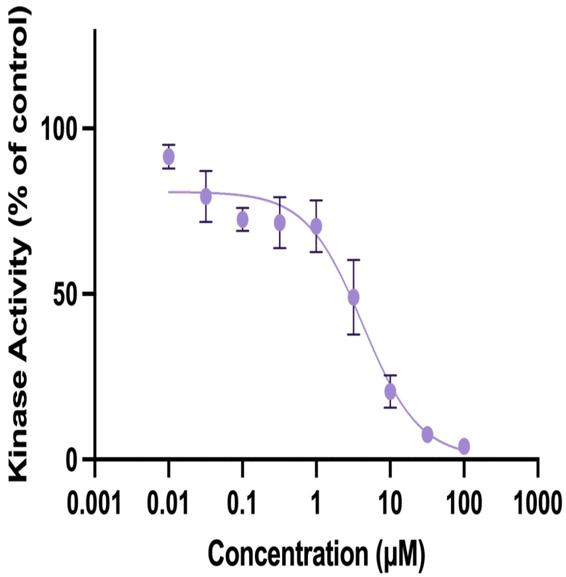

LATS1_HVE_BEST_1	0.10070	-1.4560	0.9551	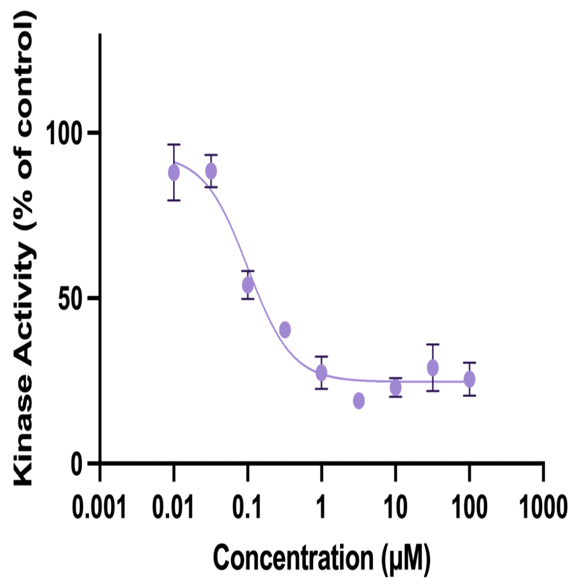

LATS1_HVE_BEST_2	0.07637	-1.0110	0.9969	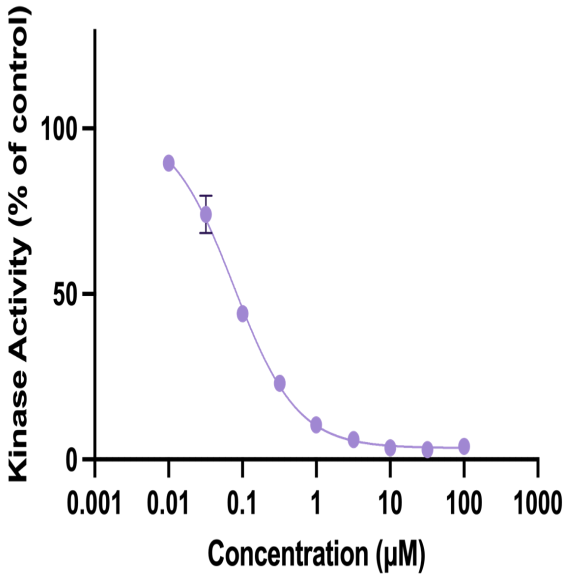

LATS1_HVE_BEST_3	0.02283	-0.6026	0.9925	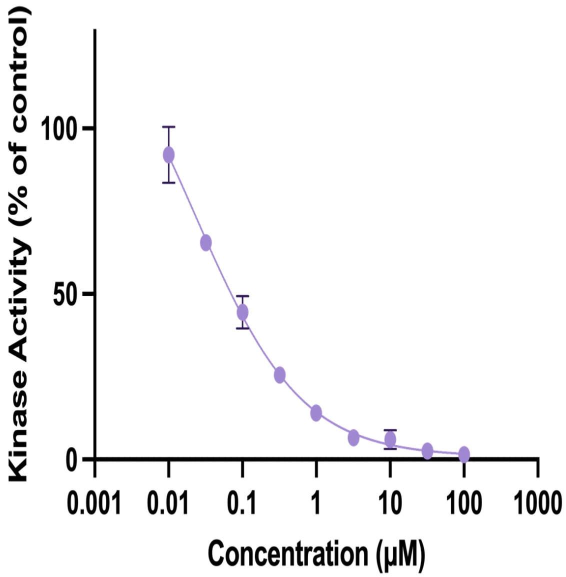

LATS1_HVE_BEST_4	0.92020	-0.3087	0.9599	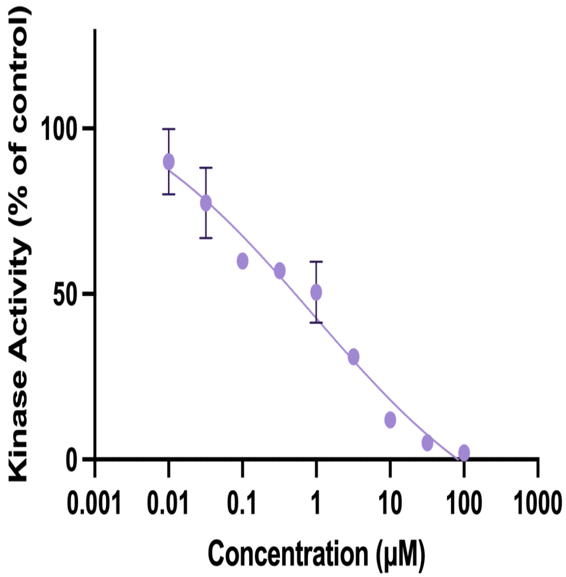

LATS1_HVE_BEST_5	0.03417	-2.2440	0.9913	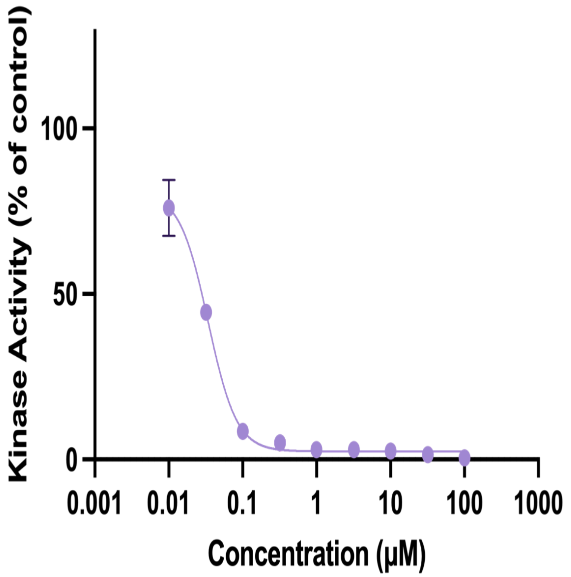

LATS1_HVE_BEST_6	0.39100	-0.4623	0.9711	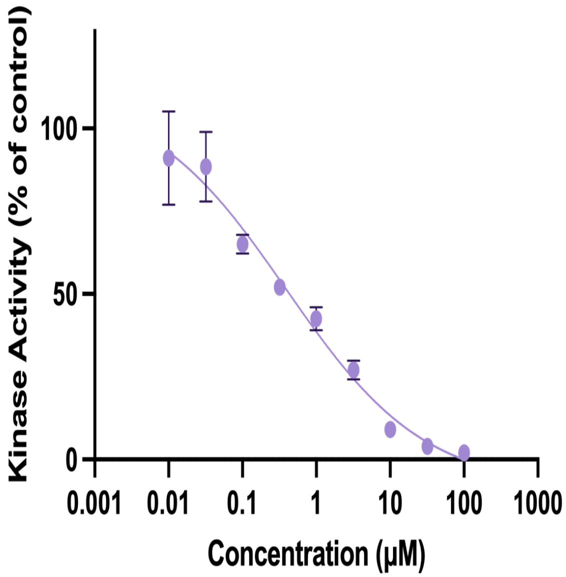

LATS1_HVE_BEST_7	0.92570	-0.8672	0.9503	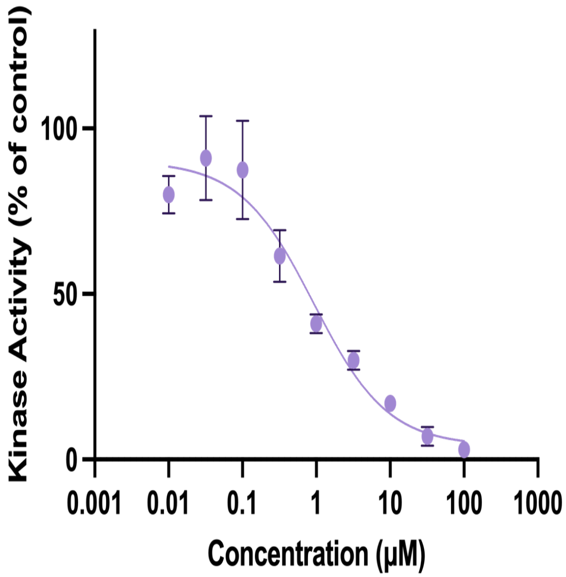

LATS1_HVE_BEST_8	0.19890	-1.0870	0.9814	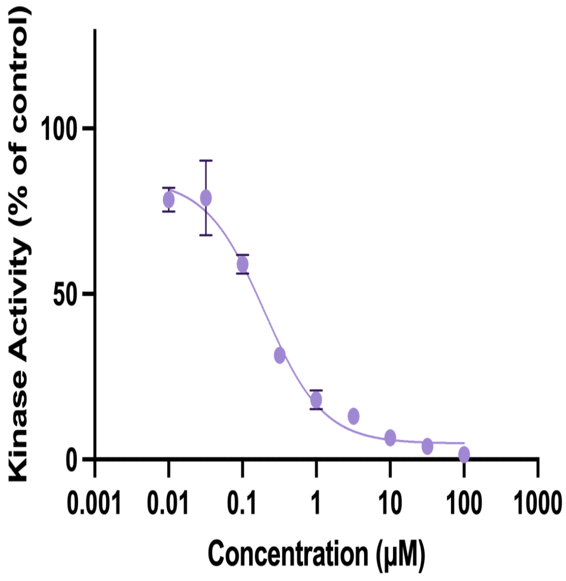

Supplementary Table 9. Dose-response curves of the bioactive compounds reported for LATS1 and VCP in the Internal Portfolio Validation section. Curve plots, hill slopes, R^2, and IC50 or Kd values were generated using Prism[2].

[2] GraphPad Software, San Diego, California USA, www.graphpad.com

Compound ID	10 most potent analogs	Analog potency (IC50, nM)	Nearest Neighbor (NN)	NN Similarity (ECFP4, Tanimoto)	NN potency (Kd/IC50,nM)
LATS1_HVE_1	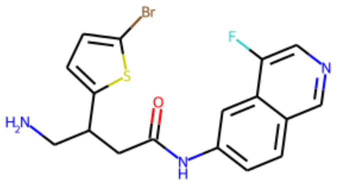	34	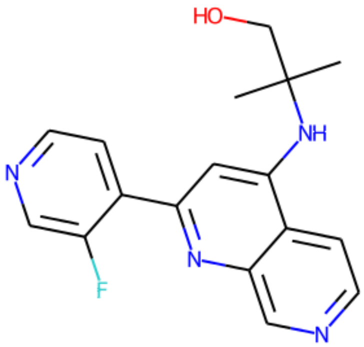	0.23	20.0
LATS1_HVE_2	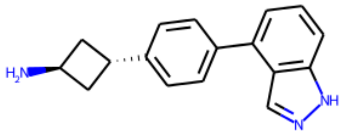	75	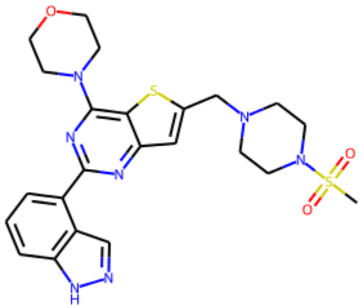	0.28	10000.0

LATS1_HVE_3	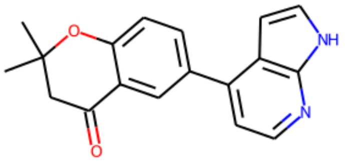	79	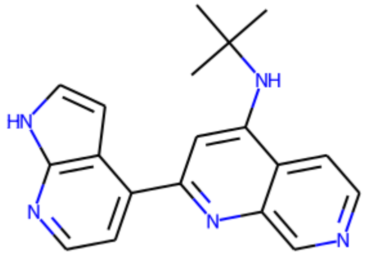	0.33	4.0
LATS1_HVE_4	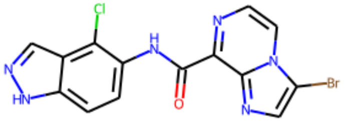	101	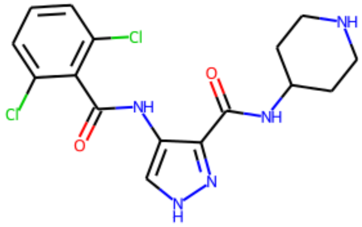	0.23	10000.0

LATS1_HVE_5	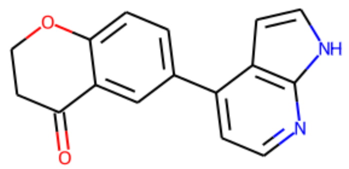	110	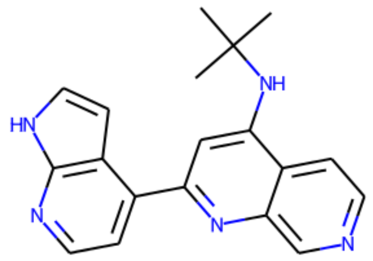	0.33	4.0
LATS1_HVE_6	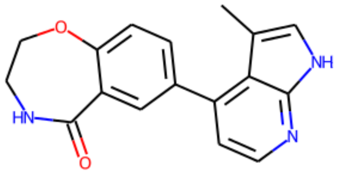	118	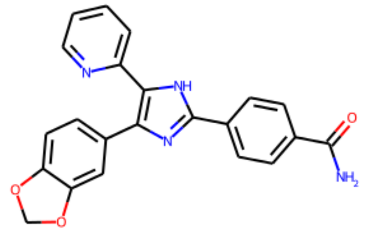	0.23	10000.0

LATS1_HVE_7	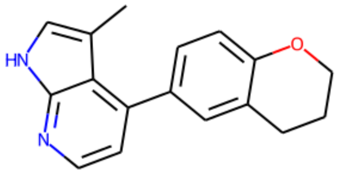	122	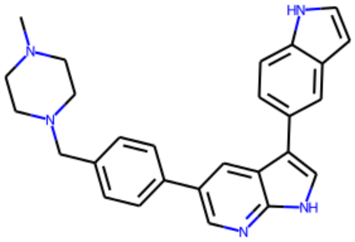	0.26	NA
LATS1_HVE_8	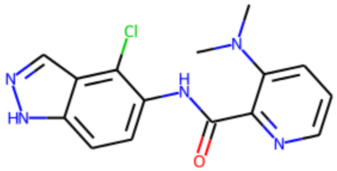	143	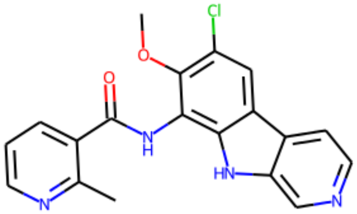	0.29	10000.0

LATS1_HVE_9	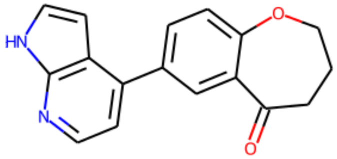	154	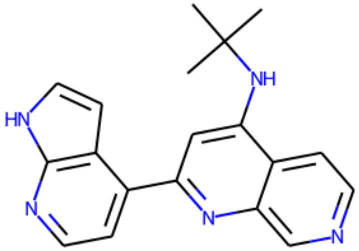	0.32	4.0
LATS1_HVE_10	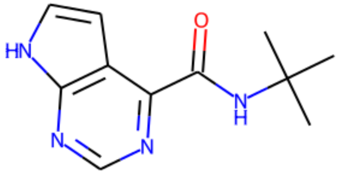	199	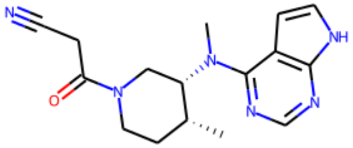	0.31	10000.0

Supplementary Table 10. Top 10 active compounds identified for LATS1 in the analog expansion. We selected 8 compounds for analog expansion for a total of 834 analogs. Of the 834 analogs tested in a KinaseProfiler functional assay, 435 showed more than 50% inhibition at 50μM, out of which 382 demonstrated DR behavior ranging from 0.034μM to 76μM. Most similar LATS1 compounds in the Atomwise database for each of the compounds are shown. The similarity between hits and database compounds (NN) is based on the Tanimoto similarity and ECFP4 fingerprints. NN Potencies are the median activities in the Atomwise database.

Compound ID	Most potent analog	Analog potency (IC50, nM)	Parent ID	Parent scaffold	Parent potency (IC50,nM)
LATS1_HVE_BEST_1	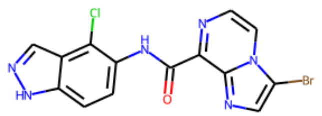	100.7	LATS1_HVE_PARENT_1	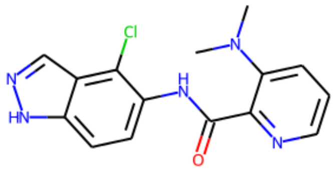	142.5
LATS1_HVE_BEST_2	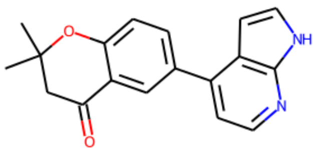	78.6	LATS1_HVE_PARENT_2	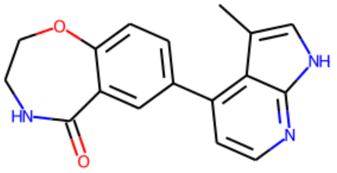	117.5

LATS1_HVE_BEST_3	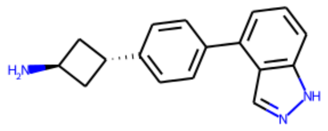	75.3	LATS1_HVE_PARENT_3	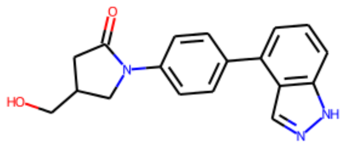	4894
LATS1_HVE_BEST_4	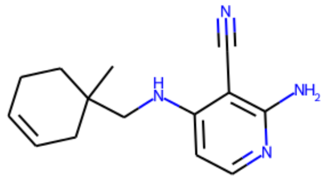	472.9	LATS1_HVE_PARENT_4	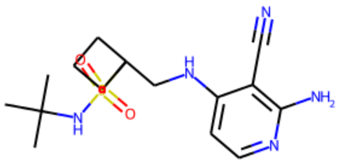	1112.2
LATS1_HVE_BEST_5	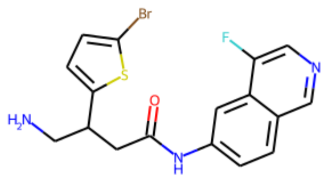	34.2	LATS1_HVE_PARENT_5	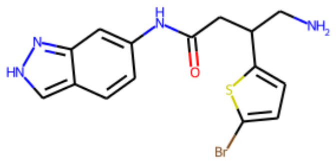	2608

LATS1_HVE_BEST_6	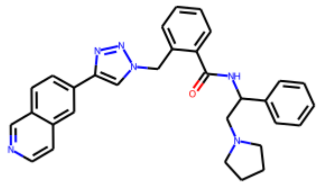	428.0	LATS1_HVE_PARENT_6	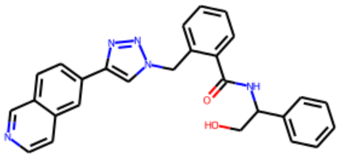	3809.4
LATS1_HVE_BEST_7	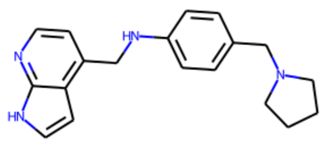	925.6	LATS1_HVE_PARENT_7	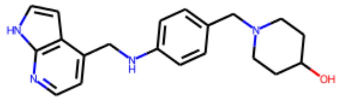	3938.3
LATS1_HVE_BEST_8	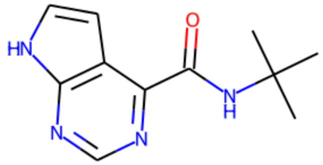	198.9	LATS1_HVE_PARENT_8	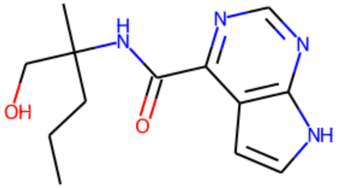	4261.3

Supplementary Table 11. An illustration of the structural relations between the 8 LATS1 scaffolds selected for analog expansion and the most potent compound identified for the respective analog series.

Compound ID	Identified hit	MOA (competitive, uncompetitive, noncompetitive)	Hit potency (Kd, uM)	Nearest Neighbor (NN)	NN Similarity (ECFP4, Tanimoto)	NN potency (Kd/IC50,uM)
VCP_HID_1	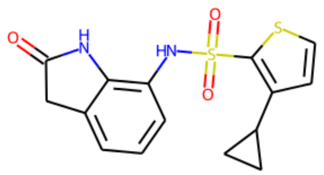	Noncompetitive	11.3	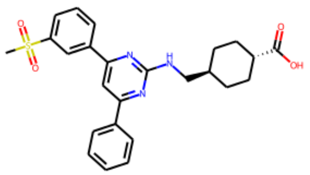	0.16	0.23
VCP_HID_2	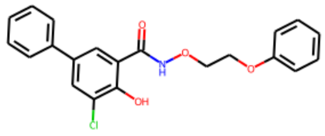	Noncompetitive	11.7	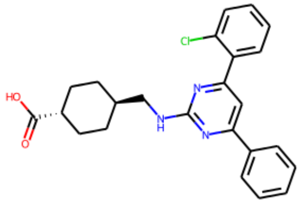	0.26	1.40

VCP_HID_3	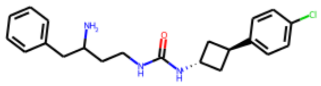	Noncompetitive	27.6	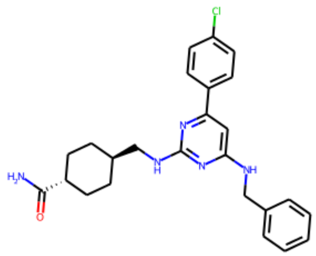	0.30	0.21
VCP_HID_4	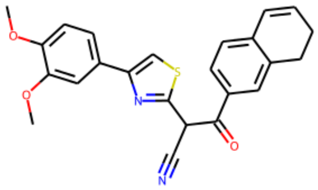	Competitive	33.9	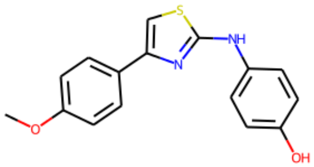	0.25	3.99
VCP_HID_5	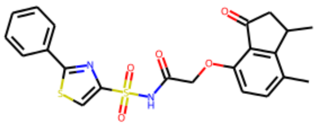	Competitive	56.2	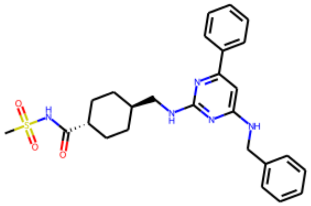	0.28	0.27

VCP_HID_6	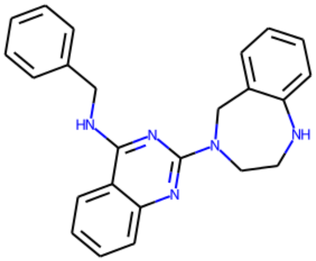	Competitive	2.9	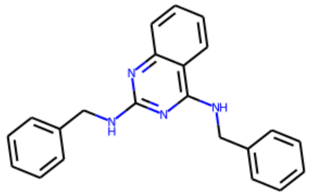	0.47	3.98
VCP_HID_7	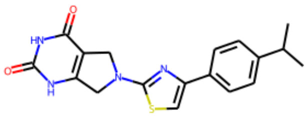	Uncompetitive	38.6	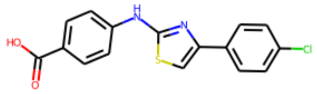	0.25	NA

Supplementary Table 12. Top seven compounds discovered in the VCP R155H experimental screens of the virtual screening predictions. Initially, 416 predictions representing a diverse set of novel chemicals were screened at 50 μM in the VCP R155H ADP-Glo bioluminescence assay, 20 of which were then advanced to dose-response studies with 19 showing dose-dependent inhibition (IC50s = 2.4 - 99.7 μM). Of the 19 compounds, seven compounds of interest were selected based on their potency and mechanism of action to be progressed to hit validation and expansion studies. Interestingly, one competitive compound (second from the last row of the table) is much closer to the boundary of our 0.5 Tanimoto distance cut-off than the other molecules, and the compound is more visually similar to its nearest neighbor than the other identified hits are to theirs.

Acknowledgments

Authors	Funding Acknowledgements
Adolfo A Ferrando	NIH [grants CA210065 and CA206501]
Adriano D. Andricopulo	The São Paulo Research Foundation (FAPESP), Brazil [CEPID-CIBFar grant 13/07600-3] and the National Council for Scientific and Technological Development, Brazil [grant 309844/2017-7]
Agnidipta Ghosh	NIH [grant S10 OD020068]
Alan Kie Leong Toh	QUT Centre for Genomics and Personalised Health Innovation grant
Alan V. Smrcka	NIH [grant R35GM127303]
Aleksandr Sverzhinsky	Canadian Institutes of Health Research Grants MOP142354 and PJT173370 to J.M.P.
Alessandra Mara de Sousa	PhD scholarship from Coordenação de Aperfeiçoamento de Pessoal de Nível Superior - Brasil (CAPES) [88887.604141/2021-00]
Alexander I. Agoulnik	NIDDK/NIH [grant 1R01DK110167]
Alexander N Freiberg	Institute for Human Infections & Immunity and Sealy & Smith Foundation
Alexander V. Statsyuk	NIH [grant R01GM115632]
Alexei Degterev	NIAID [grants R01AI144400 and R21AI164003]

Alice Vrielink	National Health and Medical Research Council of Australia [grant APP1078642]
Andrea Caporali	BHF-PG/16/58/32275 and RE/18/5/34216
Andrea Chini	PID2019-107012RB-100 (MICINN/FEDER) and AEI/10.13039/501100011033, "Severo Ochoa" Programme for Centres of Excellence in R&D (SEV-2017-0712)
Andrea Ilari	RAREST-JHD project [RF-2016-02364123]
Andrea Mattevi	Associazione Italiana per la Ricerca sul Cancro [AIRC; IG19808]
Andrea Trabocchi	Fondazione Cassa di Risparmio di Pistoia e Pescia (Bando Giovani@Ricerca scientifica 2018)
Andreas Stahl	NIH [grants R01 CA221916, UH3 DK120004, and R01 DK118940]
Andrew B. Herr	Cincinnati Children's Hospital and Innovation Fund Award
Andrew Freywald	Canadian Institutes of Health Research [Project Grant PJT-156401]
Andrew White	University of Michigan Center for the Discovery of New Medicines [UM-CDNM]
Anil K Ojha	NIH [grants AI132422, R21AI163599 and AI144474]
Anna A. L. Motyl	LifeArc/CSO [grant CSO-003]
Antonio Del Rio Flores	NIH [grant R01GM136758]
Antonio E. Garmendia	USDA/NIFA
Antonito T. Panganiban	CDMrC [grant PR182311]

Ariela Samantha	National Health and Medical Research Council of Australia [grant APP1078642]
Ashley L. St. John	Singapore Ministry of Education [grants MOE-T2EP30120-0011 and MOE2019-T2-1-146]
Bakary N'tji Diallo	Wellcome Trust [grant 107740/Z/15/Z/DELGEME]
Brian V. Geisbrecht	NIH [grant R35GM140852]
Bruce D Hammock	NIEHS [grant R35 ES030443] and NIEHS Superfund Program [P42 ES004699]
Bruno Eduardo Fernandes Mota	The author would like to thank UFMG, its intramural funds, and DRI for their support.
Camille Lempicki	Camille Lempicki was supported by grants from the Deutsche Forschungsgemeinschaft (DFG) to Tobias Hermle: HE 7456/3-1, HE 7456/4-1, and project-ID 431984000 – SFB 1453.
Carrie L. Partch	NIH [grant GM141849]
Chao-Yie Yang	NIH [grant R01HL141432]
Charlene M. Kahler	National Health and Medical Research Council of Australia [grant APP1078642]
Charles Karan	Department of Medicine, Columbia University Irving Medical Center
Charles Keller	Golf Fights Cancer
Cheryl Peltier	CancerCare Manitoba Foundation (CCMF), Manitoba Medical Services Foundation (MMSF), and University of Manitoba (UM)
Christian Weber	DFG SFB1123
Clara Reglero	The Leukemia & Lymphoma Society Special Fellowship

Daniel A. Keedy	NIH [grant R35GM133769]
David A. Hildeman	Cincinnati Children's Hospital and Innovation Fund Award
David H Drewry	The Chordoma Foundation and The Mark Foundation for Cancer Research
David J Dowling	U.S. National Institutes of Health (NIH)/ National Institutes of Allergy and Infectious Diseases (NIAID) awards, including Adjuvant Discovery (75N93019C00044) and NIH grant (1R21AI137932-01A1) to D.J.D.
David P. Siderovski	NIH [grants R01 DA048153 and U18 DA052497]
David Shum	MSIT 2017M3A9G6068254
Davide Maria Ferraris	Roche per la ricerca 2017
Deborah H Anderson	Canadian Institutes of Health Research (Funds 137154 and 179769)
Deirdre R Coombe	Centre for Genomics and Personalised Health Innovation Grant
Diana Ortiz	NIH [grant R33AI127591]
Dipayan Chaudhuri	NIH [grant R01HL165797]
Donald R. Ronning	NIH [grant R01AI105084]
Donghan Lee	James Graham Brown Cancer Center
Douglas William Zochodne	Canadian Institutes of Health Research [grant FRN15686]

Dylan M Glubb	QIMR Berghofer Proof of Concept Award
Edward C. Hsiao	Department of Medicine, University of California, San Francisco
Elena Lenci	Fondazione Cassa di Risparmio di Pistoia e Pescia (Bando Giovani@Ricerca scientifica 2018)
Emily R Mason	NIH [grant U54AG065181]
Enrico Petretto	Target Translation Consortium (TTC) and Duke-NUS Medical School
Eric J. Rubin	Bill and Melinda Gates Foundation INV-009173
Erick Strauss	NIH [grant R01AI136836]
Erik W. Thompson	Centre for Genomics and Personalised Health
Erkang Fan	NIH [grant R01 AI152358]
Erna Geessien Kroon	CNPq [grant 315750/2020-0] and FAPEMIG [grant PPM-00452-17]. The author would like to thank UFMG, its intramural funds, and DRI for their support.
Evripidis Gavathiotis	NIH/NCI [grant CA238229]
Franco J. Vizeacoumar	Canadian Institute of Health Research [grants PJT-178137 and PJT-156309]
Frank Luh	TMU Research Center of Cancer Translational Medicine of the Higher Education Sprout Project and the Ministry of Education (MOE) in Taiwan
Frederick S Buckner	NIH [grant R01 AI152358]
G. Marcela Rodriguez	New Jersey Health Foundation [Grant # PC 112-21]

Gaétan Mayer	Montreal Heart Institute Foundation
Geoffrey L. Greene	Ludwig Fund for Cancer Research
George A. Garcia	University of Michigan Center for the Discovery of New Medicines [UM-CDNM]
Gergely L. Lukacs	Canadian Institute of Health Research [grants PJT-153095 and PJT-173342], Cystic Fibrosis Foundation [grant LUKACS20G0], and Cystic Fibrosis Canada [grant 609247]
Gian Carlo G. Parico	Howard Hughes Medical Institute (HHMI) Gilliam Fellowship; UCSC Division of Graduate Studies
Giovanni Roti	AIRC Start-up Investigator Grant [n. 17107 GR], Leukemia Research Foundation, and IFAB Foundation
Giuseppe Fiermonte	Italian Association for Cancer Research [AIRC grant IG 2022 Id. 27121 to G.F.] and Italian Ministero dell'Istruzione, dell'Università e della Ricerca [grant no. 2017PAB8EM_002 to G.F.]
Gracjan Michlewski	Dioscuri, a programme initiated by the Max Planck Society, jointly managed with the National Science Centre in Poland, and mutually funded by Polish Ministry of Science and Higher Education and German Federal Ministry of Education and Research [2019/02/H/NZ1/00002 to G.M.]; Polish National Agency for Academic Exchange within Polish Returns Programme; National Science Centre [2021/01/1/NZ1/00001 to G.M.]
Guilherme Eduardo de Souza	FAPESP 2013/07600-3 and 2018/07287-7
Gustav Akk	NIH NIGMS [grant R35GM140947]

Guzmán Alvarez	grant I+D grupos CSIC UdelaR (ID_35)
Gökhan Cildir	The Hospital Research Foundation (THRF) Early Career Fellowship
Hai Li	Department of Medicine, Columbia University Irving Medical Center
Hamed Jafar-Nejad	NIH [grant R35GM130317]
Hannah P. Moore	NIH [grant R01AR062587]
Haynes (Shek Hei) Yuan	CancerCare Manitoba Foundation (CCMF), Terry Fox Research Institute (TFRI) and Canadian Institutes of Health Research (CIHR)
Heinrich Hoppe	Rhodes University Sandisa Imbewu grant, Grand Challenges Africa programme (GCA/DD/rnd3/032)
Helena Chaytow	My Name’5 Doddie Foundation [Ref: MN5DF Ox/Ed Feb 2019]
Holly Van Remmen	Department of Veteran Affairs Merit grant I01 BX004453 and OMRF funds
Hongyang Xu	Department of Veteran Affairs Merit grant I01 BX004453 and OMRF funds
Howard B. Lieberman	Department of Medicine, Columbia University Irving Medical Center
Hua-Ying Fan	NIH/NCI Cancer Center support grant [P30CA118100]
Hui Feng	Boston University Ignition Award, American Cancer Society [RSG-17-204-01-TBG], and National Cancer Institute [grant R01CA215059]
Hyeong Jun Kim	National Research Foundation of Korea [grant 2019R1A2C1085154]

Ingrid M Verhamme	NIH NHLBI [grant R01 HL130018]
Isin Cakir	The American Diabetes Association [grant no. 7-21-JDF-032]
J. Jefferson P. Perry	DoD [W81XWH-19-1-0327]
Jacob Ferreira	1 F31 HD105363
Jadel Müller Kratz	DNDi is grateful to its donors, public and private, who have provided funding for all DNDi activities since its inception in 2003.
James G. Granneman	NIH [grant R01DK076629]
James Shorter	NIH/NIGMS [grant R01GM099836]
Jarrod J. Mousa	University of Georgia
Jeffrey P. MacKeigan	National Cancer Institute [grants CA252430, CA197398 and CA263133]
Jia Zhou	NIH [grant R01 DA038446]
Jiabei Lin	AARF from Alzheimer's association, Warren Alpert Distinguished Scholar Award
Jiake Xu	Pathfinder, the University of Western Australia; NHMRC, APP1163933
Jianghai Wang	TMU Research Center of Cancer Translational Medicine of the Higher Education Sprout Project and the Ministry of Education (MOE) in Taiwan
Jinshi Zhao	AI139216
Jiyoun Lee	The National Research Foundation of Korea [NRF-2018R1D1A1B07042846]

Joana Reis	AIRC and European Union’s Horizon 2020 research and innovation programme under the Marie Skłodowska-Curie grant program [agreement No 800924.]
Jochen Buck	5 P50 HD100549
Joerg Stetefeld	Tier-1 Canada Research Chair, Canadian Institute of Health Research [CIHR-PJT 152935]
John J. Tanner	NIH [grant R01GM065546]
John M. Pascal	Canadian Institutes of Health Research [grant PJT173370]
Jose A. Costoya	Ministerio de Ciencia e Innovación (PID2020-113501RB-I00); Consellería de Cultura, Educación e Ordenación Universitaria (GPC GI-1862, ED431B 2020/26); Xunta de Galicia (Centro de investigación de Galicia accreditation 2019-2022; ED431G 2019/02) and the European Union (European Regional Development Fund – ERDF
Joseph J. Maciag	Cincinnati Children's Hospital and Innovation Fund Award
Joshua Alexander Nasburg	T32GM099608
Juan José Lasarte	Paula and Rodger Riney Foundation
Julian Milosavljevic	Julian Milosavljevic was supported by grants from the Deutsche Forschungsgemeinschaft (DFG) to Tobias Hermle HE 7456/3-1, HE 7456/4-1, and project-ID 431984000 – SFB 1453
Julijus Bogomolovas	Marie Sklodowska-Curie Individual Fellowships (Titin Signals, 656636)
Jung-Min Kee	National Research Foundation of Korea (2019R1A2C1085154)
Jung-Min Kee	2019R1A2C1085154

Jônatas Santos Abrahão	CNPq [grant 302081/2018-6], FAPEMIG [grant 27103] and CAPES [grant 88882.348380/2010-1]. The author would like to thank UFMG, its intramural funds, and DRI for their support.
Kasper B. Hansen	NIH-NINDS [grant NS116055 and NIH-NIHGMS [grant GM103546]
Kevin M. Hopkins	Department of Medicine, Columbia University Irving Medical Center
Khaled Machaca	Biomedical Research Program at Weill Cornell Medical College in Qatar (BMRP), a program funded by Qatar Foundation
Kirk James McManus	CIHR [Project Grant 162374]
Kiterie M E Faller	Wellcome Trust Institutional Translational Partnership Award [WT iTPA PIII-067] and LifeArc/CSO [grant CSO-003]
Kiyo Nagamori	Golf Fights Cancer
Konstantin V. Korotkov	NIH NCATS [grant UL1TR001998]
Kyung Hyeon Lee	NIH [grant R01HL132287]
Lajos Pusztai	The Susan G Komen Foundation [Leadership Grant SAC 160076]
Lari Lehtiö	Sigrid Juselius Foundation
Larisa M Haupt	Centre for Genomics and Personalised Health
Leila Su	TMU Research Center of Cancer Translational Medicine of the Higher Education Sprout Project and the Ministry of Education (MOE) in Taiwan
Ling Zhuo	Chinese Scholarship Council
Lizbeth Hedstrom	NIH NIAID [grant AI125362 to G.D. Cuny, University of Houston]

Lizbeth Hedstrom	NIH NIGMS [grant R01GM142041]
Lonny Levin	5 P50 HD100549
Luke Whitesell	Fellowship Grant from PRiME Initiative at University of Toronto
Maira Harume Nagai	NIH/NIDCD [grant DC016224]
Marc P. Lussier	Natural Sciences and Engineering Research Council of Canada [Discovery Grant # RGPIN-2017-05392]
Marc P. Windisch	National Research Foundation of Korea (NRF) grant funded by the Korean government (MSIT) (NRF-2020R1A2C2009529, NRF-2019R1A2C1090515, and NRF-2017M3A9G6068246).
Marco Lolicato	Fondazione Cariplo Grant# 2019-1809 -- “Programma per Giovani Ricercatori—Rita Levi Montalcini 2013” [project number: PGR16HTPSF]
Maria J. Macias	Ministerio de Ciencia, Innovación y Universidades -- BFU2017-82675-P
Marilia Valli	Fapesp #2019/05967-3
Marim M Barghash	Ontario Graduate Scholarship and Dorthy Sterling Dow Walsh Award
Mario Mellado	PID2020-114980RB-I00
Mark A. Wilson	NIH [grant R01GM139978]
Mark Hannink	University of Missouri Spinal Cord Injuries Research Program
Martin K Safo	NIH/NHLBI [grant R61HL156158] and NIH/NIMHD [grant R01MD009124]
Martin St. Maurice	NIH [grant R15 GM131356]

Mary Ann McDowell	Department of Defense Telemedicine and Advanced Technology Research Center (TATRC) awards #W81XWH-10-1-0085 and W81XWH-11-1-0415
Masfique Mehedi	NIH/NIGMS [grant P20GM113123]
Mateus Sá Magalhães Serafim	CAPES [grant number 88887.595578/2020-00] and the author would like to thank UFMG, its intramural funds, and DRI for their support.
Matthew B. Soellner	NIH [grant 5R01GM125881]
Maxim Skorodinsky	CancerCare Manitoba Foundation (CCMF), Manitoba Medical Services Foundation (MMSF), and University of Manitoba (UM)
Michael Mowat	CancerCare Manitoba Foundation and Cancer Research Society
Michael R Jackson	NIH [grant 5R01AG058446]
Mikell Paige	NIH [grant R01HL132287]
Natalia Stec	Dioscuri, a programme initiated by the Max Planck Society, jointly managed with the National Science Centre in Poland, and mutually funded by Polish Ministry of Science and Higher Education and German Federal Ministry of Education and Research [2019/02/H/NZ1/00002 to G.M.]; Polish National Agency for Academic Exchange within Polish Returns Programme; National Science Centre [2021/01/1/NZ1/00001 to G.M.]
Neha S. Gandhi	Center for Genomics and Personalised Health
Nicola Longo	Research grant from the Association for Creatine Deficiencies
Nirvan Rouzbeh	NIH-NINDS [grant NS116055] and NIH-NIHGMS [grant GM103546]

Njabulo Joyfull Gumede	SAMRC - Research Capacity Development Initiative for Historically Disadvantaged Institutions
Noureddine Ben Khalaf	AGU Intramural Grant LS_NB_18
Olli Silvennoinen	Academy of Finland, Sigrid Jusélius Foundation, Finnish Cancer Foundation, Jane and Aatos Erkko Foundation, Tampere Tuberculosis Foundation, and Pirkanmaa hospital district competitive research funding
Paul V. Fish	Alzheimer’s Research UK [520909]
Paulo Otávio Lourenço Moreira	PhD scholarship from Coordenação de Aperfeiçoamento de Pessoal de Nível Superior - Brasil (CAPES) [88887.473451/2020-00]
Pei Zhou	AI139216
Pengda Liu	Gabrielle’s Angel Foundation Medical Research Award
Percy Agogo-Mawuli	NIH [grants R01 DA048153 and U18 DA052497]
Peter L Jones	NIH/NIAMS [grant R01AR062587]
Philbert Ip	Mitacs Accelerate fellowship (IT09755)
Philipp von Hundelshausen	DFG SFB1123 A2
Pil H. Lee	University of Michigan Center for the Discovery of New Medicines [UM-CDNM]
Rafael Balaña-Fouce	SAF2017-83575-R
Rafael V. C. Guido	FAPESP 2013/07600-3 and 2020/12904-5

Rafaela Salgado Ferreira	CAPES, FAPEMIG [Rede Mineira de Imunobiologicos grant # REDE-00140-16], and CNPq Researcher Scholarship [Bolsa de Produtividade em Pesquisa, 310197/2021-0]. The author would like to thank UFMG, its intramural funds, and DRI for their support.
Rajendra K. Agrawal	NIH [grant R01 GM061576]
Rajesh Ramachandran	NIH [grant R01 GM121583]
Rajkumar Verma	START PPOC by Office of Vice president for Research at UConn
Remus T. Dame	Netherlands Organization for Scientific Research [VICI 016.160.613]; Leiden University-Gratama fund [2020–09/W20386-1-GSL]
Richard E. Taylor	NIH [grant R01GM129465] and Grace Science Foundation
Richard H. Ebright	NIH [grant GM041376]
Robert A. Batey	NSERC
Robert Blelloch	Patel Family Grant Award
Robert J Vandenberg	NIH [grant R01DA048879]
Robert J. Hickey	DoD CDRMP BCRP W81XWH-19-1-0327
Robert J. Kelm, Jr.	University of Vermont Cancer Center Pilot Project Award
Robert K. Bradley	Washington Research Foundation (Commercialization Program Phase 2 Award)

Roberto Solano	PID2019-107012RB-100 (MICINN/FEDER) and AEI/10.13039/501100011033, "Severo Ochoa" Programme for Centres of Excellence in R&D (SEV-2017-0712)
Ronan R McCarthy	BBSRC New Investigator Award BB/V007823/1 and Academy of Medical Sciences/the Wellcome Trust/ the Government Department of Business, Energy and Industrial Strategy/the British Heart Foundation/Diabetes UK Springboard Award [SBF006\1040]
Rosa Maria Reguera	SAF2017-83575-R
Ruben Vazquez Uribe	Novo Nordisk Foundation [grant NNF20CC0035580]
Rubens Lima do Monte-Neto	Minas Gerais State Agency for Research and Development – Fapemig [grant PPM-00699-18] and CNPq - Brazilian National Council for Scientific and Technological Development - Research fellowship [grant 312965/2020-6]
Ryan T. Cullinane	NIH NIGMS [grant R01GM142041]
Sachin Katyal	CancerCare Manitoba Foundation (CCMF), Terry Fox Research Institute (TFRI) and Canadian Institutes of Health Research (CIHR)
Samuel A Shelburne	NIH/NIAID [grant R01AI125292]
Scott M Landfear	NIH [grant R21 AI114842]
Seung-Yong Seo	NRF-2022R1A2C1092715
Shaoyou Chu	NIH [grant U54AG065181]
Shefali Chauhan	Golf Fights Cancer

Shireen R. Ashkar	University of Michigan Center for the Discovery of New Medicines [UM-CDNM]
Show-Ling Shyng	NIH [grant R01DK066485]
Silvia Buroni	Italian Ministry of Education, University and Research (MIUR) (Dipartimenti di Eccellenza, Program 2018–2022)
Siran Zhu	Dioscuri, a programme initiated by the Max Planck Society, jointly managed with the National Science Centre in Poland, and mutually funded by Polish Ministry of Science and Higher Education and German Federal Ministry of Education and Research [2019/02/H/NZ1/00002 to G.M.]; Polish National Agency for Academic Exchange within Polish Returns Programme; National Science Centre [2021/01/1/NZ1/00001 to G.M.]
Soonju Park	National Research foundation of Korea (NRF) grant funded by the Korea government(MSIT)(NRF-2017M3A9G6068246)
Sophia Q. Xu	NIH NIGMS [grant R35GM140947]
Sourav Banerjee	Cancer Research UK EDDPMA-May21\100005
Stefan Zahler	Dr. Pfleger Stiftung
Stephen J. Capuzzi	The Eshelman Institute for Innovation
Stephen N. Waggoner	NIH [grants DA038017 and AI148080]
Steven H Olson	NIH [grant R01 AG071694-01A1]
Steven R Van Doren	Hirshberg Foundation for Pancreatic Cancer Research and University of Missouri Research Council
Suryakala Sarilla	NIH NHLBI [grant R01 HL130018]

Tatjana Abaffy	NIH/NIDCD [grant DC016224 to Hiroaki Matsunami]
Thilina D Jayasinghe	NIH [grant R01AI105084]
Thomas H Gillingwater	LifeArc/CSO [grant CSO-003]
Thomas Kampourakis	British Heart Foundation [PG/19/52/34497]
Timothy I. Richardson	NIH [grant U54AG065181]
Timothy J. Herdendorf	NIH [grant R35GM140852]
Timothy W. Corson	NIH/NEI [grant R01EY025641]; BrightFocus Foundation
Tobias Hermle	Deutsche Forschungsgemeinschaft (DFG) [HE 7456/3-1, HE 7456/4-1, and project-ID 431984000 – SFB 1453]
Tomisin Happy Ogunwa	Takeda Science Foundation, Japan under the International Fellowship Programs for Foreign Researchers
Tong Su	University of Missouri Spinal Cord Injuries Research Program
Tracy A O'Mara	National Health and Medical Research Council of Australia Investigator Fellowship (APP1173170)
Tsui-Fen Chou	National Institute of Neurological Disorders and Stroke (NINDS) [grant R01NS102279]
Ulrich Baumann	Deutsche Forschungsgemeinschaft (DFG, German Research Foundation)–Project-ID 73111208 - SFB 829
Umesh R Desai	NIH [grants HL107152 and HL128639]

Van Chi Thai	National Health and Medical Research Council of Australia [grant APP1078642]
Versha Banerji	CancerCare Manitoba Foundation, Manitoba Medical Services Foundation, and University of Manitoba
Victoria L. Robinson	Program in Innovative Therapeutics for Connecticut's Health (PITCH); UConn Research Foundation
Vignesh Gunasekharan	Lion Heart Pilot Grant
Vincent FM Segers	Fund for Scientific Research Flanders [ID: 1842219N and G021420N]
Vinícius Gonçalves Maltarollo	FAPEMIG [grant number APQ-01818-21] and the author would like to thank UFMG, its intramural funds, and DRI for their support.
W. Brent Clayton	NIH [grant U54AG065181]
W. Todd Lowther	Oxalosis and Hyperoxaluria Foundation
Walid A. Houry	Canadian Institutes of Health Research [operating grants PJT-173345 and PJT-173491]
Wenjun Zhang	NIH [grant R01GM136758]
Wesley C. Van Voorhis	NIH/NIAID (contract HHSN272200700059C)
William A. Donaldson	NIH [grant GM131356]
William C. Hahn	NIH [grant R03 TR003343]
William G. Kerr	NIH [R01 AG059717]
Xin Qi	NIH [grants R01AG065240, R01NS115903, and R01AG076051]

Xin Xu	NIH [grant R35GM140852]
Xingyou Wang	NIH NIAID [grant AI125362 to G.D. Cuny, University of Houston]
Xu Zhang	China Scholarship Council (CSC) postdoctoral fellowship No. 201808440648
Yasser Ali Aldhamen	Clinical and Translational Science Institute
Yihe Li	Pathfinder, the University of Western Australia; NHMRC, APP1163933
Yogesh K. Gupta	NIH/NIAID [grant 1R01AI161363] and and Welch Foundation (AQ-2101)
Yolanda Pérez-Pertejo	SAF2017-83575-R
Yong Li	Cancer Prevention and Research Institute of Texas [RR190043] and NIH [grants CA219556 and CA229080]
Young Tang	USDA/National Institution for Food and Agriculture (NIFA) [grant 2017–67016-26675], the Program In Innovative Therapeutics for Connecticut’s Health (PITCH) Promising Project Award, and the University of Connecticut Research Excellence Program
Yuan He	NIH [grant R01AI148544]
Yuk-Ching Tse-Dinh	National Institute Of General Medical Sciences of the National Institutes of Health [grant R35GM139817]
Yulia A. Sidorova	Academy of Finland, GA1325555
Yun Yen	TMU Research Center of Cancer Translational Medicine of the Higher Education Sprout Project and the Ministry of Education (MOE) in Taiwan

Zhengchen Su	NIH [grant R01AI121012 to B.G.]
Zhenghe Wang	UH2CA223670; P50CA150964
Zhiguo Zhang	GM118015
Zhongle Liu	Fellowship Grant from PRiME Initiative at University of Toronto

Bibliography

1. Calses, P. C., Crawford, J. J., Lill, J. R. & Dey, A. Hippo Pathway in Cancer: Aberrant Regulation and Therapeutic Opportunities. *Trends Cancer* **5**, 297–307 (2019).
2. Moya, I. M. & Halder, G. Hippo–YAP/TAZ signalling in organ regeneration and regenerative medicine. *Nat. Rev. Mol. Cell Biol.* **20**, 211–226 (2019).
3. Moroishi, T. *et al.* The Hippo Pathway Kinases LATS1/2 Suppress Cancer Immunity. *Cell* **167**, 1525-1539.e17 (2016).
4. Kastan, N. *et al.* Small-molecule inhibition of Lats kinases may promote Yap-dependent proliferation in postmitotic mammalian tissues. *Nat. Commun.* **12**, 3100 (2021).
5. Automated comparative protein structure modeling with SWISS‐MODEL and Swiss‐PdbViewer: A historical perspective - Guex - 2009 - ELECTROPHORESIS - Wiley Online Library. https://analyticalsciencejournals.onlinelibrary.wiley.com/doi/10.1002/elps.200900140.
6. SWISS-MODEL Repository—new features and functionality | Nucleic Acids Research | Oxford Academic. https://academic.oup.com/nar/article/45/D1/D313/2605750.
7. van den Boom, J. & Meyer, H. VCP/p97-Mediated Unfolding as a Principle in Protein Homeostasis and Signaling. *Mol. Cell* **69**,

182–194 (2018).

8. Nalbandian, A. *et al.* A progressive translational mouse model of human valosin-containing protein disease: The VCPR155H/+ mouse. *Muscle Nerve* **47**, 260–270 (2013).

9. Yin, H. Z. *et al.* Slow development of ALS-like spinal cord pathology in mutant valosin-containing protein gene knock-in mice. *Cell Death Dis.* **3**, e374–e374 (2012).

10. VCP/p97 is essential for maturation of ubiquitin-containing autophagosomes and this function is impaired by mutations that cause IBMPFD: Autophagy: Vol 6, No 2. https://www.tandfonline.com/doi/abs/10.4161/auto.6.2.11014.

11. Critical Role of VCP/p97 in the Pathogenesis and Progression of Non-Small Cell Lung Carcinoma | PLOS ONE. https://journals.plos.org/plosone/article?id=10.1371/journal.pone.0029073.

12. Li, C. *et al.* p97/VCP is highly expressed in the stem-like cells of breast cancer and controls cancer stemness partly through the unfolded protein response. *Cell Death Dis.* **12**, 1–16 (2021).

13. Magnaghi, P. *et al.* Covalent and allosteric inhibitors of the ATPase VCP/p97 induce cancer cell death. *Nat. Chem. Biol.* **9**, 548–556 (2013).

14. Nishimura, N. *et al.* Novel p97/VCP inhibitor induces endoplasmic reticulum stress and apoptosis in both bortezomib-sensitive and -resistant multiple myeloma cells. *Cancer Sci.* **110**, 3275–3287 (2019).

15. Chou, T.-F. *et al.* Reversible inhibitor of p97, DBeQ, impairs both ubiquitin-dependent and autophagic protein clearance pathways. *Proc. Natl. Acad. Sci.* **108**, 4834–4839 (2011).

16. Banerjee, S. *et al.* 2.3 Å resolution cryo-EM structure of human p97 and mechanism of allosteric inhibition. *Science* **351**, 871–875

(2016).

17. Anderson, D. J. *et al.* Targeting the AAA ATPase p97 as an Approach to Treat Cancer through Disruption of Protein Homeostasis. *Cancer Cell* **28**, 653–665 (2015).
18. ADP-Glo: A Bioluminescent and Homogeneous ADP Monitoring Assay for Kinases | ASSAY and Drug Development Technologies. https://www.liebertpub.com/doi/10.1089/adt.2009.0222.
19. Bickerton, G. R., Paolini, G. V., Besnard, J., Muresan, S. & Hopkins, A. L. Quantifying the chemical beauty of drugs. *Nat. Chem.* **4**, 90–98 (2012).
20. *RDKit: Open-source cheminformatics*.
21. Hsieh, C.-H. *et al.* Miro1 Marks Parkinson's Disease Subset and Miro1 Reducer Rescues Neuron Loss in Parkinson's Models. *Cell Metab.* **30**, 1131-1140.e7 (2019).
22. Huang, C. *et al.* Small molecules block the interaction between porcine reproductive and respiratory syndrome virus and CD163 receptor and the infection of pig cells. *Virol. J.* **17**, 116 (2020).
23. Bon, C. *et al.* Discovery of Novel Trace Amine-Associated Receptor 5 (TAAR5) Antagonists Using a Deep Convolutional Neural Network. *Int. J. Mol. Sci.* **23**, 3127 (2022).
24. Pedicone, C. *et al.* Discovery of a novel SHIP1 agonist that promotes degradation of lipid-laden phagocytic cargo by microglia. *iScience* **25**, 104170 (2022).
25. Su, S. *et al.* SPOP and OTUD7A Control EWS–FLI1 Protein Stability to Govern Ewing Sarcoma Growth. *Adv. Sci.* **8**, 2004846 (2021).

26. Stecula, A., Hussain, M. S. & Viola, R. E. Discovery of Novel Inhibitors of a Critical Brain Enzyme Using a Homology Model and a Deep Convolutional Neural Network. *J. Med. Chem.* **63**, 8867–8875 (2020).
