# Supplementary material for: AI is a viable alternative to high throughput screening: a 318-target study
Source: Sci Rep. 2024 Apr 2;14:7526. doi: 10.1038/s41598-024-54655-z (PMC10987645; doi:10.1038/s41598-024-54655-z)
Supplement: Supplementary file 1 — Supplementary Information 1. [file 41598_2024_54655_MOESM1_ESM.zip › Nature SREP/QC_AIMS_files/Proj007.doc]

```
Data File D:\DATA\891\2FG-5401.D
Sample Name: HA050667P2-F-07
Instrument 1 22.11.2010 8:01:00  #6
Column: SpeedROD Rp-18e 50x4.6mm | 3.75ml/min | Columns Reg Valve
Gradient: "A"->@2.2min->"B"(Hold 0.4min)->@0.2min->"A"->PostRun
PMP1, Solvent A         : 0.1%TFA in Acn/H2O (2.5:97.5)
PMP1, Solvent B         : 0.1% TFA in AcN
PMP1, Solvent C         : 0.1%FA in ACN/H2O (2.5:97.5)
PMP1, Solvent D         : 0.1%FA in ACN
Ionization mode             :    APCI Positive

Signal 1: ADC1 A, ELSD
Peak RetTime Type  Width     Area      Height     Area
  #   [min]        [min]   [mV*s]      [mV]         %
----|-------|----|-------|----------|----------|--------|
   1   1.790 PB    0.0300 1455.49133  753.80945 100.0000
Totals :                  1455.49133  753.80945


Signal 2: DAD1 A, Sig=300,200 Ref=off
Peak RetTime Type  Width     Area      Height     Area
  #   [min]        [min]   [mAU*s]     [mAU]        %
----|-------|----|-------|----------|----------|--------|
   1   1.739 PB    0.0322  641.29364  321.92618 100.0000
Totals :                   641.29364  321.92618


Signal 3: MSD1 TIC, MS File
Peak RetTime Type  Width     Area      Height     Area
  #   [min]        [min]                            %
----|-------|----|-------|----------|----------|--------|
   1   1.764 PB    0.0317 2.56970e6  1.22981e6  100.0000
Totals :                  2.56970e6  1.22981e6
```
