# Supplementary material for: AI is a viable alternative to high throughput screening: a 318-target study
Source: Sci Rep. 2024 Apr 2;14:7526. doi: 10.1038/s41598-024-54655-z (PMC10987645; doi:10.1038/s41598-024-54655-z)
Supplement: Supplementary file 1 — Supplementary Information 1. [file 41598_2024_54655_MOESM1_ESM.zip › Nature SREP/QC_AIMS_files/Proj019.doc]

```
Data File R:\HPLC\AUTO\FC947956\2BD-3001.D
Sample Name: FC947956P2-B-04
Instrument 1 27/04/2012 11:53:57
PMP1, Solvent A         : 0.1%TFA in Acn/H2O (2.5:97.5)
PMP1, Solvent B         : 0.1% TFA in AcN
PMP1, Solvent C         : 0.1%FA in ACN/H2O (2.5:97.5)
PMP1, Solvent D         : 0.1%FA in ACN
Ionization mode             :    APCI Positive

Signal 1: ADC1 A, ELSD
Peak RetTime Type  Width     Area      Height     Area
  #   [min]        [min]   [mV*s]      [mV]         %
----|-------|----|-------|----------|----------|--------|
   1   1.537 MM    0.0334  432.93094  216.20387 100.0000
Totals :                   432.93094  216.20387


Signal 2: DAD1 A, Sig=300,200 Ref=off
Peak RetTime Type  Width     Area      Height     Area
  #   [min]        [min]   [mAU*s]     [mAU]        %
----|-------|----|-------|----------|----------|--------|
   1   1.483 MM    0.0280 2113.02905 1257.14343 100.0000
Totals :                  2113.02905 1257.14343


Signal 3: MSD1 TIC, MS File
Peak RetTime Type  Width     Area      Height     Area
  #   [min]        [min]                            %
----|-------|----|-------|----------|----------|--------|
   1   1.551 MM    0.0545 9.50154e6  2.90438e6  100.0000
Totals :                  9.50154e6  2.90438e6
```
