# Supplementary material for: AI is a viable alternative to high throughput screening: a 318-target study
Source: Sci Rep. 2024 Apr 2;14:7526. doi: 10.1038/s41598-024-54655-z (PMC10987645; doi:10.1038/s41598-024-54655-z)
Supplement: Supplementary file 1 — Supplementary Information 1. [file 41598_2024_54655_MOESM1_ESM.zip › Nature SREP/QC_AIMS_files/Proj060.DOC]

```
Data File R:\HPLC\AUTO\FORPIL~1\HURRY\ST5270\1GA-0801.D
Sample Name: ST5270P1-G-01
Instrument 1 05/03/2019 18:30:36
Column: Onyx C18 50x4.6mm | 3.75ml/min | Columns Reg Valve
Gradient: "A"->@2.0min->"B"(Hold 0.6min)->@0.2min->"A"->PostRun
PMP1, Solvent A         : 0.1%TFA, 2.5%AcN in H2O
PMP1, Solvent B         : 0.1%TFA in AcN
PMP1, Solvent C         : --NOT USED--
PMP1, Solvent D         : --NOT USED--
Ionization mode             :    API-ES Positive

Signal 1: ADC1 B, ELSD
Peak RetTime Type  Width     Area      Height     Area
  #   [min]        [min]   [mV*s]      [mV]         %
----|-------|----|-------|----------|----------|--------|
   1   1.073 BB    0.0369  652.84778  263.20938  99.3788
   2   1.332 BB    0.0380    4.08084    1.53346   0.6212
Totals :                   656.92862  264.74284


Signal 2: DAD1 A, Sig=300,200 Ref=off
Peak RetTime Type  Width     Area      Height     Area
  #   [min]        [min]   [mAU*s]     [mAU]        %
----|-------|----|-------|----------|----------|--------|
   1   0.996 BV    0.0275 2906.71899 1735.58752  88.8635
   2   1.086 MM    0.0407   90.15130   36.91222   2.7561
   3   1.166 MM    0.0484   72.15075   24.84426   2.2058
   4   1.253 BB    0.0256  141.85014   84.58226   4.3366
   5   1.434 MM    0.0460   60.12140   21.79029   1.8380
Totals :                  3270.99258 1903.71655


Signal 3: MSD1 TIC, MS File
Peak RetTime Type  Width     Area      Height     Area
  #   [min]        [min]                            %
----|-------|----|-------|----------|----------|--------|
   1   1.024 MM    0.0482 1.29944e6  4.49110e5  100.0000
Totals :                  1.29944e6  4.49110e5
```
