# Supplementary material for: AI is a viable alternative to high throughput screening: a 318-target study
Source: Sci Rep. 2024 Apr 2;14:7526. doi: 10.1038/s41598-024-54655-z (PMC10987645; doi:10.1038/s41598-024-54655-z)
Supplement: Supplementary file 1 — Supplementary Information 1. [file 41598_2024_54655_MOESM1_ESM.zip › Nature SREP/QC_AIMS_files/Proj098.doc]

```
Data File R:\HPLC\AUTO\ST6958\1EC-4401.D
Sample Name: ST6958P1-E-03
Instrument 1 22/04/2020 12:48:00
Column: Onyx C18 50x4.6mm | 3.75ml/min | Columns Reg Valve
Gradient: "A"->@2.0min->"B"(Hold 0.6min)->@0.2min->"A"->PostRun
PMP1, Solvent A         : 0.1%TFA, 2.5%AcN in H2O
PMP1, Solvent B         : 0.1%TFA in AcN
PMP1, Solvent C         : --NOT USED--
PMP1, Solvent D         : --NOT USED--
Ionization mode             :    API-ES Positive

Signal 1: ADC1 B, ELSD
Peak RetTime Type  Width     Area      Height     Area
  #   [min]        [min]   [mV*s]      [mV]         %
----|-------|----|-------|----------|----------|--------|
   1   1.690 BB    0.0248  138.09586   85.85629 100.0000
Totals :                   138.09586   85.85629


Signal 2: DAD1 A, Sig=300,200 Ref=off
Peak RetTime Type  Width     Area      Height     Area
  #   [min]        [min]   [mAU*s]     [mAU]        %
----|-------|----|-------|----------|----------|--------|
   1   1.205 MM    0.0209   19.97787   15.90067   2.5873
   2   1.644 MM    0.0330  752.18628  379.66046  97.4127
Totals :                   772.16415  395.56113


Signal 3: MSD1 TIC, MS File
Peak RetTime Type  Width     Area      Height     Area
  #   [min]        [min]                            %
----|-------|----|-------|----------|----------|--------|
   1   1.707 MM    0.0576 5.94628e6  1.72184e6  100.0000
Totals :                  5.94628e6  1.72184e6
```
