# Supplementary material for: AI is a viable alternative to high throughput screening: a 318-target study
Source: Sci Rep. 2024 Apr 2;14:7526. doi: 10.1038/s41598-024-54655-z (PMC10987645; doi:10.1038/s41598-024-54655-z)
Supplement: Supplementary file 1 — Supplementary Information 1. [file 41598_2024_54655_MOESM1_ESM.zip › Nature SREP/QC_AIMS_files/Proj174.DOC]

```
Data File R:\HPLC\AUTO\1R\ST8589\1BB-1001.D
Sample Name: ST8589P1-B-02
Instrument 1 11/01/2021 10:52:00  1
Column: Onyx C18 50x4.6mm | 3.75ml/min | Columns Reg Valve
Gradient: "A"->@2.0min->"B"(Hold 0.4min)->@0.2min->"A"->PostRun
PMP1, Solvent A         : 0.1TFA in 2.5%AcN/W
PMP1, Solvent B         : 0.1TFA in AcN
PMP1, Solvent C         : 0.1FA in 2.5%AcN/W
PMP1, Solvent D         : 0.1FA in AcN
Ionization mode             :    API-ES Positive

Signal 1: ADC1 A, ADC1 ELSD
Peak RetTime Type  Width     Area      Height     Area
  #   [min]        [min]   [mAU*s]     [mAU]        %
----|-------|----|-------|----------|----------|--------|
   1   1.027 MM    0.0305  179.94725   98.31998 100.0000
Totals :                   179.94725   98.31998


Signal 2: DAD1 A, Sig=300,200 Ref=off
Peak RetTime Type  Width     Area      Height     Area
  #   [min]        [min]   [mAU*s]     [mAU]        %
----|-------|----|-------|----------|----------|--------|
   1   0.981 MM    0.0343 1317.99121  640.86871 100.0000
Totals :                  1317.99121  640.86871


Signal 3: MSD1 TIC, MS File
Peak RetTime Type  Width     Area      Height     Area
  #   [min]        [min]                            %
----|-------|----|-------|----------|----------|--------|
   1   0.990 MM    0.0510 3.38460e6  1.10663e6  100.0000
Totals :                  3.38460e6  1.10663e6
```
