# Supplementary material for: AI is a viable alternative to high throughput screening: a 318-target study
Source: Sci Rep. 2024 Apr 2;14:7526. doi: 10.1038/s41598-024-54655-z (PMC10987645; doi:10.1038/s41598-024-54655-z)
Supplement: Supplementary file 1 — Supplementary Information 1. [file 41598_2024_54655_MOESM1_ESM.zip › Nature SREP/QC_AIMS_files/Proj216.doc]

```
Data File R:\HPLC\AUTO\ST1306\2BA-5001.D
Sample Name: ST1306P2-B-01
Instrument 1 22/10/2015 16:33:40
Column: Onyx C18 50x4.6mm | 3.75ml/min | Columns Reg Valve
Gradient: "A"->@2.2min->"B"(Hold 0.4min)->@0.2min->"A"->PostRun
PMP1, Solvent A         : 0.1TFA in AcN
PMP1, Solvent B         : 00.1TFA in 2.5%AcN/W
PMP1, Solvent C         :
PMP1, Solvent D         :
Ionization mode             :    API-ES Positive

Signal 1: ADC1 A, ADC1 ELSD
Peak RetTime Type  Width     Area      Height     Area
  #   [min]        [min]   [mAU*s]     [mAU]        %
----|-------|----|-------|----------|----------|--------|
   1   1.037 BB    0.0294   15.85568    8.97475 100.0000
Totals :                    15.85568    8.97475


Signal 2: DAD1 A, Sig=300,200 Ref=off
Peak RetTime Type  Width     Area      Height     Area
  #   [min]        [min]   [mAU*s]     [mAU]        %
----|-------|----|-------|----------|----------|--------|
   1   0.976 BB    0.0278  147.11844   86.80284 100.0000
Totals :                   147.11844   86.80284


Signal 3: MSD1 TIC, MS File
Peak RetTime Type  Width     Area      Height     Area
  #   [min]        [min]                            %
----|-------|----|-------|----------|----------|--------|
   1   1.016 BP    0.0487 1.22096e6  4.16276e5  100.0000
Totals :                  1.22096e6  4.16276e5
```
