# Supplementary material for: AI is a viable alternative to high throughput screening: a 318-target study
Source: Sci Rep. 2024 Apr 2;14:7526. doi: 10.1038/s41598-024-54655-z (PMC10987645; doi:10.1038/s41598-024-54655-z)
Supplement: Supplementary file 1 — Supplementary Information 1. [file 41598_2024_54655_MOESM1_ESM.zip › Nature SREP/QC_AIMS_files/Proj217.DOC]

```
Data File R:\HPLC\AUTO\FORPIL~1\FC\ST6139\1EA-8201.D
Sample Name: ST6139P1-E-01
Instrument 1 26/09/2019 14:21:17
Column: Onyx C18 50x4.6mm | 3.75ml/min | Columns Reg Valve
Gradient: "A"->@2.0min->"B"(Hold 0.6min)->@0.2min->"A"->PostRun
PMP1, Solvent A         : 0.1%TFA, 2.5%AcN in H2O
PMP1, Solvent B         : 0.1%TFA in AcN
PMP1, Solvent C         : --NOT USED--
PMP1, Solvent D         : --NOT USED--
Ionization mode             :    API-ES Positive

Signal 1: ADC1 B, ELSD
Peak RetTime Type  Width     Area      Height     Area
  #   [min]        [min]   [mV*s]      [mV]         %
----|-------|----|-------|----------|----------|--------|
   1   1.326 BB    0.0327   49.92316   22.67562 100.0000
Totals :                    49.92316   22.67562


Signal 2: DAD1 A, Sig=300,200 Ref=off
Peak RetTime Type  Width     Area      Height     Area
  #   [min]        [min]   [mAU*s]     [mAU]        %
----|-------|----|-------|----------|----------|--------|
   1   0.551 MM    0.0418   14.85479    5.92832   2.3891
   2   0.971 MM    0.0340    8.03594    3.93568   1.2924
   3   1.046 MM    0.0487   16.55864    5.66350   2.6631
   4   1.115 MM    0.0339   10.19667    5.01595   1.6399
   5   1.260 BB    0.0303  572.12500  299.10663  92.0154
Totals :                   621.77103  319.65008


Signal 3: MSD1 TIC, MS File
Peak RetTime Type  Width     Area      Height     Area
  #   [min]        [min]                            %
----|-------|----|-------|----------|----------|--------|
   1   1.291 MM    0.0506 5.01257e6  1.65000e6  100.0000
Totals :                  5.01257e6  1.65000e6
```
