# Supplementary material for: AI is a viable alternative to high throughput screening: a 318-target study
Source: Sci Rep. 2024 Apr 2;14:7526. doi: 10.1038/s41598-024-54655-z (PMC10987645; doi:10.1038/s41598-024-54655-z)
Supplement: Supplementary file 1 — Supplementary Information 1. [file 41598_2024_54655_MOESM1_ESM.zip › Nature SREP/QC_AIMS_files/Proj237.doc]

```
Data File R:\HPLC\AUTO\!IVANOVA\ST10514\1DB-0601.D
Sample Name: ST10514P1-D-02
Instrument 1 27/12/2021 11:53:03 PM
Column: Onyx C18 50x4.6mm | 3.75ml/min | Columns Reg Valve
Gradient: "A"->@2.0min->"B"(Hold 0.6min)->@0.2min->"A"->PostRun
PMP1, Solvent A         : 0.1TFA in 2.5%AcN/W
PMP1, Solvent B         : 0.1TFA in AcN
PMP1, Solvent C         : 0.1FA in 2.5%AcN/W
PMP1, Solvent D         : 0.1FA in AcN
Ionization mode             :    API-ES Positive

Signal 1: ADC1 A, ADC1 ELSD
Peak RetTime Type  Width     Area      Height     Area
  #   [min]        [min]   [mAU*s]     [mAU]        %
----|-------|----|-------|----------|----------|--------|
   1   1.219 PB    0.0358 1070.58838  449.21436 100.0000
Totals :                  1070.58838  449.21436


Signal 2: DAD1 A, Sig=300,200 Ref=off
Peak RetTime Type  Width     Area      Height     Area
  #   [min]        [min]   [mAU*s]     [mAU]        %
----|-------|----|-------|----------|----------|--------|
   1   1.076 PV    0.0303   47.49384   22.79737   2.0457
   2   1.162 VB    0.0360 2274.11523 1020.13507  97.9543
Totals :                  2321.60907 1042.93244


Signal 3: MSD1 TIC, MS File
Peak RetTime Type  Width     Area      Height     Area
  #   [min]        [min]                            %
----|-------|----|-------|----------|----------|--------|
   1   1.196 MM    0.0575 5.20271e6  1.50676e6  100.0000
Totals :                  5.20271e6  1.50676e6
```
