# Supplementary material for: AI is a viable alternative to high throughput screening: a 318-target study
Source: Sci Rep. 2024 Apr 2;14:7526. doi: 10.1038/s41598-024-54655-z (PMC10987645; doi:10.1038/s41598-024-54655-z)
Supplement: Supplementary file 1 — Supplementary Information 1. [file 41598_2024_54655_MOESM1_ESM.zip › Nature SREP/QC_AIMS_files/Proj242.pdf]

MaxPeak: 97.23%
Ret_Time: 0.729 min

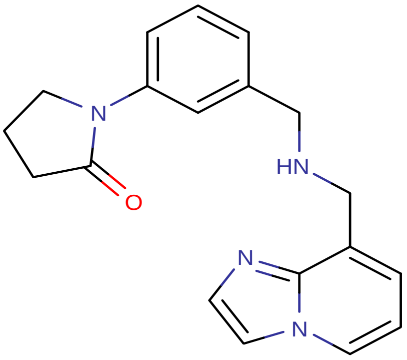

Mol Wt 320.39

Exact Mass 320.19

#	Time	Area%
1	0.729	97.23
2	1.171	2.77

L152178$2

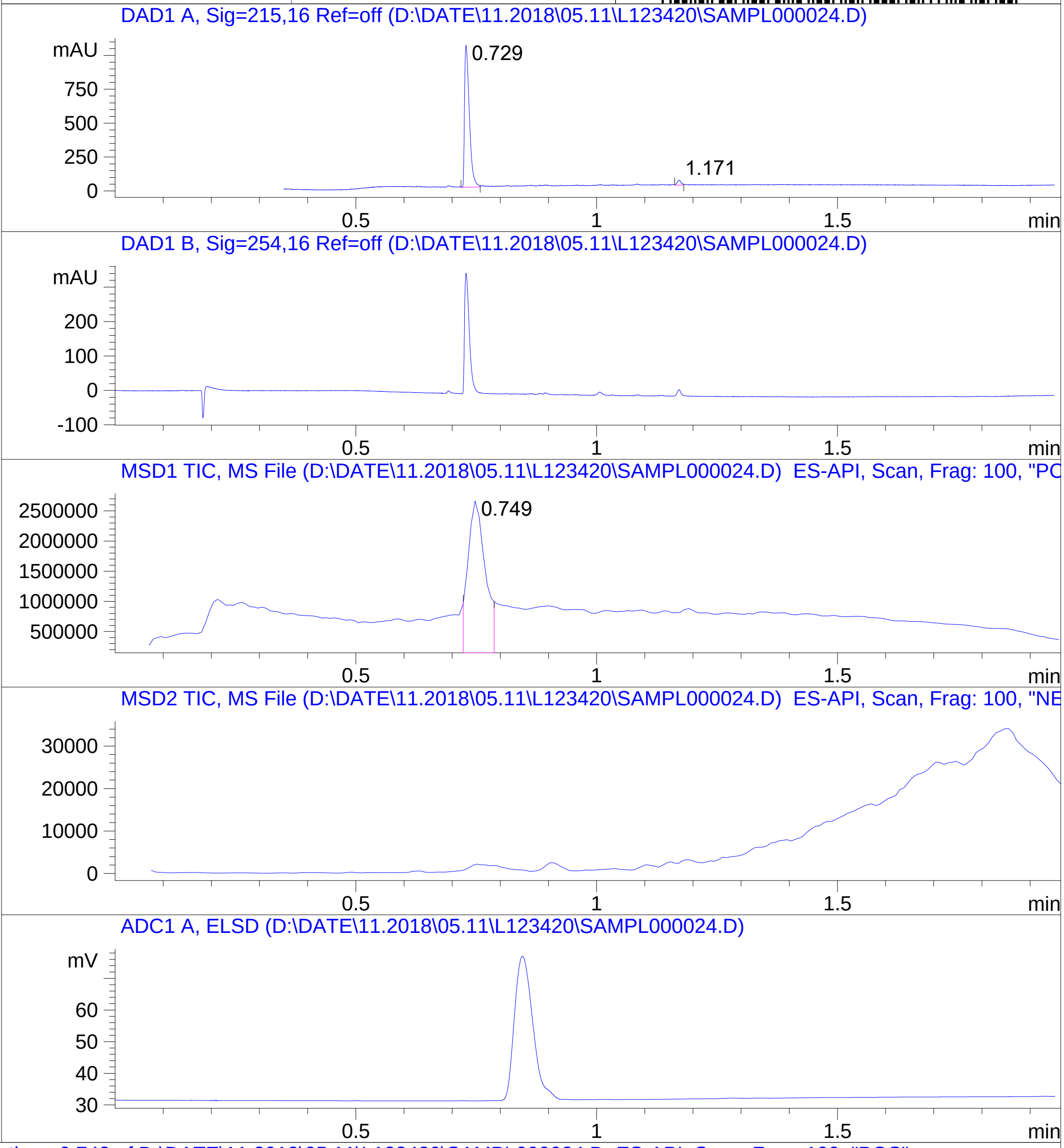

RT 0.749

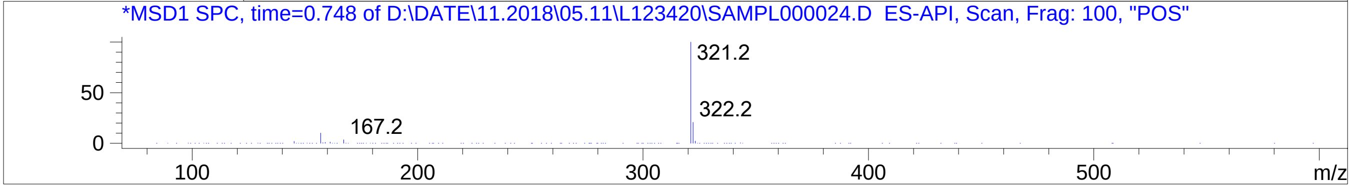

Inj.Date 11/2/2018 AK P2-C-06 -3- Acq. Method C:\CHEM32\-> ->
