# Supplementary material for: AI is a viable alternative to high throughput screening: a 318-target study
Source: Sci Rep. 2024 Apr 2;14:7526. doi: 10.1038/s41598-024-54655-z (PMC10987645; doi:10.1038/s41598-024-54655-z)

MaxPeak: 100.00%
Ret_Time: 1.111 min

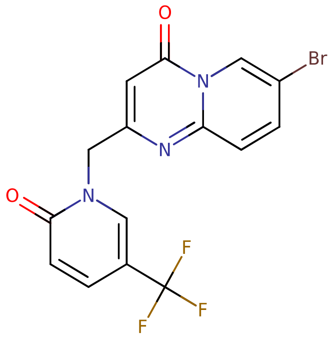


**Mol Wt** 400.15

**Exact Mass** 398.99

| # | Time | Area% |
| --- | --- | --- |
| 1 | 1.111 | 100.00 |

# T6409153

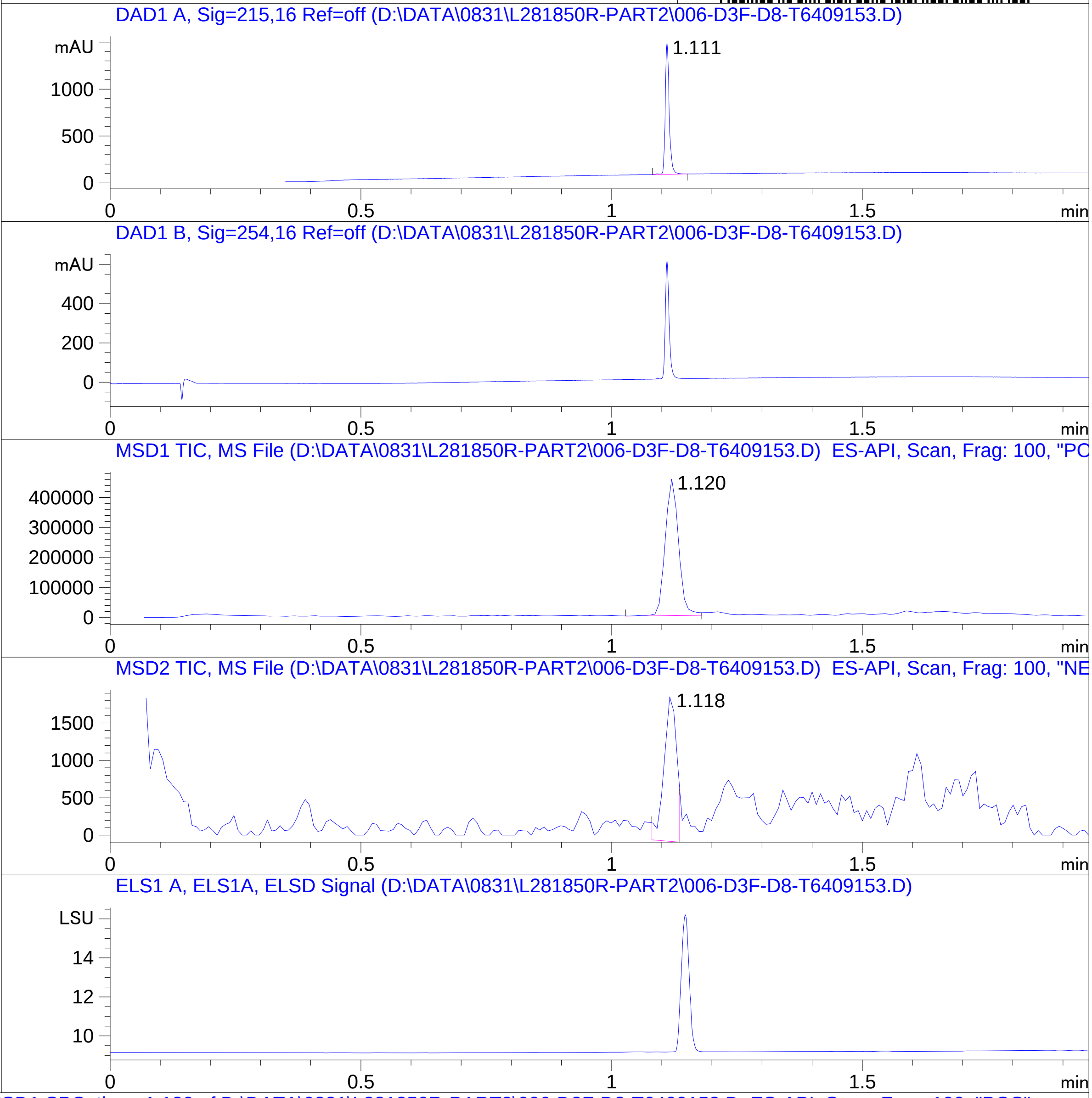


RT 1.120

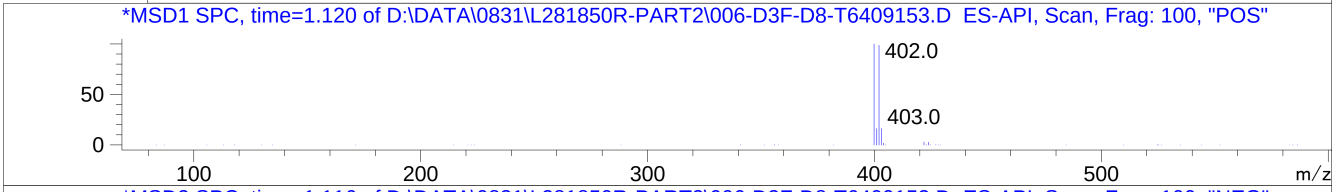


RT 1.118

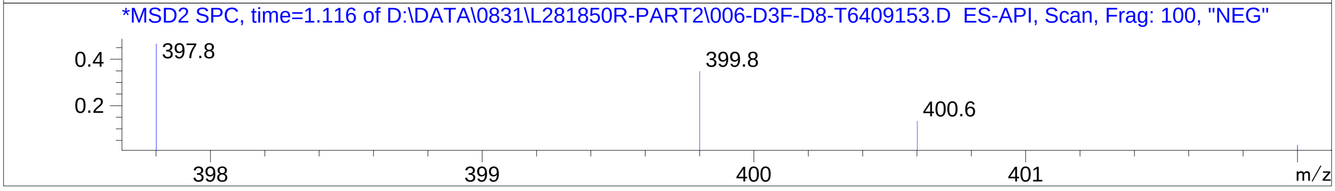

Supplement: Supplementary file 1 — Supplementary Information 1. [file 41598_2024_54655_MOESM1_ESM.zip › Nature SREP/QC_AIMS_files/Proj245.pdf]
