# Supplementary material for: AI is a viable alternative to high throughput screening: a 318-target study
Source: Sci Rep. 2024 Apr 2;14:7526. doi: 10.1038/s41598-024-54655-z (PMC10987645; doi:10.1038/s41598-024-54655-z)
Supplement: Supplementary file 1 — Supplementary Information 1. [file 41598_2024_54655_MOESM1_ESM.zip › Nature SREP/QC_AIMS_files/Proj247.pdf]

T6941678

MaxPeak: 96.73%
Ret_Time: 0.832 min

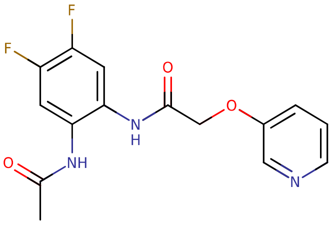

Mol Wt 321.28

Exact Mass 321.1

#	Time	Area%
1	0.832	96.73
2	0.879	3.27

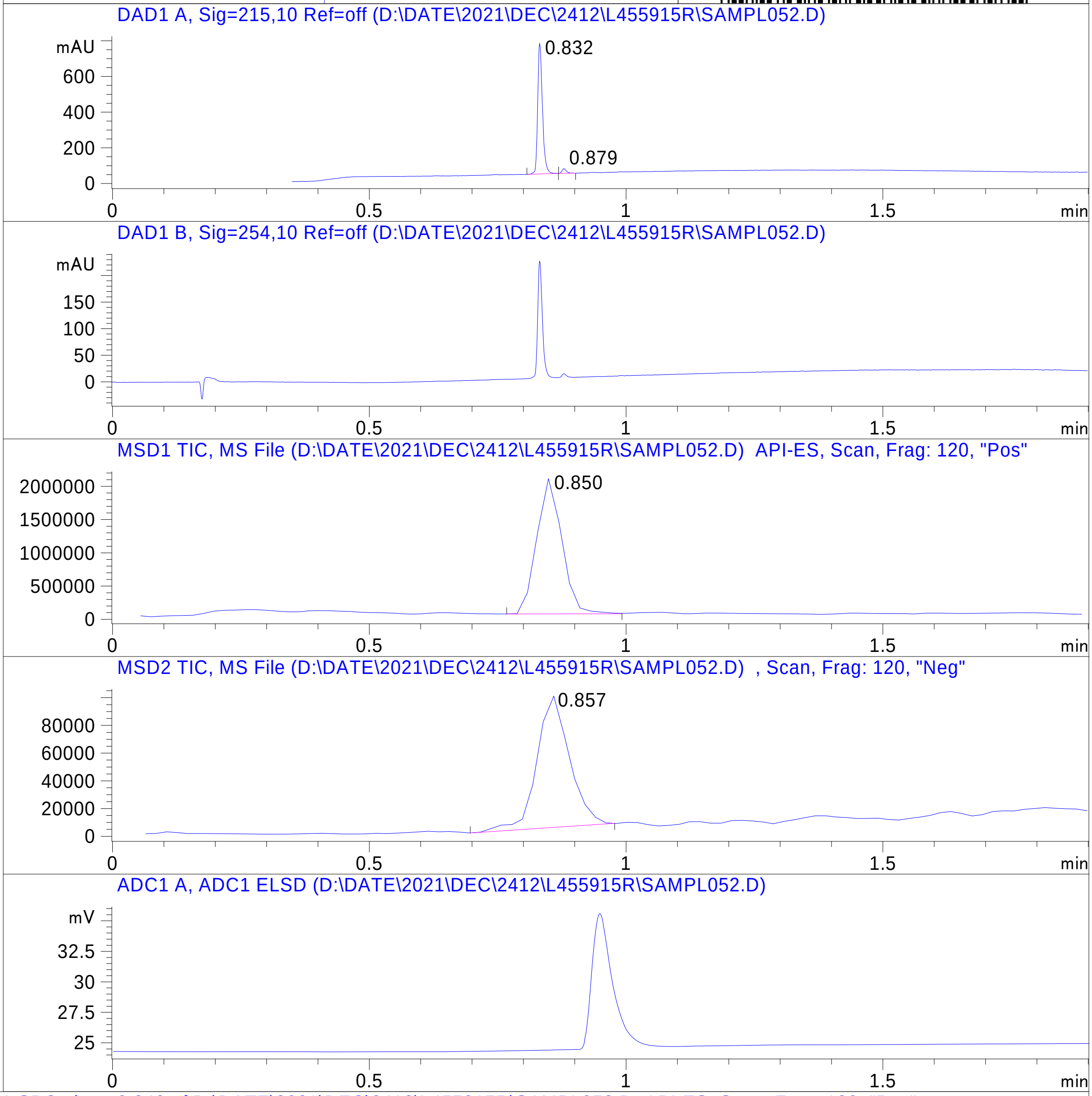

RT 0.850

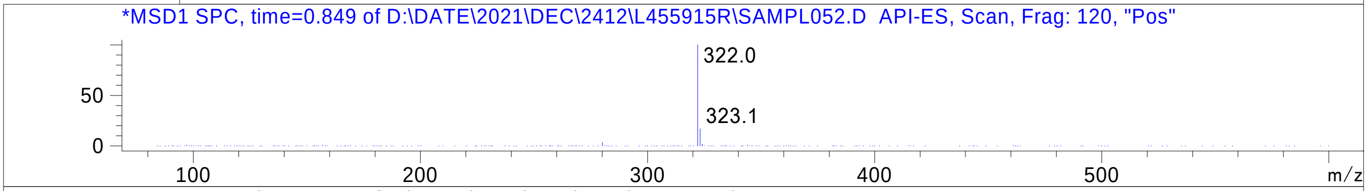

RT 0.857

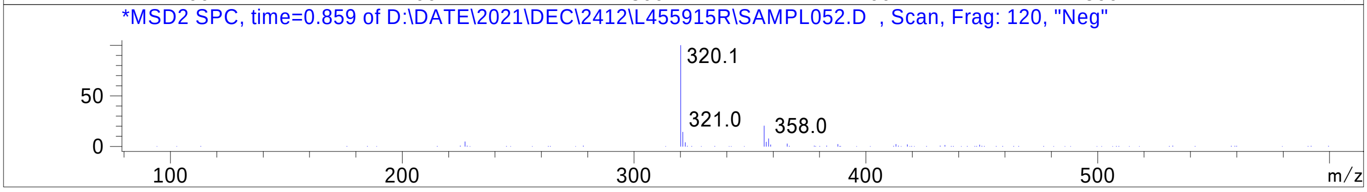

Inj.Date 24-Dec-21 Z P2-F-08 -VL- Acq. Method C:\HPCHEM\-> ->
