# Supplementary material for: AI is a viable alternative to high throughput screening: a 318-target study
Source: Sci Rep. 2024 Apr 2;14:7526. doi: 10.1038/s41598-024-54655-z (PMC10987645; doi:10.1038/s41598-024-54655-z)
Supplement: Supplementary file 1 — Supplementary Information 1. [file 41598_2024_54655_MOESM1_ESM.zip › Nature SREP/QC_AIMS_files/Proj248.pdf]

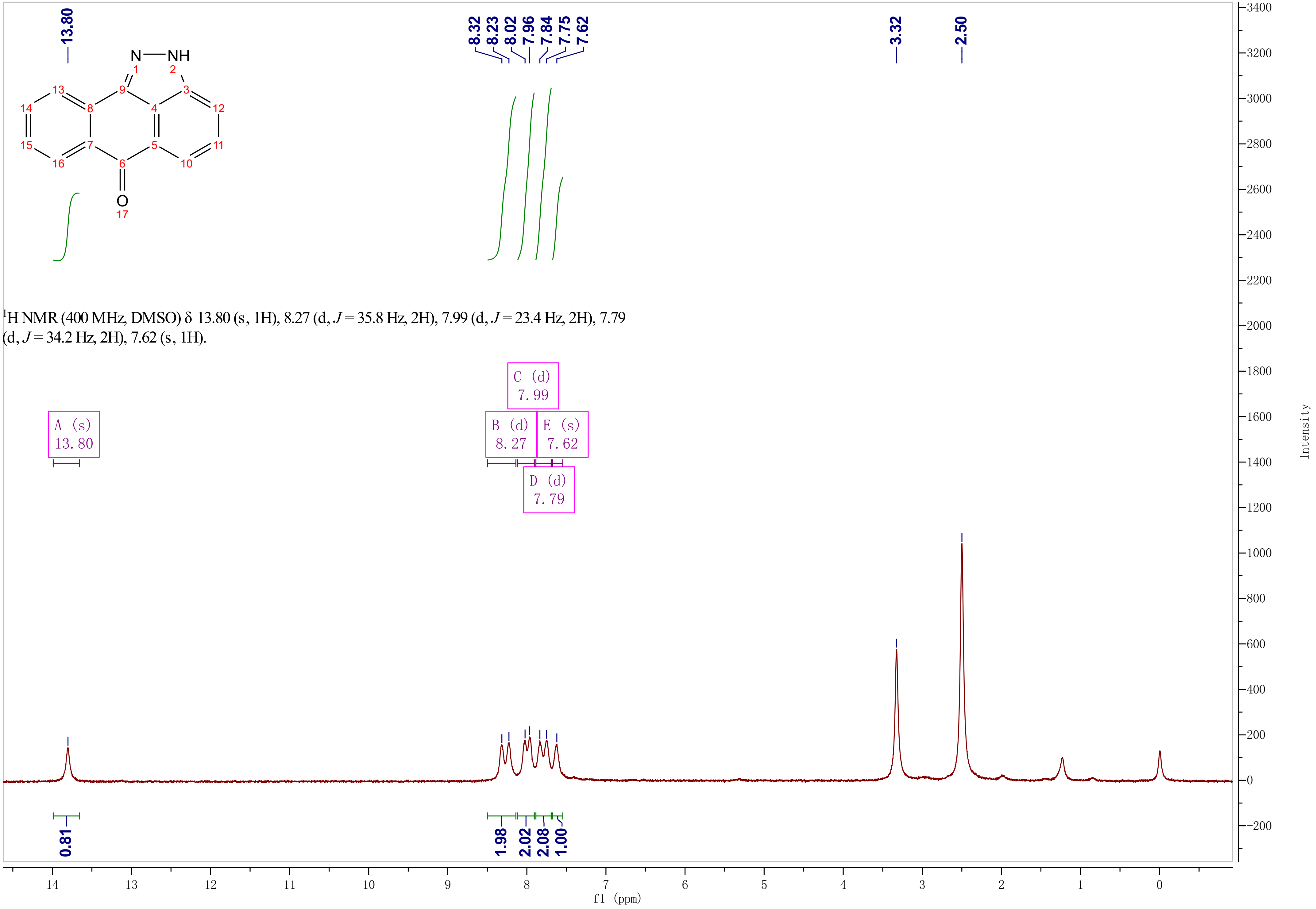
13.80
8.32
8.23
8.02
7.96
7.84
7.75
7.62
3.32
2.50
N
NH
1
2
3
4
5
6
7
8
9
10
11
12
13
14
15
16
O
17
1H NMR (400 MHz, DMSO) δ 13.80 (s, 1H), 8.27 (d, J = 35.8 Hz, 2H), 7.99 (d, J = 23.4 Hz, 2H), 7.79 (d, J = 34.2 Hz, 2H), 7.62 (s, 1H).
A (s) 13.80
B (d) 8.27
C (d) 7.99
D (d) 7.79
E (s) 7.62
0.81
1.98
2.02
2.08
1.00
f1 (ppm)
Intensity
