# Supplementary material for: AI is a viable alternative to high throughput screening: a 318-target study
Source: Sci Rep. 2024 Apr 2;14:7526. doi: 10.1038/s41598-024-54655-z (PMC10987645; doi:10.1038/s41598-024-54655-z)
Supplement: Supplementary file 1 — Supplementary Information 1. [file 41598_2024_54655_MOESM1_ESM.zip › Nature SREP/QC_AIMS_files/Proj249.pdf]

MaxPeak: 100.00%
Ret_Time: 1.135 min

P723885$3

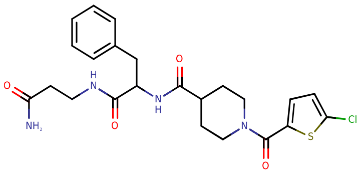

Mol Wt 491

Exact Mass 490.17

```
#  Time    Area%
-----------------
1  1.135  100.00
```

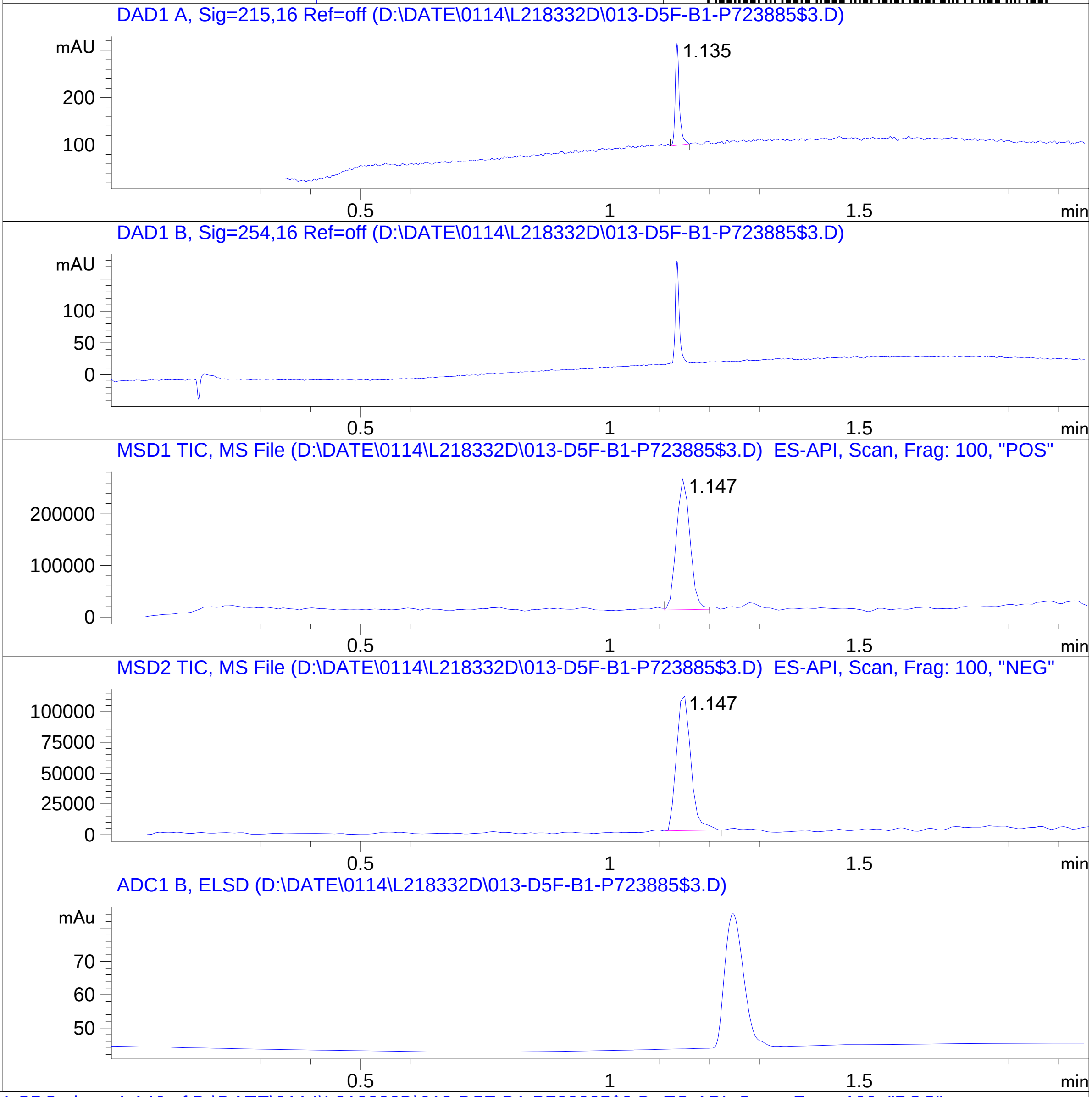

RT 1.147

RT 1.147

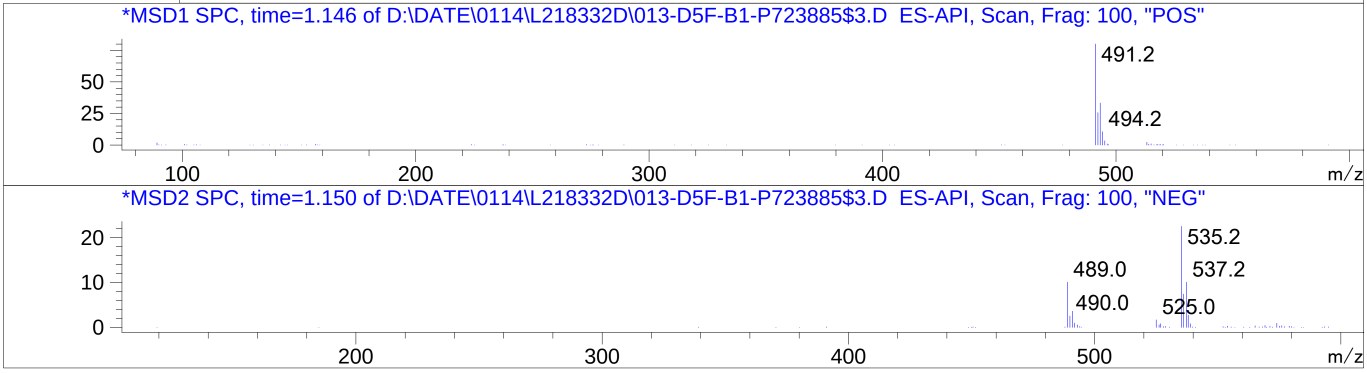

Inj.Date 1/14/2020 E <invalid> -10- Acq. Method C:\Chem32\-> ->
