# Supplementary material for: AI is a viable alternative to high throughput screening: a 318-target study
Source: Sci Rep. 2024 Apr 2;14:7526. doi: 10.1038/s41598-024-54655-z (PMC10987645; doi:10.1038/s41598-024-54655-z)
Supplement: Supplementary file 1 — Supplementary Information 1. [file 41598_2024_54655_MOESM1_ESM.zip › Nature SREP/QC_AIMS_files/Proj250.pdf]

MaxPeak: 91.30%
Ret_Time: 1.645 min

8825207$2

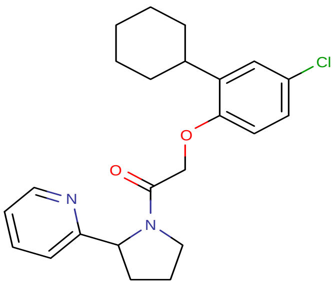

Mol Wt 398.93

Exact Mass 398.22

#	Time	Area%
1	1.037	2.91
2	1.610	5.79
3	1.645	91.30

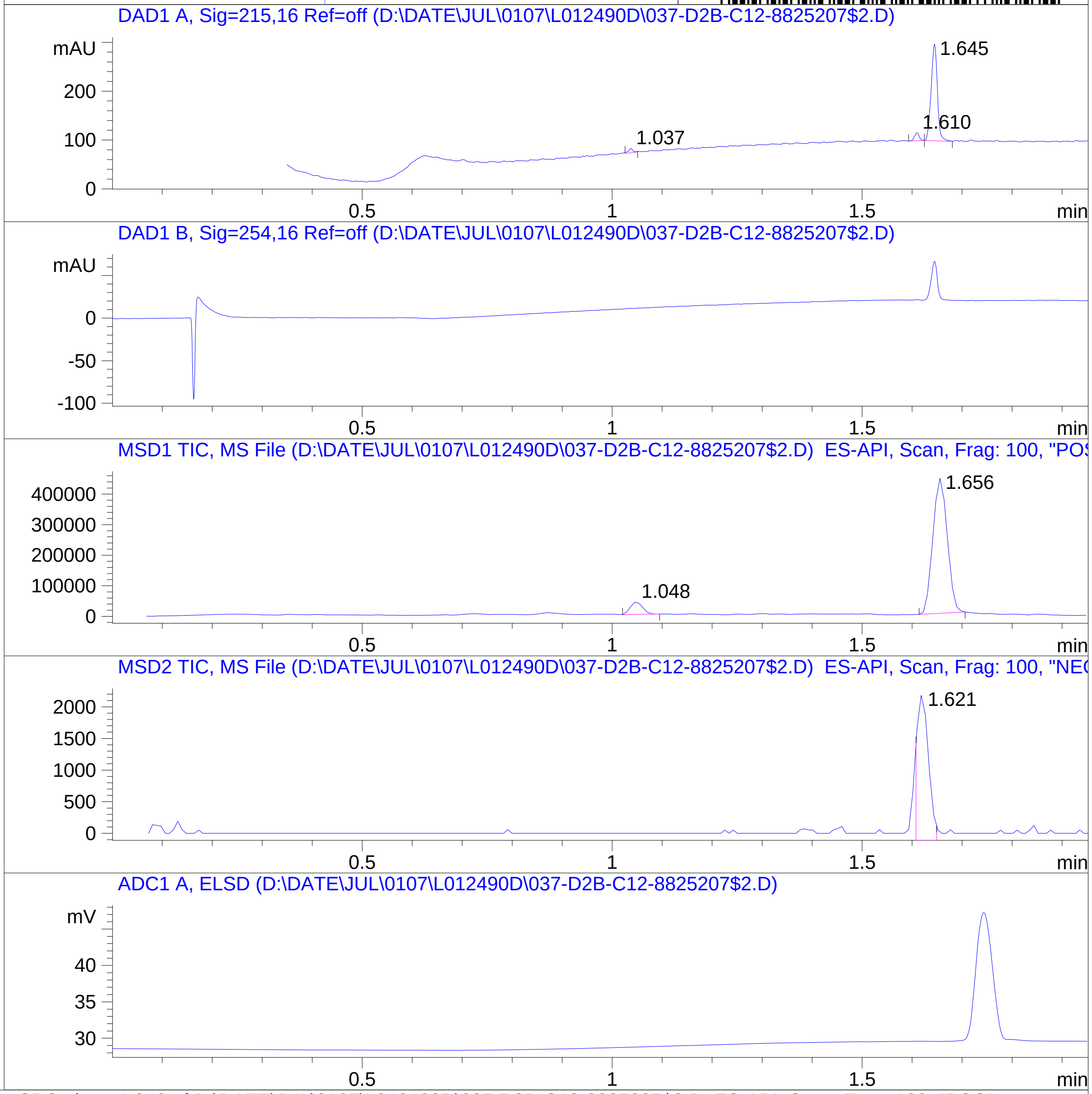

RT 1.048

RT 1.656

RT 1.621

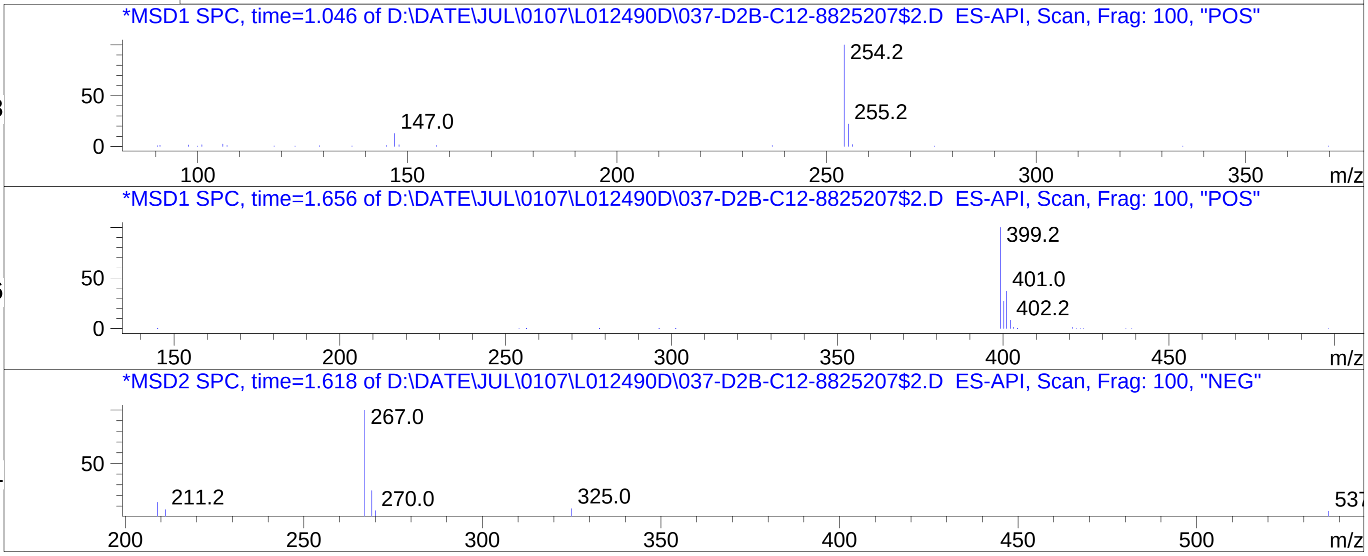

Inj.Date 7/1/2016 A <invalid> -6- Acq. Method C:\Chem32\-> ->
