# Supplementary material for: AI is a viable alternative to high throughput screening: a 318-target study
Source: Sci Rep. 2024 Apr 2;14:7526. doi: 10.1038/s41598-024-54655-z (PMC10987645; doi:10.1038/s41598-024-54655-z)

MaxPeak: 98.37%
Ret_Time: 1.470 min

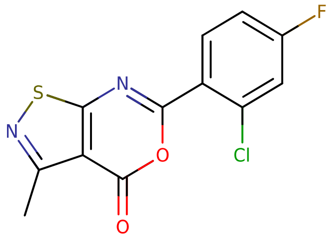

Mol Wt 296.7

Exact Mass 295.98

#	Time	Area%
1	1.238	1.63
2	1.470	98.37

T5343492

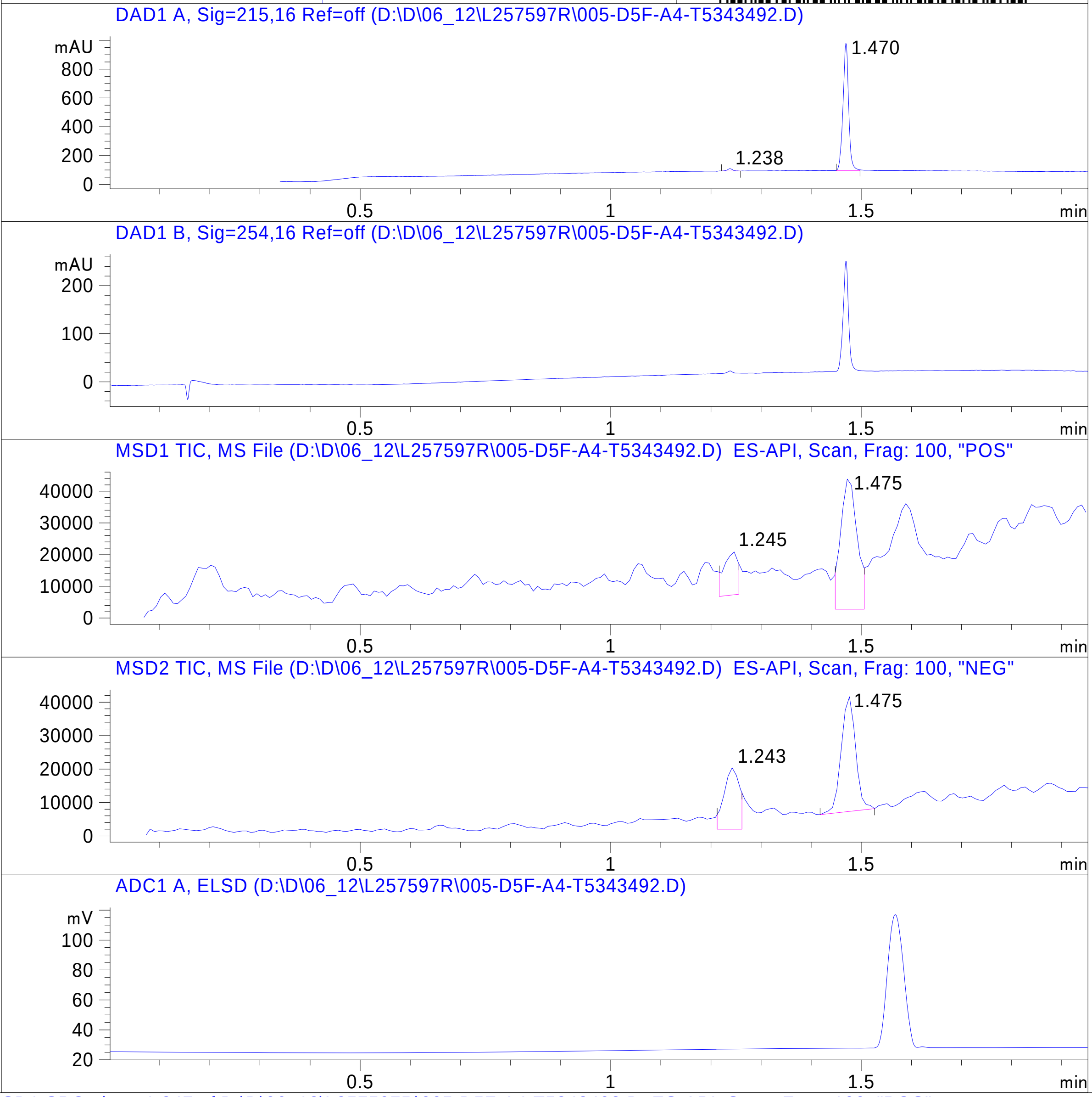

RT 1.245

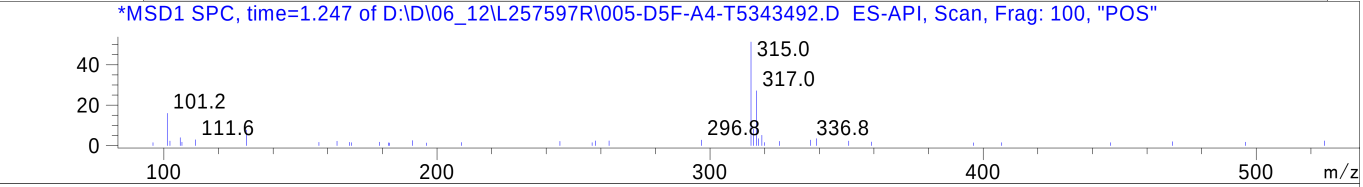

RT 1.475

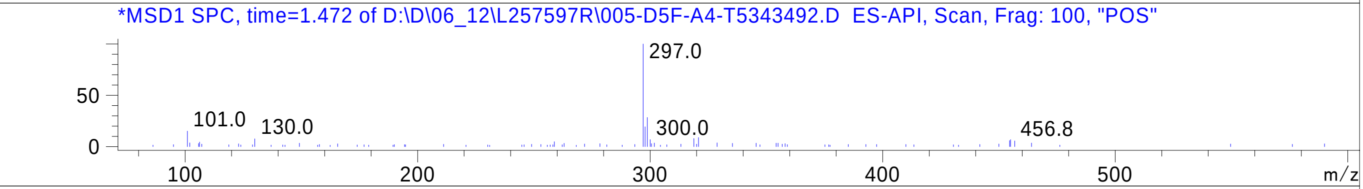

RT 1.243

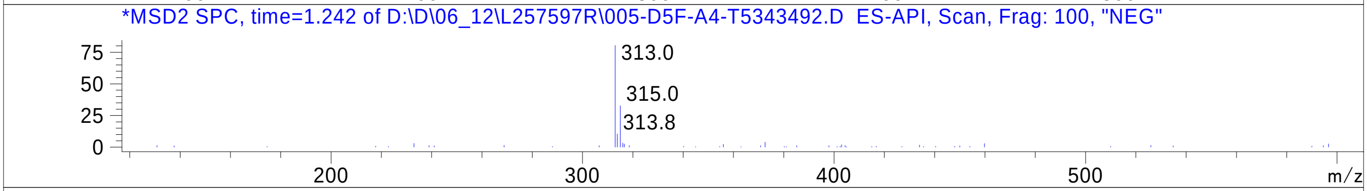

RT 1.475

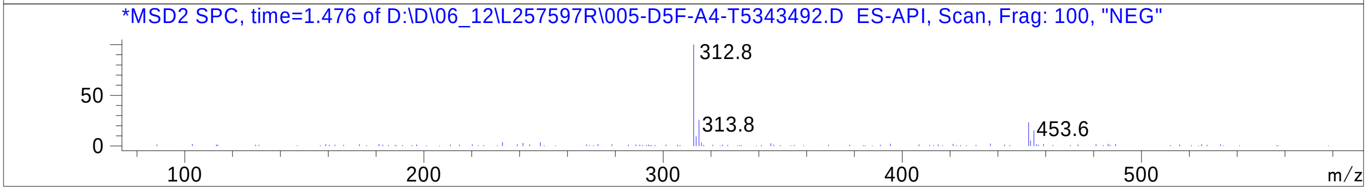

Supplement: Supplementary file 1 — Supplementary Information 1. [file 41598_2024_54655_MOESM1_ESM.zip › Nature SREP/QC_AIMS_files/Proj251.pdf]
