# Supplementary material for: AI is a viable alternative to high throughput screening: a 318-target study
Source: Sci Rep. 2024 Apr 2;14:7526. doi: 10.1038/s41598-024-54655-z (PMC10987645; doi:10.1038/s41598-024-54655-z)

MaxPeak: 97.32%
Ret_Time: 1.472 min

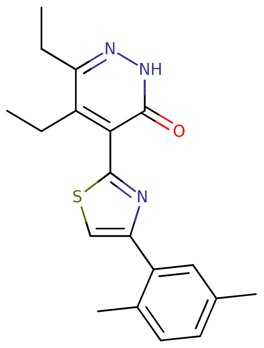

**Mol Wt** 339.46

**Exact Mass** 339.17

| # | Time | Area% |
|---|---|---|
| 1 | 1.196 | 2.68 |
| 2 | 1.472 | 97.32 |

T7372813

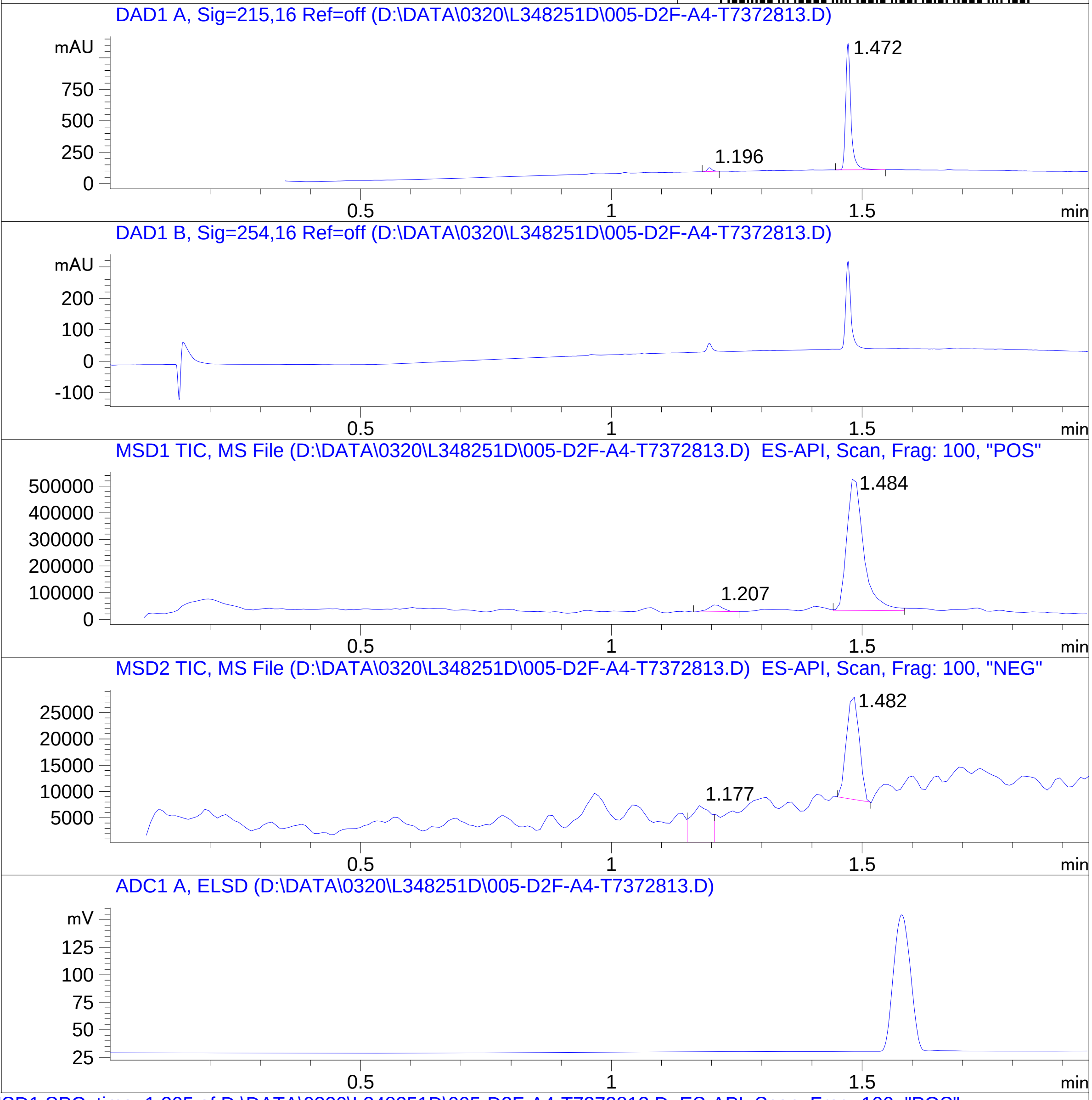


RT 1.207

RT 1.484

RT 1.177

RT 1.482

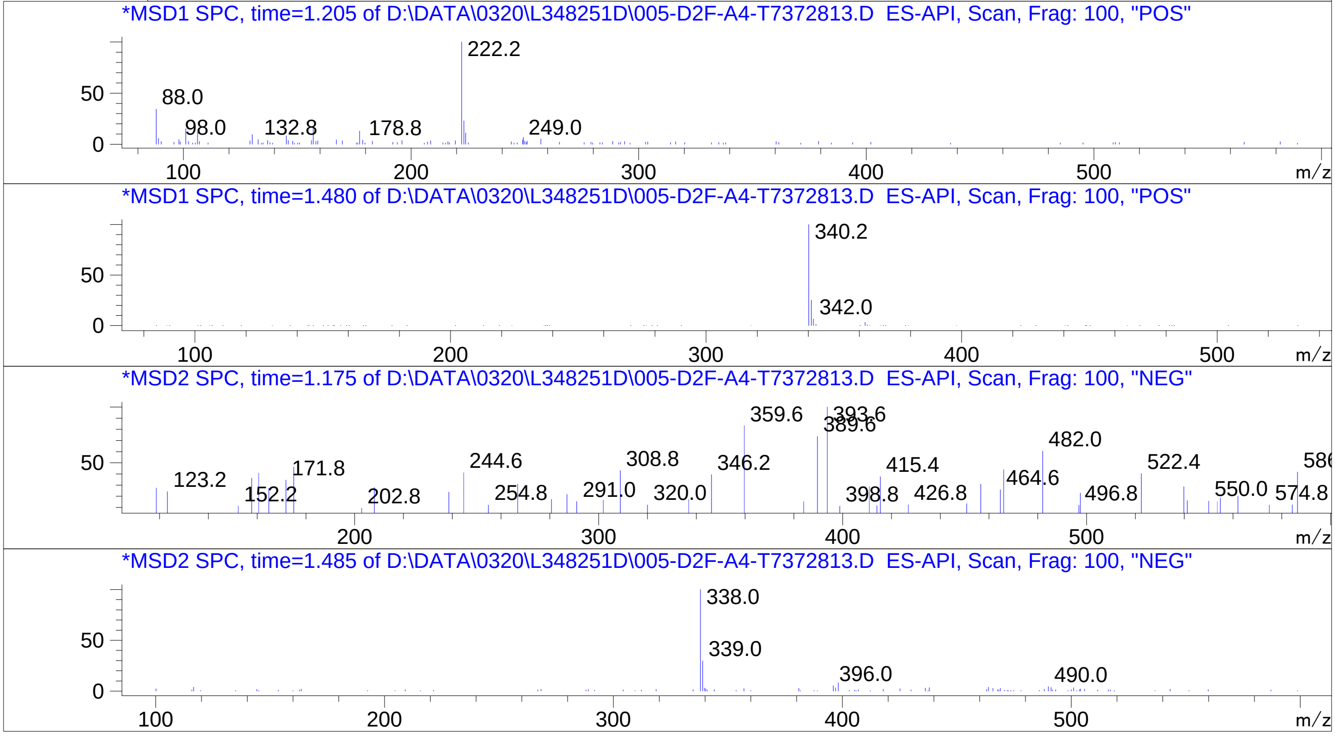

Supplement: Supplementary file 1 — Supplementary Information 1. [file 41598_2024_54655_MOESM1_ESM.zip › Nature SREP/QC_AIMS_files/Proj252.pdf]
