# Supplementary material for: AI is a viable alternative to high throughput screening: a 318-target study
Source: Sci Rep. 2024 Apr 2;14:7526. doi: 10.1038/s41598-024-54655-z (PMC10987645; doi:10.1038/s41598-024-54655-z)

MaxPeak: 93.98%
Ret_Time: 0.762 min

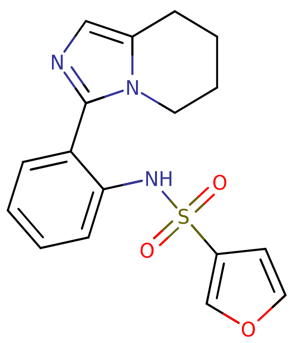

Mol Wt 343.4

Exact Mass 343.11

#	Time	Area%
1	0.675	6.02
2	0.762	93.98

T8443858

DAD1 A, Sig=215,16 Ref=off (D:\DATA\1223\L455943R-PART1\028-D5B-E8-T8443858.D)

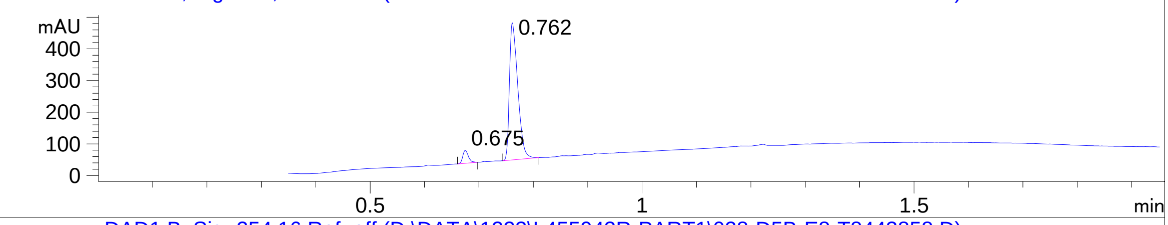

DAD1 B, Sig=254,16 Ref=off (D:\DATA\1223\L455943R-PART1\028-D5B-E8-T8443858.D)

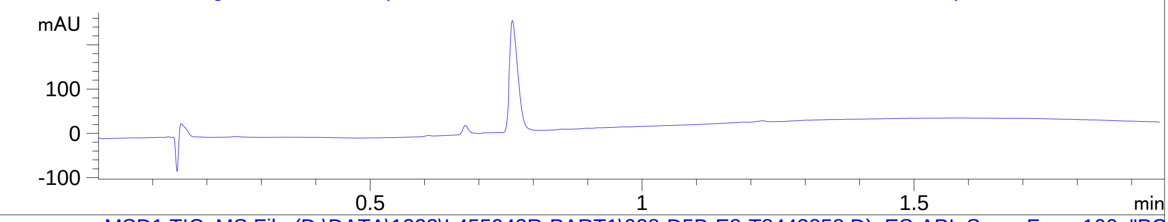

MSD1 TIC, MS File (D:\DATA\1223\L455943R-PART1\028-D5B-E8-T8443858.D) ES-API, Scan, Frag: 100, "PO

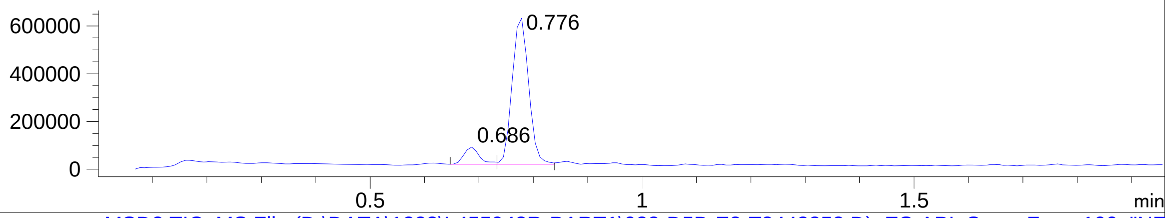

MSD2 TIC, MS File (D:\DATA\1223\L455943R-PART1\028-D5B-E8-T8443858.D) ES-API, Scan, Frag: 100, "NE

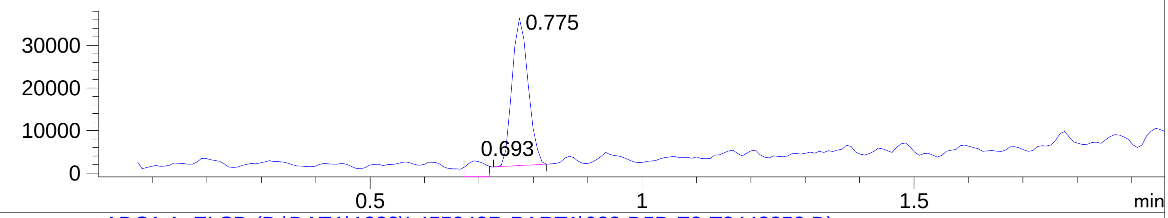

ADC1 A, ELSD (D:\DATA\1223\L455943R-PART1\028-D5B-E8-T8443858.D)

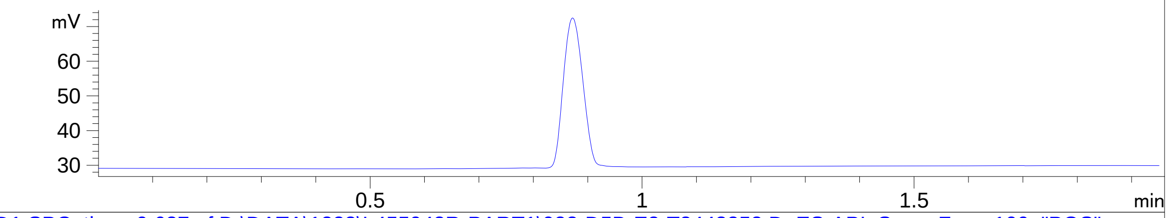

RT 0.686

*MSD1 SPC, time=0.687 of D:\DATA\1223\L455943R-PART1\028-D5B-E8-T8443858.D ES-API, Scan, Frag: 100, "POS"

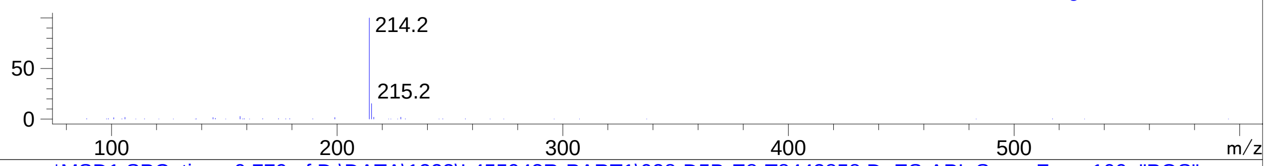

RT 0.776

*MSD1 SPC, time=0.779 of D:\DATA\1223\L455943R-PART1\028-D5B-E8-T8443858.D ES-API, Scan, Frag: 100, "POS"

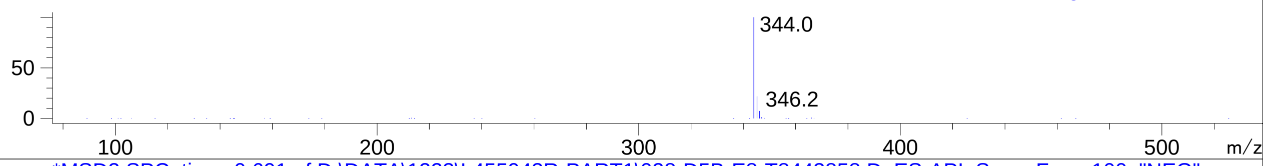

RT 0.693

*MSD2 SPC, time=0.691 of D:\DATA\1223\L455943R-PART1\028-D5B-E8-T8443858.D ES-API, Scan, Frag: 100, "NEG"

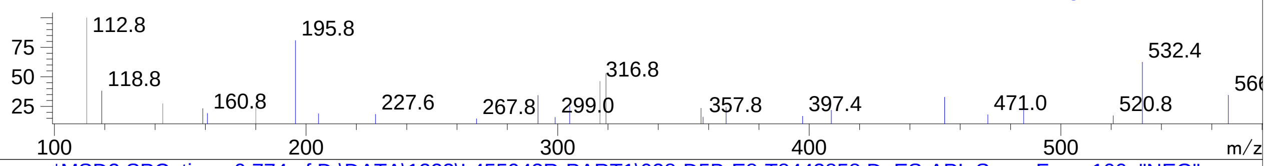

RT 0.775

*MSD2 SPC, time=0.774 of D:\DATA\1223\L455943R-PART1\028-D5B-E8-T8443858.D ES-API, Scan, Frag: 100, "NEG"

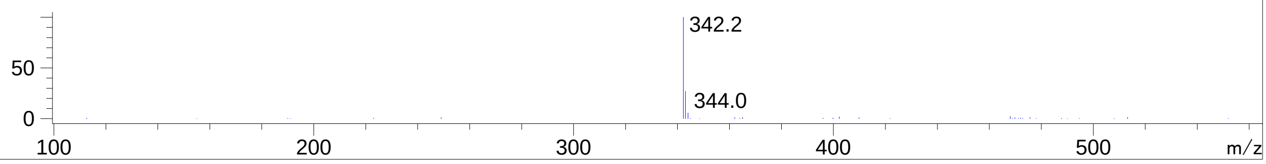

Supplement: Supplementary file 1 — Supplementary Information 1. [file 41598_2024_54655_MOESM1_ESM.zip › Nature SREP/QC_AIMS_files/Proj253.pdf]
