# Supplementary material for: AI is a viable alternative to high throughput screening: a 318-target study
Source: Sci Rep. 2024 Apr 2;14:7526. doi: 10.1038/s41598-024-54655-z (PMC10987645; doi:10.1038/s41598-024-54655-z)
Supplement: Supplementary file 1 — Supplementary Information 1. [file 41598_2024_54655_MOESM1_ESM.zip › Nature SREP/QC_AIMS_files/Proj254.pdf]

T5151152

MaxPeak: 91.42%
Ret_Time: 1.508 min

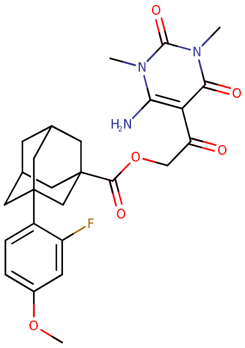

Mol Wt 499.53

Exact Mass 499.24

#	Time	Area%
1	0.621	2.24
2	0.668	1.43
3	1.405	4.91
4	1.508	91.42

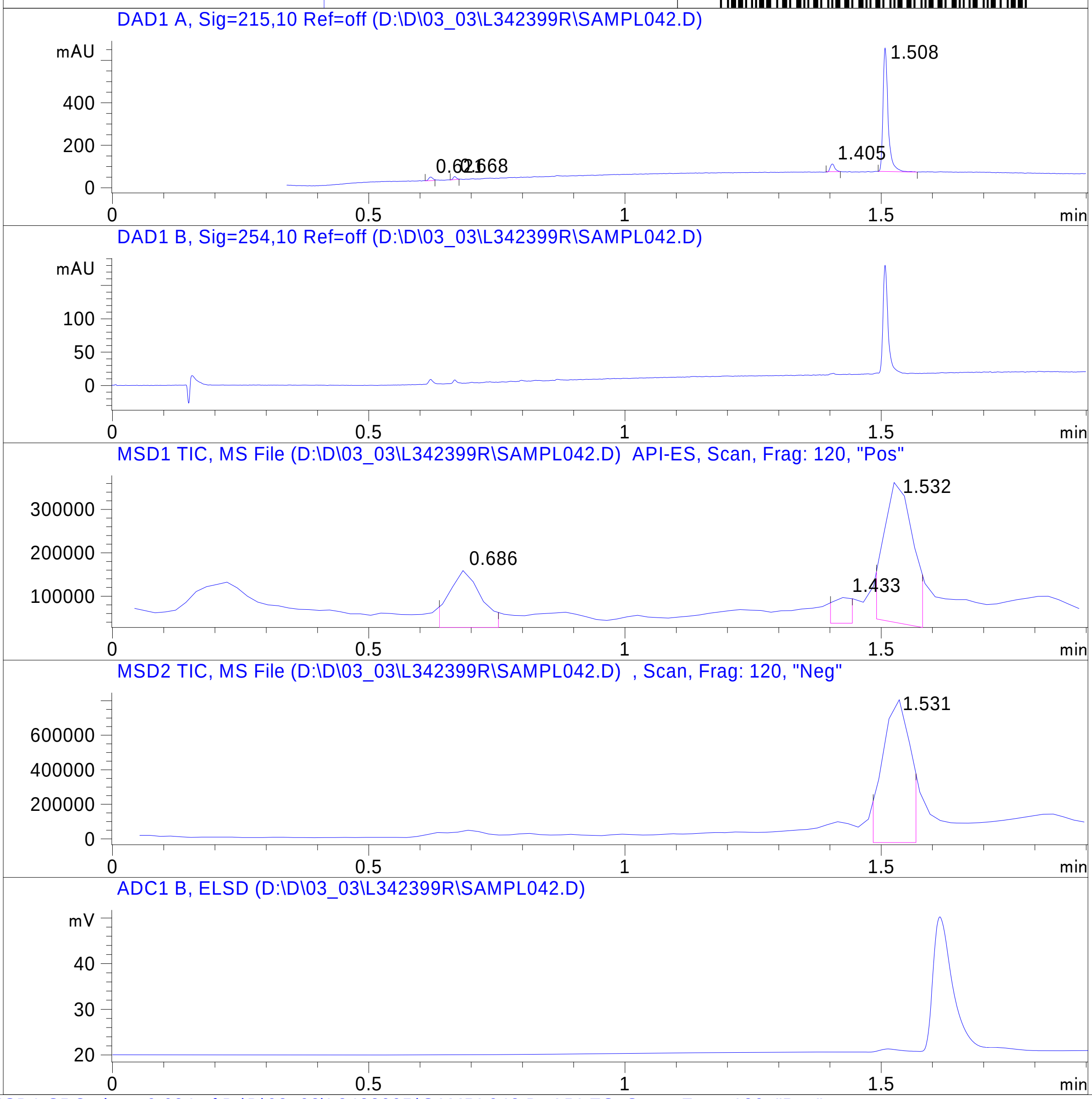

RT 0.686

RT 1.433

RT 1.532

RT 1.531

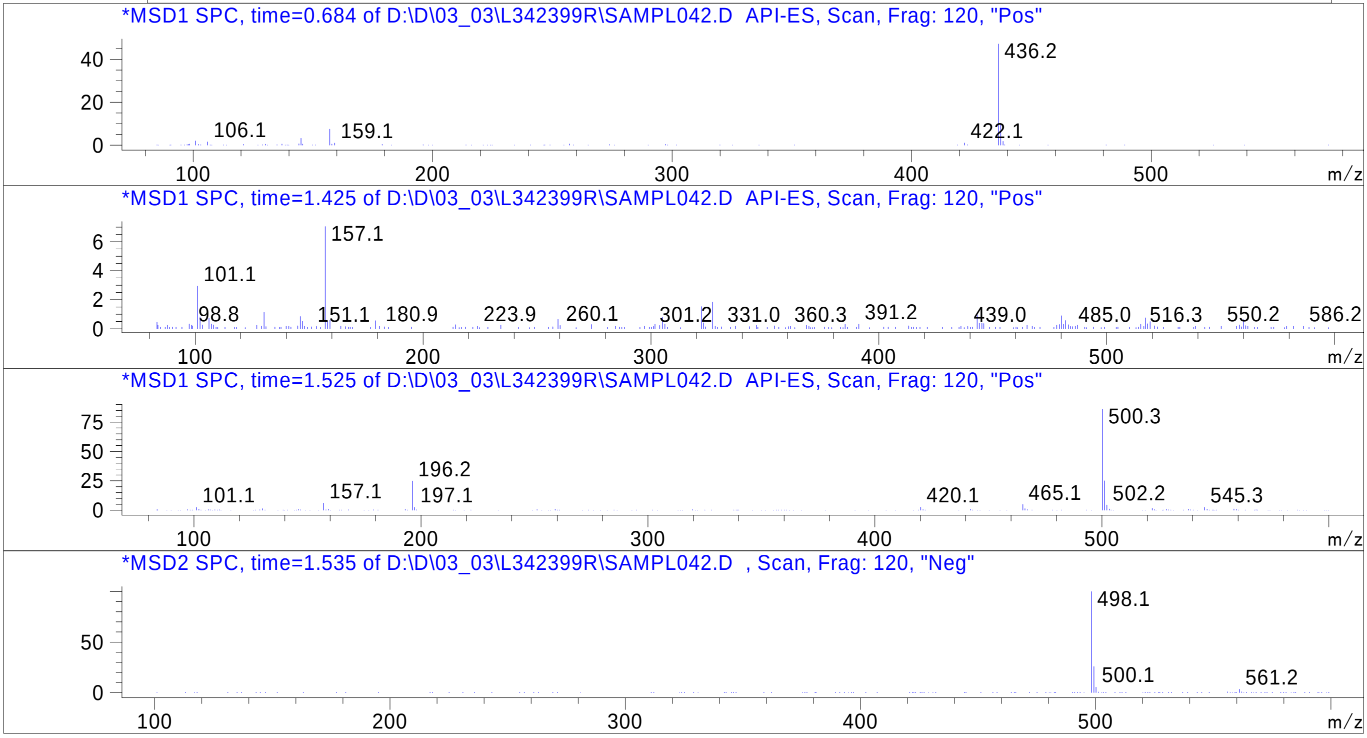

Inj.Date 3/4/2021 N -SL- Acq. Method C:\HPCHEM\-> ->
