# Supplementary material for: AI is a viable alternative to high throughput screening: a 318-target study
Source: Sci Rep. 2024 Apr 2;14:7526. doi: 10.1038/s41598-024-54655-z (PMC10987645; doi:10.1038/s41598-024-54655-z)

MaxPeak: 96.47%
Ret_Time: 1.439 min

S031508$1

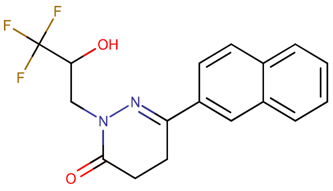

Mol Wt 336.31

Exact Mass 336.13

#	Time	Area%
1	1.439	96.47
2	1.590	3.53

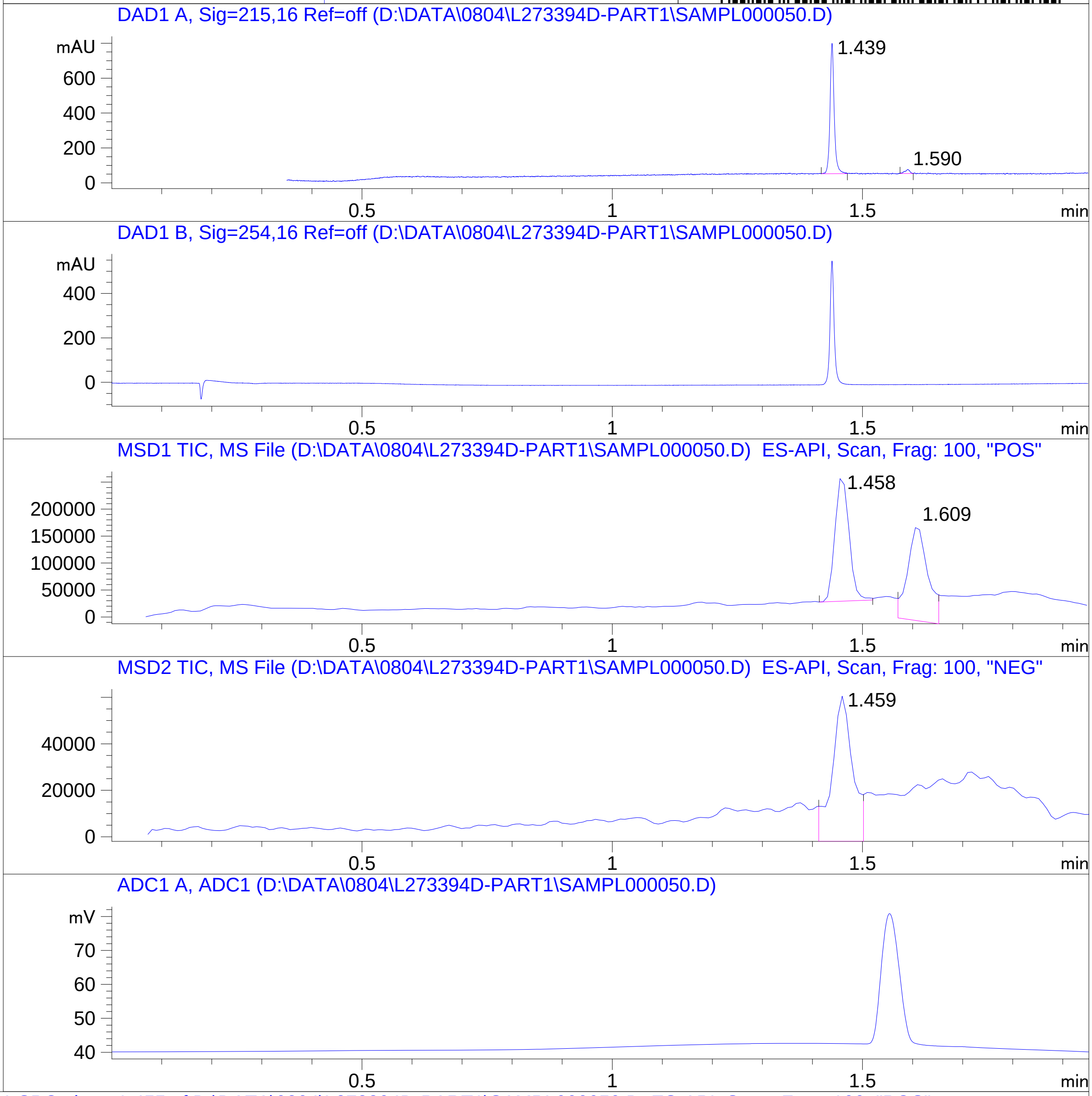

RT 1.458

RT 1.609

RT 1.459

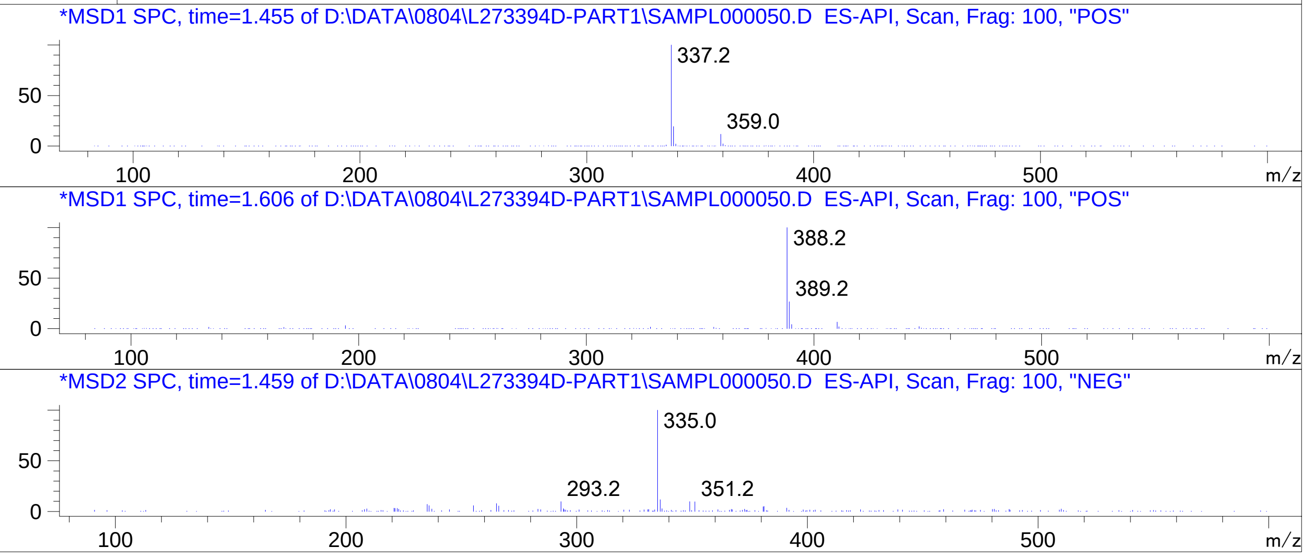

Supplement: Supplementary file 1 — Supplementary Information 1. [file 41598_2024_54655_MOESM1_ESM.zip › Nature SREP/QC_AIMS_files/Proj255.pdf]
