# Supplementary material for: AI is a viable alternative to high throughput screening: a 318-target study
Source: Sci Rep. 2024 Apr 2;14:7526. doi: 10.1038/s41598-024-54655-z (PMC10987645; doi:10.1038/s41598-024-54655-z)

T8396166

MaxPeak: 96.52%
Ret_Time: 0.967 min

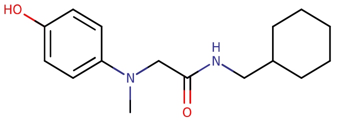

Mol Wt 276.37

Exact Mass 276.22

#	Time	Area%
1	0.821	1.09
2	0.967	96.52
3	1.153	1.04
4	1.352	1.35

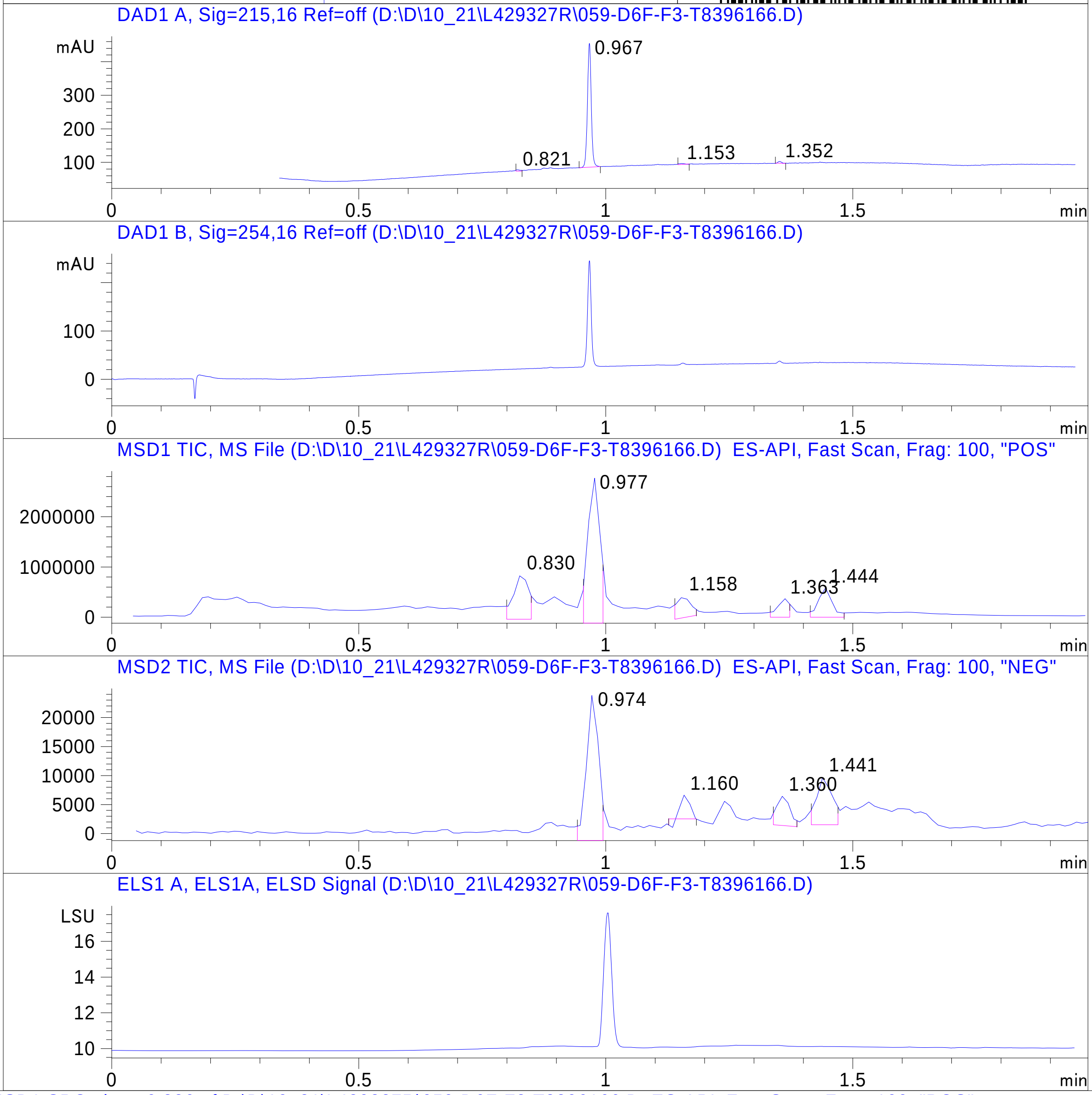

RT 0.830

RT 0.977

RT 1.158

RT 1.363

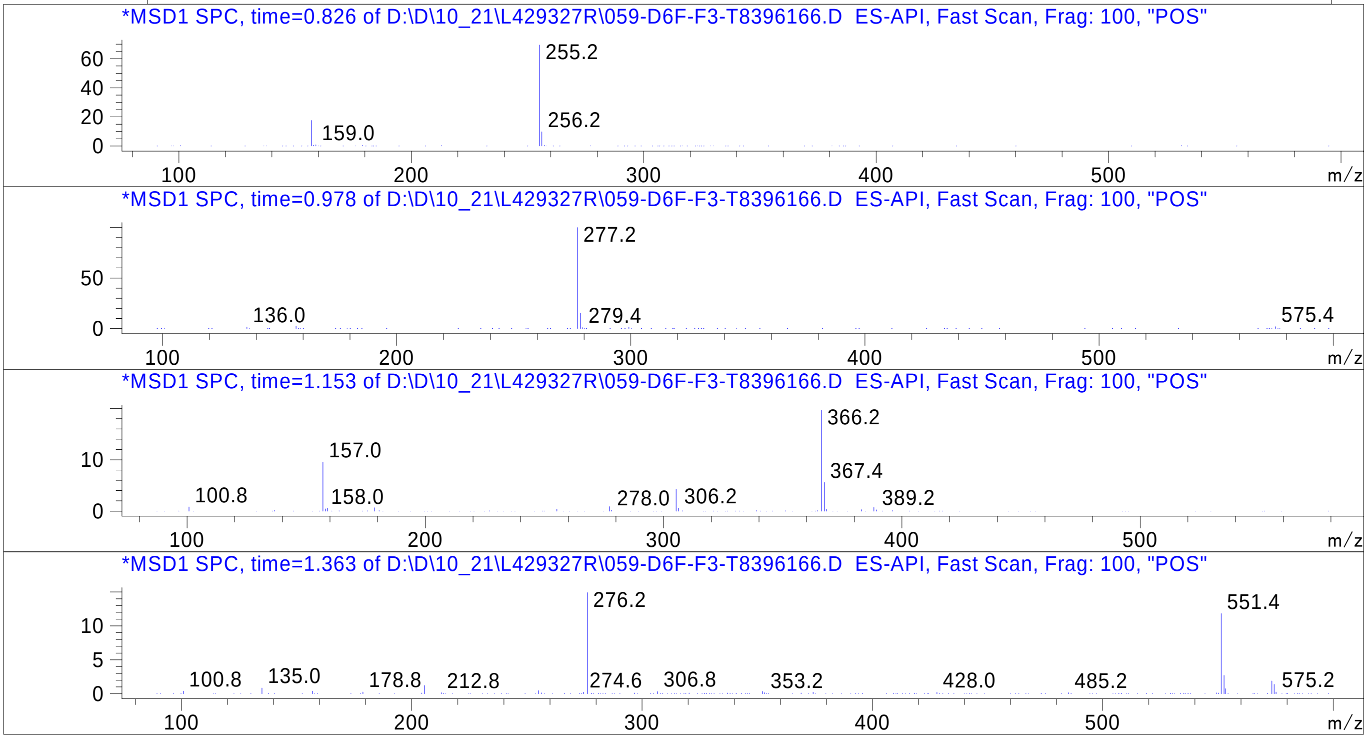

Inj.Date 10/21/2021 N Acq. Method C:\Users\ -> ->

RT 1.444

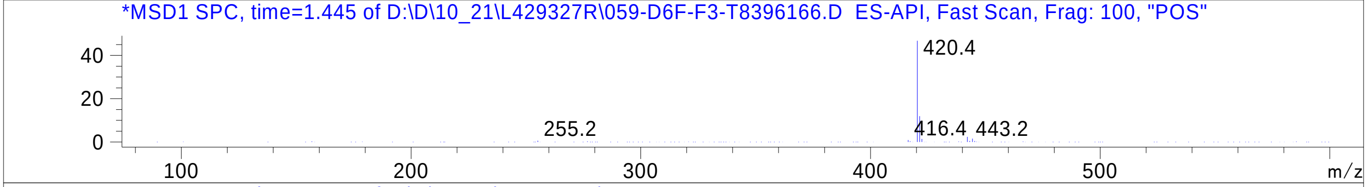

RT 0.974

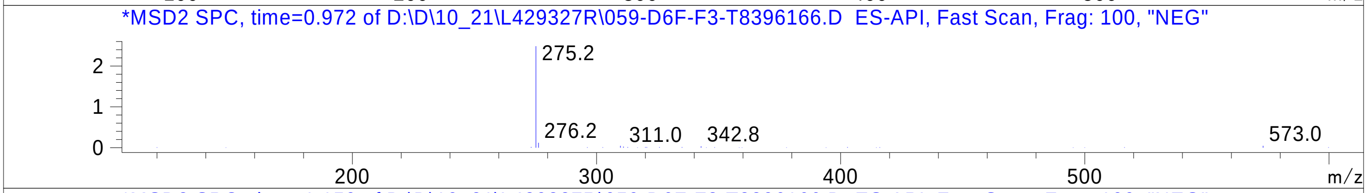

RT 1.160

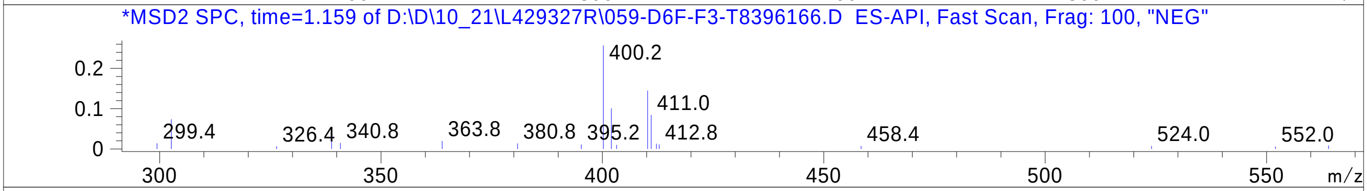

RT 1.360

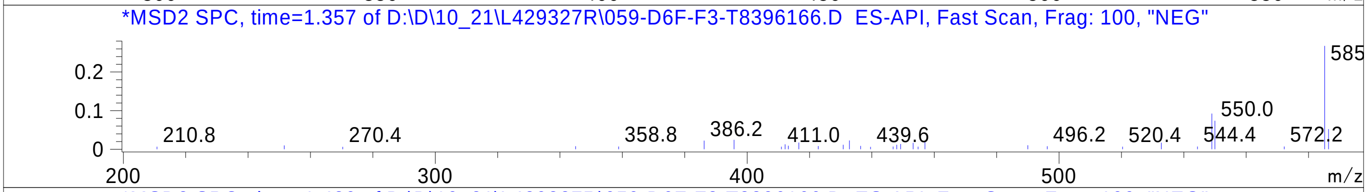

RT 1.441

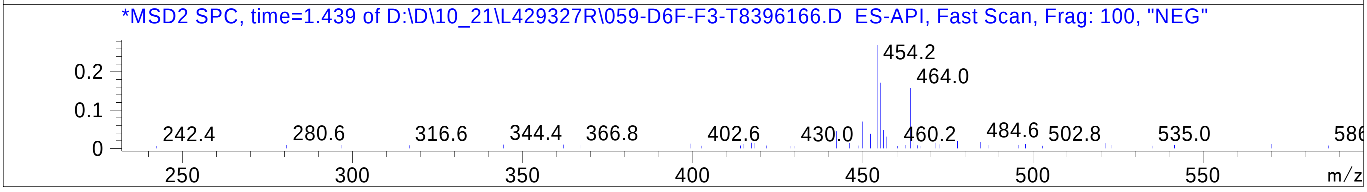

Supplement: Supplementary file 1 — Supplementary Information 1. [file 41598_2024_54655_MOESM1_ESM.zip › Nature SREP/QC_AIMS_files/Proj256.pdf]
