# Supplementary material for: AI is a viable alternative to high throughput screening: a 318-target study
Source: Sci Rep. 2024 Apr 2;14:7526. doi: 10.1038/s41598-024-54655-z (PMC10987645; doi:10.1038/s41598-024-54655-z)
Supplement: Supplementary file 1 — Supplementary Information 1. [file 41598_2024_54655_MOESM1_ESM.zip › Nature SREP/QC_AIMS_files/Proj257.pdf]

MaxPeak: 96.67%
Ret_Time: 1.116 min

6575680

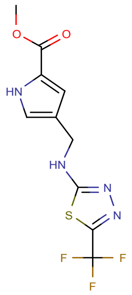

Mol Wt 306.26
Exact Mass 306.04

#	Time	Area%
1	0.711	3.33
2	1.116	96.67

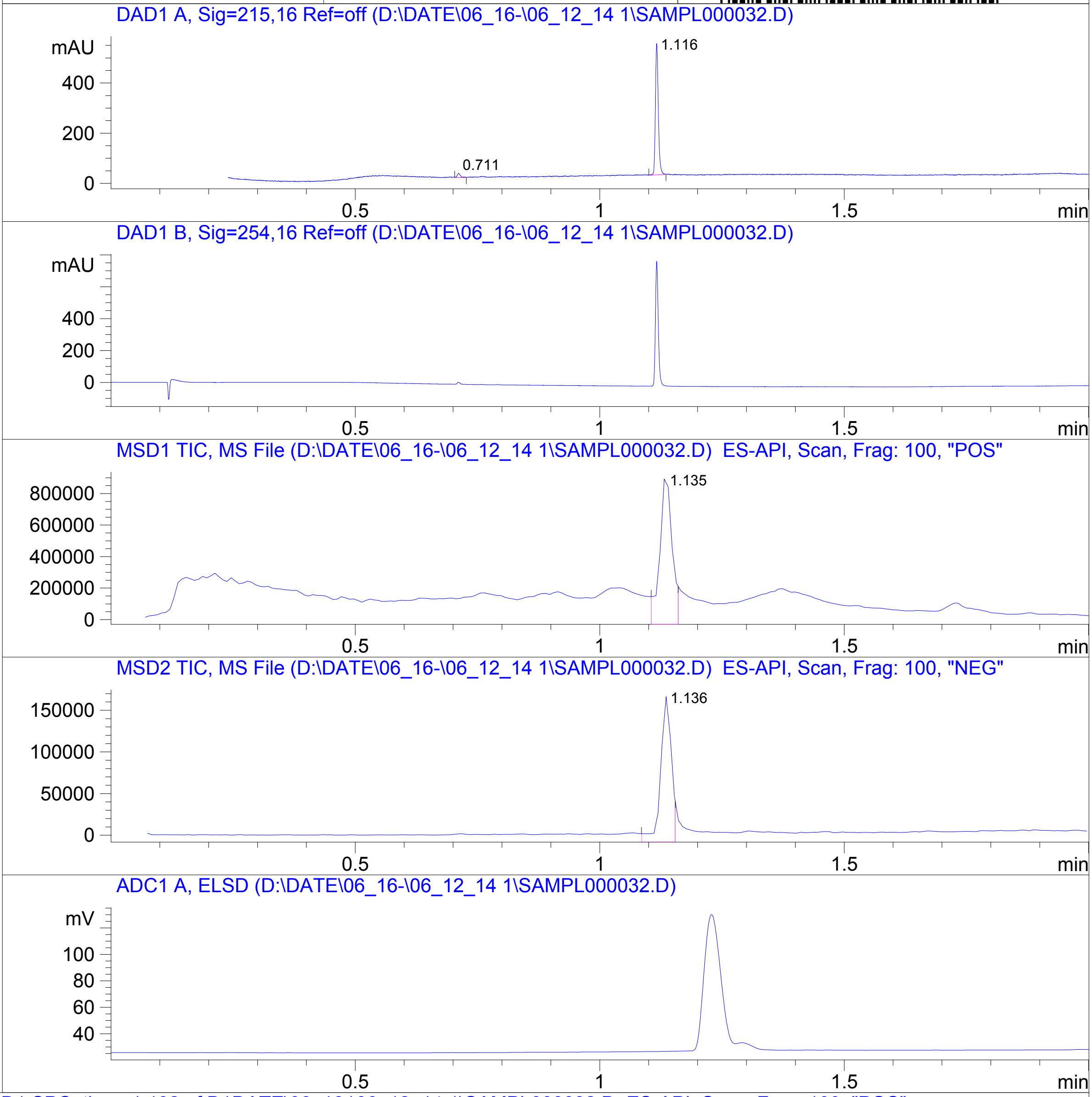

RT 1.135

RT 1.136

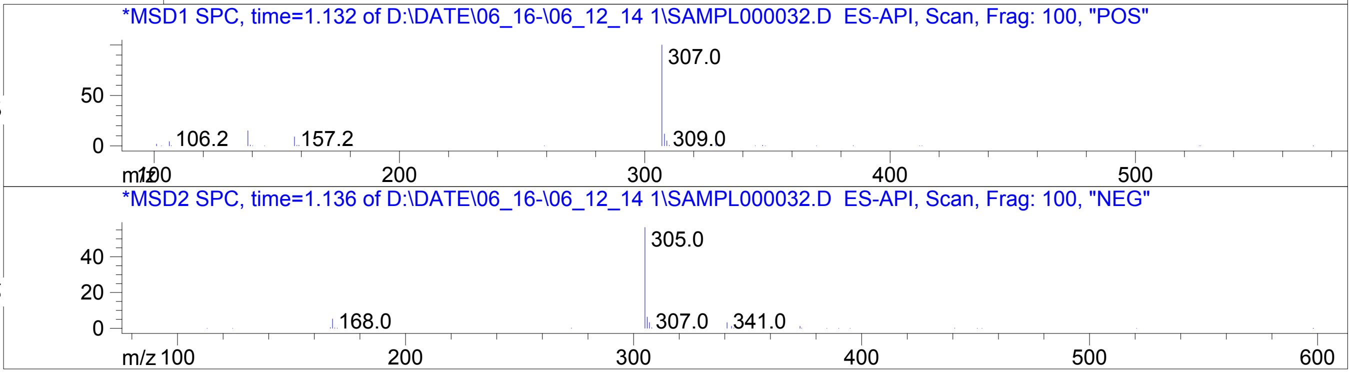

Inj.Date 6/15/2014 L P2-D-05 -5- Acq. Method C:\CHEM32\-> ->
