# Supplementary material for: AI is a viable alternative to high throughput screening: a 318-target study
Source: Sci Rep. 2024 Apr 2;14:7526. doi: 10.1038/s41598-024-54655-z (PMC10987645; doi:10.1038/s41598-024-54655-z)

MaxPeak: 93.74%
Ret_Time: 1.246 min

T8165847

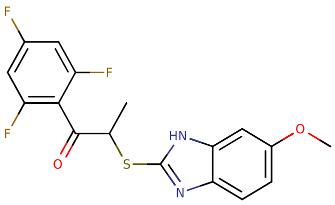

Mol Wt 366.36

Exact Mass 366.08

#	Time	Area%
1	0.787	6.26
2	1.246	93.74

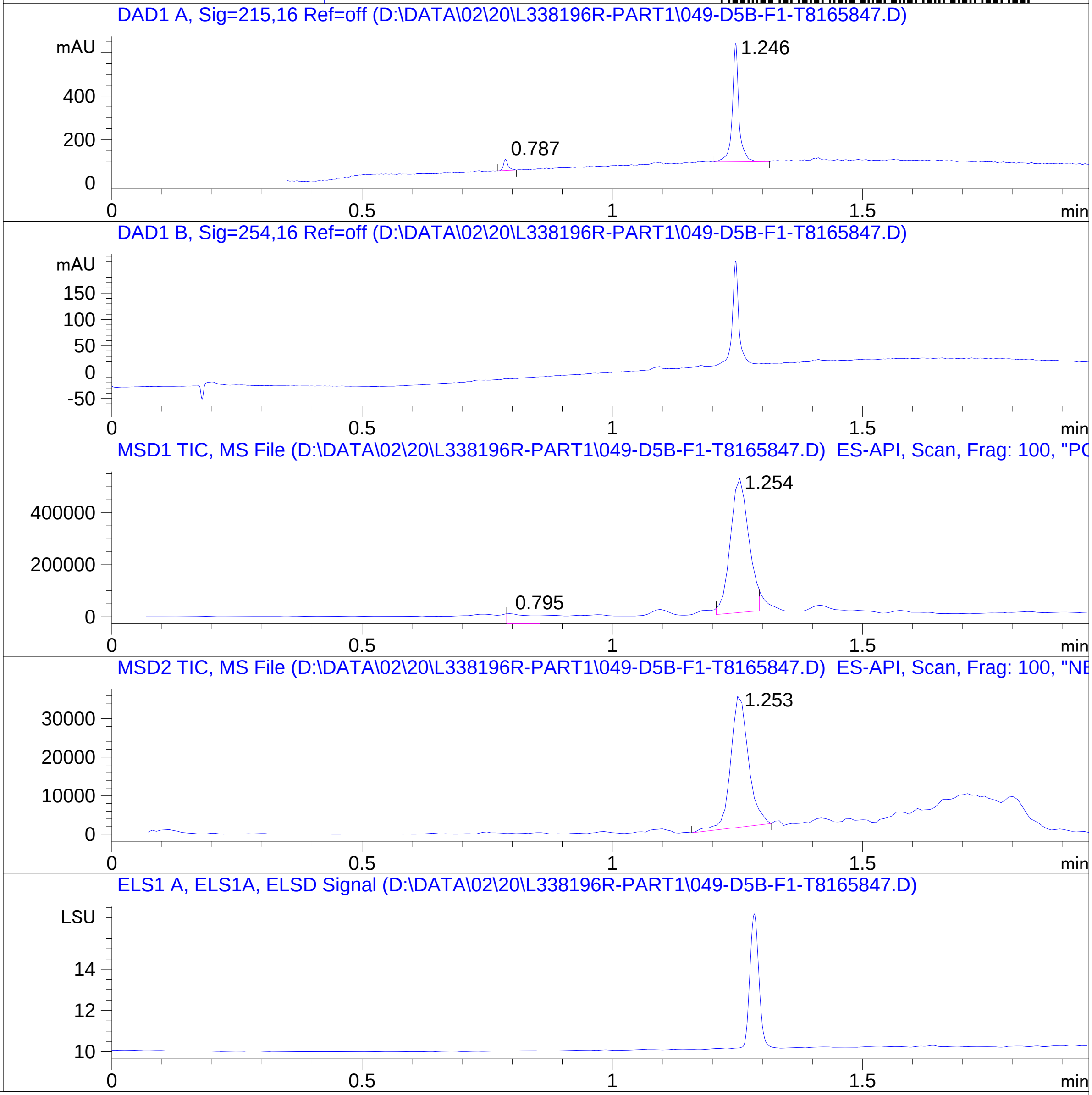

RT 0.795

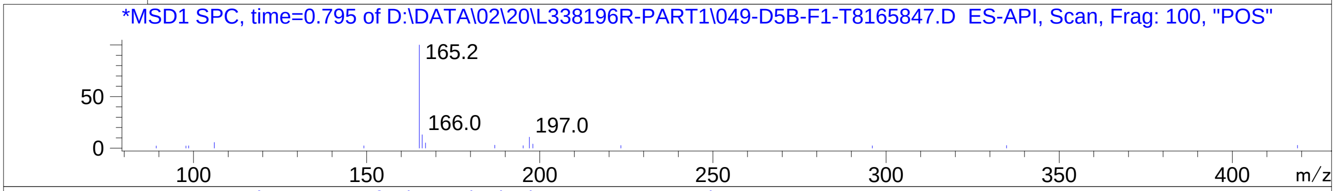

RT 1.254

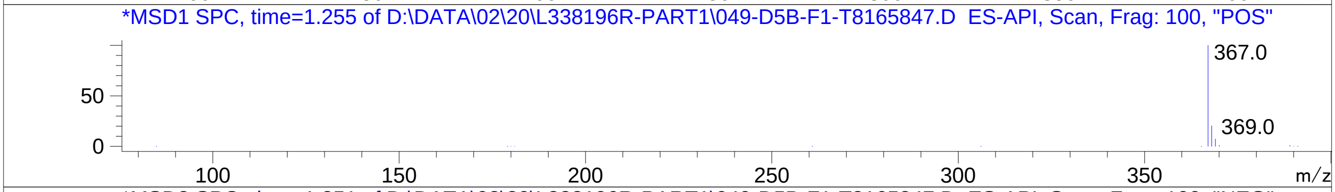

RT 1.253

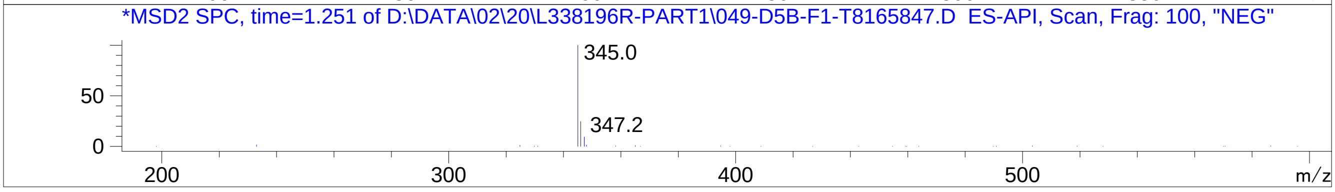

Supplement: Supplementary file 1 — Supplementary Information 1. [file 41598_2024_54655_MOESM1_ESM.zip › Nature SREP/QC_AIMS_files/Proj258.pdf]
