# Supplementary material for: AI is a viable alternative to high throughput screening: a 318-target study
Source: Sci Rep. 2024 Apr 2;14:7526. doi: 10.1038/s41598-024-54655-z (PMC10987645; doi:10.1038/s41598-024-54655-z)
Supplement: Supplementary file 1 — Supplementary Information 1. [file 41598_2024_54655_MOESM1_ESM.zip › Nature SREP/QC_AIMS_files/Proj260.pdf]

MA1631

70514-0454

(A)

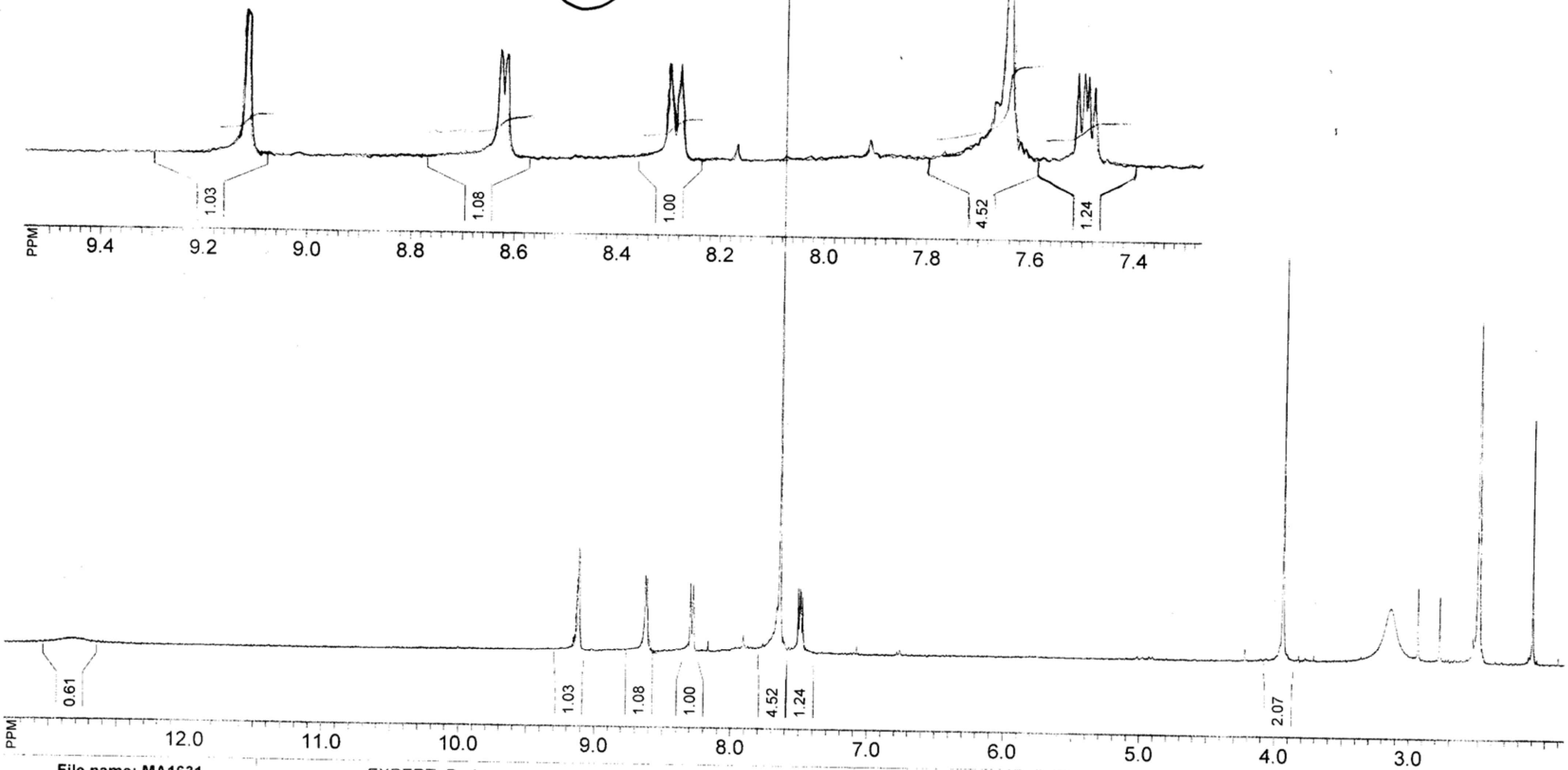


| | | | | | |
|---|---|---|---|---|---|
| **File name: MA1631** | EXPERT: Dmitry | SF: 400.3965 MHz | NSC: 0 | PW: 9.00 usec, RG: 30 | SI: 65536 |
| Date: 04-Sep-2002 | Solvent: DMSO-d6 + CCl4 | SW: 8000 Hz | TE: 293 K | AQ: 2.00 sec, RD: 0.00 sec | |
