# Supplementary material for: AI is a viable alternative to high throughput screening: a 318-target study
Source: Sci Rep. 2024 Apr 2;14:7526. doi: 10.1038/s41598-024-54655-z (PMC10987645; doi:10.1038/s41598-024-54655-z)

MaxPeak: 97.59%
Ret_Time: 1.319 min

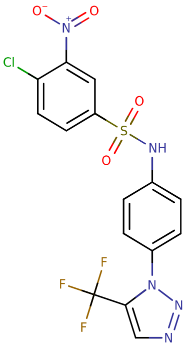

Mol Wt 447.78

Exact Mass 446.99

#	Time	Area%
1	1.012	2.41
2	1.319	97.59

T6771403

DAD1 A, Sig=215,16 Ref=off (D:\DATA\0203\L470487R\035D5B-D3T6771403.D)

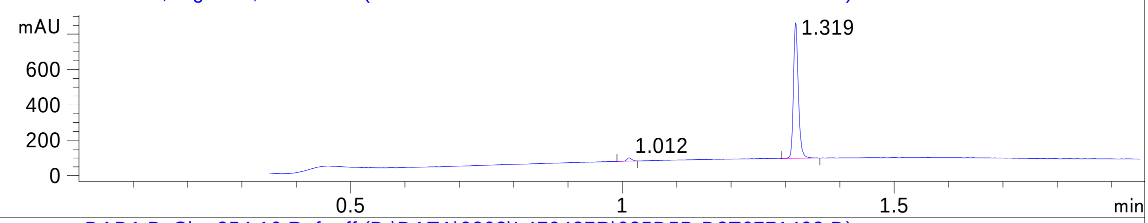

DAD1 B, Sig=254,16 Ref=off (D:\DATA\0203\L470487R\035D5B-D3T6771403.D)

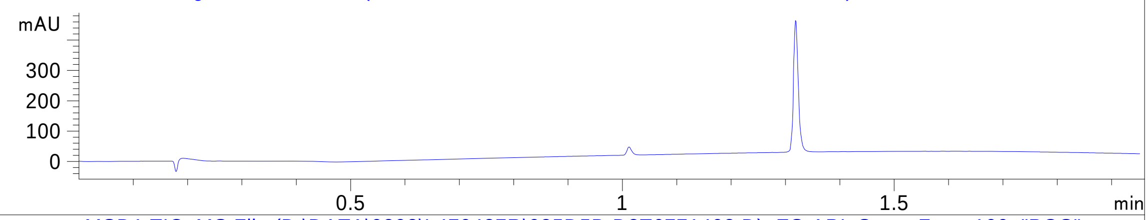

MSD1 TIC, MS File (D:\DATA\0203\L470487R\035D5B-D3T6771403.D) ES-API, Scan, Frag: 100, "POS"

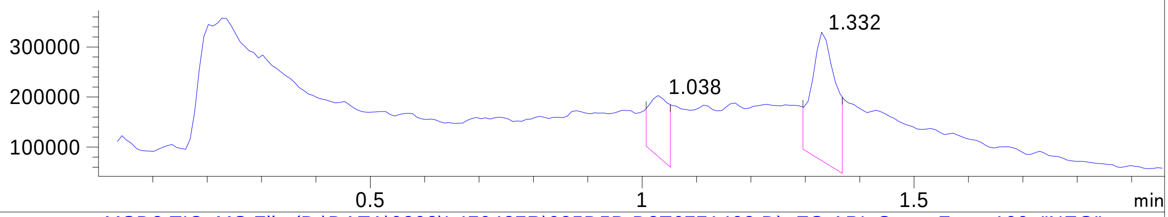

MSD2 TIC, MS File (D:\DATA\0203\L470487R\035D5B-D3T6771403.D) ES-API, Scan, Frag: 100, "NEG"

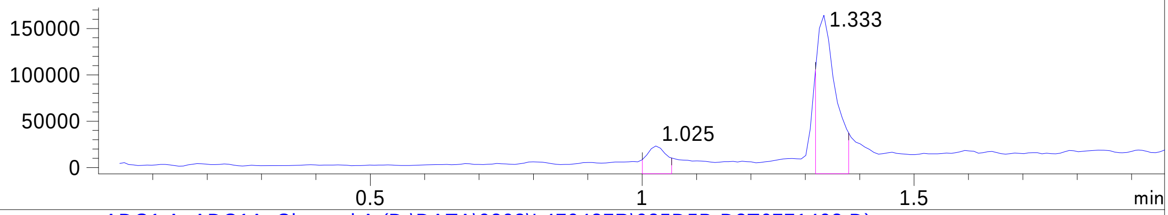

ADC1 A, ADC1A, Channel A (D:\DATA\0203\L470487R\035D5B-D3T6771403.D)

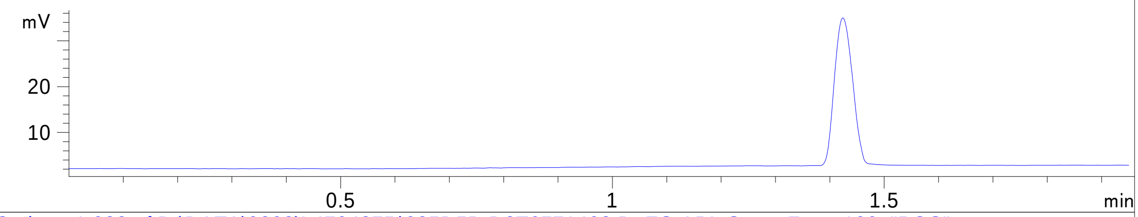

RT 1.038

*MSD1 SPC, time=1.038 of D:\DATA\0203\L470487R\035D5B-D3T6771403.D ES-API, Scan, Frag: 100, "POS"

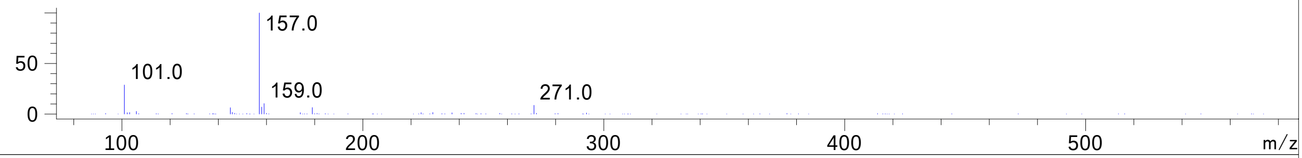

RT 1.332

*MSD1 SPC, time=1.330 of D:\DATA\0203\L470487R\035D5B-D3T6771403.D ES-API, Scan, Frag: 100, "POS"

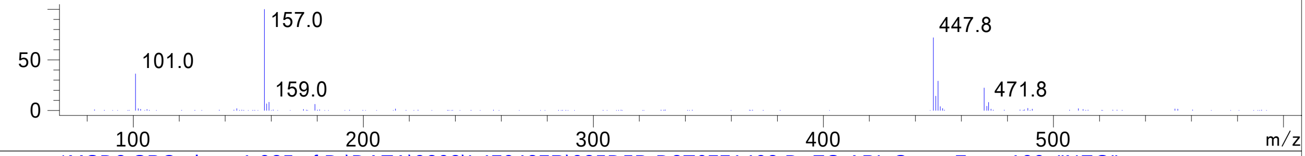

RT 1.025

*MSD2 SPC, time=1.025 of D:\DATA\0203\L470487R\035D5B-D3T6771403.D ES-API, Scan, Frag: 100, "NEG"

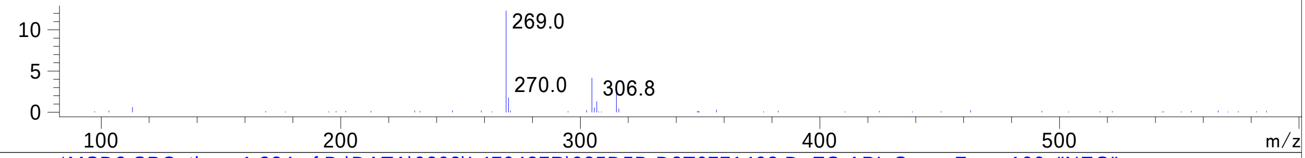

RT 1.333

*MSD2 SPC, time=1.334 of D:\DATA\0203\L470487R\035D5B-D3T6771403.D ES-API, Scan, Frag: 100, "NEG"

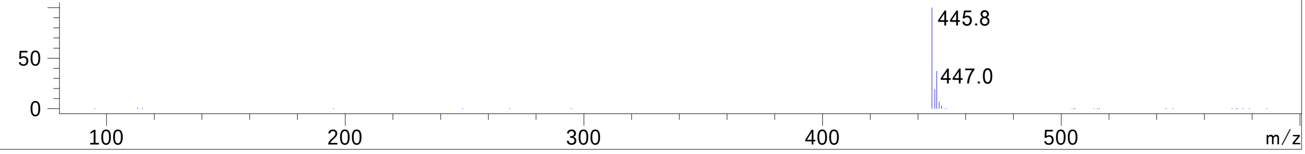

Supplement: Supplementary file 1 — Supplementary Information 1. [file 41598_2024_54655_MOESM1_ESM.zip › Nature SREP/QC_AIMS_files/Proj262.pdf]
