# Supplementary material for: AI is a viable alternative to high throughput screening: a 318-target study
Source: Sci Rep. 2024 Apr 2;14:7526. doi: 10.1038/s41598-024-54655-z (PMC10987645; doi:10.1038/s41598-024-54655-z)
Supplement: Supplementary file 1 — Supplementary Information 1. [file 41598_2024_54655_MOESM1_ESM.zip › Nature SREP/QC_AIMS_files/Proj263.pdf]

MaxPeak: 100.00%
Ret_Time: 0.803 min

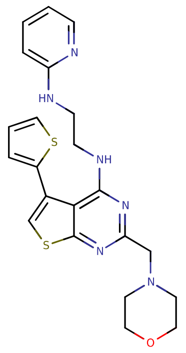

Mol Wt 452.6

Exact Mass 452.17

#	Time	Area%
1	0.803	100.00

T7994735

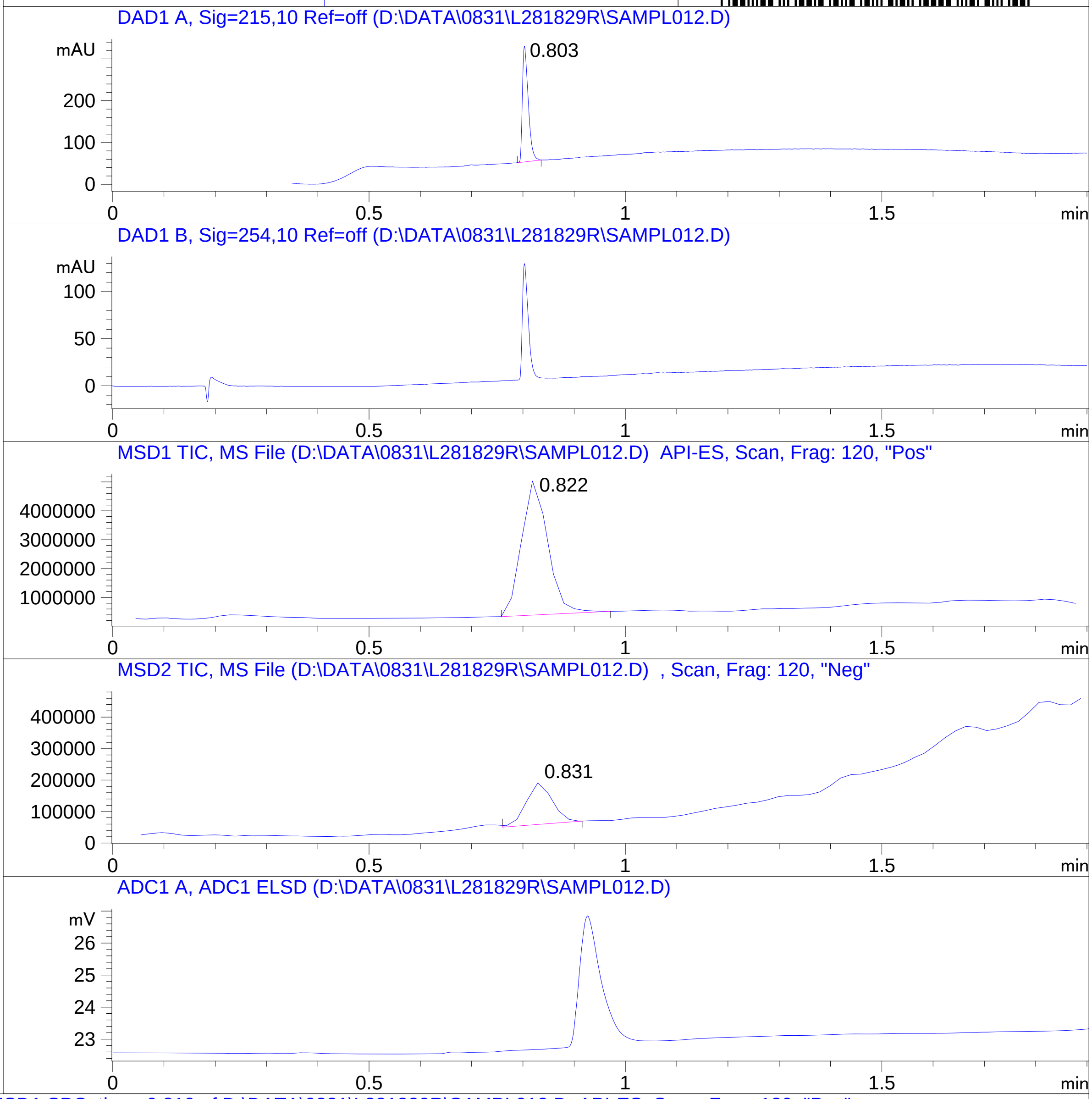

RT 0.822

RT 0.831

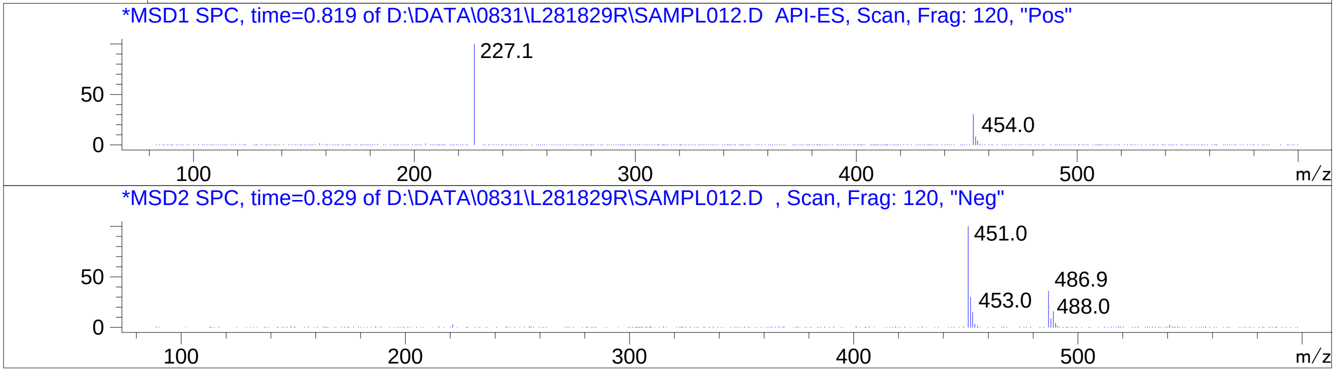

Inj.Date 8/31/2020 OA -VL- Acq. Method C:\HPCHEM\-> ->
