# Supplementary material for: AI is a viable alternative to high throughput screening: a 318-target study
Source: Sci Rep. 2024 Apr 2;14:7526. doi: 10.1038/s41598-024-54655-z (PMC10987645; doi:10.1038/s41598-024-54655-z)
Supplement: Supplementary file 1 — Supplementary Information 1. [file 41598_2024_54655_MOESM1_ESM.zip › Nature SREP/QC_AIMS_files/Proj268.pdf]

MaxPeak: 97.04%
Ret_Time: 1.490 min

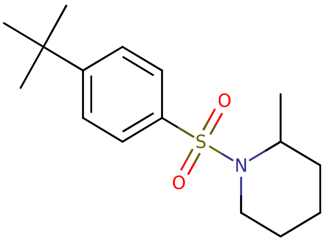

Mol Wt 295.44

Exact Mass 295.2

#	Time	Area%
1	0.653	2.96
2	1.490	97.04

1212980944

DAD1 A, Sig=215,16 Ref=off (D:\DATA\05\15\L248887R\010-D1B-B8-1212980944.D)

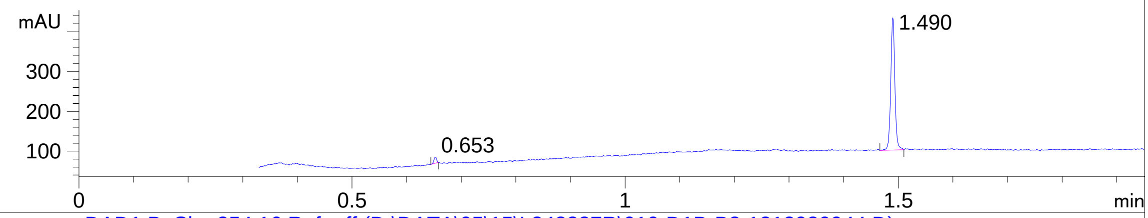

DAD1 B, Sig=254,16 Ref=off (D:\DATA\05\15\L248887R\010-D1B-B8-1212980944.D)

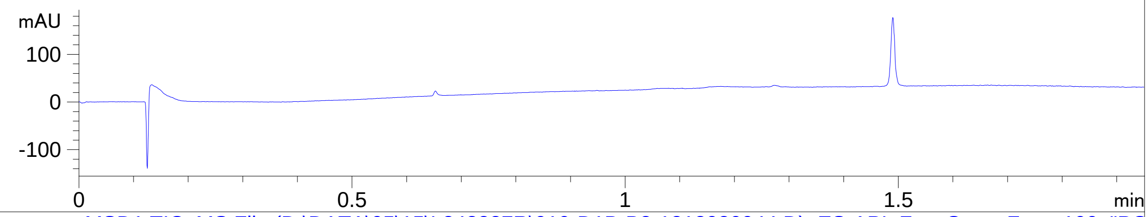

MSD1 TIC, MS File (D:\DATA\05\15\L248887R\010-D1B-B8-1212980944.D) ES-API, Fast Scan, Frag: 100, "PO

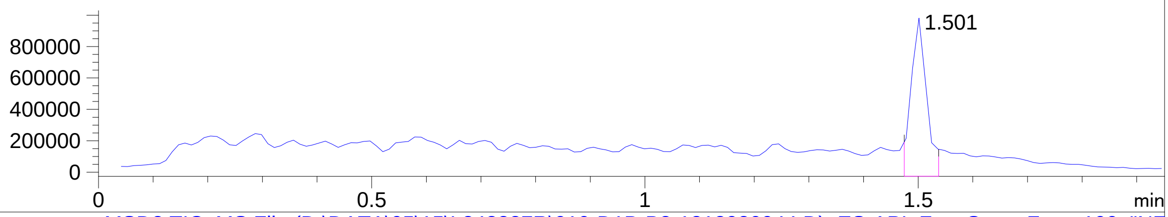

MSD2 TIC, MS File (D:\DATA\05\15\L248887R\010-D1B-B8-1212980944.D) ES-API, Fast Scan, Frag: 100, "NE

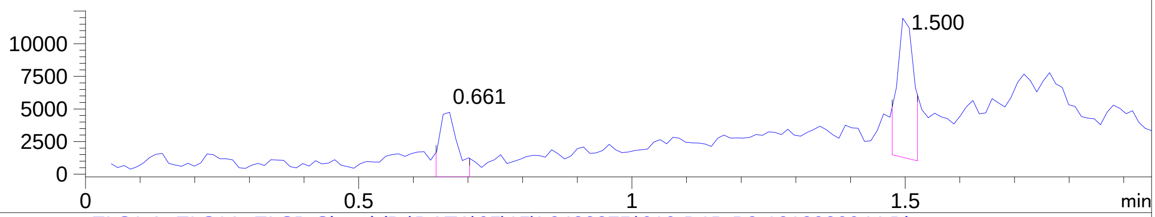

ELS1 A, ELS1A, ELSD Signal (D:\DATA\05\15\L248887R\010-D1B-B8-1212980944.D)

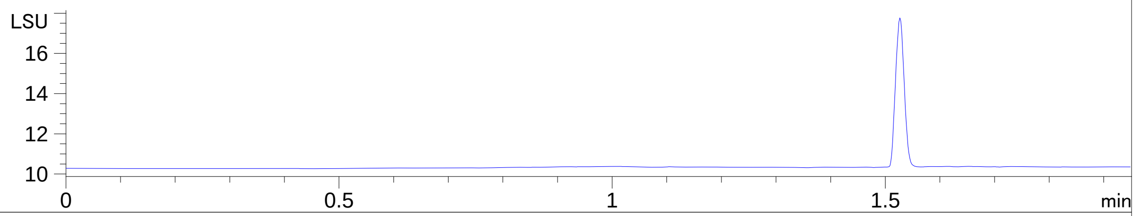

RT 1.501

*MSD1 SPC, time=1.501 of D:\DATA\05\15\L248887R\010-D1B-B8-1212980944.D ES-API, Fast Scan, Frag: 100, "POS"

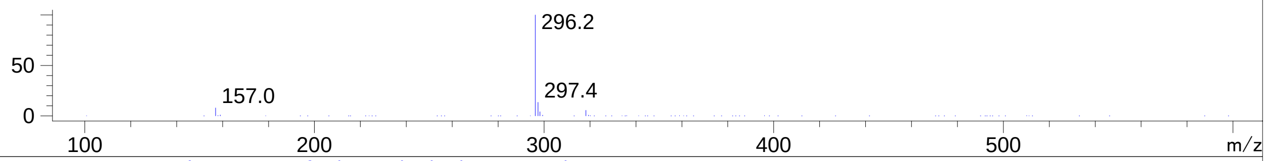

RT 0.661

*MSD2 SPC, time=0.666 of D:\DATA\05\15\L248887R\010-D1B-B8-1212980944.D ES-API, Fast Scan, Frag: 100, "NEG"

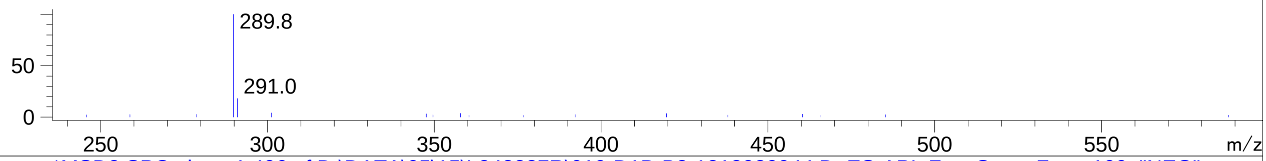

RT 1.500

*MSD2 SPC, time=1.496 of D:\DATA\05\15\L248887R\010-D1B-B8-1212980944.D ES-API, Fast Scan, Frag: 100, "NEG"

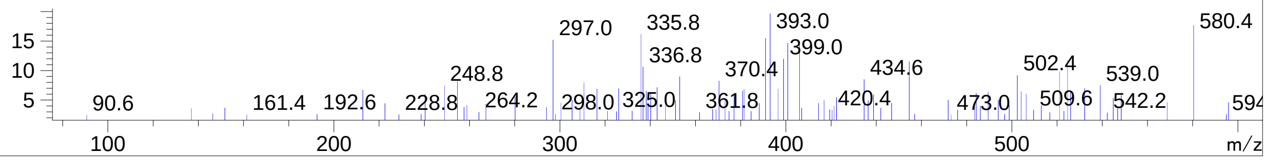

Inj.Date 5/15/2020   LB   Acq. Method C:\Users\ ->   ->
