# Supplementary material for: AI is a viable alternative to high throughput screening: a 318-target study
Source: Sci Rep. 2024 Apr 2;14:7526. doi: 10.1038/s41598-024-54655-z (PMC10987645; doi:10.1038/s41598-024-54655-z)
Supplement: Supplementary file 1 — Supplementary Information 1. [file 41598_2024_54655_MOESM1_ESM.zip › Nature SREP/QC_AIMS_files/Proj269.doc]

```
Data File R:\HPLC\AUTO\FORPIL~1\FC\FC9427~1\2CB-1401.D
Sample Name: FC9427015P2-C-02
Instrument 1 18/02/2019 15:32:46
Column: Onyx C18 50x4.6mm | 3.75ml/min | Columns Reg Valve
Gradient: "A"->@2.0min->"B"(Hold 0.6min)->@0.2min->"A"->PostRun
PMP1, Solvent A         : 0.1%TFA, 2.5%AcN in H2O
PMP1, Solvent B         : 0.1%TFA in AcN
PMP1, Solvent C         : --NOT USED--
PMP1, Solvent D         : --NOT USED--
Ionization mode             :    API-ES Positive

Signal 1: ADC1 B, ELSD
Peak RetTime Type  Width     Area      Height     Area
  #   [min]        [min]   [mV*s]      [mV]         %
----|-------|----|-------|----------|----------|--------|
   1   1.478 PV    0.0297   14.67814    7.55927   5.9919
   2   1.541 MF    0.0375  224.57251   99.92152  91.6754
   3   1.611 FM    0.0224    5.71425    4.24762   2.3327
Totals :                   244.96490  111.72841


Signal 2: DAD1 A, Sig=300,200 Ref=off
Peak RetTime Type  Width     Area      Height     Area
  #   [min]        [min]   [mAU*s]     [mAU]        %
----|-------|----|-------|----------|----------|--------|
   1   1.401 BV    0.0234  148.38150   99.46256   7.9604
   2   1.466 VB    0.0262 1666.75818 1067.40784  89.4189
   3   1.591 BP    0.0254   48.84812   32.76331   2.6206
Totals :                  1863.98780 1199.63371


Signal 3: MSD1 TIC, MS File
Peak RetTime Type  Width     Area      Height     Area
  #   [min]        [min]                            %
----|-------|----|-------|----------|----------|--------|
   1   1.490 MM    0.0357 7.47334e5  3.49000e5  100.0000
Totals :                  7.47334e5  3.49000e5
```
