# Supplementary material for: AI is a viable alternative to high throughput screening: a 318-target study
Source: Sci Rep. 2024 Apr 2;14:7526. doi: 10.1038/s41598-024-54655-z (PMC10987645; doi:10.1038/s41598-024-54655-z)
Supplement: Supplementary file 1 — Supplementary Information 1. [file 41598_2024_54655_MOESM1_ESM.zip › Nature SREP/QC_AIMS_files/Proj271.pdf]

MaxPeak: 96.16%
Ret_Time: 0.492 min

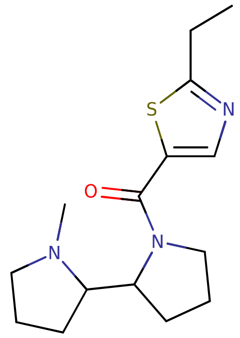

Mol Wt 293.43

Exact Mass 293.19

#	Time	Area%
1	0.472	1.64
2	0.492	96.16
3	0.919	2.19

T13296764$2

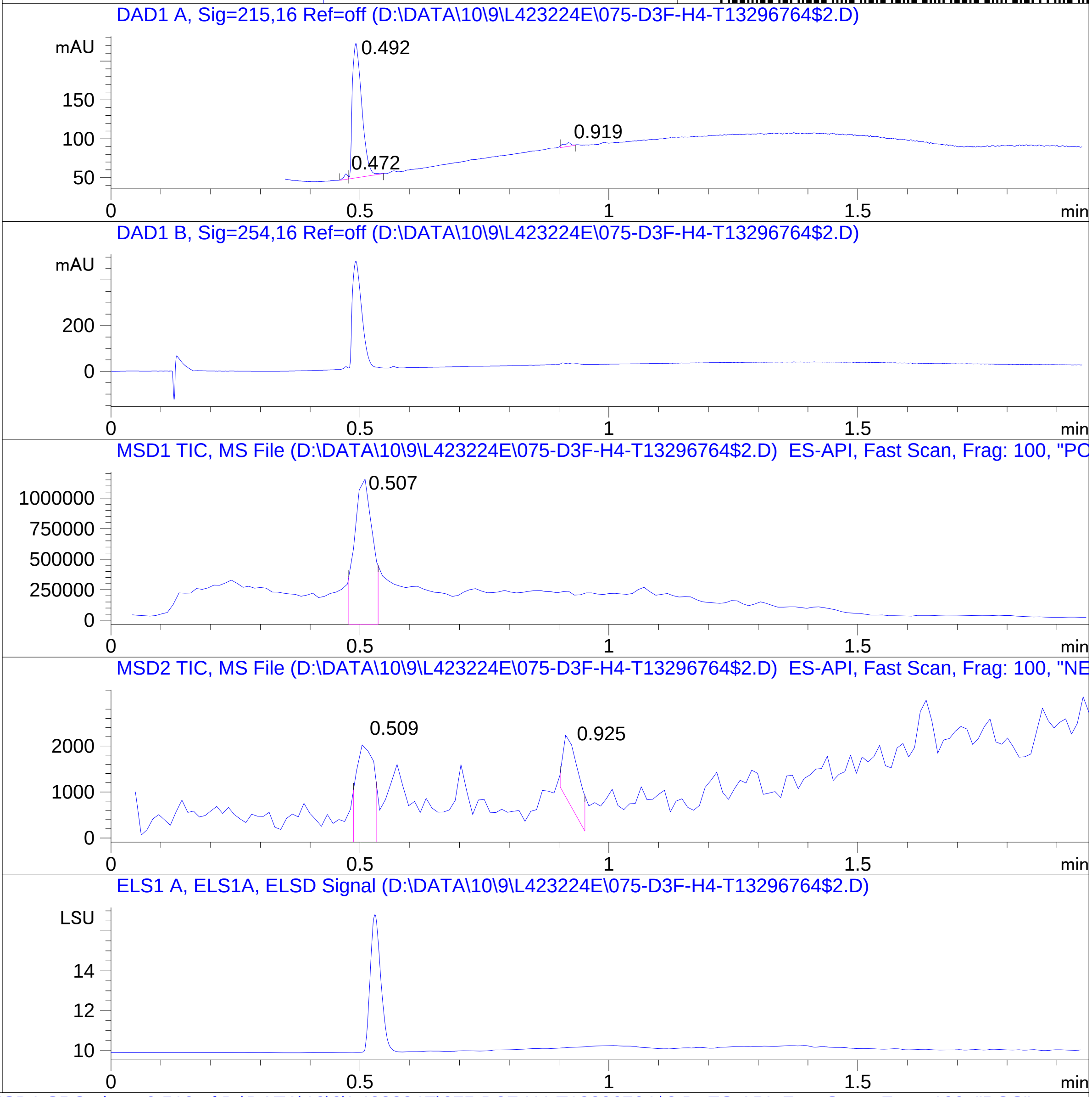

RT 0.507

RT 0.509

RT 0.925

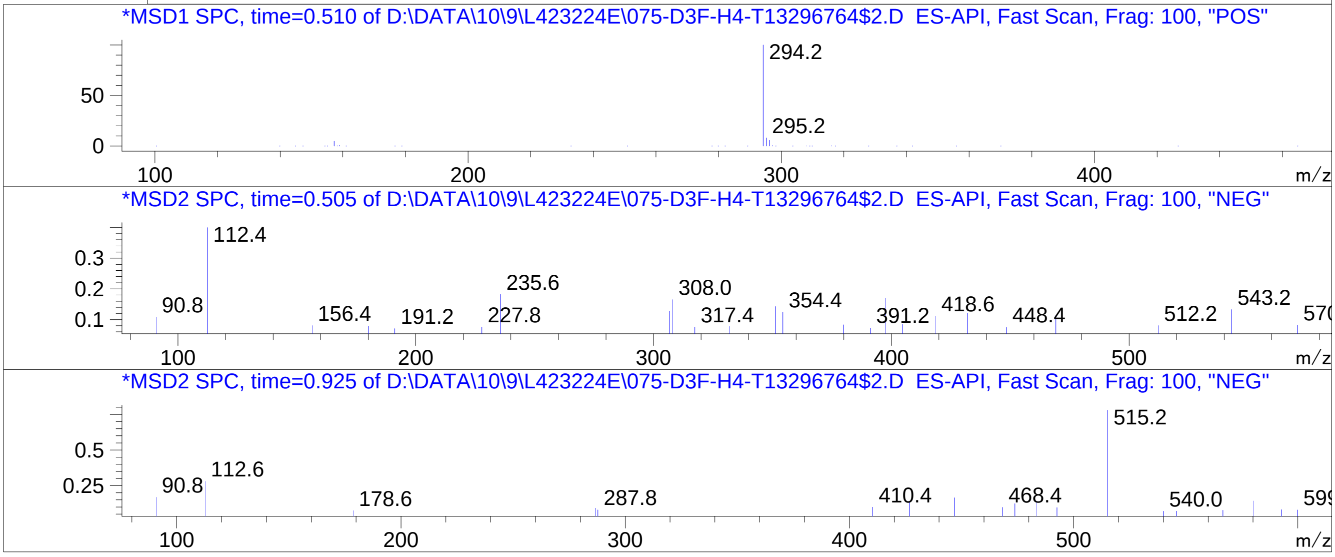

Inj.Date 10/7/2021 LB Acq. Method C:\Users\ -> ->
