# Supplementary material for: AI is a viable alternative to high throughput screening: a 318-target study
Source: Sci Rep. 2024 Apr 2;14:7526. doi: 10.1038/s41598-024-54655-z (PMC10987645; doi:10.1038/s41598-024-54655-z)
Supplement: Supplementary file 1 — Supplementary Information 1. [file 41598_2024_54655_MOESM1_ESM.zip › Nature SREP/QC_AIMS_files/Proj283.PDF]

MaxPeak: 97.10%
Ret_Time: 1.137 min

5633673

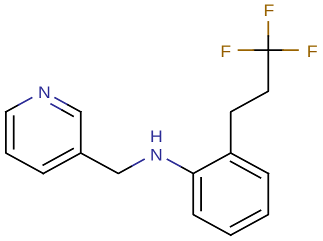

Mol Wt 280.29
Exact Mass 280.15

#	Time	Area%
1	1.137	97.10
2	1.487	2.90

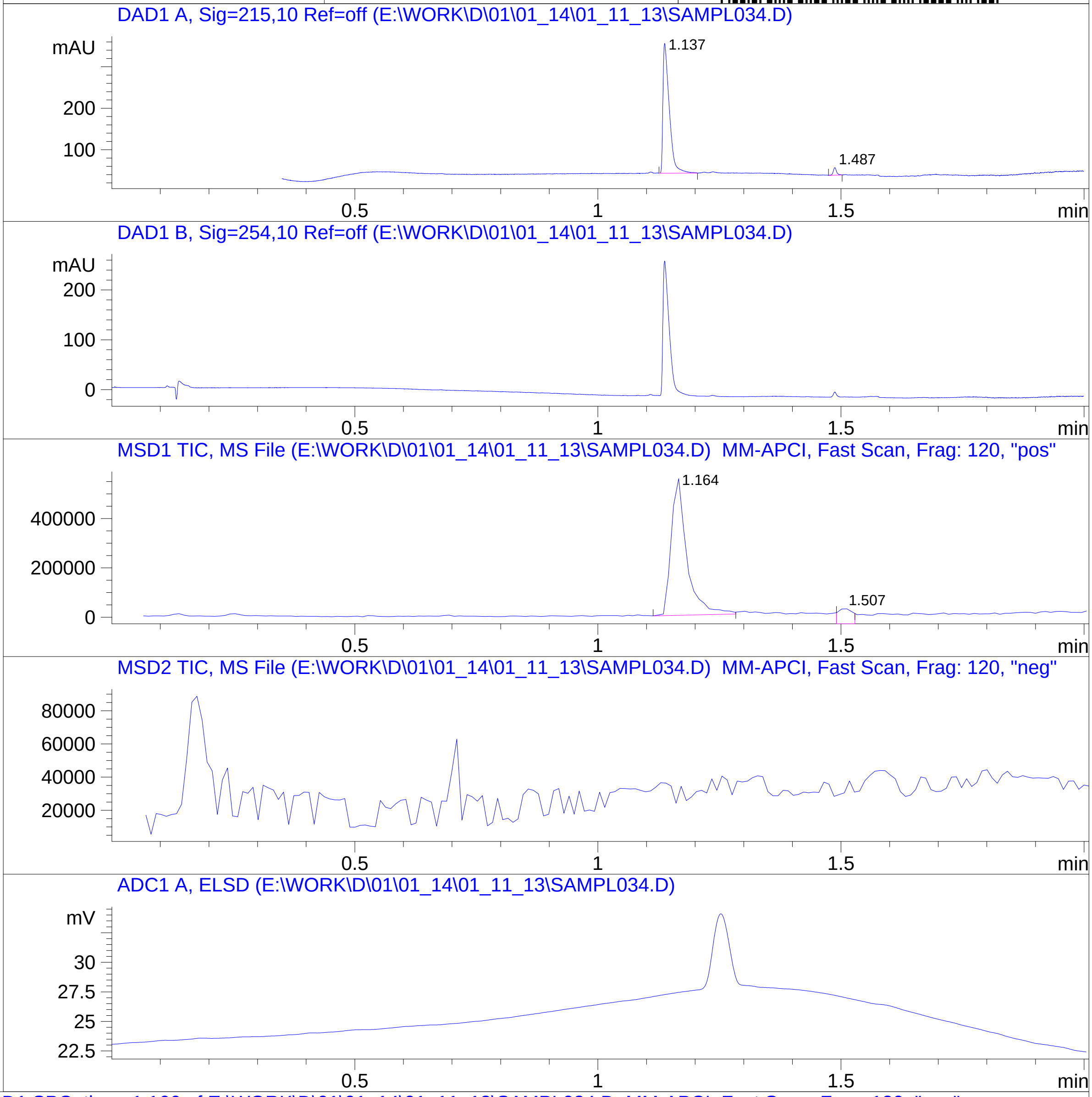

RT 1.164

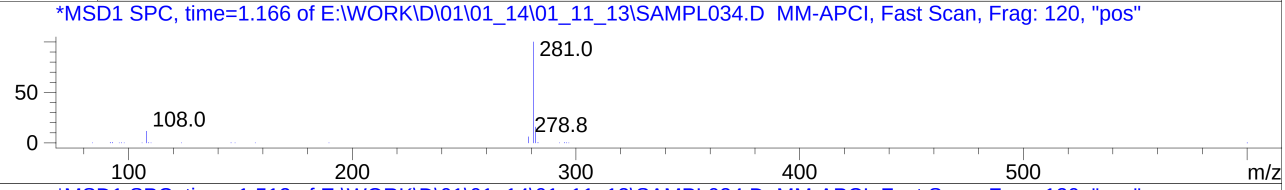

RT 1.507

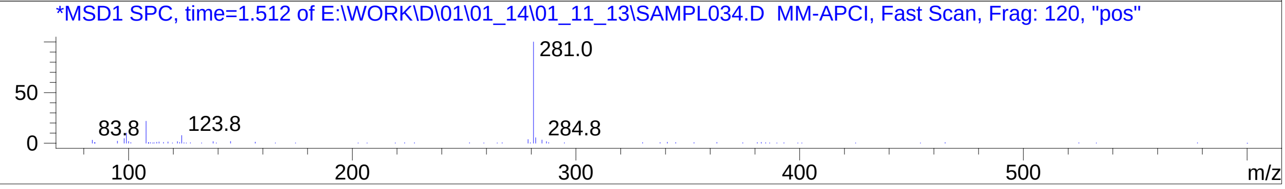

Inj.Date 1/12/2013 K P2-E-01 RR Acq. Method C:\CHEM32\-> ->
