# Supplementary material for: AI is a viable alternative to high throughput screening: a 318-target study
Source: Sci Rep. 2024 Apr 2;14:7526. doi: 10.1038/s41598-024-54655-z (PMC10987645; doi:10.1038/s41598-024-54655-z)
Supplement: Supplementary file 1 — Supplementary Information 1. [file 41598_2024_54655_MOESM1_ESM.zip › Nature SREP/QC_AIMS_files/Proj285.PDF]

MaxPeak: 97.08%
Ret_Time: 1.001 min

5431469

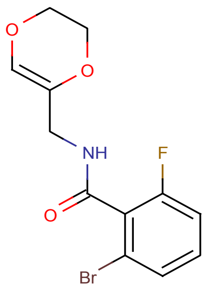

Mol Wt 316.12
Exact Mass 315

#	Time	Area%
1	0.900	1.14
2	0.931	1.78
3	1.001	97.08

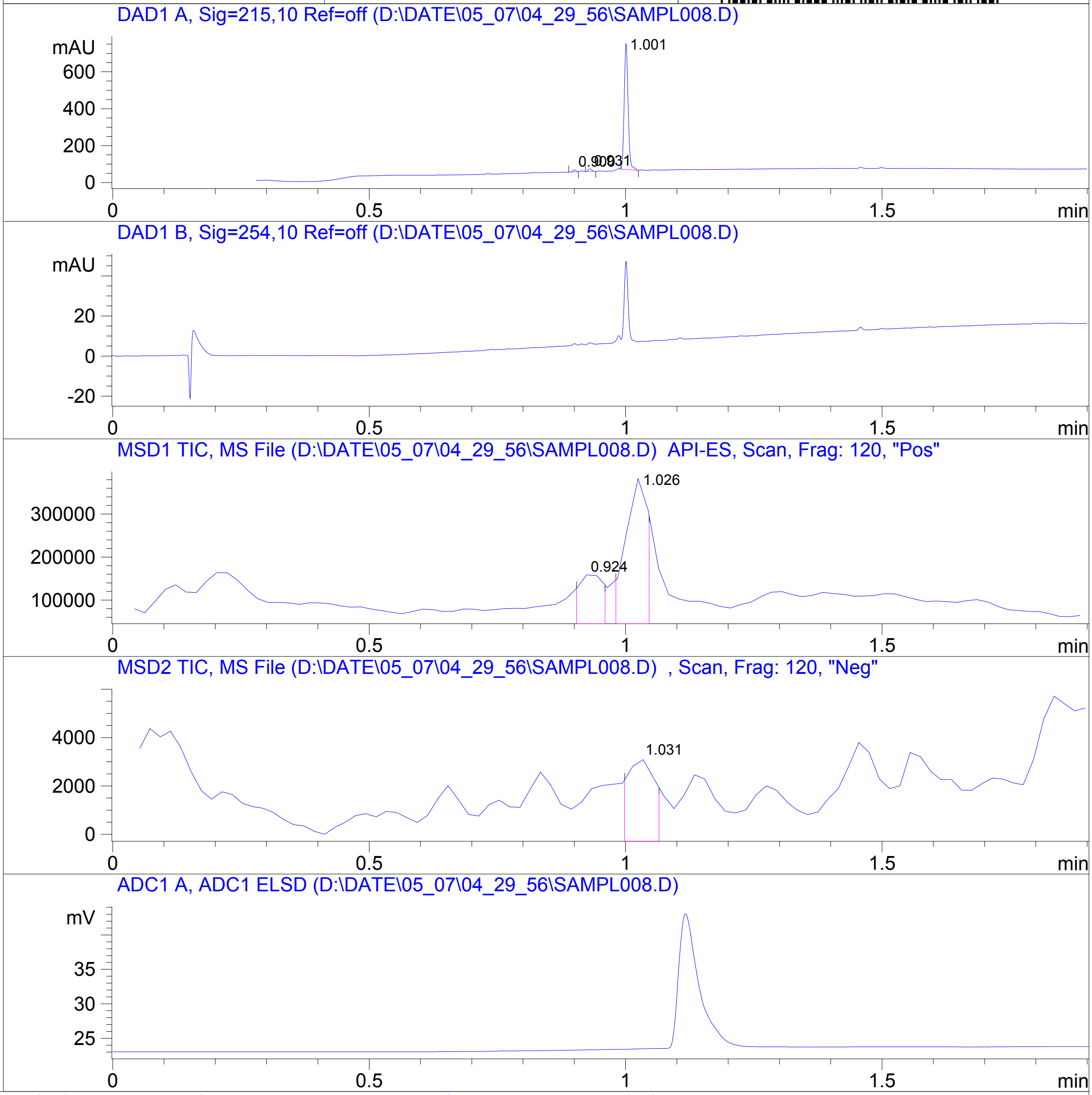

RT 0.924

RT 1.026

RT 1.031

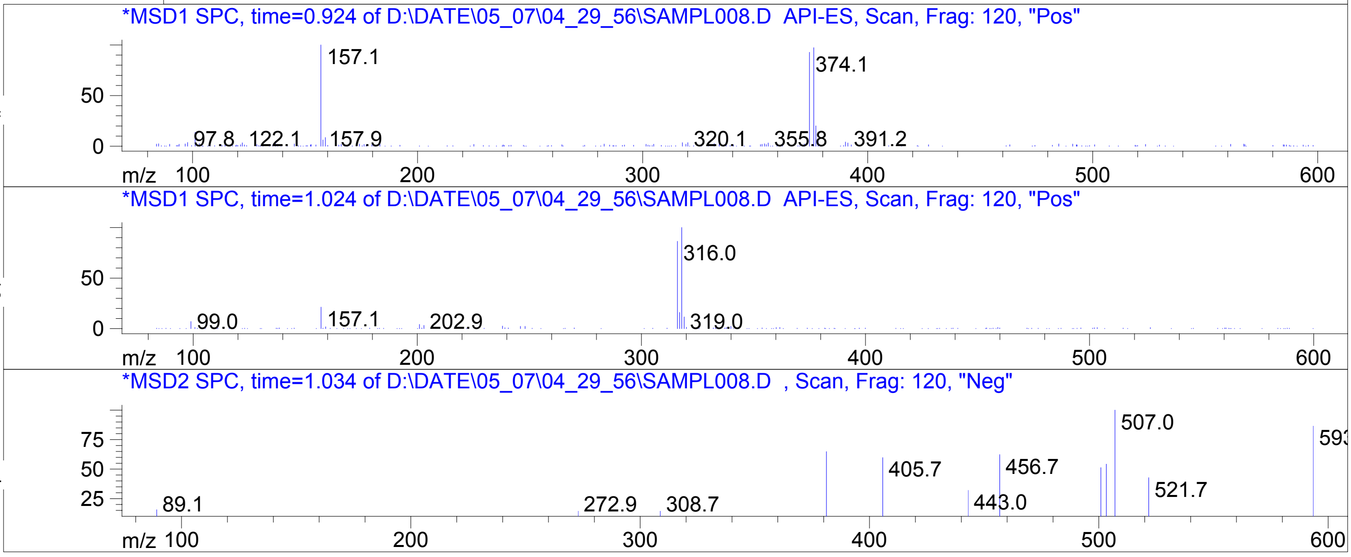

Inj.Date 5/3/2013 L P2-A-08 VL Acq. Method C:\HPCHEM\-> ->
