# Supplementary material for: AI is a viable alternative to high throughput screening: a 318-target study
Source: Sci Rep. 2024 Apr 2;14:7526. doi: 10.1038/s41598-024-54655-z (PMC10987645; doi:10.1038/s41598-024-54655-z)
Supplement: Supplementary file 1 — Supplementary Information 1. [file 41598_2024_54655_MOESM1_ESM.zip › Nature SREP/QC_AIMS_files/Proj286.doc]

```
Data File R:\HPLC\AUTO\1R\ST11018\1HF-4801.D
Sample Name: ST11018P1-H-06
Instrument 1 01/04/2022 11:35:11 AM
Column:  C18 50x2.1mm | 0.80ml/min | Columns Reg Valve
Gradient: "A"->@2.2min->"B"(Hold 0.4min)->@0.01min->"A"(Hold 1.0min)->PostRun
PMP1, Solvent A         : 0.1%TFA in Acn/H2O (2.5:97.5)
PMP1, Solvent B         : 0.1%TFA in AcN
PMP1, Solvent C         : 0.1%FA in Acn/H2O (2.5:97.5)
PMP1, Solvent D         : 0.1%FA in AcN
Ionization mode             :    APCI Positive

Signal 1: ADC1 B, ELSD
Peak RetTime Type  Width     Area      Height     Area
  #   [min]        [min]   [mAu*s]     [mAu]        %
----|-------|----|-------|----------|----------|--------|
   1   2.264 MF    0.0422   93.00948   36.76202 100.0000
Totals :                    93.00948   36.76202


Signal 2: DAD1 A, Sig=300,200 Ref=off
Peak RetTime Type  Width     Area      Height     Area
  #   [min]        [min]   [mAU*s]     [mAU]        %
----|-------|----|-------|----------|----------|--------|
   1   2.170 MF    0.0485  812.23529  279.20313  97.8066
   2   2.318 FM    0.0580   18.21519    5.23238   2.1934
Totals :                   830.45048  284.43551


Signal 3: MSD1 TIC, MS File
Peak RetTime Type  Width     Area      Height     Area
  #   [min]        [min]                            %
----|-------|----|-------|----------|----------|--------|
   1   2.215 MF    0.0936 1.71697e7  3.05697e6  100.0000
Totals :                  1.71697e7  3.05697e6
```
