# Supplementary material for: AI is a viable alternative to high throughput screening: a 318-target study
Source: Sci Rep. 2024 Apr 2;14:7526. doi: 10.1038/s41598-024-54655-z (PMC10987645; doi:10.1038/s41598-024-54655-z)
Supplement: Supplementary file 1 — Supplementary Information 1. [file 41598_2024_54655_MOESM1_ESM.zip › Nature SREP/QC_AIMS_files/Proj291.PDF]

MaxPeak: 100.00%
Ret_Time: 1.278 min

9035435

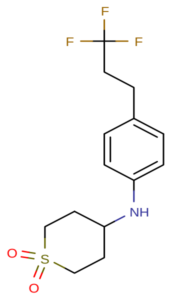

Mol Wt 321.36

Exact Mass 321.13

#	Time	Area%
1	1.278	100.00

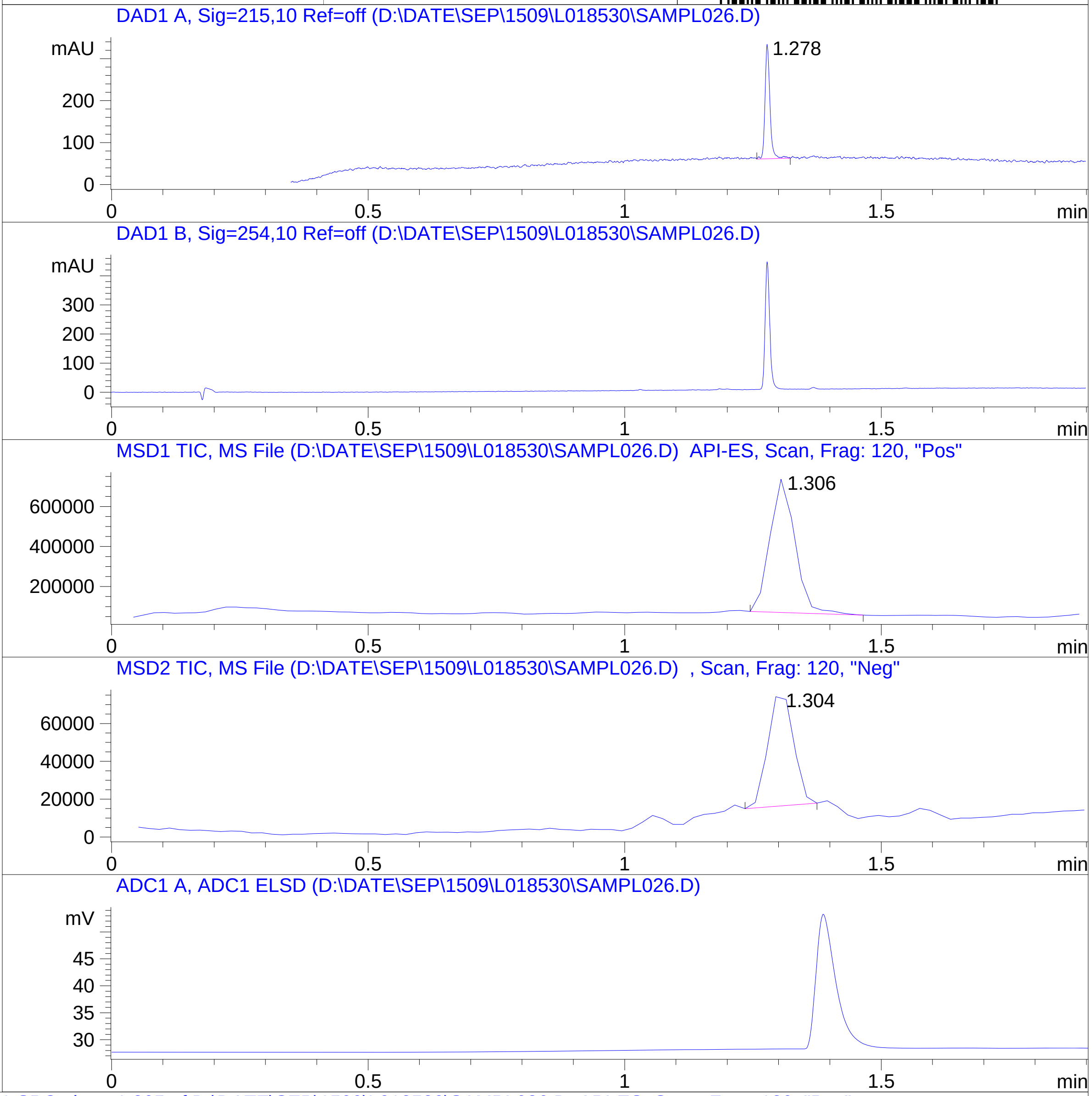

RT 1.306

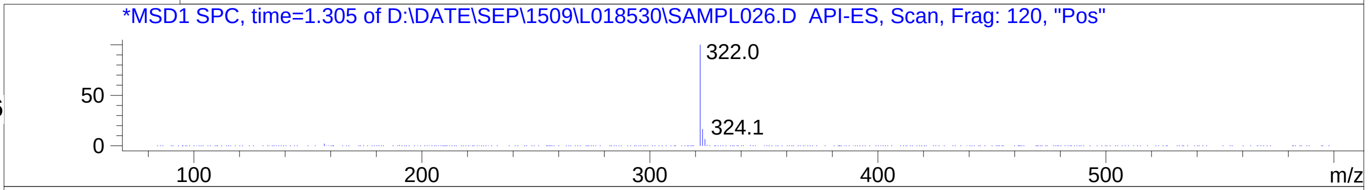

RT 1.304

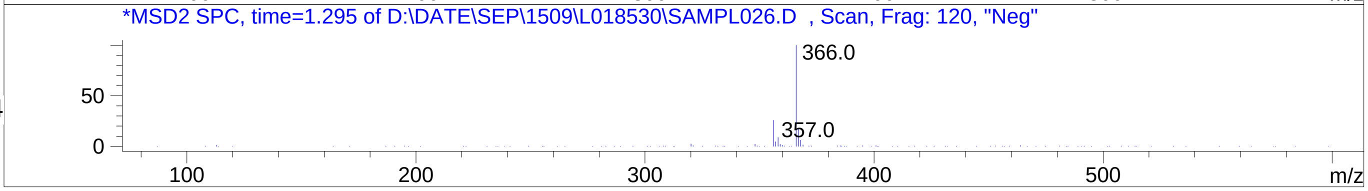

Inj.Date 9/12/2016 A P2-C-09 VL Acq. Method C:\HPCHEM\-> ->
