# Supplementary material for: AI is a viable alternative to high throughput screening: a 318-target study
Source: Sci Rep. 2024 Apr 2;14:7526. doi: 10.1038/s41598-024-54655-z (PMC10987645; doi:10.1038/s41598-024-54655-z)
Supplement: Supplementary file 1 — Supplementary Information 1. [file 41598_2024_54655_MOESM1_ESM.zip › Nature SREP/QC_AIMS_files/Proj293.doc]

```
Data File R:\HPLC\AUTO\ST11180\2BB-5801.D
Sample Name: ST11180P2-B-02
Instrument 1 19/04/2022 12:42:51 PM
Column:  C18 50x2.1mm | 0.80ml/min | Columns Reg Valve
Gradient: "A"->@2.2min->"B"(Hold 0.4min)->@0.01min->"A"(Hold 1.0min)->PostRun
PMP1, Solvent A         : 0.1%TFA in Acn/H2O (2.5:97.5)
PMP1, Solvent B         : 0.1%TFA in AcN
PMP1, Solvent C         : 0.1%FA in Acn/H2O (2.5:97.5)
PMP1, Solvent D         : 0.1%FA in AcN
Ionization mode             :    APCI Positive

Signal 1: ADC1 B, ELSD
Peak RetTime Type  Width     Area      Height     Area
  #   [min]        [min]   [mAu*s]     [mAu]        %
----|-------|----|-------|----------|----------|--------|
   1   2.012 MM    0.0438  817.39148  310.95743 100.0000
Totals :                   817.39148  310.95743


Signal 2: DAD1 A, Sig=300,200 Ref=off
Peak RetTime Type  Width     Area      Height     Area
  #   [min]        [min]   [mAU*s]     [mAU]        %
----|-------|----|-------|----------|----------|--------|
   1   1.357 MM    0.0330  226.15005  114.26944   8.3445
   2   1.825 MM    0.0366   48.86954   22.24111   1.8032
   3   1.941 MM    0.0437 2435.15332  929.32947  89.8523
Totals :                  2710.17291 1065.84002


Signal 3: MSD1 TIC, MS File
Peak RetTime Type  Width     Area      Height     Area
  #   [min]        [min]                            %
----|-------|----|-------|----------|----------|--------|
   1   1.998 MM    0.0890 1.88780e7  3.53628e6  100.0000
Totals :                  1.88780e7  3.53628e6
```
