# Supplementary material for: AI is a viable alternative to high throughput screening: a 318-target study
Source: Sci Rep. 2024 Apr 2;14:7526. doi: 10.1038/s41598-024-54655-z (PMC10987645; doi:10.1038/s41598-024-54655-z)
Supplement: Supplementary file 1 — Supplementary Information 1. [file 41598_2024_54655_MOESM1_ESM.zip › Nature SREP/QC_AIMS_files/Proj294.pdf]

MaxPeak: 95.24%
Ret_Time: 1.335 min

T5965059

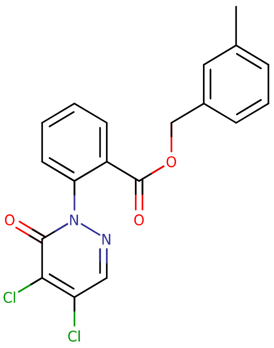

Mol Wt 389.23

Exact Mass 388.05

#	Time	Area%
1	0.880	2.27
2	1.335	95.24
3	1.743	2.49

DAD1 A, Sig=215,16 Ref=off (D:\DATA\0525\L489832R\003-D2B-A3-T5965059.D)

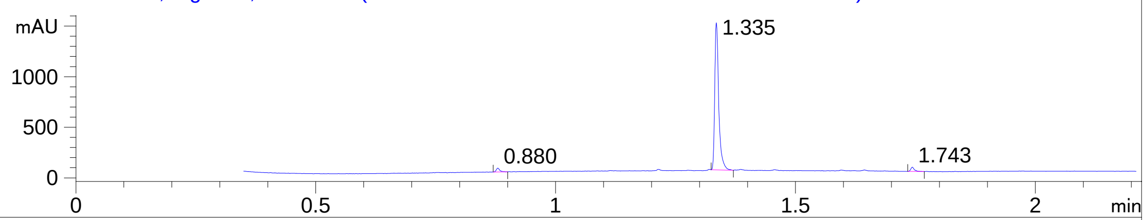

DAD1 B, Sig=254,16 Ref=off (D:\DATA\0525\L489832R\003-D2B-A3-T5965059.D)

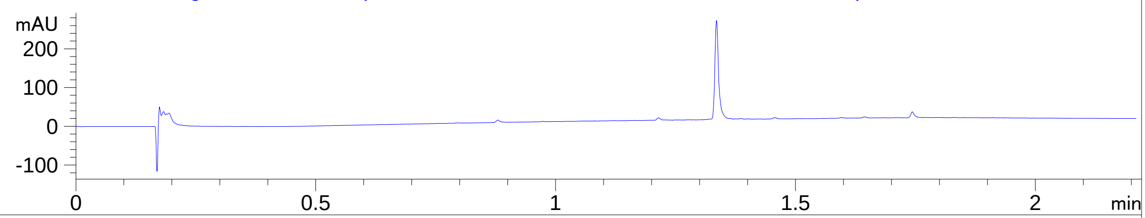

MSD1 TIC, MS File (D:\DATA\0525\L489832R\003-D2B-A3-T5965059.D) ES-API, Fast Scan, Frag: 100, "POS"

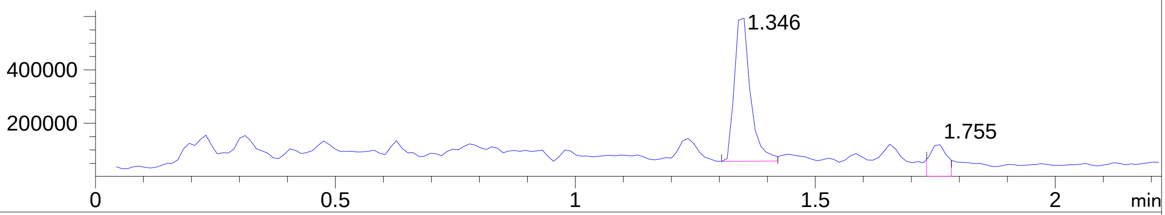

MSD2 TIC, MS File (D:\DATA\0525\L489832R\003-D2B-A3-T5965059.D) ES-API, Fast Scan, Frag: 100, "NEG"

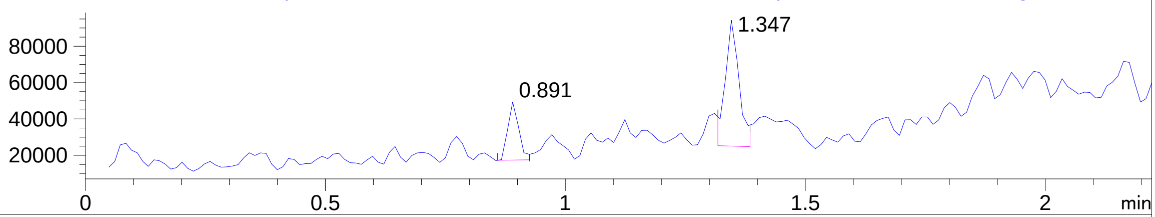

ELS1 A, ELS1A, ELSD Signal (D:\DATA\0525\L489832R\003-D2B-A3-T5965059.D)

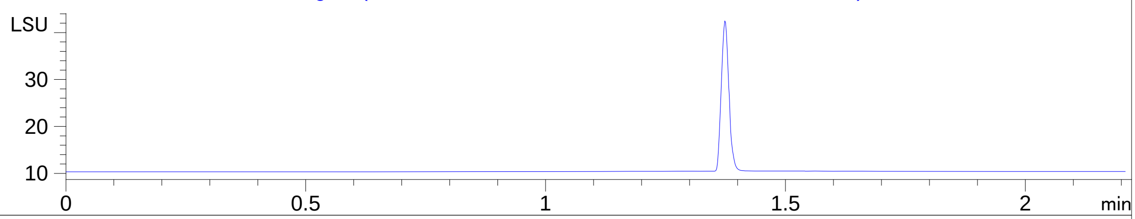

*MSD1 SPC, time=1.352 of D:\DATA\0525\L489832R\003-D2B-A3-T5965059.D ES-API, Fast Scan, Frag: 100, "POS"

RT 1.346

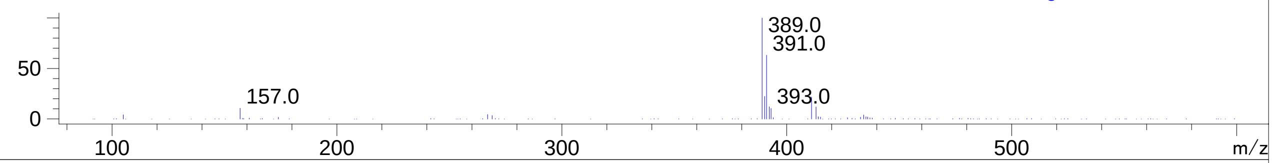

*MSD1 SPC, time=1.760 of D:\DATA\0525\L489832R\003-D2B-A3-T5965059.D ES-API, Fast Scan, Frag: 100, "POS"

RT 1.755

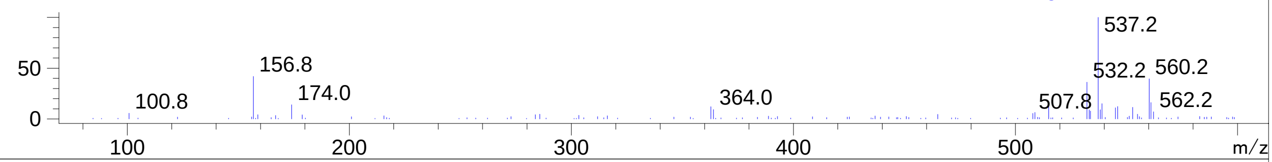

*MSD2 SPC, time=0.890 of D:\DATA\0525\L489832R\003-D2B-A3-T5965059.D ES-API, Fast Scan, Frag: 100, "NEG"

RT 0.891

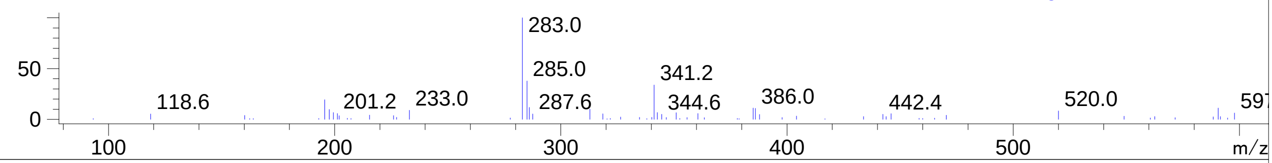

*MSD2 SPC, time=1.346 of D:\DATA\0525\L489832R\003-D2B-A3-T5965059.D ES-API, Fast Scan, Frag: 100, "NEG"

RT 1.347

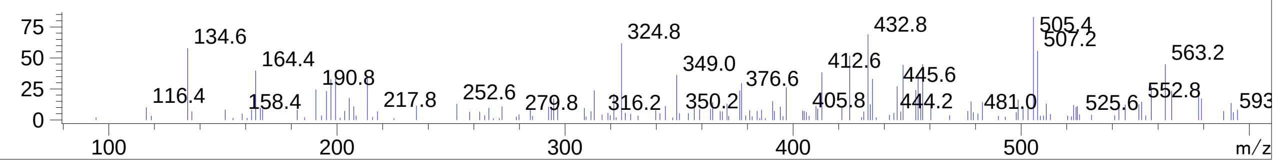

Inj.Date 5/24/2022   OA   Acq. Method C:\Users\ -> ->
