# Supplementary material for: AI is a viable alternative to high throughput screening: a 318-target study
Source: Sci Rep. 2024 Apr 2;14:7526. doi: 10.1038/s41598-024-54655-z (PMC10987645; doi:10.1038/s41598-024-54655-z)

T6801402

MaxPeak: 98.02%
Ret_Time: 1.453 min

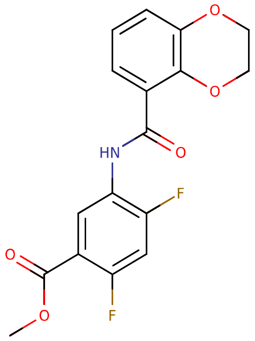

Mol Wt 349.29

Exact Mass 349.08

#	Time	Area%
1	0.975	1.98
2	1.453	98.02

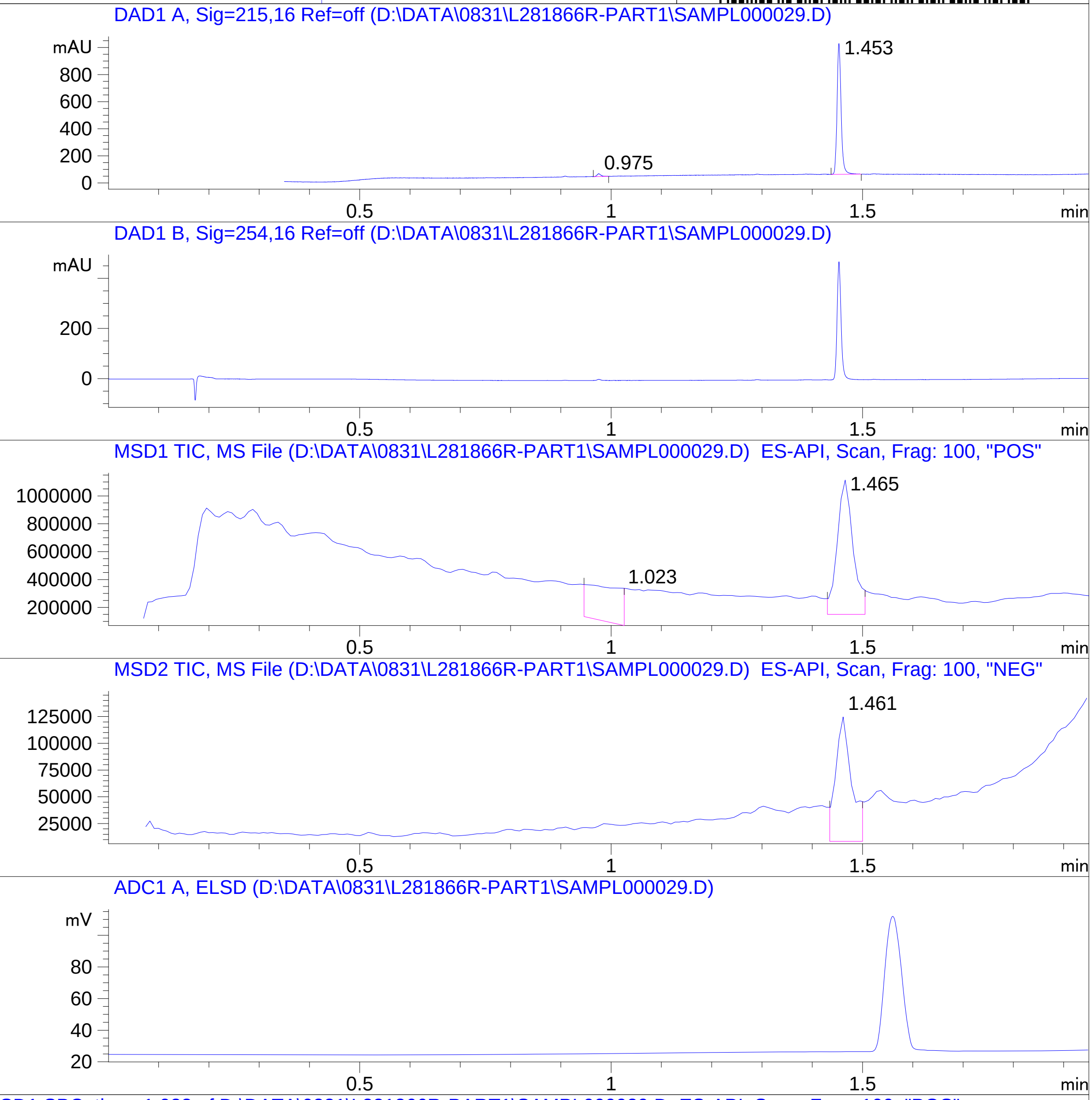

RT 1.023

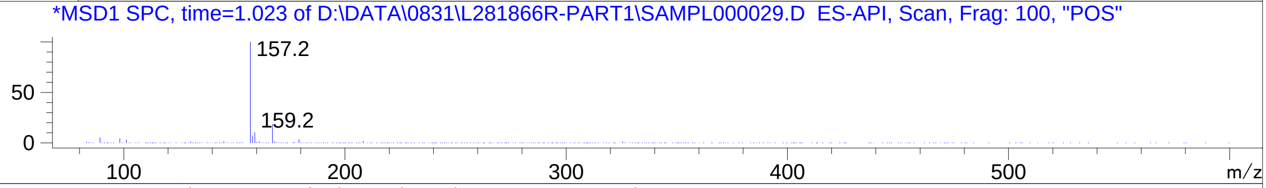

RT 1.465

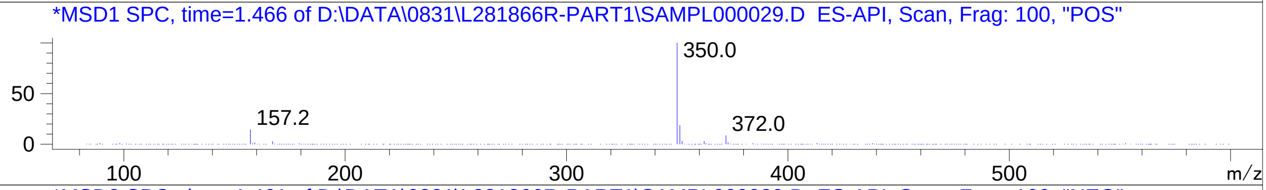

RT 1.461

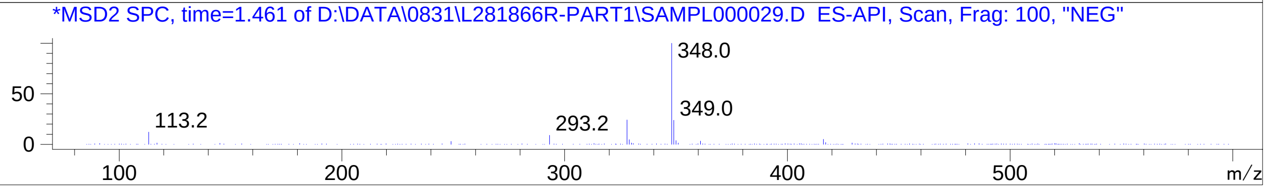

Supplement: Supplementary file 1 — Supplementary Information 1. [file 41598_2024_54655_MOESM1_ESM.zip › Nature SREP/QC_AIMS_files/Proj295.pdf]
